# Chemical recycling of hydrofluorocarbons by transfer fluorination

**DOI:** 10.1038/s41557-026-02096-8

**Published:** 2026-03-13

**Authors:** Niko A. Jenek, Sarah L. Brock, Jiahuang Mao, Amanda A. Fogh, Andreas Phanopoulos, Mark R. Crimmin

**Affiliations:** 1 Department of Chemistry, Imperial College London, London, UK; 2 Department of Chemistry, University of Bath, Bath, UK

**Keywords:** Chemical synthesis, Green chemistry, Inorganic chemistry

## Abstract

Fluorochemicals improve our quality of life; however, there is increasing concern over how they are produced and their negative effects on health and the environment. Here we report an approach to the recycling of fluorochemicals. Treatment of hydrofluorocarbons with a potassium base (KHMDS or KO^*t*^Bu) results in rapid defluorination to produce anhydrous potassium fluoride. This potassium fluoride can then be used to prepare a wide range of fluorinated organic and inorganic molecules, including sulfonyl fluorides, aryl fluorides, alkyl fluorides and a range of *p*-block fluorides, in an overall one-pot transfer fluorination process. The scope of fluorochemicals that can be recycled by transfer fluorination includes industrially relevant refrigerants (hydrofluorocarbons), hydrofluoroolefins, fluoroethers—including anaesthetics and battery additives—perfluorooctanoic acid and poly(vinylidene) difluoride. Aspects of the transfer fluorination mechanism have been investigated using density functional theory calculations, and approaches to scale up using batch (50 g) and flow (1.5 g h^−1^) chemistry are presented.

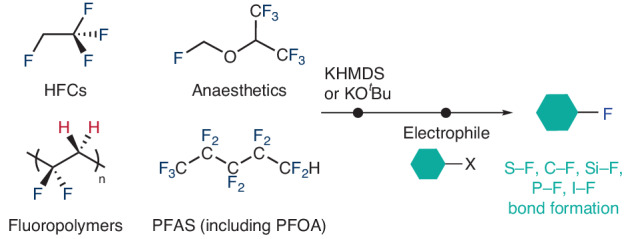

## Abstract (web summary)

Fluorochemicals improve our quality of life, but there is an increasing concern over their production and the negative impact on health and the environment. Now chemical recycling of fluorochemicals via base-induced defluorination has been demonstrated, and the resulting potassium fluoride has been used to synthesize organic and inorganic compounds.

## Main

Fluorine-containing molecules and materials are embedded in modern society and improve our quality of life. They find applications as refrigerants, foaming agents, insulating materials, coatings, active pharmaceutical ingredients and agrochemicals^1^. Fluorocarbons have historically been created from fluorspar (CaF_2_) through generation of the toxic and corrosive intermediate hydrofluoric acid. Elegant methods have emerged, however, for the direct production of fluorocarbons from fluorspar^2,3^. Nevertheless, fluorspar is classified as a critical raw material and fluorine-containing products are often single use and many end up in the environment, where they can contribute to climate breakdown and negative health outcomes^4^.

For example, third generation refrigerants are volatile hydrofluorocarbons (HFCs) that have long atmospheric lifetimes and high global warming potentials, meaning that they effectively contribute to climate change^5^. Per(fluoroalkyl) and poly(fluoroalkyl) substances (PFAS), such as perfluorooctanoic acid (PFOA), are damaging to health and can potentially contaminate water supplies and food chains^6^. Concerns over the negative impact of fluorochemicals has led to the proposal of REACH (registration, evaluation, authorization and restriction of chemicals) legislation, which would categorize all compounds containing –CF_3_ and –CF_2_– groups (when not connected to H, Cl, Br or I atoms) as PFAS^7^. If we are to continue to rely on fluorochemicals to improve our quality of life, we must improve sustainability of the sector^8–11^.

Very recently, major breakthroughs have been made in the chemical recycling of fluorocarbons^12^. Treatment of non-volatile PFAS, including fluorinated polymers, with potassium salts or sodium metal under mechanochemical conditions allows the production of group 1 fluoride salts that can be used as fluorinating agents^13–15^. These studies have built on proof-of-concept work in which fluoride was harvested from ‘chemically inert’ fluorocarbons and delivered to another molecule^16–20^. Anhydrous fluoride reagents have also been prepared from specialist, reactive, fluorocarbons (for example, hexafluorobenzene and pentafluoropyridine)^21,22^.

Despite these recent developments, no broad recycling approach that can be applied to volatile HFCs currently exists. This is notable as fluorinated gases, including third and fourth generation refrigerants, account for a substantial quantity of the fluorochemicals manufactured, with legacy stocks expected to be created as use is phased down^11^. Volatile HFCs may not be easily compatible with mechanochemical conditions and as such complementary approaches to their recycling are highly desirable.

**Fig. 1:**
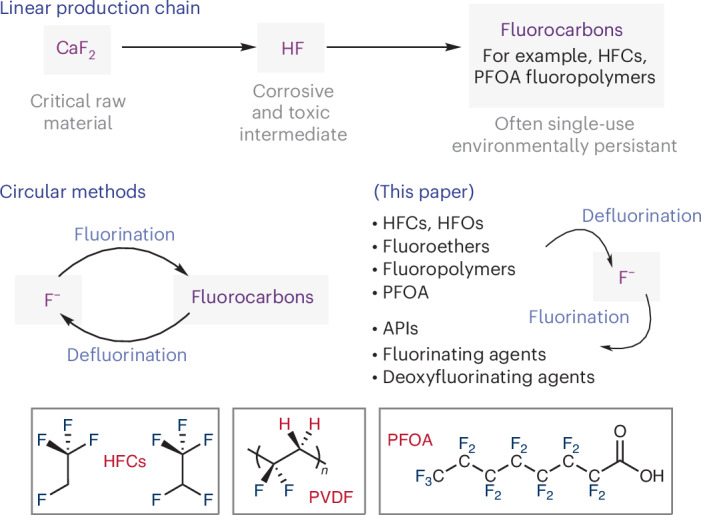
Linear and circular production chains for fluorocarbons. API, active pharmaceutical ingredient; HFOs, hydrofluoroolefins.

In this paper, we report a simple transfer fluorination method that allows the fluorine content of HFCs to be harvested in the form of anhydrous KF and then used in the onwards synthesis of fluorochemicals (Fig. 1). The approach can be readily applied to the chemical recycling of volatile HFCs, hydrofluoroolefins, anaesthetics, battery technology cosolvents, low molecular weight PFAS as well as PFOA and the widely used fluoropolymer poly(vinylidene) difluoride (PVDF). These materials can be used as latent fluorine sources for production of a wide range of fluorochemicals, marking an important contribution to sustainable and circular fluorine chemistry.

**Fig. 2:**
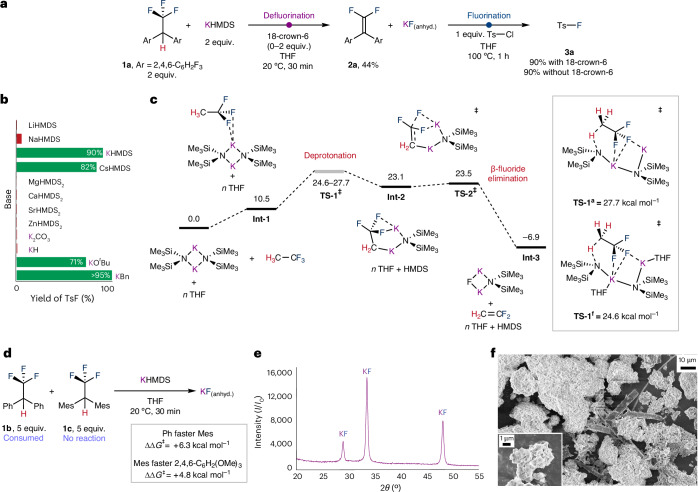
Discovery and mechanistic investigation of a transfer fluorination process. **a**, Synthetic procedure for the one-pot transfer fluorination procedure using **1a** as a fluorine source and TsCl as an acceptor. **b**, Comparison of yields of TsF from transfer fluorination from **1a** with different group 1 and 2 bases. **c**, DFT calculated pathway for the defluorination of **1a** with dimeric KHMDS via deprotonation and β-fluoride elimination transition states. Gibbs energies in kcal mol^−1^. B3PW91-D3(BJ)/def2-QZVPPD/SMD (THF)/goodvibes//B3PW91-D3(BJ)//def2-TZVP[C,H]/def2-TZVPP[N,O,F,Si,K]/SMD (THF). **d**, Competition experiment between **1b** and **1c** along with calculated ΔΔ*G*^‡^_298K_ value for TS-1 for **1b** versus **1c** and **1c** versus **1d**. **e**, Powder X-ray diffraction data of in situ generated KF. **f**, SEM of in situ generated KF. Ts, tosyl; TS, transition state; anhyd., anhydrous; Int, intermediate; Mes, mesityl or 1,3,5-Me_3_C_6_H_2_; OMe, methoxy; O^*t*^Bu, *tert*-butoxy; SMD, solvation model density. Source data

Compound **1a** was investigated as a model for transfer fluorination^23^. Treatment of **1a** with 1.5 equiv. of potassium hexamethyldisilazide (KHMDS) in the presence of 18-crown-6 in tetrahydrofuran (THF) at 25 °C resulted in a fast defluorination reaction to form **2a** (ref. ^24^). Subsequent addition of *p*-tolylsulfonyl chloride (TsCl) to the product mixture and heating to 100 °C for 60 minutes led to transfer fluorination and generation of *p*-tolylsulfonyl fluoride (TsF) in 90% yield (Fig. 2a). The reaction proceeds just as efficiently in the absence of 18-crown-6. A series of bases related to KHMDS were investigated for the transfer fluorination reaction with optimal yields of TsF formed with KO^*t*^Bu (71%), KBn (>95%) and CsHMDS (82%). The efficiency proved dependent on the nature of the counteraction with alternative group 1, 2 and 12 metals (for example, Li, Na, Mg, Ca, Sr and Zn) proving less effective than potassium and caesium (Fig. 2b). This can be rationalized on the basis of the comparison of lattice enthalpies and enthalpies of dissolution of metal fluorides^25^, with heavier group 1 salts such as KF and CsF having the most favourable thermodynamics for onwards reactions.

Density functional theory (DFT) calculations were carried out to gain insight into the defluorination step. The reaction of CH_3_CF_3_ (a simplified model of **1a**) with KHMDS was studied using the B3PW91-D3(BJ) functional with a hybrid basis set (Fig. 2c). The speciation of group 1 hexamethyldisilazide (HMDS, or N(SiMe_3_)_2_) bases in solution has been considered previously^26^. The Gibbs free energies of monomeric, dimeric and trimeric aggregation states of KHMDS were calculated alongside analogues with explicit solvation by THF. On the basis of the relative stability of these species, the ground state in THF solution was assumed to be the non-solvated dimer [KHMDS]_2_, with solvated analogues containing one, two, or three THF molecules accessible but slightly higher in energy (Δ*G*°_298K_ = 0.2–4.4 kcal mol^−1^). Reaction of [KHMDS]_2_ with CH_3_CF_3_ was calculated to occur through initial formation of a weakly bound encounter complex **Int-1** (Δ*G*°_298K_ = +10.5 kcal mol^−1^) in which CH_3_CF_3_ is associated with one of the K^+^ ions through an apparent *κ*^2^-binding of the CF_3_ group by electrostatic K–F interactions. **Int-1** evolves to **TS-1**, in which the C–H bond of CH_3_CF_3_ is deprotonated by one of the basic amide groups of KHMDS to generate **Int-2** (ref. ^27^). The role of explicit solvation was considered in this step, and a series of structures of **TS-1** with varying THF coordination to K^+^ considered. All identified transition states are close in energy ranging from Δ*G*^‡^_298K_ = 24.6 to 27.7 kcal mol^−1^. The lowest energy isomer contains one THF coordinated at each K^+^ site, the highest is the non-solvated species. These calculations strongly suggest that THF plays a role in facilitating the C–H deprotonation but leads to only a small stabilization of transition state geometries. **Int-2** is an unstable organometallic intermediate (Δ*G*°_298K_ = 23.1 kcal mol^−1^) that can undergo extremely facile β-fluoride elimination via **TS-2** (Δ*G*^‡^_298K_ = 23.5 kcal mol^−1^).

The overall sequence results in a 1,2-elimination from CH_3_CF_3_ to form CH_2_=CF_2_ alongside **Int-3** and HMDS. Alternative pathways involving both solvated and non-solvated monomeric [KHMDS] as a reagent were found to be inaccessible (Δ*G*^‡^_298K_ > 35 kcal mol^−1^ for deprotonation)^28^. The calculations predict that the C–H deprotonation step should be rate-limiting. As such the electronics (p*K*_a_) of the fluorine donor should influence reactivity. Competition experiments support this hypothesis with more electron-deficient analogues of **1a** reacting faster than more electron-rich ones (Fig. 2d).

While in the computational model KF is generated as an adduct with a further equivalent of KHMDS for example as **Int-3**, this species is probably unstable with respect to formation of KF(s) due to its high lattice enthalpy. During the course of the reaction of 1,2,2,2-tetrafluoroethane (HFC-134a) and KHMDS, a fine precipitate assumed to be anhydrous KF was observed. Isolation and redissolving this precipitate in D_2_O gave a diagnostic ^19^F resonance at *δ* = −122.17 ppm, consistent with that expected for KF in aqueous solution (commercial KF −122.04 ppm). Solid state ^19^F NMR spectroscopy confirmed this to be the only fluorine-containing product present in the precipitate. Further characterization was carried out by powder X-ray diffraction and scanning electron microscopy (SEM) and confirmed that the isolated solid contained anhydrous KF (Fig. 2e,f). The SEM images show an additional amorphous component, likely to be carbon-based, and microanalysis of the solid suggests that it is composed of not only KF but also a very small quantity of carbon. Solid state ^13^C NMR spectroscopy suggests a low concentration of both *sp*^3^ and *sp*^2^ carbon environments. The amount of KF produced was measured through the direct reaction of KHMDS with 1,2,2,2-tetrafluoroethane (HFC-134a), followed by extraction in D_2_O and quantification against an internal standard. These experiments demonstrated near quantitative formation of KF (93%) using a 2.5:1 ratio of 1,2,2,2-tetrafluoroethane:KHMDS. The isolated KF proved competent for the fluorination of TsCl to form TsF in 75% yield.

The scope of molecules capable of acting as fluorine donors in this transformation was expanded to include electron-rich and electron-deficient (2,2,2-trifluoroethane-1,1-diyl)-dibenzenes (**1a**–**1d**), 2,2,2-trifluoroethylbenzene (**1e**), (3,3,3-trifluoropropyl)benzene (**1f**) and 3,3,3-trifluoropropanenitrile (**1g**). Several industrially relevant fluorocarbons were then studied in transfer fluorination. The scope incorporates a series of HFCs including 2,2-difluoroethane (**1h**, HFC-152a) 2,2,2-trifluoroethane (**1i**, HFC-143a), 1,2,2,2-tetrafluoroethane (**1j**, HFC-134a) and 1,1,1,2,2-pentafluoroethane (**1k**, HFC-125). The last two HFCs are the largest use refrigerants in circulation and are set to be phased down in the next decade, creating legacy stocks for disposal or chemical recycling. Similarly, next generation refrigerant 2,3,3,3-tetrafluoropropene (**1l**) could be used as a fluorine source to form TsF in the one-pot two-step process. Fluorinated ethers are also effective fluorine atom donors in transfer fluorination, with the scope including currently and formerly used anaesthetics sevoflurane (**1m**), isoflurane (**1n**) and enflurane (**1o**), along with a handful of high-performing electrolyte cosolvents (**1p**–**1r**) used in battery technologies^29,30^. Fluorinated anaesthetics are of increasing concern due to their high global warming potentials, and some health services are already taking steps to phase down their use. The fluorination methodology could also be extended to include volatile PFAS as fluorine donors (**1s**–**1t**). The are some small limitations in scope, 1-fluorohexane, trifluoro(methyl)cyclohexane and selected α-fluoroesters did not participate in transfer fluorination.

**Fig. 3:**
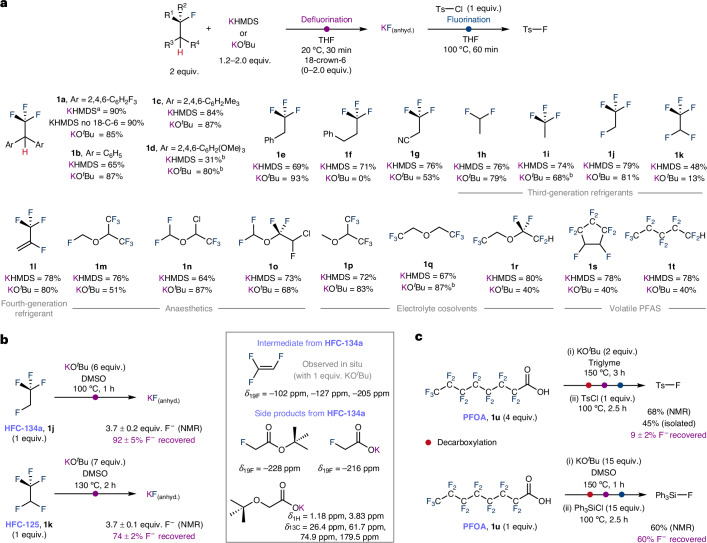
Scope of fluorocarbons used as fluorine sources in transfer fluorination. **a**, Synthetic procedure for the one-pot transfer fluorination procedure using fluorocarbons as the fluorine source and TsCl as an acceptor. **b**, Defluorination of HFC-134a and HFC-125 and identification of intermediates and side products. **c**, Tandem decarboxylation and transfer fluorination using PFOA. Yields of products based on full conversion of the limiting reagent, measured by ^19^F NMR spectroscopy using 1,2-difluorobenzene or NaOTf as an internal standard. ^a^Unless otherwise indicated reactions with KHMDS were carried out with 2 equiv. of 18-crown-6 present. ^b^The defluorination step was carried out at elevated temperature of 60 °C or 100 °C. Ts, tosyl; OTf, triflate.

The coproducts of defluorination of HFCs could clearly be identified in crude reaction mixtures by ^19^F NMR spectroscopy. Hence, transfer fluorination using 2,2-difluoroethane (**1h**) forms fluoroethene, while 1,2,2,2-tetrafluoroethane (**1j**) generates 1,2,2-trifluoroethene. These fluoroalkenes are known monomers used in the preparation of fluorinated polymers. There is an important safety consideration, however, as these fluoroalkenes are potentially highly toxic and explosive, hence it is desirable to also find conditions for complete destruction of the HFC with recycling of its total fluorine content^31^. Reaction of 1,2,2,2-tetrafluoroethane (**1j**) with 6 equiv. of KO^*t*^Bu in batch conditions led to the formation of 3.7 ± 0.2 equiv. of KF corresponding to 92 ± 5% recovery of the available fluorine content. 1,2,2-trifluoroethene is not present at the end of the reaction (Fig. 3b). Several carbon-containing side products could be identified by ^1^H, ^13^C and ^19^F NMR spectroscopy, including *tert*-butyl 2-fluoroacetate, potassium 2-fluoroacetate and potassium 2-(*tert*-butoxy)acetate. Similar reaction of pentafluoroethane (**1k**) with 7 equiv. of KO^*t*^Bu led to near complete defluorination with production of 3.7 ± 0.1 equiv. of KF corresponding to 74 ± 2% recovery of the available fluorine content, with no tetrafluoroethene present at the end of the reaction.

PFOA is one of the most prominent PFAS of environmental concern. This compound does not contain the 1,2-vicinal H–C–C–F functional group essential to the proposed defluorination mechanism. Nevertheless, PFOA was considered a viable fluorine donor as it is known to undergo thermal decarboxylation to form 1H-perfluoroheptane under basic conditions^32,33^. Reaction of 4 equiv. of PFOA with 2 equiv. of KO^*t*^Bu in triglyme at 150 °C for 3 h, followed by addition of TsCl, led to transfer fluorination producing TsF in 68% in situ and 45% isolated yield (Fig. 3c). Due to concerns over the generation of PFAS containing side products in this reaction, conditions were again sought for total destruction and fluoride recovery. Hence, reaction of 1 equiv. PFOA with 15 equiv. of KO^*t*^Bu in dimethylsulfoxide (DMSO) at 150 °C for 1 h led to formation of 12 ± 2 equiv. of KF corresponding to 80 ± 13% fluoride recovery. Examination of the soluble organic products by ^19^F NMR spectroscopy confirmed that no PFAS remains at the end of the reaction. The solution containing KF_(DMSO)_ could be used to directly fluorinate triphenylsilane chloride in 60% yield based on the 15 fluorine atoms available in PFOA as the limiting reagent.

**Fig. 4:**
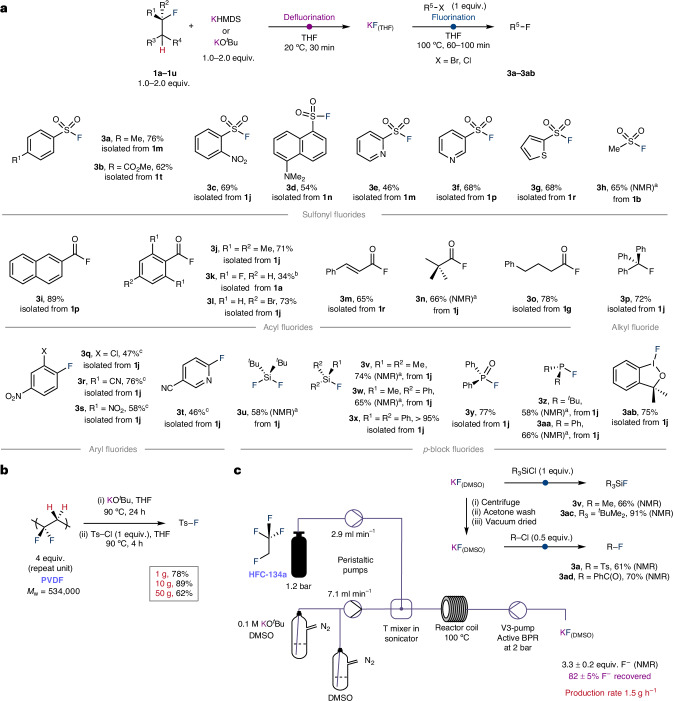
Scope and scalability of transfer fluorination process. **a**, Synthetic procedure for the one-pot transfer fluorination procedure showing the scope of fluorination products accessible. The fluorine donor (F^−^) is given by compound number. Unless stated otherwise isolated yields based on full conversion of the limiting reagent are given for each entry. **b**, Scaled-up transfer fluorination using PVDF as a fluorine donor. **c**, Scaled-up transfer fluorination using HFC-134a in flow. ^a^Due to volatility of the product an in situ yield measured by ^19^F NMR spectroscopy was recorded. ^b^Isolated yield was limited by the poor stability of this compound on silica paired with very high volatility. ^c^Fluorination in DMSO between 130 °C and 140 °C. *M*_w_, weight-average molecular weight of the polymer.

The transfer fluorination methodology could be used to construct a range of valuable products through use of the recycled anhydrous KF. It appears this anhydrous solid shows excellent reactivity in fluorination methodology, probably due to its high surface area. Onwards reactions with electrophiles could be used to construct sulfonyl fluorides (**3a**–**3h**)^34^, acyl fluorides (**3i**–**3o**)^35^, alkyl and aryl fluorides (**3p**–**3t**), along with a variety of *p*-block fluorides encompassing Si–F, P–F and I–F (**3u**–**3ab**) bond formation (Fig. 4a). To showcase the potential of transfer fluorination in the repurposing of fluorocarbons, products were obtained using primarily third-generation HFC refrigerants as the fluorine donors, but also using anaesthetics, battery additives, volatile PFAS substances and a fluorinated polymer. Fluorinated products available through this approach include a glutathione *S*-transferase inhibitor (**3b**), an antibiotic (**3c**), a protease inhibitor (**3d**), deoxyfluorinating agents (**3e**, **3k**)^36,37^, aryl fluorides that are known building blocks for a range of active pharmaceutical ingredients (**3q–3t**) and a hypervalent iodine-based fluorinating agent used for electrophilic fluorinations (**3ab**)^38^. A series of benzyl bromides, phenacyl bromides and cinnamyl halides could also be used as fluoride acceptors but with only modest yields under the current conditions, while an epoxide was unreactive in the transfer fluorination.

To demonstrate the scalability of the approach, reactions were conducted using PVDF (**1v**) as a fluorine source on a 1-g, 10-g and 50-g scale with little drop-off in the yield of the isolated product of transfer fluorination (Fig. 4b). As part of these experiments, it was found that KO^*t*^Bu could be handled and weighed outside of a glovebox, with reactions conducted under a purge of N_2_. The recovered PVDF following transfer fluorination was characterized by powder X-ray diffraction, solid state NMR spectroscopy, elemental analysis and differential scanning calorimetry. Data are consistent with the formation of sites of C=C unsaturation in the polymer following defluorination. Transfer fluorination using volatile HFC gases was also scaled up using a Vapourtec flow system (Fig. 4c). Optimization of gas pressures, flow rates, reagents concentrations, residence times and reactor coil lengths allowed near complete defluorination of 1,2,2,2-tetrafluoroethane (**1j**) in flow with the formation of 3.3 ± 0.2 equiv. of KF corresponding to 82 ± 5% recovery of the available fluorine content. The KF prepared by this flow method could be used directly as a DMSO solution or dried and washed before use allowing the transfer fluorination of a range electrophiles such as silyl chlorides, sulfonyl chlorides or acyl chlorides in 66–90% yield.

These protocols open up a broad and scalable route for the chemical recycling of the fluorine content of important, often single use, industrially relevant fluorocarbons. In particular, the efficient and scalable method to recycle nearly all the fluorine content of third-generation refrigerants complements methodologies in which these gases are used as direct sources of carbon fragments in the fluoroalkylation of electrophiles^11,28,39,40^. We believe the findings represent an important step in the development of a circular economy for fluorine.

## Methods

### Screening transfer fluorination reactions of volatile gases

A one-pot procedure for the screening of gaseous fluorine donors was performed as follows. A J Young NMR tube was charged with 300 µl of THF, followed by a solution of KO^*t*^Bu in THF (200 µl, 0.126 M, 1.2 equiv.) using a micropipette. The solution in the NMR was frozen using liquid N_2_ and the head space was removed under vacuum, before warming to room temperature. The NMR tube was then charged with the fluorine donor gas to 1 bar. The NMR tube was inverted every 10 min to insure thorough mixing for a total of 2 h. A solution of TsCl and 1,2-difluorobenzene (1,2-DFB) in THF (100 µl, 0.105 M (TsCl), 0.105 M (1,2-DFB), 1 equiv.) was transferred by micropipette into the NMR tube before being sealed and heated at 100 °C for 1 h. A quantitative ^19^F NMR spectrum (D_1_ = 55 s) was measured to determine the yield of TsF produced compared with the internal standard 1,2-DFB.

### Flow reactions

Flow reactions were performed using Vapourtec EasyScholar Integrated Flow Chemistry System. Reagents and solvents were delivered under nitrogen with a 19-gauge stainless steel luer lock needle (1.07-mm outer diameter), then 1.6 mm (1/16’’) outer diameter FEP tubing was used (internal diameter 1 mm). HFC-134a was delivered from a lecture bottle of 150 bar regulated to a range of 1.0–1.2 bar (absolute pressure) through a peristaltic pump to control flow rate, and a T junction was used as a micromixer. DMSO and HFC gas was primed at reaction flow rate for at least 1 reactor coil volume. DMSO was switched to base solution to initiate flow reaction. After discarding 1–4 reactor coil volumes, 4 × 1 ml reaction mixture was collected for steady state yield determination, diluted with deionized water and addition of 0.5–1.5 M NaOTf internal standard for ^19^F quantitative NMR spectroscopy.

### 10-g scale transfer fluorination using PVDF

A 1-l two-neck round-bottomed flask with a condenser was charged with PVDF (10 g, 156 mmol) and THF (450 ml; Merck 2.5 l ≥99.0%, ACS reagent, no previous drying or degassing) under a flush of N_2_. The stirring solution (300 rpm) was cooled in an ice bath before adding KO^*t*^Bu (8.762 g, 78.09 mmol) under a flush of N_2_. The reaction mixture was heated to 100 °C in an oil bath for 24 h before cooling to atmospheric temperature. The TsCl (7.443 g, 39.04 mmol) was added to the reaction under a flush of N_2_ before heating the reaction mixture back to 100 °C for an extra 4 h. The cooled solution was filtered slowly through a bed of activated charcoal (diameter 6 cm, thickness 2 cm), and the solids were rinsed with THF (3 × 20 ml). The volatiles were removed under reduced pressure (5 × 10^−2 ^mbar), without addition of an external heating source to avoid the sublimation of **3a**, to obtain off white crystalline TsF (6.029 g, 34.61 mmol, 89%).

## Online content

Any methods, additional references, Nature Portfolio reporting summaries, source data, extended data, supplementary information, acknowledgements, peer review information; details of author contributions and competing interests; and statements of data and code availability are available at 10.1038/s41557-026-02096-8.

## Supplementary information

Supplementary Information Materials, Methods and Experimental Details; Sections 1–17, including Figs. 1–92 and Tables 1–44.

Supplementary Data 1 Supplementary data file with the Cartesian coordinates (*xyz*) for theoretical calculations.

## Source data

Source Data Fig. 2 Source data for Fig. 2e.

## Data Availability

CCDC 2394846 (**1a**) and 2394847 (**2a**) contain the supplementary crystallographic data for this paper. These data can be obtained free of charge available at The Cambridge Crystallographic Data Centre via www.ccdc.cam.ac.uk/data_request/cif, by emailing data_request@ccdc.cam.ac.uk or by contacting The Cambridge Crystallographic Data Centre, 12 Union Road, Cambridge CB2 1EZ, UK. Materials and methods, synthetic procedures, NMR spectra of all compounds, crystallographic data, computational methods (PDF), and Cartesian coordinates of the DFT-optimized structures (*xyz*) are available in the paper or Supplementary Information. Source data are provided with this paper.
