# Supplementary material for: Chemical recycling of hydrofluorocarbons by transfer fluorination
Source: Nat Chem. 2026 Mar 13;18(5):899–904. doi: 10.1038/s41557-026-02096-8 (PMC13149033; doi:10.1038/s41557-026-02096-8)
Supplement: Supplementary file 1 — Materials, Methods and Experimental Details; Sections 1–17, including Figs. 1–92 and Tables 1–44. [file 41557_2026_2096_MOESM1_ESM.pdf]

nature chemistry

Supplementary information

https://doi.org/10.1038/s41557-026-02096-8

Chemical recycling of hydrofluorocarbons by transfer fluorination

In the format provided by the authors and unedited

Table of Contents

General experimental information: Materials and methods 5

1. Synthesis and characterisation data of fluorine donors **1a** and **1d** 7

2. Reaction Optimisation 9

2.1. Optimisation of Additives 9

2.2. Optimisation involving Fluorinated gases 11

2.3. Optimisation of Solvent 12

2.4. Optimisation of Base 13

2.5. Characterisation of Fluoroalkene Side-product **2a** 14

3. Competition Experiments using KHMDS 15

4. Procedures for the transfer fluorination of tosyl chloride in the fluorine donor substrate scope 16

5. The scope of fluorine donors **1a–1t** 19

5.1. Fluorination using **1a** (Procedure A) 20

5.2. Fluorination using **1b** (Procedure A) 21

5.3. Fluorination using **1c** (Procedure A) 22

5.4. Fluorination using **1d** (Procedure A) 23

5.5. Fluorination using **1e** (Procedure A) 24

5.6. Fluorination using **1f** (Procedure A) 25

5.7. Fluorination using **1g** (Procedure A) 26

5.8. Fluorination using **1h**: HFC-152a (Procedure B) 27

5.9. Fluorination using **1i**: HFC-143a (Procedure B) 28

5.10. Fluorination using **1j**: HFC-134a (Procedure B) 29

5.11. Fluorination using **1l**: HFO-1234yf (Procedure B) 30

5.12. Fluorination using **1m**: Sevoflurane (Procedure A) 31

5.13. Fluorination using **1n**: Isoflurane (Procedure A) 32

5.14. Fluorination using **1o**: Enflurane (Procedure A) 33

5.15. Fluorination using **1p**: Hexafluoroisopropyl methyl ether (Procedure A) 34

5.16. Fluorination using **1q**: Bis(2,2,2-trifluoroethyl) ether (Procedure A) 35

5.17. Fluorination using **1r**: TFTFE (Procedure A) 36

5.18. Fluorination using **1s**: Volatile PFAS (Procedure A) 37

5.19. Fluorination using **1t**: Volatile PFAS (Procedure A) 38

5.20 Fluorination using **1a** (Procedure C) 39

5.21 Fluorination using **1b** (Procedure C) 40

5.22 Fluorination using **1c** (Procedure C) 41
5.23 Fluorination using **1d** (Procedure C) 42
5.24 Fluorination using **1e** (Procedure C) 43
5.25 Fluorination using **1g** (Procedure C) 44
5.26 Fluorination using **1h**: HFC-152a (Procedure D) 45
5.27 Fluorination using **1i**: HFC-143a (Procedure D) 46
5.28. Fluorination using **1j**: HFC-134a (Procedure D) 47
5.29 Fluorination using **1k**: HFC-125 (Procedure D) 48
5.30 Fluorination using **1l**: HFO-1234yf (Procedure D) 49
5.31 Fluorination using **1m**: Sevoflurane (Procedure C) 50
5.32 Fluorination using **1n**: Isoflurane (Procedure C) 51
5.33 Fluorination using **1o**: Enflurane (Procedure C) 52
5.354 Fluorination using **1p**: Hexafluoroisopropyl methyl ether (Procedure C) 53
5.35 Fluorination using **1q**: Bis(2,2,2-trifluoroethyl) ether (Procedure C) 54
5.36 Fluorination using **1r**: TFTFE (Procedure C) 55
5.37 Fluorination using **1s**: Volatile PFAS (Procedure C) 56
5.38 Fluorination using **1t**: Volatile PFAS (Procedure C) 57
5.39 Donor limitation of the transfer fluorination protocol 58
6. KF Quantification 59
7. Characterisation of the heterogenous fluorinating species 62
7.1. Photograph of NMR scale reaction 62
7.2. Isolation of KF 63
7.3. Identification of KF species *via* X-ray powder diffraction 64
7.4. Characterisation of KF species *via* field emission scanning electron microscopy 67
7.5. Characterisation of KF species *via* MAS Solid State NMR spectroscopy 71
8. Total Defluorination of HFC-134a and Near Total Defluorination of HFC-125: 73
8.1 Equivalent Screening for KO^{t}Bu in THF for Defluorination of HFC-134a 73
8.2. Solvent Screening for Defluorination of HFC-134a: 74
8.3. Base Screening in DMSO for Defluorination of HFC-134a: 75
8.4. Temperature and Time Study for 4 Equivalent of KO*t*Bu in DMSO for Defluorination of HFC-134a: 76
8.5. Equivalent Screening for KO^{t}Bu in DMSO for Defluorination of HFC-134a: 77
8.6. Heating Time Study for 6 Equivalent of KO*t*Bu in DMSO for Defluorination of HFC-134a: 78
8.7. Heating Time Study and Effect of Shaking for 5 Equivalent of KO*t*Bu in DMSO for Defluorination of HFC-134a: 79
8.8. 1 Bar (Absolute) Pressure Study of HFC-134a with 6 Equivalent of KOtBu in DMSO: 80

8.9. Example Quantitative ^{19}F NMR Spectra for Defluorination Fluoride Yield: 81
8.10. Temperature and Time Study of 7 Equivalent KOtBu in DMSO for Defluorination of HFC-125: 82
9. Mechanism of Total Defluorination of HFC-134a and Final Product: 83
10. Transfer fluorination using PFOA (**1u**) 87
11. Synthesis of fluorochemicals by transfer fluorination 93
11.1. S–F bond formation: Synthesis and characterisation of sulfonyl fluorides 94
11.2. sp^{2}C–F bond formation: Synthesis and characterisation of acyl fluorides 102
11.3. sp^{3}C–F and sp^{2}C–F bond formation: Synthesis and characterisation of alkyl and aryl fluorides 110
11.4. Si–F bond formation: Synthesis and characterisation of products 115
11.5. P–F bond formation: Synthesis and characterisation of products 121
11.6. I–F bond formation: Synthesis and characterisation of products 124
11.7. Less successful fluorination acceptors 125
12. Fluorination using **1v**: Polyvinylidene fluoride 126
12.1. Fluorine transfer procedure of **1v** using KHMDS 126
12.2 Fluorine transfer procedure of **1v** using KO^{t}Bu 128
12.3 Characterisation of post reaction precipitate for **1v** 131
12.3.1 FTIR 131
12.3.2 CHN Elemental Analysis 132
12.3.3 Differential scanning calorimetry (DSC) 133
12.3.4 Solid-State NMR 136
12.2.5 Powder X-Ray Diffraction (XRD) 138
13. Total Defluorination of HFC-134a Scale-up in Flow: 139
13.1. General Flow Chemistry Procedure: 139
13.2. Flow Chemistry Stoichiometry Calculations and Assumptions: 139
13.3. Flow Chemistry Setup Overview: 140
13.4. Temperature and Residence Time Study for Total Defluorination of HFC-134a: 140
13.5. Direct *in situ* Transfer-fluorination: 142
13.6. Isolation of KF from Flow: 145
13.7. Transfer-fluorination with Isolated KF from Flow: 148
14. NMR spectra 150
14.1. NMR spectra of species **1a**, **2a** and **1d** 150
14.2. NMR spectra of fluorinated products 155
15. Single-crystal X-ray crystallography 198
15.1. X-Ray data 198

15.2. Refinement details 199
15.3. Crystal structures 199
16. Computational chemistry – Calculations 200
17. References 341

General experimental information: Materials and methods

General: Unless stated otherwise, reactions were carried out using standard Schlenk-line and glovebox (MBraun Labmaster, operating at 20 °C and <0.1 ppm H_2O and <0.1 ppm O_2) techniques under an inert atmosphere of nitrogen. Reactions were heated using silicon oil baths, except when reactions were carried out in round bottom flasks, where aluminium heating blocks were used. Glassware was dried overnight at 120 °C prior to usage. Pre-coated TLC sheets from Merck (silica gel 60 with fluorescent indicator UV_{254}) were used for thin layer chromatography, and glass-plate based 20 cm x 20 cm TLC sheets from Merck Silica gel 60 (silica gel 60 with fluorescent indicator UV_{254}) were used for preparative thin layer chromatography. Silica gel (technical grade, pore size 60 Å, 230-400 mesh particle size, 40-63 µm particle size) was used for liquid chromatography.

Analysis: NMR spectroscopy was conducted using BRUKER 400 MHz instruments at 25 °C. Chemical shifts (δ) are reported in ppm relative to the residual proton or carbon chemical shift of the deuterated solvent used (^{1}H: 7.26 for $CDCl_3$ and 7.16 for C_6D_6; ^{13}C(^{1}H): 77.16 for $CDCl_3$, 128.06 for C_6D_6 and 1.32 for CD_3CN). ^{19}F NMR, ^{29}Si NMR and ^{31}P NMR spectra were referenced relative to external standards ($CFCl_3$ for ^{19}F; 1% Me_4Si in $CDCl_3$ for ^{29}Si; H_3PO_4 for ^{31}P). Quantitative ^{19}F NMR with a delay of 55 s was used for the determination of *in situ* yields, and the peak intensities were derived against the internal standard signal of *ortho*-difluorobenzene (δ = -139.8 ppm). NMR data was processed using TopSpin 3.6.5 software and MestReNova software package. Multiplicities in NMR are reported as s (singlet), d (doublet), t (triplet), q (quartet), m (multiplet), *pseudo*-t (*pseudo*-triplet), dd (doublet of doublets), dm (doublet of multiplets), quintd (quintet of doublets) and ddd (doublet of doublets of doublets). Accurate mass spectrometry measurements were acquired using a Thermo Fisher Q-Exactive Orbitrap mass spectrometer with atmospheric pressure chemical ionization (APCI) as ionisation technique (capillary temperature at 320 °C and discharge voltage of 4.0 kV). AT-IR spectra were recorded on an Agilent Technologies Cary 630 FTIR spectrometer. X-ray powder diffraction (PXRD) data was collected at room temperature using a Bruker D2 Phaser X-ray diffractometer. Field emission scanning electron microscopy (FE-SEM) was performed using a SIGMA 300 Zeiss Gemini instrument. Samples were coated with chromium of 15 nm depth prior to data collection. Melting points were determined using a Stuart Melting Point Apparatus SMP10. MAS-SS-NMR measurements were carried out by Dr. Nasima Kanwal at Queen Mary University London on a Bruker 400 MHz spectrometer.

Solvents: Tetrahydrofuran (THF), diethyl ether (Et_2O) and toluene were dried over activated alumina from a SPS (solvent purification system) and stored over 3 Å molecular sieves inside a glovebox before usage. Triglyme was distilled from CaH_2 under reduced pressure and stored over 3 Å molecular sieves inside a glovebox before usage. Benzene-d_6 (C_6D_6), *ortho*-difluorobenzene (*o*-DFB) and dimethyl sulfoxide (DMSO) were degassed via freeze-pump-thaw technique as well as stored over 3 Å molecular sieves inside a glovebox before usage. All other solvents utilised in workups or chromatography were used as received wet solvents. Anhydrous DMSO (Sigma-Aldrich, 2.5L) was dried over 25% v/v% of activated 3 Å molecular sieves three times each for 4 – 7 days, before degassing three times via freeze-pump-thaw technique and stored over activated sieves in glovebox. Karl_Fischer titration moisture level was less than 10 ppm.

Reagents: All commercially available reagents were purchased from commercial suppliers (Fluorochem (vast majority), VWR International, Fisher Scientific, Merck Life Science UK, Tokyo Chemical Industry UK, and Apollo Scientific), and used without further purification – unless stated otherwise. Commercial anhydrous potassium fluoride was purchased from Fluorochem and stored in a dinitrogen filled glovebox as received. Potassium fluoride from ACROS Organics was stored on a laboratory shelf under atmospheric conditions.

Commercially available fluorine donors: The fluorine donors ((2,2,2-trifluoroethyl)-benzene (**1e**); (3,3,3-trifluoropropyl)-benzene (**1f**); 3,3,3-trifluoropropanenitrile (**1g**)), the anaesthetics (sevoflurane (**1m**); isoflurane (**1n**); enflurane (**1o**)), the electrolyte additives/co-solvents (hexafluoroisopropyl methyl ether (**1p**); bis(2,2,2-trifluoroethyl) ether (**1q**); 1,1,2,2-tetrafluoroethyl 2,2,2-trifluoroethyl ether (**1r**)) and the polyfluoroalkyl substances (1H,2H-octafluorocyclopentane (**1s**); 1H-perfluoropentane (**1t**); perfluorooctanoic acid (**1u**)) as well as poly(vinylidene difluoride)(**1v**) were purchased from Fluorochem or Apollo Scientific and stored over 3 Å molecular sieves in a glovebox where appropriate. Fluorinated gases (1,1-difluoroethane (**1h**); 1,1,1-trifluoroethane (**1i**); 1,1,1,2-tetrafluoroethane (**1j**); 1,1,1,2,2-pentafluoroethane (**1k**); 2,3,3,3-tetrafluoropropene (**1l**)) were acquired from Apollo Scientific or CK Gas Products and used without further purification.

Reagents (synthesised): The fluorine donors 1,1,1-trifluoro-2,2-diphenyl-ethane (**1b**) and 1,1,1-trifluoro-2,2-(bismesityl)-ethane (**1c**) were synthesised according to literature.[1] For the synthesis of the fluorine acceptor 2-pyridinesulfonyl chloride, the corresponding literature procedure was followed.[2]

1. Synthesis and characterisation data of fluorine donors **1a** and **1d**

2,2-bis-(2,4,6-Trifluorophenyl)-1,1,1-trifluoroethane (1a)

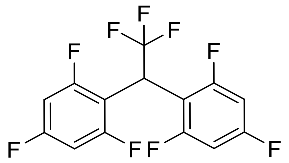

Using a modification of the protocol reported by Prakash *et al.*,[1] commercially available 1,3,5-trifluorobenzene (5.20 mL; 50.30 mmol) and trifluoroacetaldehyde ethyl hemiacetal of 90% purity (1.03 mL, 9.45 mmol) were added to a round bottom flask containing a stirrer bar. While stirring at 20 °C, triflic acid (22.50 mL, 170.00 mmol) was added slowly *via* dropping funnel over a range of 13 minutes, causing a colour change from colourless to pink. The dropping funnel was replaced after completed addition with a condenser, and the reaction temperature was then increased to 70 °C. The reaction mixture was stirred at this temperature for 23 h. The reaction mixture was then allowed to cool down to room temperature over a period of 30 minutes and poured over 200 g ice in a glass beaker. Any remaining liquid in reaction flask was rinsed out with few drops of water and transferred to ice- mixture as well. After the addition of a larger stirrer bar, the resulting cold mixture was slowly neutralised with small portions of $NaHCO_3$ (in total 24 g) while stirring, followed by extraction with 4x125 mL CH_2Cl_2. The organic fractions were combined, dried over Na_2SO_4 and concentrated *en vacuo* (55 °C and <50 mbar) for several hours, providing a white solid of pure product **1a** in 99% yield (3.22 g, 9.37 mmol). Material suitable for single-crystal X-ray diffraction (sc-XRD) was produced by slow diffusion of MeOH into a benzene solution of the product.

^{1}H NMR (400 MHz, $CDCl_3$, 298 K): δ = 6.75–6.66 (*m*, 4H, Ar*H*), 5.35 (q, $^3J_{HF}$ = 10.0 Hz, 1H, $(Ar)_2C\mathit{H}CF_3$).

^{13}C[^{1}H] NMR (101 MHz, $CDCl_3$, 298 K): δ = 163.0 (dt, $^1J_{CF}$ = 251.7 Hz, $^3J_{CF}$ = 15.7 Hz, *p*-$_{Ar}$*C*F), 161.5 (dm, $^1J_{CF}$ = 252.6 Hz, *o*-$_{Ar}$*C*F), 124.7 (q, $^1J_{CF}$ = 280.0 Hz, *C*F_3), 106.8–106.2 (m, C_{Ar}), 101.0 (dd, $^2J_{CF}$ = 31.2 Hz, $^2J_{CF}$ = 26.0 Hz, C_{Ar}H), 35.9 (q, $^2J_{CF}$ = 34.5 Hz, *C*HCF_3).

^{19}F NMR (377 MHz, $CDCl_3$, 298 K): δ = -66.4 (quintd, $^5J_{FF}$ = 10.9 Hz, $^3J_{FH}$ = 10.0 Hz, $C\mathit{F}_3$, 3F), -106.2–(-106.4) (m, Ar*F*, 2F), -107.6–(-107.8) (m, Ar*F*, 4F).

HRMS (APCI): Expected for $[C_{14}H_5F_9]^+$: 344.0242; found: 344.0243.
Melting point (from diethyl ether): 95–96 °C.

2,2-bis-(2,4,6-Trimethoxyphenyl)-1,1,1-trifluoroethane (1d)

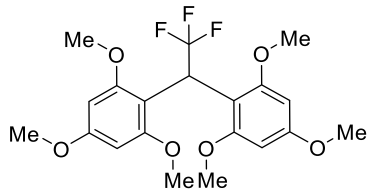

Using a modification of the protocol reported by Prakash *et al.*,[1] commercially available 1,3,5-trimethoxybenzene (5.06 g; 30.09 mmol) and trifluoroacetaldehyde ethyl hemiacetal of 90% purity (0.57 mL, 5.40 mmol) were combined in a round bottom flask containing a stirrer bar. The heterogenous mixture was stirred and cooled down to 0 °C, followed by the slow addition of triflic acid (9.00 mL, 102.00 mmol) *via* dropping funnel over a range of 12 minutes at 0 °C. The reaction mixture was stirred at 0 °C for further 3 hours after which it was poured into a glass beaker containing a larger stirrer bar and 90 g ice, while carefully rinsing out any remaining liquid in reaction flask with few drops of water into the cold mixture as well. The resulting cold mixture was slowly neutralised with small portions of $NaHCO_3$ (in total 10 g) while stirring, followed by extraction with 4x50 mL CH_2Cl_2. The organic fractions were combined, dried over $MgSO_4$ and concentrated *en vacuo* at 45 °C and p>50 mbar. The crude product was then further purified *via* liquid chromatography on silica gel with CH_2Cl_2/*n*-hexane gradients, providing a white solid of pure product **1d** in 93% yield (2.08 g, 5.00 mmol).

^{1}H NMR (400 MHz, $CDCl_3$, 298 K): δ = 6.10 (s, 4H, Ar*H*), 5.65 (q, $^3J_{HF}$ = 11.7 Hz, 1H, C*H*CF_3), 3.78 (s, 6H, *p*-OC*H*$_3$), 3.73 (s, 12H, *o*-OC*H*$_3$).

^{13}C[^{1}H] NMR (101 MHz, $CDCl_3$, 298 K): δ = 160.2 (s, C_{Ar}), 159.9 (s, C_{Ar}), 127.3 (q, $^1J_{CF}$ = 278.5 Hz, *C*F_3),

107.0 (s, C_{Ar}), 91.4 (s, C_{Ar}H), 56.2 (s, *o*-O*C*H_3), 55.3 (s, *p*-O*C*H_3), 36.6 (q, $^2J_{CF}$ = 32.1 Hz, *C*HCF_3).

^{19}F NMR (377 MHz, $CDCl_3$, 298 K): δ = -64.3 (d, $^3J_{HF}$ = 11.7 Hz, 3F, C*F*$_3$).

Spectroscopic data in agreement with reported data.[3]

2. Reaction Optimisation

2.1. Optimisation of Additives

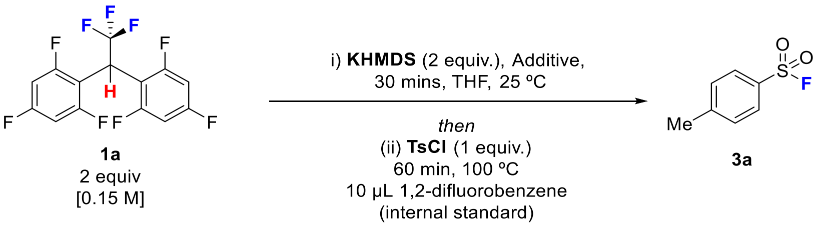

Supplementary Scheme 1: Investigating the role of additives on the outcome of transfer fluorination.

Transfer fluorination of TsCl using 1a: In a dinitrogen containing glovebox, of **1a** (0.12 mmol, 38 mg) and the additive (see Supplementary Table 1 for composition and amounts) were dissolved in 0.4 mL THF and transferred into a J. Youngs NMR tube. To this reaction mixture, a solution of KHMDS (0.12 mmol) in 0.2 mL THF was added at room temperature. The reaction mixture instantly became heterogeneous and with solid being deposited on the wall of the J Young NMR tube. At longer reaction times, a dark precipitate was also observed. The tube was sealed and inverted every 10 minutes to maintain proper mixing. After 30 minutes, approximately TsCl (0.06 mmol, 11.4 mg) in 0.2 mL THF was added followed by the addition of 10 µL *o*-DFB as internal standard. The NMR tube was then sealed, removed from the glovebox and transferred into a 100 °C hot oil bath. The NMR tube was inverted every 15 minutes to maintain a proper mixing of the reactants. After a reaction time of 60 minutes, the tube was removed from the oil bath and allowed to cool to room temperature, after which the yield of TsF was determined *via* quantitative ^{19}F NMR spectroscopy.

Control reaction using commercial KF: In a dinitrogen containing glovebox, KF (0.12 mmol, 7 mg; dry KF from Fluorochem or KF stored at ambient atmosphere from Acros Organics) was suspended in 0.6 mL THF. The suspension was treated with approximately 0.06 mmol TsCl (11.4 mg) in 0.2 mL THF, followed by the addition of 10 µL *o*-DFB as internal standard. The NMR tube was then sealed, removed from the glovebox and transferred into a 100 °C hot oil bath. The NMR tube was inverted every 15 minutes to maintain a proper mixing of the reactants. After a reaction time of 60 minutes, the tube was removed from the oil bath and allowed to cool to room temperature, after which the yield of TsF was determined *via* quantitative ^{19}F NMR spectroscopy (see Supplementary Table 1).

Supplementary Table 1: Investigation of the influence of additives and nature of KF on transfer fluorination.

Entry	F- donor (D)	Additive (A)	Ratio D:A	Ratio donor: acceptor	Fluorination time [h]	Yield R–F [%]
**1**	**1:1 KHMDS:1a**	**18-crown-6**	**1:1**	**2:1**	**1**	**90**
**2**	**1:1 KHMDS:1a**	**18-crown-6**	**10:1**	**2:1**	**1**	**81**
**3**	1:1 KHMDS:**1a**	2.2.2-cryptand	1:1	2:1	1	70
**4**	**1:1 KHMDS:1a**	**None**	**None**	**2:1**	**1**	**90**
**5**	Commercial anhydrous KF (stored in glovebox)	None	None	2:1	1	5
**6**	Commercial anhydrous KF (stored under air)	None	None	2:1	1	11
**7**	**Isolated KF**	**None**	**None**	**2:1**	**1**	**75**
**8**	1.2:1 KHMDS:*o*-DFB (no **1a**)	None	None	-	1	0

2.2. Optimisation involving Fluorinated gases

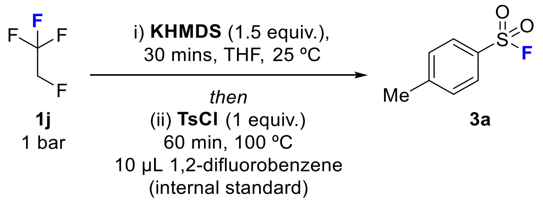

Supplementary Scheme 2: Investigation of the influence of concentration on the fluorination outcome.

In a dinitrogen containing glovebox, KHMDS (0.04–0.32 mmol, 1.52 equiv.) was dissolved in 0.6 mL THF and transferred into a J. Youngs NMR tube. The tube was removed from the glovebox and degassed via freeze-pump-thaw technique twice before the fluorinated gas (1 bar) was added (Supplementary Scheme 2). The reaction mixture instantly became heterogeneous and with solid being deposited on the wall of the J Young NMR tube. At longer reaction times, a dark precipitate was also observed. The tube was sealed and inverted every 10 minutes to maintain proper mixing. After 30 minutes, the tube was transferred back into the glovebox. The tube was opened, and tosyl chloride (0.03–0.22 mmol; 1 equiv.; limiting reagent) in 0.2 mL THF was added, followed by the addition of 10 µL *o*-DFB as internal standard (Supplementary Scheme 2). The NMR tube was then sealed, removed from the glovebox and transferred into a 100 °C hot oil bath. The NMR tube was inverted every 15 minutes to maintain a proper mixing. After a reaction time of 60 minutes, the tube was removed from the oil bath and allowed to cool to room temperature, after which the yield of tosyl fluoride (TsF; **3a**) was determined *via* quantitative ^{19}F NMR spectroscopy. Supplementary Table 2 displays relevant parameters and outcomes.

Supplementary Table 2: KHMDS concentration optimisation for reactions containing gas.

Entry	KHMDS [mmol]	HFC-134a [mL]	HFC-134a [mmol]	Mol ratio KHMDS:gas	THF volume [mL]	Yield TsF [%]
1	0.040	1.9	0.1	2.5	0.8	77
2	0.082	1.9	0.1	1.2	0.8	78
3	0.167	1.9	0.1	0.6	0.8	90
4	0.323	1.9	0.1	0.3	0.8	52

2.3. Optimisation of Solvent

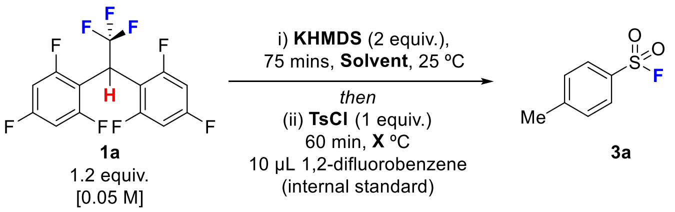

Supplementary Scheme 3: Solvent influence on the transfer fluorination of TsCl with **1a**.

In a dinitrogen containing glovebox, **1a** (0.055 mmol, 18.9 mg) was dissolved in 0.4 mL solvent (Supplementary Table 3) and transferred into a J Youngs NMR tube. To this mixture, a solution of KHMDS (0.090 mmol, 18.0 mg) in 0.5 mL solvent was added at room temperature. The reaction mixture instantly became heterogeneous and with solid being deposited on the wall of the J Young NMR tube. At longer reaction times, a dark precipitate was also observed. The tube was sealed and inverted every 15 minutes to maintain proper mixing. After an activation period of 75 minutes, TsCl (0.045 mmol, 8.5 mg) in 0.2 mL solvent was added, followed by the addition of 10 µL *o*-DFB as internal standard. The NMR tube was then sealed, removed from the glovebox and transferred into an oil bath of corresponding temperature. The NMR tube was inverted every 15 minutes to maintain a proper mixing of the reactants. After a reaction time of 60 minutes, the tube was removed from the oil bath and allowed to cool to room temperature, after which the yield of TsF was determined *via* quantitative ^{19}F NMR spectroscopy (Supplementary Table 3).

Supplementary Table 3: Investigation of solvent influence on transfer fluorination. *#KOtBu used as base in place of KHMDS.*

**Solvent**	**Boiling point of solvent [°C]**	**Fluorination temperature [°C]**	**Yield 3a [%]**
THF	**66**	**100**	**90**
C_6D_6	**79**	**100**	**80**
**Toluene**	111	100	54
**Hexane**	69	100	39
**Et_2O**	35	60	69
**Triglyme**	216	100	67
**DMF**	153	100	69
**NMP**	202	100	0
**NMP**	202	100	16#
**MeCN**	82	100	66
**DMSO**	189	100	0
**DMSO**	189	100	0#

2.4. Optimisation of Base

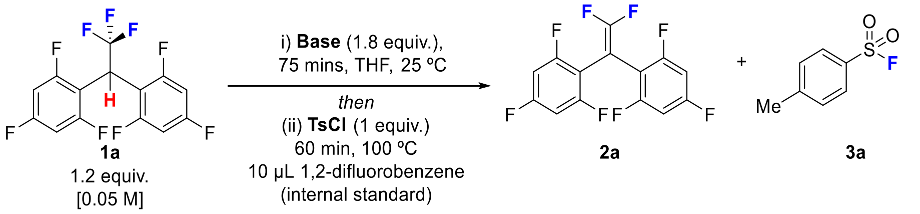

Supplementary Scheme 4: The effect of the base on transfer fluorination of TsCl using **1a**.

In a dinitrogen containing glovebox, **1a** (0.055 mmol, 18.9 mg) was dissolved in 0.4 mL THF and transferred into a J. Youngs NMR tube. To this mixture, a solution or suspension of base (0.083 mmol, see Supplementary Table 4) in 0.5 mL THF was added at room temperature. The tube was sealed and inverted every 10 minutes to maintain proper mixing, in some cases a precipitate was observed. After 75 minutes, 10 µL *o*-DFB was added as internal standard and the consumption of **1a** was determined *via* quantitative ^{19}F NMR spectroscopy. After which point the NMR tube was returned to the glovebox and TsCl (0.045 mmol, 8.6 mg) in 0.3 mL THF was added. The NMR tube was then sealed, removed from the glovebox and transferred into a 100 °C hot oil bath. The NMR tube was inverted every 15 minutes to maintain a proper mixing of the reactants. After a reaction time of 60 minutes, the tube was removed from the oil bath and allowed to cool to room temperature, after which the yield of TsF was determined *via* quantitative ^{19}F NMR spectroscopy (see Supplementary Table 4).

Supplementary Table 4: Influence of the base on the transfer fluorination.

Base	1a consumption [%]	Yield 2a [%]	Yield 3a [%]
**LiHMDS**$^{\#\$}$	>95%	>95%	0
**NaHMDS**	91	45	5
KHMDS	**>95%**	**44**	**90**
CsHMDS	**>95%**	**91**	**82**
**Mg(HMDS)$_2$**	3	0	0
**Sr(HMDS)$_2$**	67	51	0
**Zn(HMDS)$_2$**	0	0	0
**KH**	0	0	0
KCH_2Ph	**73**	**49**	**>95%**
KO*t*Bu	**92**	**43**	**71**
**KOH**	1	1	0
**K_2CO_3**	0	0	0
**KCp**	11	6	0
**NaCp**	15	15	0
**NaO*t*Am**	95	72	2
**NaOMe**	3	3	0
**Ca(HMDS)$_2$**	70	56	9

$^{\#}$Only 1.1 eq. of LiHMDS used. $^{\$}$12-crown-6 ether as additive did also not facilitate fluorination.

2.5. Characterisation of Fluoroalkene Side-product **2a**

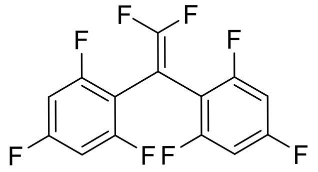

2,2-Bis-(2,4,6-trifluorophenyl)-1,1-difluoroethene

In a nitrogen filled glovebox, **1a** (34 mg; 0.10 mmol) was dissolved in 0.3 mL THF in a vial. Separately, LiHMDS (20 mg; 0.11 mmol) was dissolved in 0.3 mL THF and added dropwise to the donor-solution. The reaction mixture was transferred into a J.Young NMR tube and ^{19}F *in situ* NMR showed quantitative conversion to **2a** after 1 h reaction time. The NMR tube was then connected to a Schlenk line and the solvent removed under high vacuum. **2a** could be isolated as a colourless solid ($R_f \approx$ 0.88; 3:1 petroleum ether:diethyl ether).

^{1}H NMR (400 MHz, $CDCl_3$, 298 K): δ = 6.70 (*pseudo*-t, $^3J_{HF}$ = 8.0 Hz, $^3J_{HF}$ = 7.9 Hz, 4H, Ar*H*).

^{13}C{^{1}H} NMR (101 MHz, $CDCl_3$, 298 K): δ = 163.1 (dt, $^1J_{CF}$ = 251.4 Hz, $^3J_{CF}$ = 15.2 Hz, *p*-$_{Ar}$*C*F), 161.1 (dm, $^1J_{CF}$ = 253.2 Hz, *o*-$_{Ar}$*C*F), 154.2 (t, $^1J_{CF}$ = 296.1 Hz, C=*C*F_2), 106.0 (C_{Ar}), 100.7 (*pseudo*-t, $^2J_{CF}$ = 27.0 Hz, $^2J_{CF}$ = 26.6 Hz, C_{Ar}H), 72.8 (*C*=CF_2, confirmed *via* ^{19}F-^{13}C-HMBC).

^{19}F NMR (377 MHz, $CDCl_3$, 298 K): δ = -79.6 (s, C=CF_2, 2F), -106.3–(-106.5) (m, *p*-Ar*F*, 2F), -108.0–(-108.1) (m, *o*-Ar*F*, 4F).

APCI-HRMS: Expected for $[C_{14}H_4F_8]^+$: 324.0180; found: 324.0175.

Melting point (from CH_2Cl_2): 138 °C.

3. Competition Experiments using KHMDS

General Procedure of Competition Study:

In separate 1 mL vials, the two different fluorine donors (**1b** and **1c** or **1c** and **1d**) were weighed out (0.12-0.16 mmol, 5 equiv.) and transferred into a J Young NMR tube with a total of 0.6 mL THF (3 X 0.2 mL). A capillary insert, with a 1:1 mixture of C_6D_6:1,2-difluorobenzene, was inserted into the NMR tube before sealing and a T_0 ^{19}F NMR spectrum (D_1 = 55 s) was measured. KHMDS (0.025-0.033 mmol, 1 equiv.) was weighed out and added as a solid to the reaction solution in the NMR tube before sealing and inverting the NMR tube multiple time to insure thorough mixing. Another ^{19}F NMR spectrum (D_1 = 55 s) was measured to determine the consumption ratio of the fluorine donors.

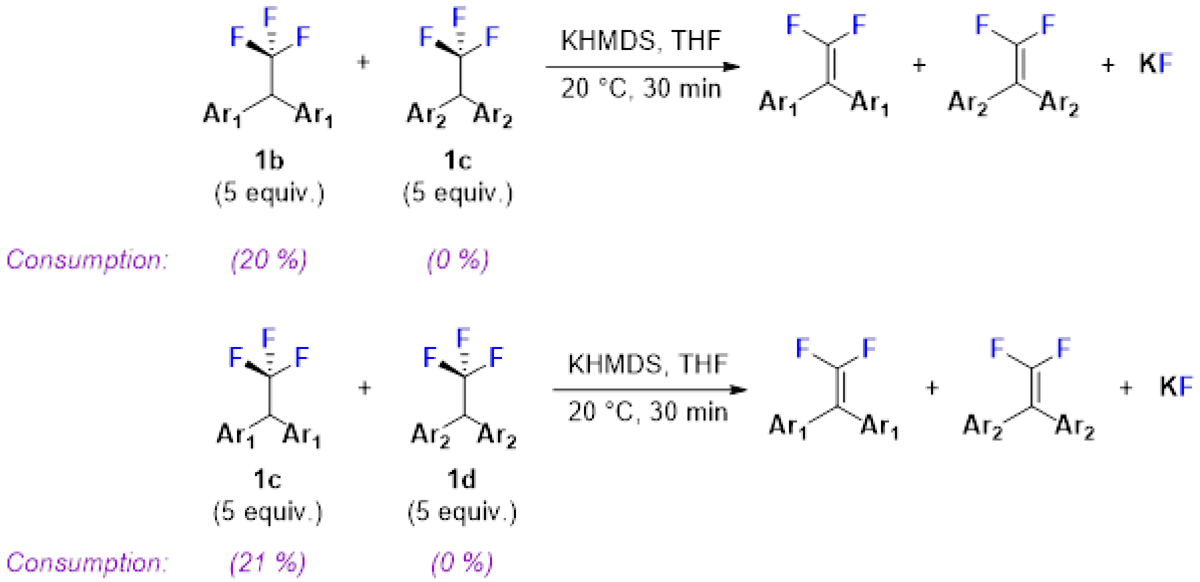

Supplementary Scheme 5: Competition reactions between **1b** and **1c** (top) or **1c** and **1d** (bottom).

4. Procedures for the transfer fluorination of tosyl chloride in the fluorine donor substrate scope

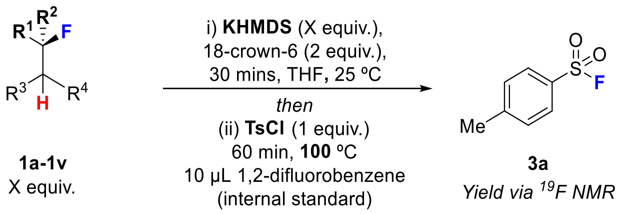

Supplementary Scheme 6: Procedure for the donor screening using KHMDS (Procedure A and B).

General Procedure A: Solid/Liquid Fluorine Donors Substrate Scope using KHMDS

A one-pot procedure for the screening of liquid and solid fluorine donors was performed as follows (Supplementary Scheme 6). In a dinitrogen containing glovebox, the fluorine donor (0.11–0.13 mmol) and 18-crown-6 (0.12 mmol, 31.7 mg) were dissolved in 0.4 mL THF and transferred into a J. Youngs NMR tube. To this reaction mixture, a solution of KHMDS (0.11–0.13 mmol, 21.9–25.9 mg) in 0.2 mL THF was added at room temperature and the dehydrofluorination reaction was carried out within 20–100 °C (depending on donor) for 30 minutes. The reaction mixture instantly became heterogeneous and with solid being deposited on the wall of the J Young NMR tube. At longer reaction times, a dark precipitate was also observed. The NMR tube was sealed and inverted every 10 minutes to maintain proper mixing. After 30 minutes, tosyl chloride (TsCl, 0.039–0.084 mmol, 7.4–16.0 mg) in 0.2 mL THF was added to the J Young tube, followed by the addition of 10 µL *o*-DFB as internal standard. The NMR tube was then sealed, removed from the glovebox and transferred into a 100 °C hot oil bath. The NMR tube was inverted every 15 minutes to maintain a proper mixing of the reactants. After a reaction time of 60 minutes, the tube was removed from the oil bath and allowed to cool to room temperature, after which the yield of tosyl fluoride (TsF; **3a**) was determined *via* quantitative ^{19}F NMR spectroscopy (D_1 = 55 s).

General Procedure B: Gas Fluorine Donors Substrate Scope using KHMDS

A one-pot procedure for the screening of gaseous fluorine donors was performed as follows (Supplementary Scheme 6). In a dinitrogen containing glovebox, 18-crown-6 (0.12 mmol, 31.7 mg) and KHMDS (0.12 mmol, 23.9 mg) were dissolved in 0.6 mL THF and transferred into a J. Youngs NMR tube. The tube was removed from the glovebox and degassed via freeze-pump-thaw technique twice before the fluorinated gas (1.0–1.4 bar) was added. The reaction mixture instantly became heterogeneous and with solid being deposited on the wall of the J Young NMR tube. At longer reaction times, a dark precipitate was also observed. The tube was sealed and inverted every 10 minutes to maintain proper mixing. After 30 minutes, the tube was transferred inside a glovebox. Inside the glovebox, the tube was opened, and tosyl chloride (TsCl, 0.044–0.082 mmol, 8.4–15.6 mg) in 0.2 mL THF was added within a minute, followed by the addition of 10 μL *o*-DFB as internal standard. The NMR tube was then sealed, removed from the glovebox and transferred into a 100 °C hot oil bath. The NMR tube was inverted every 15 minutes to maintain a proper mixing of the reactants. After a reaction time of 60 minutes, the tube was removed from the oil bath and allowed to cool to room temperature, after which the yield of tosyl fluoride (TsF; **3a**) was determined *via* quantitative ^{19}F NMR spectroscopy (D_1 = 55 s).

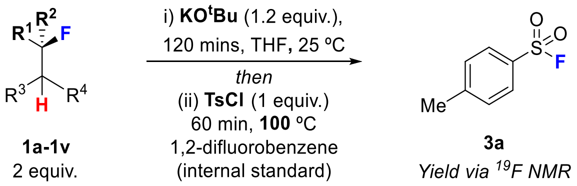

Supplementary Scheme 7: Procedure for the donor screening using KO^{t}Bu (Procedure C and D).

General Procedure C: Solid/Liquid Fluorine Donors Substrate Scope using KO^{t}Bu

A one-pot procedure for the screening of liquid and solid fluorine donors was performed as follows (Supplementary Scheme 7). In a 1 mL vial, the fluorine donor (0.04 mmol, 2 equiv.) was weighed out and transferred to a J Young NMR tube using THF (3 X 100 μL). A solution of KO^{t}Bu in THF (200 μL, 0.126 M, 1.2 equiv.) was transferred by micropipette into the NMR tube before sealing and inverting. The NMR tube was inverted every 10 min to insure thorough mixing for a total of 2 h. A solution of TsCl and 1,2-DFB in THF (100 μL, 0.105 M [TsCl], 0.105 M [1,2-DFB], 1 equiv.) was transferred by micropipette into the NMR tube before being sealed and heated at 100 °C for 1 h. A ^{19}F NMR spectrum (D_1 = 55 s) was measured to determine the yield of TsF produced compared to the internal standard 1,2-DFB.

General Procedure D: Gas Fluorine Donors Substrate Scope using KO^tBu

A one-pot procedure for the screening of gaseous fluorine donors was performed as follows (Supplementary Scheme 7). A J Young NMR tube was charged with 300 µL of THF, followed by a solution of KO^tBu in THF (200 µL, 0.126 M, 1.2 equiv.) using a micropipette. The solution in the NMR was frozen using liquid N_2 and the head space was removed under vacuum, before warming to room temperature. The NMR tube was then charged with the fluorine donor gas to 1 bar. The NMR tube was inverted every 10 min to insure thorough mixing for a total of 2 h. A solution of TsCl and 1,2-DFB in THF (100 µL, 0.105 M [TsCl], 0.105 M [1,2-DFB], 1 equiv.) was transferred by micropipette into the NMR tube before being sealed and heated at 100 °C for 1 h. A ^{19}F NMR spectrum (D_1 = 55 s) was measured to determine the yield of TsF produced compared to the internal standard 1,2-DFB.

5. The scope of fluorine donors **1a–1t**

Safety statement on tetrafluoroethene (TFE):

Tetrafluoroethylene (TFE) as a potential defluorination intermediate is a highly flammable, odorless gas that forms explosive air mixtures (~5–75 vol%) and can undergo rapid exothermic polymerization, leading to pressure buildup and explosion. Thermal decomposition or combustion produces highly toxic gases (COF_2, HF, PFIB). TFE is an asphyxiant, and repeated high-dose exposure can cause kidney toxicity and carcinogenicity. It must be handled with controlled gas release, strict exclusion of ignition sources, and adequate ventilation. Structurally related fluorinated olefin analogues may exhibit similar toxicity and hazard profiles and should be handled with comparable precautions.

The following fluorine donors were used. Yields and ^{19}F NMR spectra are given below.

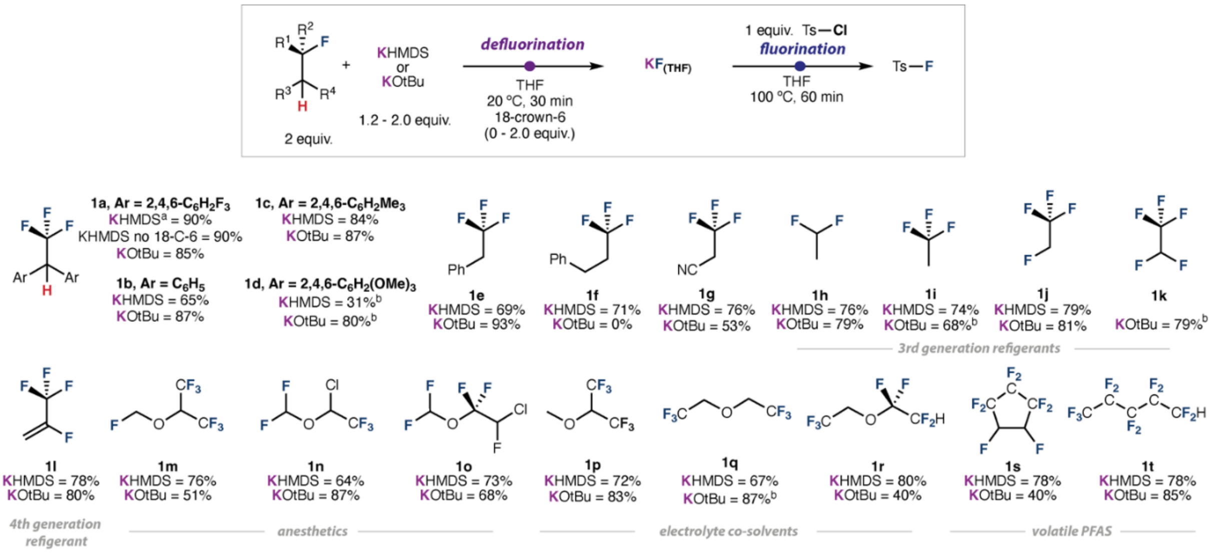

Supplementary Figure 1: List of successful fluorine donors in transfer fluorination.

5.1. Fluorination using **1a** (Procedure A)

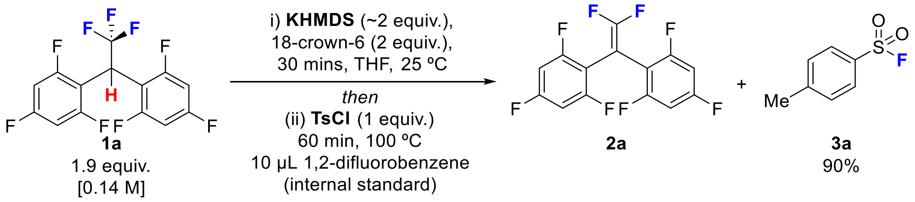

Supplementary Scheme 8: Transfer fluorination using **1a** as a donor.

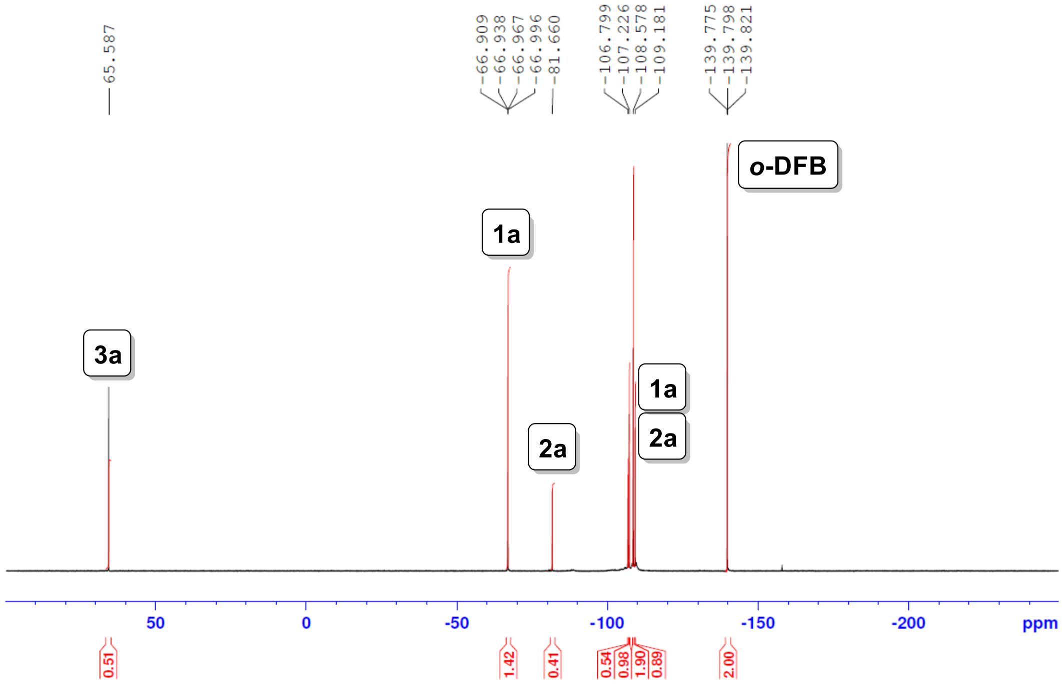

Supplementary Figure 2: ^{19}F NMR spectrum of fluorination of TsCl after 30 minutes activation of **1a** at 25 °C and 60 minutes fluorination at 100 °C (377 MHz, THF-h_8, 25 °C). 0.11 mmol **1a**, 0.12 mmol KHMDS and 0.057 mmol TsCl led to the formation of 0.051 mmol TsF.

5.2. Fluorination using **1b** (Procedure A)

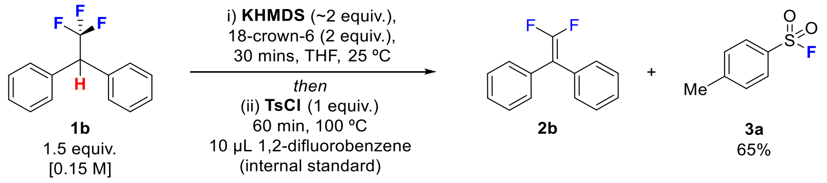

Supplementary Scheme 9: Transfer fluorination using **1b** as a donor.

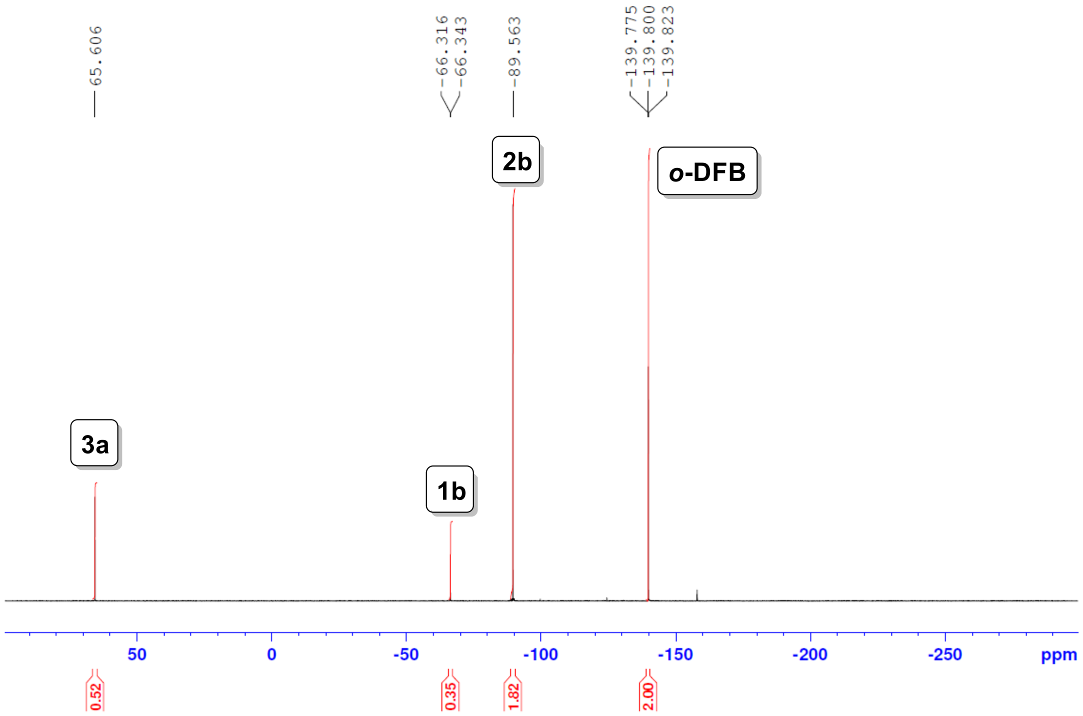

Supplementary Figure 3: ^{19}F NMR spectrum of fluorination of TsCl after 30 minutes activation of **1b** at 25 °C and 60 minutes fluorination at 100 °C (377 MHz, THF-h_8, 25 °C). 0.12 mmol **1b,** 0.17 mmol KHMDS and 0.079 mmol TsCl led to the formation of 0.052 mmol **3a**.

5.3. Fluorination using **1c** (Procedure A)

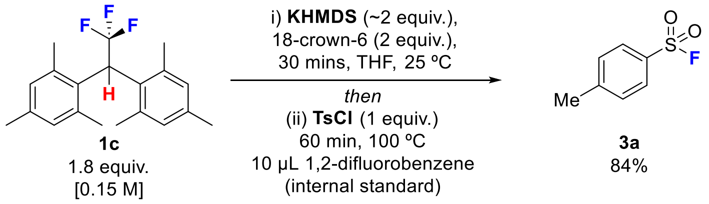

Supplementary Scheme 10: Transfer fluorination using **1c** as a donor.

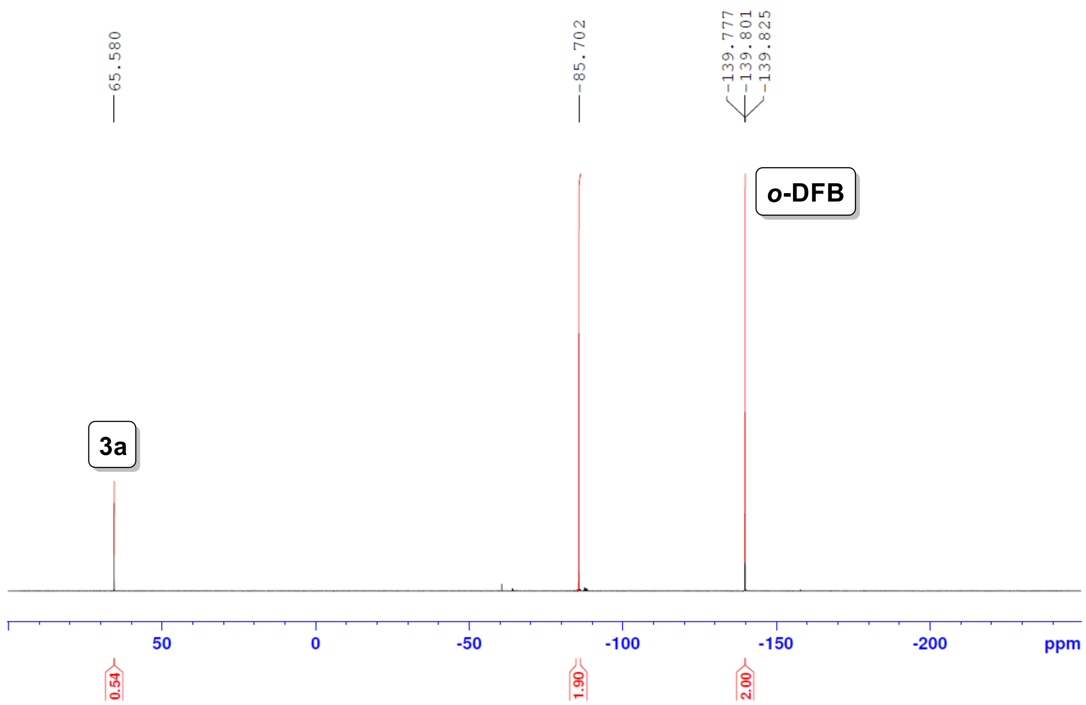

Supplementary Figure 4: ^{19}F NMR spectrum of fluorination of TsCl after 30 minutes activation of **1c** at 25 °C and 60 minutes fluorination at 100 °C (377 MHz, THF-h_8, 25 °C). 0.12 mmol **1c** and 0.065 mmol TsCl led to the formation of 0.054 mmol TsF.

5.4. Fluorination using **1d** (Procedure A)

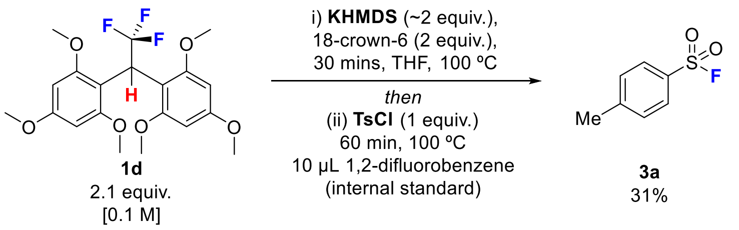

Supplementary Scheme 11: Transfer fluorination using **1d** as a donor.

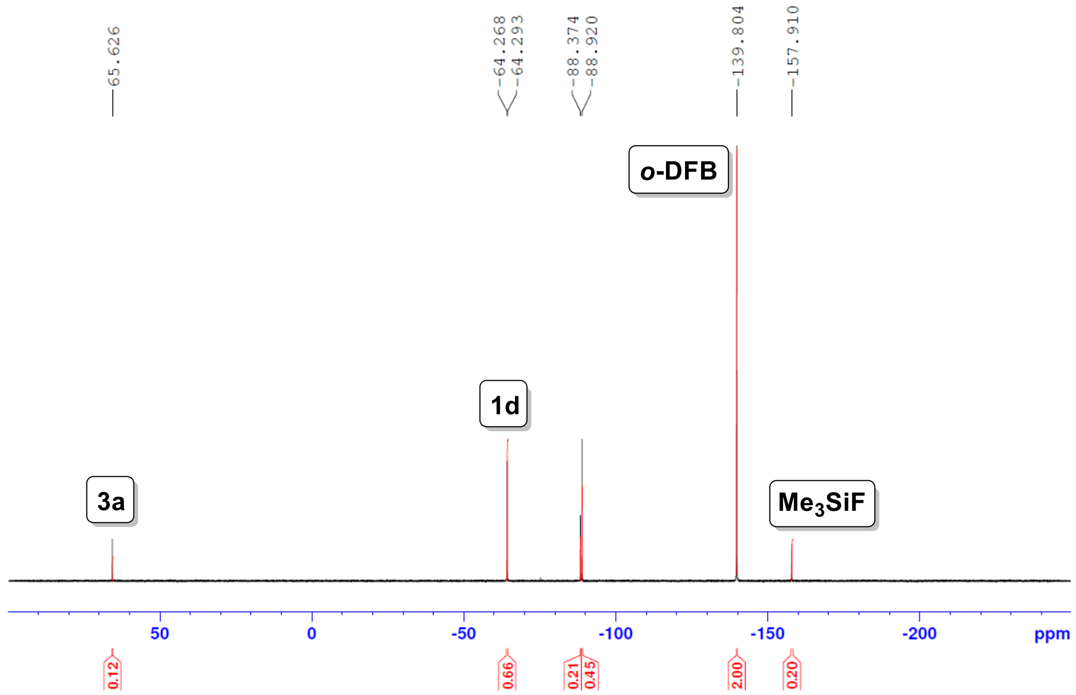

Supplementary Figure 5: ^{19}F NMR spectrum of fluorination of TsCl after 30 minutes activation of **1d** at 100 °C and 60 minutes fluorination at 100 °C (377 MHz, THF-h_8, 25 °C). 0.081 mmol **1d**, 0.081 mmol KHMDS and 0.039 mmol TsCl led to the formation of 0.012 mmol TsF. The formation of Me_3SiF was observed as well, indicating a side-reaction at this temperature.

5.5. Fluorination using **1e** (Procedure A)

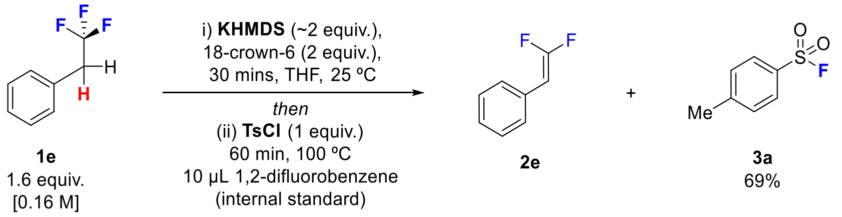

Supplementary Scheme 12: Transfer fluorination using **1e** as a donor.

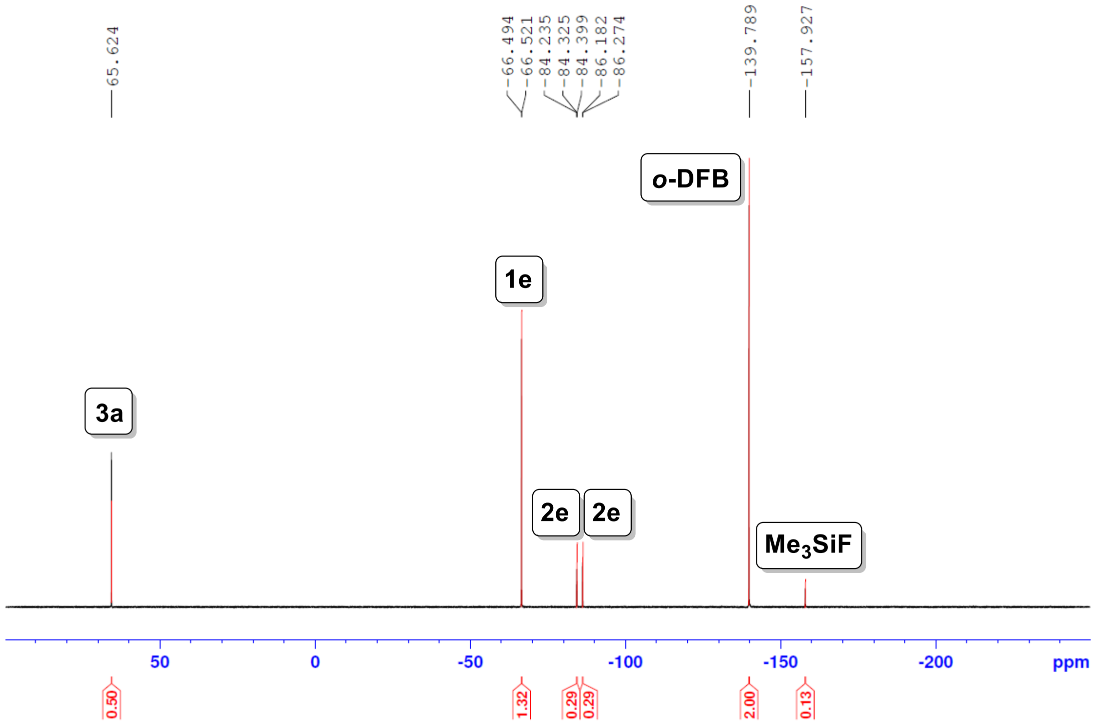

Supplementary Figure 6: ^{19}F NMR spectrum of fluorination of TsCl after 30 minutes activation of **1e** at 25 °C and 60 minutes fluorination at 100 °C (377 MHz, THF-h_8, 25 °C). 0.117 mmol **1e,** 0.14 mmol KHMDS and 0.073 mmol TsCl led to the formation of 0.050 mmol TsF. The formation of Me_3SiF was observed as well, indicating an occurrence of a side-reaction.

5.6. Fluorination using **1f** (Procedure A)

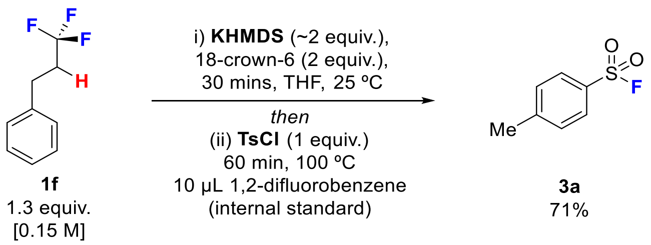

Supplementary Scheme 13: Transfer fluorination using **1f** as a donor.

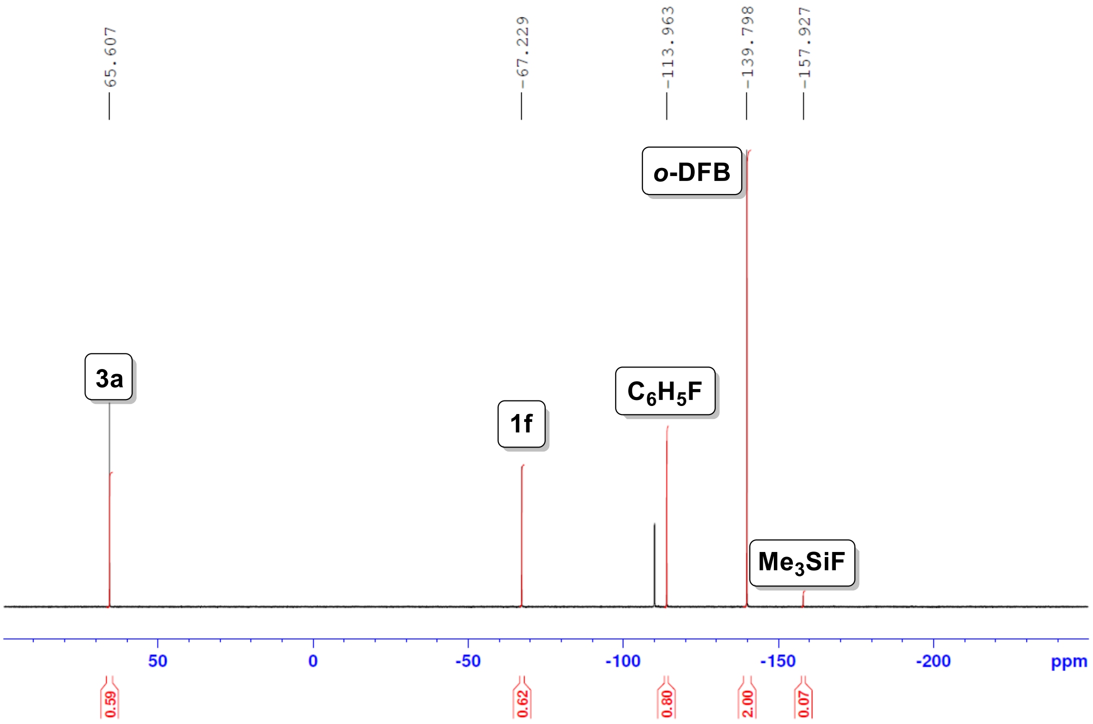

Supplementary Figure 7: ^{19}F NMR spectrum of fluorination of TsCl after 30 minutes activation of **1f** at 25 °C and 60 minutes fluorination at 100 °C (377 MHz, THF-h_8, 25 °C). 0.11 mmol **1f**, 0.15 mmol KHMDS and 0.084 mmol TsCl led to the formation of 0.059 mmol TsF. The formation of a negligible amount of Me_3SiF was observed as well, indicating an occurrence of a side-reaction. Note: Fluorobenzene is present as well due to yield confirmation with second internal standard.

5.7. Fluorination using **1g** (Procedure A)

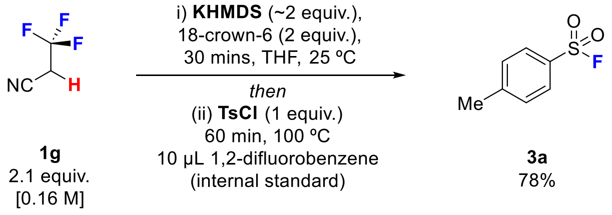

Supplementary Scheme 14: Transfer fluorination using **1g** as a donor.

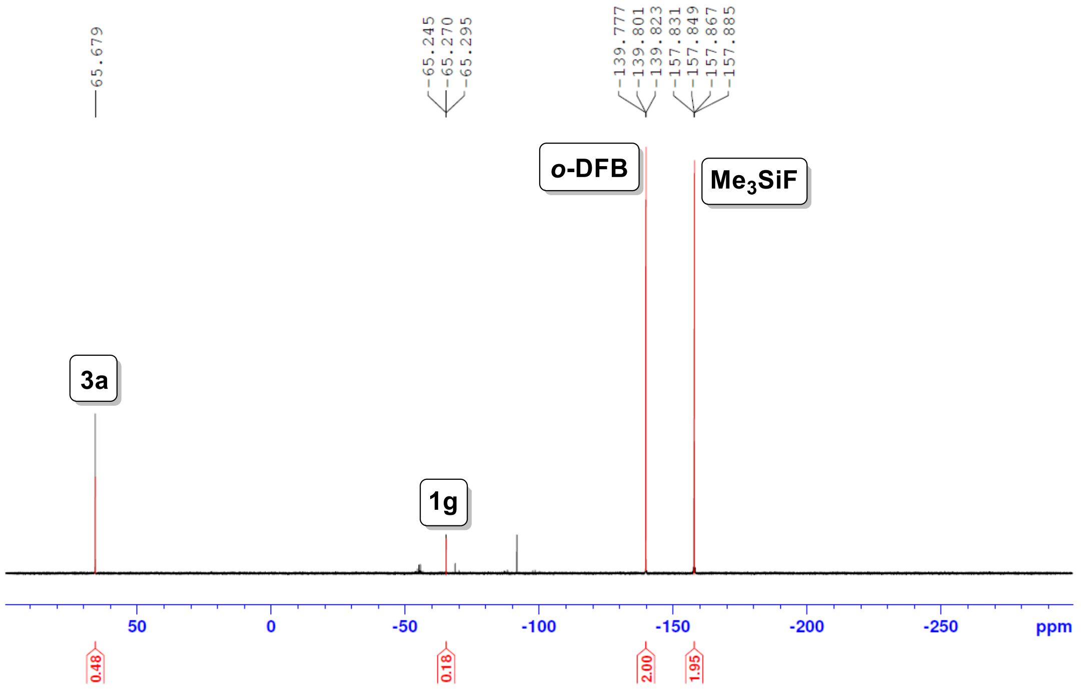

Supplementary Figure 8: ^{19}F NMR spectrum of fluorination of TsCl after 30 minutes activation of **1g** at 25 °C and 60 minutes fluorination at 100 °C (377 MHz, THF-h_8, 25 °C). 0.130 mmol **1g**, 0.125 mmol KHMDS and 0.061 mmol TsCl led to the formation of 0.048 mmol TsF. The formation of a large amount of Me_3SiF was observed as well, indicating an occurrence of a side-reaction.

5.8. Fluorination using **1h**: HFC-152a (Procedure B)

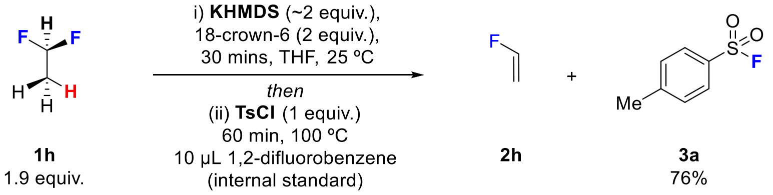

Supplementary Scheme 15: Transfer fluorination using **1h** as a donor.

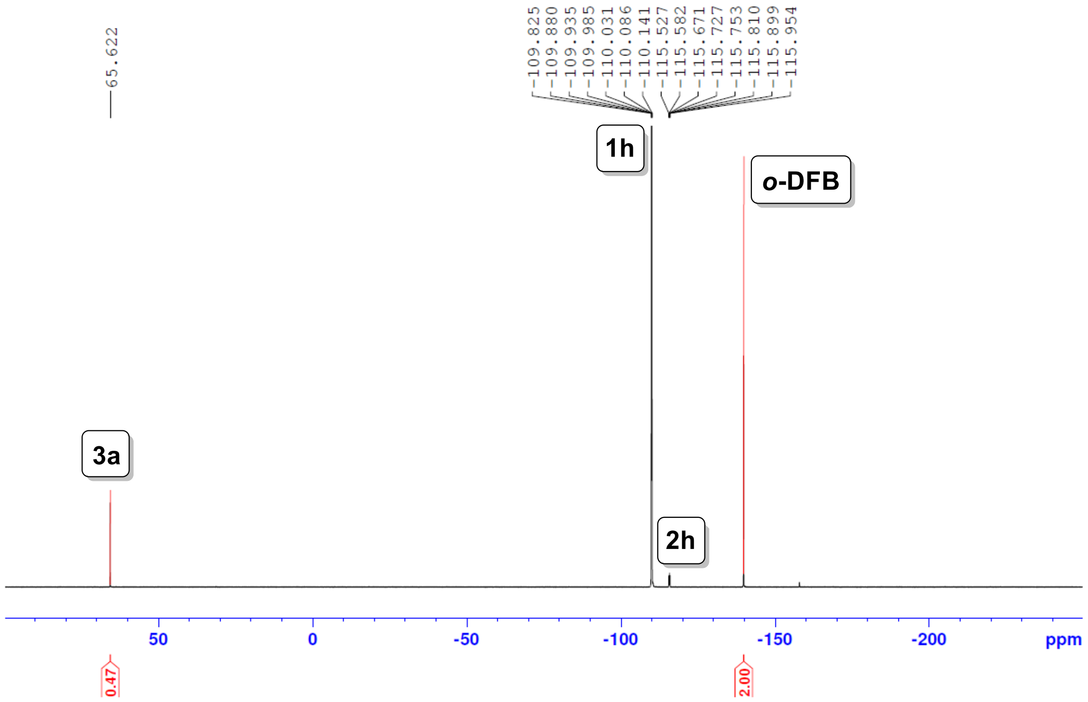

Supplementary Figure 9: ^{19}F NMR spectrum of fluorination of TsCl after 30 minutes activation of **1h** at 25 °C and 60 minutes fluorination at 100 °C (377 MHz, THF-h_8, 25 °C). 0.120 mmol **1h,** 0.120 mmol KHMDS and 0.062 mmol TsCl led to the formation of 0.047 mmol TsF. The negligible formation of Me_3SiF was observed as well.

5.9. Fluorination using **1i:** HFC-143a (Procedure B)

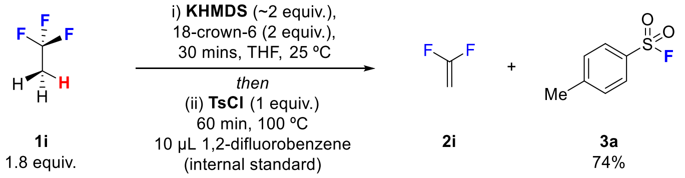

Supplementary Scheme 16: Transfer fluorination using **1i** as a donor.

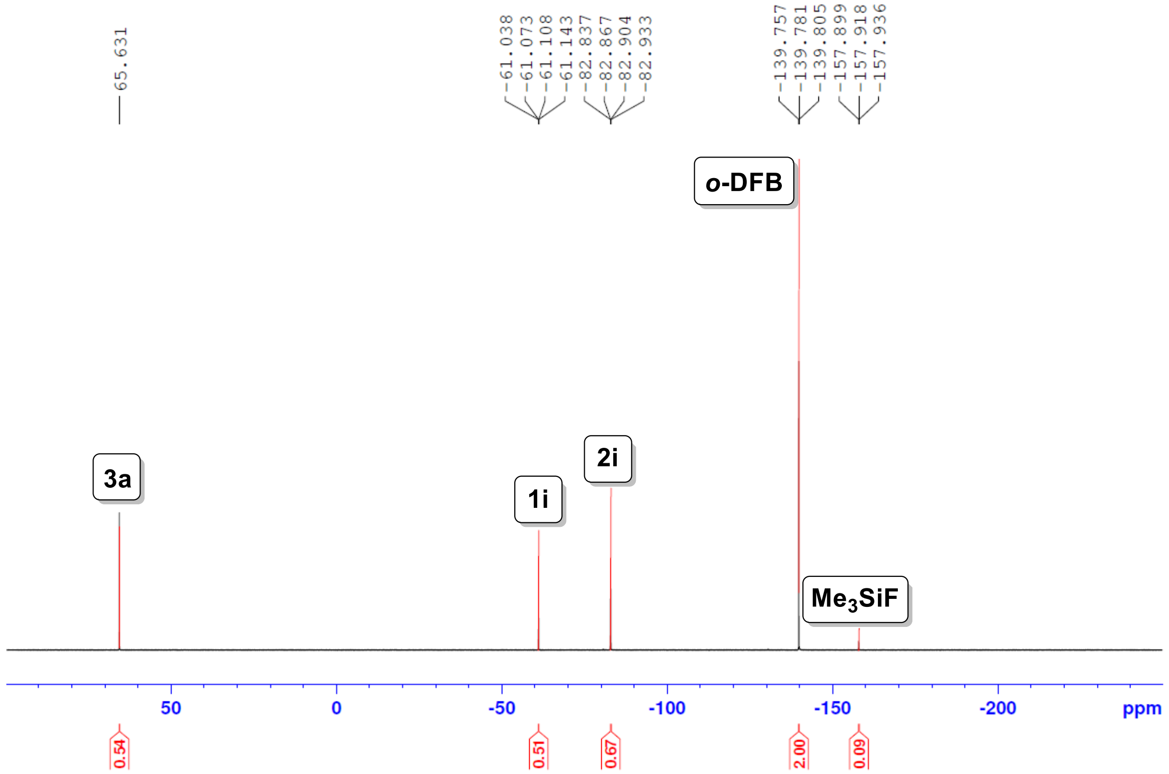

Supplementary Figure 10: ^{19}F NMR spectrum of fluorination of TsCl after 30 minutes activation of **1i** at 25 °C and 60 minutes fluorination at 100 °C (377 MHz, THF-h_8, 25 °C). 0.151 mmol **1i,** 0.155 mmol KHMDS and 0.082 mmol TsCl led to the formation of 0.065 mmol TsF. The formation of Me_3SiF was also observed.

5.10. Fluorination using **1j**: HFC-134a (Procedure B)

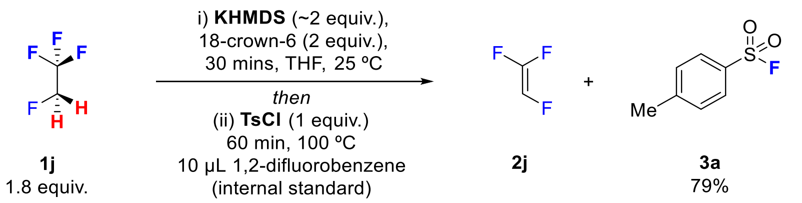

Supplementary Scheme 17: Transfer fluorination using **1j** as a donor.

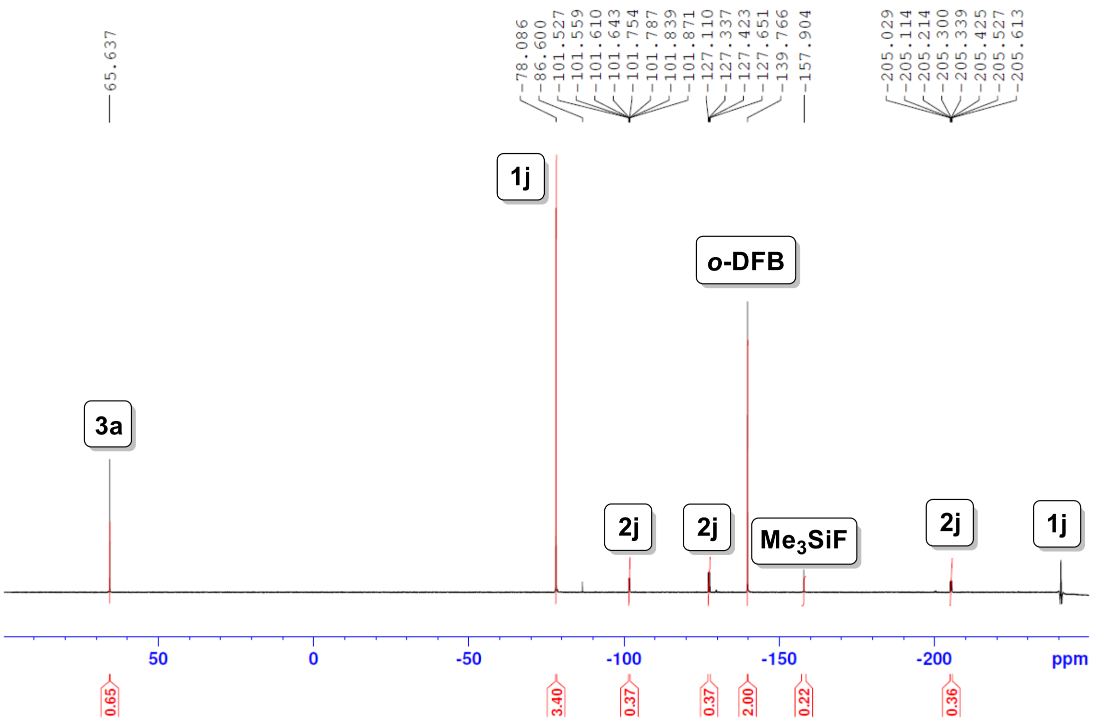

Supplementary Figure 11: ^{19}F NMR of fluorination of TsCl after 30 minutes activation of **1j** at 25 °C and 60 minutes fluorination at 100 °C (377 MHz, THF-h_8, 25 °C). 0.151 mmol **1j**, 0.150 mmol KHMDS and 0.082 mmol TsCl led to the formation of 0.065 mmol TsF. The formation of Me_3SiF was observed as well. Note: Signal at -240.6 ppm was not integrated due to phasing error.

5.11. Fluorination using **1l**: HFO-1234yf (Procedure B)

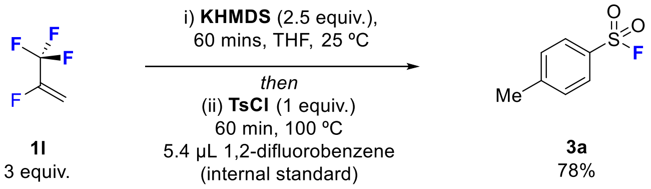

Supplementary Scheme 19: Transfer fluorination using **1l** as a donor.

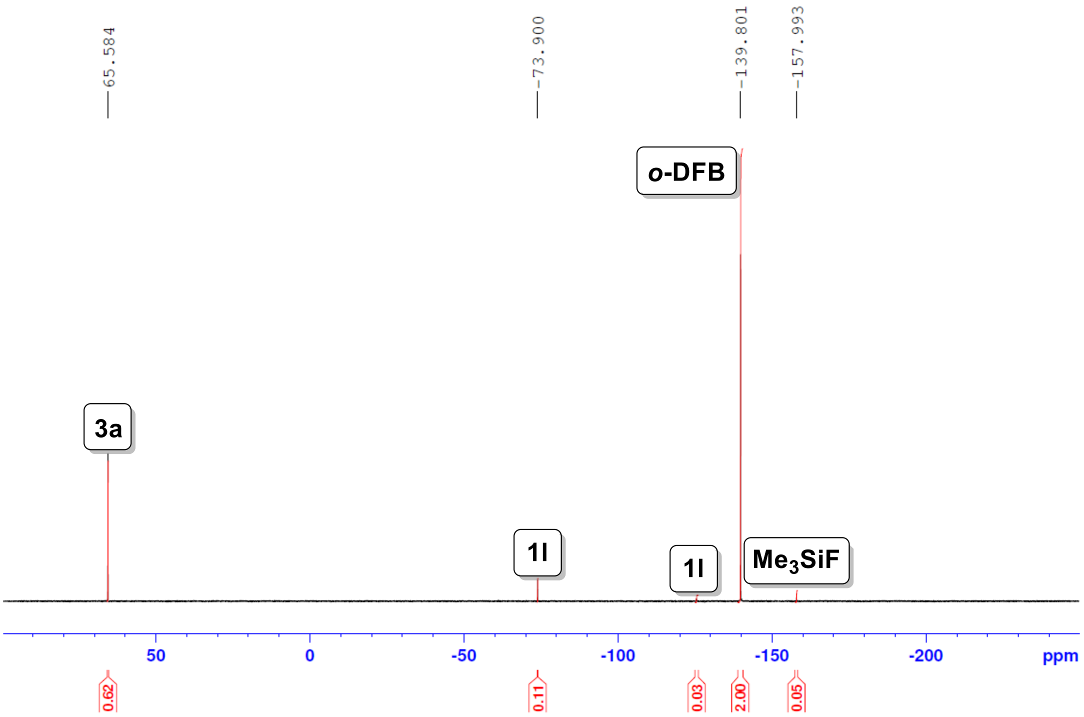

Supplementary Figure 13: ^{19}F NMR spectrum of fluorination of TsCl after 60 minutes activation of **1l** at 25 °C and 60 minutes fluorination at 100 °C (377 MHz, THF-h_8, 25 °C). 0.130 mmol **1l**, 0.110 mmol KHMDS and 0.044 mmol TsCl led to the formation of 0.034 mmol TsF. The formation of Me_3SiF was observed as well.

5.12. Fluorination using **1m**: Sevoflurane (Procedure A)

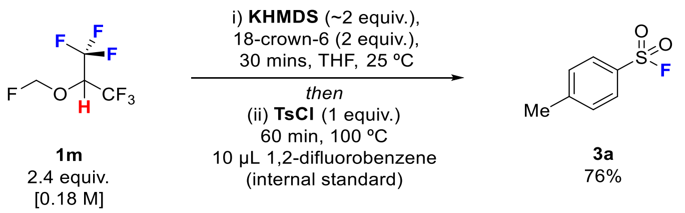

Supplementary Scheme 20: Transfer fluorination using **1m** as a donor.

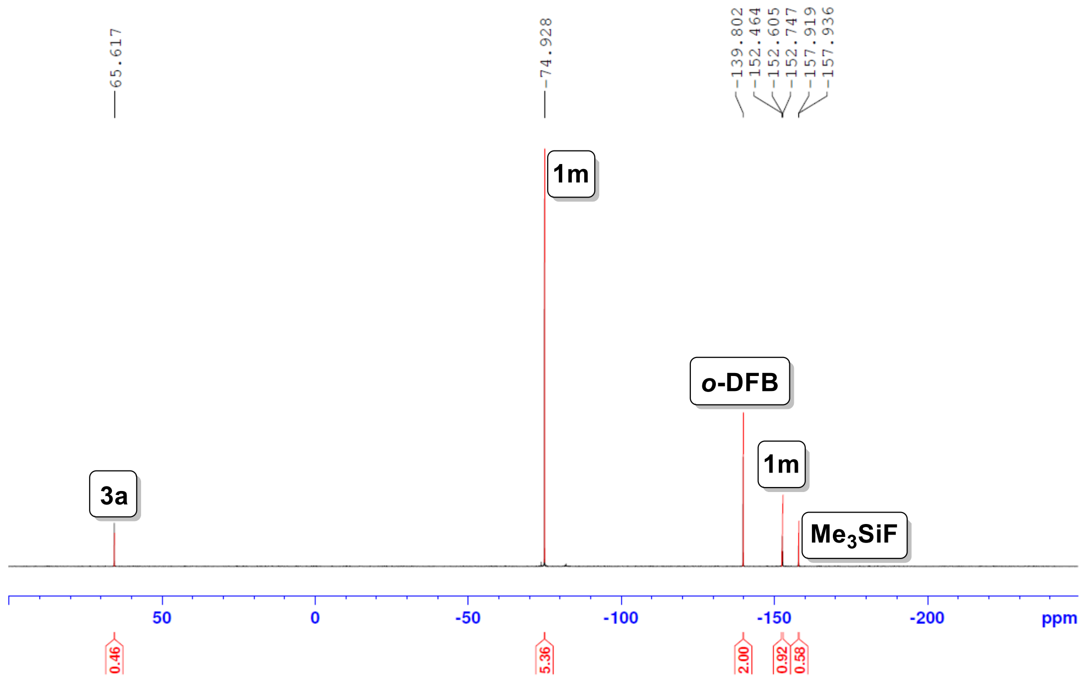

Supplementary Figure 14: ^{19}F NMR spectrum of fluorination of TsCl after 30 minutes activation of **1m** at 25 °C and 60 minutes fluorination at 100 °C (377 MHz, THF-h_8, 25 °C). 0.145 mmol **1m**, 0.120 mmol KHMDS and 0.061 mmol TsCl led to the formation of 0.046 mmol TsF. The formation of Me_3SiF was observed as well.

5.13. Fluorination using **1n**: Isoflurane (Procedure A)

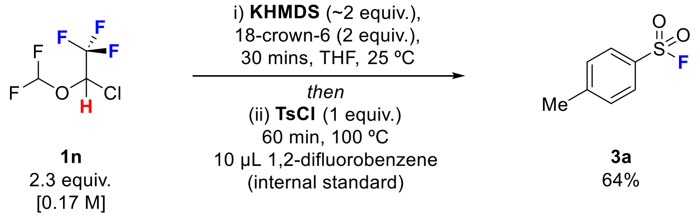

Supplementary Scheme 21: Transfer fluorination using **1n** as a donor.

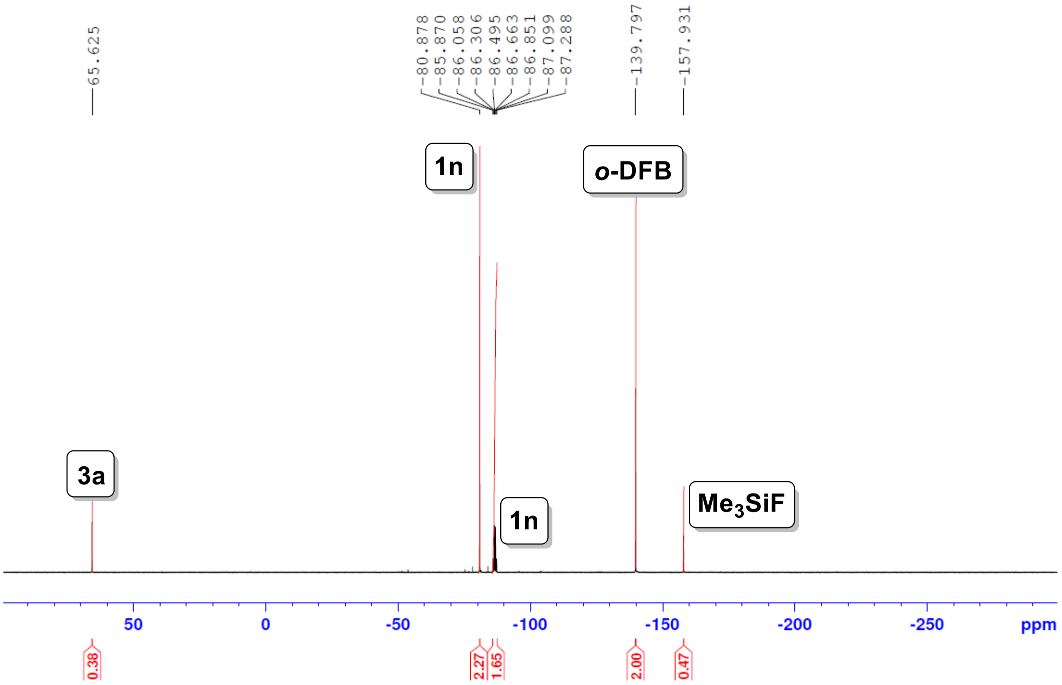

Supplementary Figure 15: ^{19}F NMR spectrum of fluorination of TsCl after 30 minutes activation of **1n** at 25 °C and 60 minutes fluorination at 100 °C (377 MHz, THF-h_8, 25 °C). 0.135 mmol **1n**, 0.120 mmol KHMDS and 0.060 mmol TsCl led to the formation of 0.038 mmol TsF. The formation of Me_3SiF was observed as well.

5.14. Fluorination using **1o**: Enflurane (Procedure A)

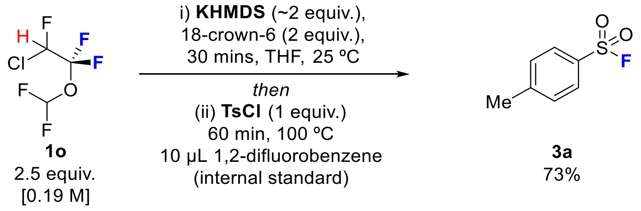

Supplementary Scheme 22: Transfer fluorination using **1o** as a donor.

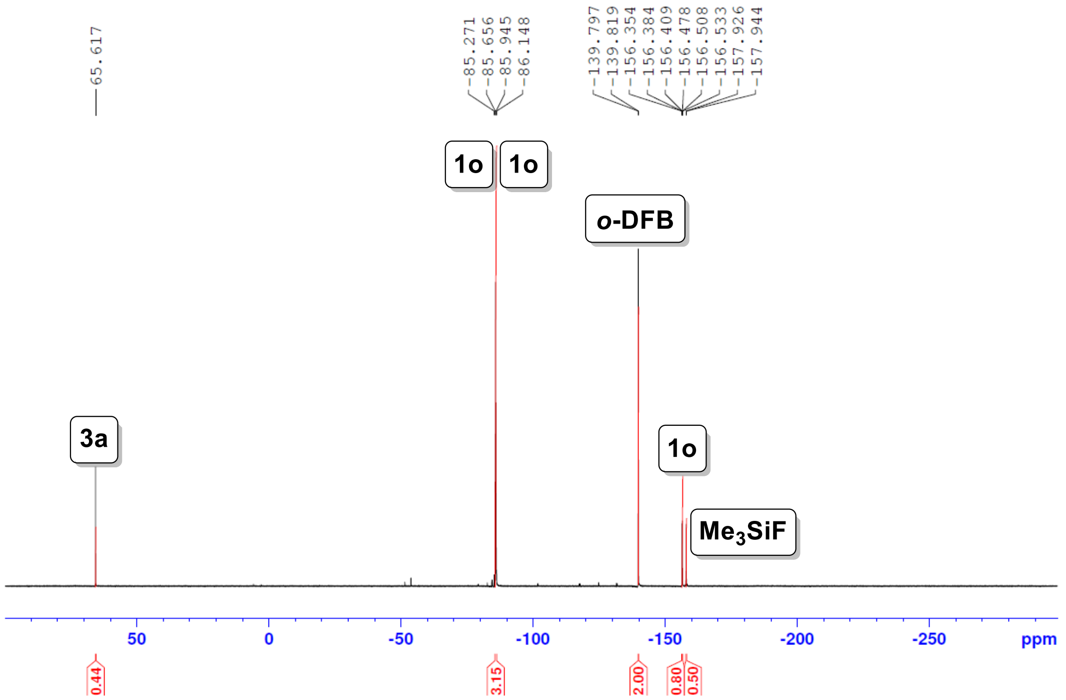

Supplementary Figure 16: ^{19}F NMR spectrum of fluorination of TsCl after 30 minutes activation of **1o** at 25 °C and 60 minutes fluorination at 100 °C (377 MHz, THF-h$_8$, 25 °C). 0.151 mmol **1o**, 0.120 mmol KHMDS and 0.060 mmol TsCl led to the formation of 0.044 mmol TsF. The formation of Me_3SiF was observed as well.

5.15. Fluorination using **1p**: Hexafluoroisopropyl methyl ether (Procedure A)

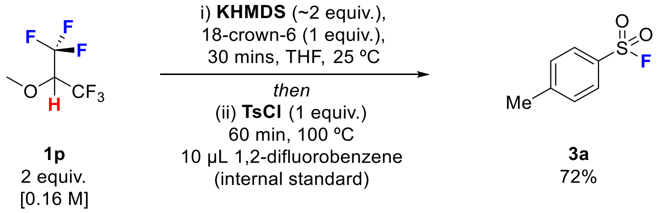

Supplementary Scheme 23: Transfer fluorination using **1p** as a donor.

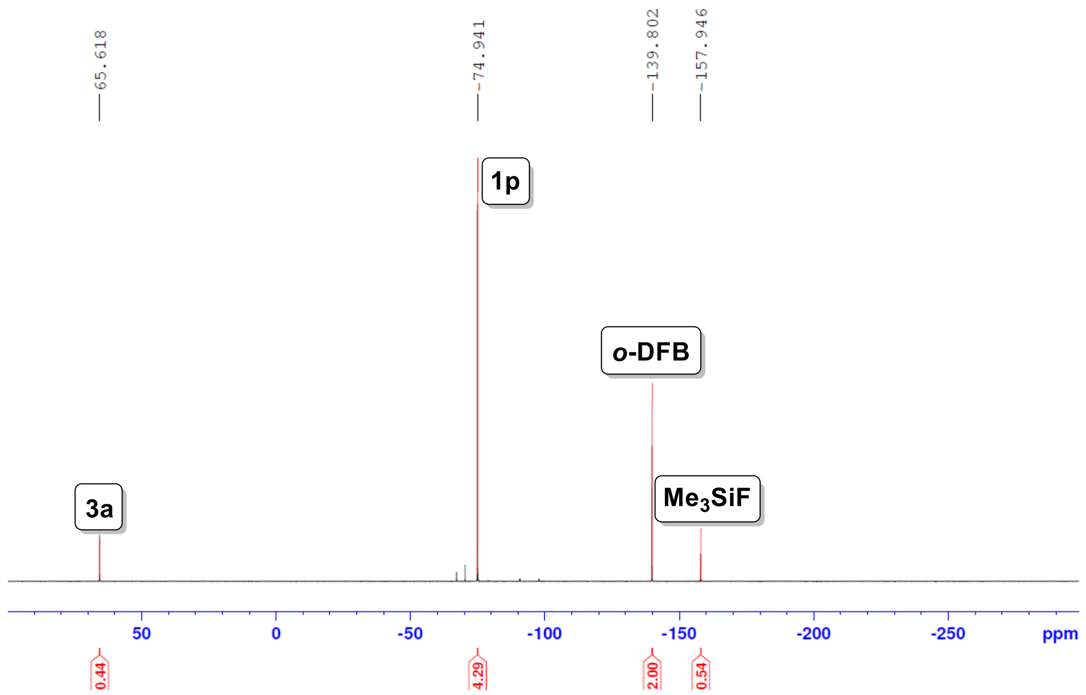

Supplementary Figure 17: ^{19}F NMR spectrum of fluorination of TsCl after 30 minutes activation of **1p** at 25 °C and 60 minutes fluorination at 100 °C (377 MHz, THF-h_8, 25 °C). 0.125 mol **1p**, 0.128 mmol KHMDS and 0.061 mmol TsCl led to the formation of 0.044 mmol TsF. The formation of Me_3SiF was observed as well.

5.16. Fluorination using **1q**: Bis(2,2,2-trifluoroethyl) ether (Procedure A)

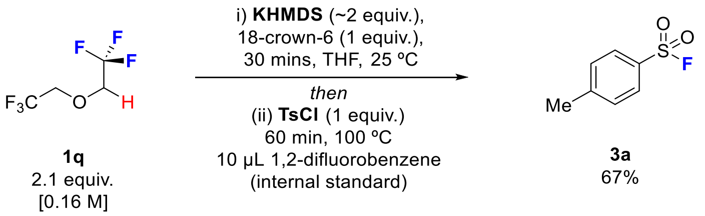

Supplementary Scheme 24: Transfer fluorination using **1q** as a donor.

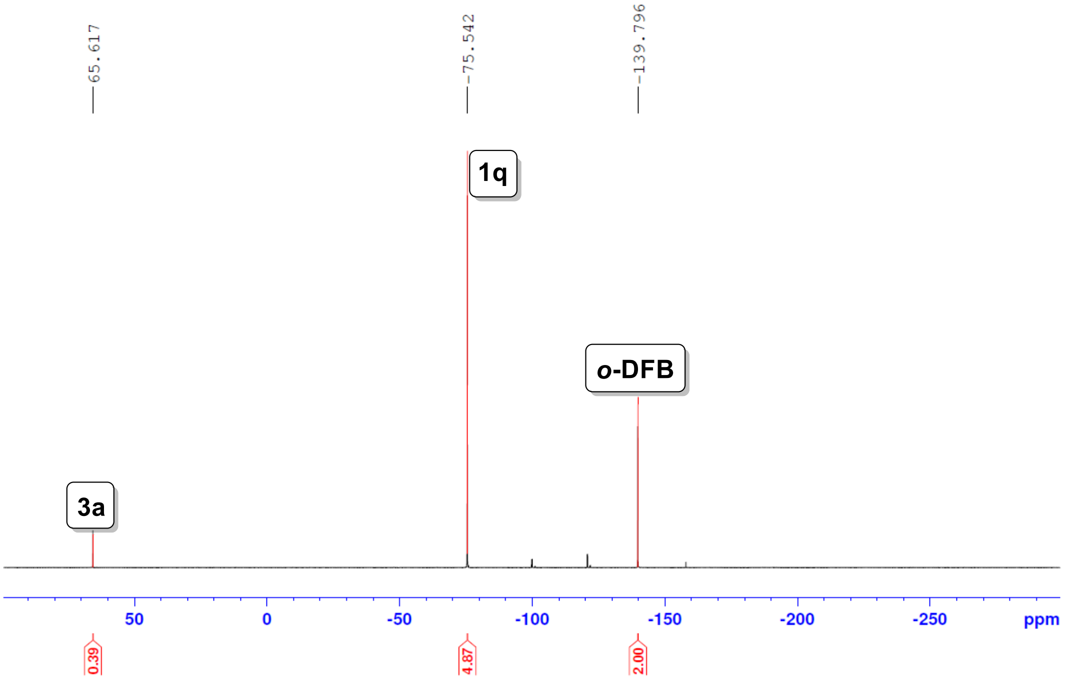

Supplementary Figure 18: ^{19}F NMR spectrum of fluorination of TsCl after 30 minutes activation of **1q** at 25 °C and 60 minutes fluorination at 100 °C (377 MHz, THF-h_8, 25 °C). 0.125 mmol **1q**, 0.125 mmol KHMDS and 0.059 mmol TsCl led to the formation of 0.039 mmol TsF. A negligible amount of Me_3SiF did form as well.

5.17. Fluorination using **1r**: TFTFE (Procedure A)

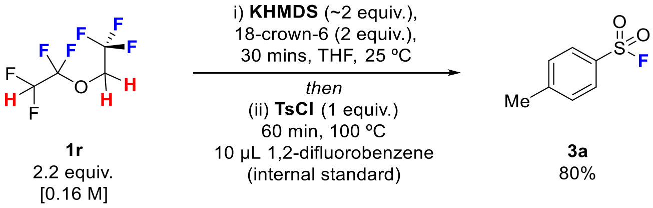

Supplementary Scheme 25: Transfer fluorination using **1r** as a donor.

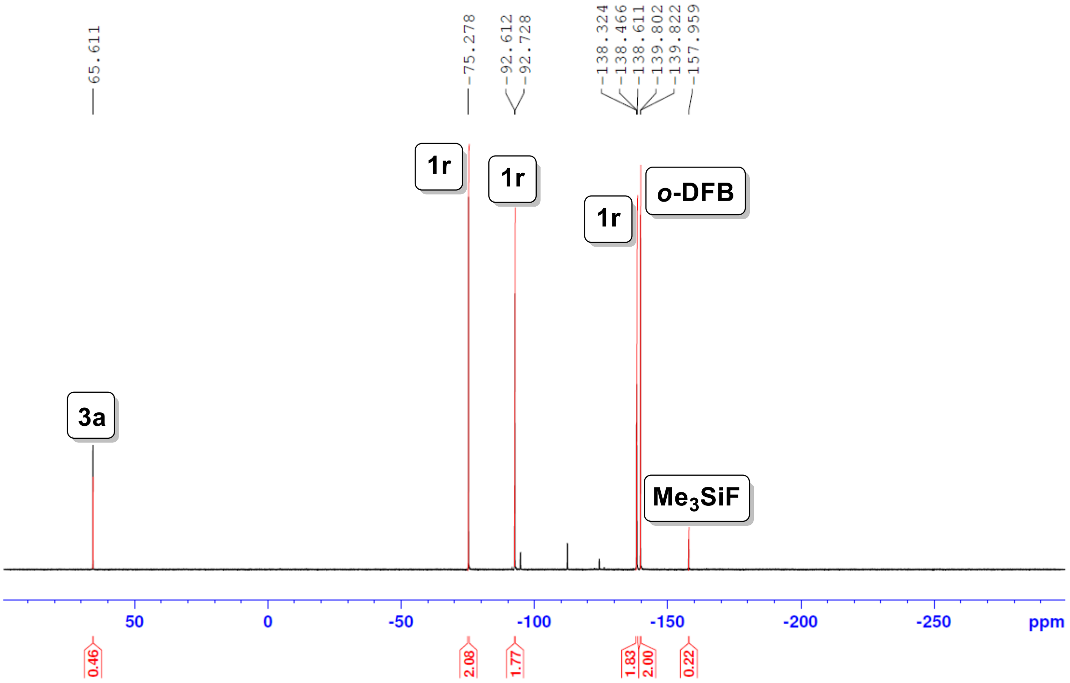

Supplementary Figure 19: ^{19}F NMR spectrum of fluorination of TsCl after 30 minutes activation of **1r** at 25 °C and 60 minutes fluorination at 100 °C (377 MHz, THF-h_8, 298 K). 0.125 mmol **1r**, 0.129 mmol KHMDS and 0.057 mmol TsCl led to the formation of 0.046 mmol TsF. The formation of Me_3SiF was observed as well.

5.18. Fluorination using **1s**: Volatile PFAS (Procedure A)

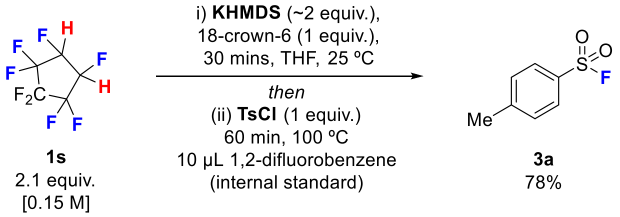

Supplementary Scheme 26: Transfer fluorination using **1s** as a donor.

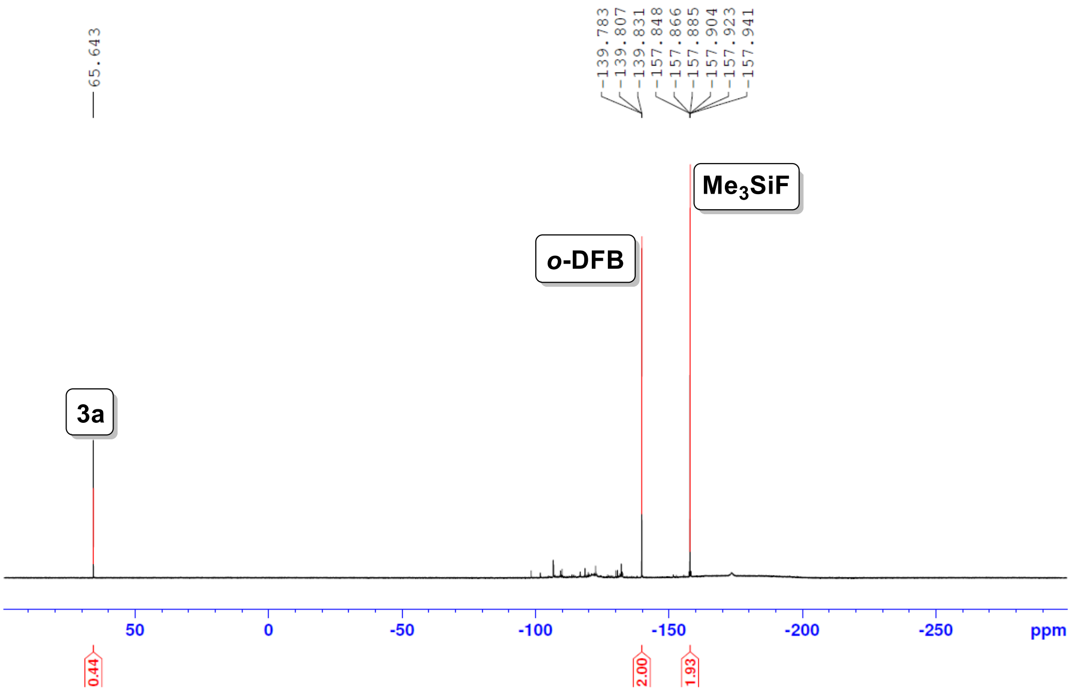

Supplementary Figure 20: ^{19}F NMR spectrum of fluorination of TsCl after 30 minutes activation of **1s** at 25 °C and 60 minutes fluorination at 100 °C (377 MHz, THF-h_8, 25 °C). 0.120 mmol **1s**, 0.120 mmol KHMDS and 0.057 mmol TsCl led to the formation of 0.044 mmol TsF. A significant amount of Me_3SiF was observed as well.

5.19. Fluorination using **1t**: Volatile PFAS (Procedure A)

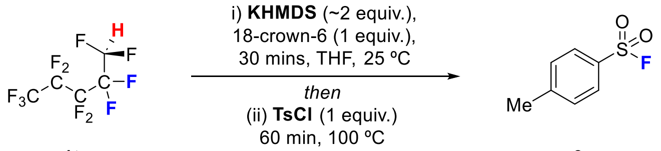

1t
2 equiv.

3a
84%

Supplementary Scheme 27: Transfer fluorination using **1t** as a donor.

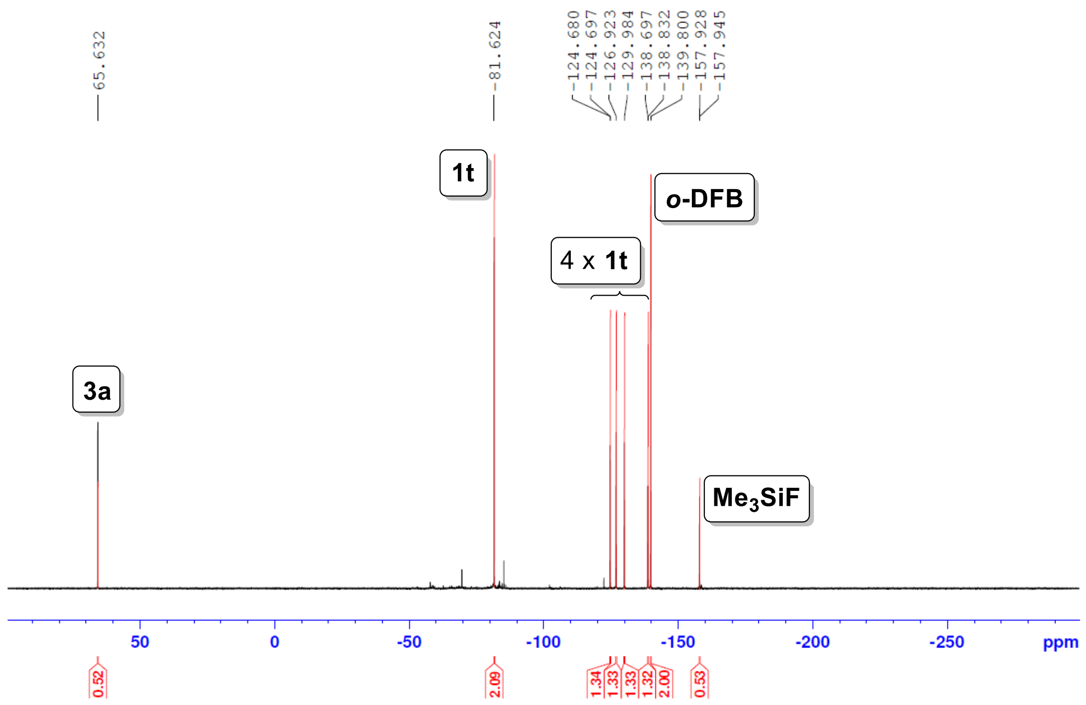

Supplementary Figure 21: ^{19}F NMR spectrum of fluorination of TsCl after 30 minutes activation of **1t** at 25 °C and 60 minutes fluorination at 100 °C (377 MHz, THF-h_8, 298 K). 0.122 mmol **1t,** 0.120 mmol KHMDS and 0.062 mmol TsCl led to the formation of 0.052 mmol TsF. The formation of Me_3SiF was observed as well.

5.20 Fluorination using **1a** (Procedure C)

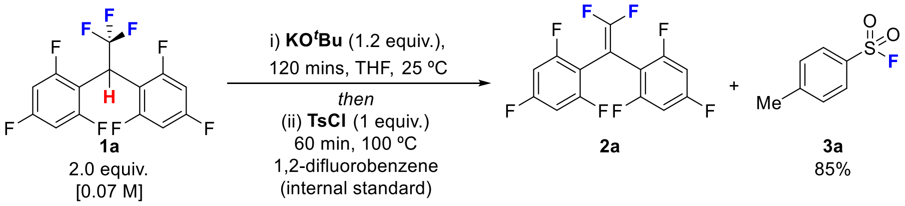

Supplementary Scheme 28: Transfer fluorination using **1a** as a donor.

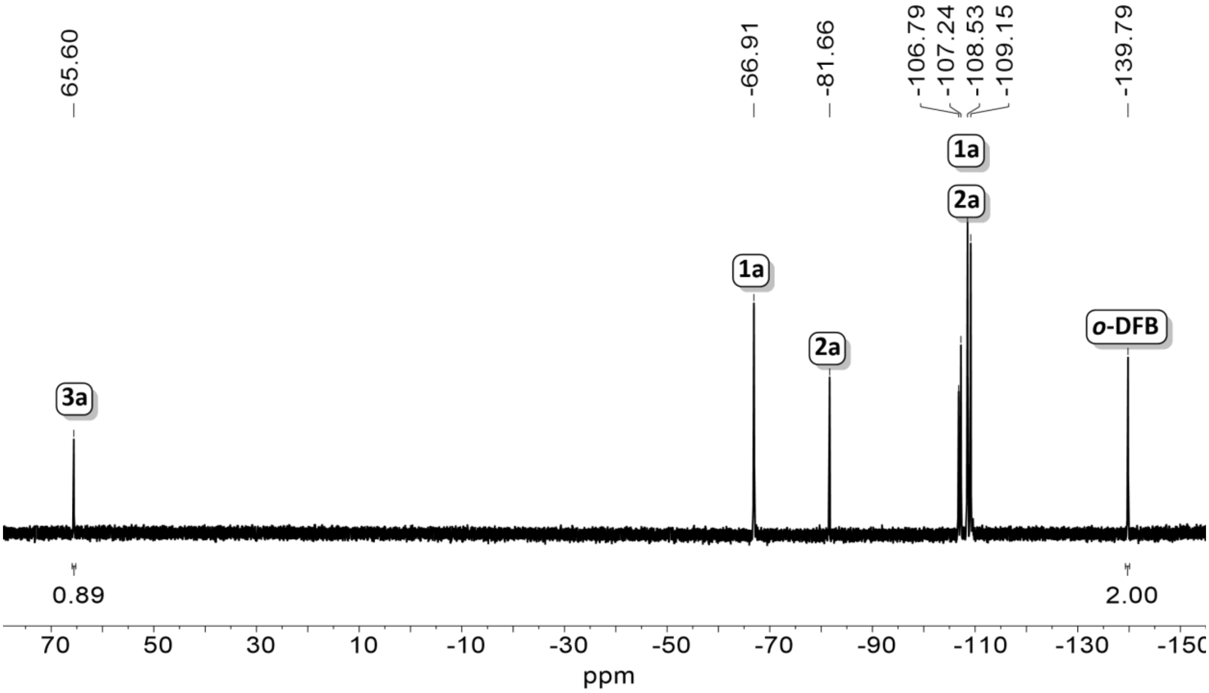

Supplementary Figure 22: ^{19}F NMR spectrum of fluorination of TsCl after 120 minutes activation of **1a** at 25 °C and 60 minutes fluorination at 100 °C (377 MHz, THF-h_8, 25 °C). 0.04 mmol **1a**, 0.025 mmol KO^{t}Bu and 0.021 mmol TsCl led to the formation of 0.018 mmol TsF.

5.21 Fluorination using **1b** (Procedure C)

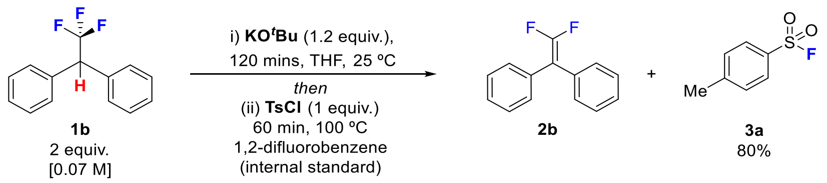

Supplementary Scheme 29: Transfer fluorination using **1b** as a donor.

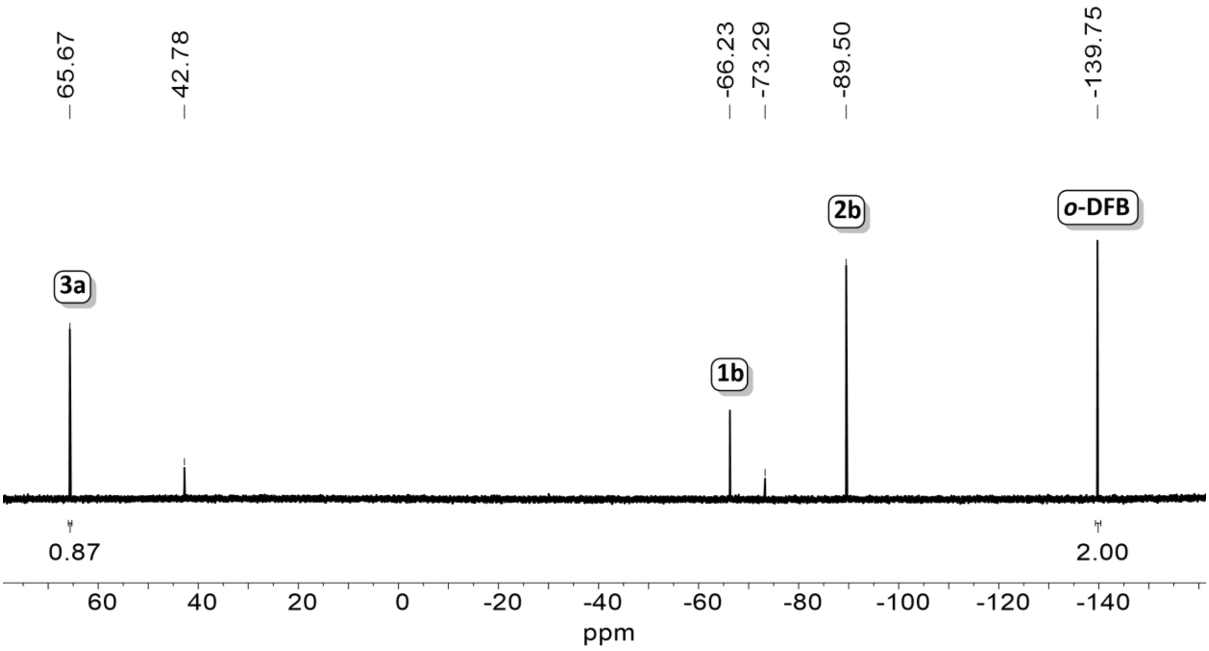

Supplementary Figure 23: ^{19}F NMR spectrum of fluorination of TsCl after 120 minutes activation of **1b** at 25 °C and 60 minutes fluorination at 100 °C (377 MHz, THF-h_8, 25 °C). 0.042 mmol **1b**, 0.025 mmol KO^{t}Bu and 0.021 mmol TsCl led to the formation of 0.018 mmol TsF.

5.22 Fluorination using **1c** (Procedure C)

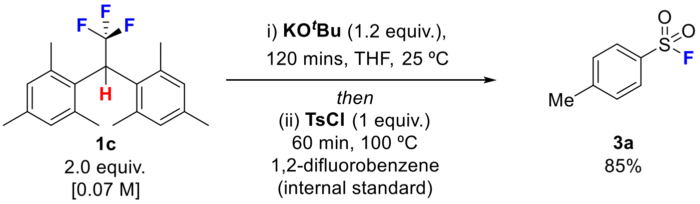

Supplementary Scheme 30: Transfer fluorination using **1c** as a donor.

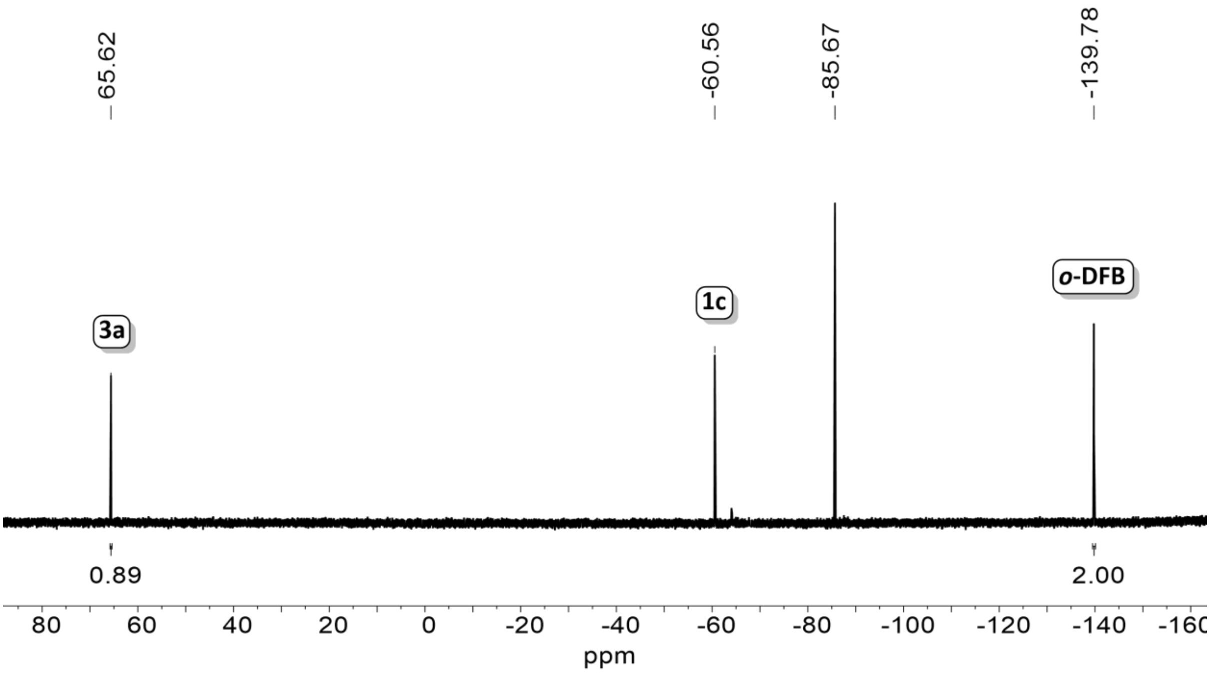

Supplementary Figure 24: ^{19}F NMR spectrum of fluorination of TsCl after 120 minutes activation of **1c** at 25 °C and 60 minutes fluorination at 100 °C (377 MHz, THF-h_8, 25 °C). 0.042 mmol **1c**, 0.025 mmol KO^{t}Bu and 0.021 mmol TsCl led to the formation of 0.018 mmol TsF.

5.23 Fluorination using **1d** (Procedure C)

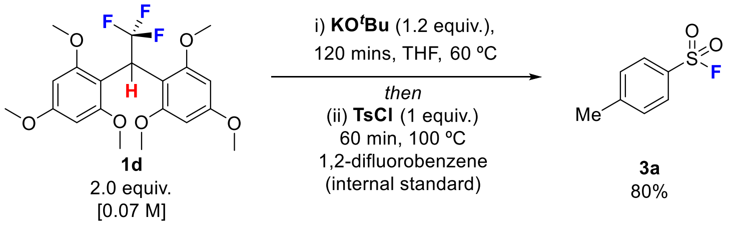

Supplementary Scheme 31: Transfer fluorination using **1d** as a donor.

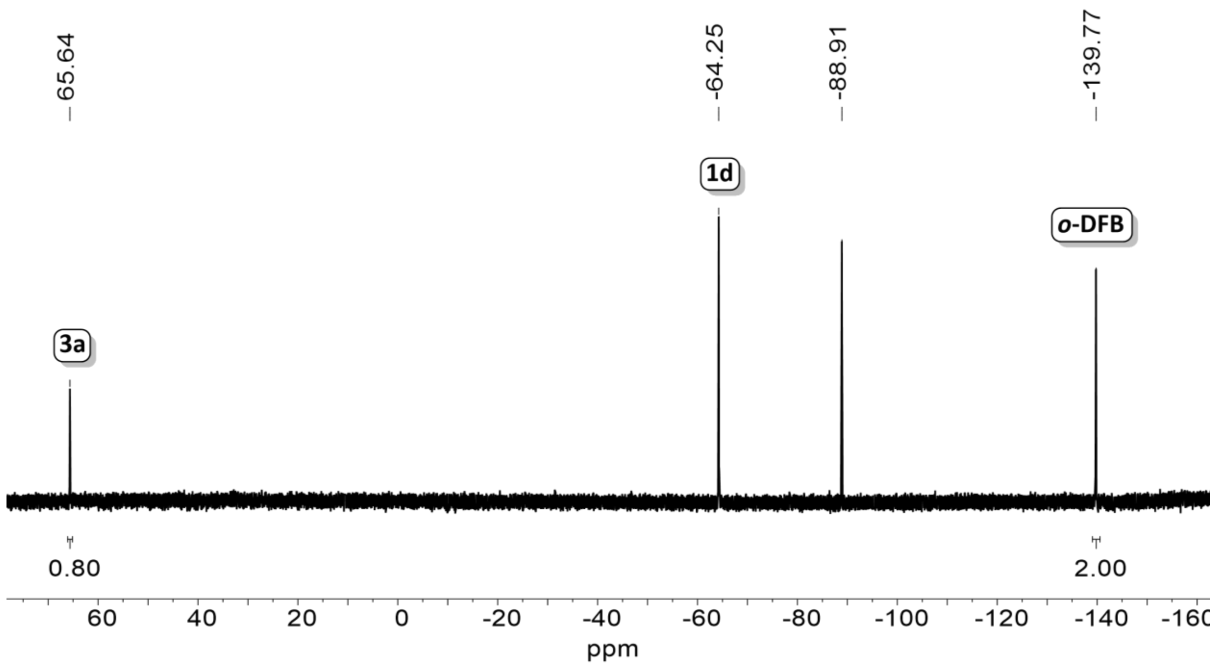

Supplementary Figure 25: ^{19}F NMR spectrum of fluorination of TsCl after 120 minutes activation of **1d** at 60 °C and 60 minutes fluorination at 100 °C (377 MHz, THF-h_8, 25 °C). 0.042 mmol **1d**, 0.025 mmol KO^{t}Bu and 0.021 mmol TsCl led to the formation of 0.017 mmol TsF.

5.24 Fluorination using **1e** (Procedure C)

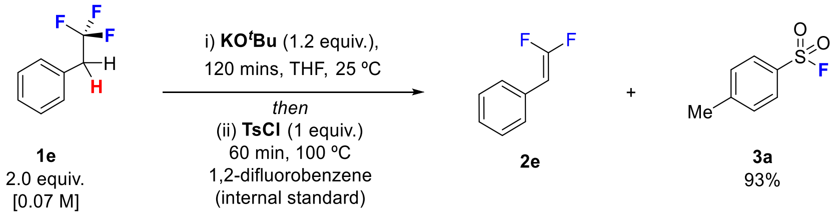

Supplementary Scheme 32: Transfer fluorination using **1e** as a donor.

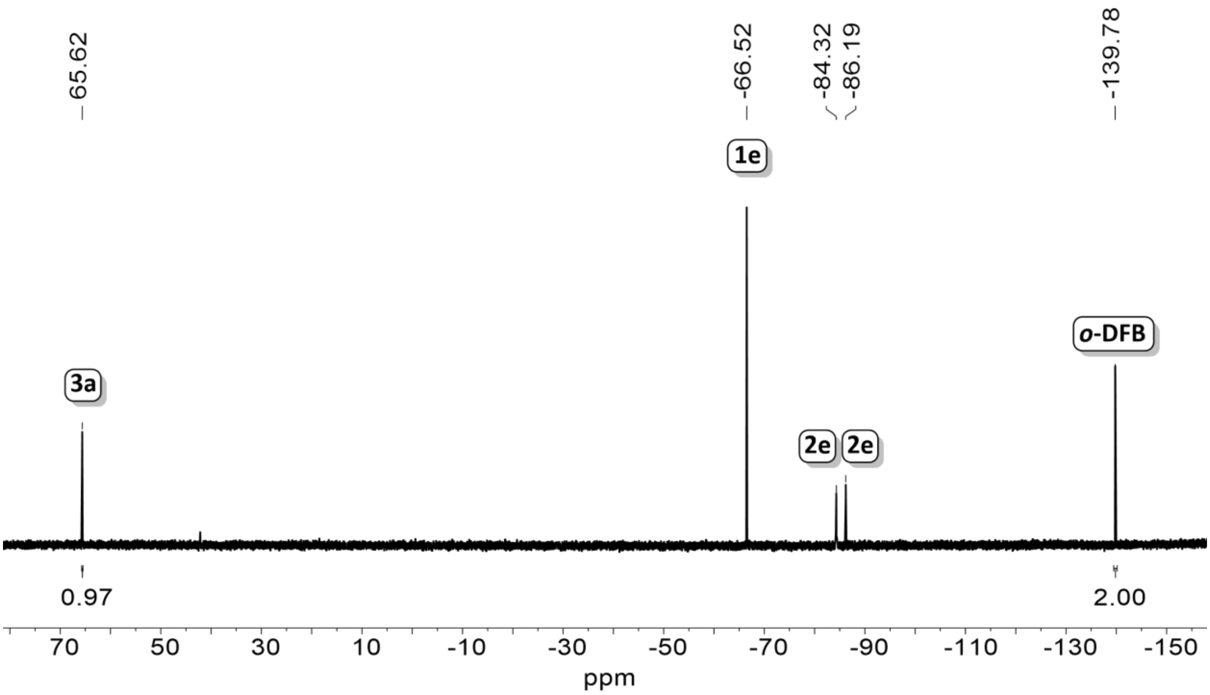

Supplementary Figure 26: ^{19}F NMR spectrum of fluorination of TsCl after 120 minutes activation of **1e** at 25 °C and 60 minutes fluorination at 100 °C (377 MHz, THF-h_8, 25 °C). 0.042 mmol **1e**, 0.025 mmol KO^{t}Bu and 0.021 mmol TsCl led to the formation of 0.019 mmol TsF.

5.25 Fluorination using **1g** (Procedure C)

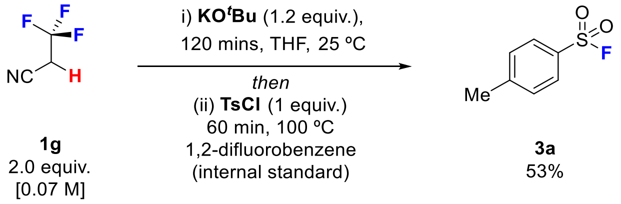

Supplementary Scheme 33: Transfer fluorination using **1g** as a donor.

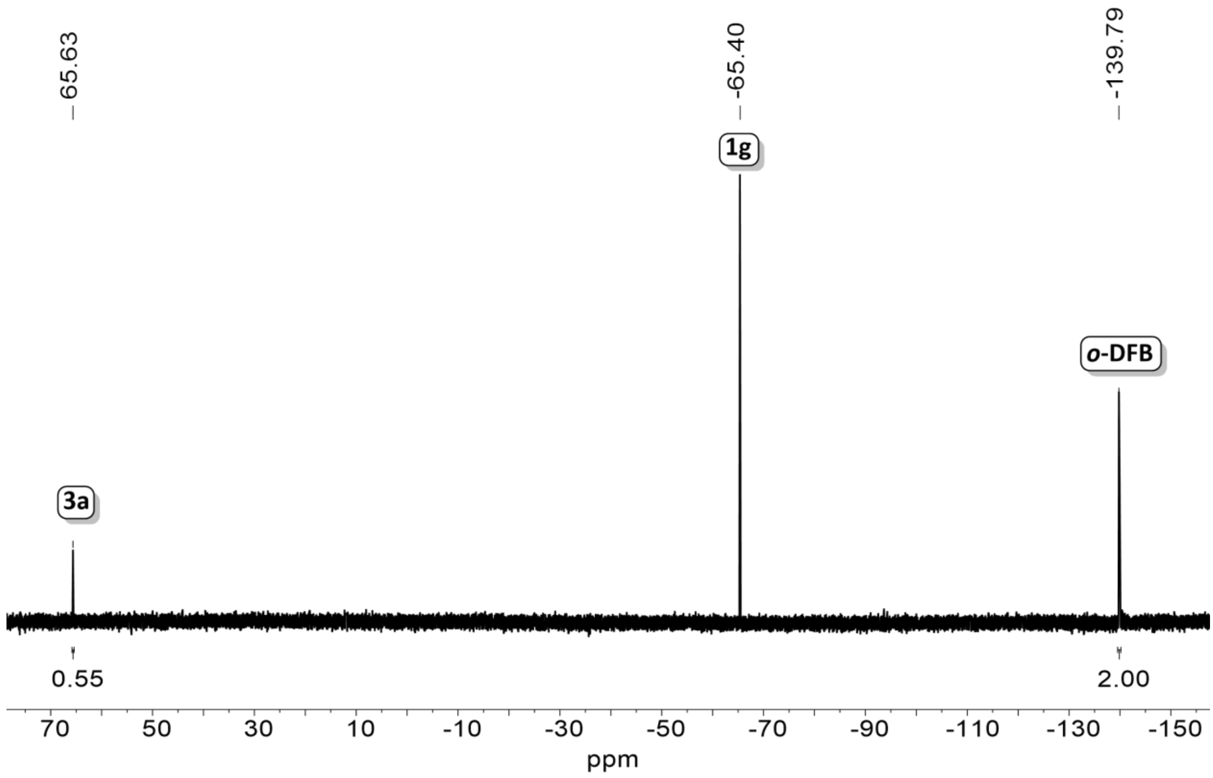

Supplementary Figure 27: ^{19}F NMR spectrum of fluorination of TsCl after 120 minutes activation of **1g** at 25 °C and 60 minutes fluorination at 100 °C (377 MHz, THF-h_8, 25 °C). 0.042 mmol **1g**, 0.025 mmol KO^{t}Bu and 0.021 mmol TsCl led to the formation of 0.011 mmol TsF.

5.26 Fluorination using **1h**: HFC-152a (Procedure D)

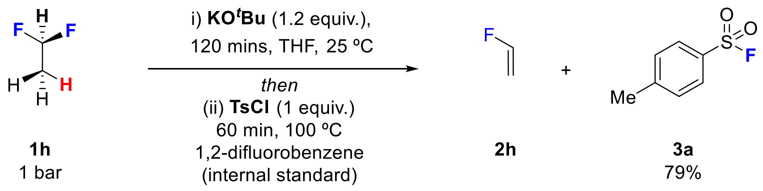

Supplementary Scheme 34: Transfer fluorination using **1h** as a donor.

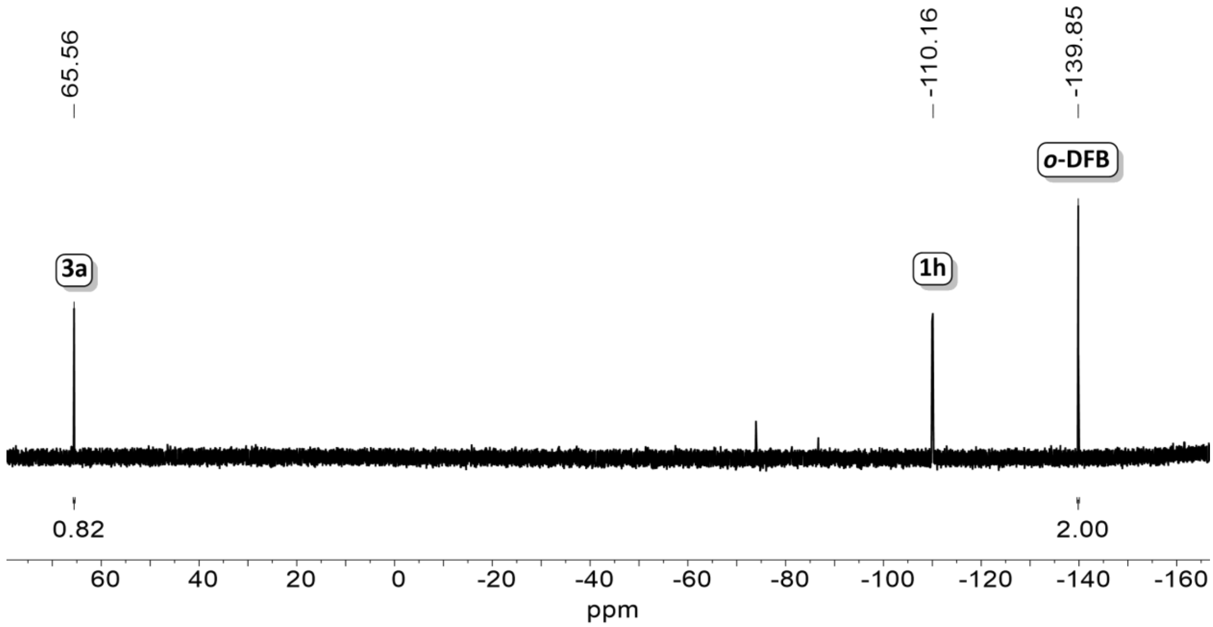

Supplementary Figure 28: ^{19}F NMR spectrum of fluorination of TsCl after 120 minutes activation of **1h** at 25 °C and 60 minutes fluorination at 100 °C (377 MHz, THF-h_8, 25 °C). 0.042 mmol **1h**, 0.025 mmol KO^{t}Bu and 0.021 mmol TsCl led to the formation of 0.017 mmol TsF.

5.27 Fluorination using **1i:** HFC-143a (Procedure D)

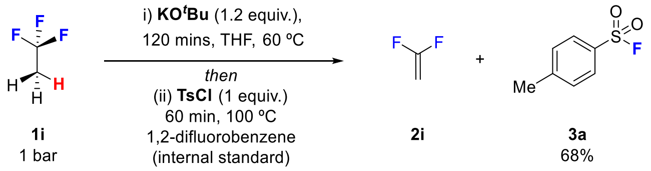

Supplementary Scheme 35: Transfer fluorination using **1i** as a donor.

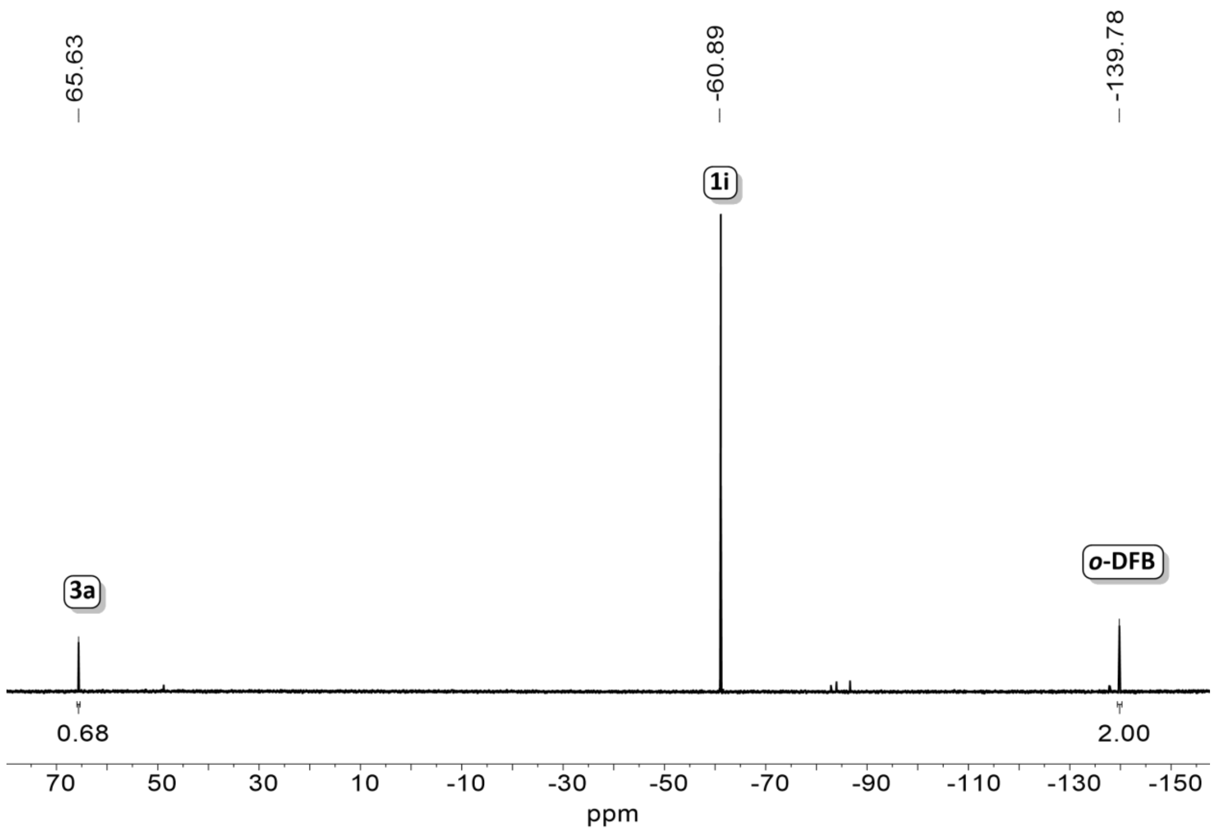

Supplementary Figure 29: ^{19}F NMR spectrum of fluorination of TsCl after 120 minutes activation of **1i** at 60 °C and 60 minutes fluorination at 100 °C (377 MHz, THF-h_8, 25 °C). 0.042 mmol **1i**, 0.025 mmol KO^{t}Bu and 0.021 mmol TsCl led to the formation of 0.014 mmol TsF.

5.28. Fluorination using **1j**: HFC-134a (Procedure D)

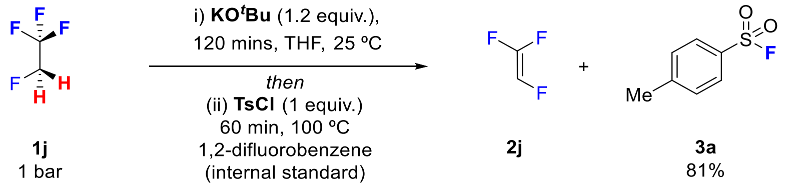

Supplementary Scheme 36: Transfer fluorination using **1j** as a donor.

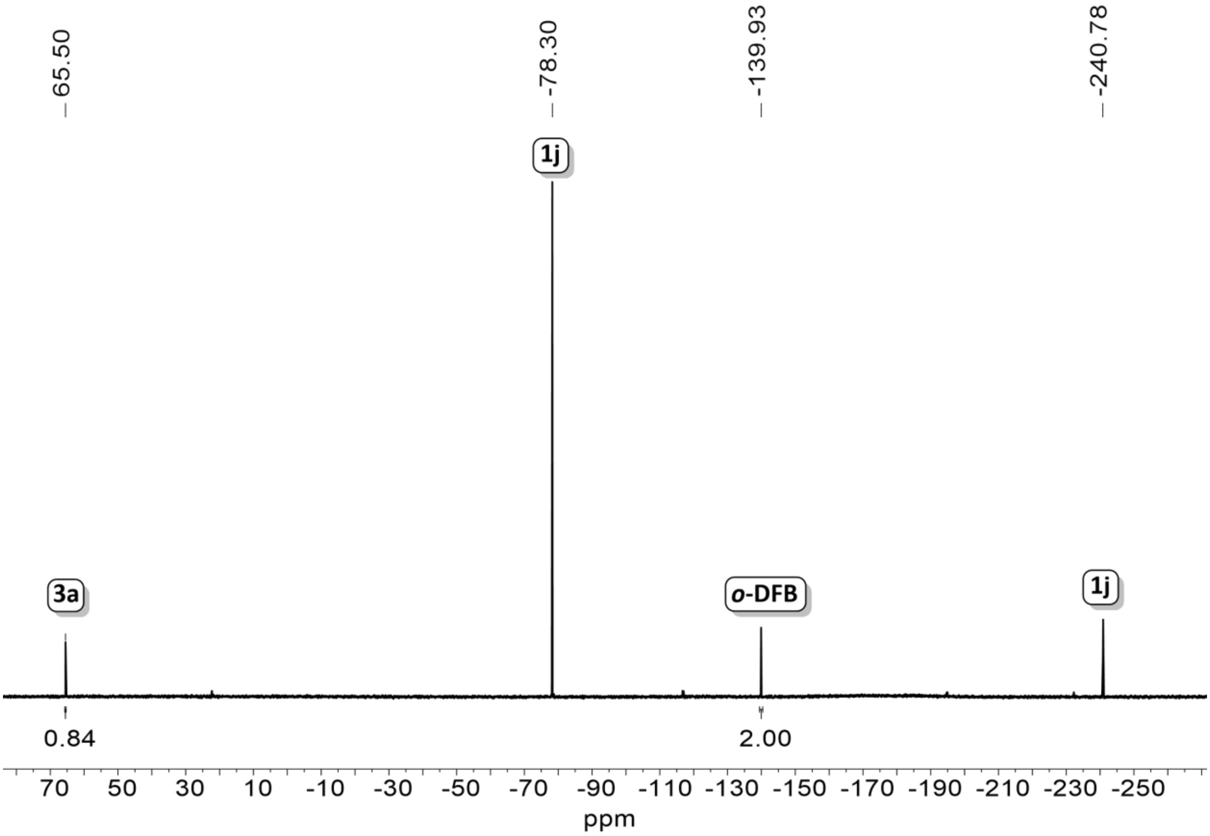

Supplementary Figure 30: ^{19}F NMR spectrum of fluorination of TsCl after 120 minutes activation of **1j** at 25 °C and 60 minutes fluorination at 100 °C (377 MHz, THF-h_8, 25 °C). 0.042 mmol **1j**, 0.025 mmol KO^{t}Bu and 0.021 mmol TsCl led to the formation of 0.017 mmol TsF.

5.29 Fluorination using **1k**: HFC-125 (Procedure D)

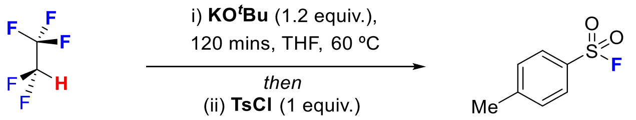

Supplementary Scheme 37: Transfer fluorination using **1k** as a donor.

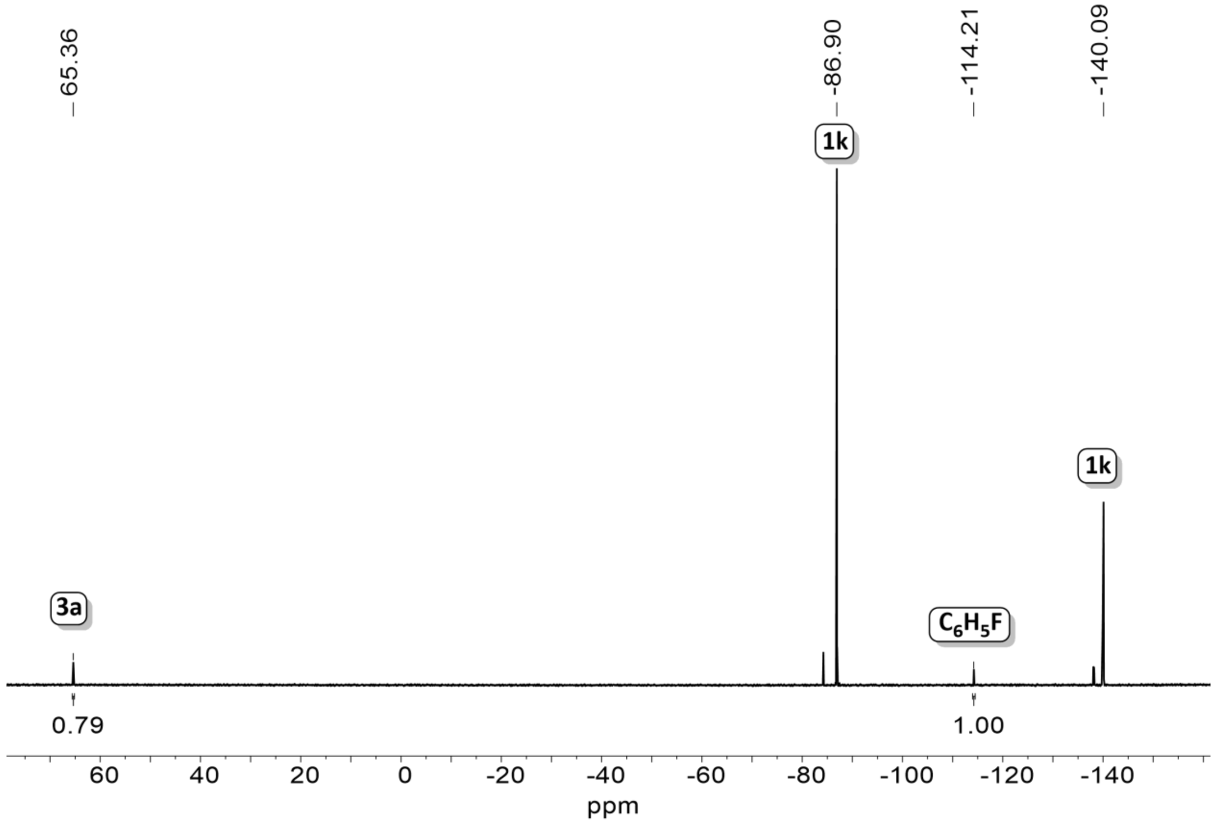

Supplementary Figure 31: ^{19}F NMR spectrum of fluorination of TsCl after 120 minutes activation of **1k** at 60 °C and 60 minutes fluorination at 100 °C (377 MHz, THF-h_8, 25 °C). 0.042 mmol **1k**, 0.025 mmol KO^{t}Bu and 0.021 mmol TsCl led to the formation of 0.017 mmol TsF. C_6H_5F was used as an internal standard due to the resonance of *o*-DFB overlapping with 1k.

5.30 Fluorination using **1l**: HFO-1234yf (Procedure D)

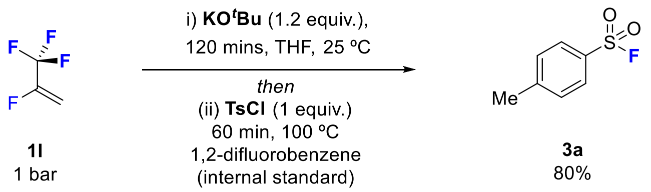

Supplementary Scheme 38: Transfer fluorination using **1l** as a donor.

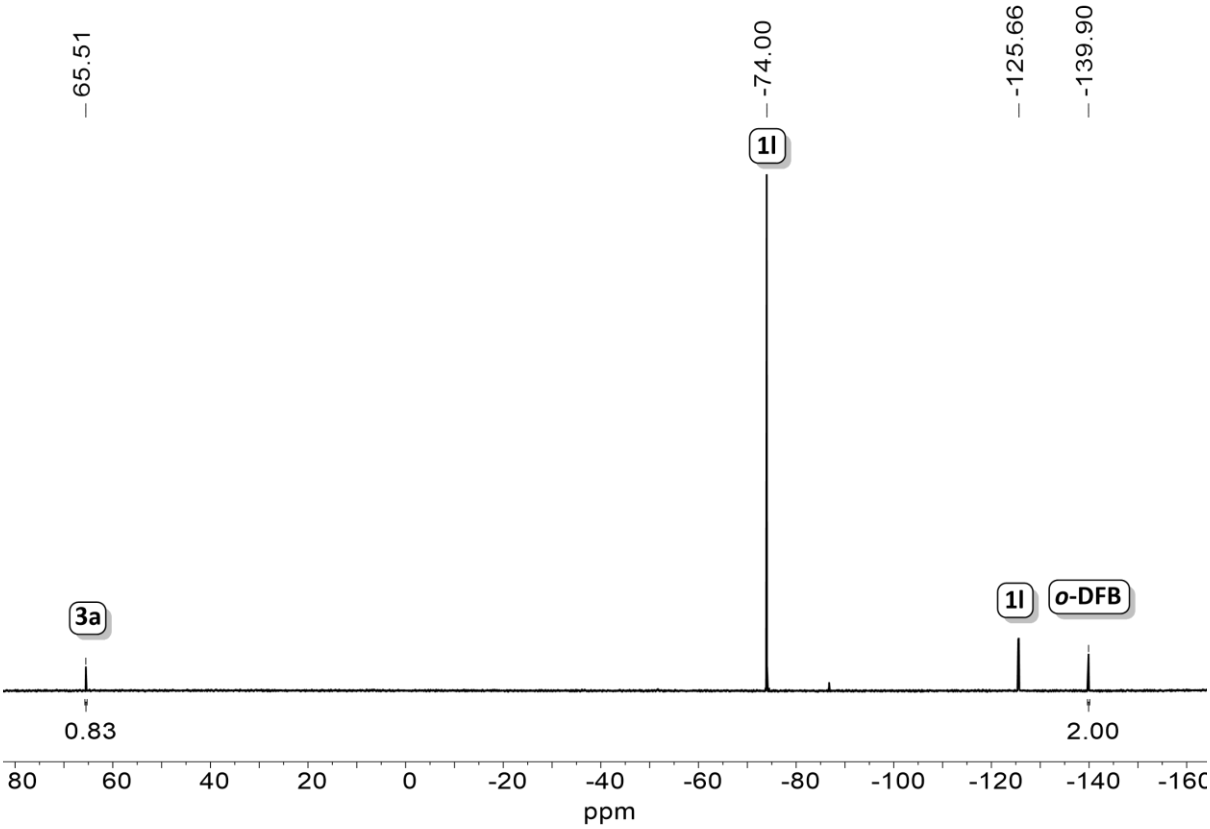

Supplementary Figure 32: ^{19}F NMR spectrum of fluorination of TsCl after 120 minutes activation of **1l** at 25 °C and 60 minutes fluorination at 100 °C (377 MHz, THF-h_8, 25 °C). 0.042 mmol **1l**, 0.025 mmol KO^{t}Bu and 0.021 mmol TsCl led to the formation of 0.017 mmol TsF.

5.31 Fluorination using **1m**: Sevoflurane (Procedure C)

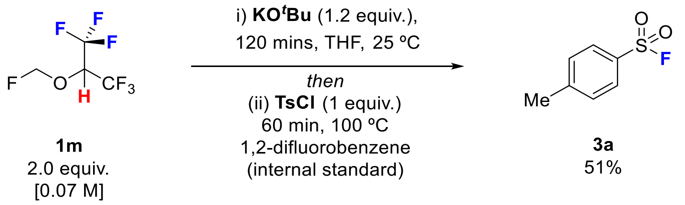

Supplementary Scheme 39: Transfer fluorination using **1m** as a donor.

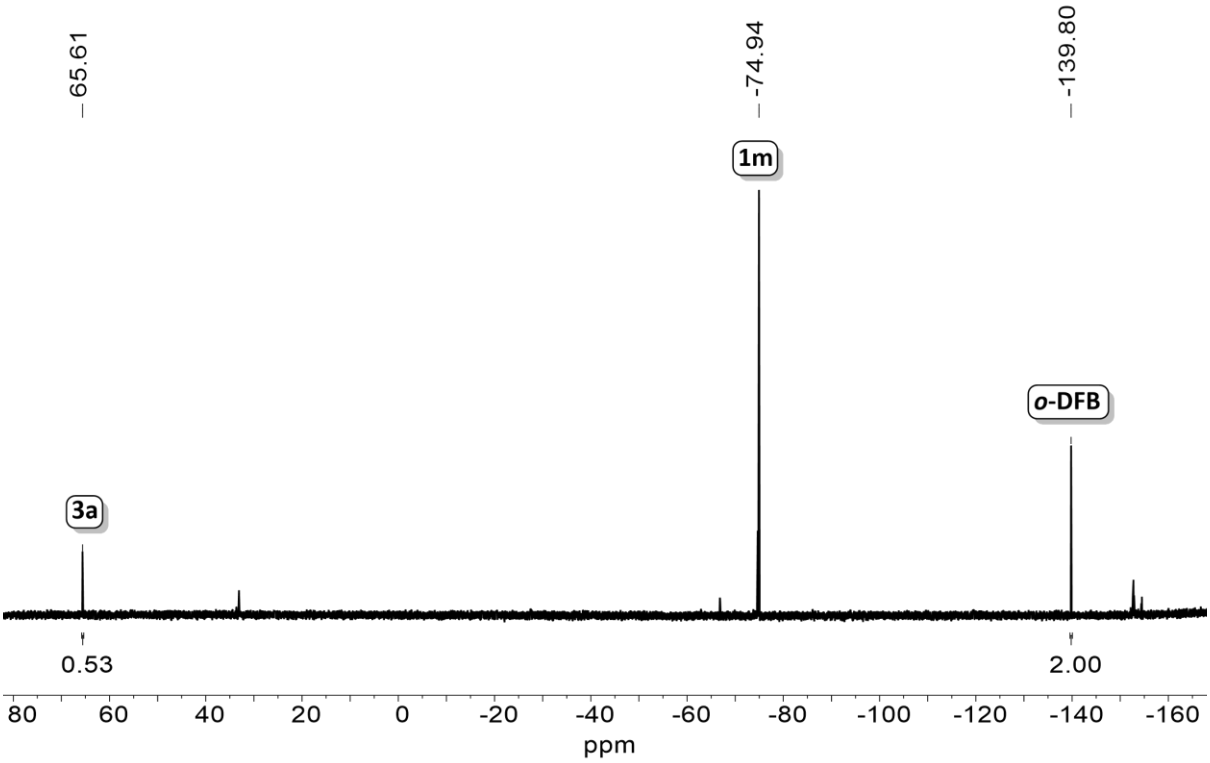

Supplementary Figure 33: ^{19}F NMR spectrum of fluorination of TsCl after 120 minutes activation of **1m** at 25 °C and 60 minutes fluorination at 100 °C (377 MHz, THF-h_8, 25 °C). 0.042 mmol **1m**, 0.025 mmol KO^{t}Bu and 0.021 mmol TsCl led to the formation of 0.011 mmol TsF.

5.32 Fluorination using **1n**: Isoflurane (Procedure C)

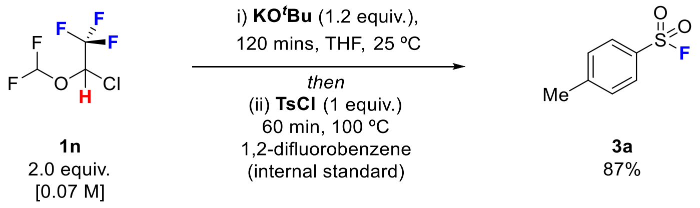

Supplementary Scheme 40: Transfer fluorination using **1n** as a donor.

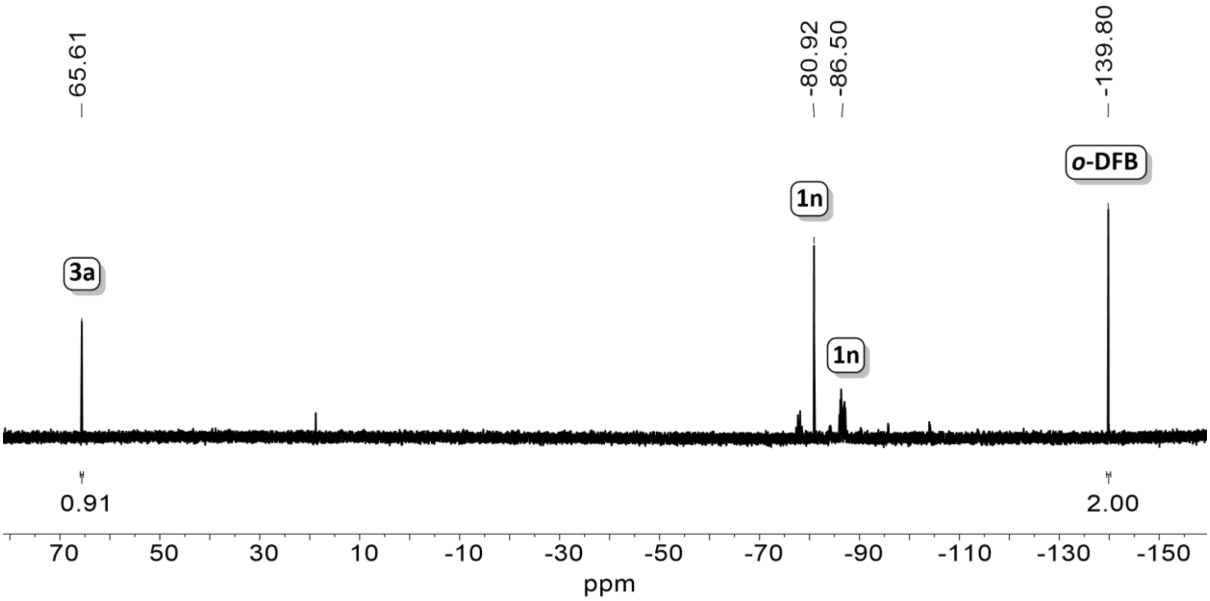

Supplementary Figure 34: ^{19}F NMR spectrum of fluorination of TsCl after 120 minutes activation of **1n** at 25 °C and 60 minutes fluorination at 100 °C (377 MHz, THF-h_8, 25 °C). 0.042 mmol **1n**, 0.025 mmol KO^{t}Bu and 0.021 mmol TsCl led to the formation of 0.018 mmol TsF.

5.33 Fluorination using **1o**: Enflurane (Procedure C)

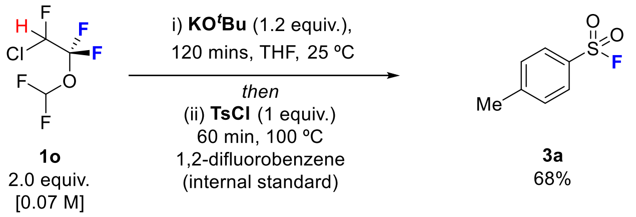

Supplementary Scheme 41: Transfer fluorination using **1o** as a donor.

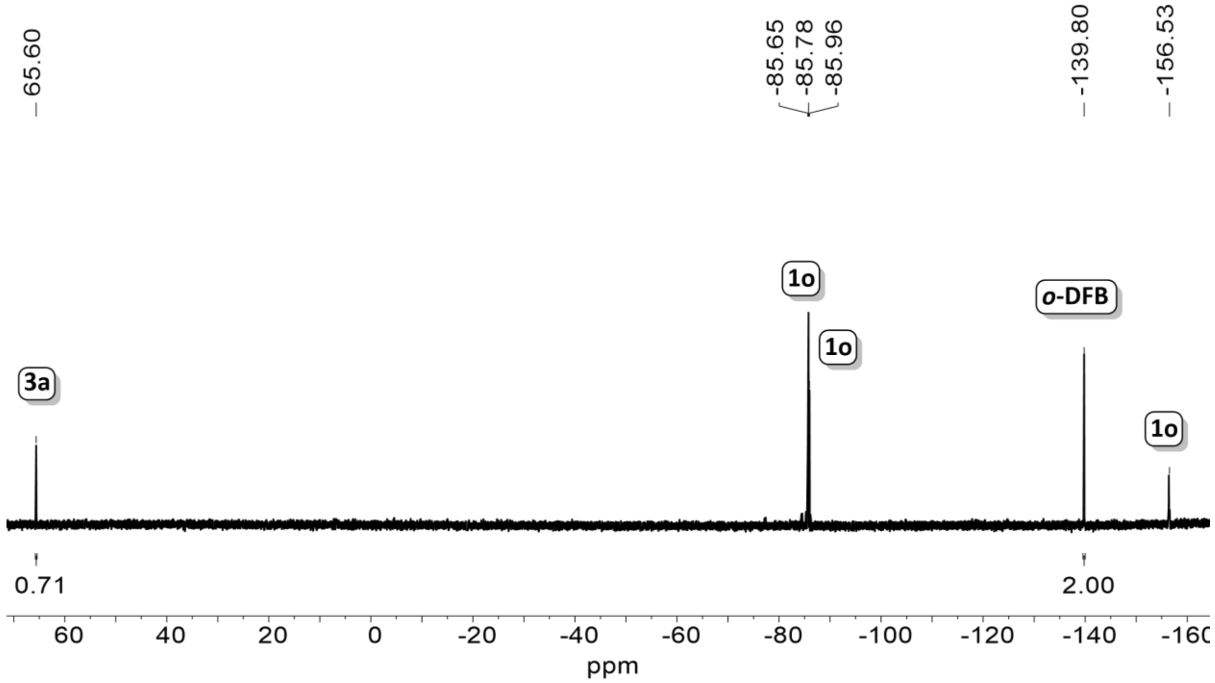

Supplementary Figure 35: ^{19}F NMR spectrum of fluorination of TsCl after 120 minutes activation of **1o** at 25 °C and 60 minutes fluorination at 100 °C (377 MHz, THF-h_8, 25 °C). 0.042 mmol **1o**, 0.025 mmol KO^{t}Bu and 0.021 mmol TsCl led to the formation of 0.014 mmol TsF.

5.354 Fluorination using **1p**: Hexafluoroisopropyl methyl ether (Procedure C)

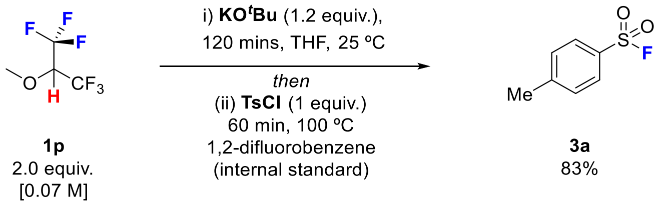

Supplementary Scheme 42: Transfer fluorination using **1p** as a donor.

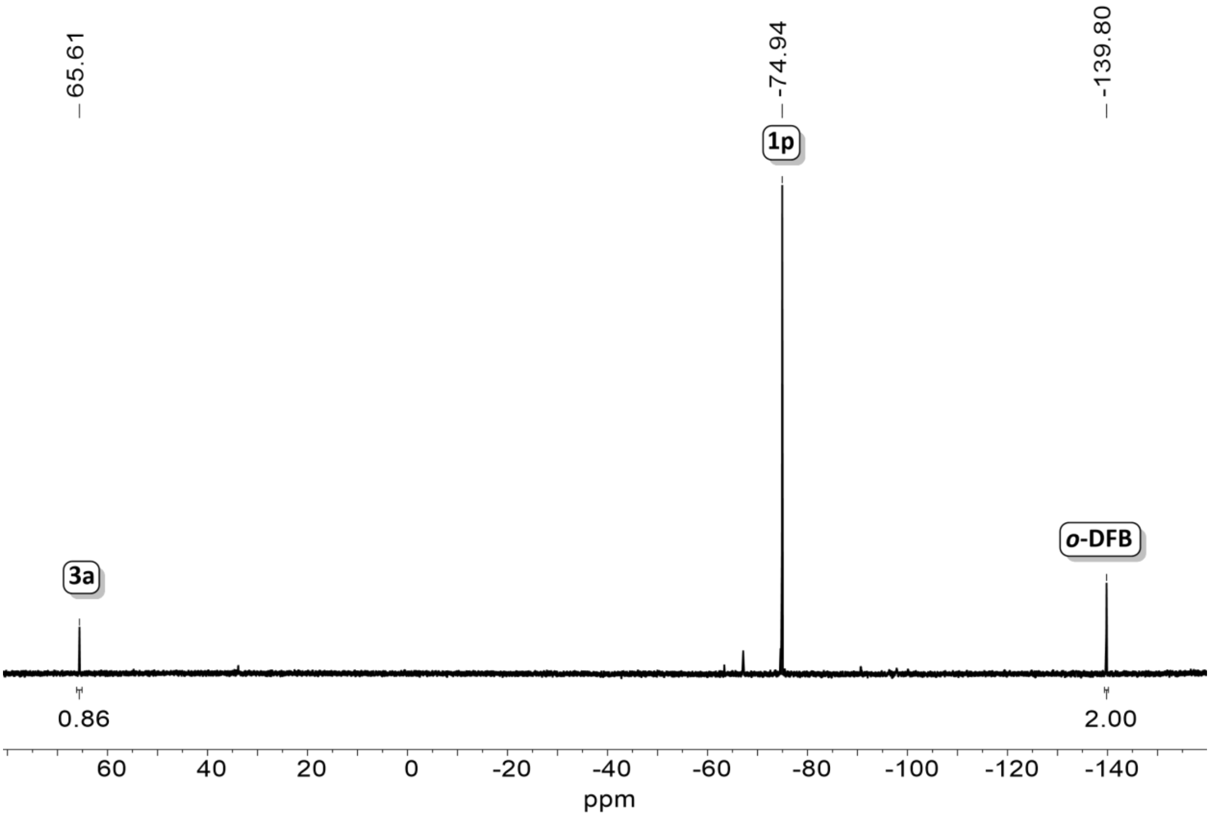

Supplementary Figure 36: ^{19}F NMR spectrum of fluorination of TsCl after 120 minutes activation of **1p** at 25 °C and 60 minutes fluorination at 100 °C (377 MHz, THF-h_8, 25 °C). 0.042 mmol **1p**, 0.025 mmol KO^{t}Bu and 0.021 mmol TsCl led to the formation of 0.017 mmol TsF.

5.35 Fluorination using **1q**: Bis(2,2,2-trifluoroethyl) ether (Procedure C)

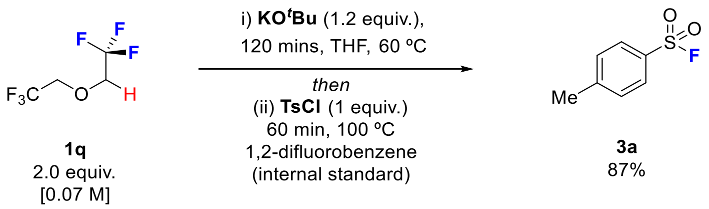

Supplementary Scheme 43: Transfer fluorination using **1q** as a donor.

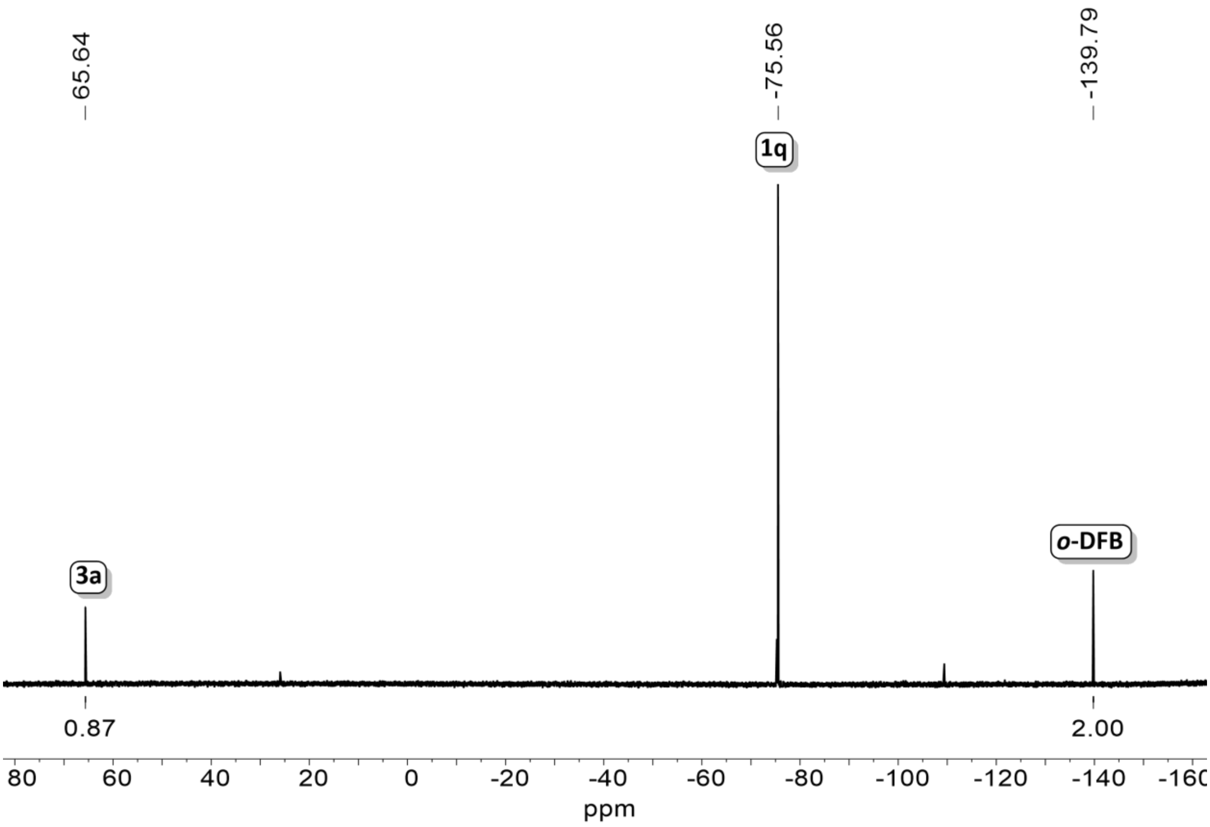

Supplementary Figure 37: ^{19}F NMR spectrum of fluorination of TsCl after 120 minutes activation of **1q** at 60 °C and 60 minutes fluorination at 100 °C (377 MHz, THF-h_8, 25 °C). 0.042 mmol **1q**, 0.025 mmol KO^{t}Bu and 0.021 mmol TsCl led to the formation of 0.018 mmol TsF.

5.36 Fluorination using **1r**: TFTFE (Procedure C)

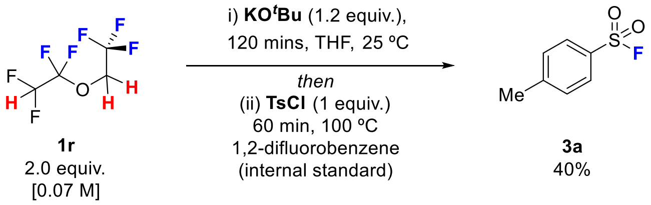

Supplementary Scheme 44: Transfer fluorination using **1r** as a donor.

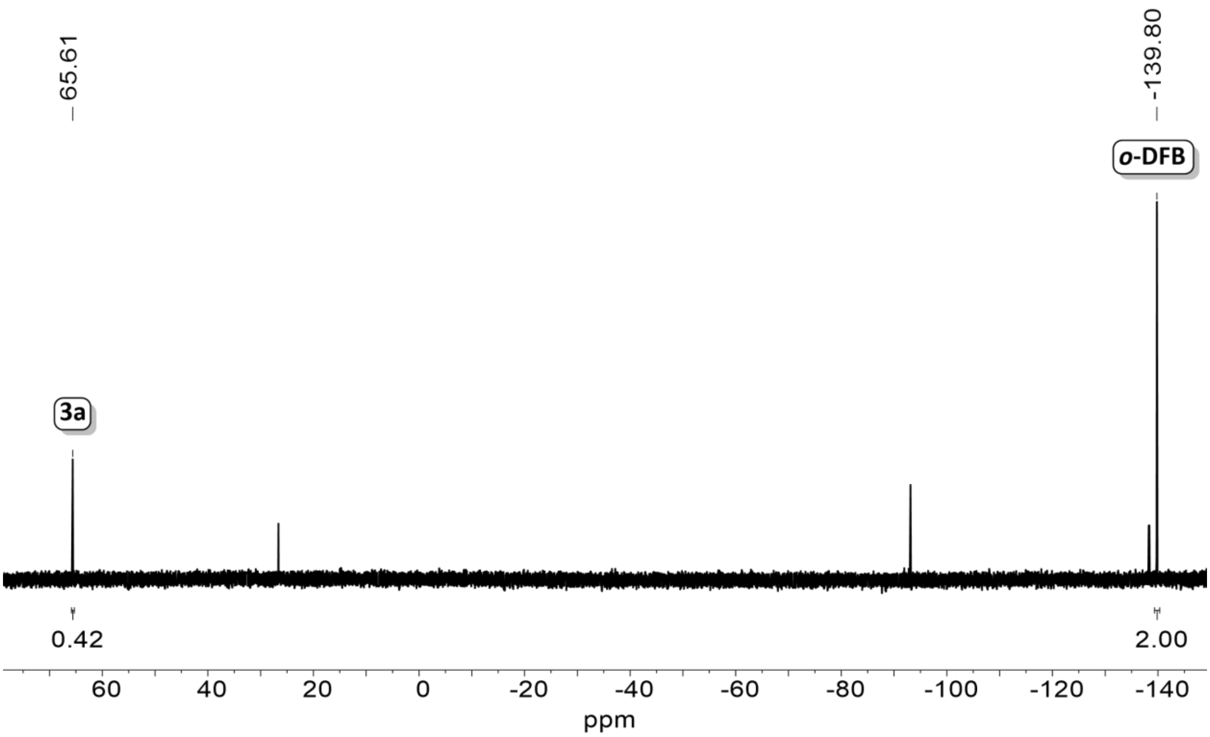

Supplementary Figure 38: ^{19}F NMR spectrum of fluorination of TsCl after 120 minutes activation of **1r** at 25 °C and 60 minutes fluorination at 100 °C (377 MHz, THF-h_8, 25 °C). 0.042 mmol **1r**, 0.025 mmol KO^{t}Bu and 0.021 mmol TsCl led to the formation of 0.008 mmol TsF.

5.37 Fluorination using **1s**: Volatile PFAS (Procedure C)

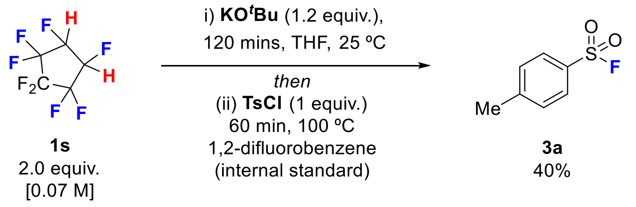

Supplementary Scheme 45: Transfer fluorination using **1s** as a donor.

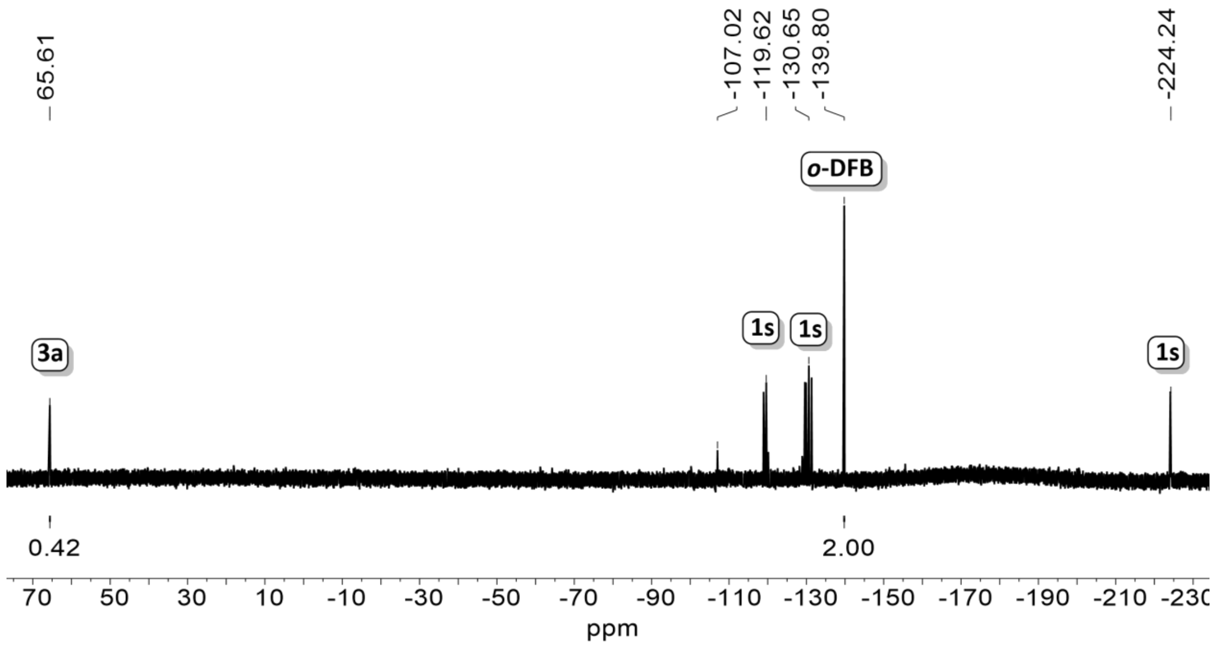

Supplementary Figure 39: ^{19}F NMR spectrum of fluorination of TsCl after 120 minutes activation of **1s** at 25 °C and 60 minutes fluorination at 100 °C (377 MHz, THF-h_8, 25 °C). 0.042 mmol **1s**, 0.025 mmol KO^{t}Bu and 0.021 mmol TsCl led to the formation of 0.008 mmol TsF.

5.38 Fluorination using **1t**: Volatile PFAS (Procedure C)

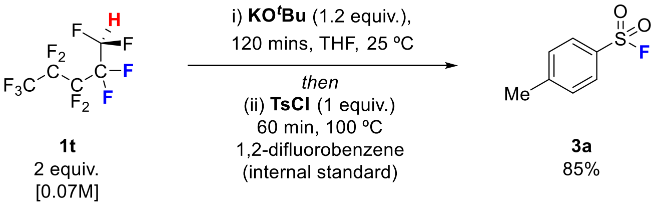

Supplementary Scheme 46: Transfer fluorination using **1t** as a donor.

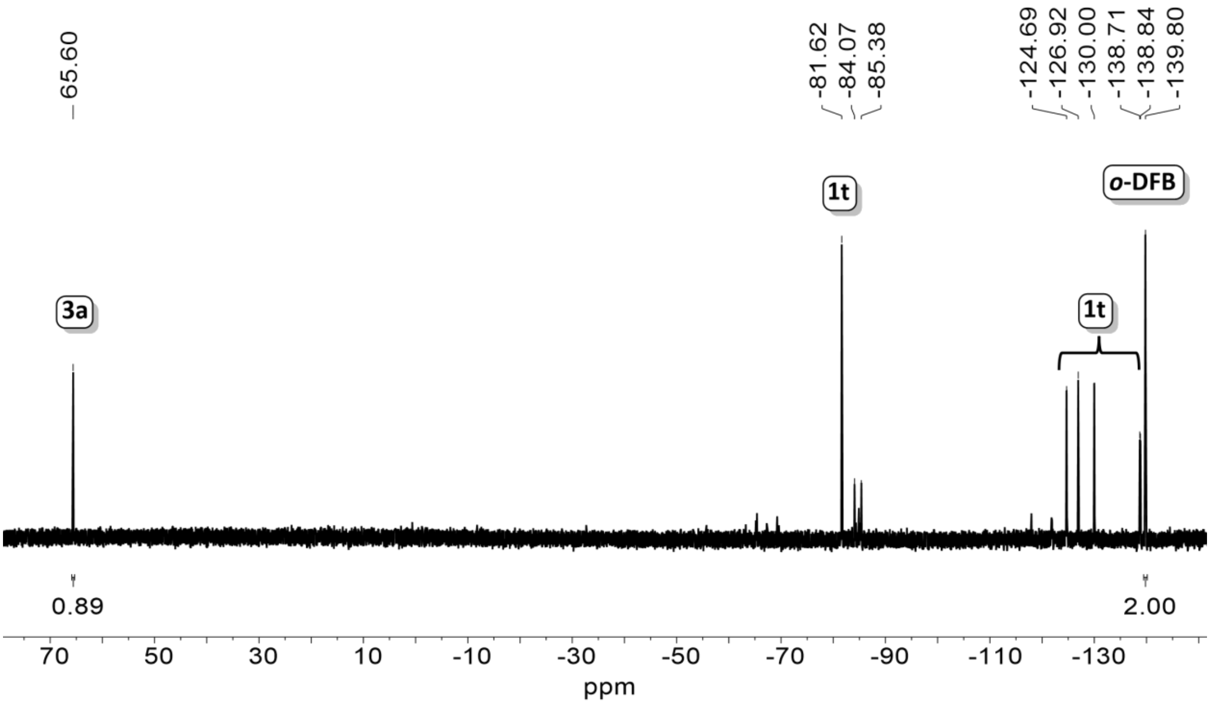

Supplementary Figure 40: ^{19}F NMR spectrum of fluorination of TsCl after 120 minutes activation of **1t** at 25 °C and 60 minutes fluorination at 100 °C (377 MHz, THF-h_8, 25 °C). 0.042 mmol **1t**, 0.025 mmol KO^{t}Bu and 0.021 mmol TsCl led to the formation of 0.018 mmol TsF.

5.39 Donor limitation of the transfer fluorination protocol

In contrast to **1a–1v**, few commercially available potential fluorine atom donors were identified as limitations. These compounds either performed poorly or showed no propensity for transfer fluorination of TsCl under the conditions investigated. These compounds and the corresponding outcomes are listed below (Supplementary Figure 41).

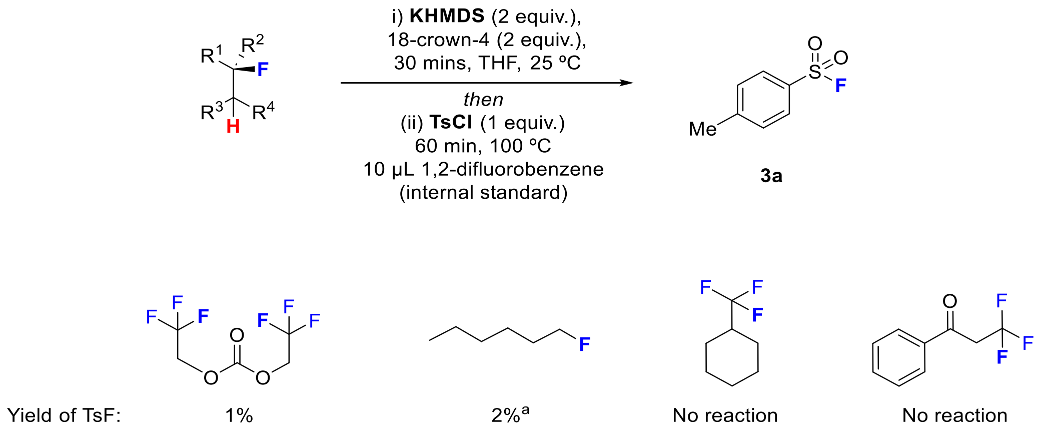

Supplementary Figure 41: Less successful candidates for the donor scope performed using protocol Procedure A.
[a] Benzyl potassium was used as the base in this case.

6. KF Quantification

The quantification of KF in the defluorination reactions was performed using ^{19}F NMR spectroscopy using sodium triflate as a standard for integration comparison. Two donors were chosen as model substrates to represent the defluorination reactions: **1a** and **1j**. The reactions performed are detailed in the table below.

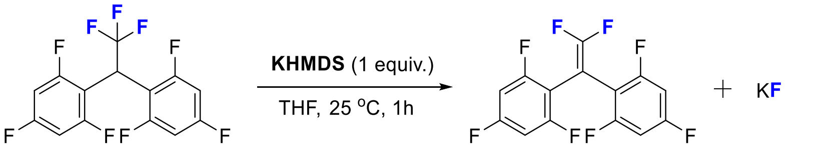

Supplementary Scheme 47: Determination of the KF yield derived from **1a**, condition in scheme are below in entry 1 of Supplementary Table 5.

Procedure of KF quantification for **1a**:

In a dinitrogen containing glovebox, KHMDS (5.8–12.3 mg, 0.029–0.062 mmol) was dissolved in 0.5 ml THF and transferred to a J Young NMR tube. **1a** (10.2 mg, 0.030 mmol) in 0.2 ml THF was added to the J Young tube. The tube was inverted several times and the reaction mixture allowed to react for 1 hour. The reaction mixture was concentrated *in vacuo* inside the J Young tube. Outside of the glovebox, D_2O (1 ml) and sodium triflate (1 M solution in D_2O, 9.88 µl) was added. A yield of 177% KF (with respect to **1a** as limiting reagent) was calculated. A control experiment in which **2a** was subjected to the reaction conditions above confirmed that the excess KF derives from further defluorination of the product.

Procedure of KF quantification for **1j**:

In a dinitrogen containing glovebox, KHMDS (9.9–36.1 mg, 0.050–0.181 mmol) was dissolved in 0.5 ml THF and transferred to a J Young NMR tube. The reaction mixture was degassed *via* freeze-pump-thaw technique before **1j** (1.4 bar, 2.3 ml, 0.130 mmol) was added. The tube was inverted several times and the reaction mixture allowed to react for 1 hour. The reaction mixture was concentrated *in vacuo* inside the J Young tube. Outside of the glovebox, D_2O (1 ml) and sodium triflate (1 M solution in D_2O, 16.5 µl) was added. A yield of 93% KF (with respect to KHMDS as limiting reagent) was obtained using ^{19}F NMR spectroscopy.

Supplementary Table 5: KF quantification results from the defluorination of **1a** and **1j**.

Entry	Donor	Donor (mmol)	Base	Base (mmol)	Mol ratio Base:donor	% KF
1	**1a**	0.029	KHMDS	0.029	1.0	97
2	**1a**	0.030	KHMDS	0.062	2.1	177
3	**2a**	0.029	KHMDS	0.062	2.1	153
4	**1j**	0.130	KOtBu	0.053	0.4	70
5	**1j**	0.130	KHMDS	0.050	0.4	93
6	**1j**	0.087	KHMDS	0.181	2.1	123

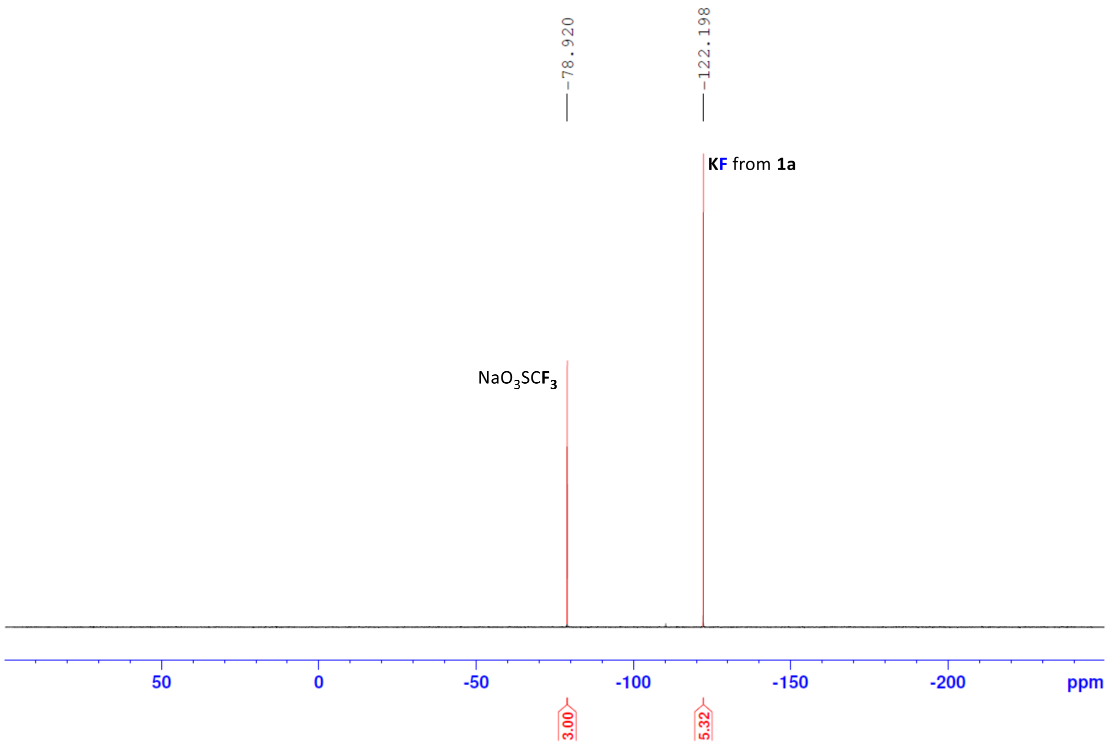

Supplementary Figure 42: Representative ^{19}F NMR spectrum for the KF quantification method, containing sodium triflate (9.88 µmol, 9.88 µl, 1.0 M) in D_2O as internal standard. This example, entry 2, gives 177% yield of KF with respect to the limiting reagent **1a** (377 MHz, D_2O, 25 °C).

The KF generated in the reaction of **1a** and KHMDS was characterised initially using ^{19}F NMR spectroscopy by comparison to commercial KF (Acros Chemicals) in D_2O, see Supplementary Figure 43.

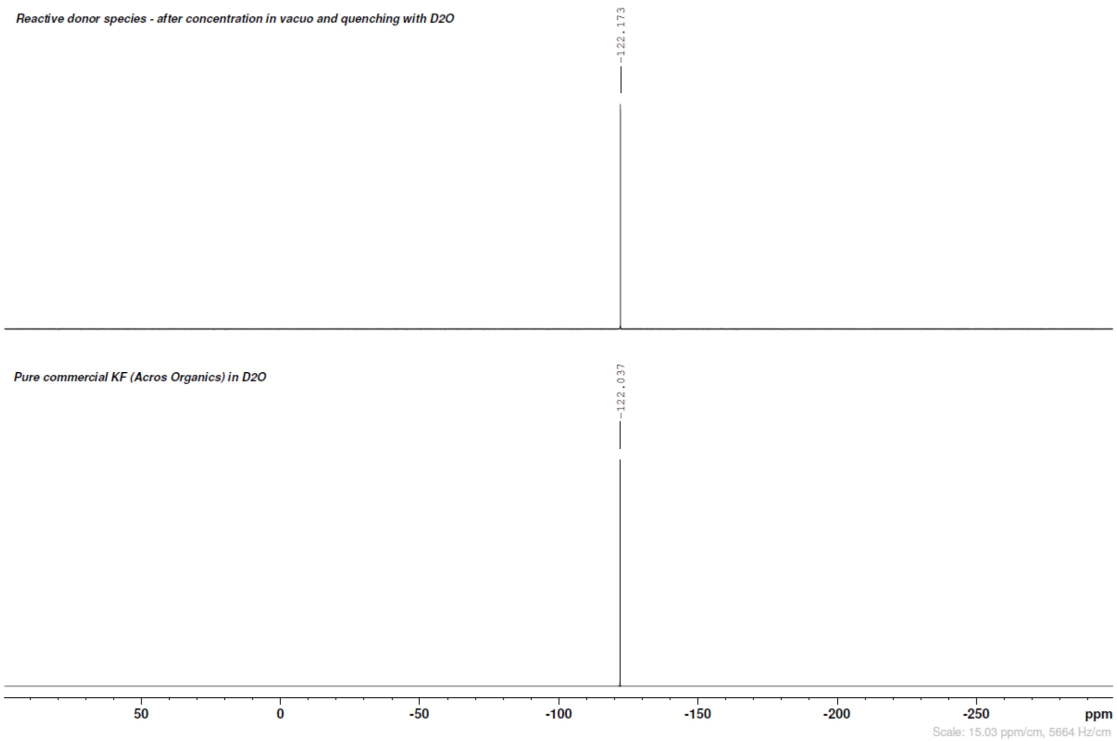

Supplementary Figure 43: ^{19}F NMR spectrum of the precipitate from **1a** treated with KHMDS in THF (top spectrum; 377 MHz, D_2O, 25 °C) and the ^{19}F NMR spectrum of commercial KF (bottom; purchased from Acros Chemicals) in comparison (377 MHz, D_2O, 25 °C).

7. Characterisation of the heterogenous fluorinating species

7.1. Photograph of NMR scale reaction

1a (17.0 mg, 0.049 mmol) was dissolved in 0.5 mL THF and transferred into a J. Young NMR tube, followed by the addition of a solution of KHMDS (15.0 mg, 0.075 mmol) in 0.4 mL THF. The tube was sealed, and the reaction allowed to progress for 90 minutes at 25 °C, while inverting the sample each 15 minutes. At the end of the reaction the tube was inverted and a photograph taken (Supplementary Figure 44).

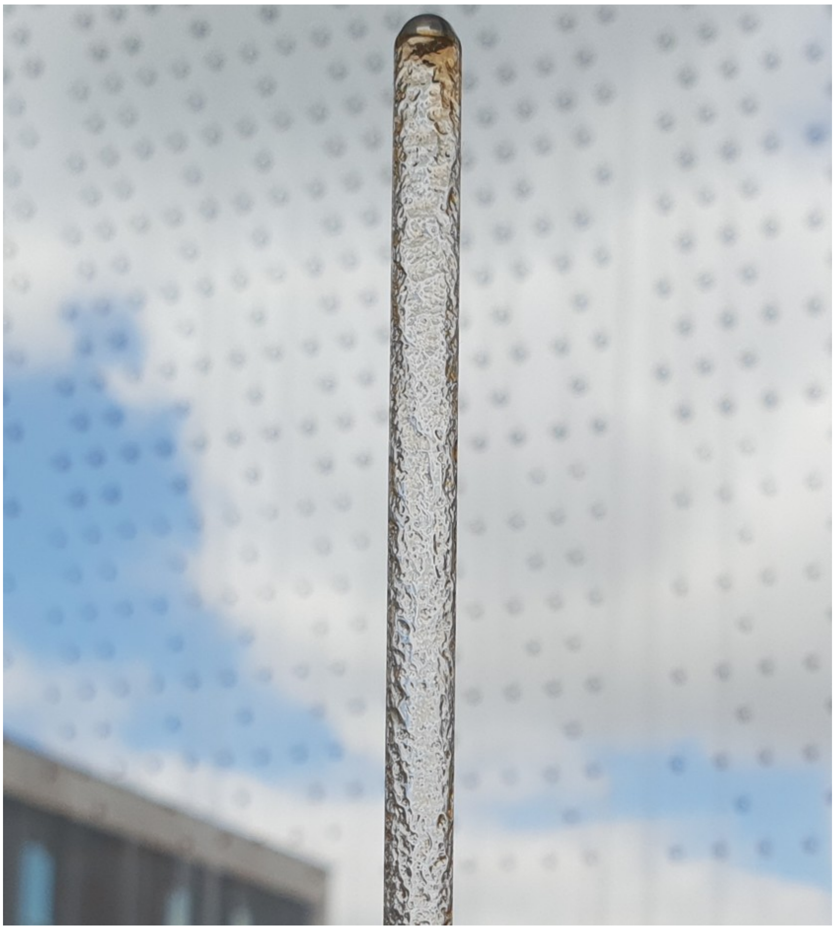

Supplementary Figure 44: Photograph of an inverted NMR sample containing the *in situ* fluorinating agent.

7.2. Isolation of KF

Method A: In a dinitrogen containing glovebox, KHMDS (343 mg, 1.72 mmol) was dissolved in 35 ml THF and transferred to a 70 ml ampoule. The reaction mixture was degassed *via* freeze-pump-thaw technique before HFC-134a (1.4 bar, 35 ml, 1.97 mmol) was added, the vessel was shaken initially then the reaction mixture allowed to vigorously stir for 60 minutes at 25 °C. A colour change from colourless solution to a brown suspension was observed. The mixture was filtered through celite via cannulation, the solid obtained was washed with n-hexane (2x5 ml) and dried *in vacuo*. A brown solid was obtained (68.2 mg, 90% KF %w/w, 1.17 mmol, 68% isolated yield). Percentage by weight %w/w KF was determined using ^{19}F NMR spectroscopy using NaOTf as an internal standard in D_2O. **Anal:** Found: C, 5.80; H, 0.13; N, 0.52.

Method B: In a dinitrogen containing glovebox, KHMDS (601.1 mg, 3.01 mmol) was dissolved in 12 ml THF and transferred to an 80 ml ampoule. The reaction mixture was degassed *via* freeze-pump-thaw technique before HFC-134a (1.2 bar, 68 ml, 3.29 mmol) was added, the vessel was shaken initially then the reaction mixture allowed to vigorously stir for 2 hours at 25 °C. A colour change from colourless solution to a black suspension was observed. The mixture was centrifuged for 30 minutes at 940 x g, the solid obtained was washed with n-hexane (2x3 ml) and dried *in vacuo*. A black solid was obtained (213 mg, 50% KF %w/w, 1.83 mmol, 61% isolated yield). Percentage by weight %w/w KF was determined using ^{19}F NMR spectroscopy using NaOTf as an internal standard in D_2O. **Anal:** Found: C, 6.64; H, 0.68; N, 0.77.

7.3. Identification of KF species *via* X-ray powder diffraction

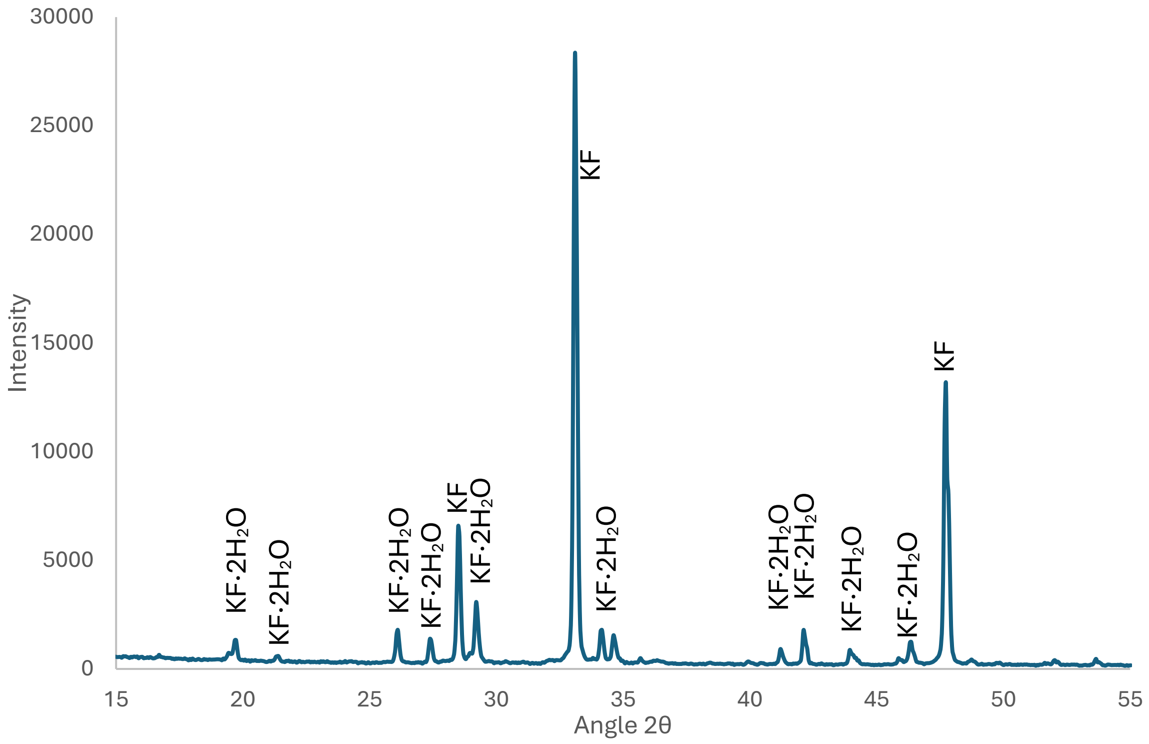

Supplementary Figure 45: Powder X-ray diffraction pattern of KF (Acros Organics) containing both crystalline anhydrous KF and KF·2H_2O. Commercial sample was stored under ambient conditions.

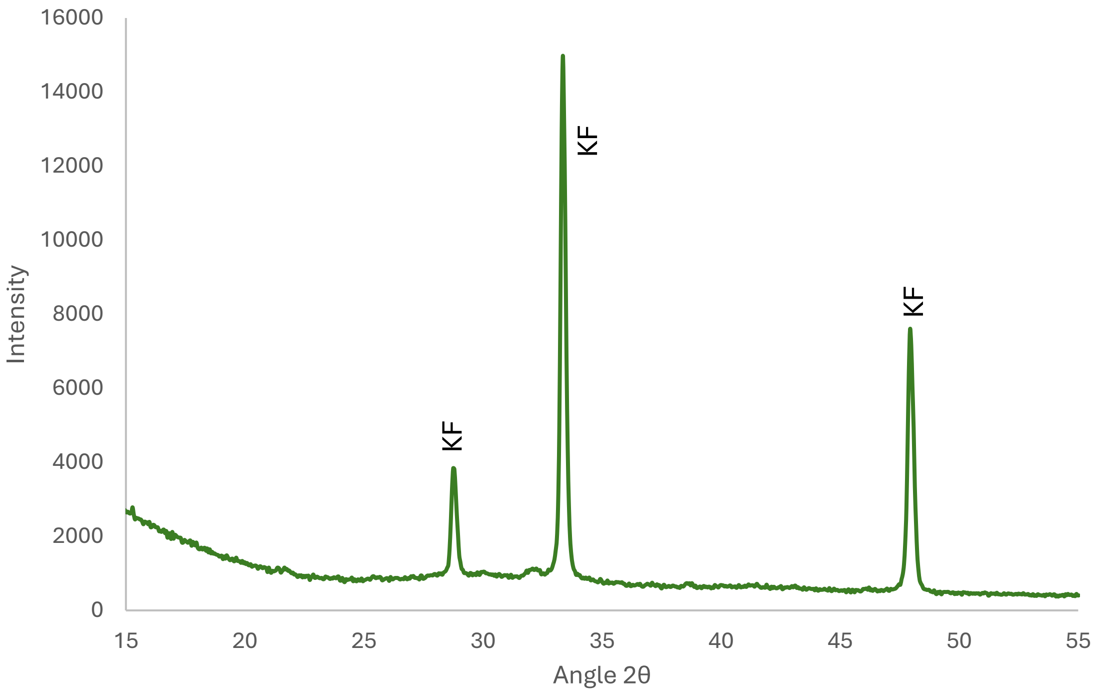

Supplementary Figure 46: Powder X-ray diffraction pattern of reactive KF species generated from KHMDS and HFC-134a and determined to have 90% KF by ^{19}F NMR spectroscopy. Sample

was isolated and stored inside a dinitrogen containing glovebox. Sample contains crystalline anhydrous KF but no $KF \cdot 2H_2O$.

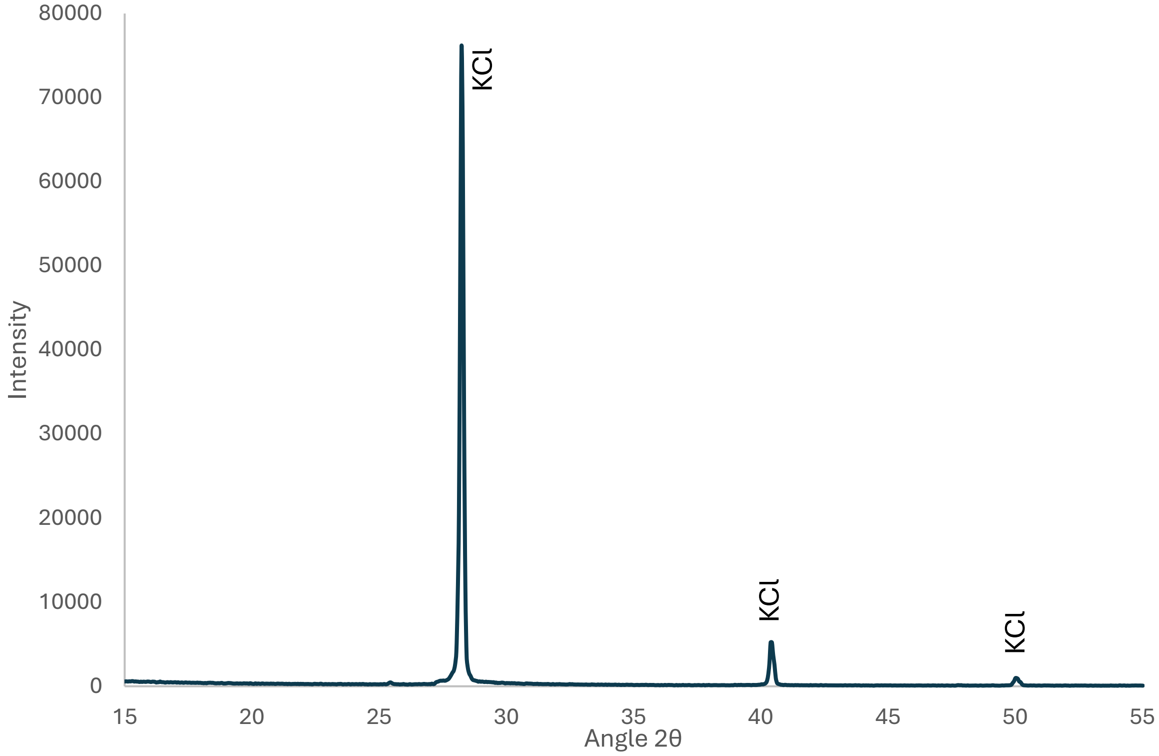

Supplementary Figure 47: Powder X-ray diffraction pattern of KCl (Sigma Aldrich). Commercial sample was stored under ambient conditions.

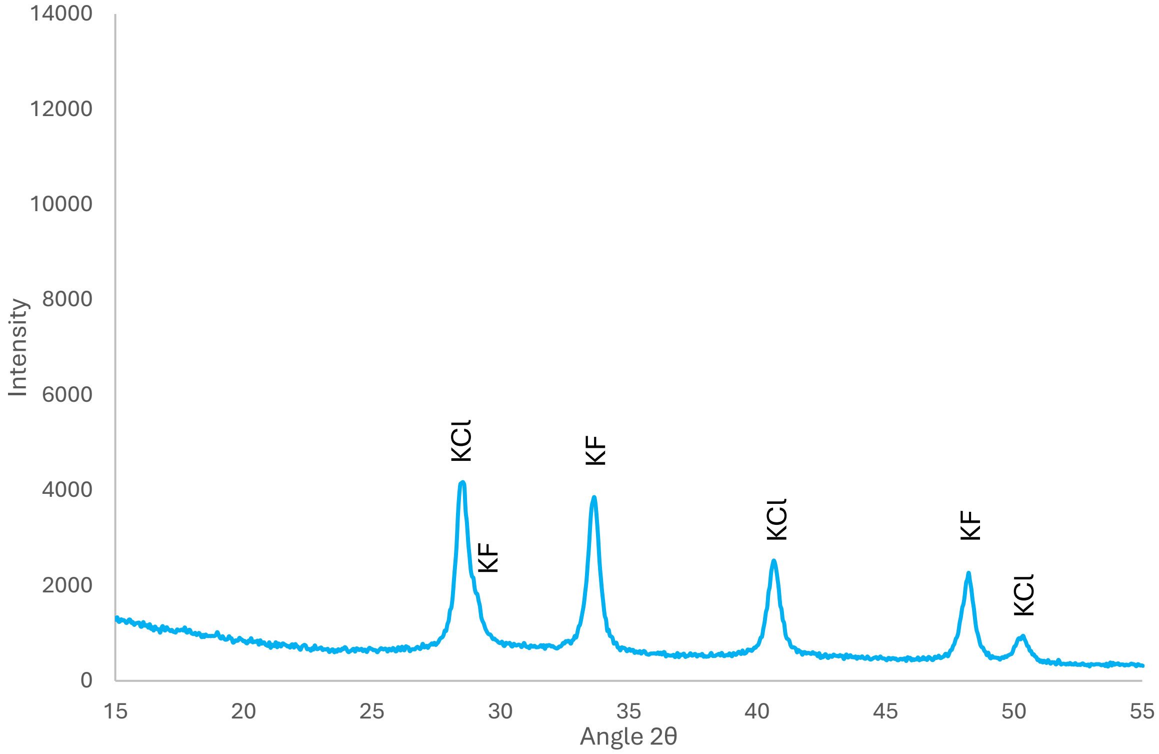

Supplementary Figure 48: Powder X-ray diffraction pattern of black precipitate isolated after the fluorination of Ph_2PCl. Isolation and storage of sample was performed in a dinitrogen containing glovebox. Sample contains unreacted anhydrous KF and generated KCl.

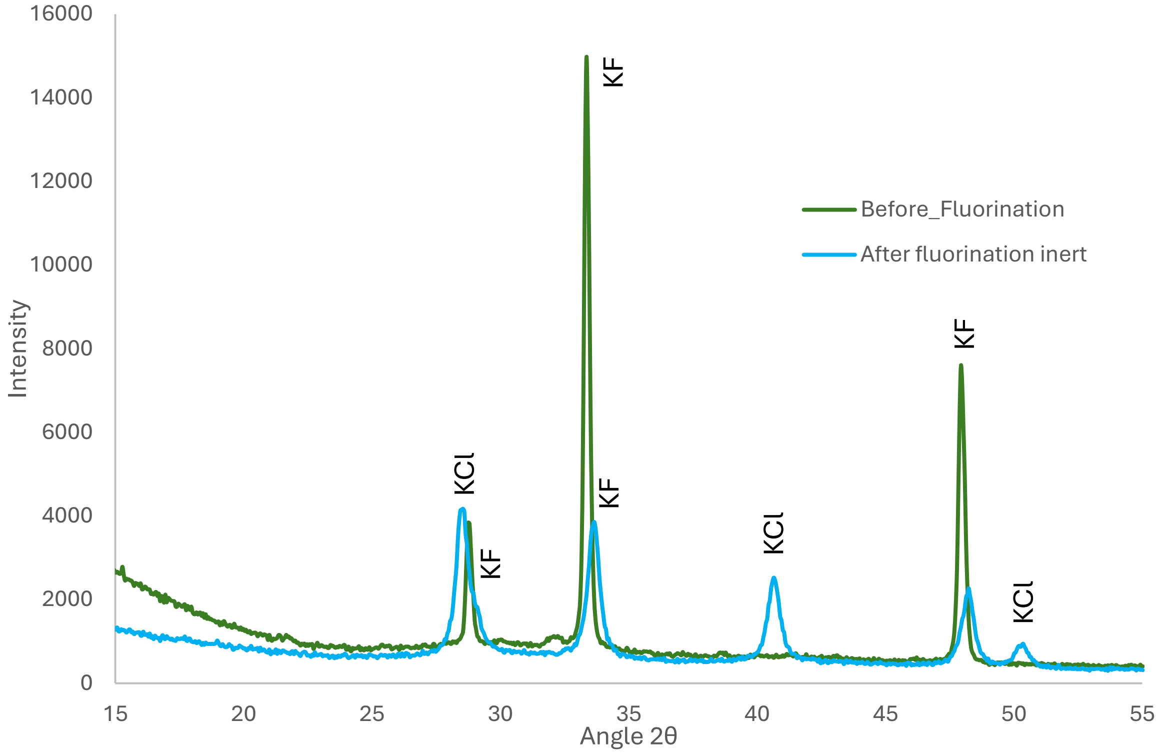

Supplementary Figure 49: Powder X-ray diffraction pattern overlay of the precipitates which were isolated before (green) and after (blue) fluorination. These samples were stored in a dinitrogen containing glovebox prior to analysis.

7.4. Characterisation of KF species *via* field emission scanning electron microscopy

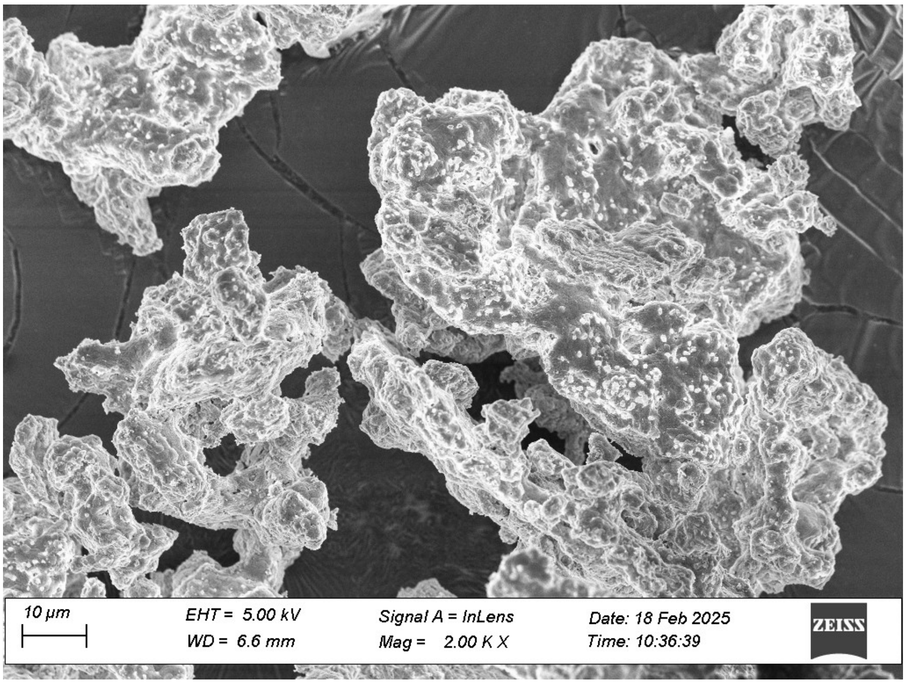

Supplementary Figure 50: FE-SEM image of commercial KF sample (Acros organics) at 2,000 X magnification. The grey regions within the particle clusters are indicative of the dihydrate present.

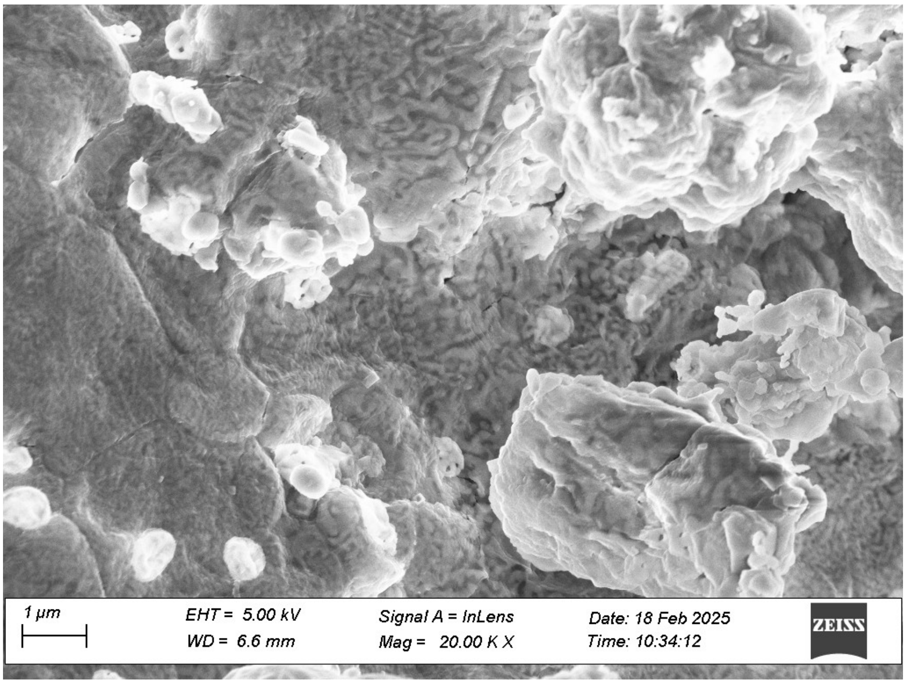

Supplementary Figure 51: FE-SEM image of commercial KF sample (Acros organics) at 20,000 X magnification.

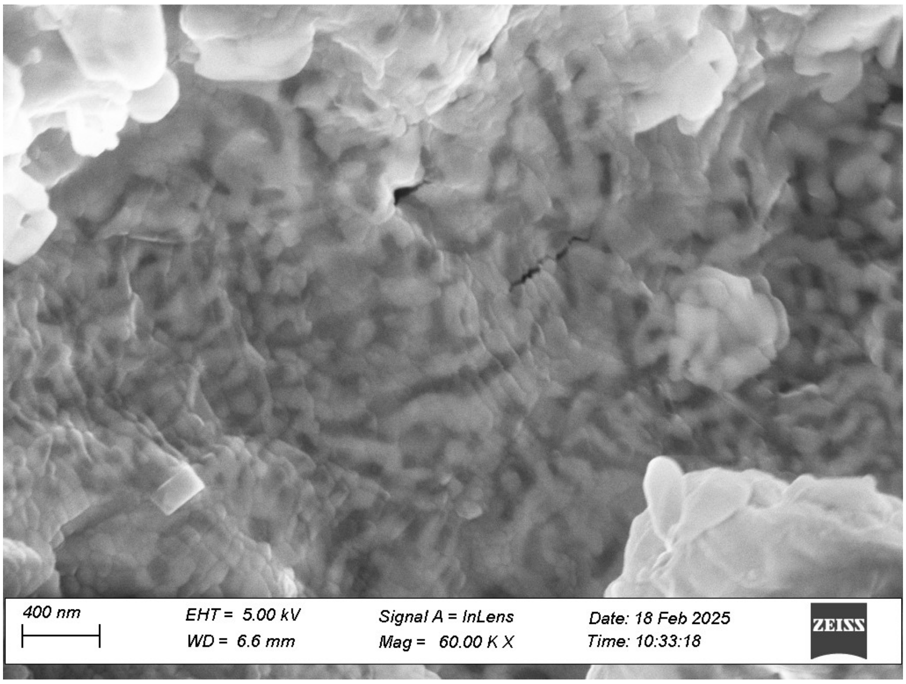

Supplementary Figure 52: FE-SEM image of commercial KF sample (Acros organics) at 60,000 X magnification.

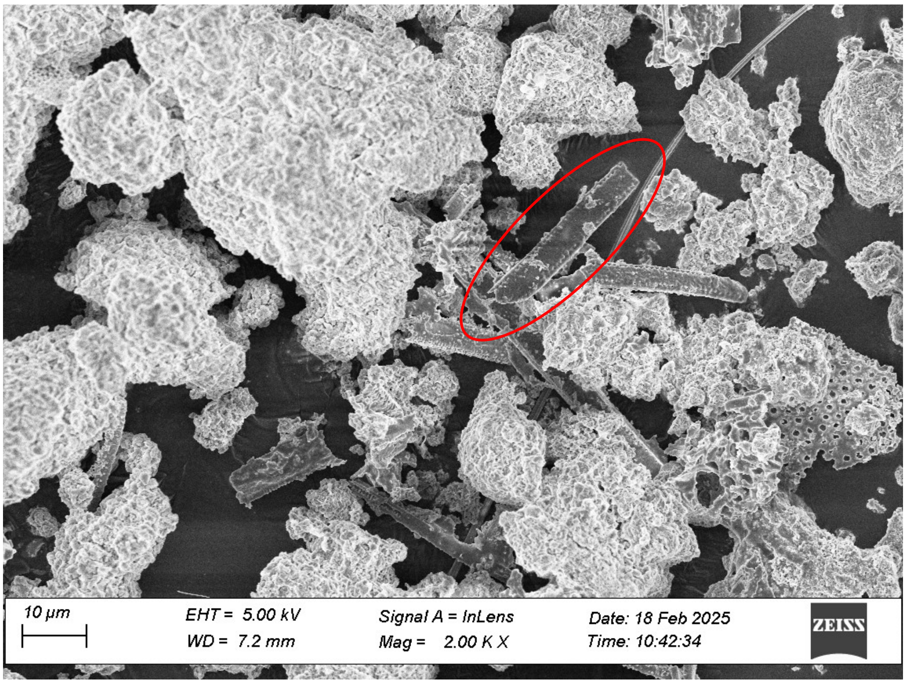

Supplementary Figure 53: FE-SEM image of reactive KF sample at 2,000 X magnification. Sample contains 90% KF by ^{19}F NMR spectroscopy. The reactive KF sample appears more granular with no grey regions within the clusters indicating no hydrate is present. The sample also contains a small amount of carbon-based structures pictured as the long flat structures (circled in red).

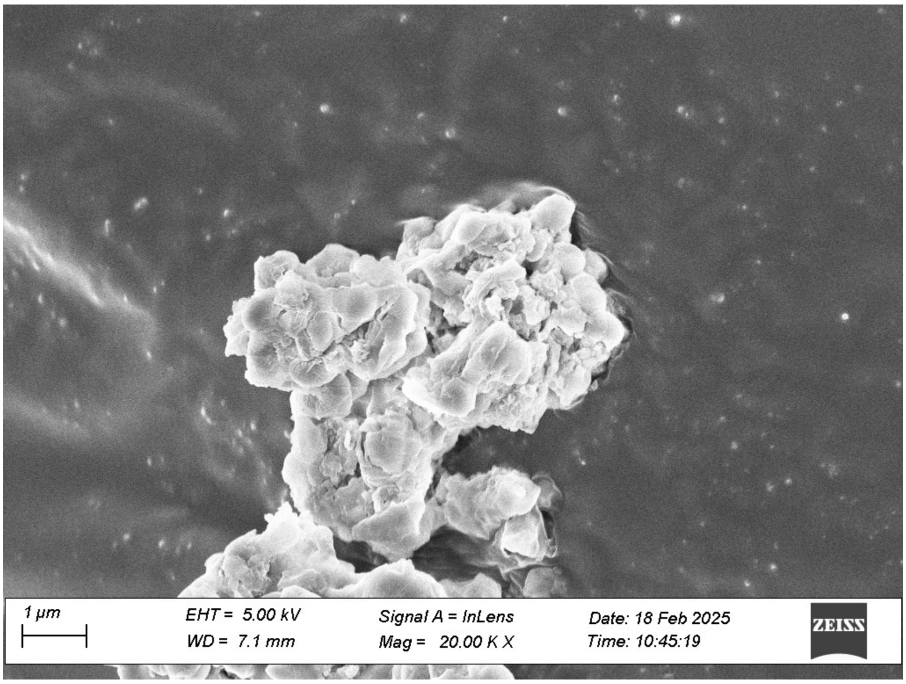

Supplementary Figure 54: FE-SEM image of reactive KF sample at 20,000 X magnification. Sample contains 90% KF by ^{19}F NMR spectroscopy.

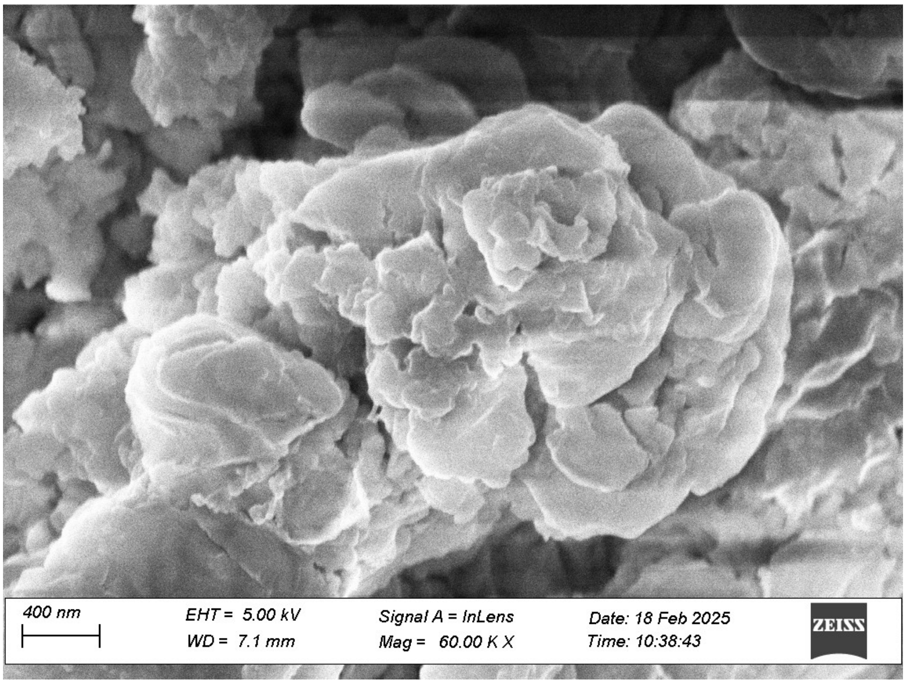

Supplementary Figure 55: FE-SEM image of reactive KF sample at 60,000 X magnification. Sample contains 90% KF by ^{19}F NMR spectroscopy.

7.5. Characterisation of KF species *via* MAS Solid State NMR spectroscopy

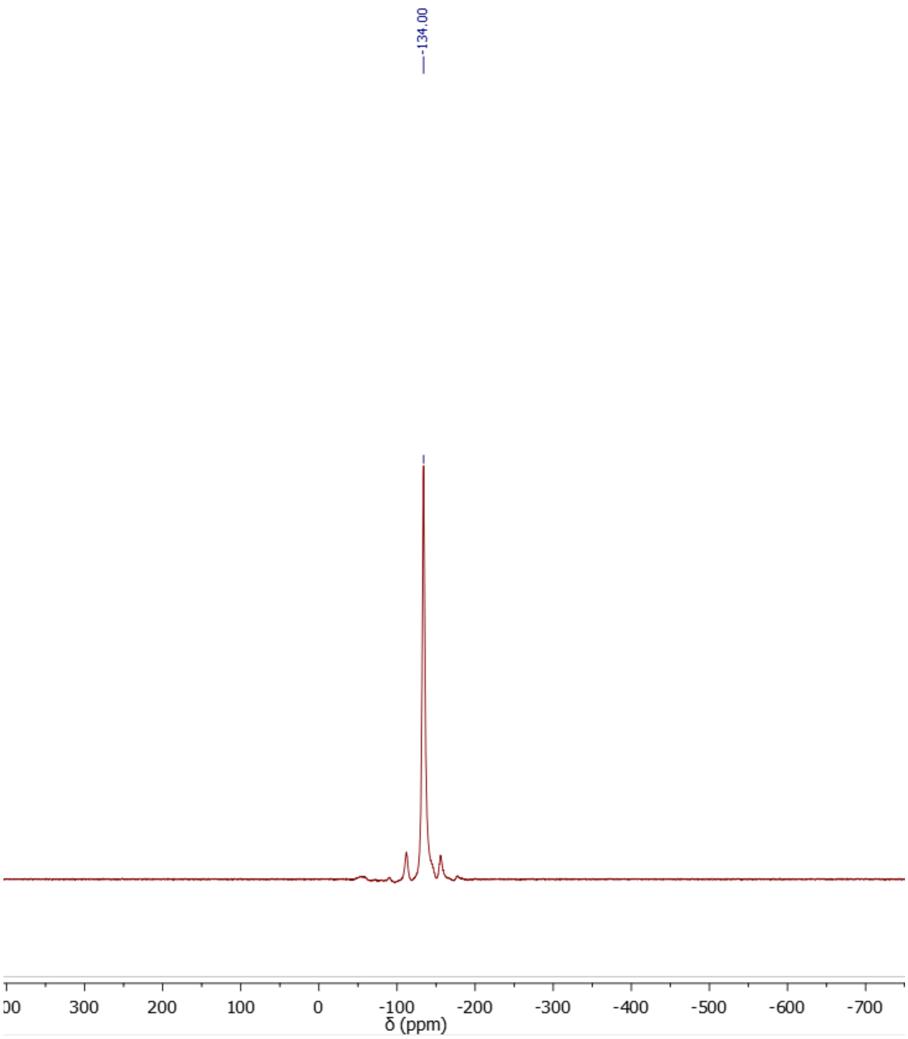

Supplementary Figure 56: MAS-SS ^{19}F NMR spectrum of isolated black KF solid from the defluorination of **1j** using a 4 mm rotor. The spectrum contains a single peak corresponding to KF with no other fluorine-containing species present. Additionally, no peaks were found in a $^{13}C/^{19}F$ correlation study.

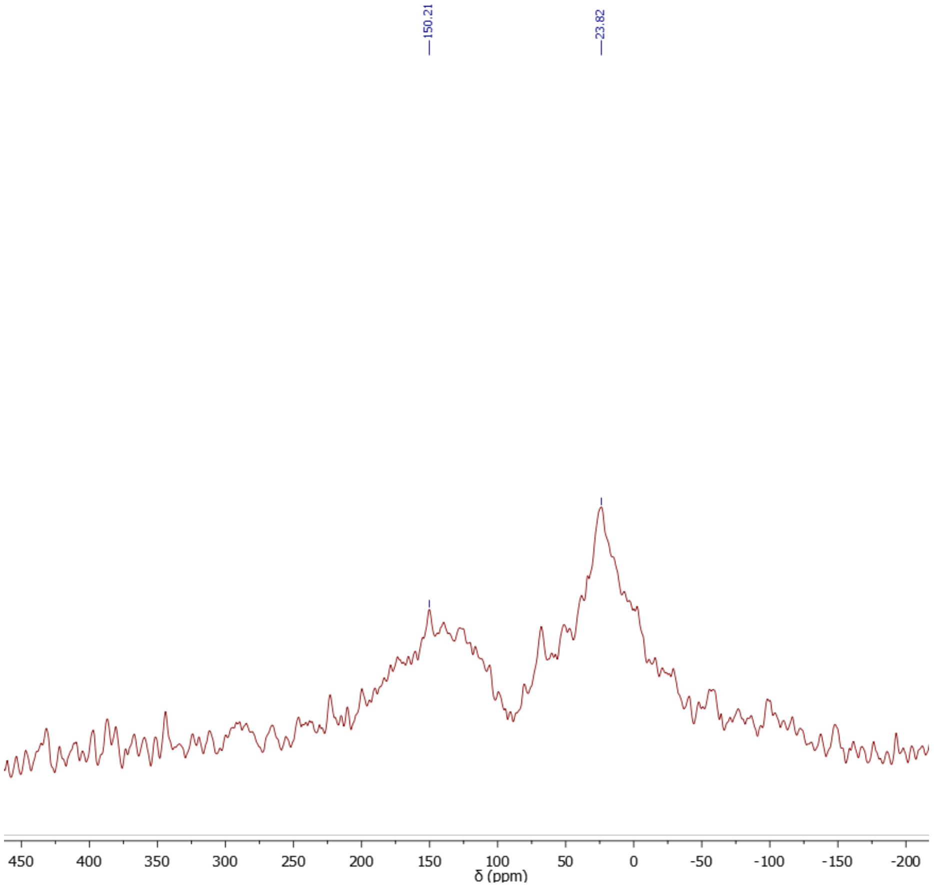

Supplementary Figure 57: MAS-SS $^{13}C/^{1}H$ NMR spectrum of isolated black solid from the defluorination of **1j** using a 2.5 mm rotor. The spectrum shows 2 broad peaks representing a distribution of aliphatic and carbonyl-containing species.

8. Total Defluorination of HFC-134a and Near Total Defluorination of HFC-125:

8.1 Equivalent Screening for KO^{t}Bu in THF for Defluorination of HFC-134a

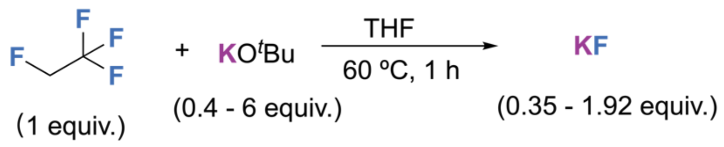

Supplementary Scheme 48: Reaction scheme of Equivalent Screening for KO*t*Bu in THF for Defluorination of HFC-134a.

In a dinitrogen containing glovebox, KO*t*Bu (5.8 - 87.5 mg, 0.052 - 0.78 mmol) was dissolved in anhydrous THF (0.5 ml) and transferred to a J Young NMR tube. The resultant solution was degassed via freeze-pump-thaw technique and HFC-134a was added (1.4 bar, 0.13 mmol). The mixture was inverted 10 times and heated at 60 °C for a reaction time of 1 hour, the reaction mixture was inverted for 10 times every 15 min. The solution was allowed to cool to room temperature before solvent was removed in vacuo. A white solid was obtained, water (0.4 mL) and NaOTf (0.1 mL, 1 M in deionised water) was added. KF was quantified by integration using ^{19}F NMR spectroscopy.

Supplementary Table 6: Screen of KO*t*Bu equivalents for the defluorination of HFC-134a in THF.

Equiv. KO*t*Bu	KF yield (%)
0.4	35%
2	125%
4	168%
6	192%

8.2. Solvent Screening for Defluorination of HFC-134a:

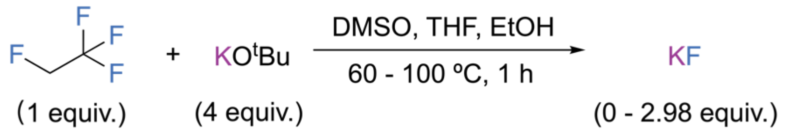

Supplementary Scheme 49: Reaction Scheme of Solvent Screening for Defluorination of HFC-134a.

In a dinitrogen containing glovebox, KO*t*Bu (58.3 mg, 0.52 mmol) was dissolved in anhydrous DMSO, THF or Ethanol (0.5 ml) and transferred to a J Young NMR tube. The resultant solution was degassed via freeze-pump-thaw technique and HFC-134a was added (1.4 bar, 0.13 mmol). The mixture was inverted 10 times and heated at 60 - 100 °C for a reaction time of 1 hour, the reaction mixture was inverted for 10 times every 15 min. The THF and ethanol solution was allowed to cool to room temperature before solvent was removed in vacuo. For THF and EtOH as solvent, white solids was obtained, water (0.4 ml) and NaOTf (0.1 ml, 1 M in deionised water) was added. KF was quantified by integration using ^{19}F NMR spectroscopy. For DMSO as solvent, a black solution with suspension was obtained, water (0.4 ml) and NaOTf (0.1 ml, 1 M in deionised water) was added directly. KF was quantified by integration using ^{19}F NMR spectroscopy.

Supplementary Table 7a: Screen of solvent for the defluorination of HFC-134a using KO*t*Bu.

**Solvent:**	**Temperature:**	**KF yield**
DMSO	100 °C	298%
THF	60 °C	168%
Ethanol	60 °C	0%

8.3. Base Screening in DMSO for Defluorination of HFC-134a:

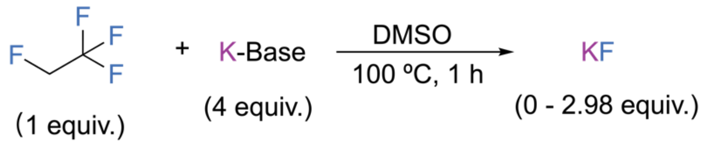

Supplementary Scheme 50: Reaction Scheme of Base Screening in DMSO for Defluorination of HFC-134a.

<u>Defluorination procedure using KOEt:</u>

In a dinitrogen containing glovebox, KOEt (43.8 mg, 0.52 mmol) was added to anhydrous DMSO (0.5 mL). KOEt partially dissolved and obtained orange solution with orange suspension. The mixture was transferred into a J Young NMR tube and degassed via freeze-pump-thaw technique. HFC-134a was added (1.4 Bar, 0.13 mmol) to the mixture and the mixture was inverted 10 times then heated at 100 °C for 1 h, the reaction mixture was inverted for 10 times every 15 min. Obtained black solution with suspension. Water (0.4 mL) and NaOTf (0.1 mL, 1M in deionised water) was added directly for KF quantification in ^{19}F NMR spectroscopy.

<u>Defluorination procedure using KOH:</u>

Outside of glovebox, KOH (146.3 mg, 2.61 mmol) was attempted for dissolution in DMSO (0.5 mL) heated at 100 °C for 1 h, the reaction mixture was inverted for 10 times every 15 min. Obtained cloudy solution with solids. The mixture was transferred into a J Young NMR tube, degassed via freeze-pump-thaw technique and HFC-134a was added (1.4 Bar, 0.13 mmol). The mixture was inverted 10 times and heated at 100 °C for 1 h, the reaction mixture was inverted for 10 times every 15 min. Obtained colourless solution with white solids. Water (0.4 mL) and NaOTf (0.1 mL, 1M in deionised water) was added directly for KF quantification in ^{19}F NMR spectroscopy.

Supplementary Table 7b: Base Screen for the defluorination of HFC-134a.

Base:	KF Yield
KOtBu	298%
KOEt	230%
KOH	0%
K_3PO_4*	N/A

*Reaction not performed due to insufficient solubility of base in DMSO

8.4. Temperature and Time Study for 4 Equivalent of KO*t*Bu in DMSO for Defluorination of HFC-134a:

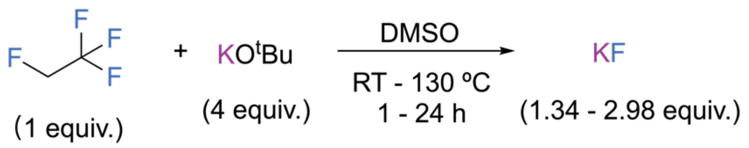

Supplementary Scheme 51: Reaction Scheme of Temperature and Time Study for 4 Equivalent of KO*t*Bu in DMSO for Defluorination of HFC-134a.

In a dinitrogen containing glovebox, KO*t*Bu (58.3 mg, 0.52 mmol) was dissolved in anhydrous DMSO (0.5 ml) and transferred to a J Young NMR tube. The resultant solution was degassed via freeze-pump-thaw technique and HFC-134a was added (1.4 bar, 0.13 mmol). The mixture was inverted 10 times and heated at RT - 130 °C for a reaction time of 1 -24 hour, for the 1-hour experiments, the reaction mixture was inverted for 10 times every 15 min. For the 24-hour experiments, the reaction mixture was inverted for 10 times evert 15 min for the first hour and left undisturbed for the rest of heating time. A black solution with suspension was obtained, water (0.4 ml) and NaOTf (0.1 ml, 1 M in deionised water) was added. KF was quantified by integration using ^{19}F NMR spectroscopy.

Supplementary Table 8: Time and temperature screen of KO*t*Bu in DMSO for the defluorination of HFC-134a.

Temperature:	Time:	KF Yield
100 °C	1 h	298%
100 °C	24 h	285%
25 °C	1 h	134%
25 °C	24 h	261%
60 °C	1 h	214%
80 °C	1 h	267%
130 °C	1 h	285%

8.5. Equivalent Screening for KO^{t}Bu in DMSO for Defluorination of HFC-134a:

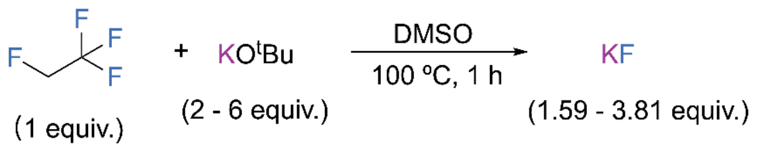

Supplementary Scheme 52: Reaction Scheme of Equivalent Screening for KO*t*Bu in DMSO for Defluorination of HFC-134a.

In a dinitrogen containing glovebox, KO*t*Bu (29.3 - 87.5 mg, 0.26 - 0.78 mmol) was dissolved in anhydrous DMSO (0.5 ml) and transferred to a J Young NMR tube. The resultant solution was degassed via freeze-pump-thaw technique and HFC-134a was added (1.4 bar, 0.13 mmol). The mixture was inverted 10 times and heated at 100 °C for a reaction time of 1 hour, the reaction mixture was inverted for 10 times every 15 min. A black solution with suspension was obtained, water (0.4 ml) and NaOTf (0.1 ml, 1 M in deionised water) was added. KF was quantified by integration using ^{19}F NMR spectroscopy.

Supplementary Table 9: KO*t*Bu equivalent screen for the defluorination of HFC-134a in DMSO.

Equiv. KO*t*Bu	KF Yield:
2	159%
4	298%
5	351%
6	381%

8.6. Heating Time Study for 6 Equivalent of KO*t*Bu in DMSO for Defluorination of HFC-134a:

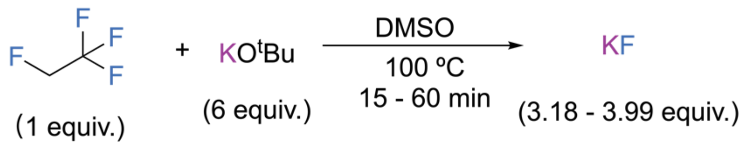

Supplementary Scheme 53: Heating Time Study for 6 Equivalent of KO*t*Bu in DMSO for Defluorination of HFC-134a.

In a dinitrogen containing glovebox, KO*t*Bu (87.5 mg, 0.78 mmol) was dissolved in anhydrous DMSO (0.5 ml) and transferred to a J Young NMR tube. The resultant solution was degassed via freeze-pump-thaw technique and HFC-134a was added (1.4 bar, 0.13 mmol). The mixture was inverted 10 times and heated at 100 °C for a reaction time of 15 – 60 min. For the 30-min and 60-min reactions, the reaction mixture was inverted 10 times every 15 min. For the 15-min reaction, the reaction mixture was inverted 10 times every 5 min. A black solution with suspension was obtained, water (0.4 ml) and NaOTf (0.1 ml, 1 M in deionised water) was added. KF was quantified by integration using ^{19}F NMR spectroscopy.

Supplementary Table 10: Reaction time screen with 6 equiv. of KO*t*Bu for the defluorination of HFC-134a.

Temperature	Reaction Time:	KF yield
100 °C	60 min	381%
100 °C	30 min	>399%
100 °C	15 min	318%
25 °C	60 min	279%

8.7. Heating Time Study and Effect of Shaking for 5 Equivalent of KO*t*Bu in DMSO for Defluorination of HFC-134a:

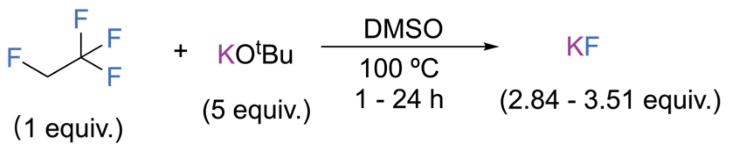

Supplementary Scheme 54: Reaction Scheme of Heating Time Study and Effect of Shaking for 5 Equivalent of KO*t*Bu in DMSO for Defluorination of HFC-134a.

In a dinitrogen containing glovebox, KO*t*Bu (72.9 mg, 0.65 mmol) was dissolved in anhydrous DMSO (0.5 ml) and transferred to a J Young NMR tube. The resultant solution was degassed via freeze-pump-thaw technique and HFC-134a was added (1.4 bar, 0.13 mmol). The mixture was inverted 10 times and heated at 100 °C for a reaction time of 1 – 24 h, the reaction mixture was inverted for 10 times every 15 min, 60 min or was not shaken at all. A black solution with suspension was obtained, water (0.4 ml) and NaOTf (0.1 ml, 1 M in deionised water) was added. KF was quantified by integration using ^{19}F NMR spectroscopy.

Supplementary Table 11: Reaction time screen and effect of shaking frequency with 5 equiv. of KO*t*Bu for the defluorination of HFC-134a.

Reaction Time:	KF Yield:	Shaking Frequency:
1 h	315%	Every 15 min
1 h	351%	Every 15 min
2 h	317%	Every 15 min
2 h	284%	Every 60 min
24 h	345%	No Shaking

8.8. 1 Bar (Absolute) Pressure Study of HFC-134a with 6 Equivalent of KOtBu in DMSO:

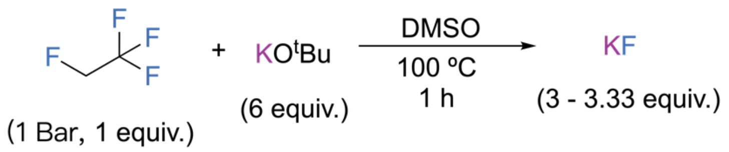

Supplementary Scheme 55: Reaction Scheme of 1 Bar (Absolute) Pressure Study of HFC-134a with 6 Equivalent of KO*t*Bu in DMSO.

In a dinitrogen containing glovebox, KO*t*Bu (62.5 g, 0.56 mmol,) was dissolved in anhydrous DMSO (0.5 ml) and transferred to a J Young NMR tube. The resultant solution was degassed via freeze-pump-thaw technique and HFC-134a was added (1.4 bar, 0.13 mmol). The mixture was inverted 10 times and heated at 100 ºC for a reaction time of 1 h. A black solution with suspension was obtained, water (0.4 ml) and NaOTf (0.1 ml, 1 M in deionised water) was added. KF was quantified by integration using ^{19}F NMR spectroscopy.

Supplementary Table 12: Defluorination of HFC-134a at 1 bar with 6 Equivalent of KOtBu in DMSO.

**Entry:**	**KF Yield:**
Run 1	333%
Run 2	300%

8.9. Example Quantitative ^{19}F NMR Spectra for Defluorination Fluoride Yield:

KF quantification was conducted by addition of known amount of NaOTf standard solution to a water-DMSO mixture. More water was added if KF peak is broad. NaOTf is referenced to –77.8 ppm and its integral is standardised to 3.

KF% was calculated by the following equation:

$$KF\% = \frac{(fluoride\ integral \times NaOTf\ concentration \times mL\ of\ NaOTf\ solution\ added)}{mmol\ of\ HFC} \times 100$$

For example:

To a reaction mixture, 0.1 mL of 1 M NaOTf was added along with 0.4 mL of water to a completed reaction of 0.13 mmol HFC-134a and 0.78 mmol of KO*t*Bu. Standardised NaOTf signal to 3.00 and obtained sharp singlet at –117 ppm:

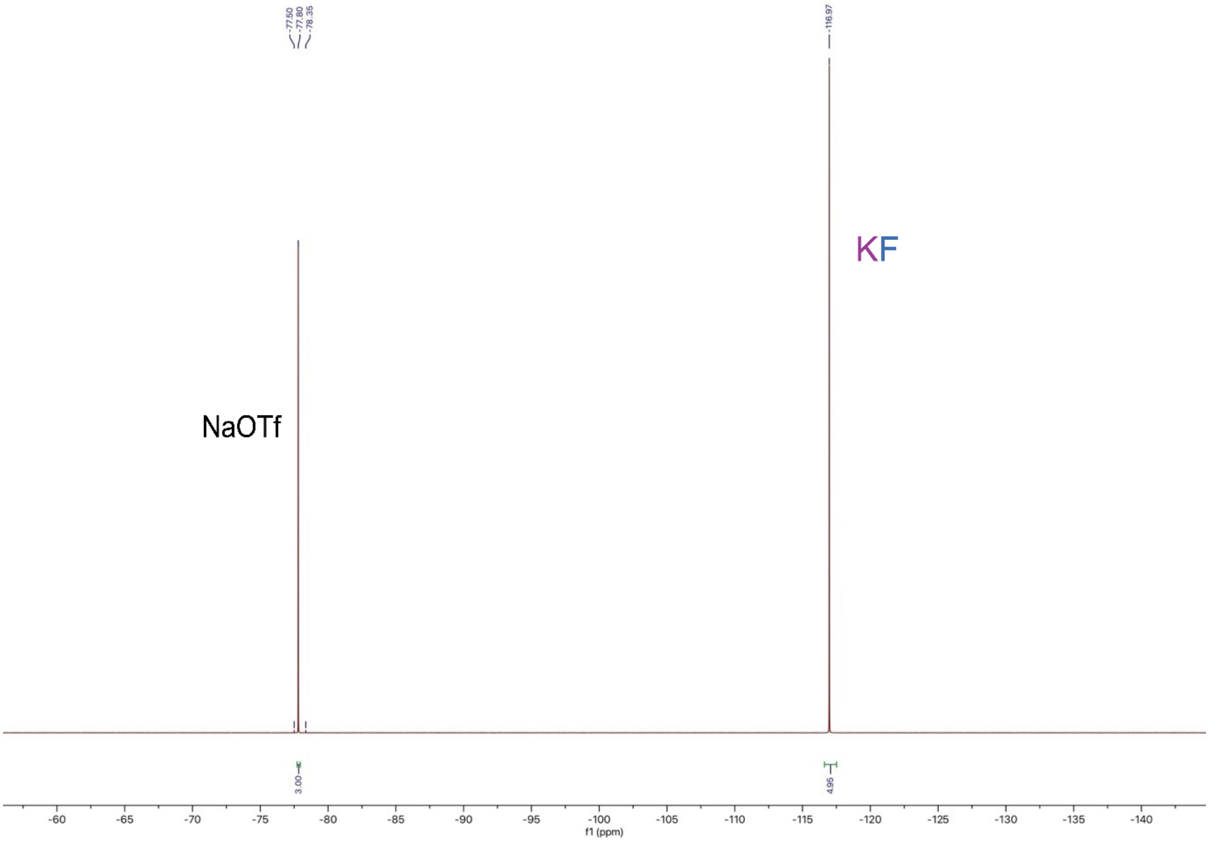

Supplementary Figure 57b: Example Quantitative ^{19}F NMR Spectra for KF% calculation.

In this case, the calculation of KF% was:

$$KF\% = \frac{(4.95 \times 1 \times 0.1)}{0.13} \times 100 = 380.7\% = 381\%$$

8.10. Temperature and Time Study of 7 Equivalent KOtBu in DMSO for Defluorination of HFC-125:

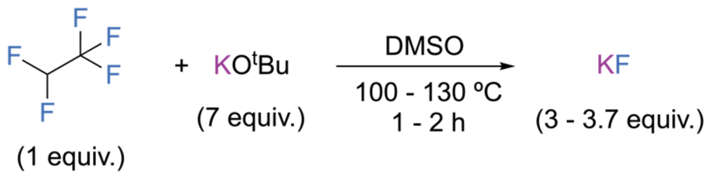

Supplementary Scheme 56: Reaction Scheme of Temperature and Time Study of 7 Equivalent KO*t*Bu in DMSO for Defluorination of HFC-125.

In a dinitrogen containing glovebox, KO*t*Bu (102.1 mg, 0.91 mmol) was dissolved in anhydrous DMSO (0.5 ml) and transferred to a J Young NMR tube. The resultant solution was degassed via freeze-pump-thaw technique and HFC-125 was added (1.4 bar, 0.13 mmol). The mixture was inverted 10 times and heated at 100 - 130 °C for a reaction time of 1 –2 h. A black solution with suspension was obtained, water (0.4 ml) and NaOTf (0.1 ml, 1 M in deionised water) was added. KF was quantified by integration using ^{19}F NMR spectroscopy.

Supplementary Table 13: Screen of reaction time and temperature with 7 equiv. of KO*t*Bu.

**Temperature:**	**Heating Time:**	**KF Yield:**
100 °C	1 h	300%
130 °C	2 h	370%

9. Mechanism of Total Defluorination of HFC-134a and Final Product:

In a dinitrogen containing glovebox, KO*t*Bu (14.6 - 87.5 mg, 0.13 - 0.78 mmol) was added to 0.5 mL of DMSO-d_6 or DMSO-h_6, obtained colourless solution. The solution was degassed via freeze-pump-thaw and HFC-134a (1.4 Bar, 0.13 mmol) was added. The interface of liquid-gas turned black immediately and the reaction was inverted for 10 times and multinuclear NMR spectroscopy was used to investigate and assign reaction intermediates and side products.

Trifluoroethylene was observed when 1 equivalent of KO*t*Bu (14.6 mg, 0.13 mmol) was reacted with HFC-134a (1.4 Bar, 0.13 mmol):

^{19}F NMR (376 MHz, DMSO-d_6, 25 °C): δ -100.7 (dd, 1F, $^2J_{FF}$ = 85.0, $^3J_{FF}$ = 30.8 Hz), -125.9 (dd, 1F, $^3J_{FF}$ = 116.3, $^2J_{FF}$ = 85.0 Hz), -204 (m, 1F)

NMR spectroscopy data in accordance with literature[63].

Isobutene was observed from trap-to-trap distillation of the volatile reaction products when 6 equivalent of KO*t*Bu (87.5 mg, 0.78 mmol) was reacted with HFC-134a (1.4 Bar, 0.13 mmol):

^{1}H NMR (400 MHz, C_6D_6, 25 °C): δ 1.60 (s, 6H, $C(CH_3)_2$) 4.75 (m, 2H, CH_2)

Proton NMR spectroscopy data in C_6D_6 is in accordance with literature[64].

^{1}H NMR (400 MHz, DMSO-d_6, 25 °C) δ 1.68 (s, 6H, $C(CH_3)_2$) 4.65 (s, 2H, CH_2)

Dried KF from flow reaction using **Method 2** (see page S180) was dissolved in D_2O, tert-Butyl α-fluoroacetate and potassium 2-(tert-butoxy) acetate were observed as minor species:

Potassium 2-fluoroacetate:

^{19}F NMR (376 MHz, D_2O, 25 °C) δ -217.2 (t, 1F, $^2J_{FH}$ = 48.4 Hz, CH_2F).

^{1}H NMR (400 MHz, D_2O, 25 °C) δ 4.79 (d, $^2J_{HF}$ = 48.3 Hz, CFH_2)

NMR spectroscopy data in accordance with literature[65].

Potassium 2-(tert-butoxy) acetate:

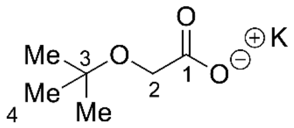

^{1}H NMR (400 MHz, D_2O, 25 °C): δ 1.18 (s, 9H, $C(CH_3)_3$), 3.83 (s, 2H, OCH_2CO).

^{13}C HMR (126 MHz, D_2O, 25 °C): δ 26.4(C(4)), 61.7(C(2)), 74.9(C(3)), 179.5(C(1))

No literature record for exact same compound, NMR spectroscopy data in accordance with corresponding acid.

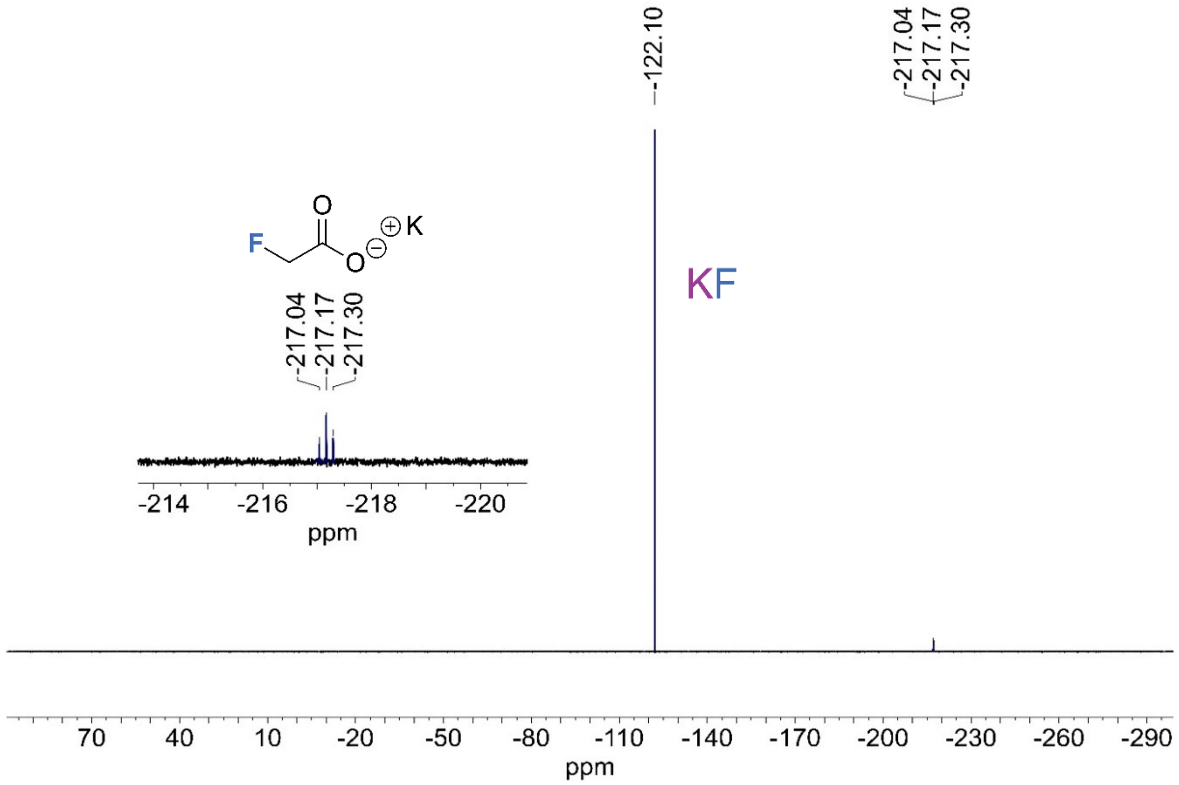

Supplementary Figure 58: ^{19}F NMR of Method 2 Isolated KF in D_2O.

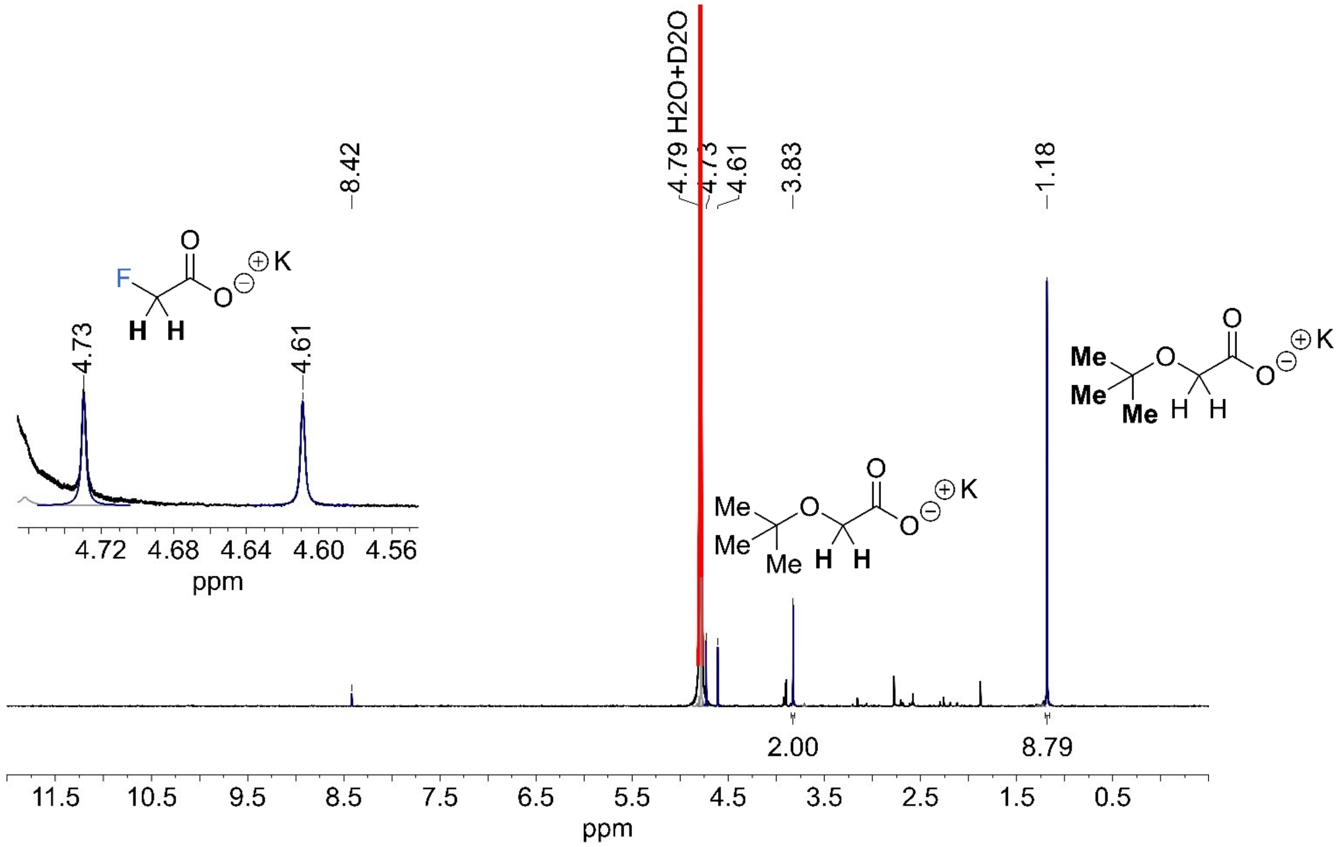

Supplementary Figure 59: ^{1}H NMR of Method 2 Isolated KF in D_2O

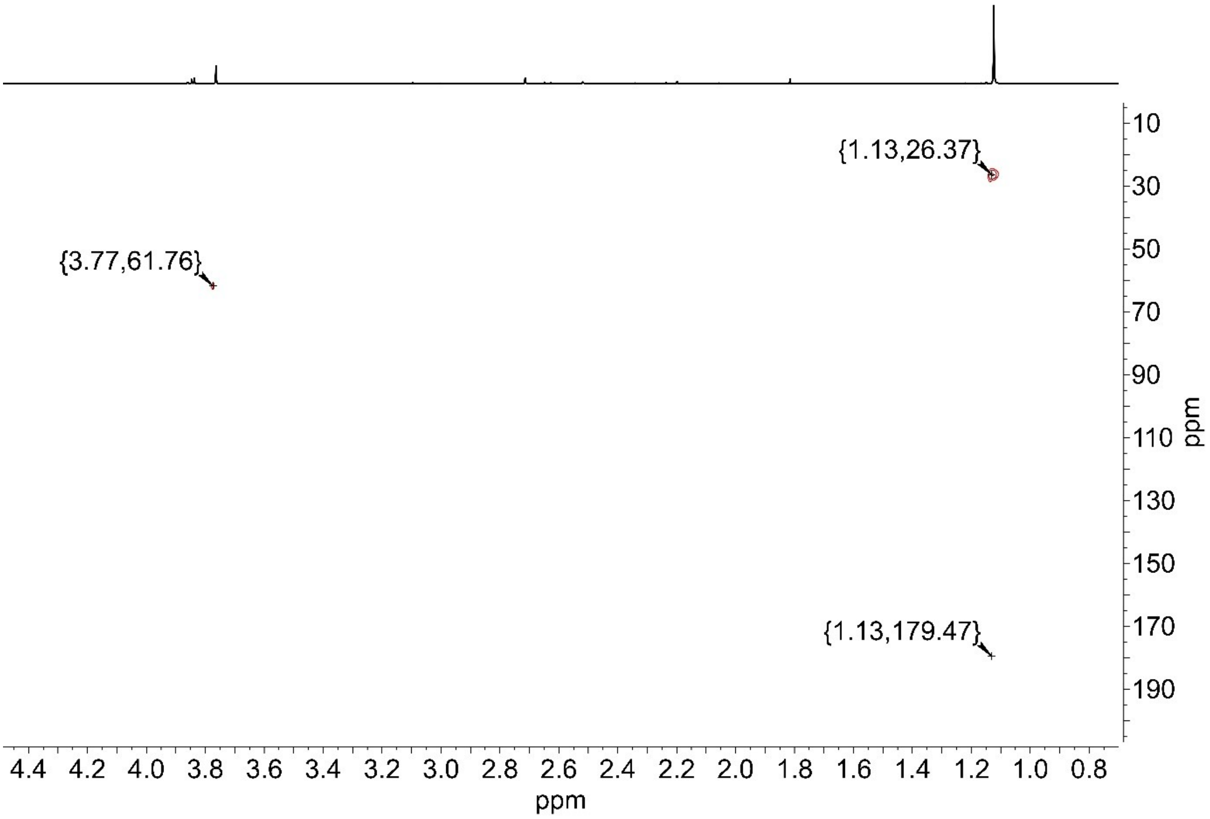

Supplementary Figure 60: ^{1}H-^{13}C HSQC of Method 2 Isolated KF in D_2O.

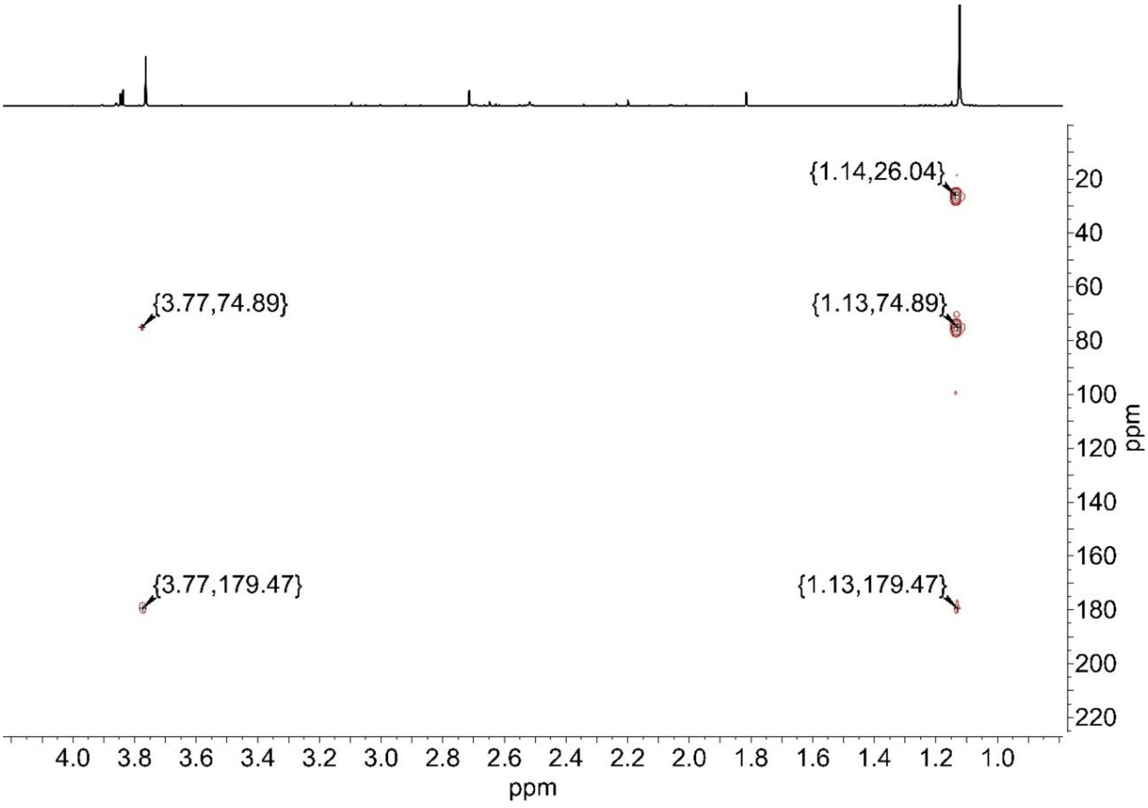

Supplementary Figure 61: ^{1}H-^{13}C HMBC of Method 2 Isolated KF in D_2O.

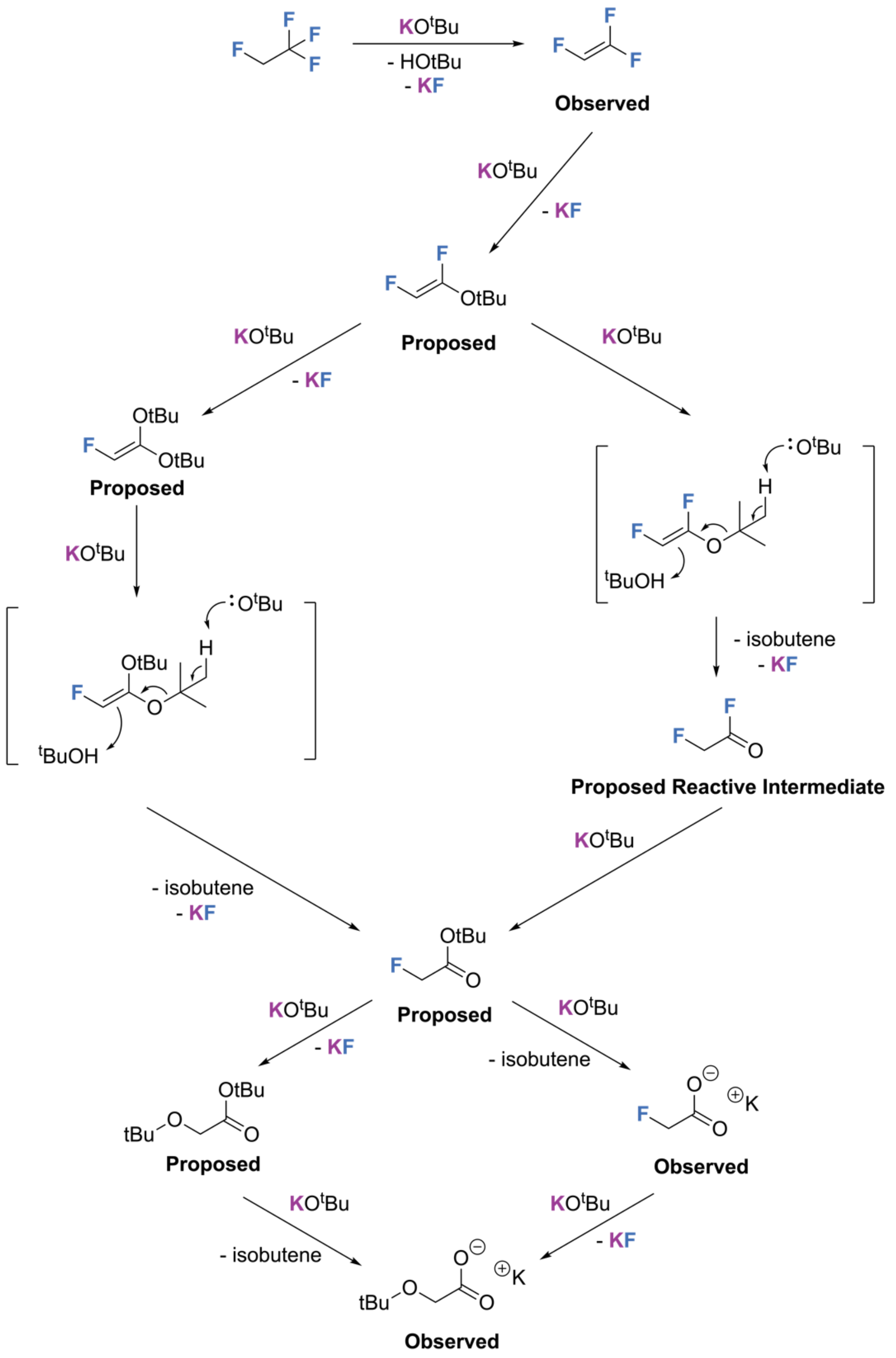

Supplementary Scheme 57: Reaction Mechanism Proposal for Total Defluorination of HFC-134a

10. Transfer fluorination using PFOA (**1u**)

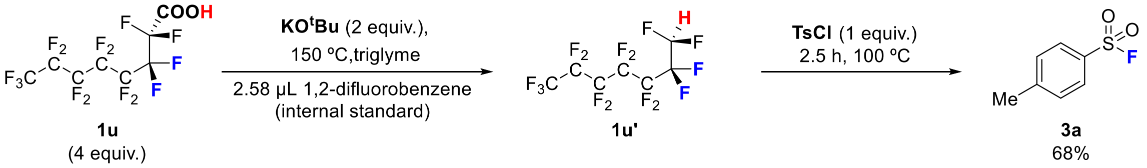

Supplementary Scheme 58: Transfer fluorination of TsCl using **1u**.

A procedure for the screening of perfluorooctanoic acid was performed as follows (Supplementary Scheme 58): ***Step 1: Decarboxylation and defluorination:*** In a dinitrogen containing glovebox, perfluorooctanoic acid (0.105 mmol, 43.6 mg) and KO*t*Bu (0.053 mmol, 5.9 mg) were dissolved in 0.8 mL triglyme and transferred into a J. Youngs NMR tube, followed by the addition of 2.58 μL *o*-DFB as internal standard. The NMR tube was sealed, removed from the glovebox and transferred into a 150 °C oil bath. After a reaction time of 3 hours, the tube was removed from the oil bath, allowed to cool to room temperature. ***Step 2: Fluorination:*** Inside a dinitrogen containing glovebox, tosyl chloride (0.025 mmol, 4.7 mg) in 0.2 mL triglyme was added to the J Young NMR tube. The NMR tube was then sealed, removed from the glovebox and transferred into a 100 °C oil bath. After a reaction time of 2.5 hours, the tube was removed from the oil bath and allowed to cool to room temperature, after which 68% yield of tosyl fluoride (TsF; **3a**) was determined *via* quantitative ^{19}F NMR spectroscopy.

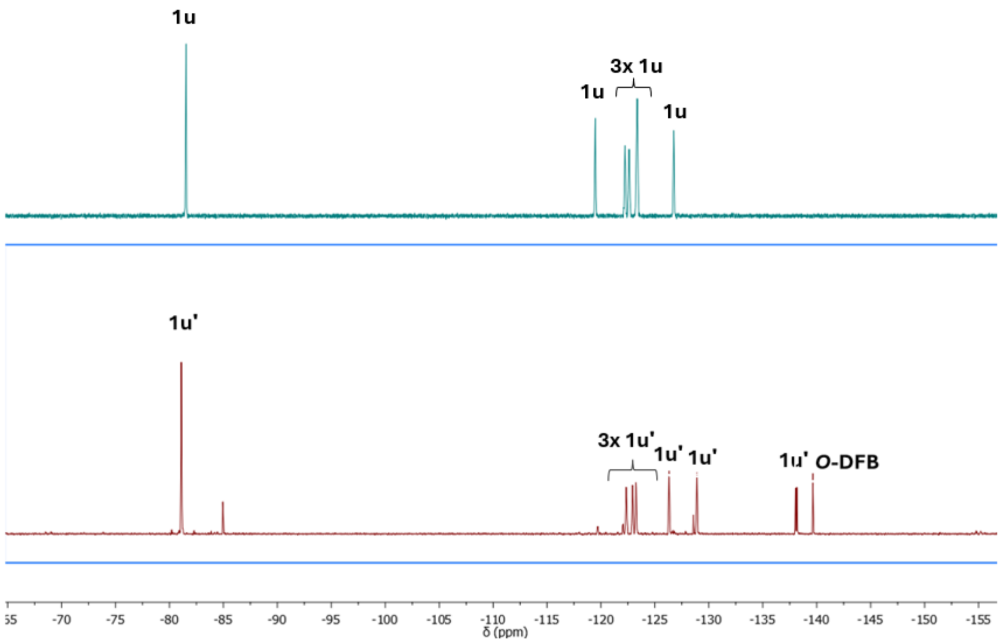

Supplementary Figure 62: ^{19}F NMR spectra of **1u** at the top and of **1u’** after decarboxylation/defluorination step below (both recorded at: 377 MHz, triglyme-h18, 25 °C).

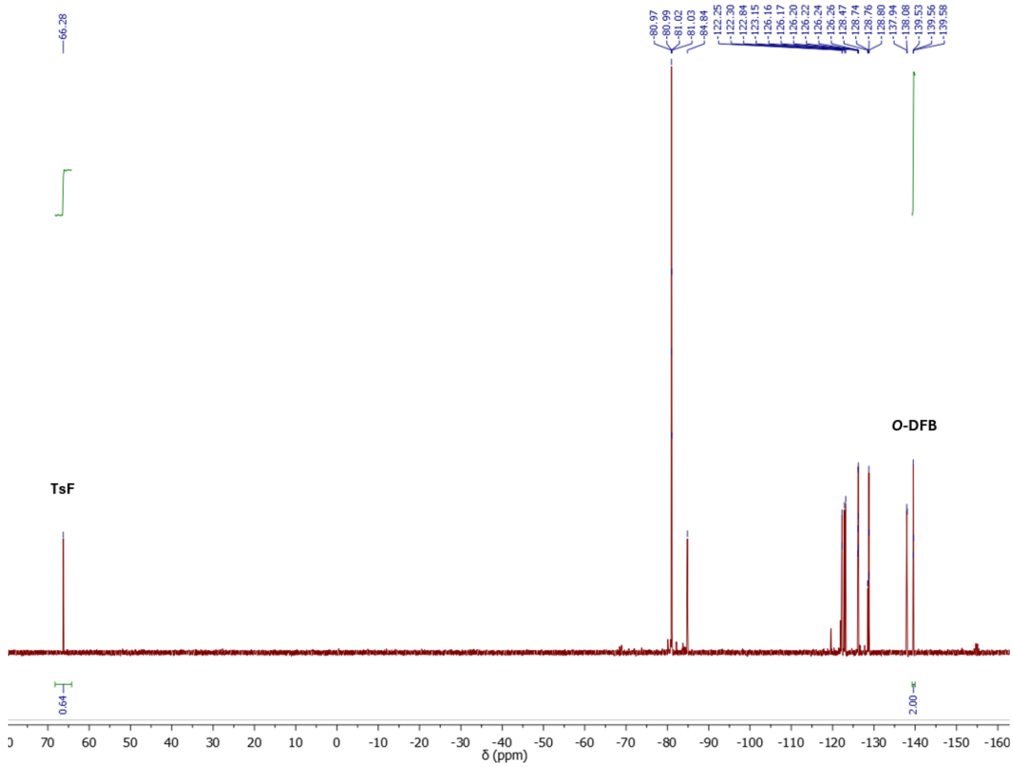

Supplementary Figure 63: ^{19}F NMR spectrum of fluorination reaction of TsCl using perfluorooctanic acid as the donor substrate (377 MHz, triglyme-h18, 25 °C). 0.105 mmol **1u** and 0.025 mmol TsCl led to the formation of 0.017 mmol TsF.

Preparative scale procedure for the transfer fluorination of tosyl chloride using perfluorooctanoic acid as a fluorine donor:

Step 1: Decarboxylation and defluorination: In a dinitrogen containing glovebox, perfluorooctanoic acid (1.57 mmol, 651.0 mg) and KO*t*Bu (0.782 mmol, 87.8 mg) was dissolved in 12 mL triglyme and transferred into a J. Youngs ampoule. The ampoule was sealed, removed from the glovebox and transferred into a 150 °C oil bath. After a reaction time of 3 hours, the ampoule was removed from the oil bath, allowed to cool to room temperature. ***Step 2: Fluorination:*** Inside a dinitrogen containing glovebox, tosyl chloride (0.392 mmol, 74.7 mg) in 3 mL triglyme was added to the J Young ampoule. The ampoule was then sealed, removed from the glovebox and transferred into a 100 °C oil bath. After a reaction time of 2.5 hours, the ampoule was removed from the oil bath and allowed to cool to room temperature. The crude reaction mixture was diluted with 15 ml toluene and washed with water (5x5 ml). The combined organic extracts were dried over magnesium sulfate, filtered, and concentrated *in vacuo* by rotary evaporation. The crude oil was purified by automated biotage flash column chromatography eluted with 2-15% ethyl acetate in *n*-hexane. A white solid, **3a**, was isolated (30.1 mg, 0.173 mg, 45%).

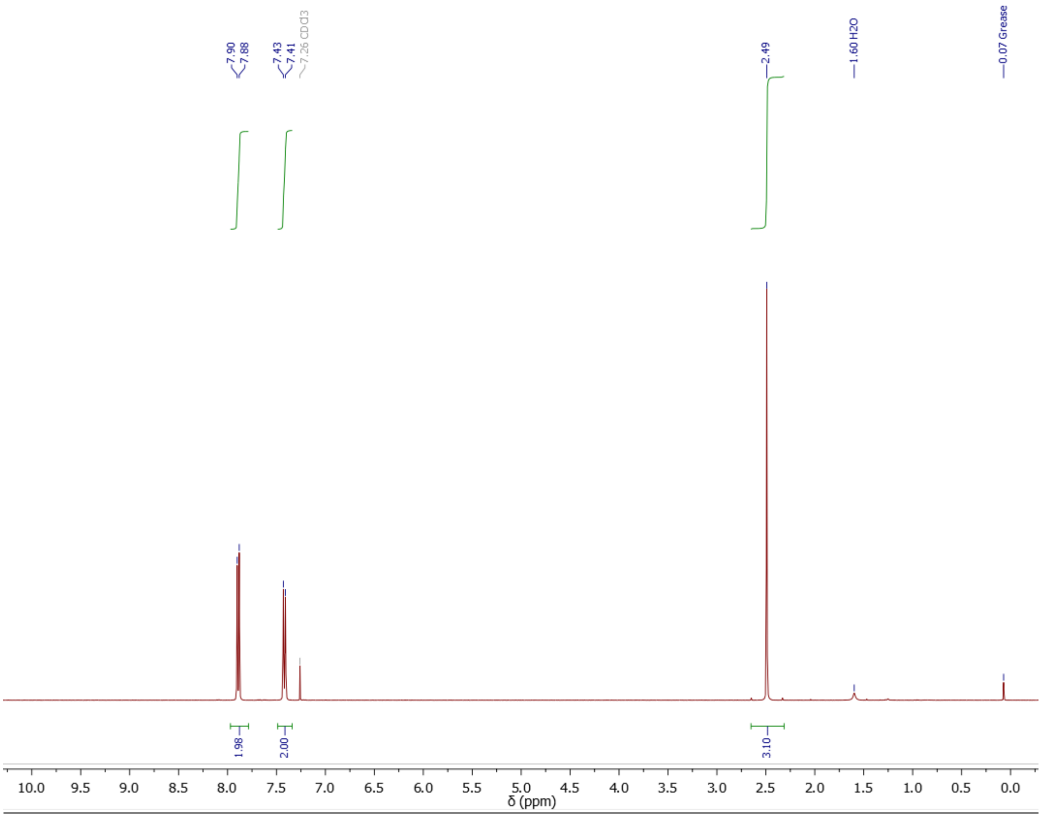

Supplementary Figure 64: ^{1}H NMR spectrum of tosyl fluoride 3a (400 MHz, $CDCl_3$, 25 °C).

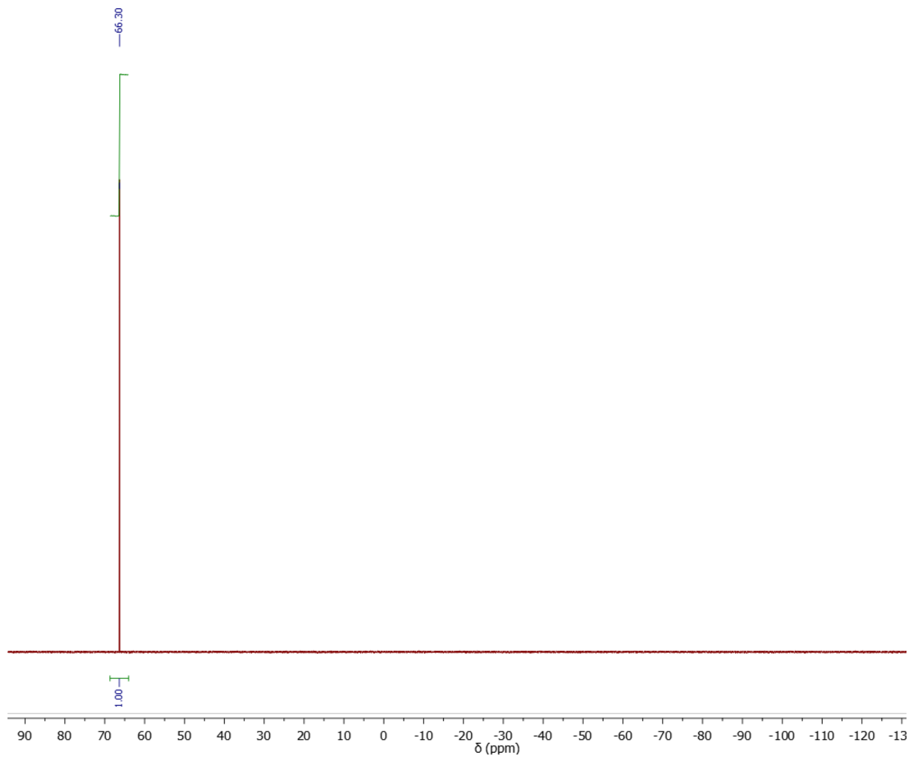

Supplementary Figure 65: ^{19}F NMR spectrum of tosyl fluoride 3a (400 MHz, CDCl3, 25 °C).

<u>**Transferring multiple equivalents of fluoride from perfluorooctanic acid, 1u**</u>

<u>**NMR scale procedure for the full defluorination of perfluorooctanoic acid:**</u> ***Step 1: Decarboxylation and defluorination:*** In a dinitrogen containing glovebox, perfluorooctanoic acid (0.037 mmol, 15.3 mg) and KO*t*Bu (0.539 mmol, 60.5 mg) was dissolved in 0.8 mL DMSO and transferred into a J. Youngs NMR tube, followed by the addition of 3.5 µL *o*-DFB as internal standard. The NMR tube was sealed, removed from the glovebox and transferred into a 150 °C oil bath. After a reaction time of 60 minutes, the tube was removed from the oil bath, allowed to cool to room temperature.

KF quantification: The solution was decanted into a vial and 2 mL water was added, followed by the addition of sodium triflate (11.6 mg, 0.067 mmol). The mixture was stirred at 25 °C for 30 mins. An aliquot was taken and the amount of KF was quantified using ^{19}F NMR spectroscopy as 0.41 mmol of KF, 11 equivalents of KF removed from perfluorooctanic acid.

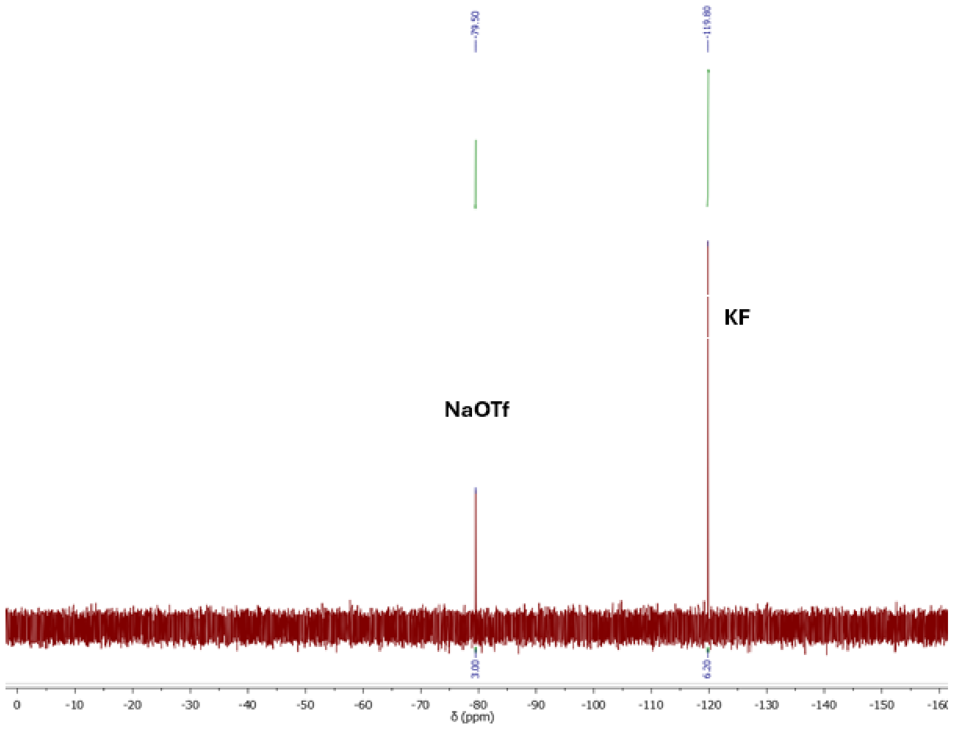

Supplementary Figure 66: KF quantification of the defluorination reaction of **1u** using ^{19}F NMR spectroscopy (377 MHz, triglyme-h18, 25 °C). 0.036 mmol **1u** and 0.539 mmol KOtBu led to the formation of 0.42 mmol KF.

NMR scale procedure for the transfer fluorination of chlorotriphenylsilane using perfluorooctanoic acid as a fluorine donor (Supplementary Scheme 59):

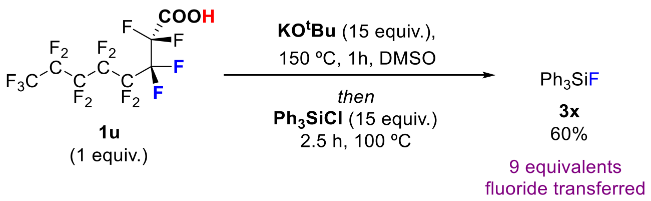

Supplementary Scheme 59: Transfer fluorination of Ph_3SiCl using **1u** as a limiting reagent.

Step 1: Decarboxylation and defluorination: In a dinitrogen containing glovebox, perfluorooctanoic acid (0.036 mmol, 14.9 mg) and KO*t*Bu (0.537 mmol, 60.3 mg) was dissolved in 0.8 mL DMSO and transferred into a J. Youngs NMR tube. The NMR tube was sealed, removed from the glovebox and transferred into a 150 °C oil bath. After a reaction time of 60 minutes, the tube was removed from the oil bath, allowed to cool to room temperature. ***Step 2: Fluorination:*** Inside a dinitrogen containing glovebox, chlorotriphenylsilane (0.532 mmol, 157 mg) in 1.0 mL DMSO was added to the J Young NMR tube. The NMR tube was then sealed, removed from the

glovebox and transferred into a 100 °C oil bath. After a reaction time of 2.5 hours, the tube was removed from the oil bath and allowed to cool to room temperature, followed by addition of 1,2-difluorobenzene (15 µl, 0.15 mmol). After which 60% yield of fluorotriphenylsilane (Ph_3SiF; **3x**) was determined *via* quantitative ^{19}F NMR spectroscopy. 9 equivalents of fluoride transferred from perfluorooctanic acid.

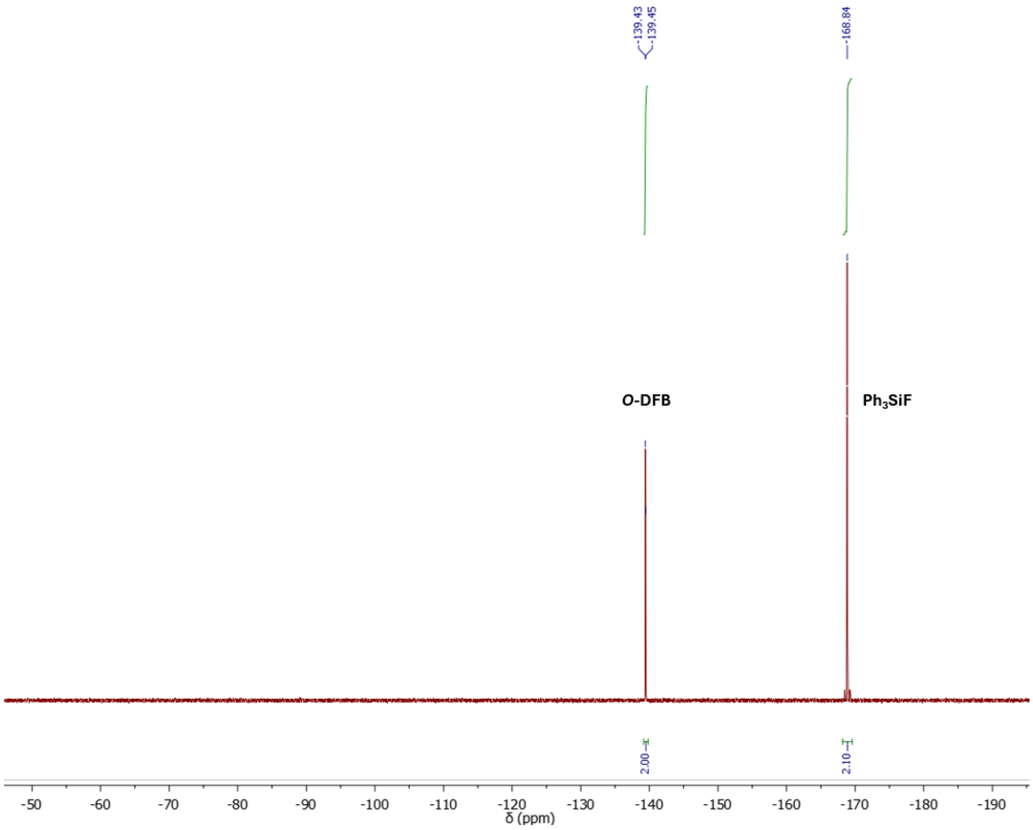

Supplementary Figure 67:: ^{19}F NMR spectrum of fluorination reaction of Ph_3SiCl using **1u** as the donor substrate (377 MHz, triglyme-h18, 25 °C). 0.036 mmol **1u** and 0.53 mmol Ph_3SiCl led to the formation of 0.32 mmol Ph_3SiF.

11. Synthesis of fluorochemicals by transfer fluorination

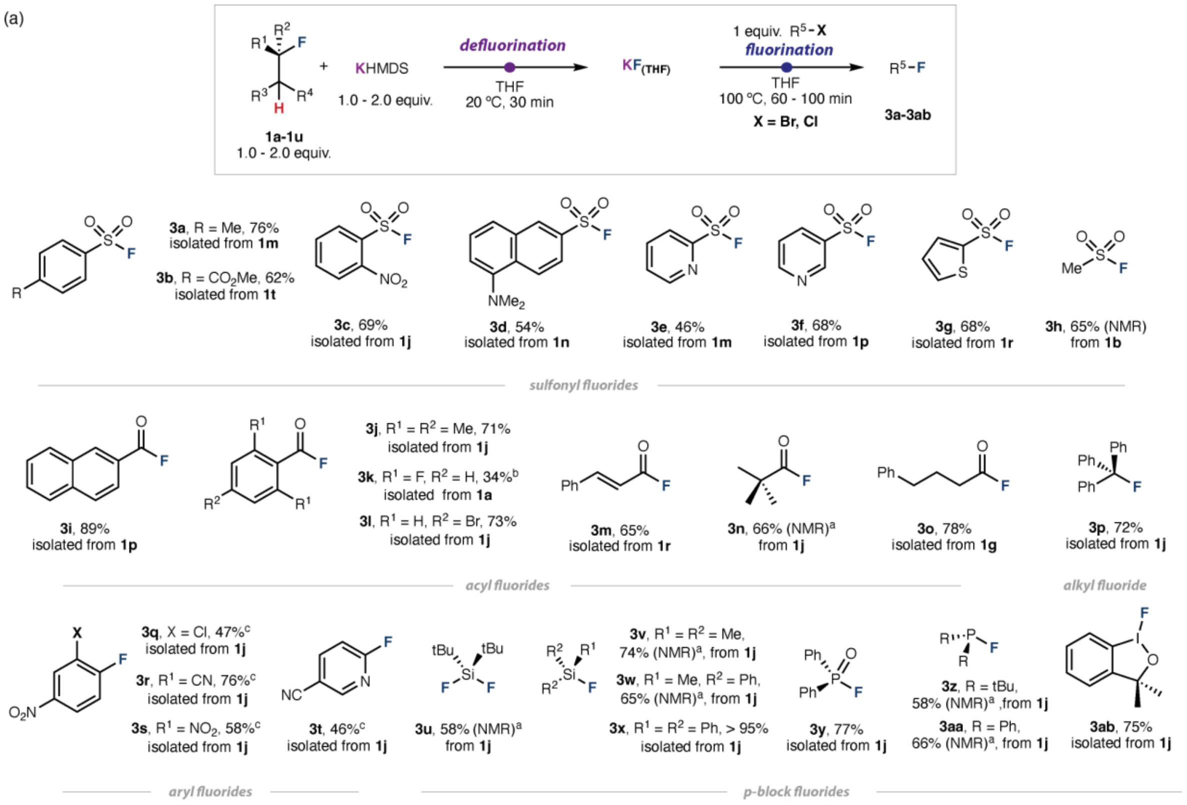

Supplementary Figure 68: List of successful electrophiles in transfer fluorination. For each the fluorine donor used in the synthesis is listed by its compound number (see Supplementary Figure 1).

11.1. S–F bond formation: Synthesis and characterisation of sulfonyl fluorides

Tosyl fluoride (3a) – CAS# 455-16-3

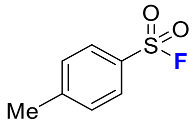

3a

CAUTION!!! The potential dehydrofluorinated variant of 1m is still subject of nephrotoxicity discussions, so caution needs to be followed when performing reaction with sevoflurane on larger scales.[4,5]

In a dinitrogen containing glovebox, KHMDS (5.98 g, 29.98 mmol) was dissolved in 28 mL dry THF and transferred to a 100 mL ampoule containing a stirrer bar. The ampoule was sealed and attached onto a Schlenk line in a fume hood and placed into a water bath of 20 °C, where the solution stirred. Under dinitrogen overpressure, a solution of sevoflurane (4.51 g, 22.54 mmol) in 5 mL dry THF was added dropwise within two minutes into the vessel while stirring. An immediate colour change from a colourless solution to a black suspension was observed as well as the formation of a small amount of gas in the initial few seconds. The vessel was sealed and the heterogenous mixture stirred for 90 minutes at 20 °C. After that, tosyl chloride (1.84 g, 98% purity, 9.47 mmol) in 11 mL dry THF was added under dinitrogen overpressure dropwise over a minute. The ampoule was sealed once again, the water bath replaced with a hot oil bath which was set to 100 °C, and the reaction mixture allowed to stir for 100 minutes at 100 °C. Once cooled to room temperature, the ampoule was opened, the mixture was filtered through filter paper using gravitation, removing a large amount of black solid. The residual solid was washed with 3x40 mL diethyl ether and the organic extracts were unified as well as concentrated *en vacuo* at 40 °C and p<10 mbar. The crude reaction mixture was then further purified *via* liquid chromatography (silica gel; 5:1 petroleum ether:ethyl acetate as eluent), giving **3a** as a colourless crystalline solid (1.20 g, 6.90 mmol, 73%; $R_f \approx 0.56$ (5:1 petroleum ether:ethyl acetate)).

^{1}H NMR (400 MHz, $CDCl_3$, 25 °C): δ = 7.89 (d, $^{3}J_{HH}$ = 7.9 Hz, 2H, Ar*H*), 7.42 (d, $^{3}J_{HH}$ = 7.9 Hz, 2H, Ar*H*), 2.49 (s, 3H, CH_3).

^{13}C{^{1}H} NMR (101 MHz, $CDCl_3$, 25 °C): δ = 147.2, 130.4, 130.2 (d, $^{2}J_{CF}$ = 24.7 Hz, CSO_2F), 128.6, 21.7.

^{19}F NMR (377 MHz, $CDCl_3$, 25 °C): δ = +66.2 (s, 1F, RSO_2F).

Spectroscopic data in agreement with reported data.[6]

Methyl 4-(fluorosulfonyl)benzoate (3b) – CAS# 124397-38-2

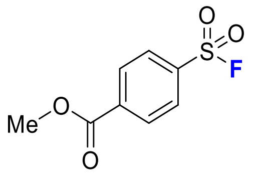

3b

In a dinitrogen containing glovebox, KHMDS (196 mg, 0.98 mmol) was dissolved in 4 mL dry THF and transferred to an ampoule containing a stirrer bar, after which a solution of 1H-perfluoropentane (170 mg, 0.63 mmol) in 0.5 mL THF was added quickly. The ampoule was sealed and attached onto a Schlenk line in a fume hood and placed into a water bath of 20 °C and stirred for 90 minutes. An immediate colour change from a colourless solution to a black suspension was observed. After that, 4-chlorosulfonyl-benzoic acid methyl ester (100 mg, 97% purity, 0.41 mmol) in 1 mL dry THF was added under dinitrogen overpressure dropwise over a minute. The ampoule was sealed, the water bath replaced with a hot oil bath which was already set to 100 °C, and the reaction mixture was allowed to stir for 100 minutes at 100 °C. Once cooled to room temperature, the ampoule was opened, and the mixture was diluted with 12 mL diethyl ether followed by filtration *via* silica plug (4 cm depth and 2.5 cm diameter) with diethyl ether as eluent. The reaction vessel was rinsed with 2x12 mL diethyl ether and each washing was filtered through the silica plug as well. The organic phases were combined and concentrated *en vacuo* at 40 °C and p<100 mbar. The crude reaction mixture was then further purified *via* hot recrystallisation (70 °C; 10:1 methanol:water), giving **3b** as a colourless crystalline solid after storing at -20 °C (56 mg, 0.25 mmol, 62%).

^{1}H NMR (400 MHz, $CDCl_3$, 25 °C): δ = 8.28 (d, $^{3}J_{HH}$ = 8.2 Hz, 2H, Ar*H*), 8.10 (d, $^{3}J_{HH}$ = 8.2 Hz, 2H, Ar*H*), 3.99 (s, 3H, CH_3).

^{13}C{^{1}H} NMR (101 MHz, $CDCl_3$, 25 °C): δ = 165.1, 136.8 (d, $^{2}J_{CF}$ = 26.2 Hz, *C*SO_2F), 136.6, 130.9, 128.7, 53.1.

^{19}F NMR (377 MHz, $CDCl_3$, 25 °C): δ = +65.8 (s, 1F, RSO_2*F*).

Spectroscopic data in agreement with reported data.[6]

2-Nitrobenzenesulfonyl fluoride (3c) – CAS# 433-98-7

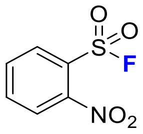

3c

In a dinitrogen containing glovebox, KHMDS (227 mg, 1.14 mmol) was dissolved in 4.2 ml THF and transferred to a 20 ml ampoule containing a stirrer bar. The reaction mixture degassed *via* freeze-pump-thaw technique before HFC-134a (1.4 bar, 15.8 mL, 8.80 mmol) was added while the solution stirred. A colour change from colourless solution to a black suspension was observed. The reaction mixture was allowed to stir vigorously for 55 minutes in a water bath of 20 °C. After that, 2-nitrobenzenesulfonyl chloride (153 mg; 98% purity; 0.68 mmol) in 1.4 mL dry THF was added under dinitrogen overpressure dropwise over a minute. The ampoule was sealed once again, the water bath replaced with a hot oil bath which was already set to 100 °C, and the reaction mixture was allowed to stir for 100 minutes at 100 °C. Once cooled to room temperature, the ampoule was opened, and the mixture was diluted with 15 mL diethyl ether followed by filtration *via* silica plug (7 cm depth and 2.5 cm diameter) with diethyl ether as eluent. The reaction vessel was rinsed with 3x15 mL diethyl ether and each washing was filtered through the silica plug as well. The organic phases were combined and concentrated *en vacuo* at 40 °C and p<100 mbar. The crude reaction mixture was then further purified *via* hot recrystallisation (50 °C; 10:1 methanol:water), and storing first at -20 °C, then 5 °C, gave **3c** as a colourless crystalline solid (97 mg, 0.47 mmol, 69%).

^{1}H NMR (400 MHz, C_6D_6, 25 °C): δ = 7.31 (dd, $^{3}J_{HH}$ = 7.7 Hz, $^{4}J_{HH}$ = 1.2 Hz, 1H, Ar*H*), 6.79 (d, $^{3}J_{HH}$ = 7.7 Hz, 1H, Ar*H*), 6.44–6.34 (m, 2H, 2 Ar*H*).

^{13}C{^{1}H} NMR (101 MHz, C_6D_6, 25 °C): δ = 148.1 (confirmed *via* ^{1}H-^{13}C-HMBC), 135.7, 132.2, 131.2, 126.5 (d, $^{2}J_{CF}$ = 28.9 Hz, *C*SO_2F, confirmed *via* ^{1}H-^{13}C-HMBC), 125.2.

^{19}F NMR (377 MHz, C_6D_6, 25 °C): δ = +65.5 (s, 1F, RSO_2*F*).

Spectroscopic data in agreement with reported data.[6]

Dansyl fluoride (3d) – CAS# 34523-28-9

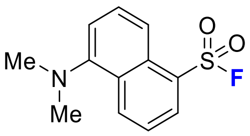

3d

In a dinitrogen containing glovebox, KHMDS (200 mg, 1.00 mmol) was dissolved in 4 mL dry THF and transferred to an ampoule containing a stirrer bar, after which a solution of isoflurane (140 mg, 0.76 mmol) in 0.5 mL THF was added quickly. The ampoule was sealed and attached onto a Schlenk line and placed into a water bath of 20 °C and stirred for 90 minutes. An immediate colour change from a colourless solution to a black suspension was observed. After that, dansyl chloride (178 mg, 96% purity, 0.63 mmol) in 3 mL dry THF was added under dinitrogen overpressure dropwise over a minute. The ampoule was sealed once again, the water bath replaced with a hot oil bath which was already set to 100 °C, and the reaction mixture was allowed to stir for 100 minutes at 100 °C. Once cooled to room temperature, the ampoule was opened, and the mixture was diluted with 12 mL diethyl ether followed by filtration *via* silica plug (6 cm depth and 2.5 cm diameter) with diethyl ether as eluent. The reaction vessel was rinsed with 3x12 mL diethyl ether and each washing was filtered through the silica plug as well. The organic phases were combined and concentrated *en vacuo* at 40 °C and p<100 mbar. The crude reaction mixture was then further purified *via* two hot recrystallisations (both 50 °C; first n-hexane, then 10:1 methanol:water), giving **3d** as a yellow solid after storing first at -20 °C, then 5 °C (87 mg, 0.34 mmol, 54%).

^{1}H NMR (400 MHz, $CDCl_3$, 298 K): δ = 8.70 (d, $^{3}J_{HH}$ = 8.7 Hz, 1H, Ar*H*), 8.35 (d, $^{3}J_{HH}$ = 7.5 Hz, 1H, Ar*H*), 8.19 (dd, $^{3}J_{HH}$ = 8.7 Hz, $^{4}J_{HH}$ = 2.9 Hz, 1H, Ar*H*), 7.65 (*pseudo*-t, $^{3}J_{HH}$ = 8.2 Hz, $^{3}J_{HH}$ = 8.2 Hz, 1H, Ar*H*),
7.61–7.55 (m, 1H, Ar*H*), 7.25 (d, $^{3}J_{HH}$ = 7.8 Hz, 1H, Ar*H*), 2.90 (s, 6H, N(C*H*$_3$)$_2$).

^{13}C{^{1}H} NMR (101 MHz, $CDCl_3$, 298 K): δ = 152.2, 133.4, 131.1, 129.9 (d, $^{3}J_{CF}$ = 6.4 Hz, C_{Ar}-$C_{Ar}SO_2F$), 129.7 (2C), 129.3 (d, $^{2}J_{CF}$ = 22.9 Hz, $C_{Ar}SO_2F$), 123.1, 118.5, 116.1, 45.9. *Note: One carbon C_{Ar} signal presumably covered by other C_{Ar} signal since ^{1}H-^{13}C-HMBC possessed overlapping cross peaks.*

^{19}F NMR (377 MHz, $CDCl_3$, 298 K): δ = +62.1 (s, 1F, RSO_2*F*).

Except one ambiguous ^{13}C signal, all spectroscopic data in agreement with reported data.[6]

2-Pyridinesulfonyl fluoride "PyFluor" (3e) – CAS# 878376-35-3

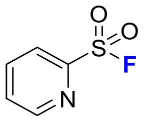

3e

In a dinitrogen containing glovebox, KHMDS (84 mg, 0.42 mmol) was dissolved in 1.2 mL dry THF and transferred into a J. Youngs NMR tube, after which a solution of sevoflurane (58 mg, 0.29 mmol) in 0.2 mL dry THF was added dropwise over one minute. An immediate colour change from a colourless solution to a black suspension was observed. The tube was sealed and inverted every 15 minutes to ensure proper mixing of the heterogenous mixture, while letting the components react for 90 minutes at 20 °C. After that, 2-pyridinesulfonyl chloride (23 mg, 0.13 mmol) in 0.4 mL dry THF was added. The NMR tube was sealed once again, transferred into a hot oil bath which was set to 100 °C, and the reaction mixture allowed to react for 100 minutes at 100 °C, while inverting the tube every 15 minutes to ensure proper mixing of the mixture. Once cooled to room temperature, the NMR tube was opened, and the content was poured into a vial. The NMR tube was rinsed with 2.4 mL diethyl ether and the ethereal solution was added to the reaction mixture. This diluted solution was then filtered through a silica plug (4 cm depth and 0.6 cm diameter) with diethyl ether as eluent. All glassware was rinsed with additional 2.4 mL diethyl ether as well as 2.4 mL of an 1:1 solution of diethyl ether:ethyl acetate, and both solutions were filtered through the silica plug was well. All organic phases were combined and concentrated *en vacuo* at 40 °C and p≥200 mbar. The crude mixture was then further purified *via* liquid chromatography (silica gel; 1:1 pentane:DCM as eluent), giving **3e** as a colourless oil (9 mg, 0.06 mmol, 46%; $R_f \approx 0.39$ (1:1 pentane:DCM)). *Note: The product is a volatile oil. Pressures lower than 200 mbar at 40 °C will diminish the yield. Drying at 50 °C at 450 mbar for 60 minutes proved effective following the purification.*

^{1}H NMR (400 MHz, CD_2Cl_2, 25 °C): δ = 8.84 (d, $^3J_{HH}$ = 4.6 Hz, 1H, $C_{sp2}H$), 8.13 (d, $^3J_{HH}$ = 7.9 Hz, 1H, $C_{sp2}H$), 8.07 (*pseudo*-t, $^3J_{HH}$ = 7.9 Hz, $^3J_{HH}$ = 7.8 Hz, 1H, $C_{sp2}H$), 7.73 (dd, $^3J_{HH}$ = 7.8 Hz, $^3J_{HH}$ = 4.6 Hz, 1H, Ar*H*).

^{13}C{^{1}H} NMR (101 MHz, CD_2Cl_2, 25 °C): δ = 151.51 (d, $^2J_{CF}$ = 29.9 Hz, $C_{sp2}SO_2F$), 151.48, 139.2, 129.8, 124.6.

^{19}F NMR (377 MHz, CD_2Cl_2, 25 °C): δ = +54.9 (s, 1F, RSO_2F).

All spectroscopic data in agreement with reported data.[6]

3-Pyridinesulfonyl fluoride (3f) – CAS# 1373307-61-9

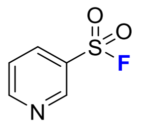

3f

In a dinitrogen containing glovebox, KHMDS (672 mg, 3.39 mmol) was dissolved in 12 mL dry THF and transferred to an ampoule containing a stirrer bar, after which a solution of hexafluoroisopropyl methyl ether (413 mg, 2.26 mmol) in 2 mL THF was added quickly. The ampoule was sealed and attached onto a Schlenk lin and placed into a water bath of 20 °C, where the reaction mixture stirred for 90 minutes. An immediate colour change from a colourless solution to a black suspension was observed. After that, pyridine-3-sulfonyl chloride (198 mg, 97% purity, 1.08 mmol) in 2 mL dry THF was added under dinitrogen overpressure dropwise over a minute. The ampoule was sealed once again, the water bath replaced with a hot oil bath which was already set to 100 °C, and the reaction mixture was allowed to stir for 100 minutes at 100 °C. Once cooled to room temperature, the ampoule was opened, and the mixture was diluted with 24 mL diethyl ether followed by filtration via silica plug (7 cm depth and 2.5 cm diameter) with diethyl ether as eluent. The reaction vessel was rinsed with 3x24 mL diethyl ether and each washing was filtered through the silica plug as well. The organic phases were combined and concentrated *en vacuo* at 40 °C and p≥100 mbar. The crude reaction mixture was then further purified *via* liquid chromatography (silica gel; DCM as eluent), giving **3f** as a colourless oil (117 mg, 0.73 mmol, 68%; $R_f \approx 0.47$ (DCM)).

^{1}H NMR (400 MHz, $CDCl_3$, 25 °C): δ = 9.19 (s, 1H, $C_{sp2}H$), 8.98 (d, $^{3}J_{HH}$ = 4.8 Hz, 1H, $C_{sp2}H$), 8.30–8.26 (m, 1H, $C_{sp2}H$), 7.60 (dd, $^{3}J_{HH}$ = 8.2 Hz, $^{3}J_{HH}$ = 4.8 Hz, 1H, $C_{sp2}H$).

$^{13}C\{^{1}H\}$ NMR (101 MHz, $CDCl_3$, 25 °C): δ = 156.1, 149.0, 136.1, 130.2 (d, $^{2}J_{CF}$ = 25.5 Hz, $C_{sp2}SO_2F$), 124.2.

^{19}F NMR (377 MHz, $CDCl_3$, 25 °C): δ = +67.9 (s, 1F, RSO_2F).

All spectroscopic data in agreement with reported data.[6]

Thiophene-2-sulfonyl fluoride (3g) – CAS# 382-99-0

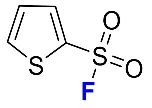

3g

In a dinitrogen containing glovebox, KHMDS (447 mg, 2.24 mmol) was dissolved in 8 mL dry THF and transferred to an ampoule containing a stirrer bar, after which a solution of 1,1,2,2-tetrafluoroethyl 2,2,2-trifluoroethyl ether (308 mg, 1.54 mmol) in 1.5 mL THF was added quickly. The ampoule was sealed and attached onto a Schlenk line and placed into a water bath of 20 °C, where the reaction mixture stirred for 90 minutes. An immediate colour change from a colourless solution to a black suspension was observed. After that, thiophene-2-sulfonyl chloride (138 mg, 97% purity, 0.73 mmol) in 3 mL dry THF was added under dinitrogen overpressure dropwise over a minute. The ampoule was sealed once again, the water bath replaced with a hot oil bath which was already set to 100 °C, and the reaction mixture was allowed to stir for 100 minutes at 100 °C. Once cooled to room temperature, the ampoule was opened, and the mixture was diluted with 18 mL diethyl ether followed by filtration via silica plug (5 cm depth and 2.5 cm diameter) with diethyl ether as eluent. The reaction vessel was rinsed with 3x18 mL diethyl ether and each washing was filtered through the silica plug as well. The organic phases were unified and concentrated *en vacuo* at 40 °C and p≥300 mbar for 15 minutes. The crude reaction mixture was then further purified *via* liquid chromatography (silica gel; 1:1 petroleum ether:DCM as eluent), giving **3g** as a pale yellow oil (117 mg, 0.73 mmol, 68%; $R_f \approx 0.57$ (*n*-hexane:DCM)). *Note: The product is a very volatile oil. Drying at 50 °C at 450 mbar for 60 minutes did not diminish amount of the product, but these conditions did not remove all the petroleum ether from the liquid chromatography elution.*

^{1}H NMR (400 MHz, $CDCl_3$, 25 °C): δ = 7.94–7.91 (m, 1H, $C_{sp2}H$), 7.88 (d, $^3J_{HH}$ = 5.0 Hz, 1H, $C_{sp2}H$), 7.24 (*pseudo*-t, $^3J_{HH}$ = 4.5 Hz, $^3J_{HH}$ = 4.4 Hz, 1H, $C_{sp2}H$).
^{13}C{^{1}H} NMR NMR (101 MHz, $CDCl_3$, 25 °C): δ = 137.0, 136.6, 131.7 (d, $^2J_{CF}$ = 30.1 Hz, $C_{sp2}SO_2F$), 128.3.
^{19}F NMR (377 MHz, $CDCl_3$, 25 °C): δ = +71.7 (s, 1F, RSO_2F).

All spectroscopic data of product in agreement with reported data.[7] *The spectrum also contained quantities of hydrocarbons species (^{1}H NMR :1.58–0.80 ppm; as well as ^{13}C[^{1}H] NMR: 41.5–11.5 ppm) which were used in the liquid chromatography for the isolation of the desired compound but could not be fully removed without further evaporation of the product.*

Methanesulfonyl fluoride (in situ; 3h) – CAS# 558-25-8

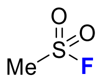

3h

In a dinitrogen containing glovebox, 2,2-di(phenyl)-1,1,1-trifluoroethane (17.1 mg, 0.07 mmol) was dissolved in 0.4 ml C_6D_6 and transferred to J Young NMR tube. KHMDS (16.2 mg, 0.08 mmol) was dissolved in 0.6 ml C_6D_6 and added to the solution in the J Young NMR tube. The reaction mixture was left for for 75 minutes at 20 °C, then heated to 100 °C for 45 minutes. A colour change from colourless solution to a dark viscous solution was observed. To this reaction mixture, methanesulfonyl chloride (5 µL, 0.06 mmol) in 0.3 mL C_6D_6 and 1,2-difluorobenzene (0.10 mmol) were added. The reaction mixture was then heated to 100 °C for 1 hour. Yields were determined by ^{19}F NMR spectroscopy using integration compared to 1,2-difluorobenzene as an internal standard. **3h** was observed (65% *in situ* yield) but due to low boiling point this substrate was not isolated, characterisation was performed from the crude reaction mixture.

^{1}H NMR (400 MHz, C_6D_6, 25 °C): δ = 1.80 (s, 3H, CH_3).

^{13}C{^{1}H} NMR (101 MHz, C_6D_6, 25 °C): δ = 35.9 (d, $^{2}J_{CF}$ = 22.0 Hz, *C*SO_2F, confirmed *via* ^{1}H-^{13}C-HSQC).

^{19}F NMR (377 MHz, C_6D_6, 25 °C): δ = +59.7 (s, 1F, RSO_2*F*).

NMR data consistent with literature, shifted as a different solvent was used.[8]

11.2. sp^{2}C–F bond formation: Synthesis and characterisation of acyl fluorides

2-naphthoyl fluoride (3i) – CAS# 37827-83-1

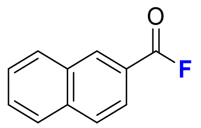

3i

In a dinitrogen containing glovebox, KHMDS (596 mg, 2.99 mmol) was dissolved in 20 mL dry THF and transferred to an ampoule containing a stirrer bar, after which a solution of hexafluoroisopropyl methyl ether (364 mg, 2.00 mmol) in 2 mL THF was added quickly. The ampoule was sealed and left in the glovebox to stir at 20 °C for 90 minutes. An immediate colour change from a colourless solution to a black suspension was observed. After that, 2-naphthoyl chloride (191 mg, 97% purity, 0.97 mmol) in 3 mL dry THF was added dropwise over a minute. The ampoule was sealed once again, removed from the glovebox and placed in a hot oil bath which was already set to 100 °C, and the reaction mixture was allowed to stir for 100 minutes at 100 °C. Once cooled to room temperature, the ampoule was opened, and the mixture was diluted with 25 mL diethyl ether followed by filtration via silica plug (6 cm depth and 2.5 cm diameter) with diethyl ether as eluent. The reaction vessel was rinsed with 3x25 mL diethyl ether and each washing was filtered through the silica plug as well. The organic phases were combined and concentrated *en vacuo* at 40 °C and p<100 mbar. Chloroform was added to the concentrated crude reaction mixture and a colourless solid precipitated. The resulting suspension was filtered through another silica plug and the glassware and silica plug rinsed with chloroform. The organic filtrate was then once again concentrated *en vacuo* at 40 °C and p<100 mbar. The resulting crude was further purified *via* liquid chromatography (silica gel; 95:5 petroleum ether:ethyl acetate as eluent), giving **3i** as a colourless crystalline solid (150 mg, 0.86 mmol, 89%; $R_f \approx 0.57$ (95:5 petroleum ether:ethyl acetate)).

^{1}H NMR (400 MHz, $CDCl_3$, 25 °C): δ = 8.63 (s, 1H, Ar*H*), 8.02–7.90 (m, 4H, Ar*H*), 7.72–7.65 (m, 1H, Ar*H*), 7.64–7.58 (m, 1H, Ar*H*).

^{13}C{^{1}H} NMR (101 MHz, $CDCl_3$, 25 °C): δ = 157.8 (d, $^1J_{CF}$ = 343.6 Hz, *C*(O)F), 136.6, 134.1 (d, $^3J_{CF}$ = 2.7 Hz, C_{Ar}-C_{Ar}C(O)F), 132.4, 129.82, 129.77, 129.2, 128.1, 127.5, 125.7 (d, $^3J_{CF}$ = 3.8 Hz, C_{Ar}-C_{Ar}C(O)F), 122.1 (d, $^2J_{CF}$ = 60.7 Hz, C_{Ar}C(O)F).

^{19}F NMR (377 MHz, $CDCl_3$, 25 °C): δ = +18.1 (s, 1F, RC(O)*F*).

All spectroscopic data in agreement with reported data.[9]

2,4,6-Trimethylbenzoyl fluoride (3j) – CAS# 826-66-4

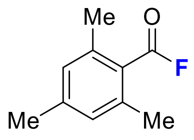

3j

In a dinitrogen containing glovebox, KHMDS (202 mg, 1.01 mmol) was dissolved in 8 ml THF and transferred to a 20 ml ampoule. The ampoule was degassed *via* freeze-pump-thaw technique before HFC-134a (1.4 bar, 12 ml, 0.678 mmol) was added, the vessel was shaken initially then allowed to vigorously stir for 70 minutes at 25 °C. A colour change from colourless solution to a black suspension was observed. To the suspension, 2,4,6-trimethylbenzoyl chloride (61.1 mg, 0.335 mmol) was added. The reaction mixture was then heated to 100 °C and allowed to stir for 1 hour. Once cooled to room temperature, the solution was concentrated by rotary evaporation and the crude reaction mixture was dry loaded onto a silica column for purification, eluted with 5-10% ethyl acetate in *n*-hexane. **3j** was obtained as a colourless solid (39.3 mg, 0.236 mmol, 71%).

^{1}H NMR (400 MHz, $CDCl_3$, 25 °C) δ = 6.93 (s, 1H, Ar*H*), 2.45 (d, $^5J_{HF}$ = 3.4 Hz, 6H, *o*-CH_3), 2.32 (s, 3H, *m*-CH_3).

^{13}C{^{1}H} NMR (101 MHz, $CDCl_3$, 25 °C) δ = 158.7 (d, $^1J_{CF}$ = 352.8 Hz, *C*(O)F), 142.9, 139.6, 129.8, 123.6 (d, $^2J_{CF}$ = 54.1 Hz, C^{iv}_{Ar}), 21.42, 21.35 (d, $^4J_{CF}$ = 3.7 Hz, *o*-CH_3).

^{19}F NMR (377 MHz, $CDCl_3$, 25 °C) δ = 52.5 (s, 1F, C(O)*F*).

All spectroscopic data in agreement with reported data.[10]

2,6-Difluorobenzoyl fluoride (3k) – CAS# 13656-41-2

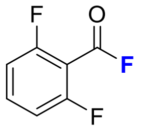

3k

In a dinitrogen containing glovebox, 1,1,1-trifluoro-2,2-bis(2,4,6-trifluorophenyl)ethane (685 mg, 1.99 mmol) and KO*t*Bu (89 mg, 2.08 mmol) in 4.7 ml of diethyl ether were transferred to an ampoule. The ampoule was sealed and transferred onto a Schlenk line and placed in a hot oil bath which was already set to 65 °C. The reaction mixture was allowed to stir for 90 minutes at this temperature. The reaction mixture became heterogenous as the reaction proceeded. After that, the ampoule was removed from the oil bath and stirred for 15 minutes to cool down, 2,6-difluorobenzoyl chloride (214 mg, 98% purity, 1.19 mmol) in 0.5 mL dry diethyl ether was added under nitrogen overpressure dropwise over a minute. The ampoule was sealed once again, and placed again in a hot oil bath which was already set to 65 °C, and the reaction mixture was allowed to stir for 100 minutes at 65 °C. Once cooled to room temperature, the ampoule was opened, and the mixture was filtered *via* silica plug (6 cm depth and 2.5 cm diameter) with diethyl ether as eluent. The reaction vessel was rinsed with 3x5 mL diethyl ether and each washing was filtered through the silica plug as well. The organic phases were combined and concentrated *en vacuo* at 30 °C at 450 mbar for 30 minutes and 350 mbar for 15 minutes. The resulting crude mixture was further purified *via* liquid chromatography (silica gel; 3:1 pentane:diethyl ether as eluents) yielding the desired product **3k**, which was obtained white solid (65 mg, 0.41 mmol, 34%). *Note: This product is a very volatile solid and labile against higher loadings of base. It also showed decomposition reactivity in the presence of KHMDS. Drying at 30 °C at 450 mbar for 50 minutes and 350 mbar for 30 minutes did not diminish amount of the product. The product is also unstable on silica gel when using hydrocarbon:DCM eluents.*

^{1}H NMR (400 MHz, CD_2Cl_2, 25 °C): δ = 7.67 (tt, $^{3}J_{HH}$ = 8.5 Hz, $^{4}J_{HF}$ = 6.1 Hz, 1H, *p*-Ar*H*), 7.08 (*pseudo*-t, $^{3}J_{HH}$ = 9.2 Hz, $^{3}J_{HF}$ = 9.1 Hz, 2H, *m*-Ar*H*).

^{13}C{^{1}H} NMR (101 MHz, CD_2Cl_2, 25 °C): δ = 162.9 (dd, $^{1}J_{CF}$ = 263.9 Hz, $^{3}J_{CF}$ = 4.0 Hz, C_{Ar}F), 151.2 (d, $^{1}J_{CF}$ = 343.8 Hz, *C*(O)F), 137.4 (t, $^{3}J_{CF}$ = 11.1 Hz, C_{Ar}H), 113.3 (t, $^{2}J_{CF}$ = 22.0 Hz, C_{Ar}H), 104.9 (dt, $^{2}J_{CF}$ = 62.9 Hz, $^{2}J_{CF}$ = 14.4 Hz, C_{Ar}),

^{19}F NMR (377 MHz, CD_2Cl_2, 25 °C): δ = +46.5 (t, $^{4}J_{FF}$ = 39.4 Hz, 1F, RC(O)*F*), –106.0 (ddd, $^{4}J_{FF}$ = 39.4 Hz, $^{3}J_{FH}$ = 9.1 Hz, $^{4}J_{FH}$ = 6.1 Hz, 2 *o*-Ar*F*).

All spectroscopic data in agreement with reported data.[11]

4-Bromobenzoyl fluoride (3l) – CAS# 72398-40-4

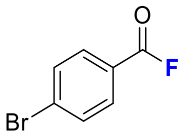

3l

In a dinitrogen containing glovebox, KO^{t}Bu (93.0 mg, 0.829 mmol) was dissolved in 4 ml THF and transferred to a 20 ml ampoule. The reaction mixture was degassed *via* freeze-pump-thaw technique before HFC-134a (1.4 bar, 16 ml, 0.904 mmol) was added, the vessel was shaken then the reaction mixture allowed to vigorously stir for 1 hour at 60 °C. To the reaction mixture, 4-bromobenzoyl chloride (88.6 mg, 0.404 mmol) was added. The reaction mixture was then heated to 100 °C and allowed to stir for 1 hour. Once cooled to room temperature, the solution was concentrated by rotary evaporation and the crude mixture was dry loaded onto a silica column for purification, eluted with 25% diethyl ether in *n*-pentane. **3l** was obtained as a colourless solid (60.1 mg, 0.296 mmol, 73%).

^{1}H NMR (400 MHz, C_6D_6, 25 °C): δ = 7.22 (d, $^3J_{HH}$ = 8.2 Hz, 2H, Ar*H*), 6.90 (d, $^3J_{HH}$ = 8.2 Hz, 2H, Ar*H*).

^{13}C{^{1}H} NMR (101 MHz, C_6D_6, 25 °C): δ = 156.6 (d, $^1J_{CF}$ = 343.4 Hz, *C*(O)F), 132.6 (d, $^3J_{CF}$ = 3.9 Hz, C_{Ar}H), 132.3, 130.5, 123.9 (d, $^2J_{CF}$ = 63.7 Hz, C^{iv}_{Ar}).

^{19}F NMR (377 MHz, C_6D_6, 25 °C): δ = 18.3 (s, 1F, C(O)*F*).

FT/IR (ν_{max} (thin film) cm^{-1}): 1803 (s, C=O stretch), 1589, 1399, 1247, 999, 1024, 740, 675.

NMR data consistent with literature, shifted as a different solvent was used.[12]

IR data consistent with literature.[13]

Cinnamoyl fluoride (3m) – CAS# 38986-89-9

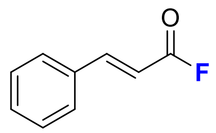

3m

In a dinitrogen containing glovebox, 1,1,2,2-tetrafluoroethyl 2,2,2-trifluoroethyl ether (370.0 mg, 1.85 mmol) was dissolved in 3 mL dry THF and transferred to an ampoule containing a stirrer bar, after which a solution of KHMDS (339 mg, 1.75 mmol) in 14 mL THF was added quickly. The ampoule was sealed and left in the glovebox to stir at 20 °C for 75 minutes. An immediate colour change from a colourless solution to a black suspension was observed. After that, 2-cinnamoyl chloride (138 mg, 95% purity, 0.79 mmol) in 3 mL dry THF was added dropwise over a minute. The ampoule was sealed once again, removed from the glovebox and placed in a hot oil bath set to 100 °C. The reaction mixture was heated for 90 minutes at 100 °C. Once cooled to room temperature, the ampoule was opened, and the mixture was diluted with 20 mL diethyl ether followed by filtration via silica plug (6 cm depth and 2.5 cm diameter) with diethyl ether as eluent. The reaction vessel was rinsed with 3x20 mL diethyl ether and each washing was filtered through the silica plug as well. The organic phases were combined and concentrated *en vacuo* at 40 °C and p≥100 mbar. The resulting crude product was further purified *via* liquid chromatography (silica gel; 100:0 to 20:1 pentane:DCM gradients as eluents), giving **3m** as a colourless oil (77 mg, 0.51 mmol, 65%; $R_f \approx 0.57$ at 20:1 hexane:DCM).

^{1}H NMR (400 MHz, $CDCl_3$, 25 °C): δ = 7.84 (d, $^{3}J_{HH}$ = 15.8 Hz, 1H, $C_{olefin}H$), 7.60–7.53 (m, 2H, Ar*H*), 7.51–7.40 (m, 3H, Ar*H*), 6.37 (dd, $^{3}J_{HH}$ = 15.8 Hz, $^{3}J_{HF}$ = 7.0 Hz, 1H, $C_{olefin}H$).

^{13}C{^{1}H} NMR (101 MHz, $CDCl_3$, 25 °C): δ = 157.2 (d, $^{1}J_{CF}$ = 338.5 Hz, *C*(O)F), 151.5 (d, $^{3}J_{CF}$ = 5.8 Hz, C_{olefin}), 133.2, 132.0, 129.3, 128.8, 112.2 (d, $^{2}J_{CF}$ = 67.1 Hz, C_{olefin}),

^{19}F NMR (377 MHz, $CDCl_3$, 25 °C): δ = +25.6 (d, $^{3}J_{FH}$ = 7.0 Hz, 1F, RC(O)*F*).

All spectroscopic data in agreement with reported data.[14]

Pivaloyl fluoride (3n) – CAS# 1478-62-2

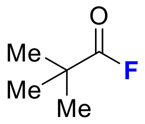

3n

In a dinitrogen containing glovebox, KHMDS (26.5 mg, 0.133 mmol) was dissolved in 0.6 ml C_6D_6 and transferred to J Young NMR tube. The reaction mixture was degassed *via* freeze-pump-thaw technique before HFC-134a (1.4 bar, 2.2 ml, 0.124 mmol) was added, the tube was inverted 3 times then allowed to stand for 70 minutes at 25 °C. A colour change from colourless solution to a black suspension was observed. To the suspension, pivaloyl chloride (5.3 mg, 0.044 mmol) and 1,2-difluorobenzene (0.100 mmol) were added. The reaction mixture was then heated to 100 °C for 1 hour. Yields were determined using integration compared to 1,2-difluorobenzene as an internal standard. The desired product **3n** was observed (66% *in situ* yield), but due to low boiling point this substrate was not isolated, characterisation was performed from the crude reaction mixture. NMR data matches literature, chemical shifts are shifted due to solvent used.[15] ^{13}C NMR signals were assigned *via* 2D NMR (see Supplementary Figure 69).

^{1}H NMR (400 MHz, C_6D_6, 25 °C): δ = 0.86 (s, 9H, $(CH_3)_3$).

^{13}C{^{1}H} NMR (101 MHz, C_6D_6, 25 °C): δ = 167.6 (d, $^{1}J_{CF}$ = 390.7 Hz, *C*(O)F), 38.6, 25.9.

^{19}F NMR (377 MHz, C_6D_6, 25 °C): δ = 23.8 (s, 1F, C(O)*F*).

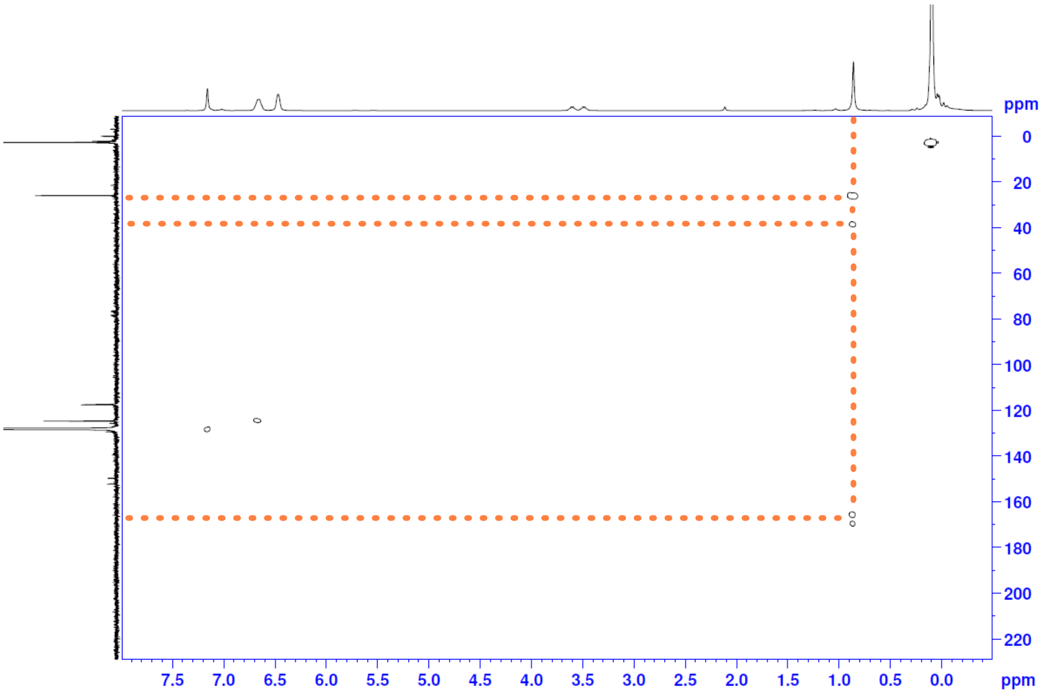

Supplementary Figure 69: *In situ* ^{1}H-^{13}C-HMBC spectrum of the reaction mixture containing **3n** (C_6D_6, 25 °C).

4-Phenylbutanoyl fluoride (3o) – CAS# 82561-66-8

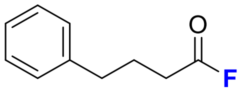

3o

In a dinitrogen containing glovebox, 3,3,3-trifluoropropanenitrile (77.4 mg, 0.71 mmol) was diluted with 0.2 mL THF and transferred into a J. Youngs NMR tube, after which a solution of KO*t*Bu (80.0 mg, 0.72 mmol) in 1.4 mL of THF was added. The NMR tube was sealed and placed in a hot oil bath at 80 °C. The reaction mixture was allowed to react for 90 minutes at this temperature, while inverting the sample each 15 minutes to promote mixing. After cooling to room temperature, the J. Youngs NMR tube was removed from the oil bath and transferred into a dinitrogen containing glovebox, where 4-phenylbutanoyl chloride (73.1 mg, 97% purity, 0.39 mmol) in 0.2 mL dry THF was added. The NMR tube was sealed and placed in a hot oil bath which was already set to 100 °C, and the reaction mixture was allowed to react for 100 minutes at this temperature, while inverting the sample each 20 minutes. Once cooled to room temperature, the NMR tube was opened, and the mixture was diluted with 3 mL diethyl ether, followed by filtration *via* silica plug (4 cm depth and 2.5 cm diameter) with diethyl ether as eluent. The reaction vessel was rinsed with 3x5 mL diethyl ether and each washing was filtered through the silica plug as well. The organic phases were combined and concentrated *in vacuo* at 40 °C at p≥200 mbar. The resulting crude mixture was further purified *via* liquid chromatography (silica gel; 5:1 pentane:diethyl ether as eluents), eluting the desired product **3o** ($R_f \approx 0.41$; 5:1 petroleum ether:diethyl ether) as a colourless oil (50.0 mg, 0.30 mmol, 78%).

^{1}H NMR (400 MHz, CD_2Cl_2, 25 °C): δ = 7.34–7.28 (m, 2H, Ar*H*), 7.25–7.18 (m, 3H, Ar*H*, Ar*H*), 2.71 (t, $^3J_{HH}$ = 7.6 Hz, 2H, -C*H*$_2$-), 2.53 (t, $^3J_{HH}$ = 7.4 Hz, 2H, -C*H*$_2$-), 2.00 (tt, $^3J_{HH}$ = 7.6 Hz, $^3J_{HH}$ = 7.4 Hz, 2H, -C*H*$_2$-),

^{13}C{^{1}H} NMR (101 MHz, CD_2Cl_2, 25 °C): δ = 163.9 (d, $^1J_{CF}$ = 360.0 Hz, *C*(O)F), 141.1, 128.9, 128.8, 126.6, 34.9, 31.7 (d, $^2J_{CF}$ = 50.5 Hz, C_{alkyl}C(O)F), 25.9.

^{19}F NMR (377 MHz, CD_2Cl_2, 25 °C): δ = +44.7 (s, 1F, RC(O)*F*).

All spectroscopic data in agreement with data reported in $CDCl_3$ as solvent.[16]

11.3. sp^3C–F and sp^2C–F bond formation: Synthesis and characterisation of alkyl and aryl fluorides

Trityl fluoride (3p) – CAS# 427-36-1

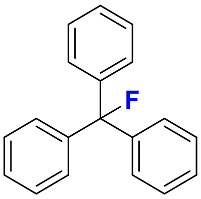

3p

In a dinitrogen containing glovebox, KHMDS (201.0 mg, 1.01 mmol) was dissolved in 8 mL THF and transferred to a 20 mL ampoule. The reaction mixture degassed *via* freeze-pump-thaw technique before HFC-134a (1.4 bar, 12 mL, 0.678 mmol) was added, the vessel was shaken then the reaction mixture allowed to vigorously stir for 70 minutes at 25 °C. A colour change from colourless solution to a black suspension was observed. To the suspension, bromotriphenylmethane (107.8 mg, 0.33 mmol) was added. The reaction mixture was then heated to 100 °C and allowed to stir for 1 hour. Once cooled to room temperature, the solution was filtered through a celite plug (2.5 cm depth, 5 cm diameter) and washed with diethyl ether (3x15 mL). The filtrate was concentrated by rotary evaporation and purified by recrystallisation using hot n-hexane (3 mL) and washed with cold n-pentane. Trityl fluoride (**3p**) was obtained as a pale orange solid (63.2 mg, 0.24 mmol, 72%).

^{1}H NMR (400 MHz, CD_2Cl_2, 25 °C): δ = 7.38–7.32 (m overlapping, 9H, ArH), 7.25–7.20 (m overlapping, 6H, ArH).

^{13}C{^{1}H} NMR (101 MHz, CD_3CN, 25 °C): δ = 144.2 (d, $^2J_{CF}$ = 24.1 Hz, C^{iV}_{Ar}), 129.3 (d, $^4J_{CF}$ = 1.6 Hz, C^{iV}_{Ar}), 129.1, 128.5 (d, $^3J_{CF}$ = 6.2 Hz, C_{Ar}H), 102.2 (d, $^1J_{CF}$ = 173.6 Hz, C^{iV}F).

^{19}F NMR (377 MHz, CD_2Cl_2, 25 °C): δ = −126.5 (s, 1F, CF).

All spectroscopic data in agreement with reported data.[17]

3-Chloro-4-fluoronitrobenzene (3q) – CAS# 350-30-1

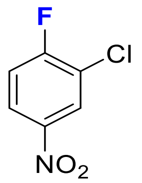

3q

In a dinitrogen containing glovebox, KHMDS (295.0 mg, 1.48 mmol) was dissolved in 3 mL THF and transferred to a 20 mL ampoule containing a stirrer bar. The solution was stirred vigorously and a solution of 1,1,2,2-tetrafluoroethyl 2,2,2-trifluoroethyl ether (235.0 mg, 1.18 mmol) in 0.4 mL THF was added, and the reaction mixture was allowed to stir for 75 minutes at 25 °C. A colour change of the solution from colourless to black was observed immediately, and the reaction mixture became more heterogenous with time. The reaction vessel was then transferred onto a Schlenk line and the volatiles removed under vacuum to obtain a black solid. Under dinitrogen, a solution of 3,4-dichloronitrobenzene (119.0 mg, 97% purity, 0.60 mmol) in 5 mL of DMSO was added to the vessel. This reaction mixture was then heated to 130 °C for 20 h. Once cooled down to room temperature, the reaction mixture was diluted with 10 mL diethyl ether and the biphasic mixture was filtered through a silica plug (6 cm depth, 2.5 cm diameter). The vessel was washed with diethyl ether (3x10 mL) and filtered through the same silica plug. The two phases were separated and the DMSO phase was diluted with 20 mL water and 20 mL brine. The now aqueous phase was washed with diethyl ether (3x10 mL) and the organic fractions were combined and washed with 20 mL water and 20 mL brine. The ethereal solution was concentrated *in vacuo* and the crude product was purified by flash column chromatography on silica gel using pentane to 95:5 pentane:diethyl ether as eluent, collecting the fastest band. This fraction was then purified *via* preparative thin layer chromatography (3:1 cyclohexane:diethyl ether), collecting the large eluting band (R_f ≈ 0.70; 2:1 cyclohexane:diethyl ether). 3-Chloro-4-fluoronitrobenzene **3q** was obtained as a colourless solid (48.0 mg, 0.28 mmol, 47%).

^{1}H NMR (400 MHz, $CDCl_3$, 25 °C): δ = 8.35 (dd, $^{4}J_{HF}$ = 6.4 Hz, $^{4}J_{HH}$ = 2.7 Hz, 1H, Ar*H*), 8.18 (ddd, $^{3}J_{HH}$ = 9.2 Hz, $^{4}J_{HF}$ = 4.1 Hz, $^{4}J_{HH}$ = 2.7 Hz, 1H, Ar*H*), 7.32 (dd, $^{3}J_{HH}$ = 9.2 Hz, $^{3}J_{HF}$ = 8.1 Hz, 1H, Ar*H*).
^{13}C{^{1}H} NMR (101 MHz, $CDCl_3$, 25 °C): δ = 162.0 (d, $^{1}J_{CF}$ = 260.2 Hz, *C*F), 144.4, 126.9, 124.2 (d, $^{3}J_{CF}$ = 9.0 Hz, C_{Ar}), 122.7 (d, $^{2}J_{CF}$ = 19.8 Hz, C_{Ar}), 117.3 (d, $^{2}J_{CF}$ = 23.2 Hz, C_{Ar}).
^{19}F NMR (377 MHz, $CDCl_3$, 25 °C): δ = -103.9 (ddd, $^{3}J_{FH}$ = 8.1 Hz, $^{4}J_{FH}$ = 6.4 Hz, $^{4}J_{FH}$ = 4.1 Hz, 1F, Ar*F*).
Beside some deviations in coupling patterns, spectroscopic data in agreement with reported data.[18]

2-Fluoro-5-nitrobenzonitrile (3r) – CAS# 17417-09-3

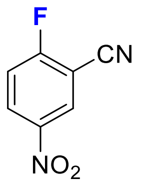

3r

In a dinitrogen containing glovebox, KHMDS (132.0 mg, 0.66 mmol) was dissolved in 5 mL THF and transferred to a 20 mL ampoule. The reaction mixture was degassed *via* freeze-pump-thaw technique before HFC-134a (1.2 bar, 15 mL, 0.73 mmol) was added, and the vessel was gently shaken. The reaction mixture was then allowed to vigorously stir for 60 minutes at 25 °C. A colour change from the colourless solution to a black solution was observed. The volatiles were removed under vacuum to obtain a black solid. 2-Chloro-5-nitrobenzonitrile (60.0 mg, 97% purity, 0.32 mmol) was dissolved in 3 mL of DMSO and added to the vessel under dinitrogen. The vessel was sealed, and reaction mixture was transferred into an oil bath, heated to 100 °C for 1 h. Once cooled down to room temperature, the solution was filtered through a silica plug (7 cm depth, 1 cm diameter). The reaction vessel was washed with ethyl acetate (4x5 mL) and the washings were filtered through the same silica plug. The combined organic layers were then washed with 3x10 mL brine and concentrated *in vacuo*. The crude product was further purified by flash column chromatography using a pentane to 9:1 pentane:ethyl acetate gradient as eluent (R_f ≈ 0.26; 9:1 pentane:ethyl acetate). 2-Fluoro-5-nitrobenzonitrile **3r** was obtained as a light yellow solid (40.3 mg, 0.24 mmol, 76%).

^{1}H NMR (400 MHz, $CDCl_3$, 25 °C): δ = 8.60–8.49 (m, 2H, Ar*H*, Ar*H*), 7.45 (dd, $^3J_{HH}$ = 9.5 Hz, $^3J_{HF}$ = 8.1 Hz, 1H, Ar*H*).

^{13}C{^{1}H} NMR (101 MHz, $CDCl_3$, 25 °C): δ = 166.3 (d, $^1J_{CF}$ = 270.1 Hz, *C*F), 144.3, 130.6 (d, $^3J_{CF}$ = 10.2 Hz, C_{Ar}), 129.7, 118.0 (d, $^2J_{CF}$ = 21.9 Hz, C_{Ar}), 111.8, 103.3 (d, $^2J_{CF}$ = 17.9 Hz, C_{Ar}).

^{19}F{^{1}H} NMR (377 MHz, $CDCl_3$, 25 °C): δ = -95.8 (s, 1F, C*F*).

All spectroscopic data in agreement with reported data.[18]

2,4-Dinitrofluorobenzene (3s) – CAS# 70-34-8

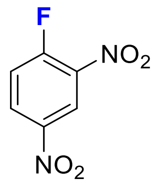

3s

In a dinitrogen containing glovebox, KHMDS (134.0 mg, 0.67 mmol) was dissolved in 5 mL THF and transferred to a 20 mL ampoule. The reaction mixture was degassed *via* freeze-pump-thaw technique before HFC-134a (1.2 bar, 15 mL, 0.727 mmol) was added and the reaction vessel was shaken. The reaction mixture was then allowed to vigorously stir for 60 minutes at 25 °C. A colour change in the solution from colourless to a black was observed. The volatiles were removed under reduced pressure to obtain a black solid. 1-Chloro-2,4-dinitrobenzene (66.7 mg, 99% purity, 0.33 mmol) was dissolved in 3 mL of DMSO and added to the solid under dinitrogen. The vessel was sealed, and reaction mixture was transferred into an oil bath, heated to 100 °C for 30 minutes. Once cooled down to room temperature, the reaction mixture was filtered through a silica plug (7 cm depth, 1 cm diameter). The reaction vessel was washed with ethyl acetate (4x5 mL) and the washings were filtered through the same silica plug. The combined organic layers were then washed with 3x10 mL brine and the organic fraction was concentrated by rotary evaporation. The crude product was first purified by flash column chromatography over silica gel using a pentane to 9:1 pentane:ethyl acetate gradient as eluent, followed by preparative thin later chromatography using 9:1 pentane:ethyl acetate as eluent ($R_f \approx 0.62$; 9:1 pentane:ethyl acetate). 1-Fluoro-2,4-dinitrobenzene **3s** was obtained as a yellow oil (33.6 mg, 0.18 mmol, 58%).

^{1}H NMR (400 MHz, CD_2Cl_2, 25 °C): δ = 8.96 (dd, $^{4}J_{HF}$ = 6.4 Hz, $^{4}J_{HH}$ = 2.7 Hz, 1H, Ar*H*), 8.57–8.51 (m, 1H, Ar*H*), 7.56 (*pseudo*-t, $^{3}J_{HF}$ = 9.2 Hz, $^{3}J_{HH}$ = 9.2 Hz, 1H, Ar*H*).

^{13}C{^{1}H} NMR (101 MHz, CD_2Cl_2, 25 °C): δ = 159.2 (d, $^{1}J_{CF}$ = 274.6 Hz, *C*F), 144.0, 137.5, 130.9 (d, $^{2}J_{CF}$ = 10.8 Hz, C_{Ar}), 122.8, 120.4 (d, $^{2}J_{CF}$ =23.0 Hz).

^{19}F{^{1}H} NMR (377 MHz, CD_2Cl_2, 25 °C): δ = -107.2 (s, 1F, C*F*)

All spectroscopic data with slight shift compared to $CDCl_3$ due to different solvent choice, but otherwise in agreement with reported data.[18]

6-Fluoronicotinonitrile (3t) – CAS# 3939-12-6

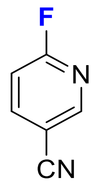

3t

In a dinitrogen containing glovebox, KHMDS (572.0 mg, 2.86 mmol) was dissolved in 10 mL THF and transferred to a 100 mL ampoule containing a stirrer bar. The solution was stirred vigorously and a solution of 1,1,2,2-tetrafluoroethyl 2,2,2-trifluoroethyl ether (590.0 mg, 2.95 mmol) in 8 mL THF was added, and the reaction mixture was allowed to stir vigorously for 75 minutes at 25 °C. A colour change of the solution from colourless to black was observed immediately, and the reaction mixture became more heterogenous with time. The reaction vessel was then transferred onto a Schlenk line and the volatiles removed under vacuum to obtain a black solid. Under dinitrogen, a solution of 6-chloronicotinonitrile (156.1 mg, 98% purity, 1.10 mmol) in 12 mL of DMSO was added to the vessel. The reaction mixture was heated to 130 °C for 19 hours. Once cooled down to room temperature, the reaction mixture was filtered through a silica plug (7 cm depth, 2.5 cm diameter). The vessel was washed with ethyl acetate (3x30 mL) and filtered through the same silica plug. The organic layers were combined and washed with 30 mL of brine. The aqueous layer was then back-extracted three times with ethyl acetate (3x15 mL). The organic fractions were united and dried over $MgSO_4$. The solution was concentrated *in vacuo* and the crude product was purified by flash column chromatography on silica gel using a 10:1 to 4:1 pentane:ethyl acetate gradient. 6-Fluoronicotinonitrile was obtained as a colourless solid (62.0 mg, 0.51 mmol, 46%; $R_f \approx 0.40$, 4:1 hexane:ethyl acetate).

^{1}H NMR (400 MHz, $CDCl_3$, 25 °C): δ = 8.57 (s, 1H, Ar*H*), 8.12–8.05 (m, 1H, Ar*H*), 7.12–7.07 (m, 1H, Ar*H*),

^{13}C{^{1}H} NMR (101 MHz, $CDCl_3$, 25 °C): δ = 165.0 (d, $^1J_{CF}$ = 249.1 Hz, C_{Ar}F), 152.3 (d, $^3J_{CF}$ = 16.8 Hz, C_{Ar}),-144.5 (d, $^3J_{CF}$ = 9.2 Hz, C_{Ar}), 115.7, 110.9 (d, $^2J_{CF}$ = 38.0 Hz, C_{Ar}), 108.1 (d, $^4J_{CF}$ = 4.5 Hz, C_{Ar}).

^{19}F NMR (377 MHz, $CDCl_3$, 25 °C): δ = -57.5 (s, 1F, C*F*).

Spectra displayed poor resolutions in ^{1}H and ^{19}F, so not all couplings were observed. However, the signal positions, integrals and ^{13}C data were all in agreement with reported data.[6]

11.4. Si–F bond formation: Synthesis and characterisation of products

Di-tert-butyldifluorosilane (3u) – CAS# 558-63-4

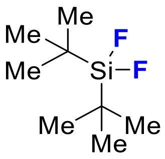

3u

In a dinitrogen containing glovebox, KHMDS (20.6 mg, 0.10 mmol) was dissolved in 0.5 mL C_6D_6 and transferred to J Young NMR tube. The reaction mixture was degassed *via* freeze-pump-thaw technique before HFC-134a (1.4 bar, 2.3 mL, 0.13 mmol) was added, the tube was inverted 3 times then allowed to stand for 70 minutes at 25 °C. A colour change from colourless solution to a black suspension was observed. To the suspension, di-*tert*-butyldichlorosilane (4.3 mg, 0.02 mmol) and 1,2-difluorobenzene (1.97 µL, 0.02 mmol) was added. The reaction mixture was then heated to 100 °C for 22 hours. Yields were determined by ^{19}F NMR spectroscopy using integration compared to 1,2-difluorobenzene as an internal standard. The desired product (**3u**) was observed (58% *in situ* yield), due to its low boiling point this substrate was not isolated, characterisation was performed from the crude reaction mixture. ^{29}Si NMR signals were assigned *via* 2D NMR (see Supplementary Figure 70).

^{1}H NMR (400 MHz, C_6D_6, 25 °C) δ = 0.98 (s, 18H, (C*H*$_3$)$_3$).

^{19}F NMR (377 MHz, C_6D_6, 25 °C) δ = −158.2 (s, 2F, Si*F*$_2$).

^{29}Si NMR (80 MHz, C_6D_6, 25 °C) δ = −7.9 (t, *Si*F$_2$; confirmed via ^{1}H-^{29}Si-HMBC, see below).

All spectroscopic data in agreement with reported data.[19]

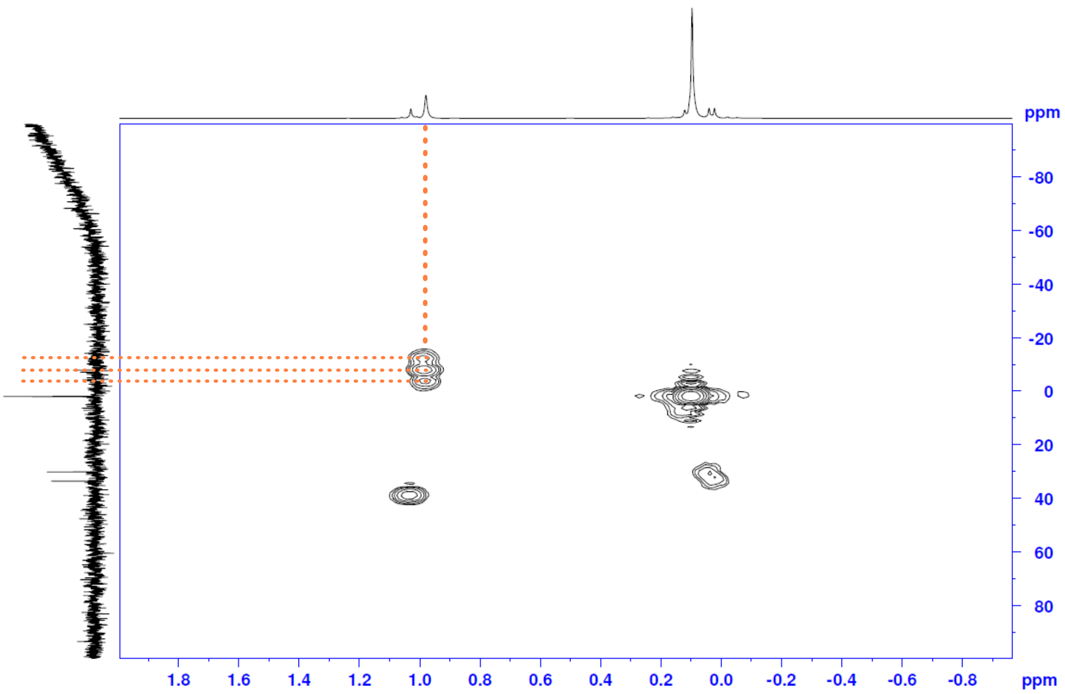

Supplementary Figure 70: ^{1}H-^{29}Si-HMBC of the reaction mixture containing **3u** (C_6D_6, 25 °C).

Trimethylsilyl fluoride (3v) – CAS# 420-56-4

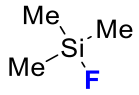

3v

In a dinitrogen containing glovebox, KHMDS (21.7 mg, 0.109 mmol) was dissolved in 1 mL C_6D_6 and transferred to J Young NMR tube. The reaction mixture was degassed *via* freeze-pump-thaw technique before HFC-134a (1.4 bar, 1.8 mL, 0.20 mmol) was added, the tube was inverted 3 times then allowed to stand for 70 minutes at 25 °C. A colour change from colourless solution to a black suspension was observed. To the suspension, chlorotrimethylsilane (5.08 µL, 0.04 mmol) and 1,2-difluorobenzene (3.93 µL, 0.04 mmol) was added. The reaction mixture was then heated to 100 °C for 1 hour. Yields were determined by ^{19}F NMR spectroscopy using integration compared to 1,2-difluorobenzene as an internal standard. To determine an accurate yield of desired product for this substrate a control reaction was performed using the same procedure in the absence of chlorotrimethylsilane. The yield of desired product was then calculated by subtracting the amount of product formed in the control reaction from that formed in the presence of chlorotrimethylsilane. The desired product **3v** was observed (74% *in situ* yield calculated), due to low boiling point this substrate was not isolated, characterisation was performed from the crude reaction mixture.

*Note: Performing a control reaction without any acceptor substrate yielded **3v** (0.008 mmol). This value was considered when calculating the in situ yield of **3v** in presence of chlorotrimethylsilane as acceptor.*

^{1}H NMR (400 MHz, C_6D_6, 25 °C): δ = 0.03 (d, $^{3}J_{HF}$ = 7.2 Hz, 9H, Si(CH_3)$_3$).

^{13}C{^{1}H} NMR (101 MHz, C_6D_6, 25 °C): δ = −0.07 (d, $^{2}J_{CF}$ = 15.2 Hz, Si(CH_3)$_3$).

^{19}F NMR (377 MHz, C_6D_6, 25 °C): δ = −158.8 (m, 1F, Si*F* with $^{1}J_{FSi}$ satellites = 278.0 Hz).

All spectroscopic data in agreement with reported data – chemical shifts deviate due to solvent used.[20]

Fluorodimethylphenylsilane (3w) – CAS# 768-33-2

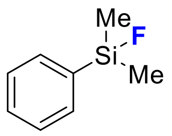

3w

In a dinitrogen containing glovebox, KHMDS (19.2 mg, 0.094 mmol) was dissolved in 1.0 mL C_6D_6 and transferred to J Young NMR tube. The reaction mixture was degassed *via* freeze-pump-thaw technique before HFC-134a (1.4 bar, 1.8 mL, 0.1 mmol) was added, the tube was inverted 3 times then allowed to stand for 70 minutes at 25 °C. A colour change from colourless solution to a black suspension was observed. To the suspension, chlorodimethylphenylsilane (6.9 mg, 0.04 mmol) and 1,2-difluorobenzene (4.32 µL, 0.044 mmol) was added. The reaction mixture was then heated to 100 °C for 1.5 hours. Yields were determined using integration compared to 1,2-difluorobenzene as an internal standard. The desired product (**3w**) was observed (65% *in situ* yield). Due to low boiling point this substrate was not isolated, characterisation was performed from the crude reaction mixture.

^{1}H NMR (400 MHz, C_6D_6, 25 °C): δ = 7.51–7.45 (m, 2H, Ar*H*), 7.20–7.13 (m, 3H, Ar*H*, Ar*H*; signals overlapping with solvent; ^{1}H-^{13}C-HSQC confirmed multiple peaks under C_6D_6), 0.26 (d, $^3J_{HF}$ = 7.2 Hz, 6H, $(CH_3)_2$).

^{19}F NMR (377 MHz, C_6D_6, 25 °C): δ = (−162.9)–(−163.1) (m, 1F, Si*F*; $^1J_{FSi}$ satallites = 275 Hz).

^{29}Si NMR (80 MHz, C_6D_6, 25 °C): δ = 20.3 (s, *Si*F; assigned *via* ^{1}H/^{29}Si HMBC – see Supplementary Figure 71 below).

All spectroscopic data in agreement with reported data – chemical shifts deviate due to solvent used.[21]

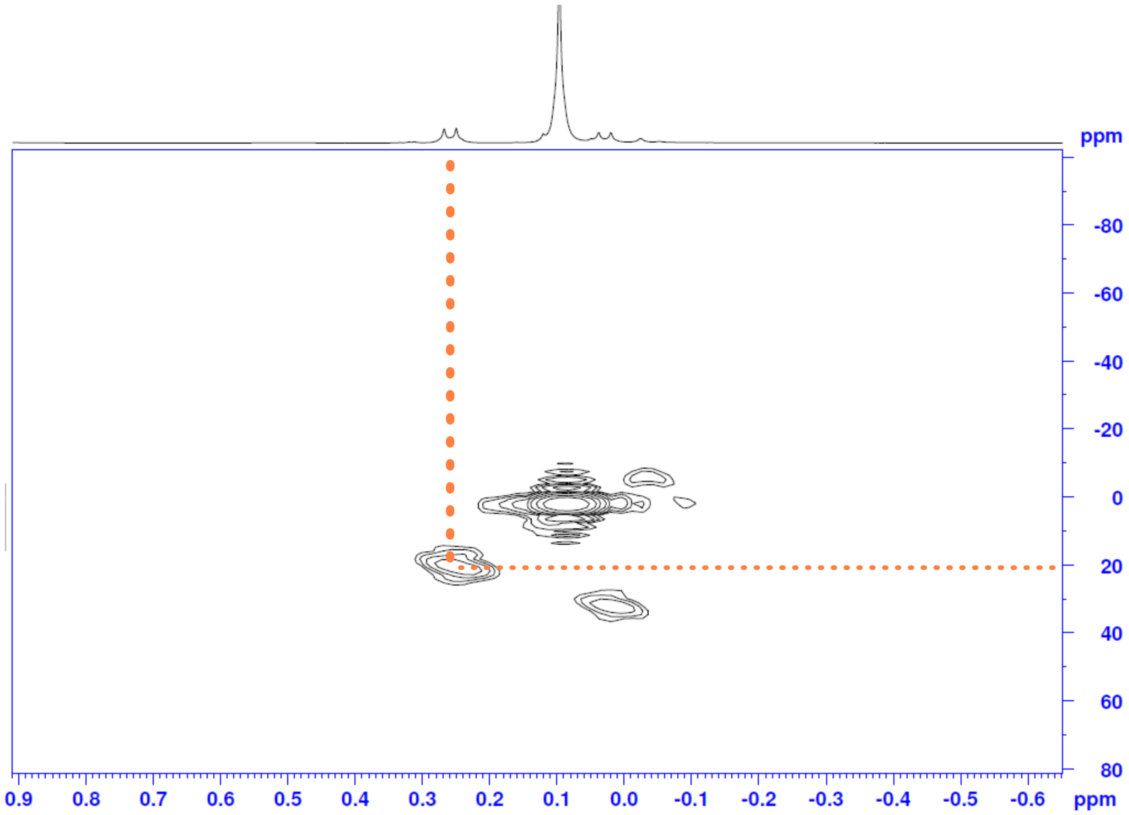

Supplementary Figure 71: ^{1}H-^{29}Si-HMBC of the reaction mixture containing **3w** (C_6D_6, 25 °C).

Fluorotriphenylsilane (3x) – CAS# 379-50-0

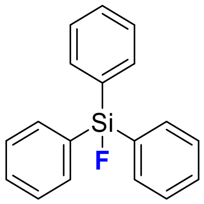

3x

In a dinitrogen containing glovebox, KHMDS (197.0 mg, 0.99 mmol) was dissolved in 8 mL THF and transferred to an 80 mL ampoule. The ampoule was degassed *via* freeze-pump-thaw technique before HFC-134a (1.2 bar, 72 mL, 3.49 mmol) was added, the vessel was shaken initially then the reaction mixture was allowed to vigorously stir for 70 minutes at 25 °C. A colour change from colourless solution to a black suspension was observed. To the suspension, chlorotriphenylsilane (97.7 mg, 0.33 mmol) was added. The reaction mixture was allowed to stir for 1 hour at 100 °C. The crude solution was centrifuged for 30 minutes at 940 x g, and the solution was decanted. The solution was filtered through a celite microfiltration plug (1 cm depth, 0.7 cm diameter) and washed with hexane (3x1 mL). The filtrate was concentrated *in vacuo*. The product (**3x**) was obtained as a pale brown oil (92.9 mg, 0.33 mmol, >99%).

^{1}H NMR (400 MHz, C_6D_6, 25 °C): δ = 7.64 (d, $^{3}J_{HH}$ = 7.2 Hz, 6H, Ar*H*), 7.15–7.05 (m, overlapping, 9H, Ar*H*).

^{13}C{^{1}H} NMR (101 MHz, C_6D_6, 25 °C): δ = 135.4, 133.1 (d, $^{2}J_{CF}$ = 17.2 Hz, C_{Ar}), 131.0, 128.4.

^{19}F NMR (377 MHz, C_6D_6, 25 °C): δ = −168.9 (s, 1F, Si–*F*; satellites: $^{1}J_{FSi}$ = 283.5 Hz).

^{29}Si NMR (80 MHz, C_6D_6, 25 °C): δ = −3.1 (*Si*–F).

^{29}Si NMR assignment based on ^{1}H-^{29}Si HMBC correlation data.

NMR data consistent with literature.[22]

11.5. P–F bond formation: Synthesis and characterisation of products

Diphenylphosphinic fluoride (3y) – CAS# 1135-98-4

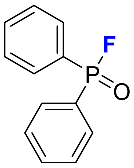

3y

In a dinitrogen containing glovebox, KHMDS (197 mg, 0.99 mmol) was dissolved in 8 mL THF and transferred to a 20 mL ampoule. The reaction mixture was degassed *via* freeze-pump-thaw technique before HFC-134a (1.4 bar, 12 mL, 0.68 mmol) was added, the vessel was shaken initially then the reaction mixture allowed to vigorously stir for 70 minutes at 25 °C. A colour change from colourless solution to a black suspension was observed. To the suspension, diphenylphosphinic chloride (78.1 mg, 0.33 mmol) was added. The reaction mixture was then heated to 100 °C and allowed to stir for 1 hour. Once cooled to room temperature, the solution was filtered via cannulation. The fine suspension was centrifuged for 30 minutes at 940 x g and the solution decanted and concentrated *in vacuo*. The resulting brown oil was dissolved in benzene (1 mL) and filtered through a celite plug microfiltration (1 cm depth, 0.7 cm diameter) and washed with benzene (3x 1.0 mL). The filtrate was concentrated *in vacuo*. The product (**3y**) was obtained as a pale brown oil (55.7 mg, 0.25 mmol, 77%).

^{1}H NMR (400 MHz, C_6D_6, 25 °C): δ = 7.75–7.67 (m, 4H, *m*-Ar*H*), 7.03–6.97 (m, 2H, *p*-Ar*H*), 6.95–6.88 (m, 4H, *o*-Ar*H*).

^{13}C{^{1}H} NMR (101 MHz, C_6D_6, 25 °C): δ = 133.0 (d, $^{4}J_{CP}$ = 2.9 Hz, *p*-C_{Ar}H), 131.7 (d, $^{3}J_{CP}$ = 11.1 Hz, *m*-C_{Ar}H), 130.1 (dd, $^{1}J_{CP}$ = 140.4 Hz, $^{2}J_{CF}$ = 23.2 Hz, C^{iV}_{Ar}), 128.9 (d, $^{2}J_{CP}$ = 13.8 Hz, *o*-C_{Ar}H).

^{19}F NMR (377 MHz, C_6D_6, 25 °C): δ = −73.1 (d, $^{1}J_{FP}$ = 1016.5 Hz, 1F, P(O)*F*).
^{31}P{^{1}H} NMR (162 MHz, C_6D_6, 25 °C): δ = 38.4 (d, $^{1}J_{PF}$ = 1016.5 Hz, 1P, *P*(O)F).

NMR consistent with literature, chemicals shifts are shifted due to solvent used.[23]

Di-tert-butylfluorophosphine (3z) – CAS# 29146-24-5

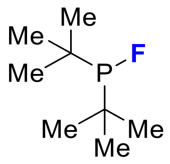

3z

In a dinitrogen containing glovebox, KHMDS (20.9 mg, 0.105 mmol) was dissolved in 1 mL C_6D_6 and transferred to J Young NMR tube. The reaction mixture was degassed *via* freeze-pump-thaw technique before HFC-134a (1.4 bar, 1.8 ml, 0.102 mmol) was added, the tube was inverted 3 times then allowed to stand for 70 minutes at 25 °C. A colour change from colourless solution to a black suspension was observed. To the suspension, di-tert-butylchlorophosphine (7.30 mg, 0.040 mmol) and 1,2-difluorobenzene (3.94 μL, 0.040 mmol) was added. The reaction mixture was then heated to 100 °C for 18 hours. Yields were determined using integration compared to 1,2-difluorobenzene as an internal standard. Desired product (**3z**) was observed (58% *in situ* yield), due to low boiling point this substrate was not isolated, characterisation was performed from the crude reaction mixture.

^{1}H NMR (400 MHz, C_6D_6, 25 °C): δ = 1.05 (d, $^3J_{HP}$ = 11.3 Hz, 18H, CH_3).

^{19}F NMR (377 MHz, C_6D_6, 25 °C): δ = −217.5 (d, $^1J_{FP}$ = 870.5 Hz, P*F*).

^{31}P{^{1}H} NMR (162 MHz, C_6D_6, 25 °C): δ = 213.1 (d, $^1J_{PF}$ = 870.5 Hz, *P*F).

NMR data consistent with literature, shifted due to solvent used.[24]

Diphenylfluorophosphine (3aa) – CAS# 20472-53-1

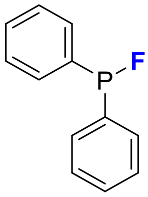

3aa

*This procedure used KF isolated from the reaction of **1j** and KHMDS, see section 7.2 for procedure method A.*

In a dinitrogen containing glovebox, isolated KF (8.4 mg, 0.14 mmol, 90%/wt KF) and chloro-diphenylphosphine (9.7 mg, 0.044 mmol) were dissolved in 1.0 mL C_6D_6 and transferred to J Young NMR tube. 1,2-difluorobenzene (4.32 µL, 0.044 mmol) was added and the reaction mixture was heated to 100 °C for a total of 14 hours. Yields were determined using integration compared to 1,2-difluorobenzene as an internal standard. Desired product (**3aa**) was observed (66% *in situ* yield). Due to the reactive nature of this substrate, attempt to isolate were unsuccessful and characterisation was performed from the crude reaction mixture.

^{1}H NMR (400 MHz, C_6D_6, 25 °C): δ = 7.44 (t, $^{3}J_{HH}$ = 7.4 Hz, 4H, Ar*H*), 7.06-6.99 (m, overlapping, 6H, Ar*H*).

^{13}C{^{1}H} NMR (101 MHz, C_6D_6, 25 °C): δ = 130.53, 130.45 (dd, $^{2}J_{CP}$ = 23.2 Hz, $^{3}J_{CF}$ = 6.3 Hz, C_{Ar}H), 129.8, 128.8 (d, $^{3}J_{CP}$ = 6.9 Hz, C_{Ar}H) – data confirmed with 2D NMR techniques.

^{19}F NMR (377 MHz, C_6D_6, 25 °C): δ = −197.0 (d, $^{1}J_{FP}$ = 882.6 Hz, P*F*).

^{31}P NMR (162 MHz, C_6D_6, 25 °C): δ = 163.5 (d, $^{1}J_{PF}$ = 882.6 Hz, *P*F).

NMR data in accordance with existing literature values.[25]

11.6. I–F bond formation: Synthesis and characterisation of products

1-Fluoro-3,3-dimethyl-1,2-benziodoxole (3ab) – CAS# 1391728-13-4

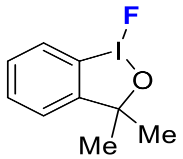

3ab

In a dinitrogen containing glovebox, KHMDS (175 mg, 0.88 mmol) was dissolved in 4 mL THF and transferred to a 20 mL ampoule. The reaction mixture was degassed *via* freeze-pump-thaw technique before HFC-134a (1.4 bar, 16 mL, 0.90 mmol) was added, the vessel was shaken initially then the reaction mixture allowed to vigorously stir for 55 minutes at 25 °C. A colour change from colourless solution to a black suspension was observed. To the suspension, 1-chloro-1,3-dihydro-3,3-dimethyl-1,2-benziodioxole (90.1 mg, 0.30 mmol) was added. The reaction mixture was allowed to stir for 1 hour at 25 °C. The crude solution was filtered through a celite plug (1 cm depth, 0.7 cm diameter) and washed with THF (3 mL). The filtrate was concentrated *in vacuo* and purified by recrystallisation using toluene/*n*-hexane (2 mL, 1:1 mixture) and washed with cold *n*-hexane. Colourless crystals of **3ab** were obtained (63.8 mg, 0.23 mmol, 75%).

^{1}H NMR (400 MHz, C_6D_6, 25 °C): δ = 7.93 (d, $^3J_{HH}$ = 8.0 Hz, 1H, Ar*H*), 6.95 (t, $^3J_{HH}$ = 7.6 Hz, 1H, Ar*H*), 6.86 (t, $^3J_{HH}$ = 7.4 Hz, 1H, Ar*H*), 6.53 (d, $^3J_{HH}$ = 7.4 Hz, 1H, Ar*H*), 1.23 (s, 6H, $(CH_3)_2$).

^{13}C{^{1}H} NMR (101 MHz, C_6D_6, 25 °C): δ = 149.1, 130.1, 129.9, 129.0 (d, $^3J_{CF}$ = 8.7 Hz, C_{Ar}H), 125.7, 116.6 (d, $^2J_{CF}$ = 9.2 Hz, C^{iV}_{Ar}), 84.2, 29.1 (d, $^4J_{CF}$ = 3.4 Hz, CH_3).

^{19}F NMR (377 MHz, C_6D_6, 25 °C): δ = −139.7 (s, 1F, I–*F*).

All spectroscopic data in accordance with literature; chemicals shifts are shifted due to solvent used.[26]

11.7. Less successful fluorination acceptors

In contrast to **3a–3ab**, few commercially available potential fluorination acceptors were identified as limitations. These compounds either performed poorly or showed no propensity for transfer fluorination under the conditions investigated. These compounds and the corresponding outcomes are listed below (Supplementary Figure 72).

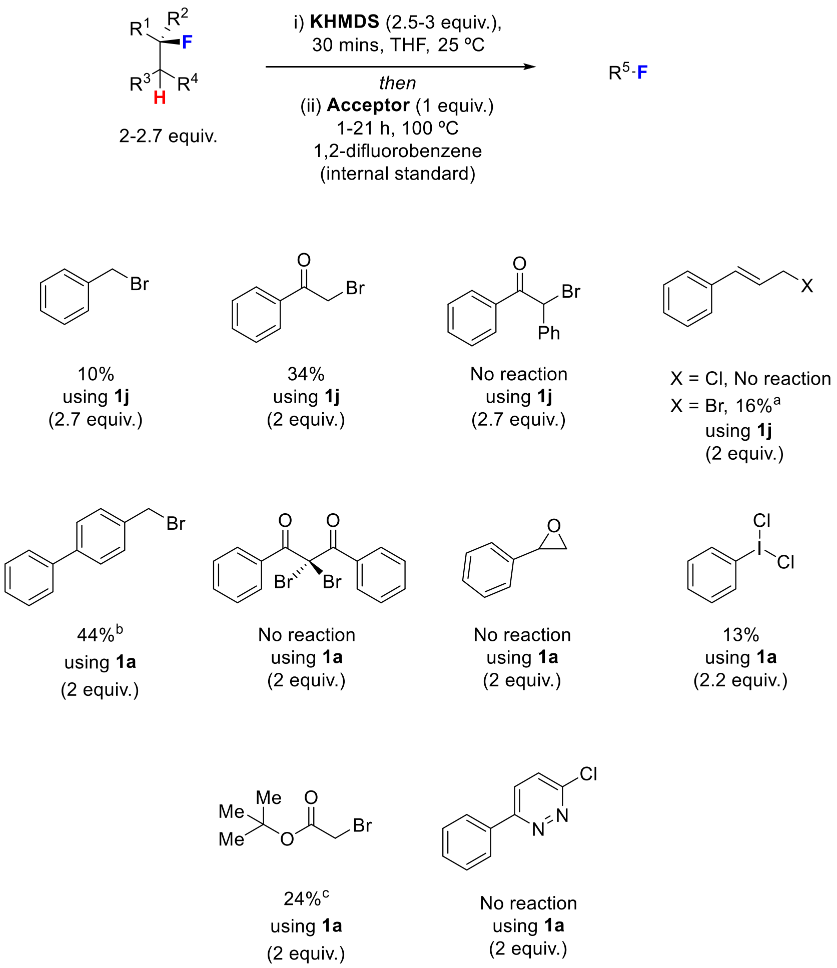

Supplementary Figure 72: Less successful candidates for the fluorination acceptor substrates performed using standard conditions albeit with extended reaction times. *In situ* yields given for corresponding fluorinated substrates using ^{19}F NMR spectroscopy using 1,2-difluorobenzene as an internal standard. Benzyl bromide and cinnamyl chloride were distilled prior to use. [a] Reaction time for this substrate was 5 days at 100 °C. [b] Reaction time for this substrate was 115 hours at 100 °C. [c] Reaction time for this substrate was 98 hours at 100 °C.

12. Fluorination using **1v**: Polyvinylidene fluoride

12.1. Fluorine transfer procedure of **1v** using KHMDS

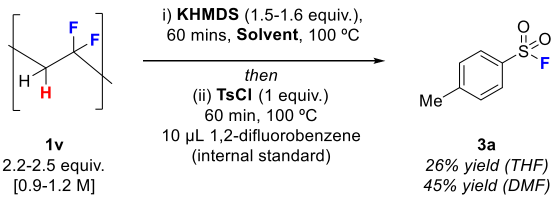

Supplementary Scheme 60: Transfer fluorination using **1v** as a donor.

A one-pot procedure (Procedure F) for the screening of polyvinylidene difluoride (PVDF) was performed as follows (Supplementary Scheme 60):

In THF (Supplementary Figure 73): In a dinitrogen containing glovebox, PVDF (8.8 mg, 0.14 mmol) and 0.5 mL THF were transferred into a J. Youngs NMR tube. To this reaction mixture, a solution of KHMDS (16.0 mg, 0.08 mmol) in 0.2 mL THF was added at room temperature and the dehydrofluorination reaction was carried out at 100 °C for 60 minutes in an oil bath. The reaction mixture instantly became heterogeneous and with solid being deposited on the wall of the J Young NMR tube. At longer reaction times, a dark precipitate was also observed. The NMR tube was sealed and inverted every 10 minutes to maintain proper mixing. After 60 minutes, the tube was taken back into the glovebox, and tosyl chloride (TsCl, 10.4 mg, 0.05 mmol) in 0.3 mL THF was added to the J Young tube, followed by the addition of 10 µL *o*-DFB as internal standard. The NMR tube was then sealed, removed from the glovebox and transferred into a 100 °C hot oil bath. The NMR tube was inverted every 15 minutes to maintain a proper mixing of the reactants. After a reaction time of 60 minutes, the tube was removed from the oil bath and allowed to cool to room temperature, after which the yield of tosyl fluoride (TsF; **3a**) was determined *via* quantitative ^{19}F NMR spectroscopy.

In DMF (Supplementary Figure 74): In a dinitrogen containing glovebox, PVDF (7.7 mg, 0.120 mmol) and 0.6 mL DMF were transferred into a J. Youngs NMR tube. To this reaction mixture, a solution of KHMDS (17.1 mg, 0.09 mmol) in 0.4 mL DMF was added at room temperature and the dehydrofluorination reaction was carried out at 100 °C for 60 minutes in an oil bath. The reaction mixture became less heterogeneous than in the case of THF as solvent, but with solids being deposited on the wall of the J Young NMR tube. At longer reaction times, a dark precipitate was also observed. The NMR tube was sealed and inverted every 10 minutes to maintain proper

mixing. After 60 minutes, the tube was taken back into the glovebox, and tosyl chloride (TsCl, 10.1 mg, 0.053 mmol) in 0.4 mL DMF was added to the J Young tube, followed by the addition of 10 μL *o*-DFB as internal standard. The NMR tube was then sealed, removed from the glovebox and transferred into a 100 °C hot oil bath. The NMR tube was inverted every 15 minutes to maintain a proper mixing of the reactants. After a reaction time of 60 minutes, the tube was removed from the oil bath and allowed to cool to room temperature, after which the yield of tosyl fluoride (TsF; **3a**) was determined *via* quantitative ^{19}F NMR spectroscopy.

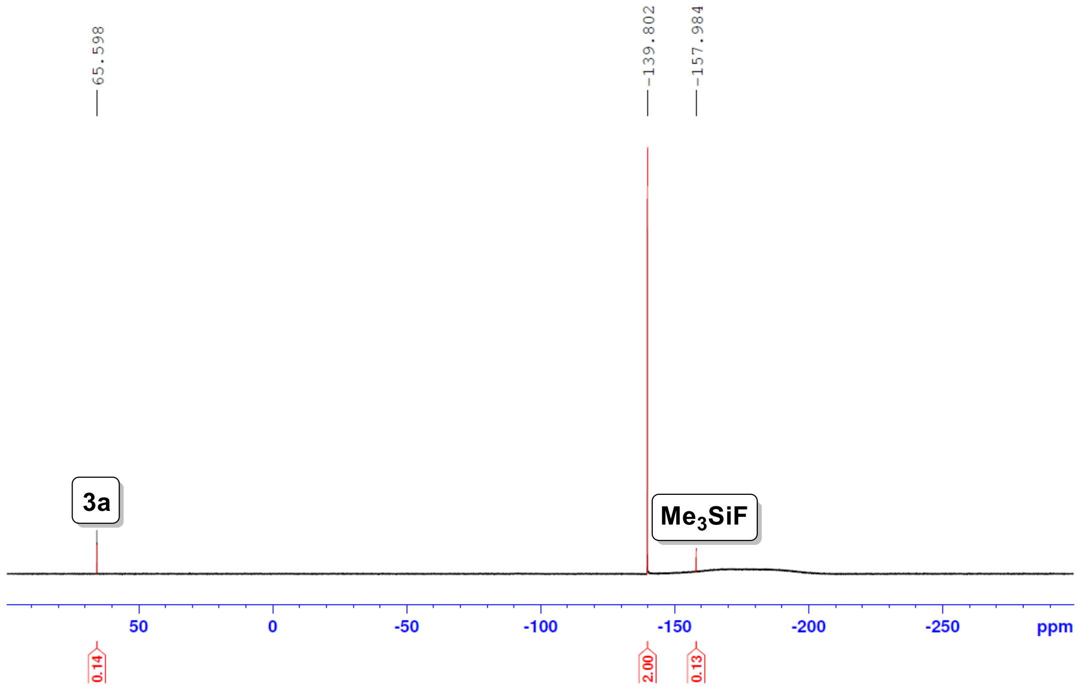

Supplementary Figure 73: ^{19}F NMR spectrum of fluorination of TsCl after 60 minutes activation of **1v** at 100 °C and 60 minutes fluorination at 100 °C (377 MHz, THF-h_8, 298K).

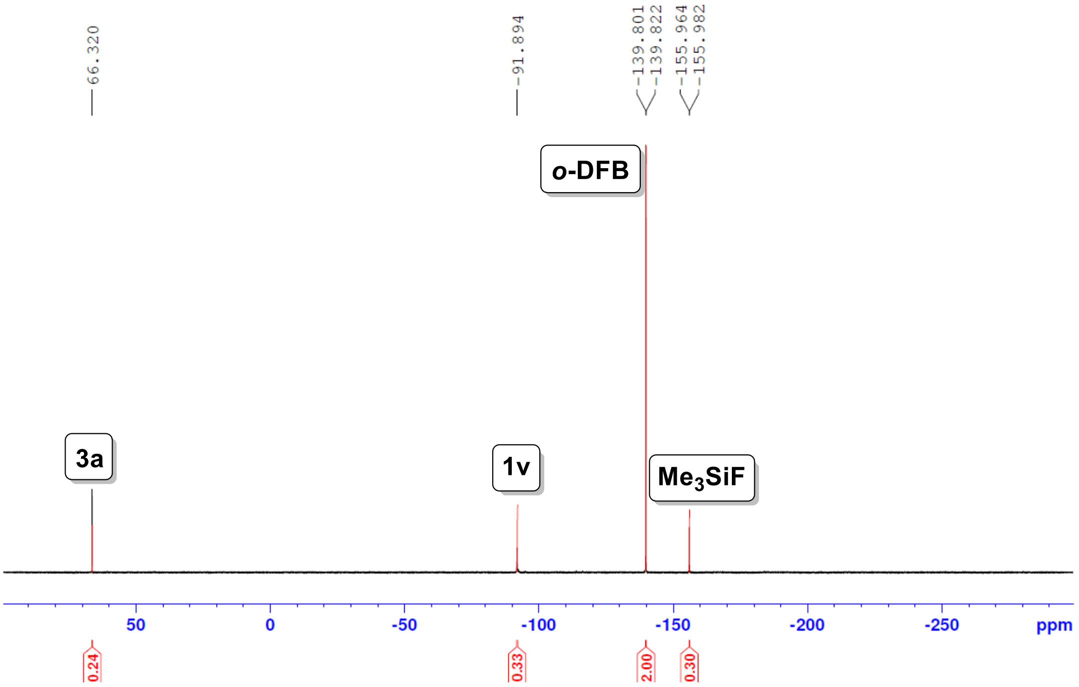

Supplementary Figure 74: ^{19}F NMR spectrum of fluorination of TsCl after 60 minutes activation of **1v** at 100 °C and 60 minutes fluorination at 100 °C (377 MHz, DMF-h_7, 298K

12.2 Fluorine transfer procedure of **1v** using KO^{t}Bu

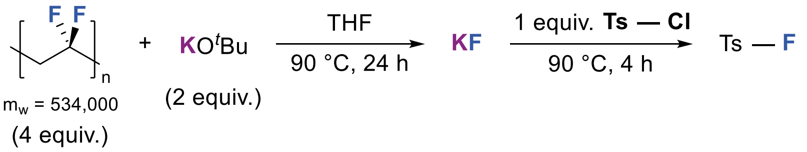

Scale of PVDF	Yield of TsF
1g	78%
10g	89%
50g	62%

Supplementary Scheme 61: Transfer fluorination using **1v** as a donor scaled up to 50 g.

<u>General Procedure for 100 mg to 1 g Scale of **1v:**</u>

In a 1 mL vials, **1v** (4 equiv.) was weighed out and transferred into an ampoule containing a stirrer bar using THF to make a 0.31 M solution. KO^{t}Bu (2 equiv.) was weighed out in a 1 mL vial and then added into the PVDF suspension using THF to transfer to make the reaction mixture a 0.25 M in relation to PVDF. The ampoule was sealed and heated to 100 °C for 4 h while stirring. The reaction mixture was allowed to cool until room temperature before adding a 0.26 M solution of TsCl in THF (1 equiv.) *via* syringe. The ampoule was resealed and heated at 100 °C for a further 1 h before cooling to room temperature. The reaction mixture was opened to air and the suspension was gravity filtered to collect the supernatant. The solids were washed with Et_2O (3 X 5 mL) and the organic fractions were combined. The volatiles were removed under reduced pressure (20 mbar) to afford a crude brown oil.

The crude product was purified by column chromatography using silica gel. Isolation achieved by column chromatography in ethyl acetate and hexane (1:20). Yields are given below based on scale.

PVDF 100 mg, 1.56 mmol (Isolated Yield of TsF: 69%, 47 mg, 0.27 mmol)
PVDF 1 g, 15.62 mmol (Isolated Yield of TsF: 78%, 528 mg, 3.03 mmol)

Procedure for 10 g Scale of **1v**:

A 1 L two-neck RBF with a condenser was charged with PVDF (10 g, 156 mmol) and THF (450 mL; Merck 2.5 L ≥99.0%, ACS reagent, no prior drying or degassing) under a flush of N_2. The stirring solution (300 rpm) was cooled in an ice bath before adding KO^{t}Bu (8.762 g, 78.09 mmol) under a flush of N_2. The reaction mixture was heated to 100 °C in an oil bath for 24 h before cooling to atmospheric temperature. The TsCl (7.443 g, 39.04 mmol)) was added to the reaction under a flush of N_2 before heating the reaction mixture back to 100 °C for an additional 4 h. The cooled solution was filtered slowly through a bed of activated charcoal (diameter = 6 cm, thickness = 2 cm), and the solids were rinsed with THF (3 X 20 mL). The volatiles were removed under reduced pressure (5 x 10^{-2} mbar), without addition of an external heating source to avoid the sublimation of **3a**, to obtain off white crystalline TsF (6.029 g, 34.61 mmol, 89%).

Procedure for 50 g Scale of **1v**:

A 5 L three-neck RBF with a condenser and a solid addition funnel was charged with PVDF (50 g, 781 mmol) and THF (2 L; Merck 2.5 L ≥99.0%, ACS reagent, no prior drying or degassing) under a flush of N_2. The stirring solution (300 rpm) was cooled in an ice bath before adding KO^{t}Bu (43.81 g, 390 mmol) under a flush of N_2 into the addition funnel and then subsequently added it to the PVDF solution over the span of 30 min. The addition funnel was removed, and the reaction mixture was heated to 90 °C in a sand bath for 24 h before cooling to atmospheric temperature. The solids addition funnel was added to the RBF and purged with N_2 before adding the TsCl (37.22 g, 195 mmol) under a flush of N_2. The TsCl was added slowly to the reaction mixture over the span of 30 min before removing the addition funnel and heating the reaction mixture back to 90 °C for an additional 4 h. The cooled solution was filtered slowly through a bed of activated charcoal (diameter = 15 cm, thickness = 5 cm) and the solids were rinsed with THF (3 X 100 mL). The volatiles were removed under reduced pressure (5 x 10^{-2} mbar), without addition of an external heating source to avoid sublimation of **3a**, to obtain off white crystalline TsF (21 g, 121 mmol, 62 %).

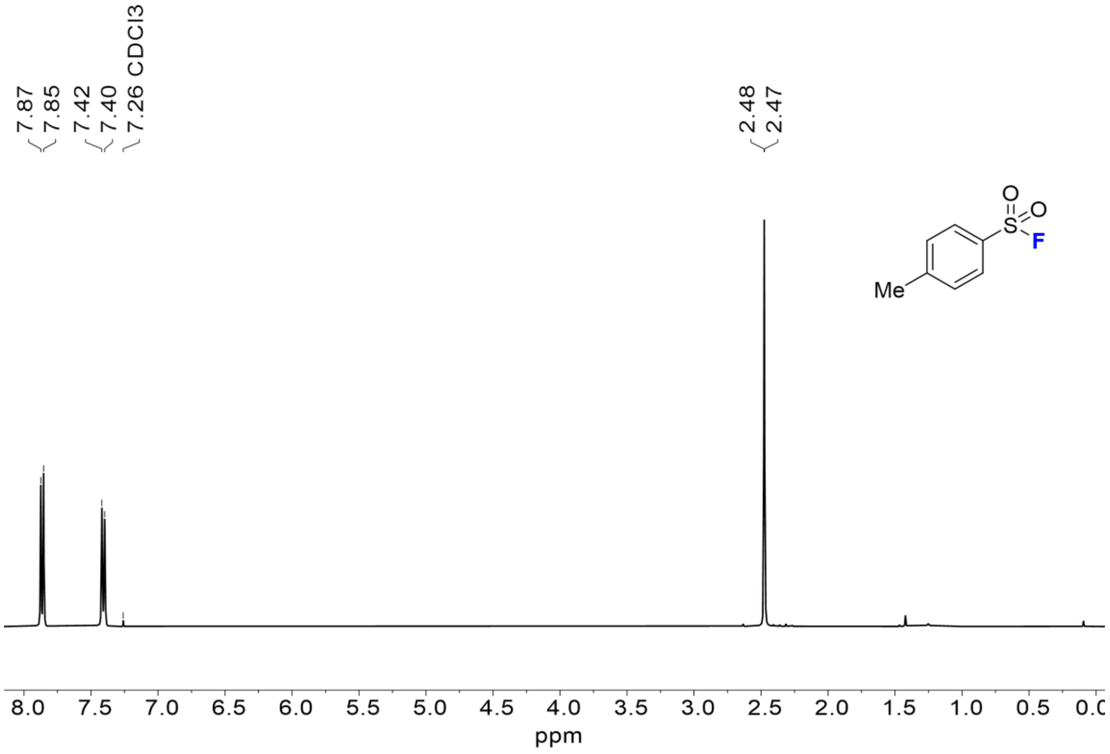

Supplementary Figure 75: ^{1}H NMR spectrum of TsF isolated from the fluorine transfer reaction of **1v** (377 MHz, $CDCl_3$, 25 °C).

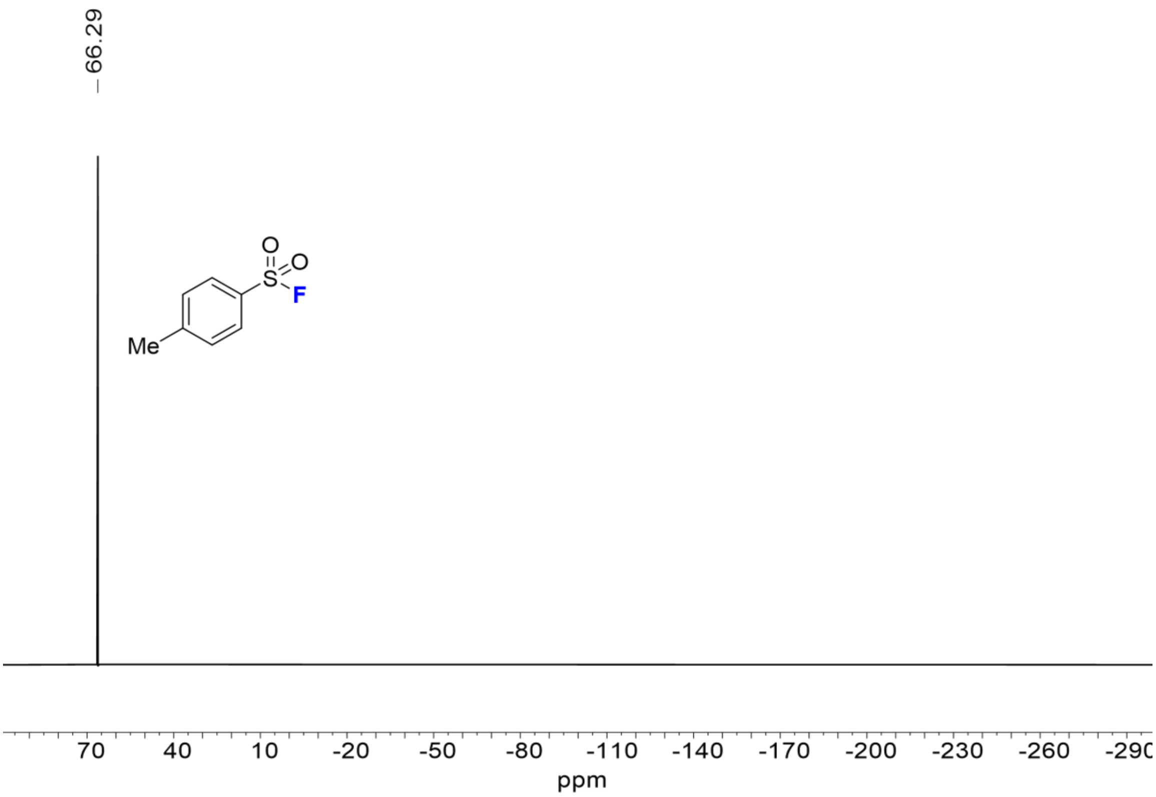

Supplementary Figure 76: ^{19}F NMR spectrum of TsF isolated from the fluorine transfer reaction of **1v** (377 MHz, $CDCl_3$, 25 °C).

12.3 Characterisation of post reaction precipitate for **1v**

In an effort to understand the fate of the PVDF after the fluorine transfer reaction, different characterisation tools were employed to gain a deeper understanding, including FTIR, CHN elemental analysis, differential scanning calorimetry, solid-state NMR, and powder XRD. Three samples were analysed to compare their properties; commercial powder PVDF (Mw = 534,000 g/mol); the collected black precipitate isolated from the reaction mixture; and the isolated black precipitate that had been washed with water to remove any water-soluble compounds, including the excess KF.

12.3.1 FTIR

IR was used to determine the nature and phase of the PVDF in the isolated precipitate (Supplementary Figure 77). The commercial powder PVDF was determined to be mainly in the α phase with a small amount in the β phase, indicated by the labelled frequencies.[61] A new frequency was observed in the spectra of the precipitate from after the reaction at 1571 cm^{-1}, indicative of newly formed C=C double bonds. This supports the proposed mechanism that the defluorination occurs through a deprotonation followed by a β-fluoride elimination. Besides the formation of C=C bonds, the other resonances match with the PVDF starting material, indicating that the remaining PVDF was still mainly in the α phase.

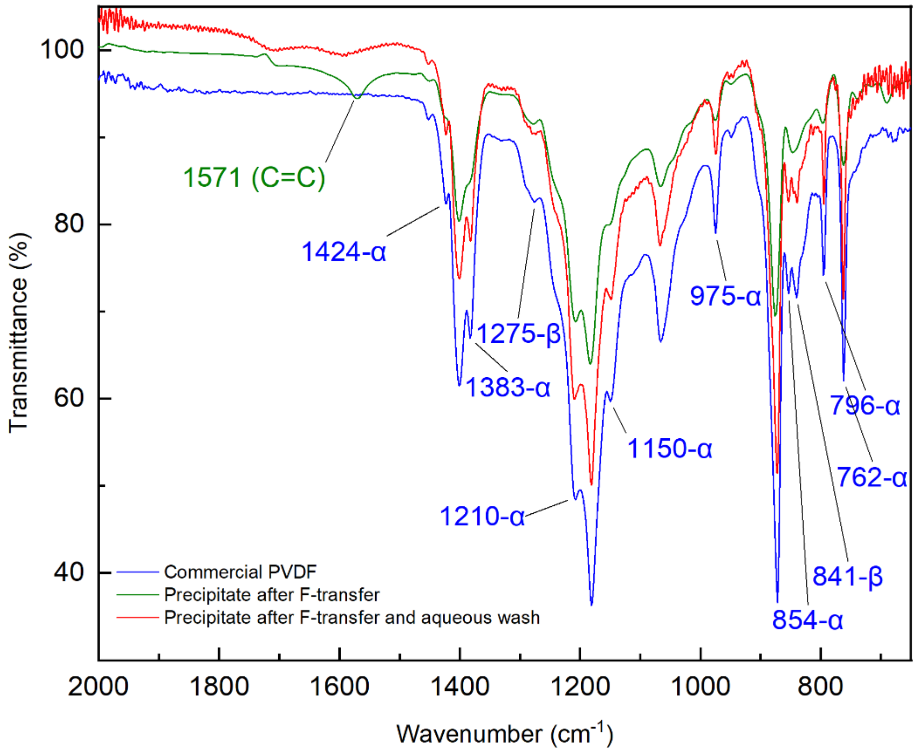

Supplementary Figure 77: FTIR spectrum of the commercial powder PVDF (blue), the post reaction isolated precipitate (green), and the aqueous washed precipitate (red).

12.3.2 CHN Elemental Analysis

In line with the proposed mechanism for the fluorine transfer, it was hypothesised that the resulting polymeric material remaining after the reaction would increase in carbon content from the increase of double bond character. The isolated precipitate post reaction was found to be lower in % carbon than the commercial PVDF, which was attributed to the high content of excess KF in the isolated precipitate. This was supported by results determined from the aqueous washed precipitate, which would extract any of the water-soluble salts in the precipitate. The carbon content was found to be in-between the expected values of PVDF and the alkene equivalent, indicating the remaining precipitate was partially defluorinated PVDF.

Supplementary Table 14: CHN elemental analysis of commercial PVFD, precipitate collected post reaction and the aqueous washed precipitate.

Sample	% Carbon	% Hydrogen	% Nitrogen
Expected 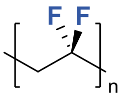	37.51	3.15	0
Expected 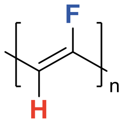	54.56	2.29	0
Commercial PVDF	37.52	2.71	0.18
Black Precipitate from F-Transfer Reaction	29.33	2.26	0
Black Precipitate after aqueous wash from F-Transfer Reaction	44.06	3.01	0

12.3.3 Differential scanning calorimetry (DSC)

The DSC data from the three samples seen in Graph S1 demonstrated that the precipitate collected after the reaction still contained PVDF, however there is also other polymeric species in the sample. This was concluded by the little change in the samples melt temperature compared to the commercial PVDF, however there is a decrease in the crystallisation temperature. In the DSC curve of the post reaction precipitate (Graph S3), the crystallisation temperature broadens and appears to have two peaks. This implies that there is a complex mixture of different polymer present after the reaction.

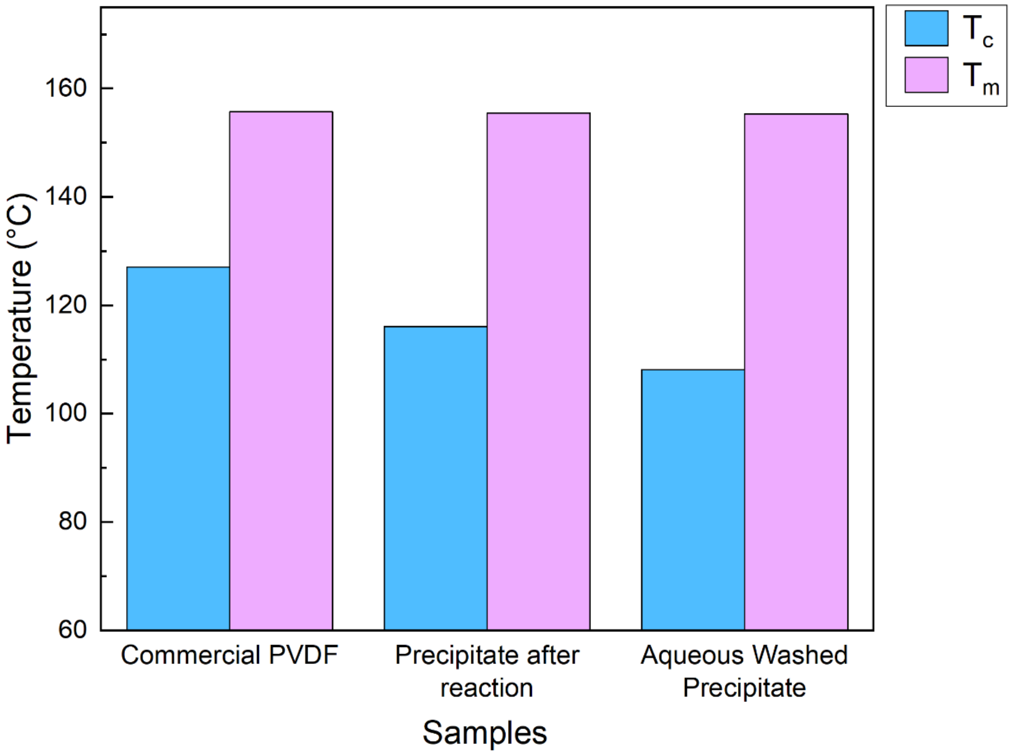

Graph S1: DSC analysis of three samples showing crystallisation temperature (T_c, blue) and melt temperature (T_m, pink)

Commercial Sample of PVDF

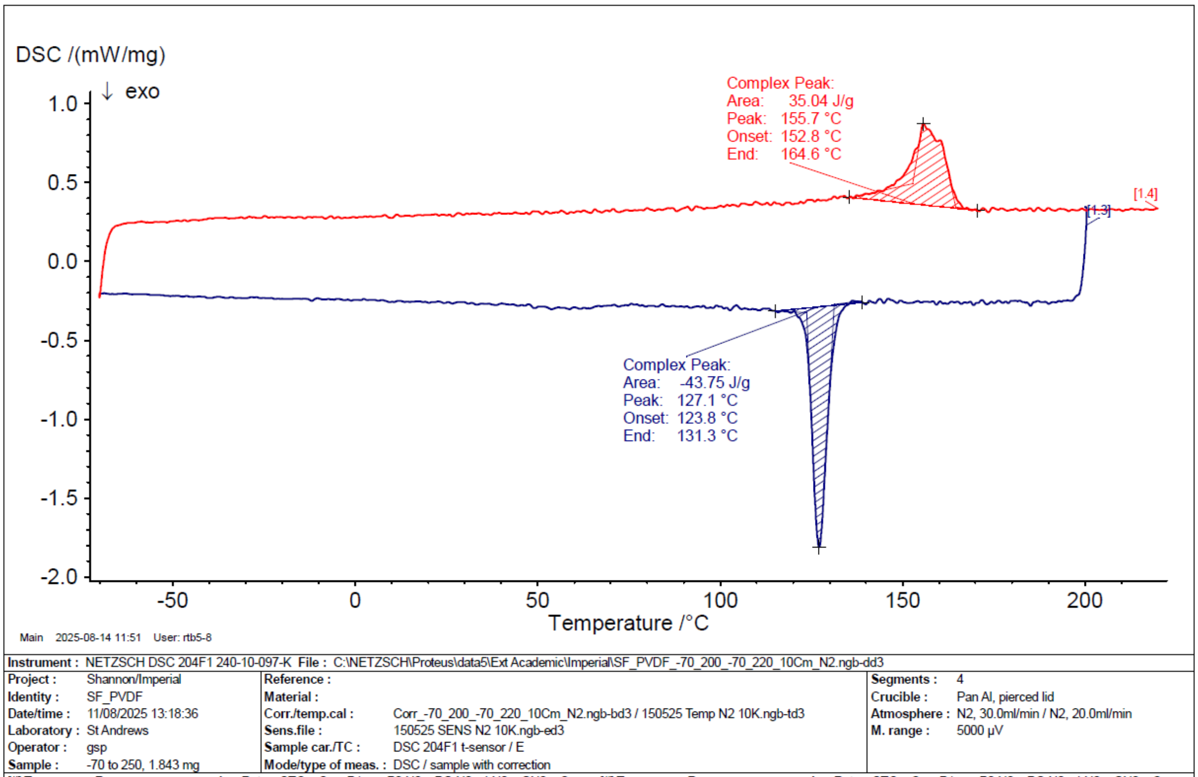

[#] Type	Range	Acq.Rate	STC	Co	P1:--	P2:N2	PG:N2	LN2	GN2	Corr.	[#] Type	Range	Acq.Rate	STC	Co	P1:--	P2:N2	PG:N2	LN2	GN2	Corr.
[1.3] Dynamic	200/10.0(K/min)/-70	300.00	1	0	0.0	30.0	20.0	5.0	Off	520	[1.4] Dynamic	-70/10.0(K/min)/220	300.00	1	0	0.0	30.0	20.0	5.0	Off	520

Graph S2: DSC curve of the second cycle for commercial PVDF with a sharp T_c at 155.7 °C and T_m at 127.1 °C.

Precipitate collected after fluorine transfer

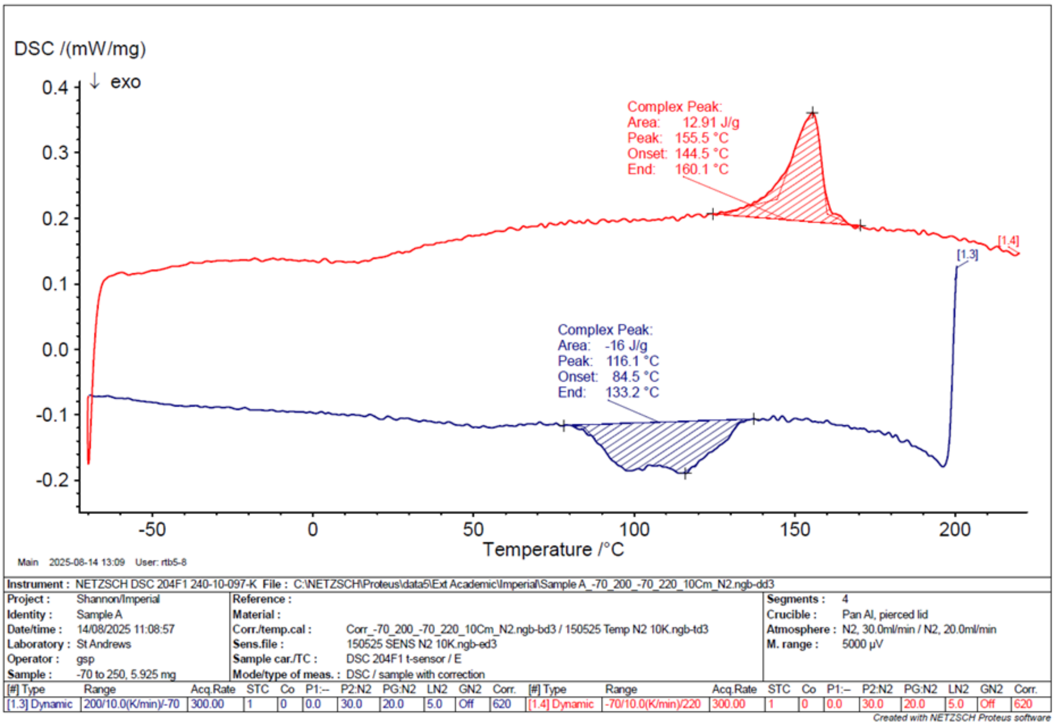

Graph S3: DSC curve of the second cycle for the precipitate collected after the fluorine transfer of **1v**, which displayed a broad T_c at 155.5 °C and T_m at 116.1 °C.

Aqueous washed precipitate collected after fluorine transfer reaction

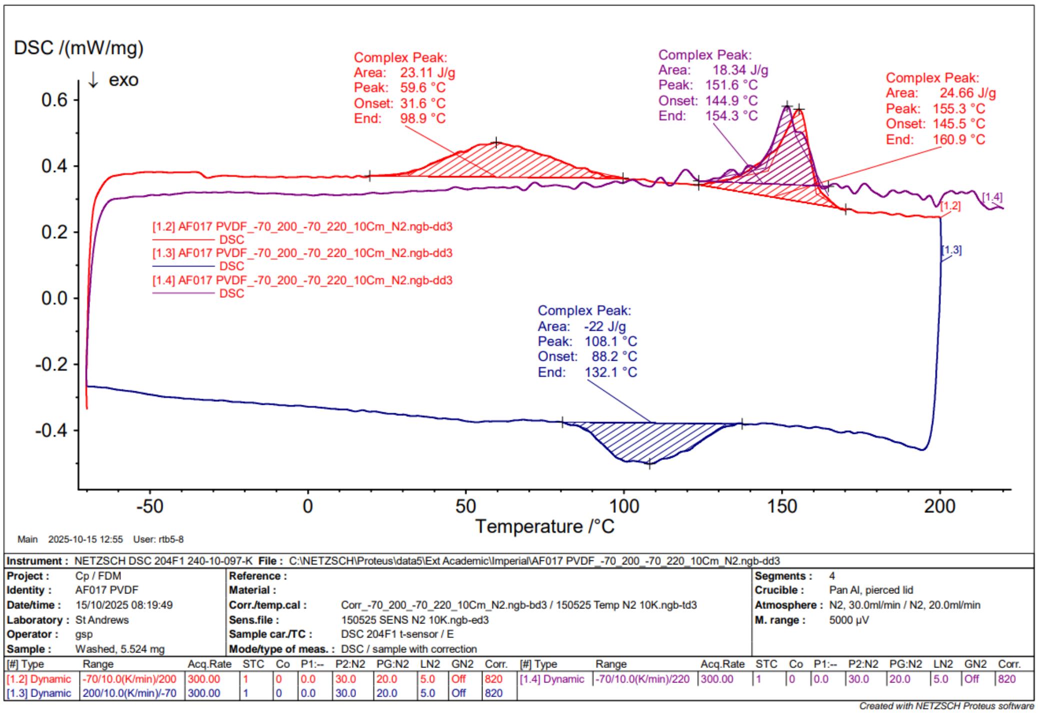

Graph S4: DSC curve of the aqueous washed precipitate collected after the fluorine transfer of **1v**, which displayed a broad T_c at 155.5 °C and T_m at 116.1 °C. Another T_c was observed at 59.6 °C on the first cycle, which was not observed on the second. This is due to the melting of poor secondary crystals of the original sample and is then not observed in the second cycle.[62]

12.3.4 Solid-State NMR

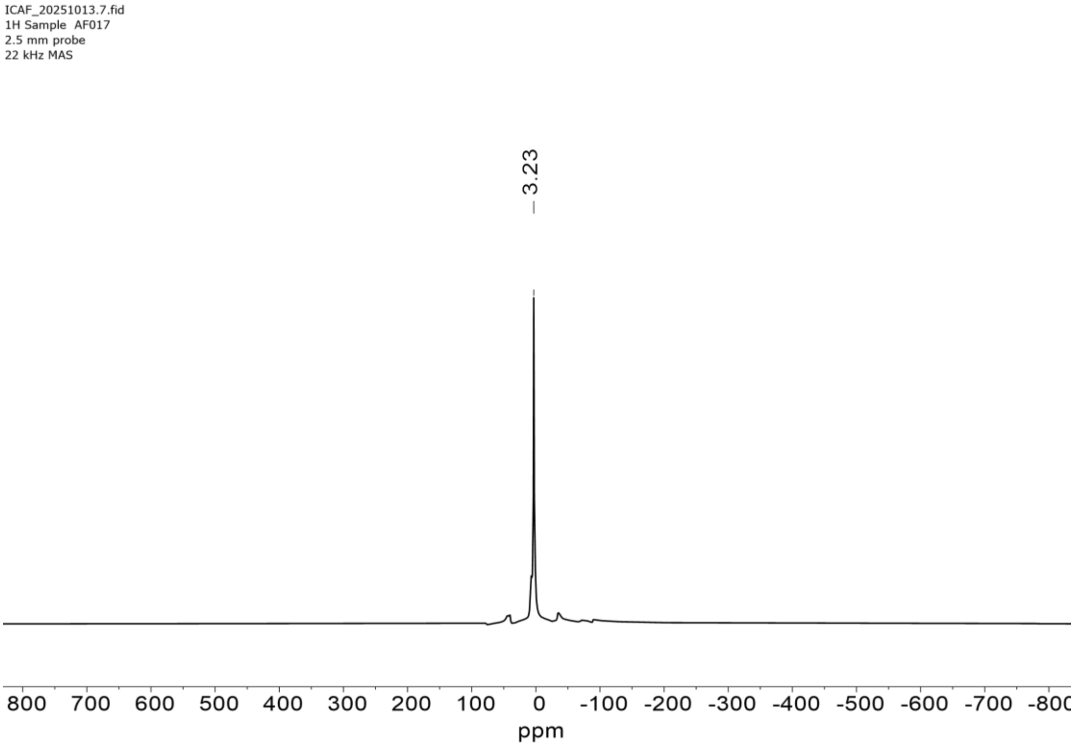

Supplementary Figure 78: MAS-NMR ^{1}H spectrum of the washed precipitate collected after the fluorine transfer reaction of **1v**.

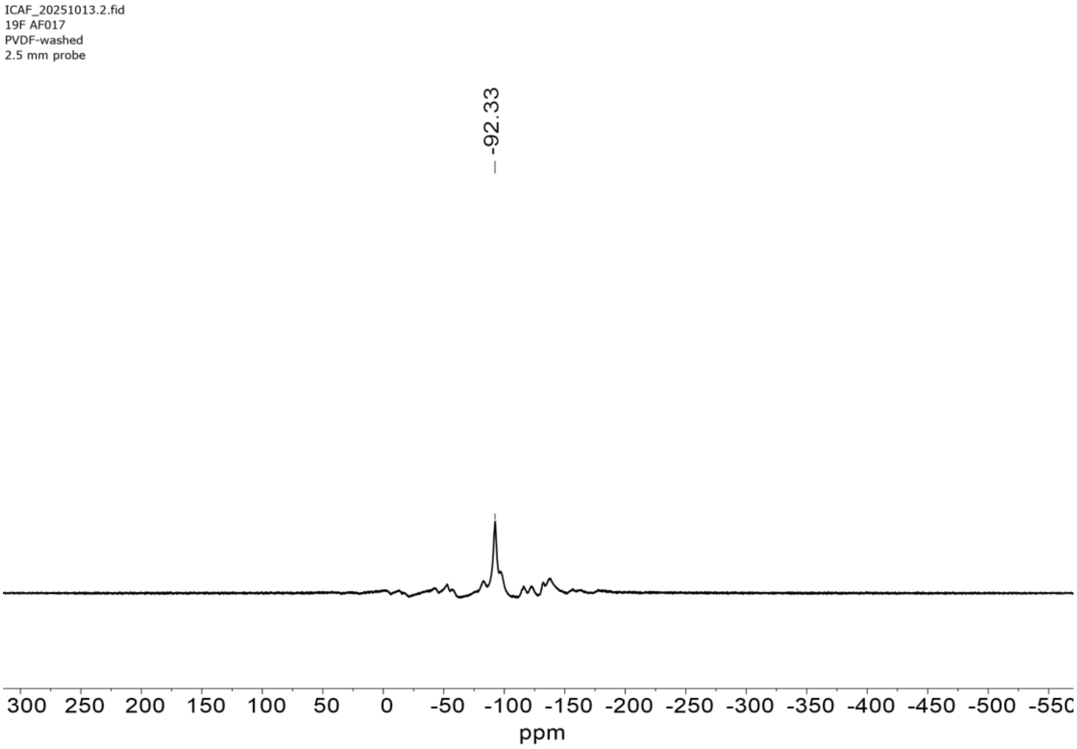

Supplementary Figure 79: MAS-NMR ^{19}F spectrum of the washed precipitate collected after the fluorine transfer reaction of **1v**.

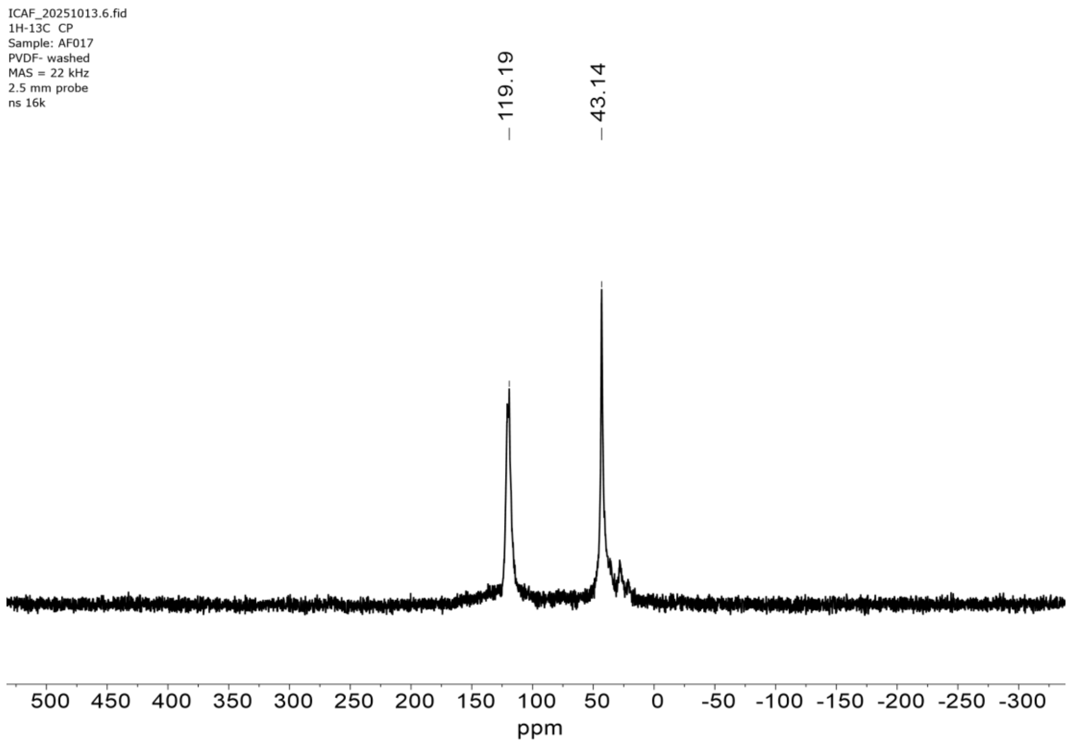

Supplementary Figure 80: MAS-NMR ^{1}H-^{13}C CP spectrum of the washed precipitate collected after the fluorine transfer reaction of **1v**.

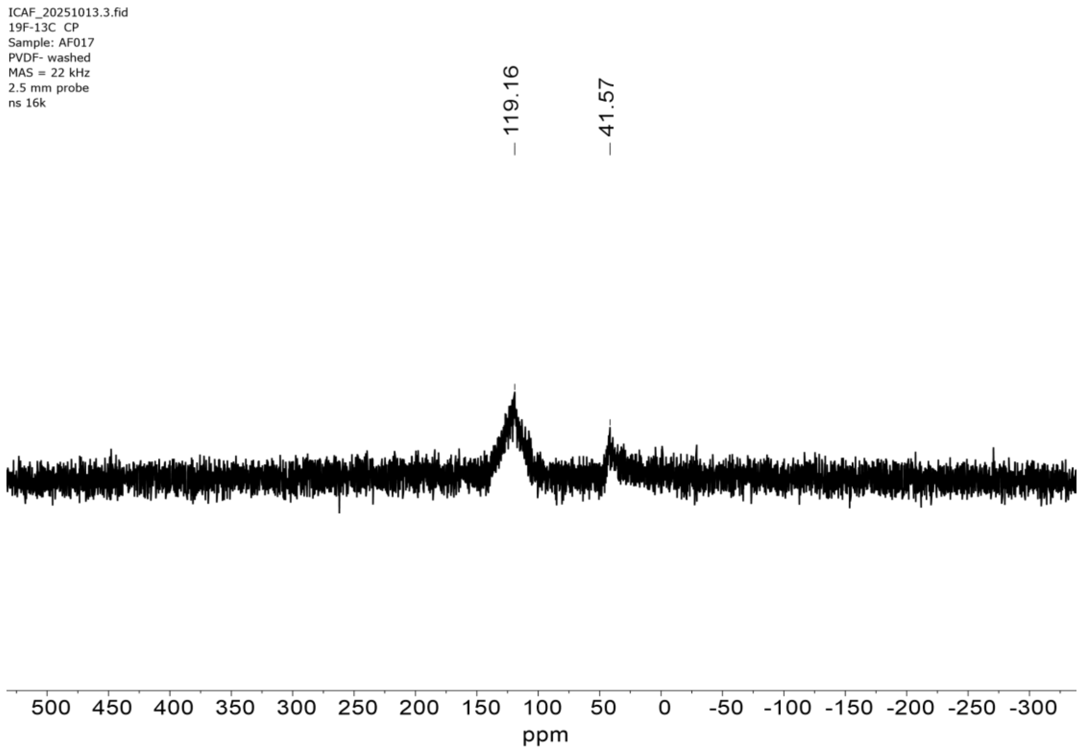

Supplementary Figure 81: MAS-NMR ^{19}F-^{13}C CP spectrum of the washed precipitate collected after the fluorine transfer reaction of **1v**.

12.2.5 Powder X-Ray Diffraction (XRD)

The powder XRD of the commercial PVDF versus the washed precipitate shows little differences in their diffraction pattern and indicate the crystalline content of the PVDF is in the α phase (Supplementary Figure 82).[61] However, the intensity of the diffraction has decreased, indicating the sample has lost part of its crystallinity. This concludes that after the aqueous wash, the remaining polymeric material is similar in structure to the starting material PVDF, likely due to the excess PVDF used in the reaction, however it has less crystalline regions. The consumed PVDF in the reaction would likely not have crystalline region and instead be amorphous and unobservable in the powder XRD spectrum.

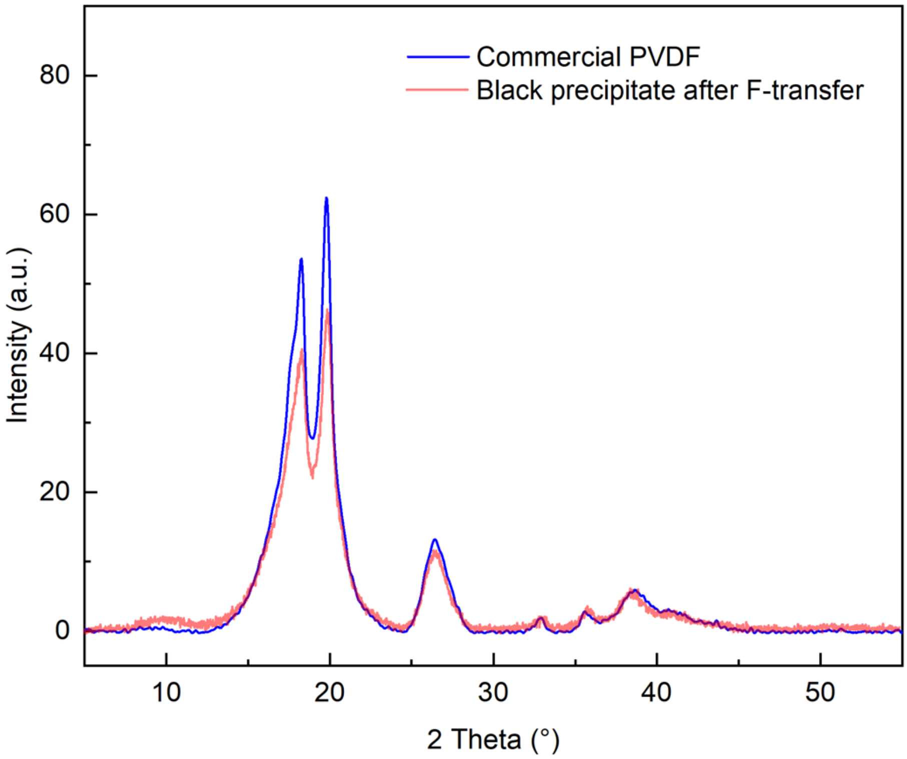

Supplementary Figure 82: Powder XRD of commercial powder PVDF (blue) and the aqueous washed reaction precipitate (red).

13. Total Defluorination of HFC-134a Scale-up in Flow:

13.1. General Flow Chemistry Procedure:

Flow reactions were performed using Vapourtec EasyScholar Integrated Flow Chemistry System. Reagents and solvents were delivered under nitrogen with a 19-gauge stainless steel luer lock needle (1.07 mm OD). 1/16’’ OD FEP tubing was used (ID = 1 mm). HFC-134a was delivered from a lecture bottle of 150 bar regulated to a range of 1.0 - 1.2 bar (absolute pressure) through a peristaltic pump to control flow rate, and a T-junction was used as a micromixer. DMSO and HFC gas was primed at reaction flow rate for at least 1 reaction coil volume. DMSO was switched to base solution to initiate flow reaction. After discarding 1 – 4 reactor coil volume, 4 x 1 mL reaction mixture was collected for steady state yield determination, diluted with deionised water and addition of 0.5 - 1.5 M NaOTf internal standard for ^{19}F quantitative NMR spectroscopy.

13.2. Flow Chemistry Stoichiometry Calculations and Assumptions:

Deviation in flow rates for standard pumping mode in V3 pump are 1 – 2%, all pumps were calibrated and checked according to manual provided from Vapourtec. HFCs were pumped using standard mode, stoichiometry was calculated by cylinder pressure and gas flow rate. Input Residence Time is the residence time for stoichiometry calculation; they are denoted as Res. Time in tables. The observed residence time was close to and slightly longer than residence time calculated only accounting liquid flow rate, as most of the gases were consumed or dissolved with a change in physical state from gas to liquid or solid.

Total defluorination is assumed where no soluble fluorine product is observed in the ^{19}F NMR spectra. Maximum defluorination yield in KF% is 400%, representing removal of 4 moles of fluorine atoms from 1 mole of HFC-134a. KF production rate is calculated with regards to liquid flow rate.

13.3. Flow Chemistry Setup Overview:

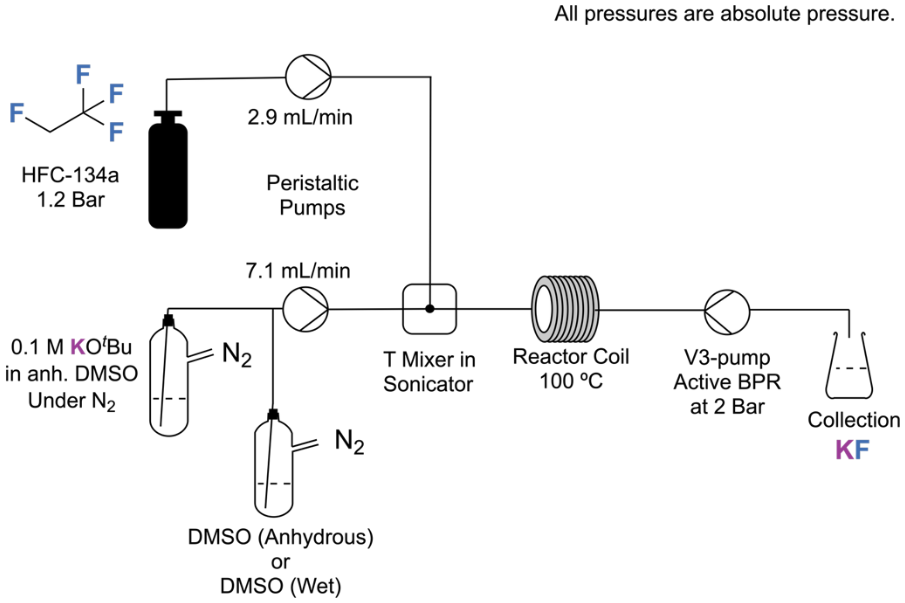

Supplementary Figure 83: Overview of Flow Reaction System Setup

13.4. Temperature and Residence Time Study for Total Defluorination of HFC-134a:

In a dinitrogen containing glovebox, a 0.1 M standard solution of KO*t*Bu (280.5 mg, 2.5 mmol) in anhydrous DMSO (25 mL) was prepared to obtain a slightly cloudy solution. The solution was transferred into a Schlenk flask, connected to the Vapourtec via a 19-gauge stainless steel luer lock needle (1.07 mm OD) and 1/16'' OD FEP tubing was used (ID = 1 mm) and the line was primed. KO*t*Bu solution was connected as reagent and anhydrous DMSO in an ampoule was connected as solvent.

Prior to the reaction, the reactor (Vapourtec E3 PFA tube reactor, 10 mL) was set to the desired reaction temperature (25 – 100 °C) and was primed with both DMSO and HFC-134a (1.2 Bar) for at least 1 reactor volume. After which the DMSO delivery was paused and the KO*t*Bu solution was delivered, the solution was mixed with HFC-134a at a T-junction. During the reaction, a brown - black solution was observed with decreasing amount of gas bubbles overtime. The initial 10 - 12 mL of reaction mixture was discarded and 4 x 1 mL aliquots of reaction mixture was collected using 1 mL volumetric flasks. The aliquots were decanted, and washed with 3 – 4 times of 1 mL deionised water. 0.1 mL of 0.5 M or 0.1 mL of 1 M NaOTf (in deionised water) was added as internal standard for quantitative ^{19}F NMR spectroscopic analysis.

Supplementary Table 15: Temperature and residence time data for total defluorination of HFC-134a

Temp. [°C]	Res. Time [min]*	Sol. Flow Rate [mL/min]	Gas Flow Rate [mL/min]	Mass KF per hour [g/h]	KF % (Max 400%) [%]	Incomplete Defluorination [Info]	KF Space Time Yield [gL-1h-1]
100	15	0.47	0.20	0.099	302%	None	9.9
100	5	1.42	0.58	0.342	345%	None	34.2
100	1	7.08	2.92	1.547	313%	Trace	154.7
80	5	1.42	0.58	0.310	329%	Trace	31.0
25	5	1.42	0.58	0.283	290%	Noticeable	28.3

*Res. Time is residence time input for stoichiometry calculation, not observed residence time.

13.5. Direct *in situ* Transfer-fluorination:

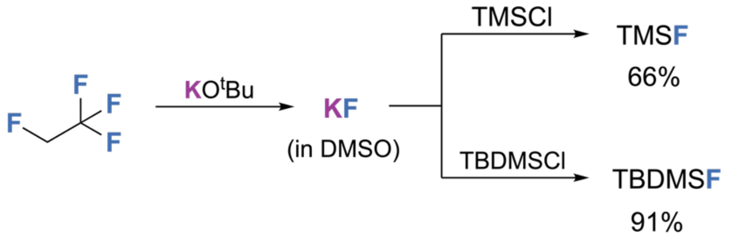

Supplementary Figure 84: Direct in situ Transfer-fluorination of KF Produced from Flow

<u>Fluorination of trimethylsilyl chloride with KF in DMSO from flow:</u>

In a dinitrogen containing glovebox, a 1 M standard solution of KO*t*Bu (2.81 g, 25 mmol) in anhydrous DMSO (25 mL) was prepared to obtain a slightly cloudy solution. The solution was transferred into a Schlenk flask, connected to Vapourtec via a 19-gauge stainless steel luer lock needle (1.07 mm OD) and 1/16'' OD FEP tubing was used (ID = 1 mm) and the line was primed. KO*t*Bu solution was connected as reagent and anhydrous DMSO in an ampoule was connected as solvent.

Prior to the reaction, the reactor (495 cm, 3.88 mL) was placed in a sonicator bath and primed for at least 1 reactor volume with DMSO and HFC-134a at a flow rate of 0.758 mL/min and 3.13 mL/min respectively, BPR pressure was set to 1 Bar (Gauge pressure). After which the DMSO delivery was paused and the KO*t*Bu solution was delivered, the solution was mixed with HFC-134a at a T-junction. During the reaction, a brown - black solution was observed with decreasing amount of gas bubbles overtime. The initial 10 - 12 mL of reaction mixture was discarded and 4 x 1 mL aliquots of reaction mixture was collected using 1 mL volumetric flasks. The aliquots were decanted, and washed with 3 – 4 times of 1 mL deionised water. 0.1 mL of 0.5 M or 0.1 mL of 1 M NaOTf (in deionised water) was added as internal standard for quantitative ^{19}F NMR spectroscopic analysis. The rest of the collected mixtures was assumed to have the same KF content for subsequent transfer-fluorination. The mixture was sonicated for 2 min and 0.5 mL was taken for quantitative ^{19}F NMR. The KF content per 1 mL reaction mixture collected was determined to be 0.562 mmol.

One of the remaining 1 mL aliquot was sonicated for 1 min and transferred to a J Young NMR tube. Under air, trimethylsilyl chloride (71.3 µL, 0.562 mmol) was added to the mixture and heated for at 100 °C for 1 h. A black solution with suspension was obtained and the yield of trimethylsilyl fluoride was determined as 66% using ^{19}F NMR.

^{19}F NMR (376 MHz, DMSO-h_6, 25°C) δ 155.2 (s, 1F, SiF).

Fluorination of tert-butyldimethylsilyl chloride with KF in DMSO from flow:

In a dinitrogen containing glovebox, a 1 M standard solution of KO*t*Bu (2.81 g, 25 mmol) in anhydrous DMSO (25 mL) was prepared to obtain a slightly cloudy solution. The solution was transferred into a Schlenk flask, connected to Vapourtec via a 19-gauge stainless steel luer lock needle (1.07 mm OD) and 1/16'' OD FEP tubing was used (ID = 1 mm) and the line was primed. KO*t*Bu solution was connected as reagent and anhydrous DMSO in an ampoule was connected as solvent.

Prior to the reaction, the reactor (495 cm, 3.88 mL) was placed in a sonicator bath and primed for at least 1 reactor volume with DMSO and HFC-134a at a flow rate of 0.758 mL/min and 3.13 mL/min respectively, BPR pressure was set to 3 Bar (Gauge pressure). After which the DMSO delivery was paused and the KO*t*Bu solution was delivered, the solution was mixed with HFC-134a at a T-junction. During the reaction, a brown - black solution was observed with decreasing amount of gas bubbles overtime. The initial 10 - 12 mL of reaction mixture was discarded and 4 x 1 mL aliquots of reaction mixture was collected using 1 mL volumetric flasks. The aliquots were decanted, and washed with 3 – 4 times of 1 mL deionised water. 0.1 mL of 0.5 M or 0.1 mL of 1 M NaOTf (in deionised water) was added as internal standard for quantitative ^{19}F NMR spectroscopic analysis. The rest of the collected mixtures was assumed to have the same KF content for subsequent transfer-fluorination. The mixture was sonicated for 2 min and 0.5 mL was taken for quantitative ^{19}F NMR. The KF content per 1 mL reaction mixture collected was determined to be 0.6 mmol.

One of the remaining aliquot was sonicated for 1 min and transferred to a J Young NMR tube. Under air, 1 mL DMSO was added to tert-butyldimethylsilyl chloride (90.4 mg, 0.6 mmol) and sonicated. The resultant suspension was subsequently transferred to the J Young NMR tube, and heated at 100 °C for 1 h. A black suspension was obtained and the yield of tert-butyldimethylsilyl fluoride was determined as 91% using ^{19}F NMR yield.

^{19}F NMR (376 MHz, DMSO-h_6, 25°C) δ 169.1 (s, 1F, SiF).

^{19}F spectroscopy data in accordance with literature, shifted due to solvent use[66].

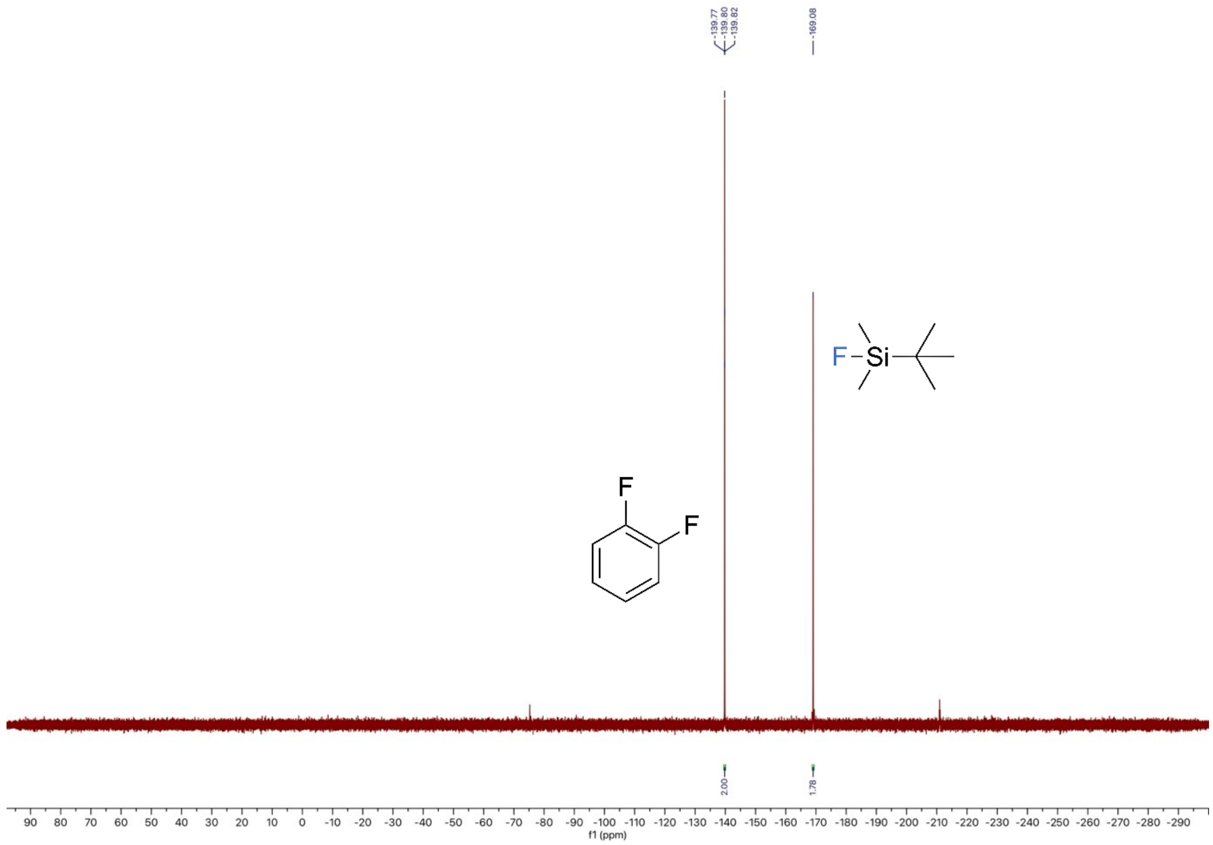

Supplementary Figure 85: ^{19}F NMR Spectra of tert-butyldimethylsilyl fluoride prepared from KF generated under flow conditions.

13.6. Isolation of KF from Flow:

In a dinitrogen containing glovebox, a 1 M standard solution of KO*t*Bu (2.81 g, 25 mmol) in anhydrous DMSO (25 mL) was prepared to obtain a slightly cloudy solution. The solution was transferred into a Schlenk flask and connected to Vapourtec via a 19-gauge stainless steel luer lock needle (1.07 mm OD) and 1/16'' OD FEP tubing was used (ID = 1 mm). The solution was transferred into a Schlenk flask, connected to Vapourtec via a 19-gauge stainless steel luer lock needle (1.07 mm OD) and 1/16'' OD FEP tubing was used (ID = 1 mm) and the line was primed. KO*t*Bu solution was connected as reagent and anhydrous DMSO in an ampoule was connected as solvent.

Prior to the reaction, the reactor (495 cm, 3.88 mL) was placed in a sonicator bath and primed for at least 1 reactor volume with DMSO and HFC-134a at a flow rate of 0.758 mL/min and 3.13 mL/min respectively, BPR pressure was set to 1 Bar (Gauge pressure). After which the DMSO delivery was paused and the KO*t*Bu solution was delivered, the solution was mixed with HFC-134a at a T-junction Observed decreasing amount of gas along the reaction coil and generated brown - black solution. The initial 10 – 12 mL reaction mixture was discarded, and the subsequent 10 mL aliquot was collected for KF isolation and purification.

Supplementary Table 16: Data obtained from the flow reaction of HFC-134a using KO*t*Bu

**Temp. [°C]**	**Coil Vol. [mL]**	**Res. Time [min]**	**Sol. Flow Rate [mL/min]**	**Gas Flow Rate [mL/min]**	**Incomplete Defluorination [Info]**
RT	3.88	1	0.758	3.13	Multiple signal, noticable

<u>KF was further purified using the following method:</u>

Method 1: 10 mL of reaction mixture was collected, centrifuged for 10 min (3260 x g). The supernatant solution was removed leaving a brown-black solid that was dried by heating at 100 °C under vacuum (0.01 mbar). The solid obtained through this route was found to be deliquescent and storage in air for prolonged periods should be avoided.

Method 2: 10 mL of reaction mixture was collected, centrifuged for 10 min (3260 x g). The liquid phase was decanted from the solid. The solid was re-suspended in 15 ml of acetone, sonicated and centrifuged for 5 min (3260 x g). The supernatant was again decanted and the resulting solid, washed

with acetone (3 times x 10 mL) each time decanting a lighter brown/yellow liquid. The obtained solid was dried by heating under reduced pressure (0.01 mbar) at 100 °C for 30 min, to obtain beige colour powder.

Method 3: 10 mL of reaction mixture was collected, centrifuged for 10 min (3260 x g). The liquid phase was decanted from the solid. The solid was re-suspended in 15 ml of acetone, sonicated and centrifuged for 5 min (3260 x g). The supernatant was again decanted and the resulting solid, washed with acetone (3 times x 10 mL) each time decanting a lighter brown/yellow liquid. The obtained solid was re-dissolved in water and freeze-dried (0.04 mbar) for 48 hours. Obtained flakes of white and brown solids.

CHN Elemental Analysis Result for Isolated KF from flow:

In all attempts of KF production from flow, trace or noticeable amount of side products was observed in ^{19}F NMR spectrum. Direct removal of DMSO from method one was expected to have residual DMSO along with high boiling point insoluble hydrocarbon or hydrofluorocarbon potassium salt. Addition of acetone wash and freeze-drying procedure in method 3 found effective in removal of some soluble carbon side product. From method 3, the overall 8.7% carbon and hydrogen content was believed to be solid inorganic salts and water, with the remainder 91.3% being KF.

Supplementary Table 17: CHN Elemental Analysis of KF obtained through flow reactions with HFC-134a.

**Isolation Method**	**% Carbon**	**% Hydrogen**	**% Nitrogen**
Method 1 KF	7.58%	4.24%	0%
Method 3 KF	4.69%	4.01%	0%

Solid State NMR:

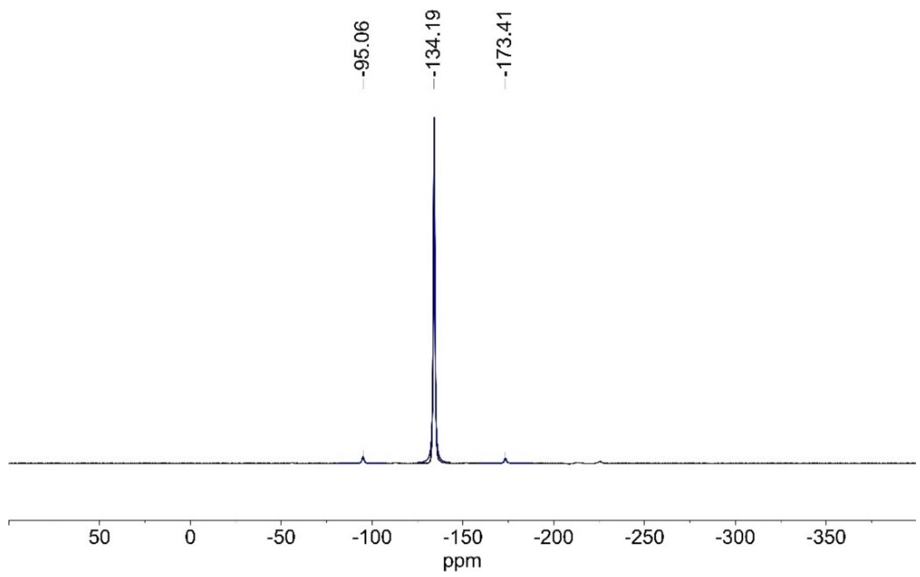

Supplementary Figure 86: MAS-SS ^{19}F NMR spectrum of isolated KF solid via Method 2 using a 2.5 mm rotor. The spectrum contains a single peak corresponding to KF with trace of side product at –225 ppm.

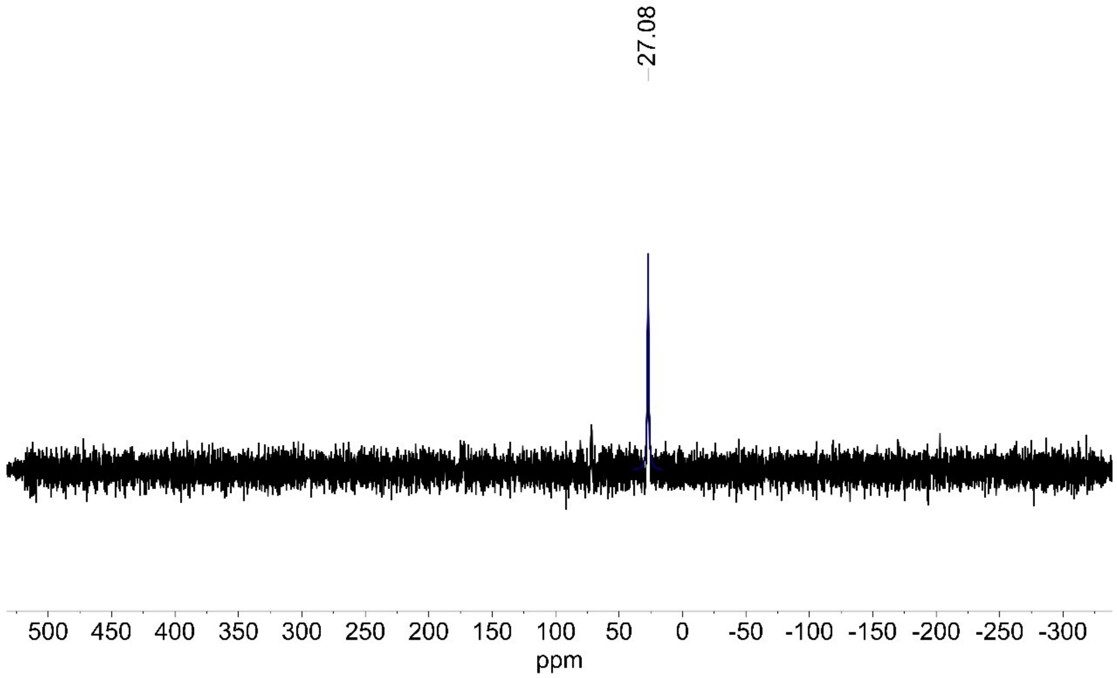

Supplementary Figure 87: MAS-SS $^{13}C/^{1}H$ NMR spectrum of isolated KF solid via Method 2 using a 2.5 mm rotor. The spectrum contains a major peak at 27.08. With trace of other product at –81 ppm.

13.7. Transfer-fluorination with Isolated KF from Flow:

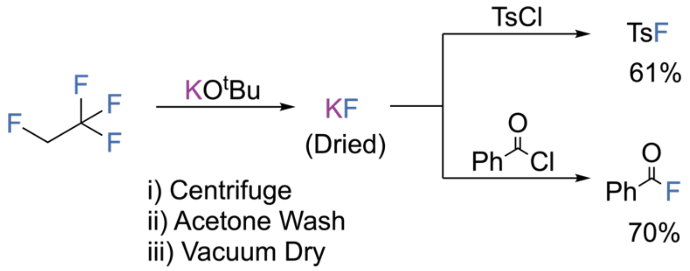

Supplementary Scheme 62: Transfer-fluorination with Isolation KF from Flow.

Fluorination of benzoyl chloride with dried KF from flow:

In a dinitrogen containing glovebox, KF (0.41 mmol, 23.6 mg) obtained from the defluorination of HFC-134a in flow and purified by **method 2**, was suspended in 1 mL THF and transferred to a J Young NMR tube. Benzoyl chloride (24 µL, 0.2 mmol) was added to the mixture, followed by 1,2-difluorobenzene (10 µL, 0.1 mmol) as internal standard. The reaction mixture was heated for 24 h to give 70% benzoyl fluoride based on comparison to the internal standard.
^{19}F NMR (376 MHz, THF-h_8, 25°C): δ 16.3 (s, 1F, C(O)F).

^{19}F spectroscopy data in accordance with literature[67].

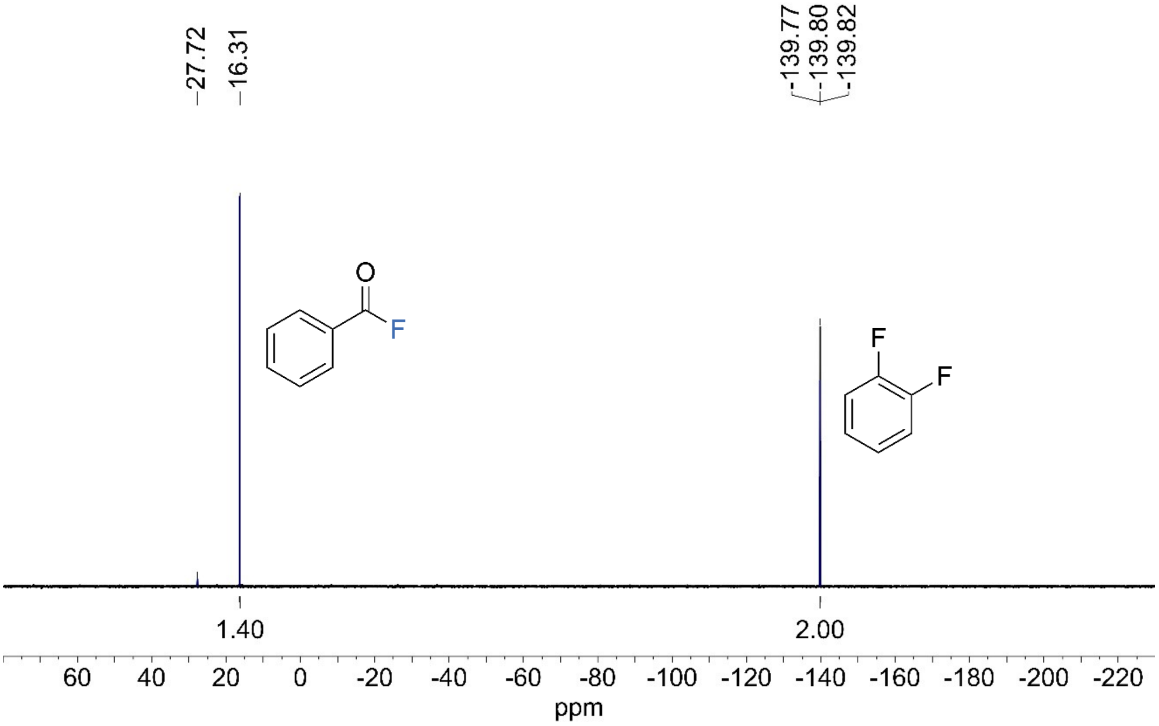

Supplementary Figure 88: ^{19}F NMR Spectra of benzoyl fluoride prepared from KF derived from HFC-134a using flow conditions.

Fluorination of tosyl chloride with dried KF from flow:

In a dinitrogen containing glovebox, KF (0.39 mmol, 22.5 mg) obtained from the defluorination of HFC-134a in flow and purified by **method 2**, was suspended in 1 mL THF and transferred to a J Young NMR tube. Tosyl chloride (0.2 mmol, 39.1 mg) was added to the mixture, followed by 1,2-difluorobenzene (10 µL, 0.1 mmol) as internal standard. The reaction mixture was heated for 1 h to yield 61% tosyl fluoride based on comparison to the internal standard.

^{19}F NMR (376 MHz, THF-h8, 25°C): δ 65.6 (s, 1F, SO_2F).

14. NMR spectra

14.1. NMR spectra of species **1a**, **2a** and **1d**

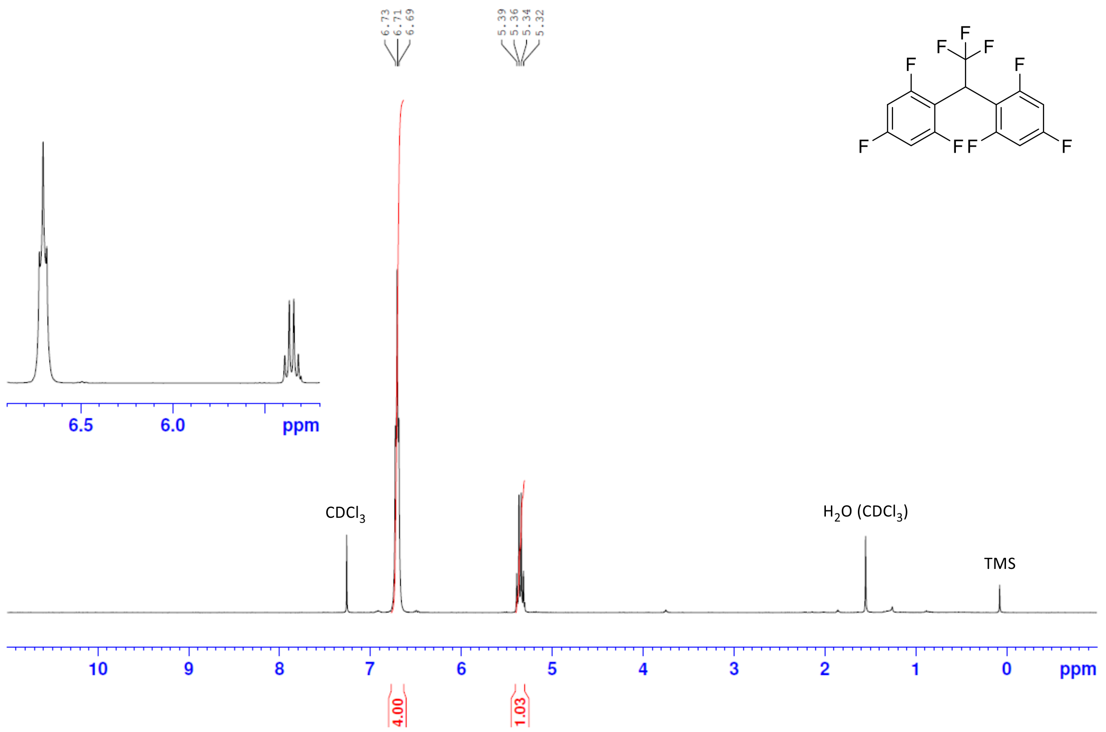

^{1}H NMR spectrum of 1,1,1-trifluor-2,2-bis(2,4,6-trifluorphenyl)-ethane **1a** (400 MHz, $CDCl_3$, 25 °C).

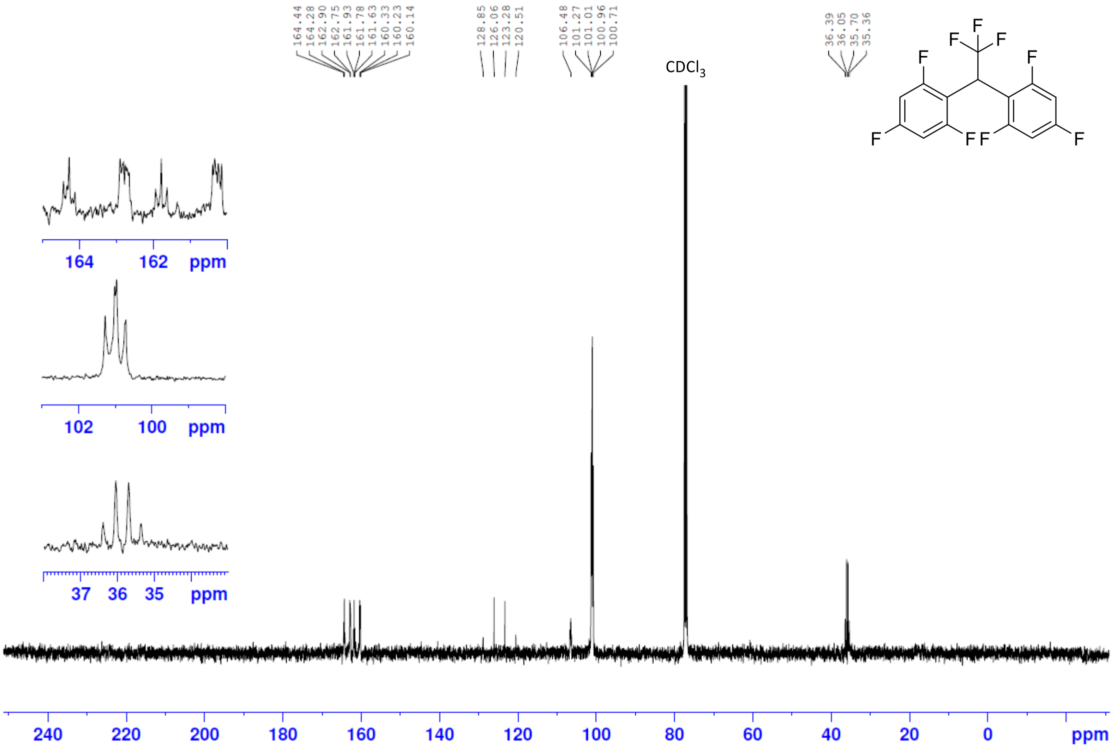

$^{13}C\{^{1}H\}$ NMR spectrum of 1,1,1-trifluor-2,2-bis(2,4,6-trifluorphenyl)-ethane **1a** (101 MHz, $CDCl_3$, 25 °C).

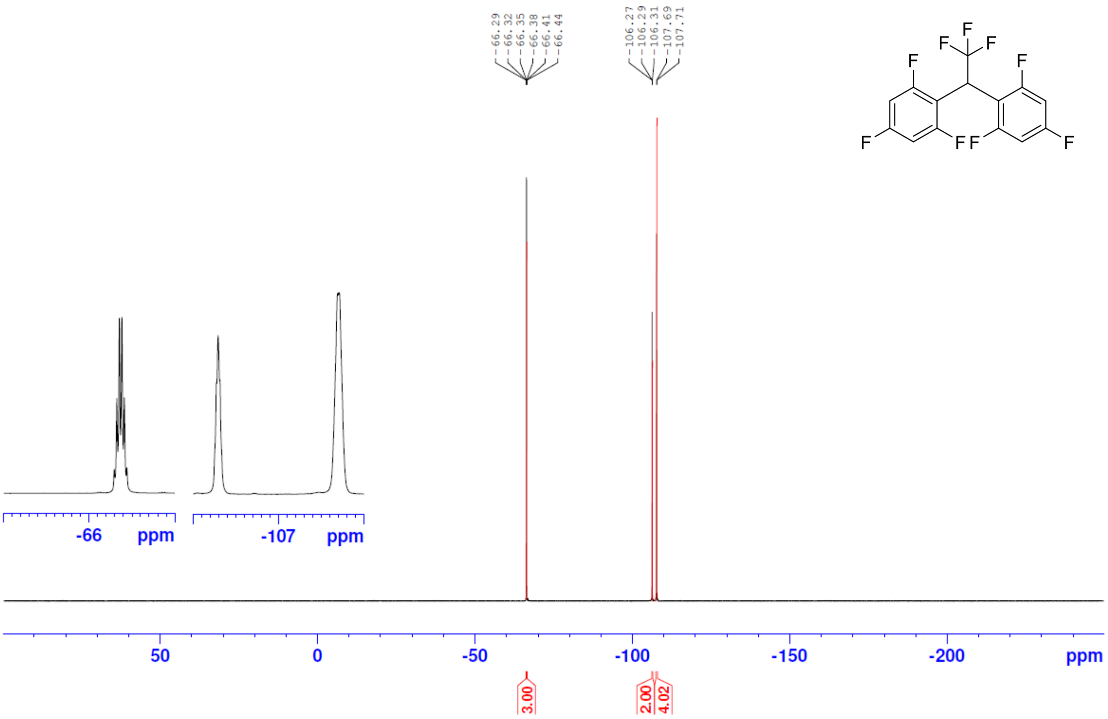

Quantitative ^{19}F NMR spectrum of 1,1,1-trifluor-2,2-bis(2,4,6-trifluorphenyl)-ethane **1a** (377 MHz, $CDCl_3$, 25 °C).

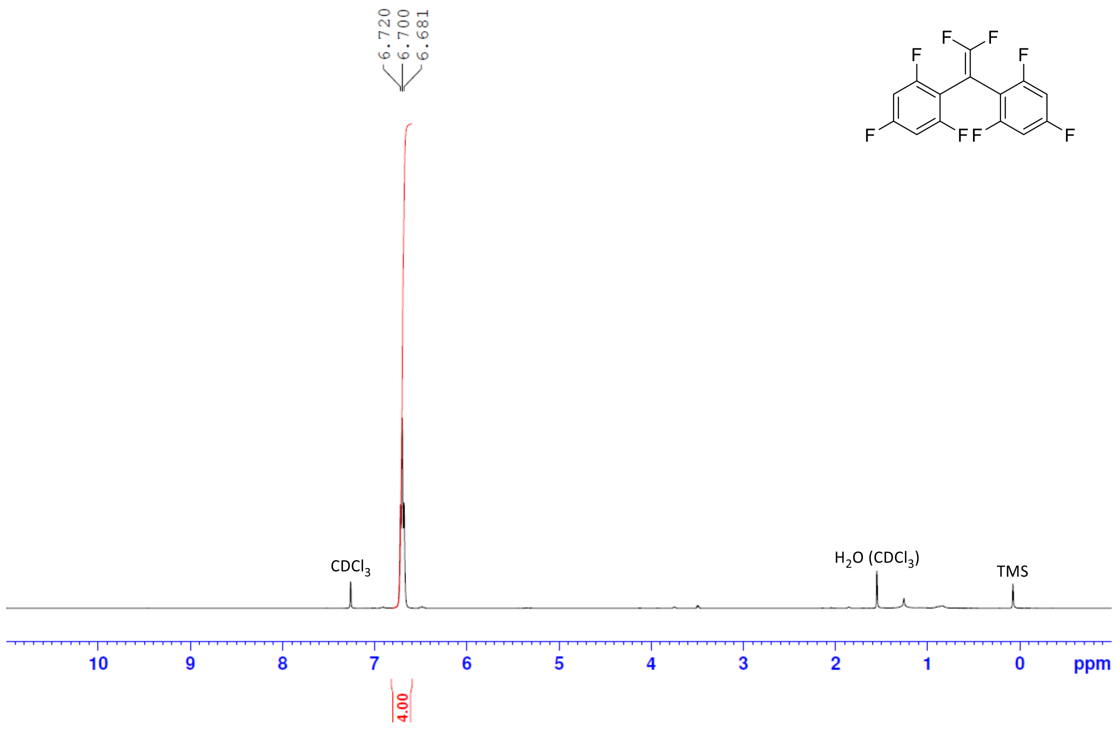

^{1}H NMR spectrum of 1,1-difluor-2,2-bis(2,4,6-trifluorphenyl)-ethene **2a**
(400 MHz, $CDCl_3$, 25 °C).

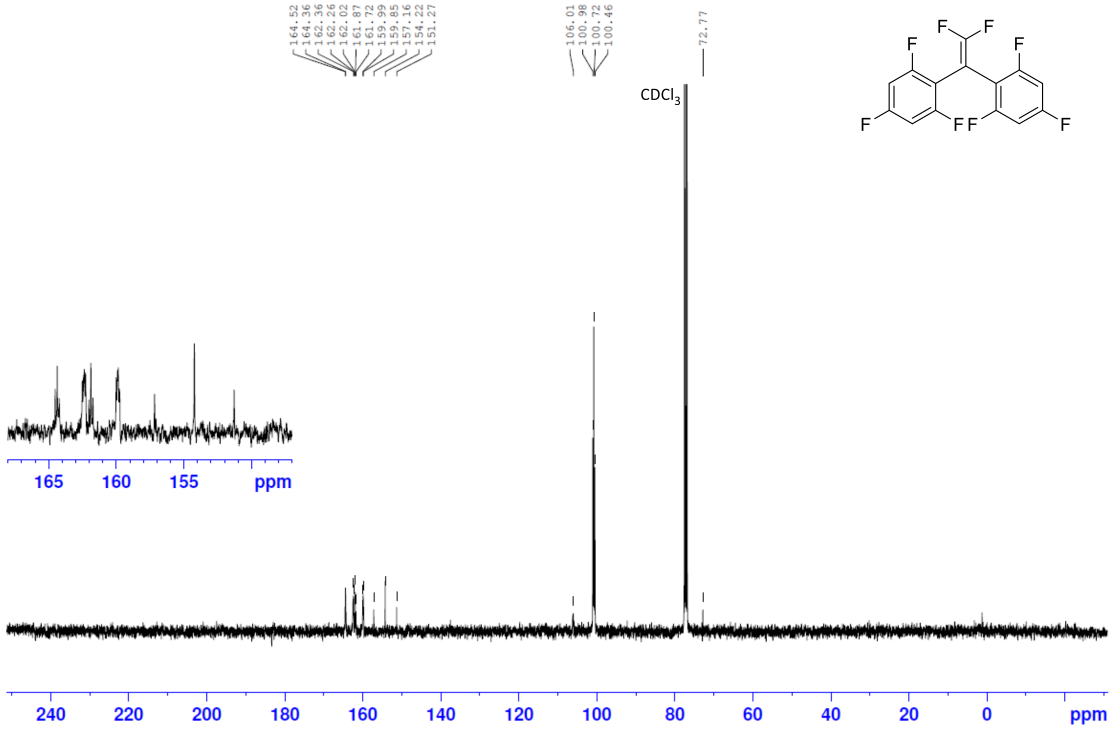

^{13}C{^{1}H} NMR spectrum of 1,1-difluor-2,2-bis(2,4,6-trifluorphenyl)-ethene **2a**
(101 MHz, $CDCl_3$, 25 °C).

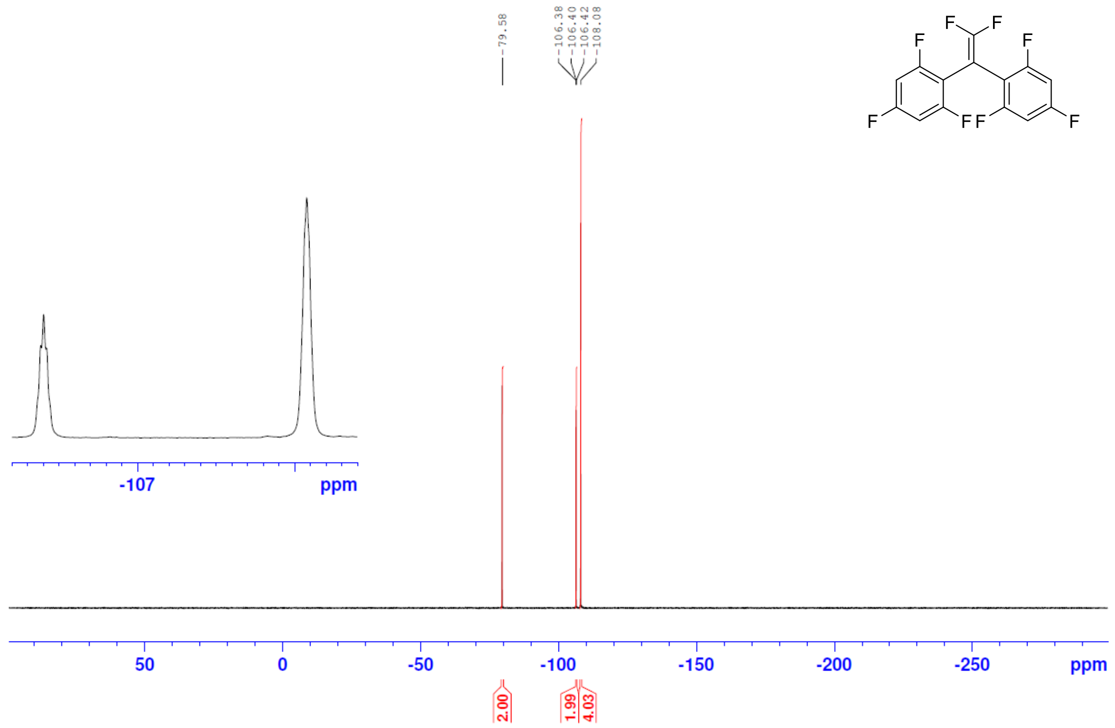

Quantitative ^{19}F NMR spectrum of 1,1-difluor-2,2-bis(2,4,6-trifluorphenyl)-ethene **2a** (377 MHz, $CDCl_3$, 25 °C).

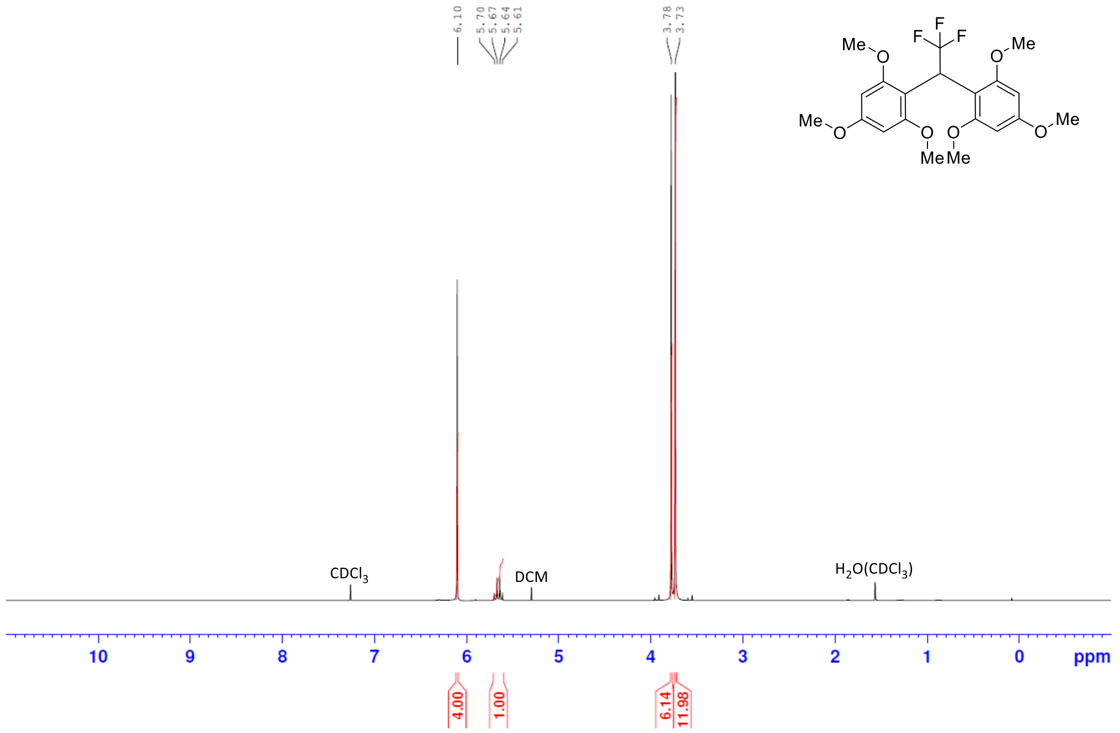

^{1}H NMR spectrum of 1,1,1-trifluor-2,2-bis(2,4,6-methoxyphenyl)-ethane **1d** (400 MHz, $CDCl_3$, 25 °C).

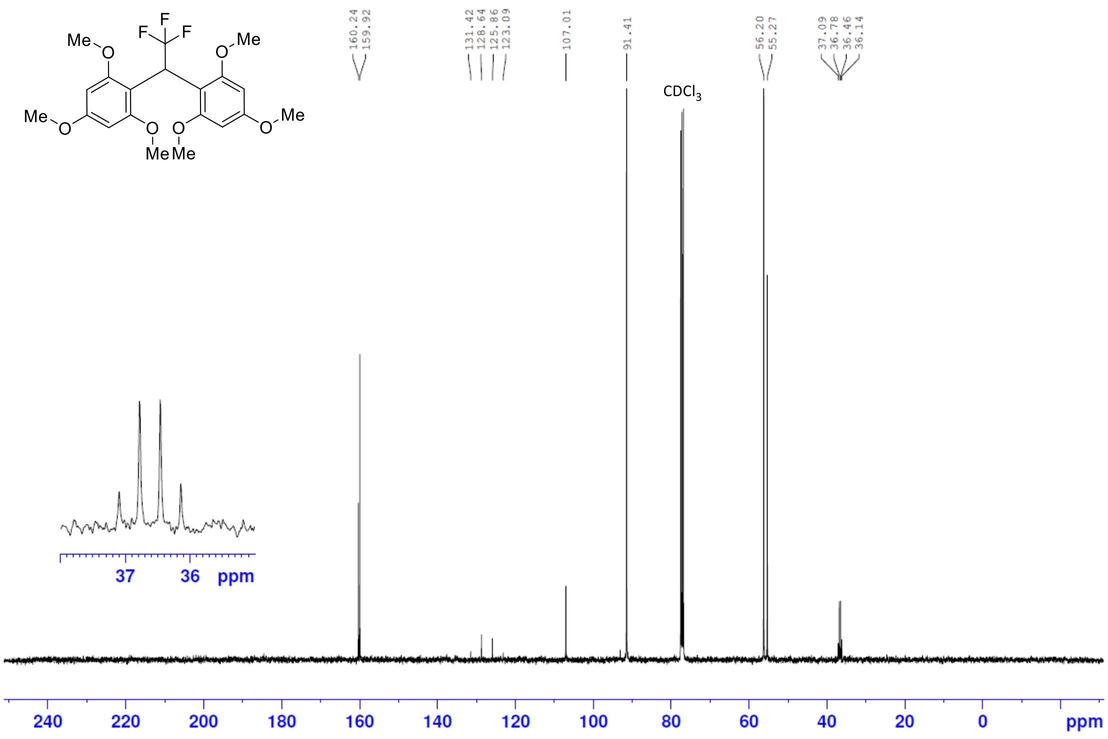

$^{13}C\{^1H\}$ NMR spectrum of 1,1,1-trifluor-2,2-bis(2,4,6-methoxyphenyl)-ethane **1d**
(101 MHz, $CDCl_3$, 25 °C).

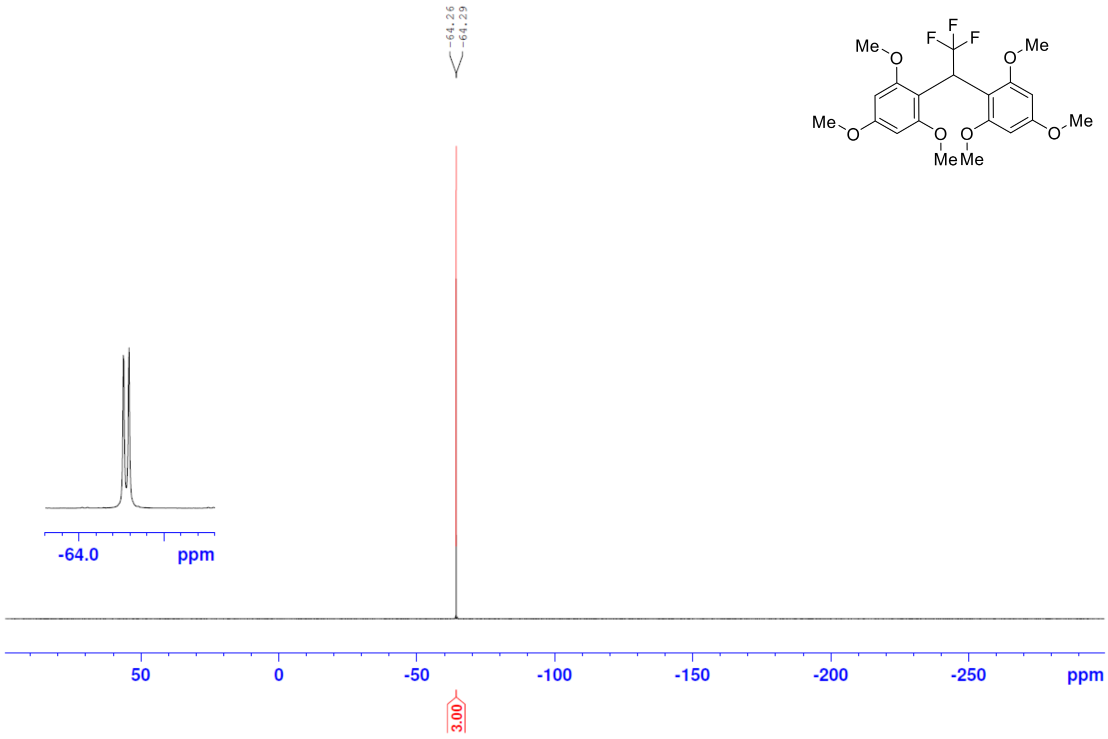

^{19}F NMR spectrum of 1,1,1-trifluor-2,2-bis(2,4,6-methoxyphenyl)-ethane **1d**
(377 MHz, $CDCl_3$, 25 °C).

14.2. NMR spectra of fluorinated products

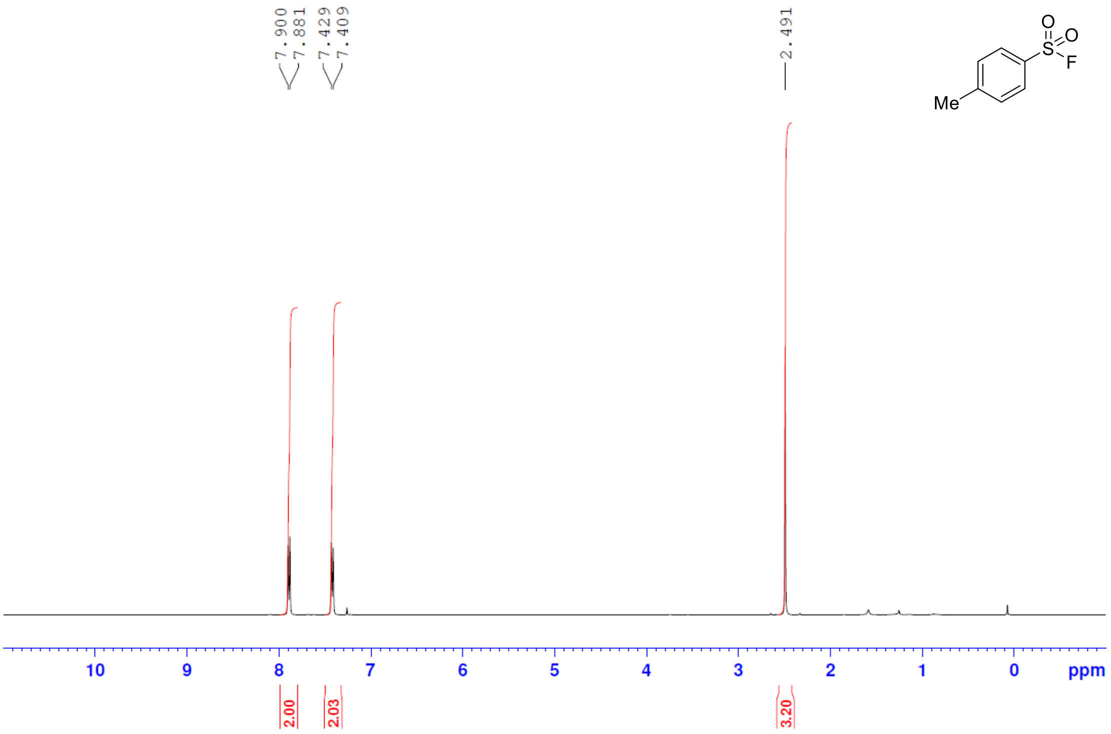

^{1}H NMR spectrum of tosyl fluoride **3a** (400 MHz, $CDCl_3$, 25 °C).

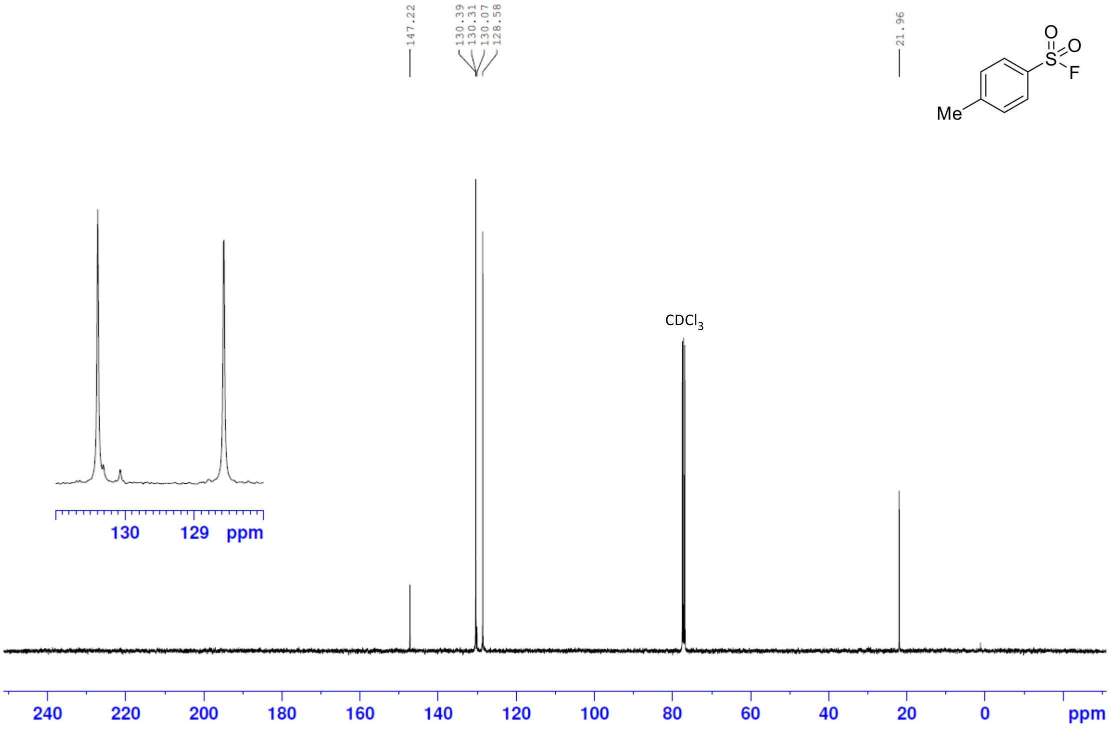

$^{13}C\{^{1}H\}$ NMR spectrum of tosyl fluoride **3a** (101 MHz, $CDCl_3$, 25 °C).

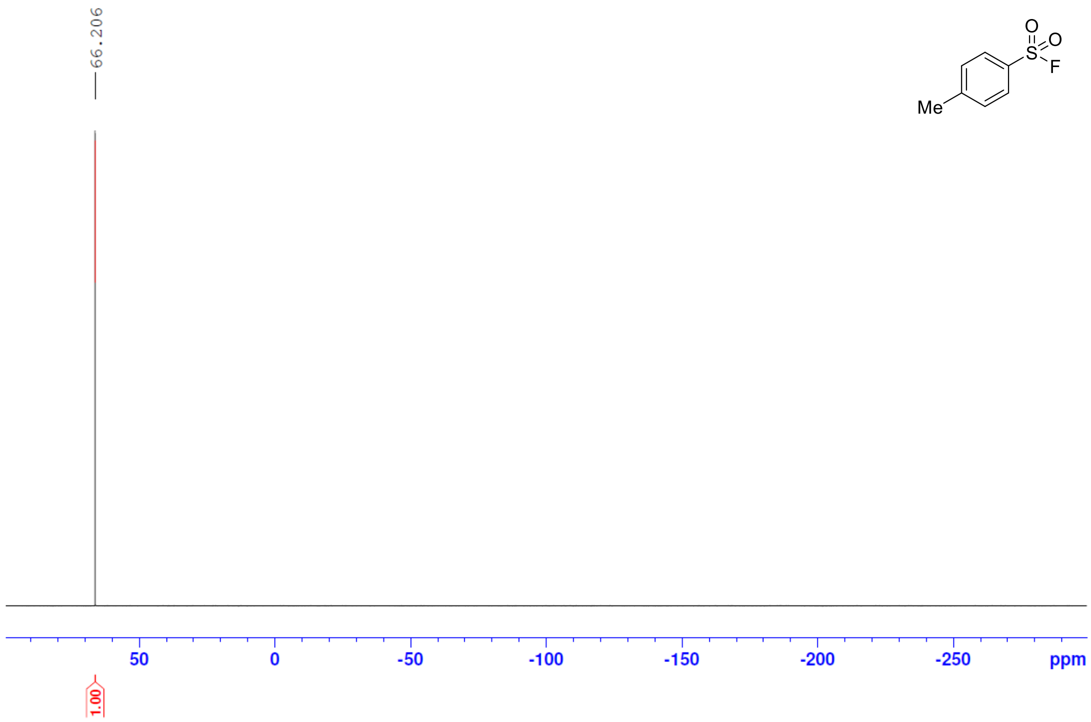

^{19}F NMR spectrum of tosyl fluoride **3a** (377 MHz, $CDCl_3$, 25 °C).

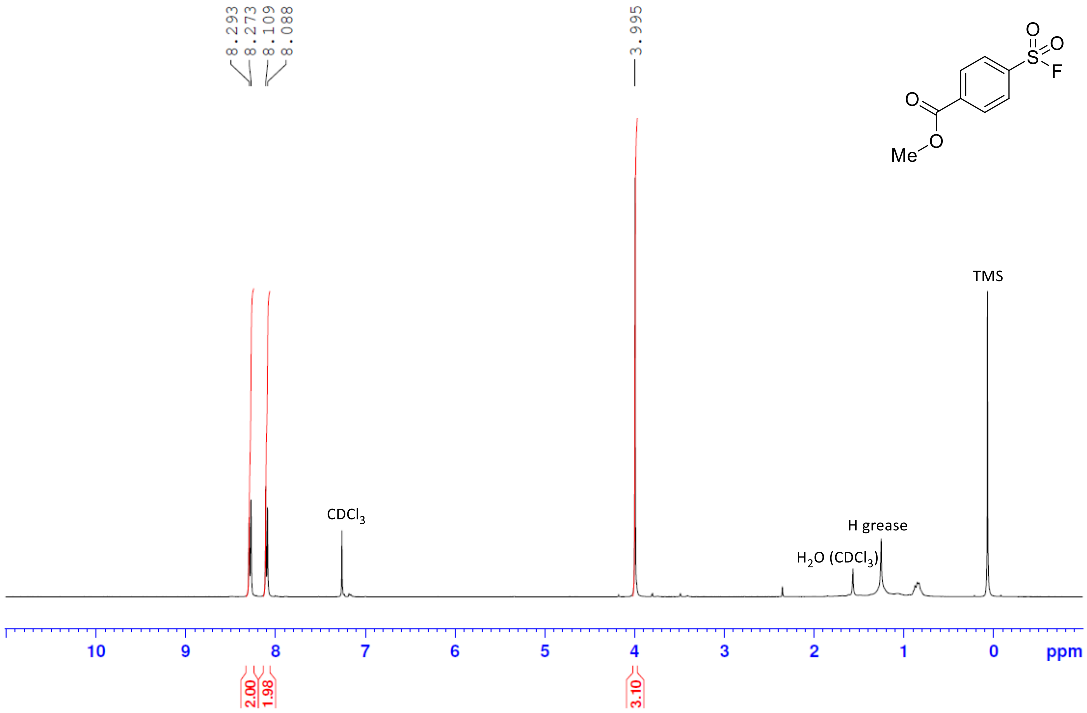

^{1}H NMR spectrum of methyl 4-(fluorosulfonyl)benzoate **3b** (400 MHz, $CDCl_3$, 25 °C).

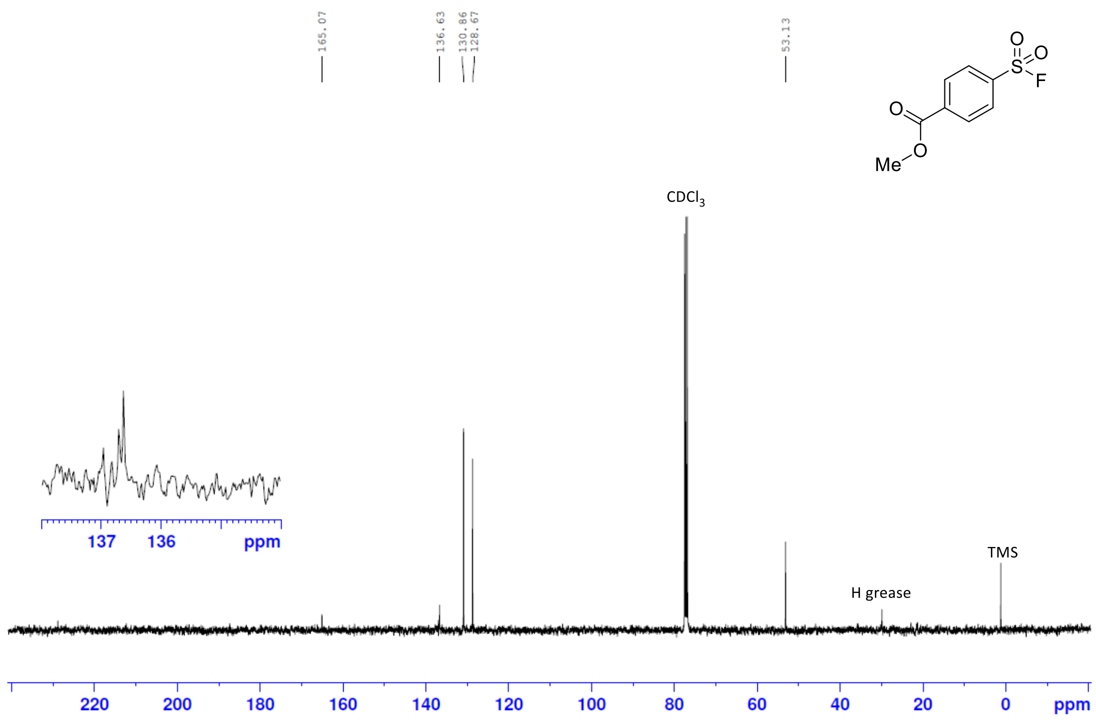

^{13}C{^{1}H} NMR spectrum of methyl 4-(fluorosulfonyl)benzoate **3b** (101 MHz, $CDCl_3$, 25 °C).

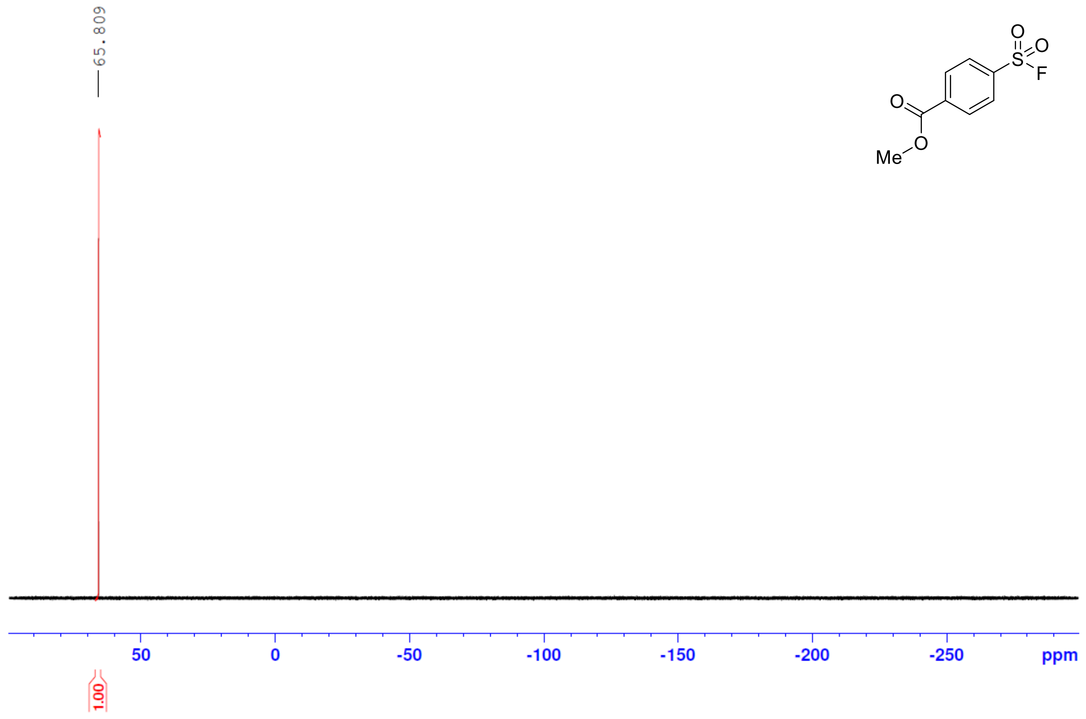

^{19}F NMR spectrum of methyl 4-(fluorosulfonyl)benzoate **3b** (377 MHz, $CDCl_3$, 25 °C).

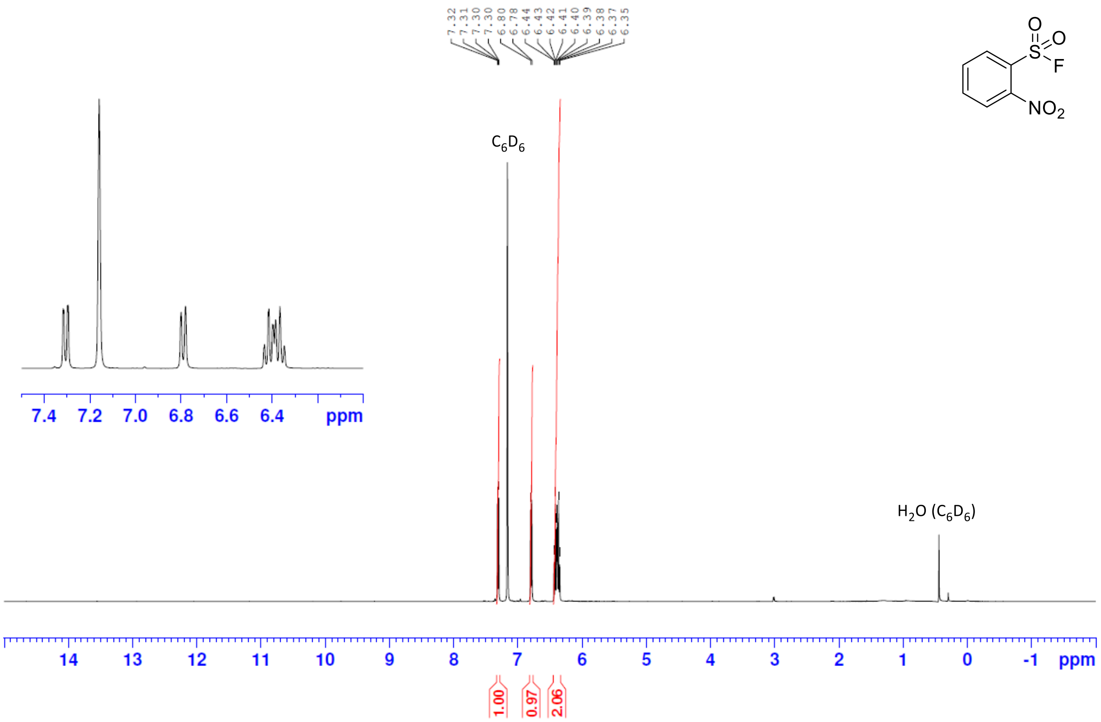

^{1}H NMR spectrum of 2-nitrobenzenesulfonyl fluoride **3c** (400 MHz, C_6D_6, 25 °C).

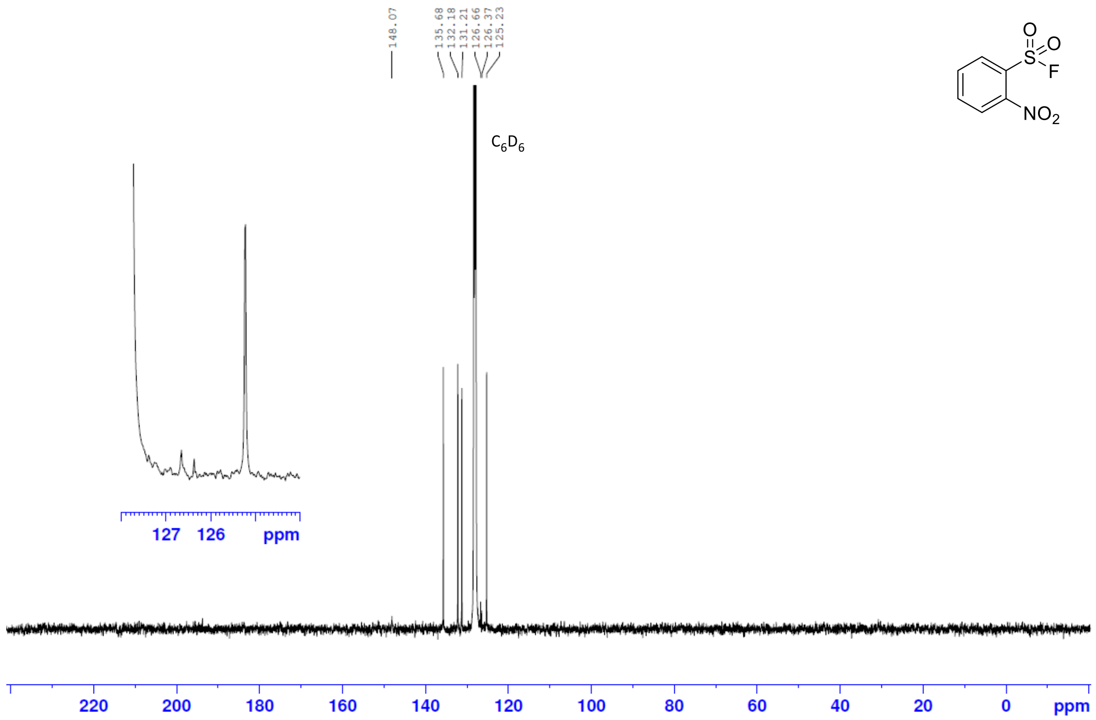

^{13}C{^{1}H} NMR spectrum of 2-nitrobenzenesulfonyl fluoride **3c** (101 MHz, C_6D_6, 25 °C).

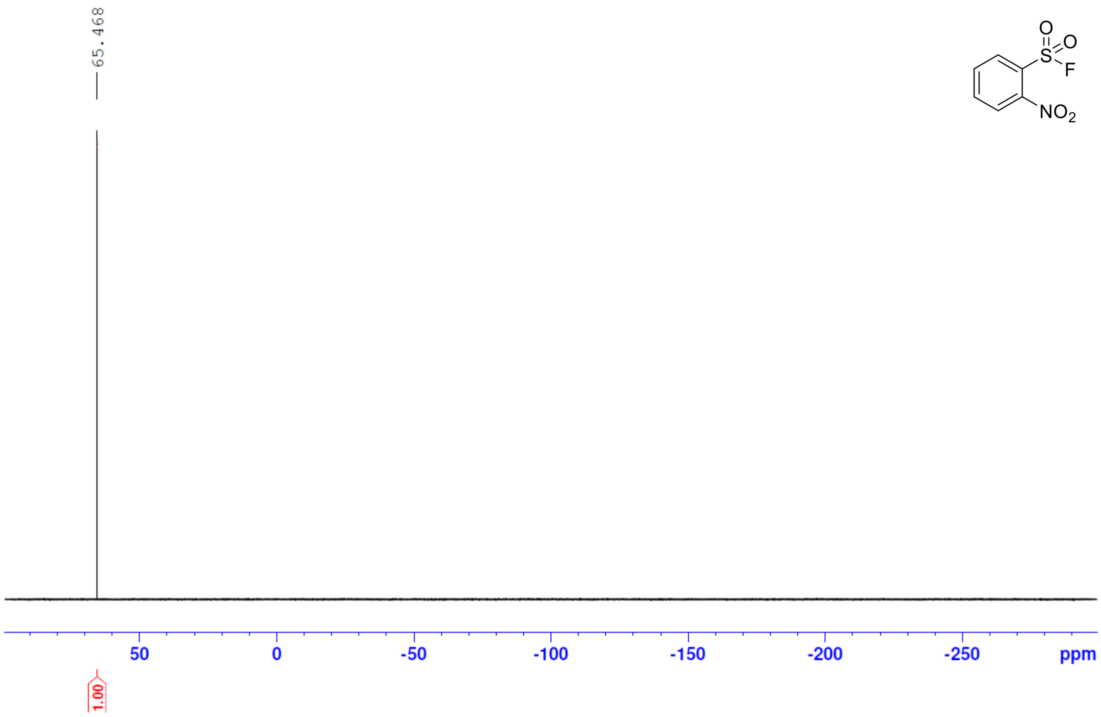

^{19}F NMR spectrum of 2-nitrobenzenesulfonyl fluoride **3c** (377 MHz, C_6D_6, 25 °C).

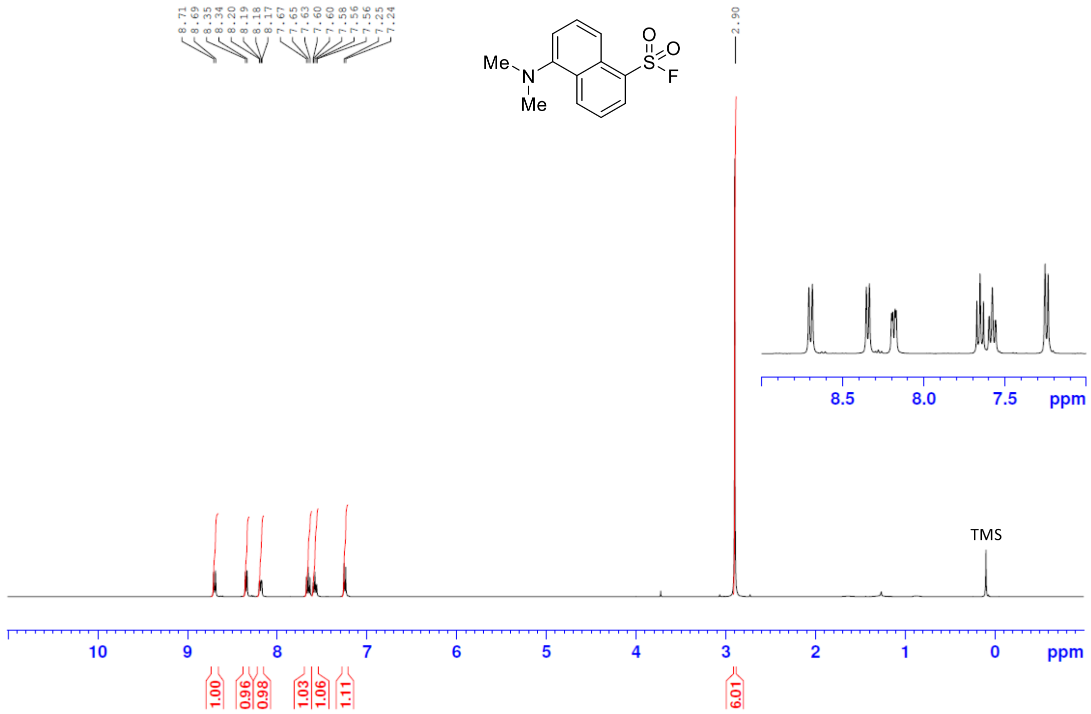

^{1}H NMR spectrum of dansyl fluoride **3d** (400 MHz, $CDCl_3$, 25 °C).

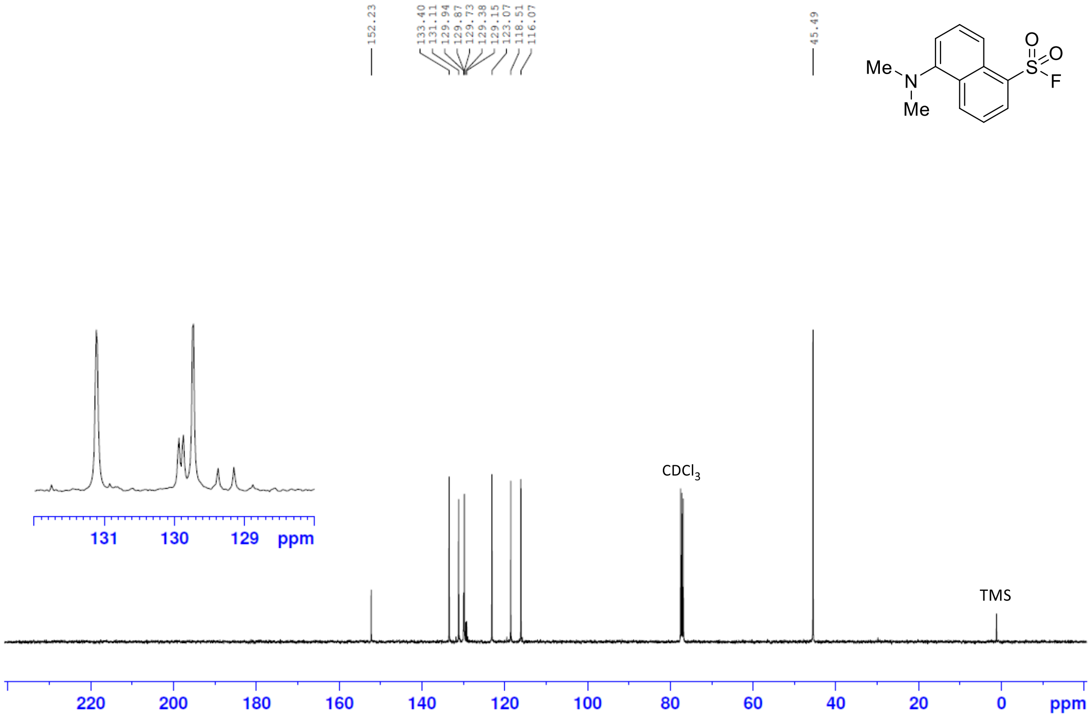

$^{13}C\{^{1}H\}$ NMR spectrum of dansyl fluoride **3d** (101 MHz, $CDCl_3$, 25 °C).

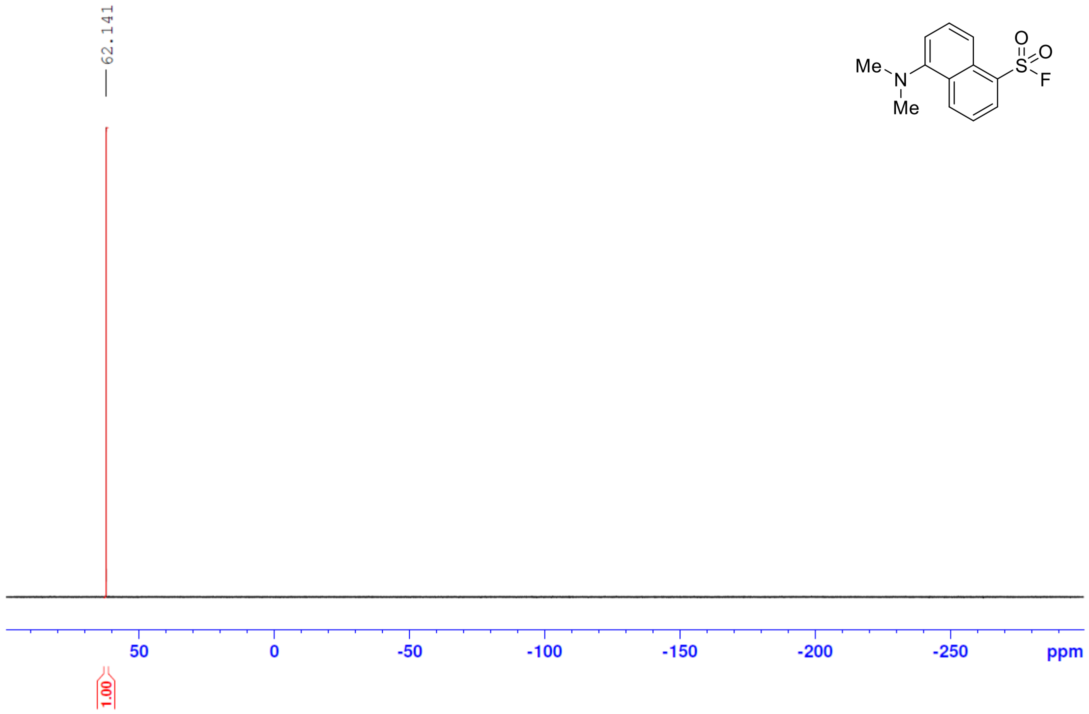

^{19}F NMR spectrum of dansyl fluoride **3d** (377 MHz, $CDCl_3$, 25 °C).

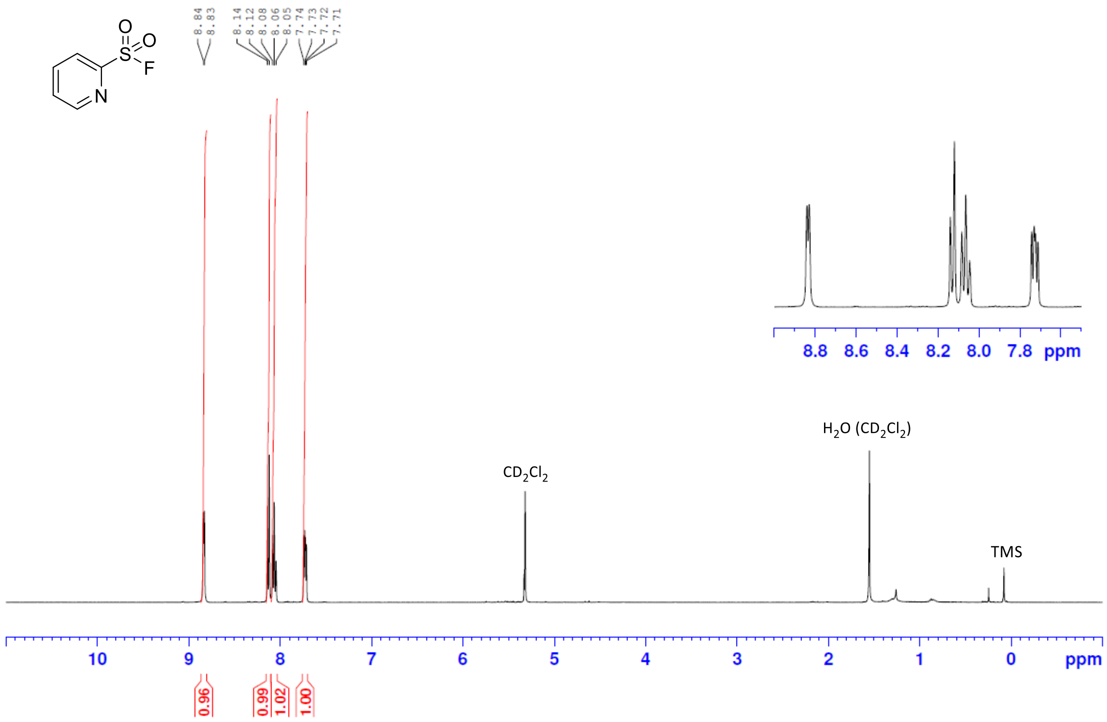

^{1}H NMR spectrum of pyridine-2-sulfonyl fluoride **3e** (400 MHz, CD_2Cl_2, 25 °C).

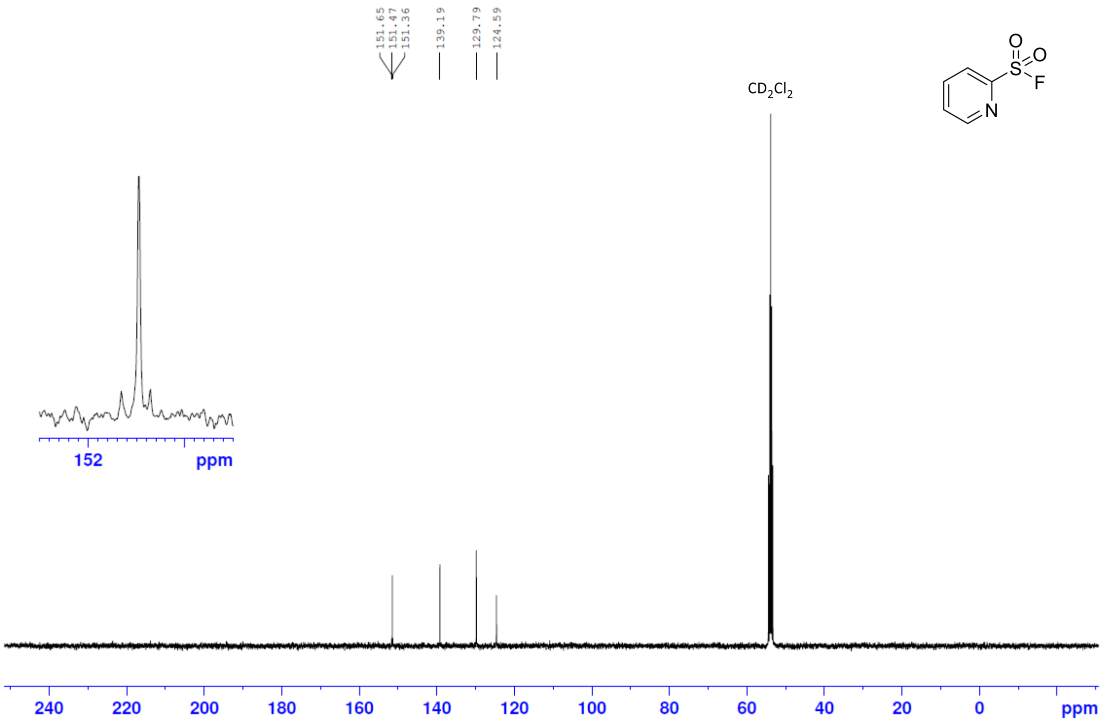

$^{13}C\{^{1}H\}$ NMR spectrum of pyridine-2-sulfonyl fluoride **3e** (101 MHz, CD_2Cl_2, 25 °C).

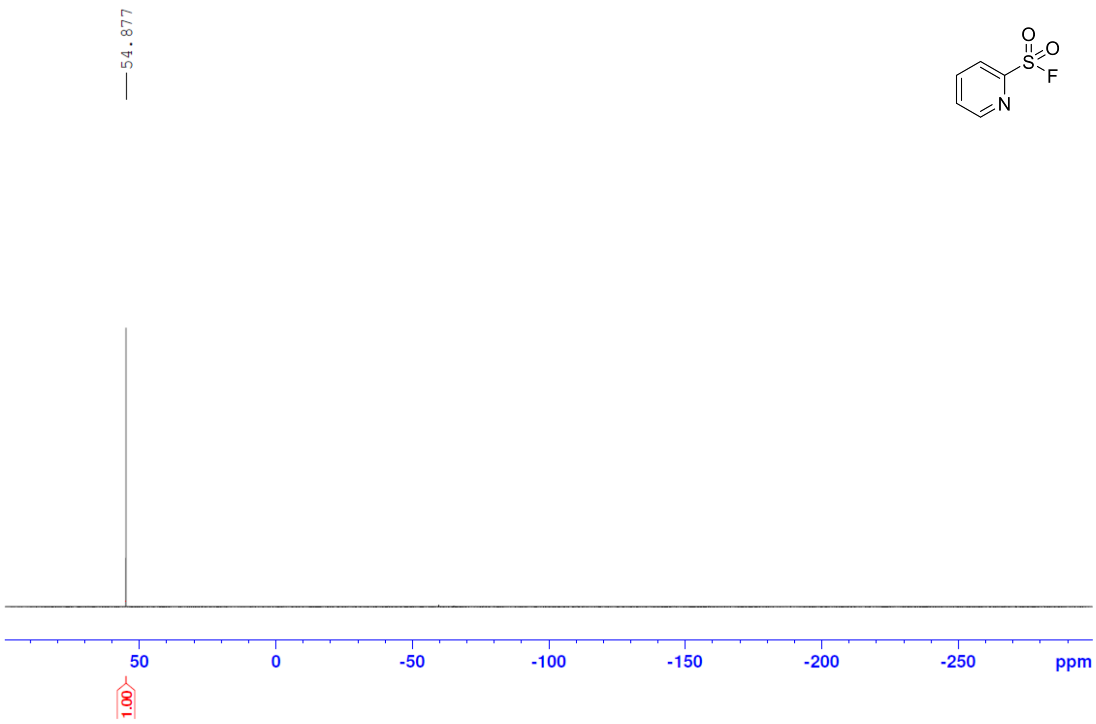

^{19}F NMR spectrum of pyridine-2-sulfonyl fluoride **3e** (377 MHz, CD_2Cl_2, 25 °C).

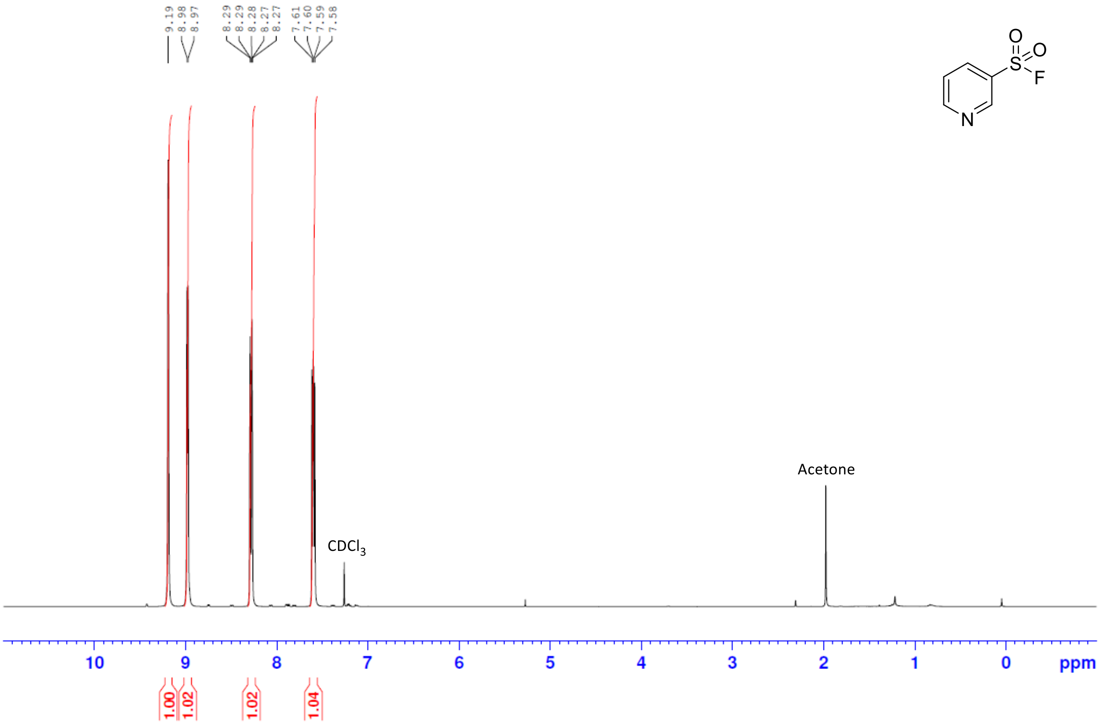

^{1}H NMR spectrum of pyridine-3-sulfonyl fluoride **3f** (400 MHz, $CDCl_3$, 25 °C).

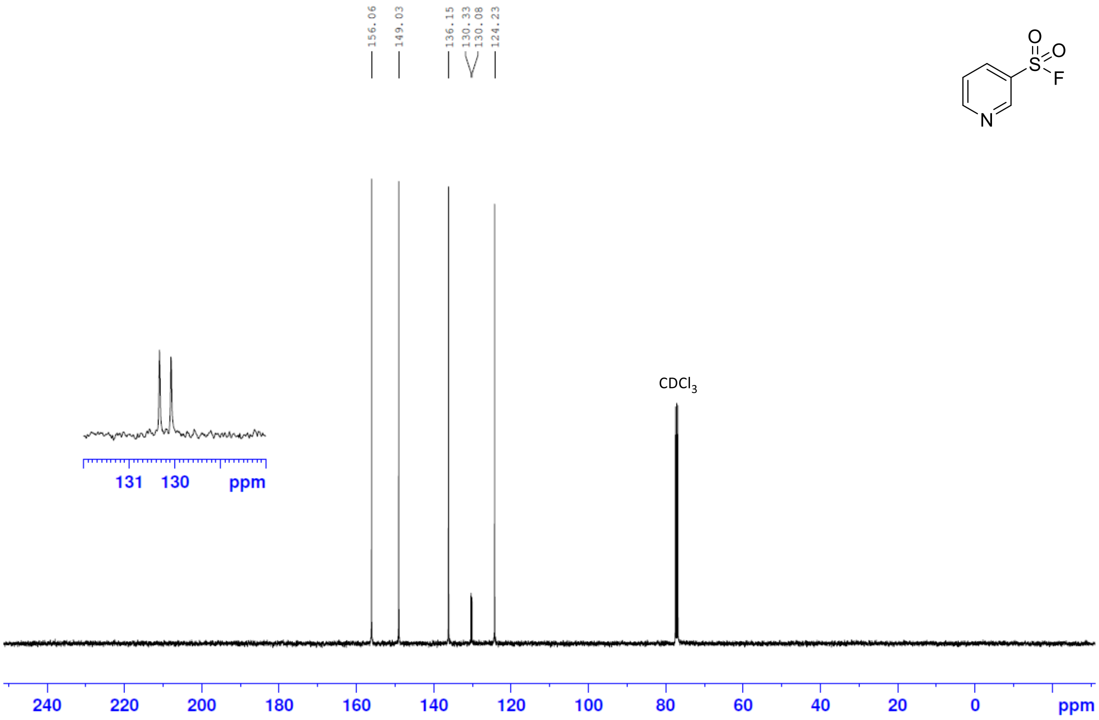

$^{13}C\{^1H\}$ NMR spectrum of pyridine-3-sulfonyl fluoride **3f** (101 MHz, $CDCl_3$, 25 °C).

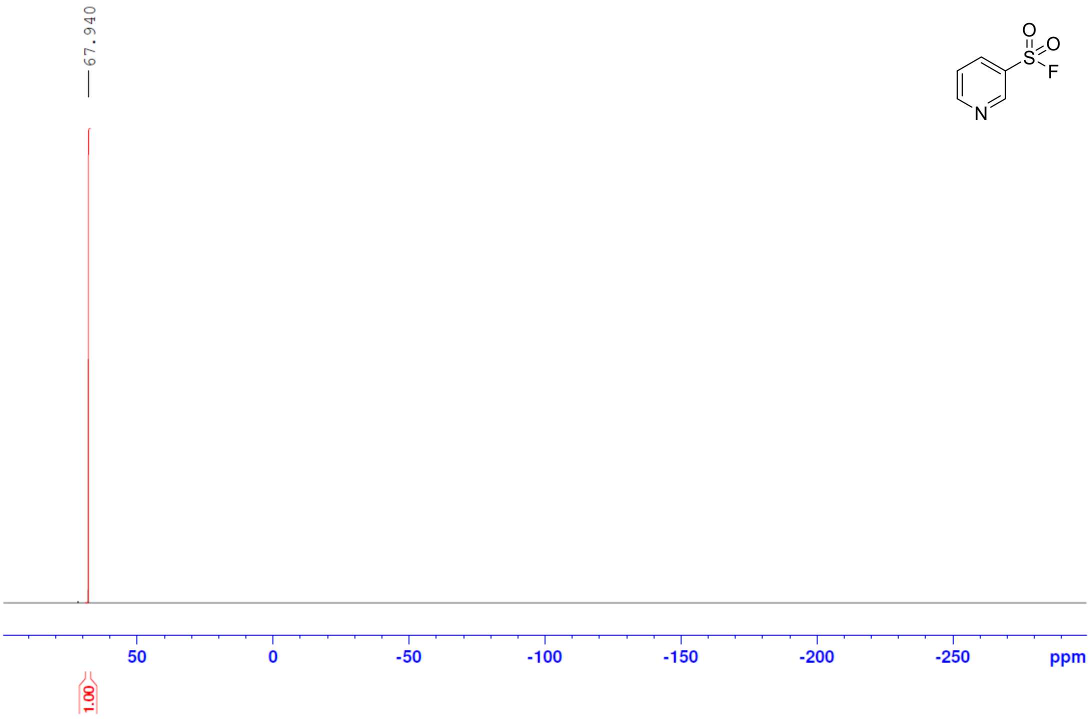

^{19}F NMR spectrum of pyridine-3-sulfonyl fluoride **3f** (377 MHz, $CDCl_3$, 25 °C).

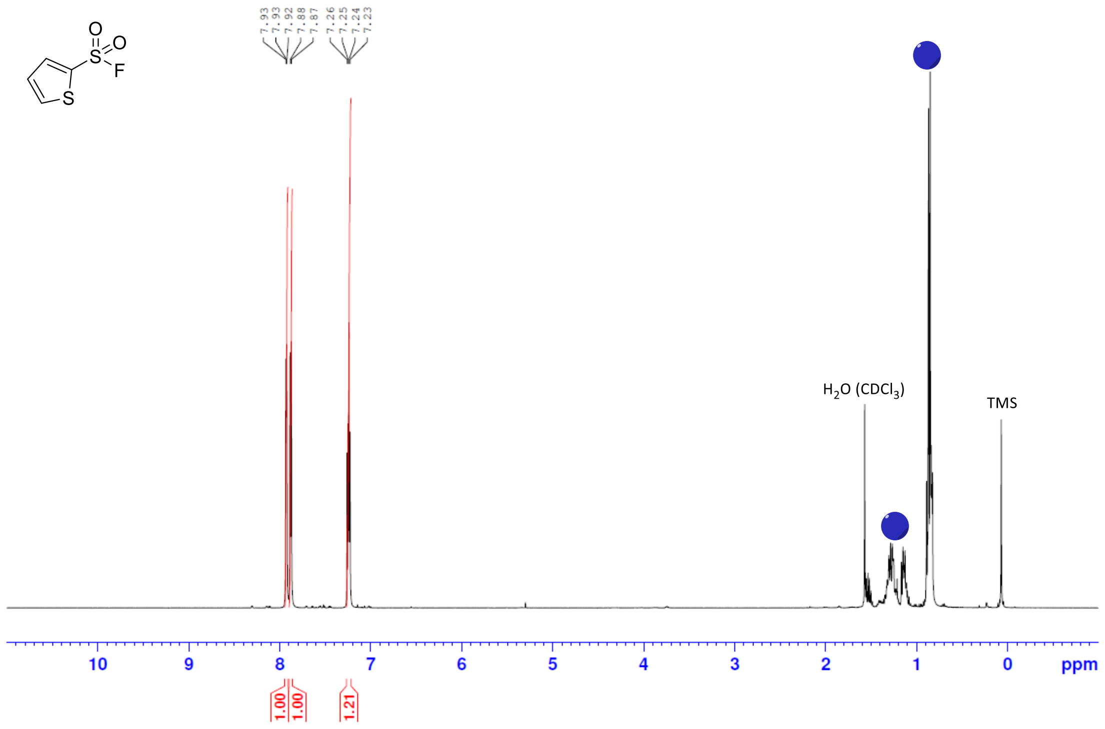

^{1}H NMR spectrum of thiophene-2-sulfonyl fluoride **3g** (400 MHz, $CDCl_3$, 25 °C). The blue filled circles represent the signals of petroleum ether which could not be sufficiently removed because of the volatility of the product molecule.

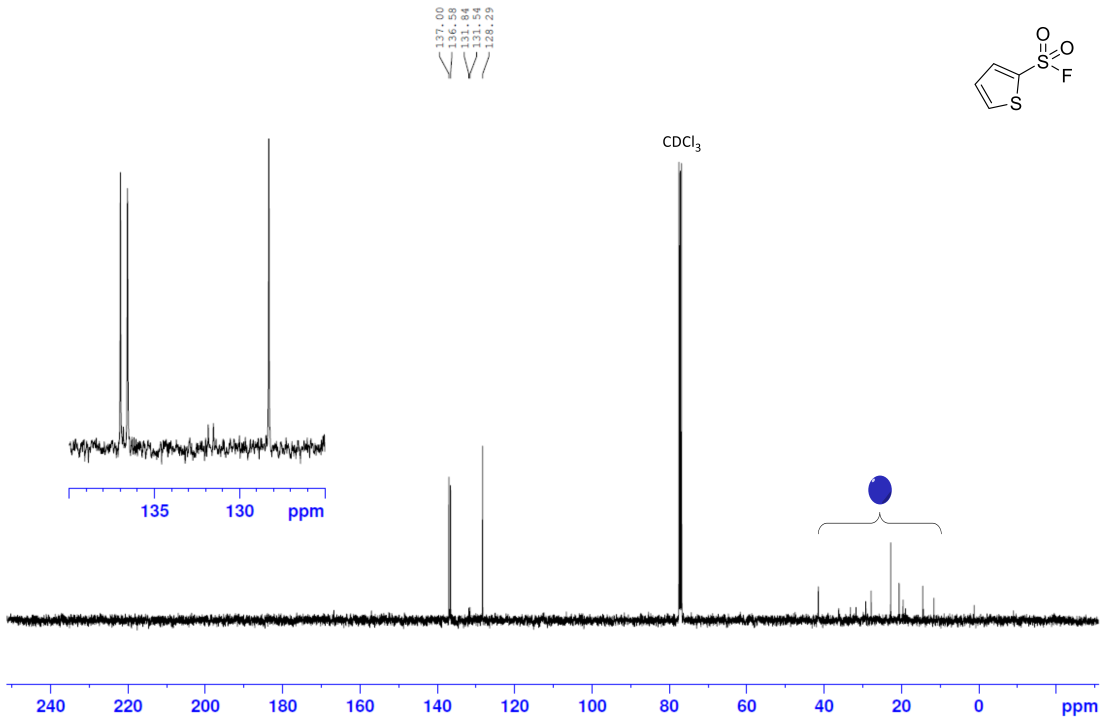

^{13}C{^{1}H} NMR spectrum of thiophene-2-sulfonyl fluoride **3g** (101 MHz, $CDCl_3$, 25 °C). The range under the blue filled circle represents the signals of petroleum ether which could not be sufficiently removed because of the volatility of the product molecule.

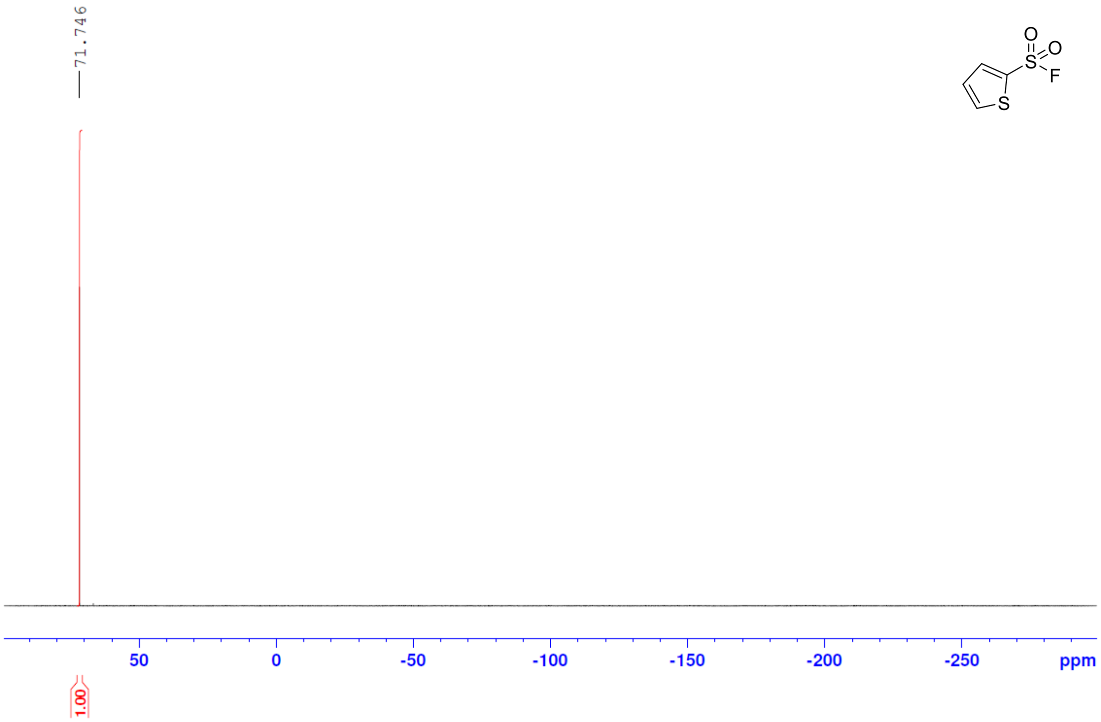

^{19}F NMR spectrum of thiophene-2-sulfonyl fluoride **3g** (377 MHz, $CDCl_3$, 25 °C).

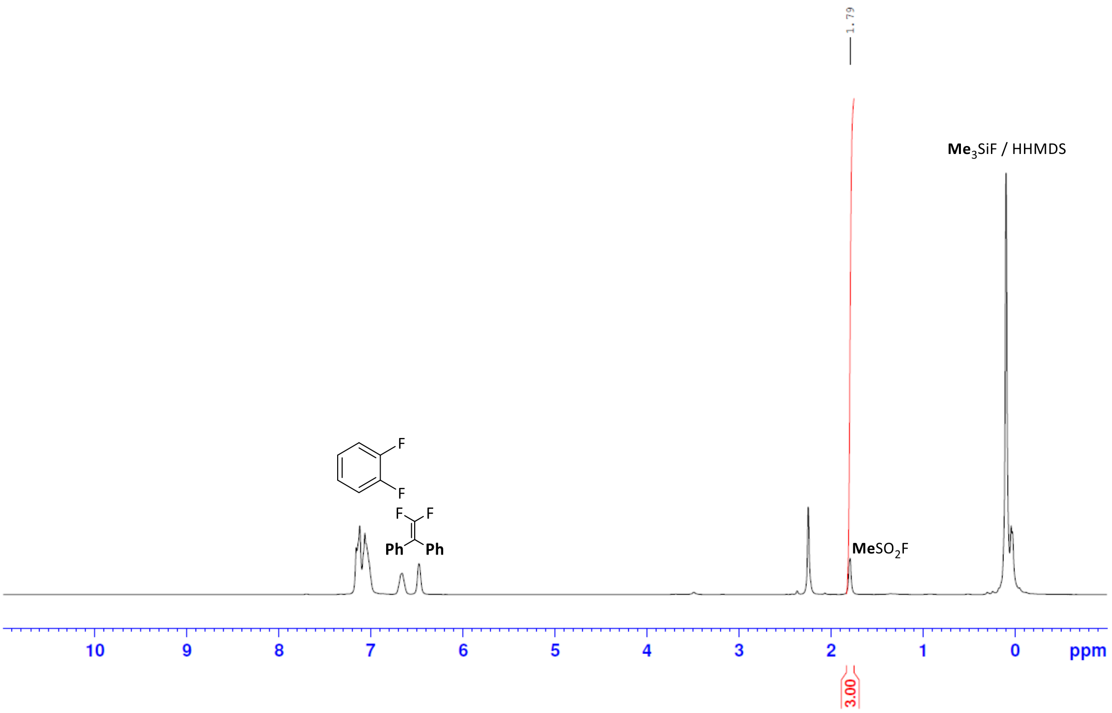

In situ ^{1}H NMR spectrum of the synthesis mixture containing methanesulfonyl fluoride **3h** (400 MHz, C_6D_6, 25 °C).

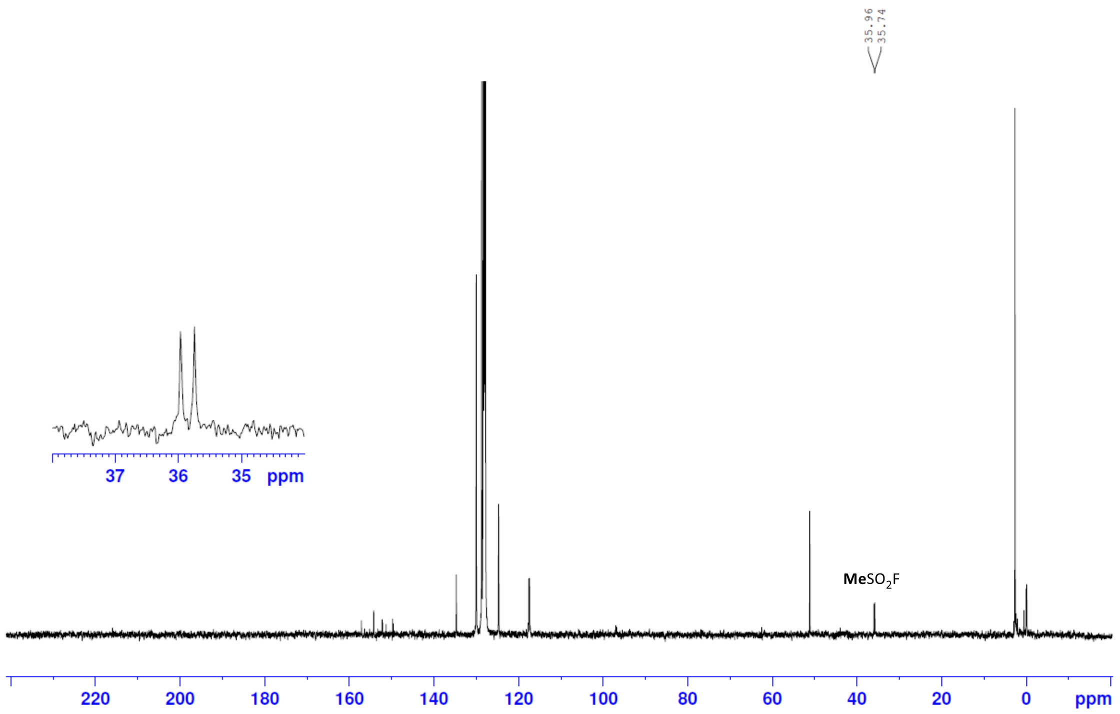

In situ $^{13}C\{^1H\}$ NMR spectrum of the synthesis mixture containing methanesulfonyl fluoride **3h** (101 MHz, C_6D_6, 25 °C).

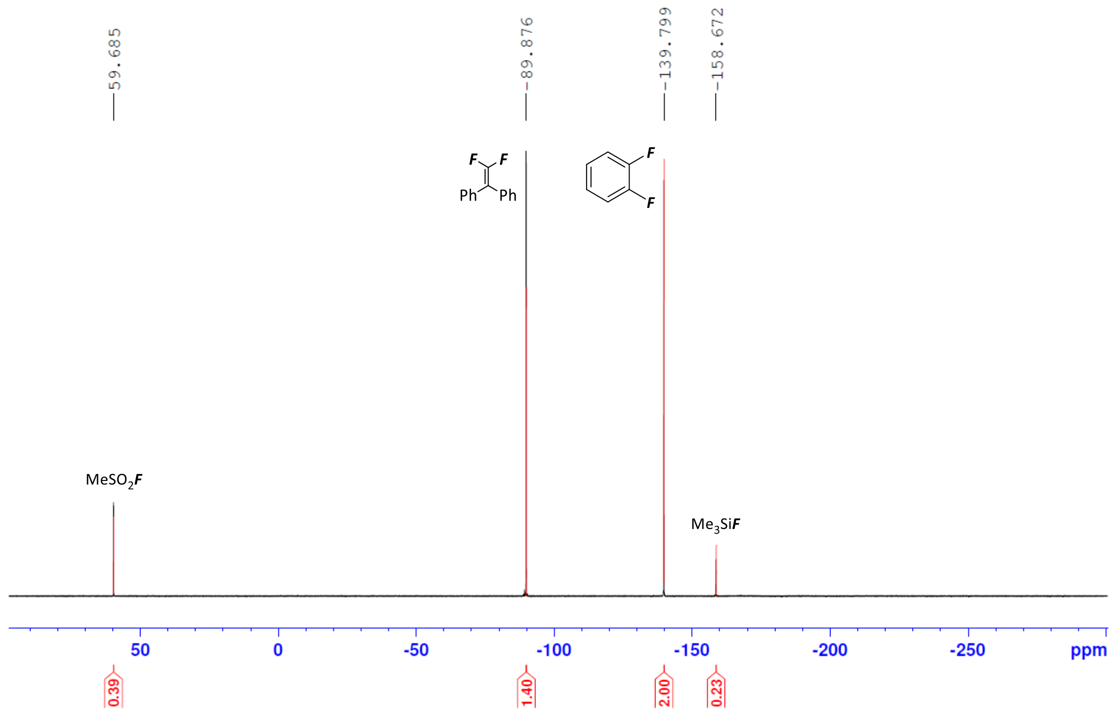

In situ quantitative ^{19}F NMR spectrum of the synthesis mixture containing methanesulfonyl fluoride **3h** (377 MHz, C_6D_6, 25 °C).

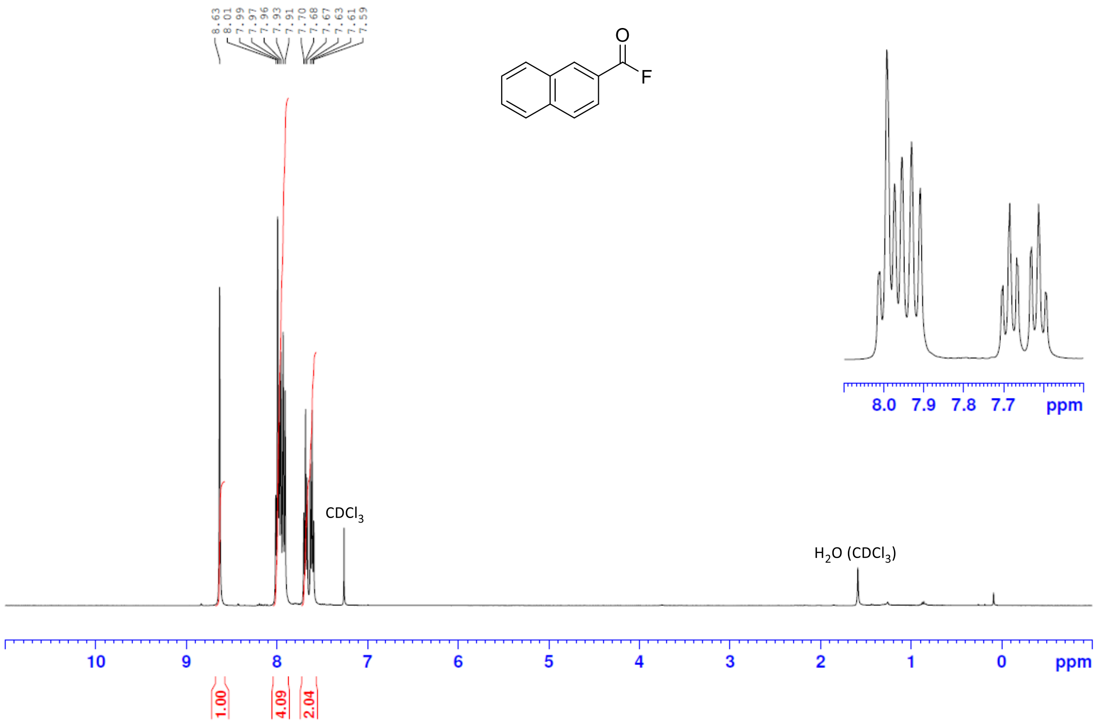

^{1}H NMR spectrum of 2-naphthoyl fluoride **3i** (400 MHz, $CDCl_3$, 25 °C).

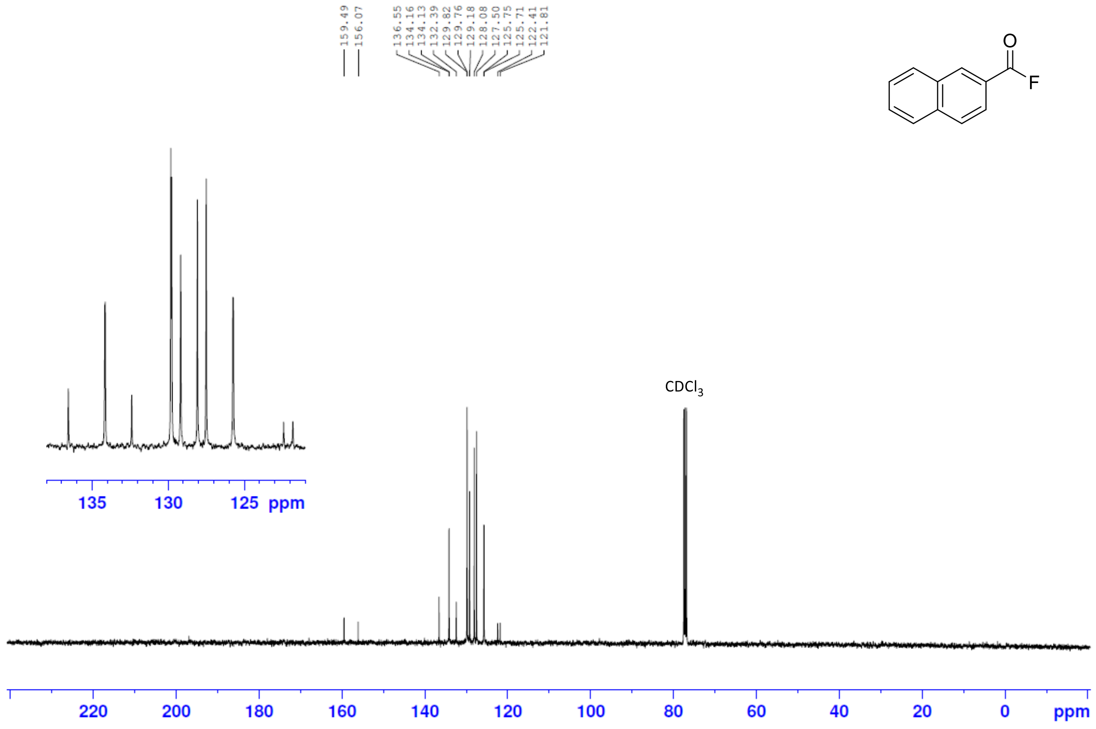

$^{13}C\{^{1}H\}$ NMR spectrum of 2-naphthoyl fluoride **3i** (101 MHz, $CDCl_3$, 25 °C).

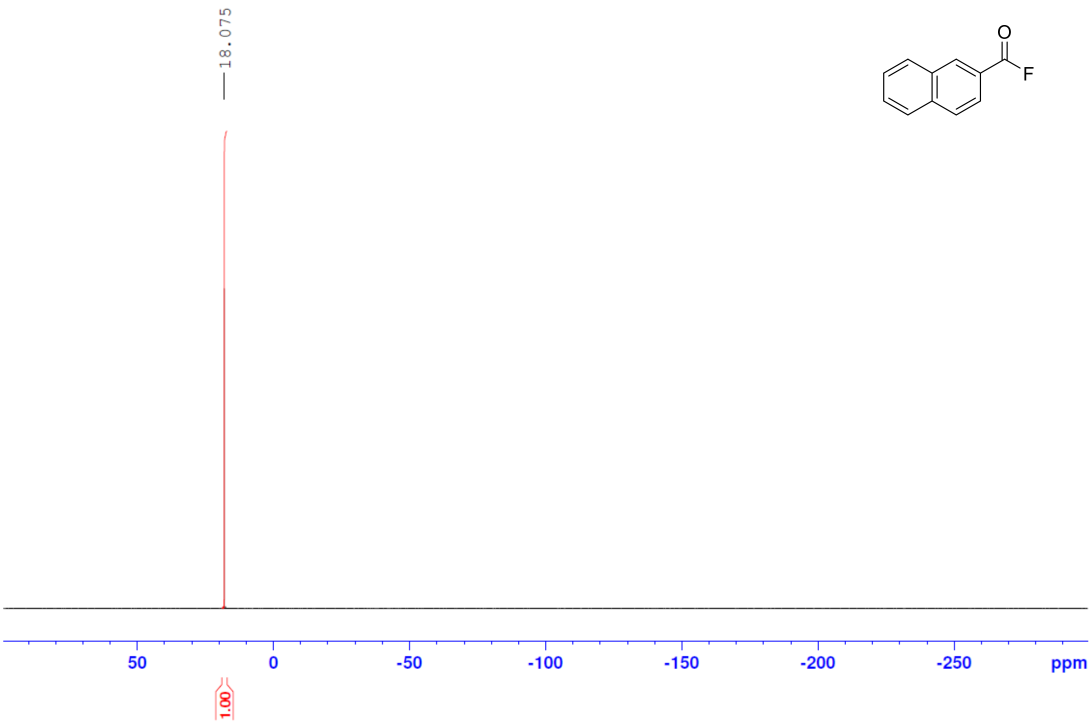

^{19}F NMR spectrum of 2-naphthoyl fluoride **3i** (377 MHz, $CDCl_3$, 25 °C).

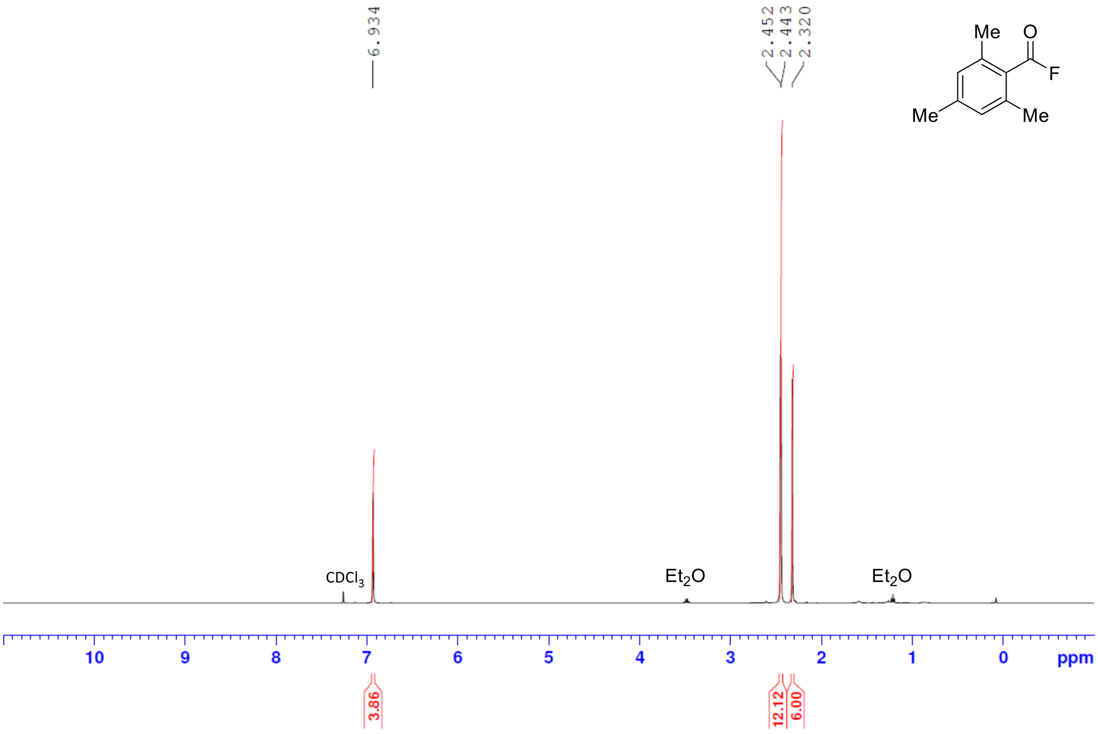

^{1}H NMR spectrum of 2,4,6-trimethylbenzoyl fluoride **3j** (400 MHz, $CDCl_3$, 25 °C).

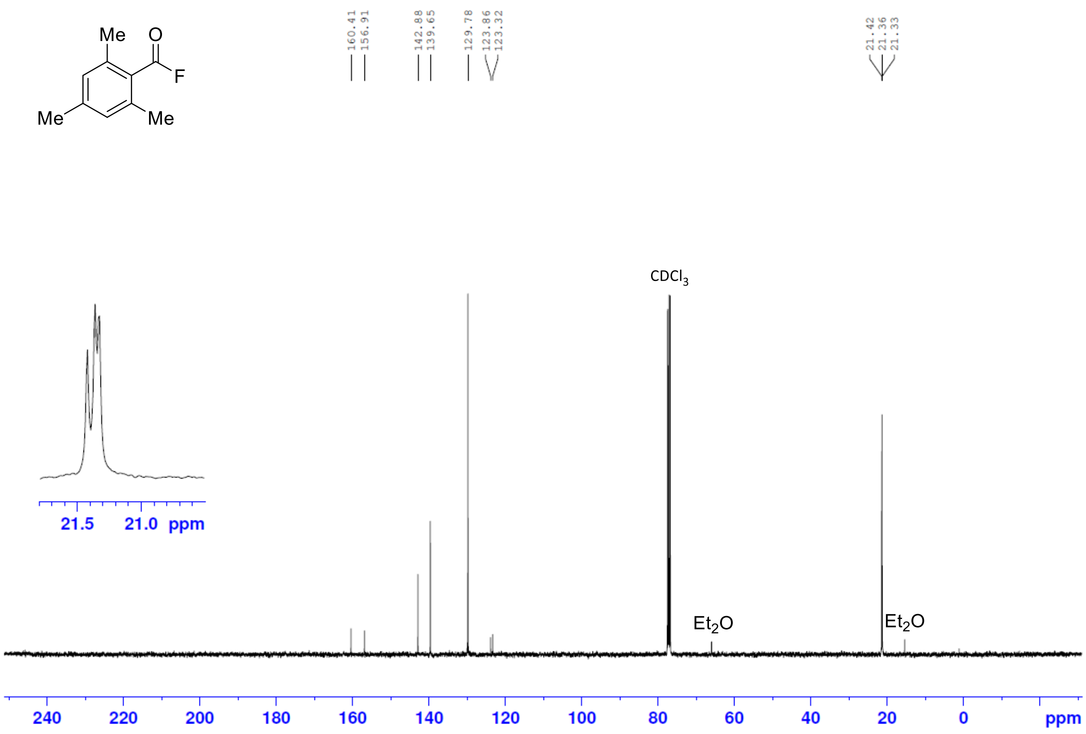

$^{13}C\{^{1}H\}$ NMR spectrum of 2,4,6-trimethylbenzoyl fluoride **3j** (101 MHz, $CDCl_3$, 25 °C).

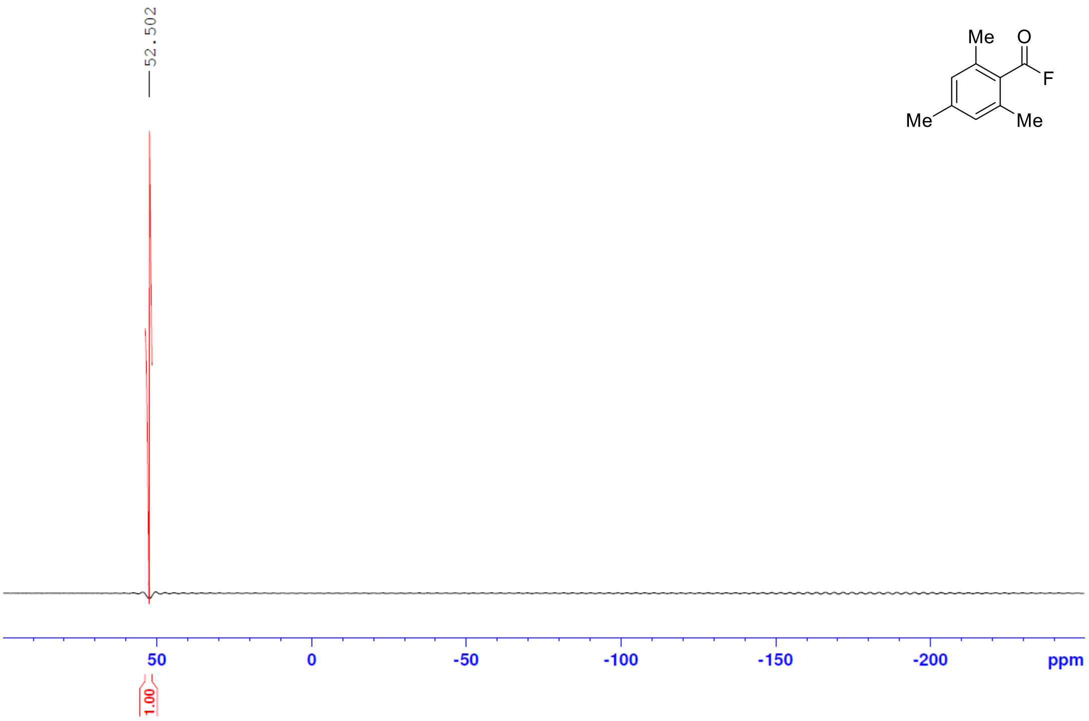

^{19}F NMR spectrum of 2,4,6-trimethylbenzoyl fluoride **3j** (377 MHz, $CDCl_3$, 25 °C).

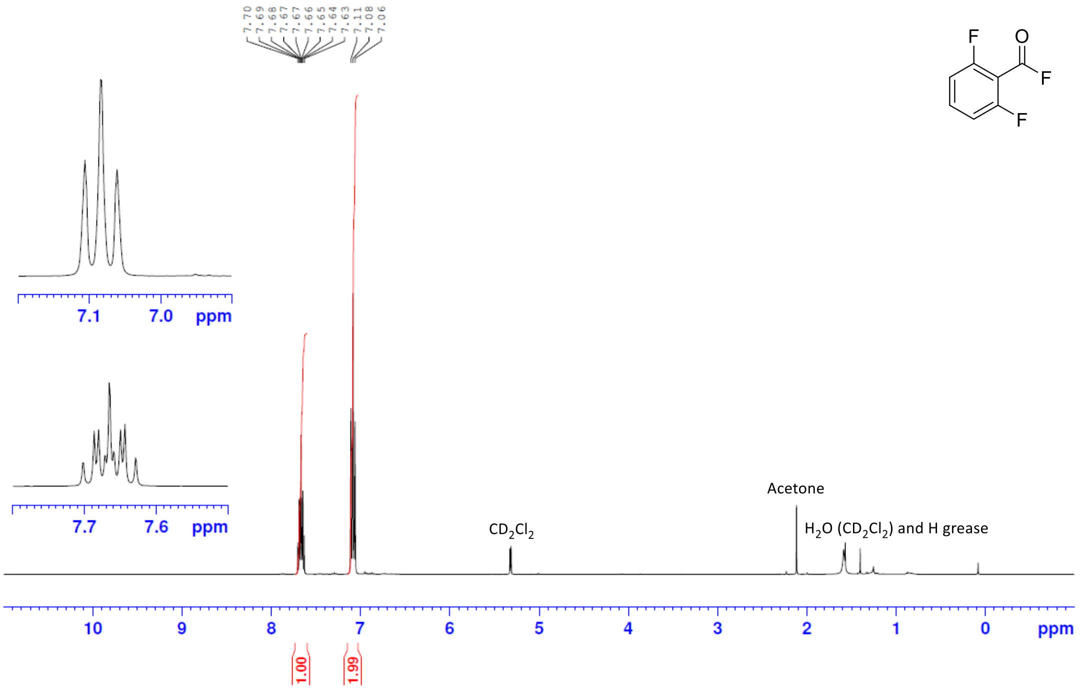

^{1}H NMR spectrum of 2,6-difluorobenzoyl fluoride **3k** (400 MHz, CD_2Cl_2, 25 °C).

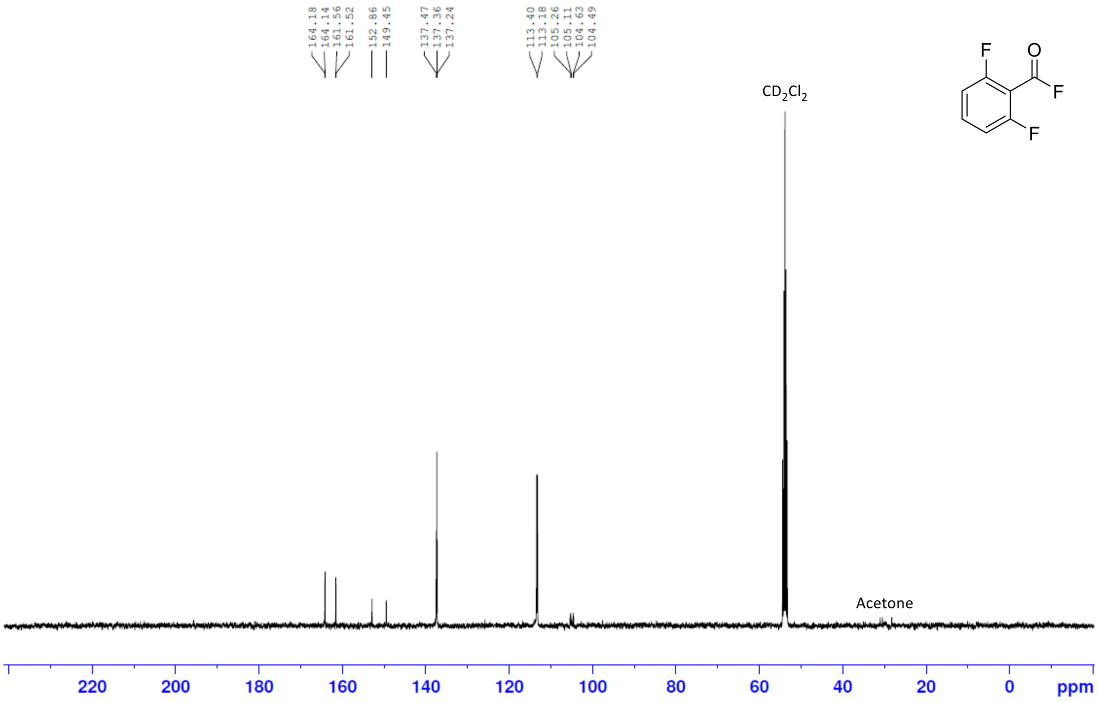

^{13}C{^{1}H} NMR spectrum of 2,6-difluorobenzoyl fluoride **3k** (101 MHz, CD_2Cl_2, 25 °C).

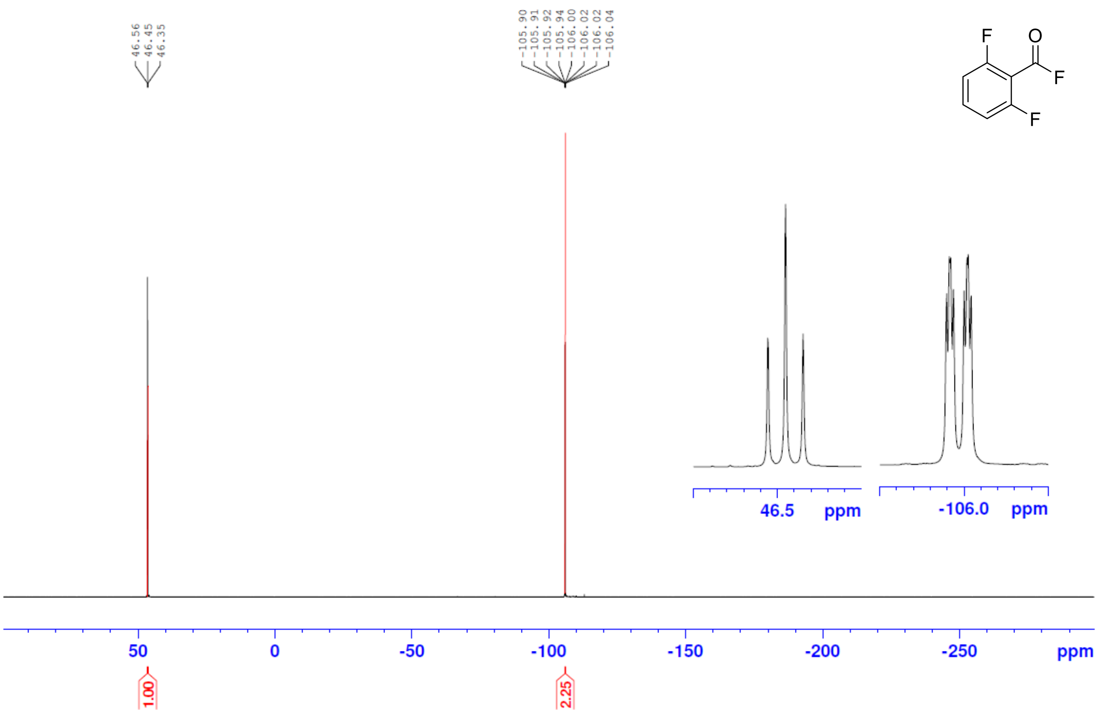

Quantitative ^{19}F NMR spectrum of 2,6-difluorobenzoyl fluoride **3k**
(377 MHz, CD_2Cl_2, 25 °C).

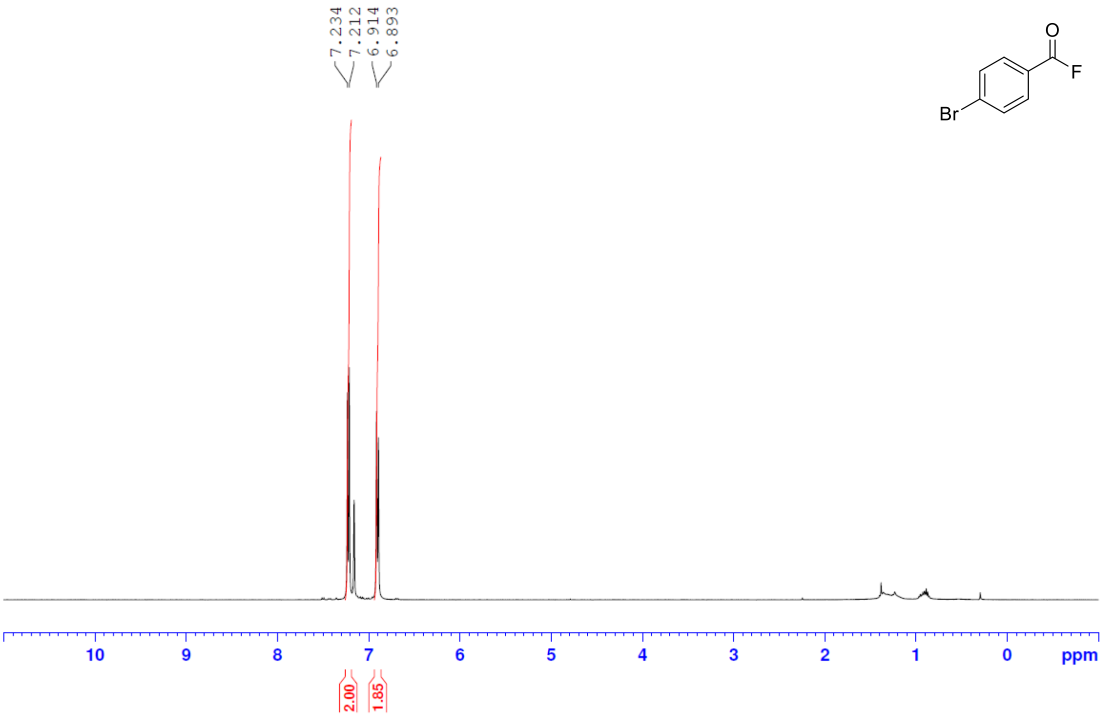

^{1}H NMR spectrum of 4-bromobenzoyl fluoride **3l** (400 MHz, C_6D_6, 25 °C).

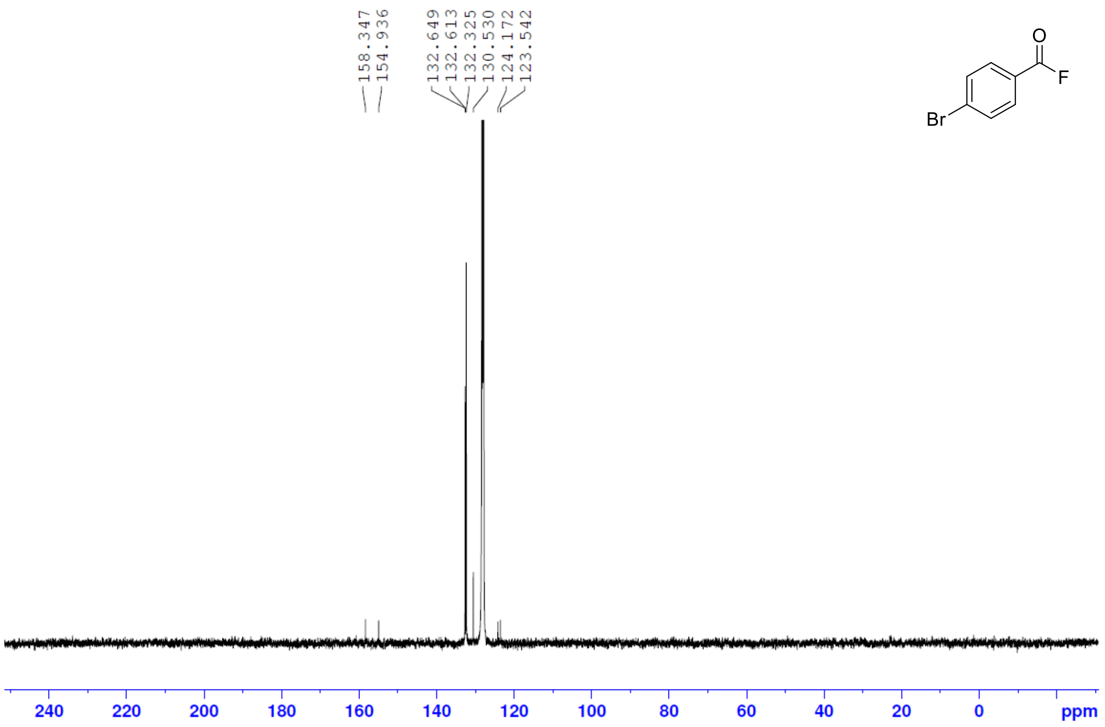

$^{13}C\{^{1}H\}$ NMR spectrum of 4-bromobenzoyl fluoride **3l** (101 MHz, C_6D_6, 25 °C).

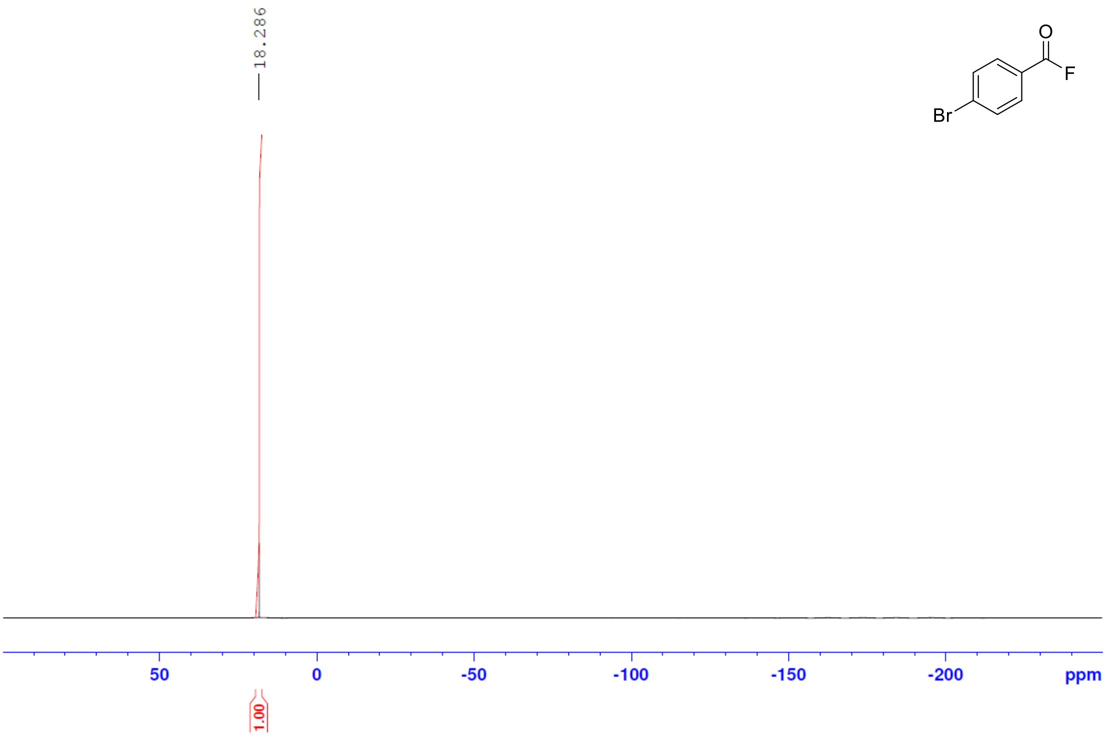

^{19}F NMR spectrum of 4-bromobenzoyl fluoride **3l** (377 MHz, C_6D_6, 25 °C).

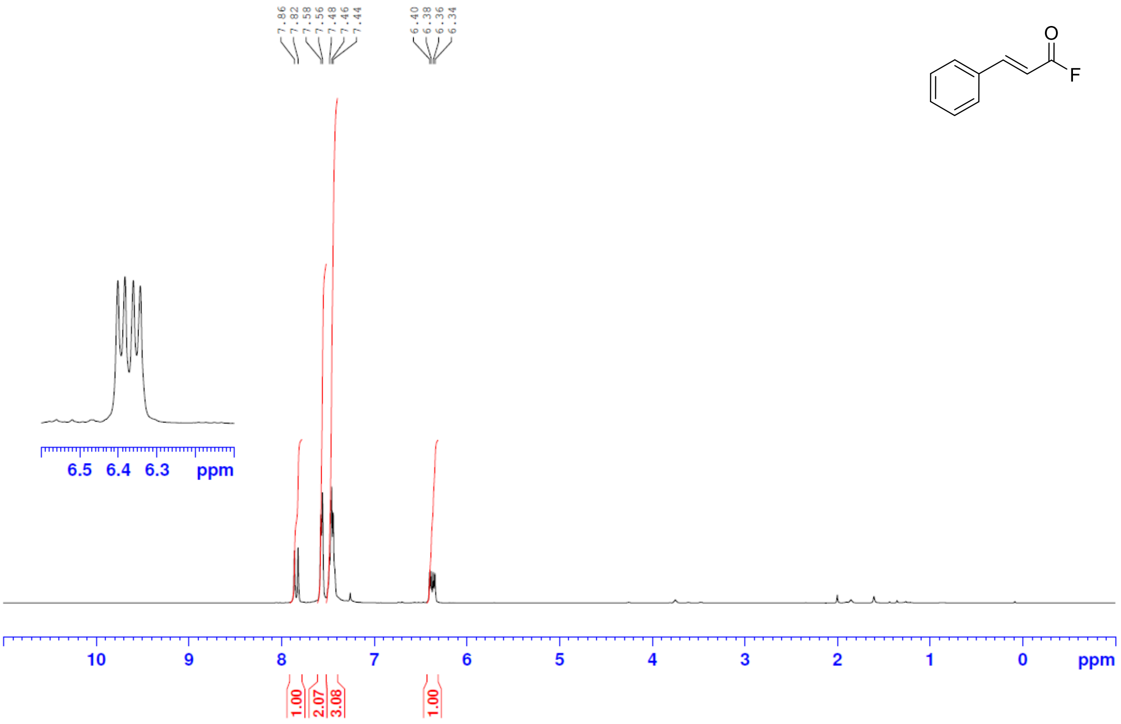

^{1}H NMR spectrum of cinnamoyl fluoride **3m** (400 MHz, $CDCl_3$, 25 °C).

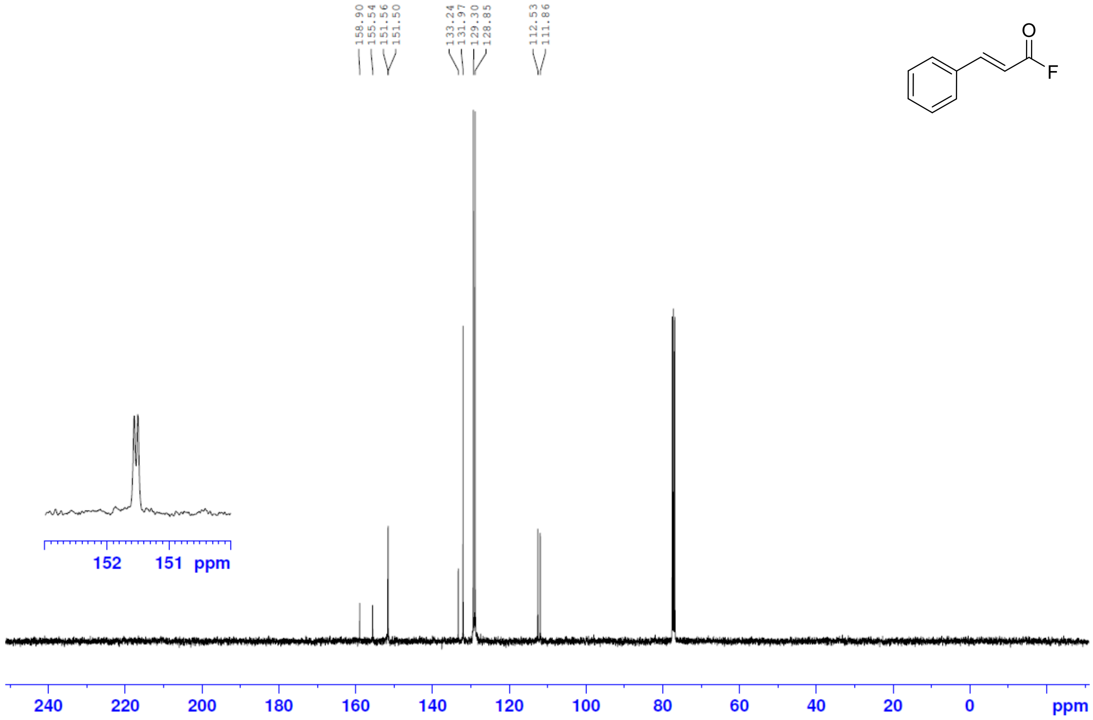

^{13}C{^{1}H} NMR spectrum of cinnamoyl fluoride **3m** (101 MHz, $CDCl_3$, 25 °C).

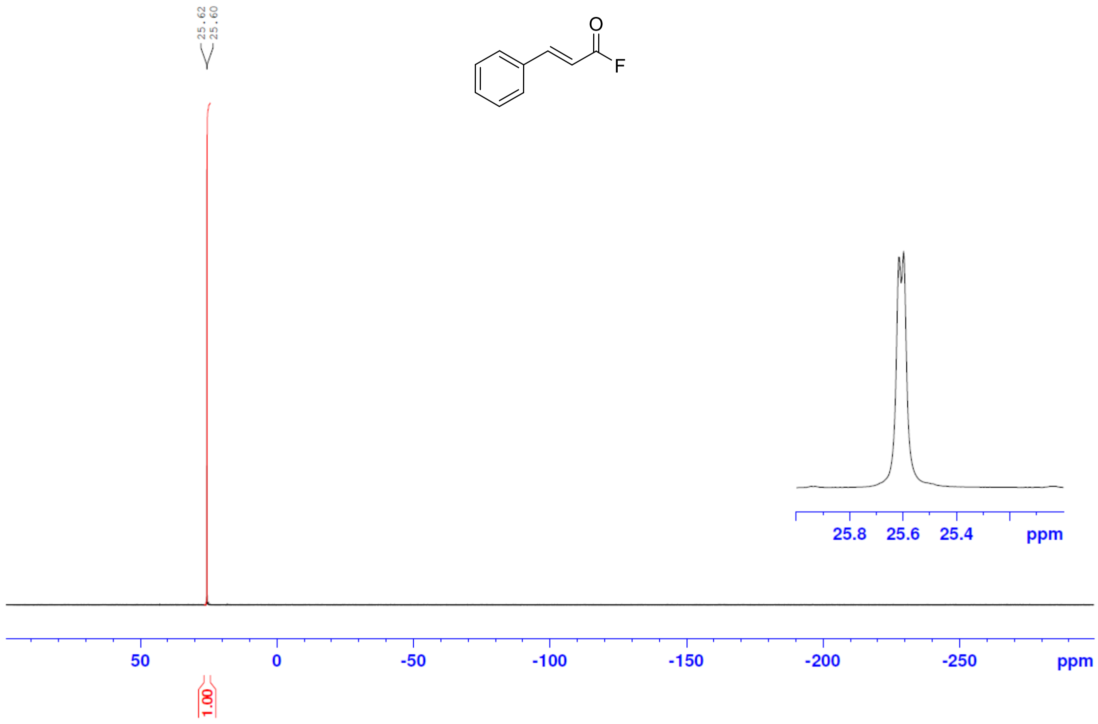

^{19}F NMR spectrum of cinnamoyl fluoride **3m** (377 MHz, $CDCl_3$, 25 °C).

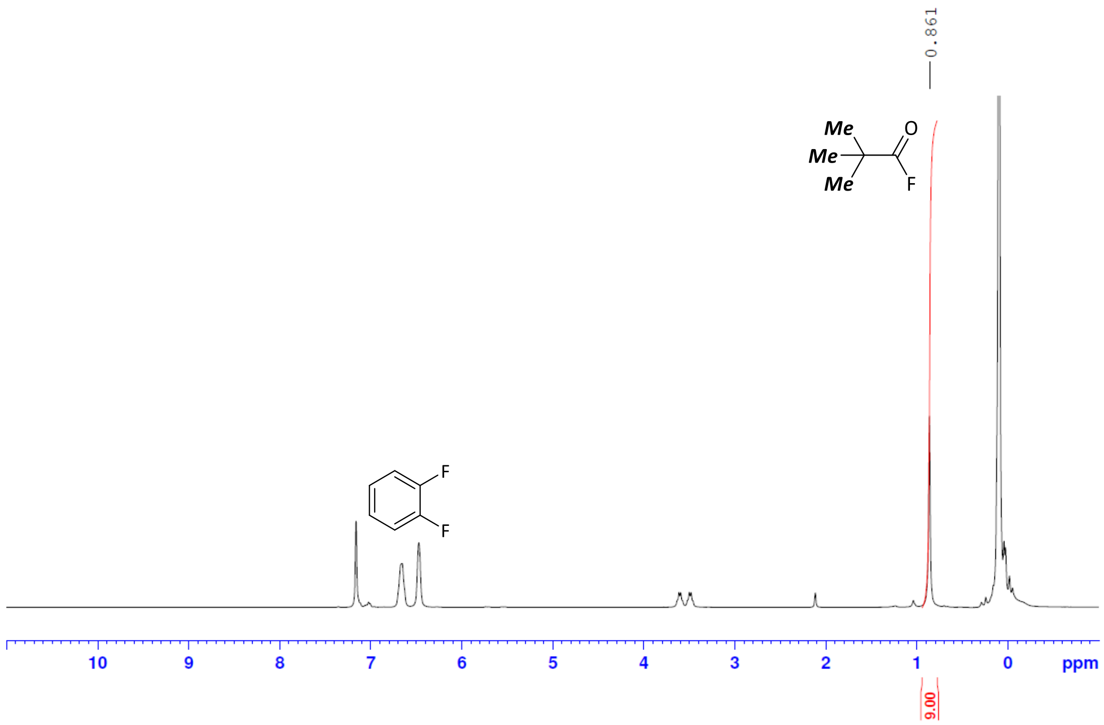

In situ ^{1}H NMR spectrum of the synthesis mixture containing pivaloyl fluoride **3n** (400 MHz, C_6D_6, 25 °C).

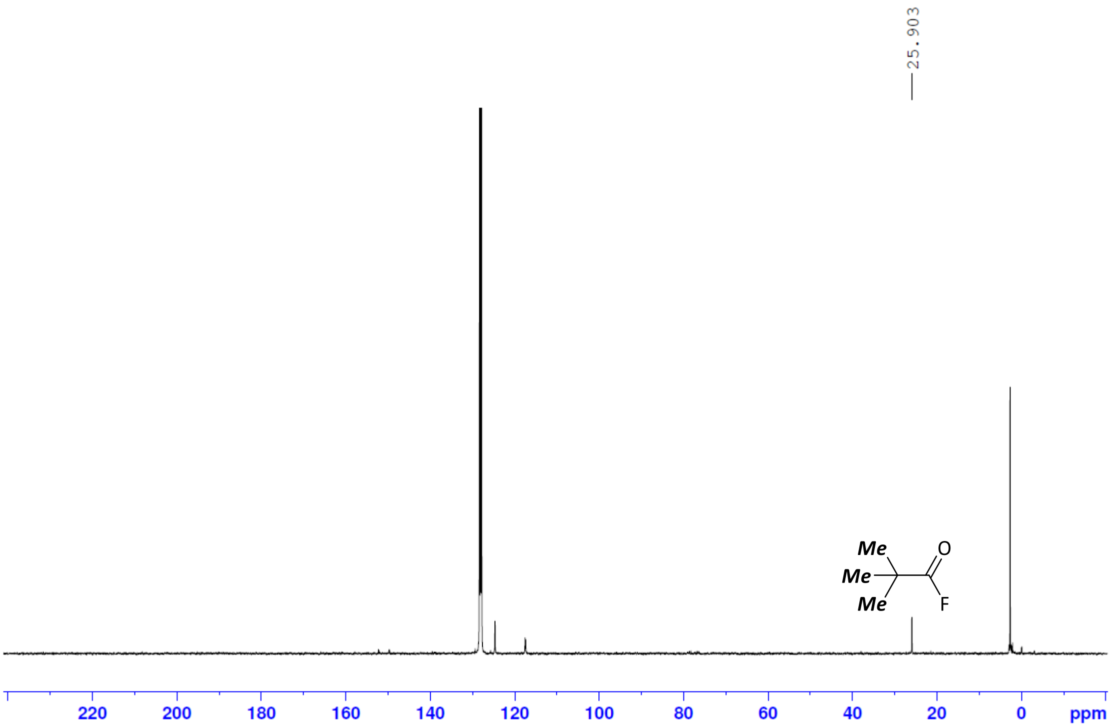

In situ $^{13}C\{^{1}H\}$ NMR spectrum of the synthesis mixture containing pivaloyl fluoride **3n** (101 MHz, C_6D_6, 25 °C).

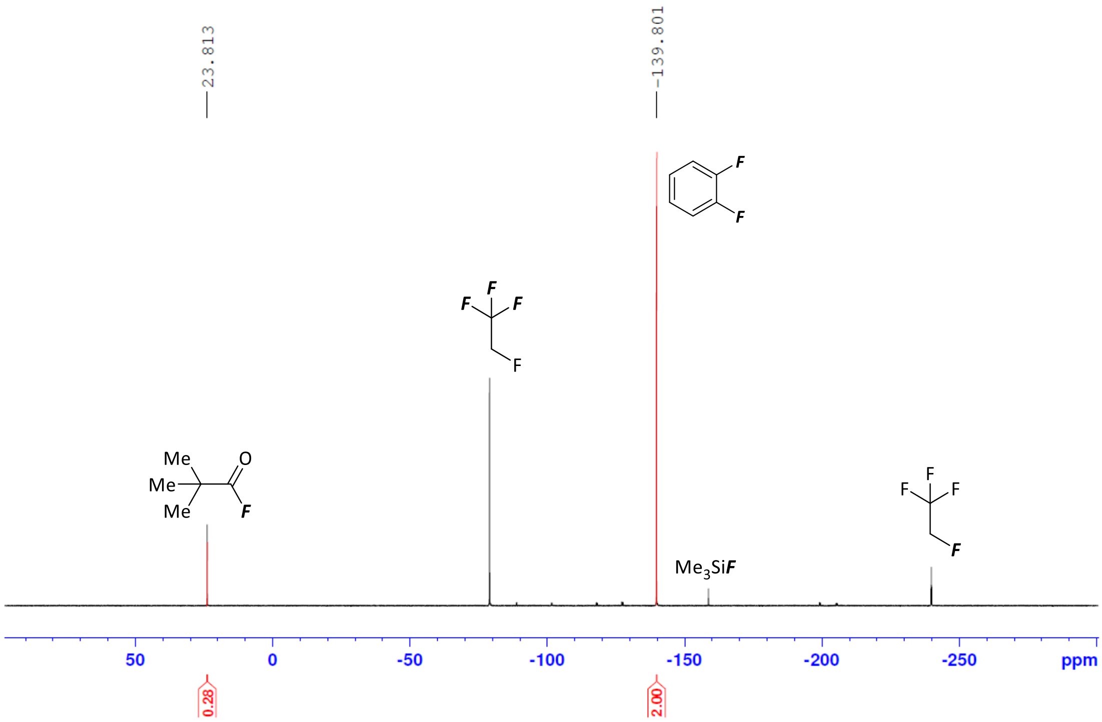

In situ quantitative ^{19}F NMR spectrum of the synthesis mixture containing pivaloyl fluoride **3n** (377 MHz, C_6D_6, 25 °C).

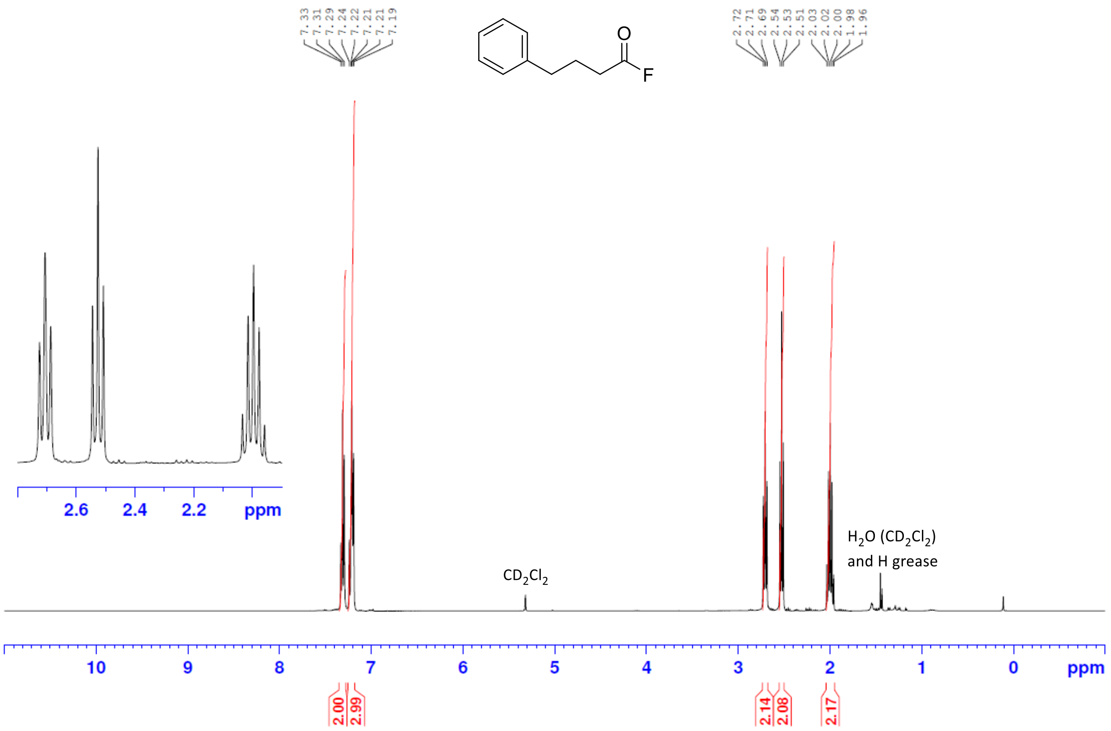

^{1}H NMR spectrum of 4-phenylbutanoyl fluoride **3o** (400 MHz, CD_2Cl_2, 25 °C).

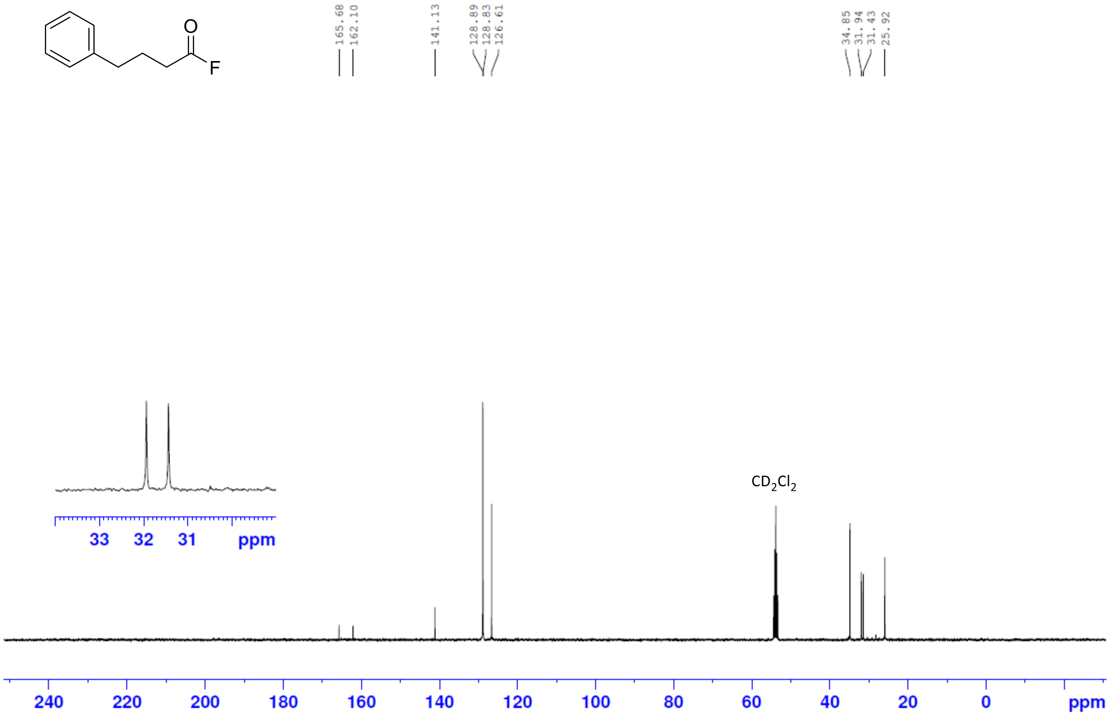

^{13}C{^{1}H} NMR spectrum of 4-phenylbutanoyl fluoride **3o** (101 MHz, CD_2Cl_2, 25 °C).

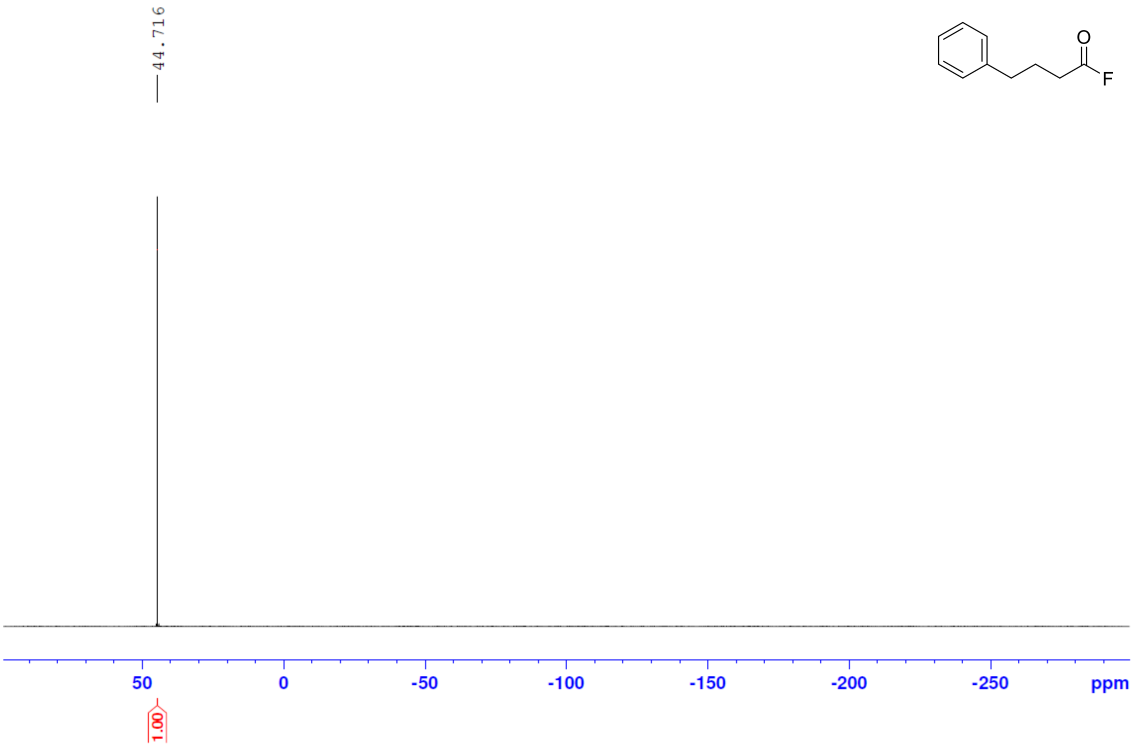

^{19}F NMR spectrum of 4-phenylbutanoyl fluoride **3o** (377 MHz, CD_2Cl_2, 25 °C).

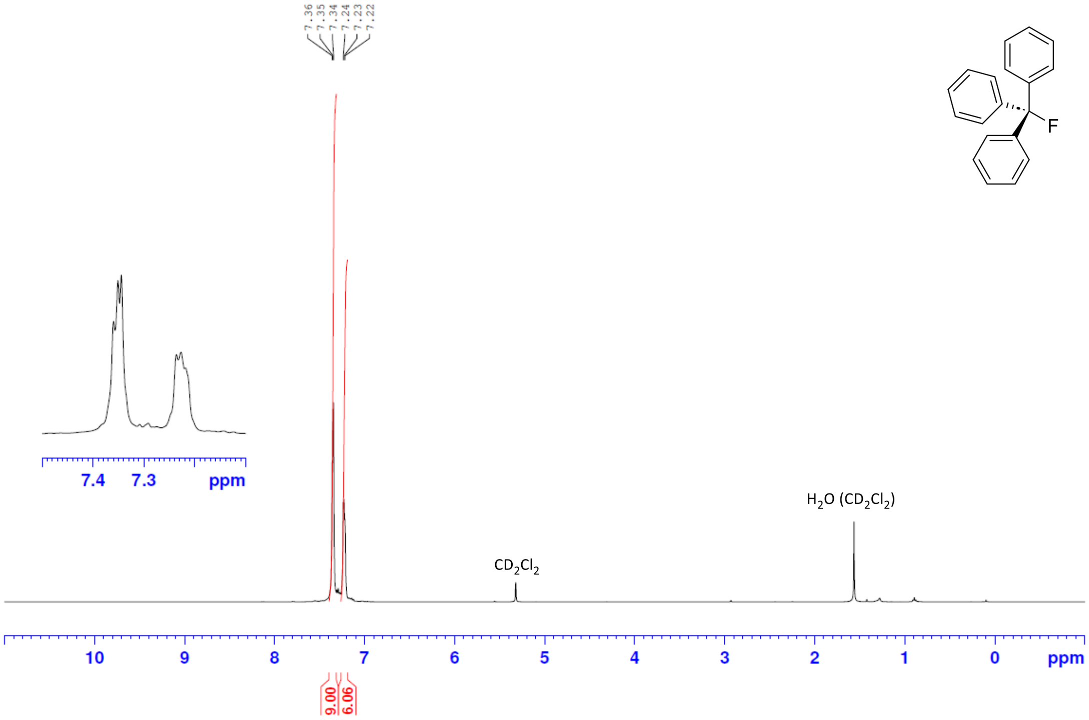

^{1}H NMR spectrum of trityl fluoride **3p** (400 MHz, CD_2Cl_2, 25 °C).

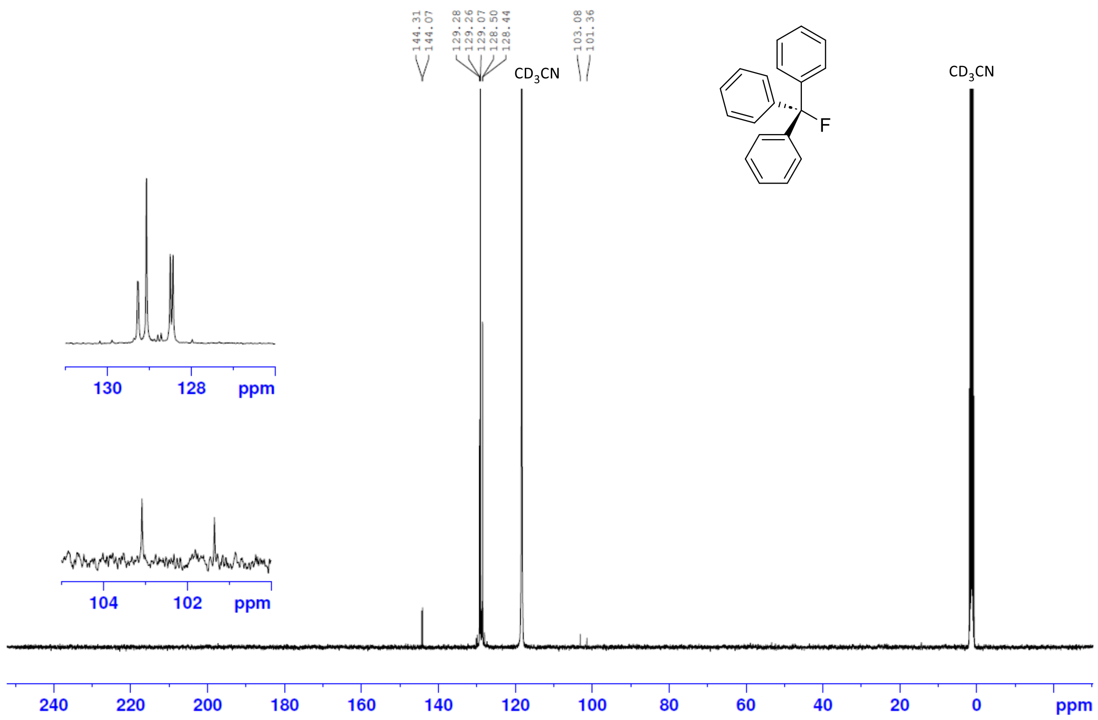

$^{13}C\{^{1}H\}$ NMR spectrum of trityl fluoride **3p** (101 MHz, CD_3CN, 25 °C).

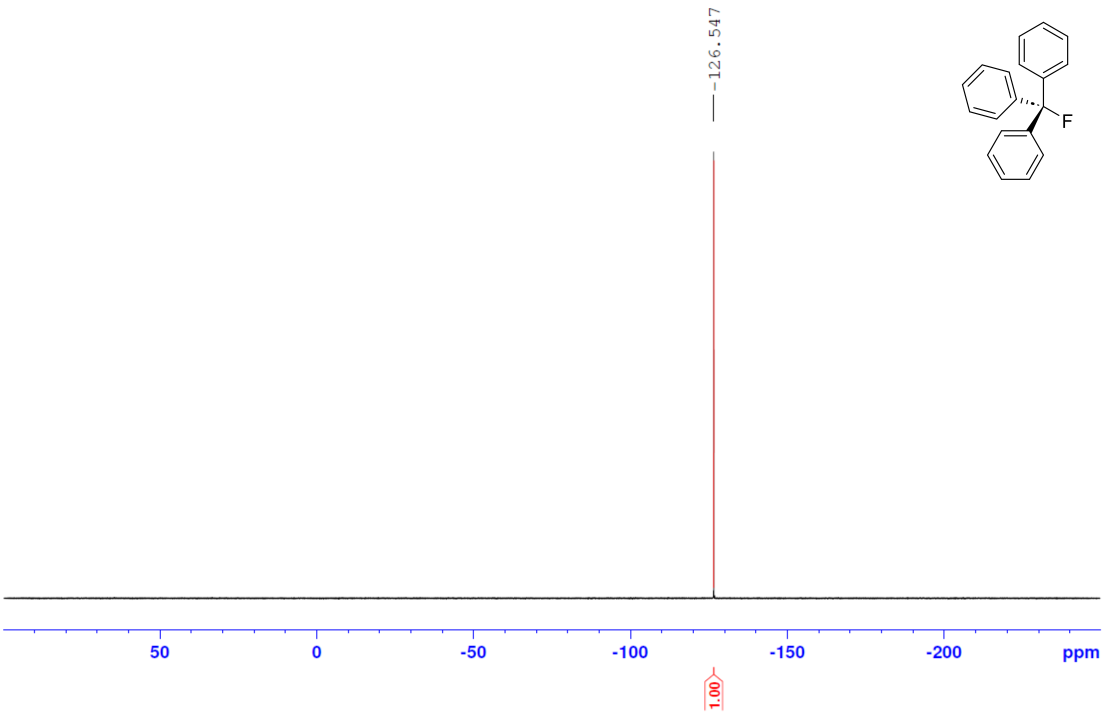

^{19}F NMR spectrum of trityl fluoride **3p** (377 MHz, CD_2Cl_2, 25 °C).

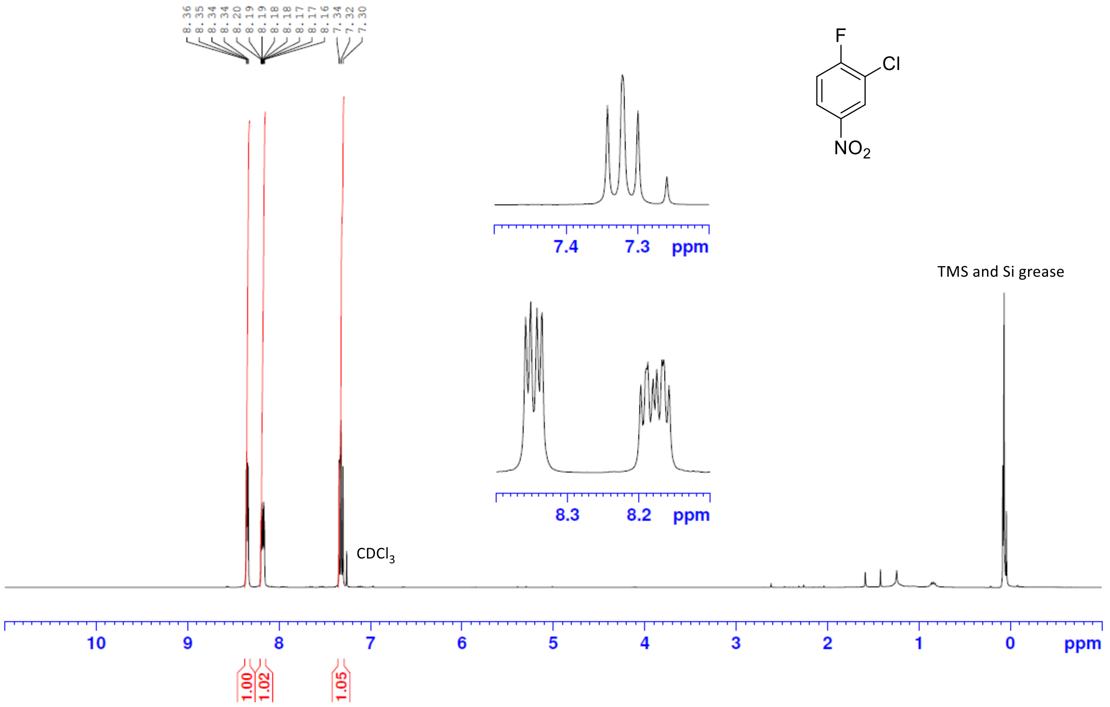

^{1}H NMR spectrum of 3-chloro-4-fluoronitrobenzene **3q** (400 MHz, $CDCl_3$, 25 °C).

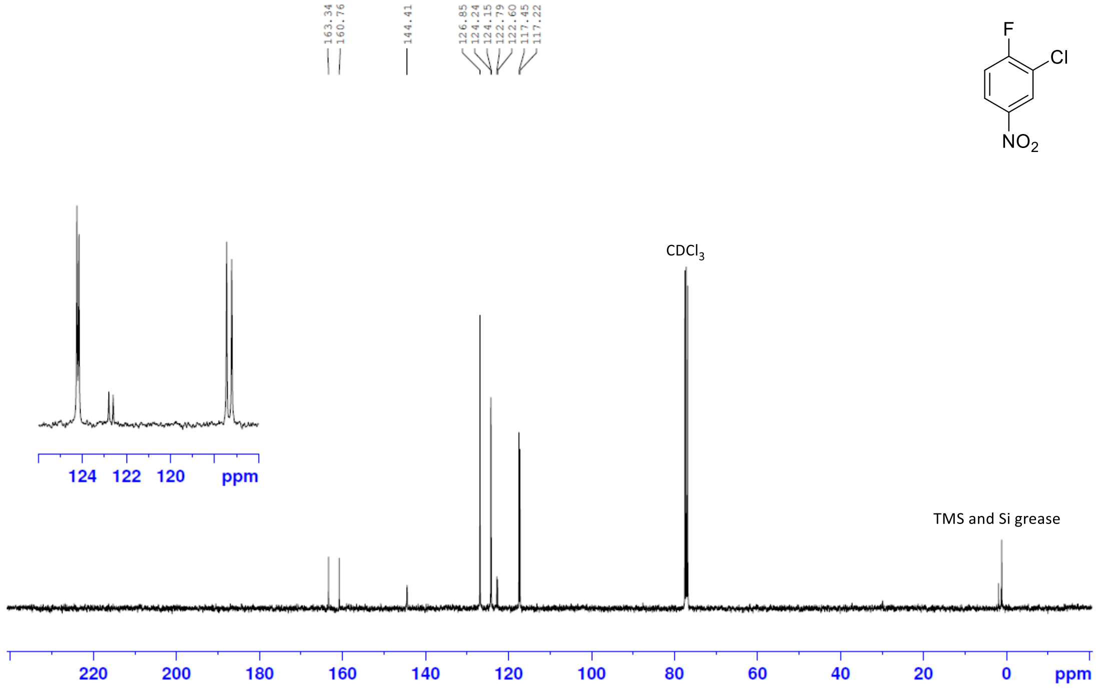

^{13}C{^{1}H} NMR spectrum of 3-chloro-4-fluoronitrobenzene **3q** (101 MHz, $CDCl_3$, 25 °C).

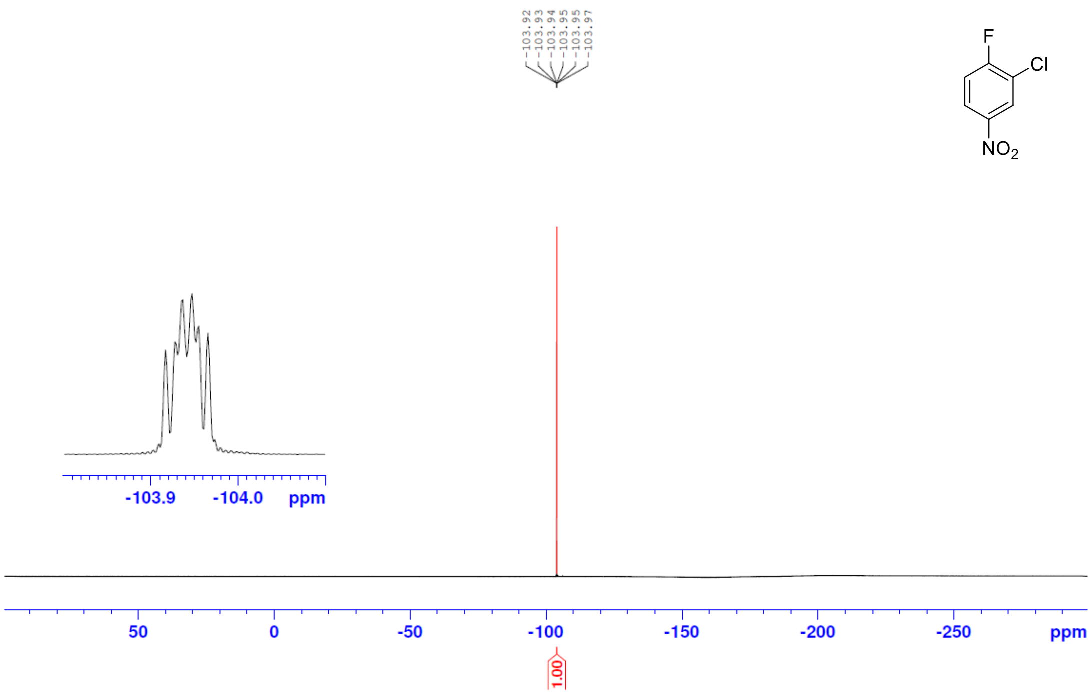

^{19}F NMR spectrum of 3-chloro-4-fluoronitrobenzene **3q** (377 MHz, $CDCl_3$, 25 °C).

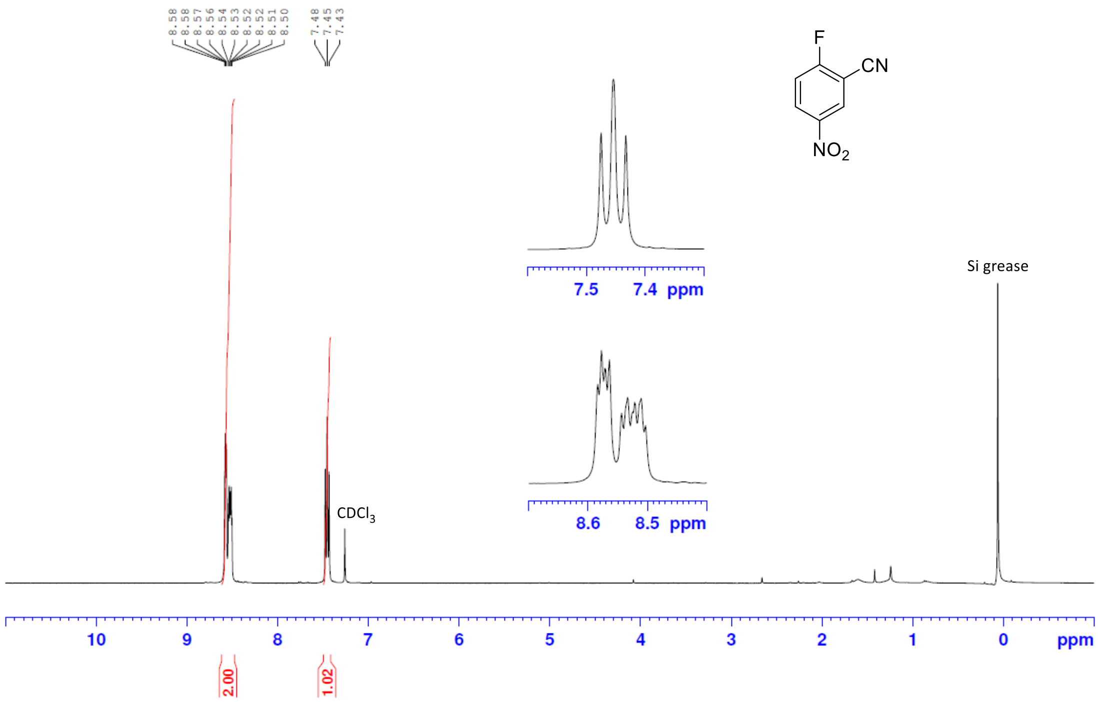

^{1}H NMR spectrum of 2-fluoro-5-nitrobenzonitrile **3r** (400 MHz, $CDCl_3$, 25 °C).

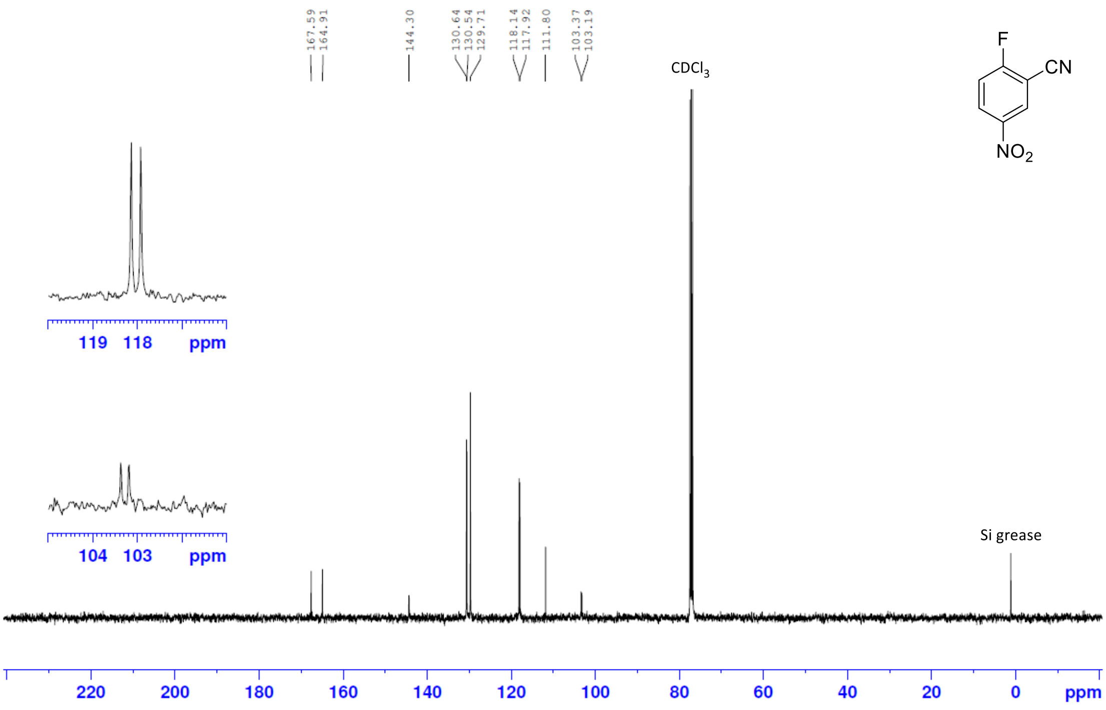

$^{13}C\{^{1}H\}$ NMR spectrum of 2-fluoro-5-nitrobenzonitrile **3r** (101 MHz, $CDCl_3$, 25 °C).

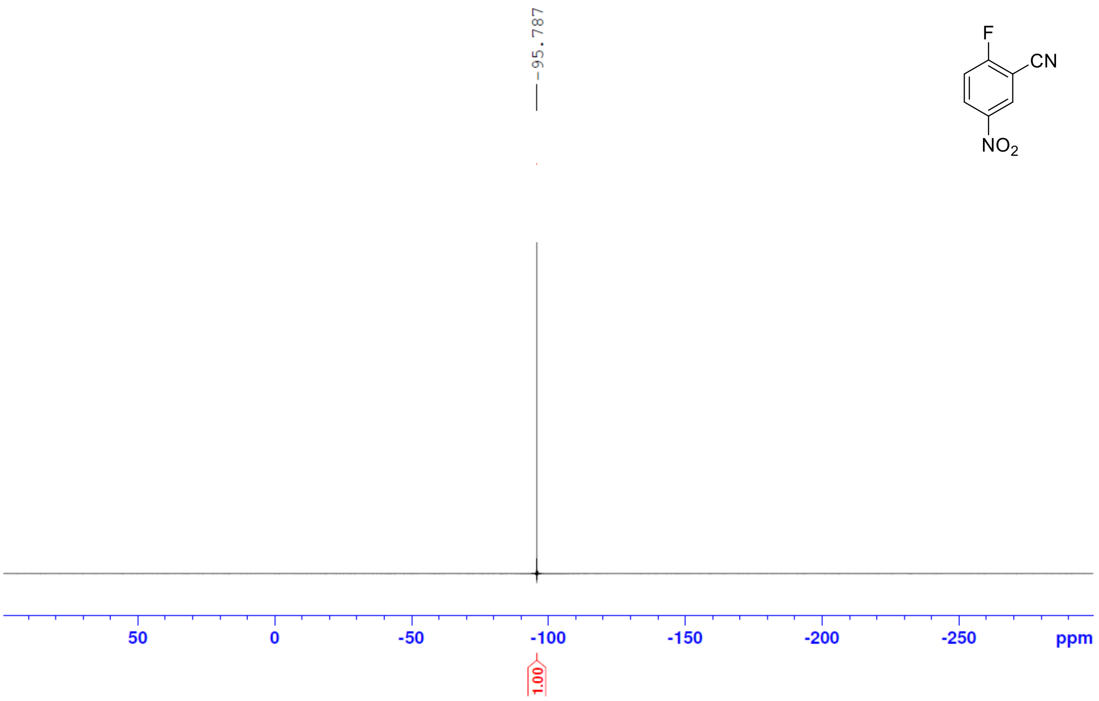

$^{19}F\{^{1}H\}$ NMR spectrum of 2-fluoro-5-nitrobenzonitrile **3r** (377 MHz, $CDCl_3$, 25 °C).

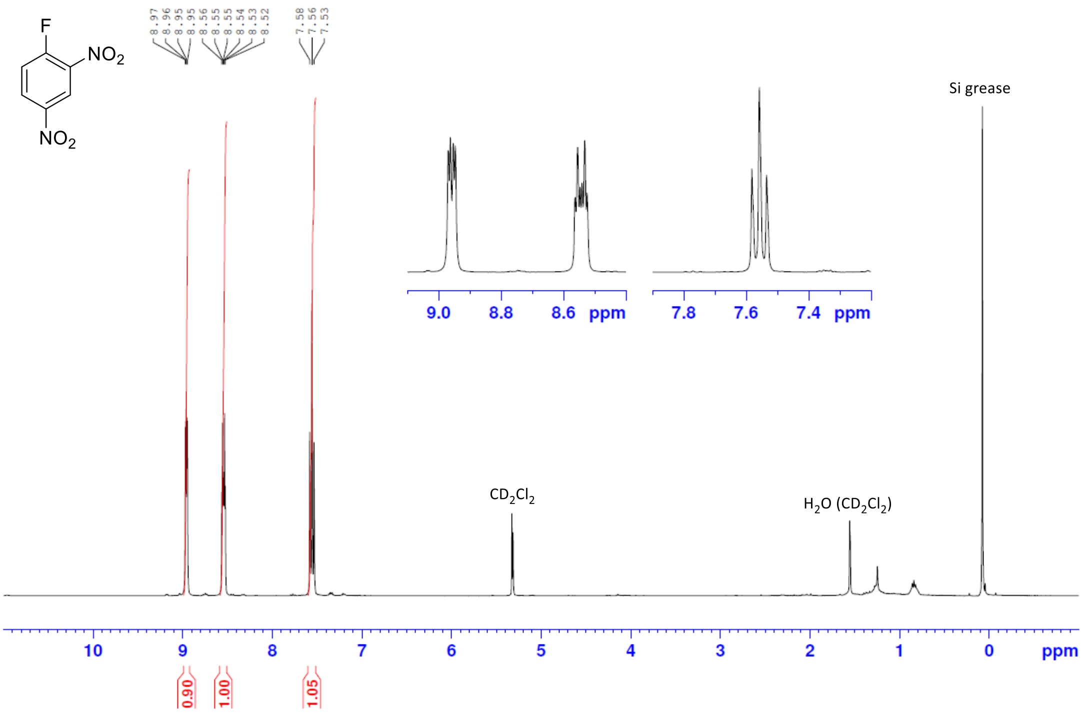

^{1}H NMR spectrum of 2,4-dinitrofluorobenzene **3s** (400 MHz, CD_2Cl_2, 25 °C).

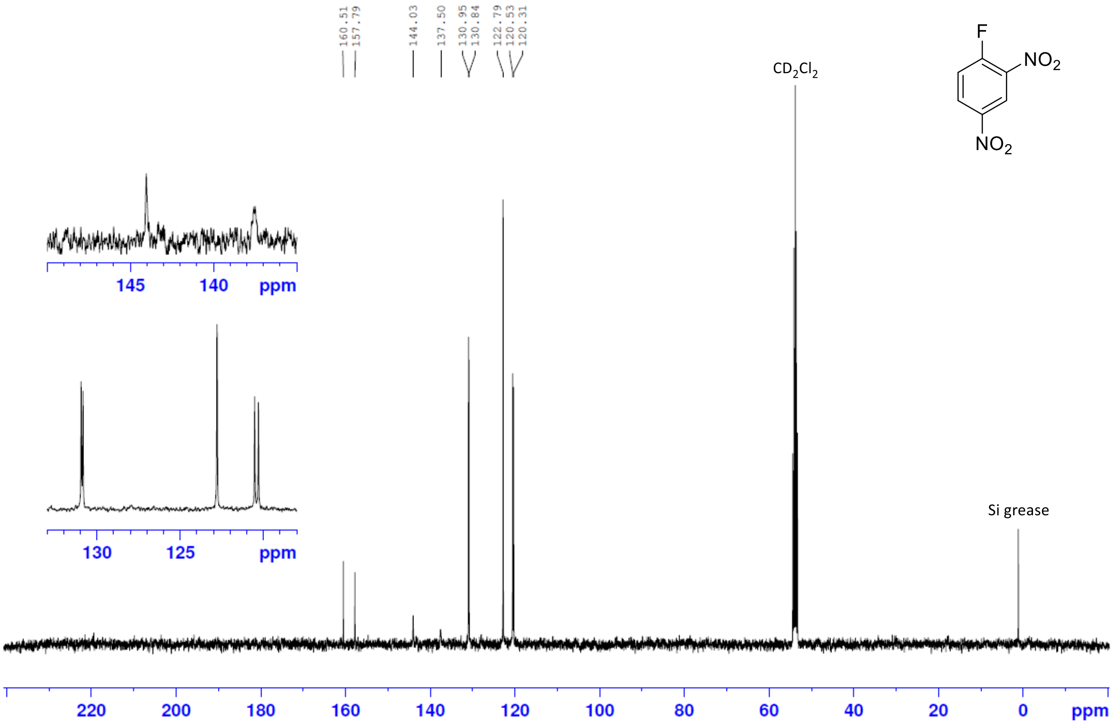

^{13}C{^{1}H} NMR spectrum of 2,4-dinitrofluorobenzene **3s** (101 MHz, CD_2Cl_2, 25 °C).

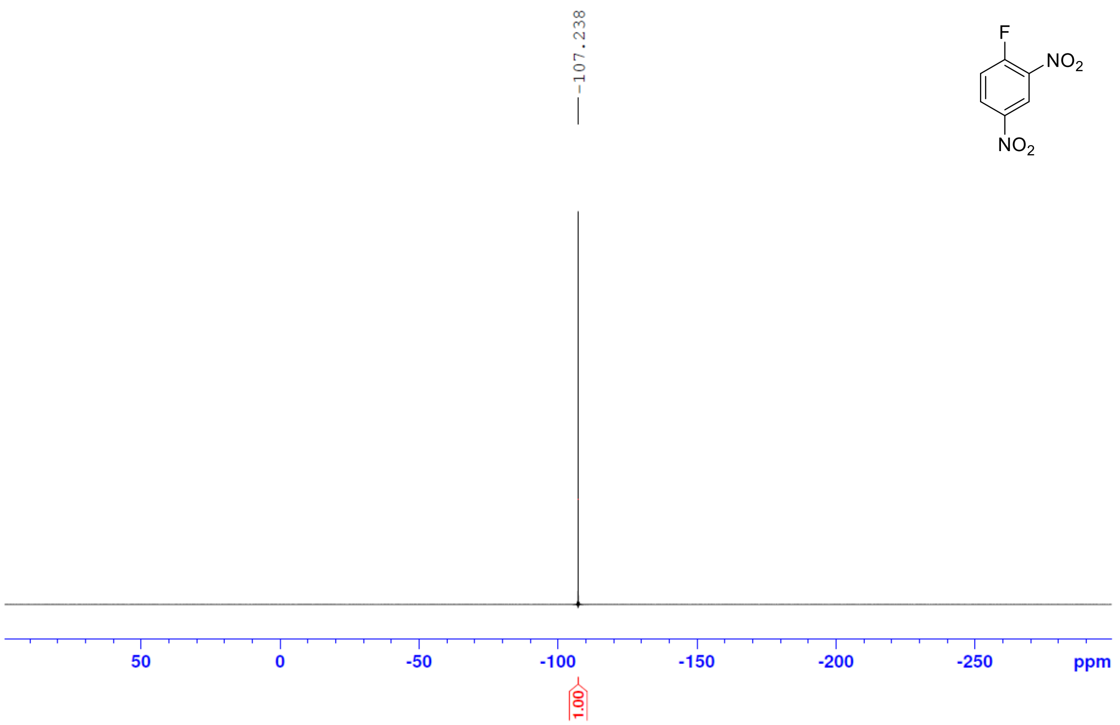

$^{19}F\{^{1}H\}$ NMR spectrum of 2,4-dinitrofluorobenzene **3s** (377 MHz, CD_2Cl_2, 25 °C).

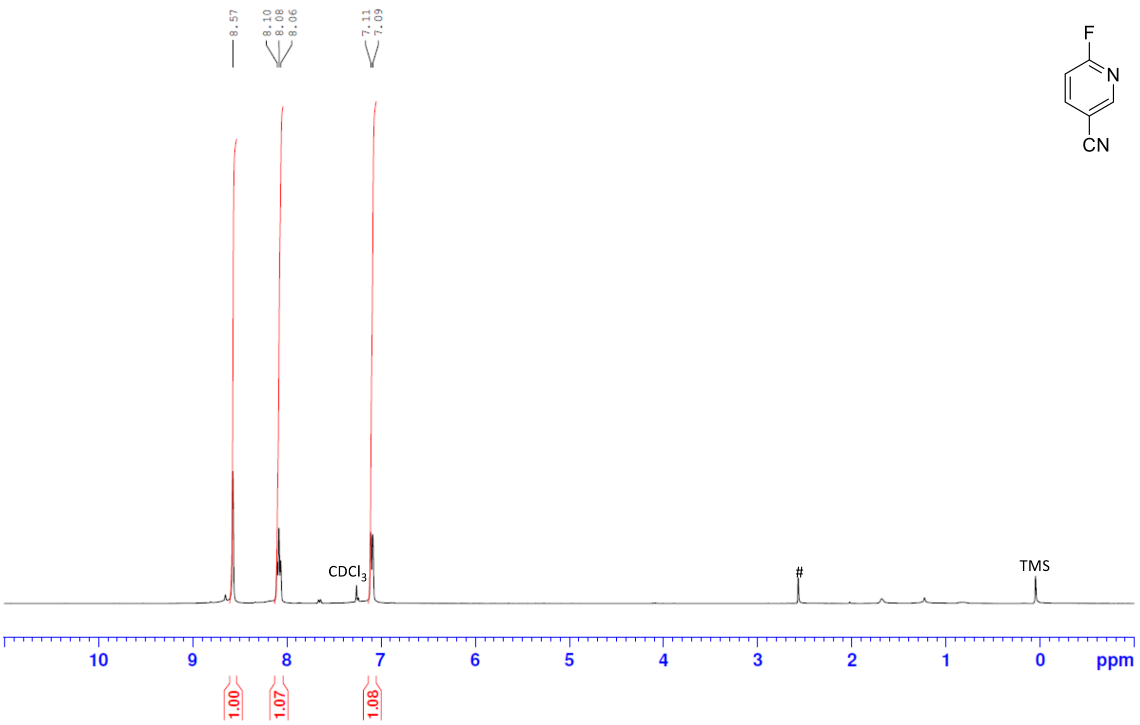

^{1}H NMR spectrum of 6-fluoronicotinonitrile **3t** (400 MHz, $CDCl_3$, 25 °C),

Note: # is an unidentified impurity showing a cross peak at 13.3 ppm in the ^{1}H-^{13}C -HSQC spectrum.

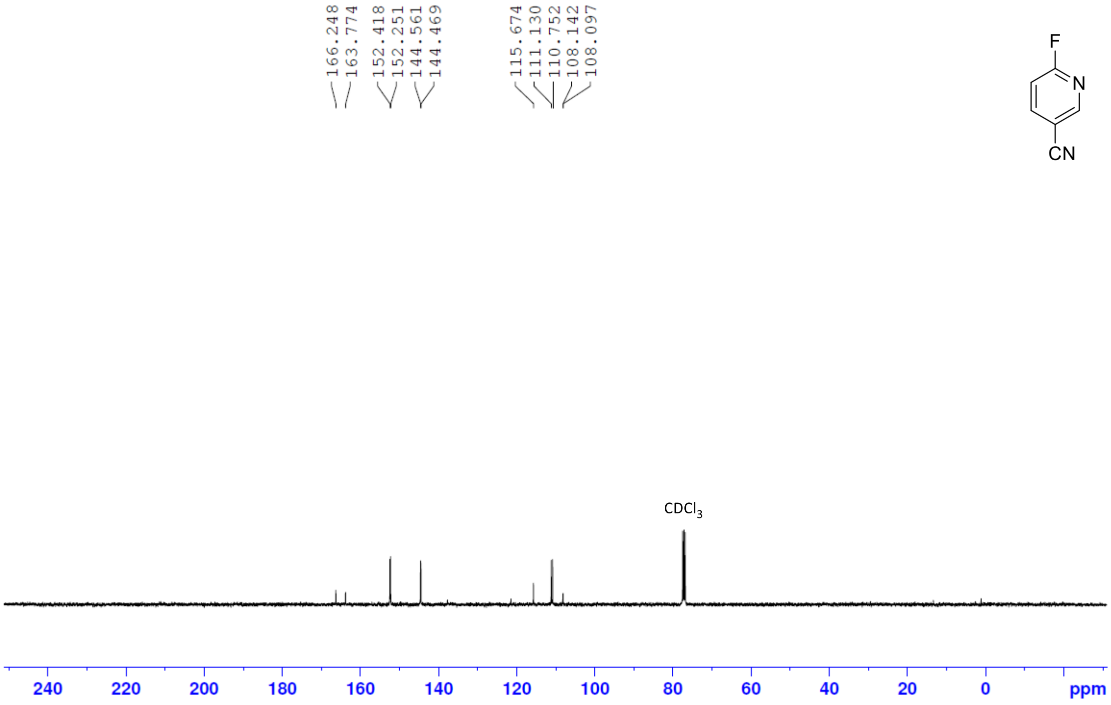

$^{13}C\{^{1}H\}$ NMR spectrum of 6-fluoronicotinonitrile **3t** (101 MHz, $CDCl_3$, 25 °C).

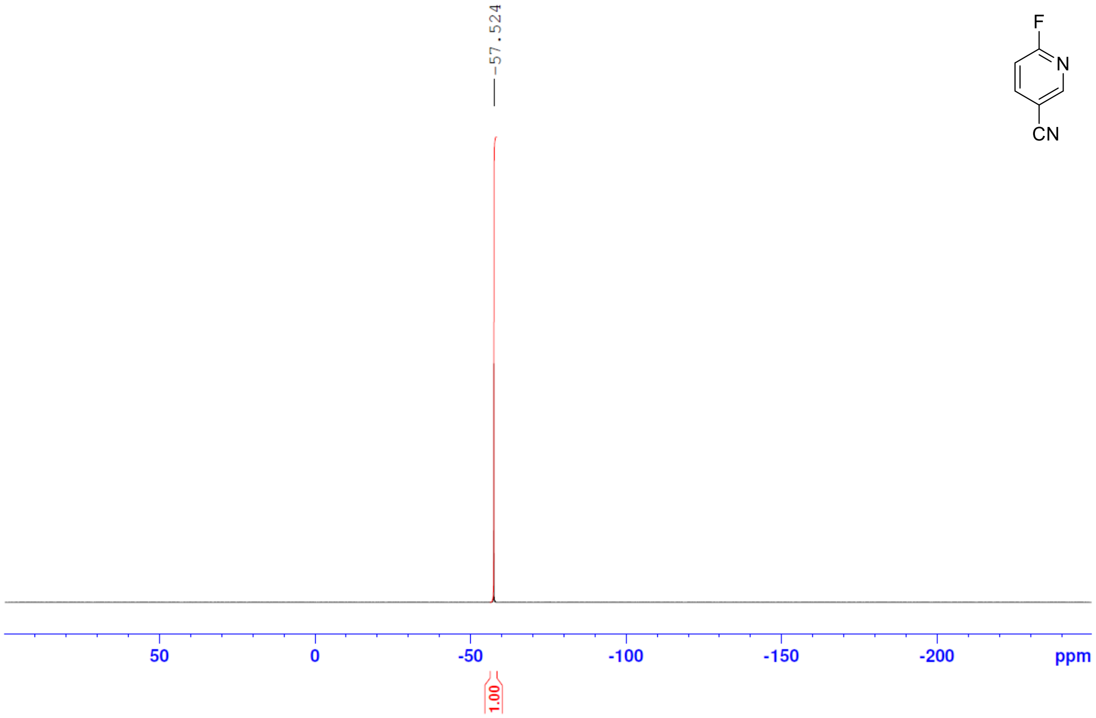

^{19}F NMR spectrum of 6-fluoronicotinonitrile **3t** (377 MHz, $CDCl_3$, 25 °C).

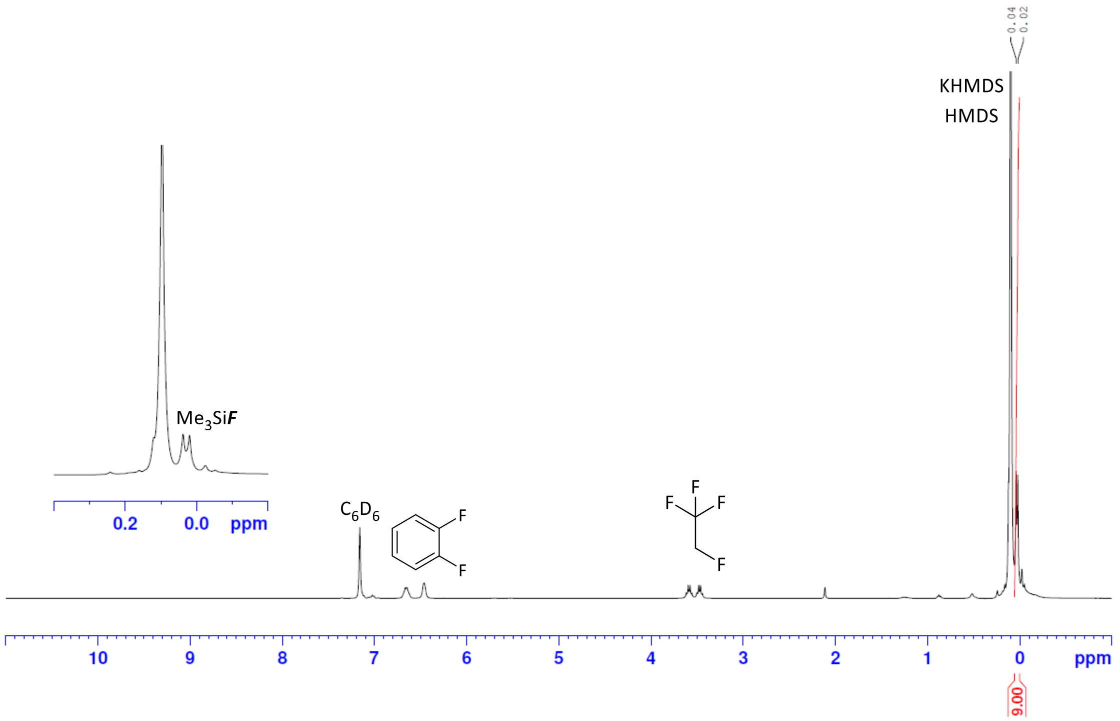

In situ ^{1}H NMR spectrum of the synthesis mixture containing trimethylfluorosilane **3u** (400 MHz, C_6D_6, 25 °C).

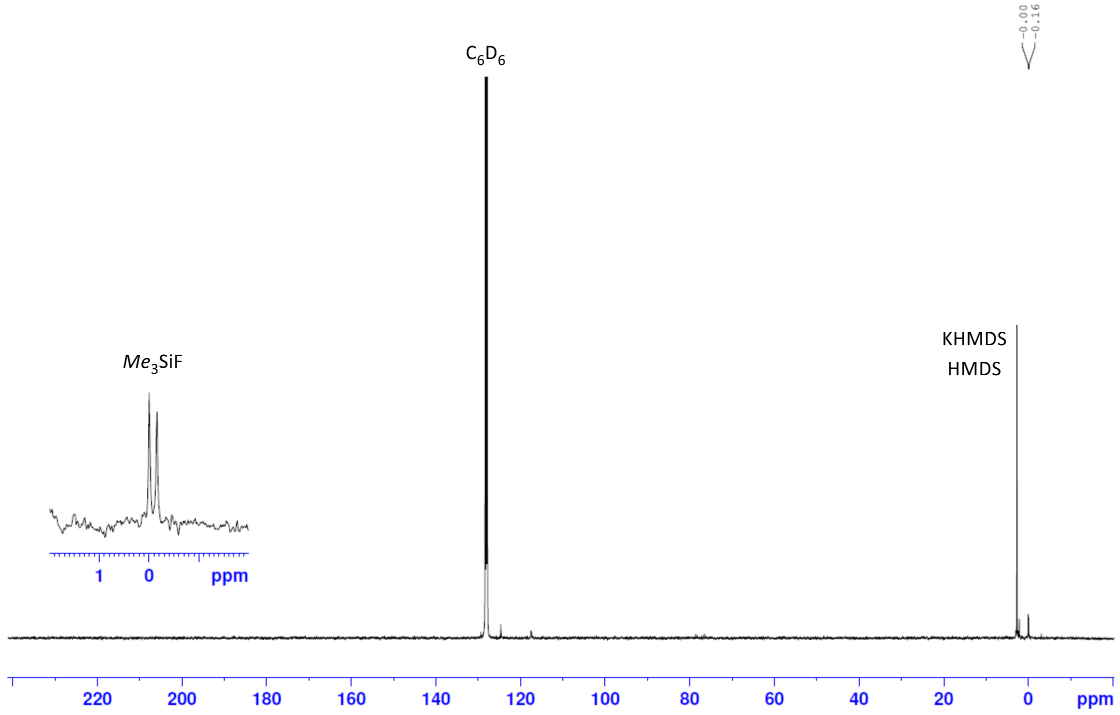

In situ $^{13}C\{^{1}H\}$ NMR spectrum of the synthesis mixture containing trimethylfluorosilane **3u** (101 MHz, C_6D_6, 25 °C).

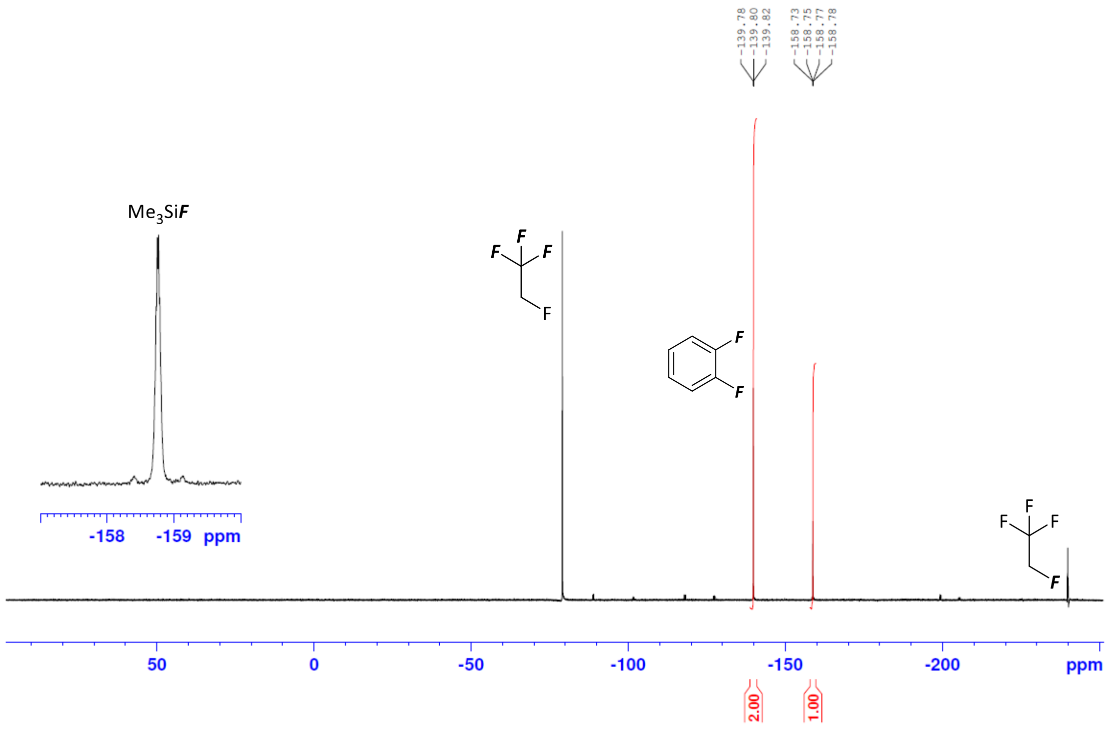

In situ quantitative ^{19}F NMR spectrum of the synthesis mixture containing trimethylfluorosilane **3u** *via* trimethylchlorosilane (377 MHz, C_6D_6, 25 °C).

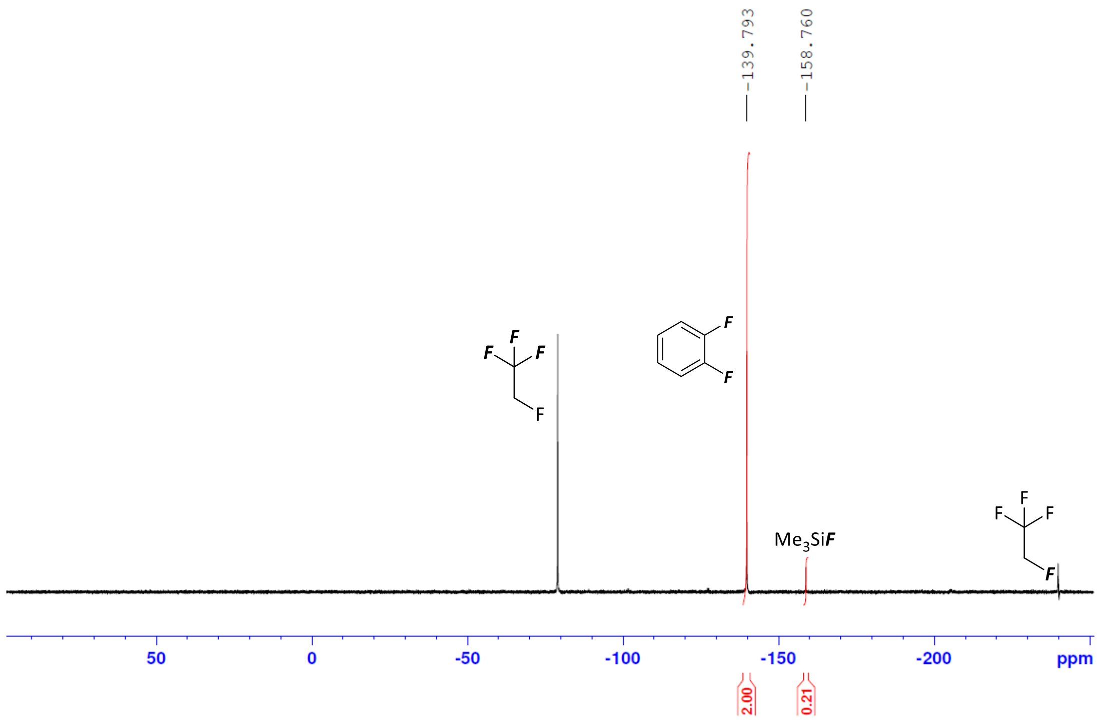

In situ quantitative ^{19}F NMR spectrum of the synthesis mixture containing trimethylfluorosilane **3u** without trimethylchlorosilane (377 MHz, C_6D_6, 25 °C).

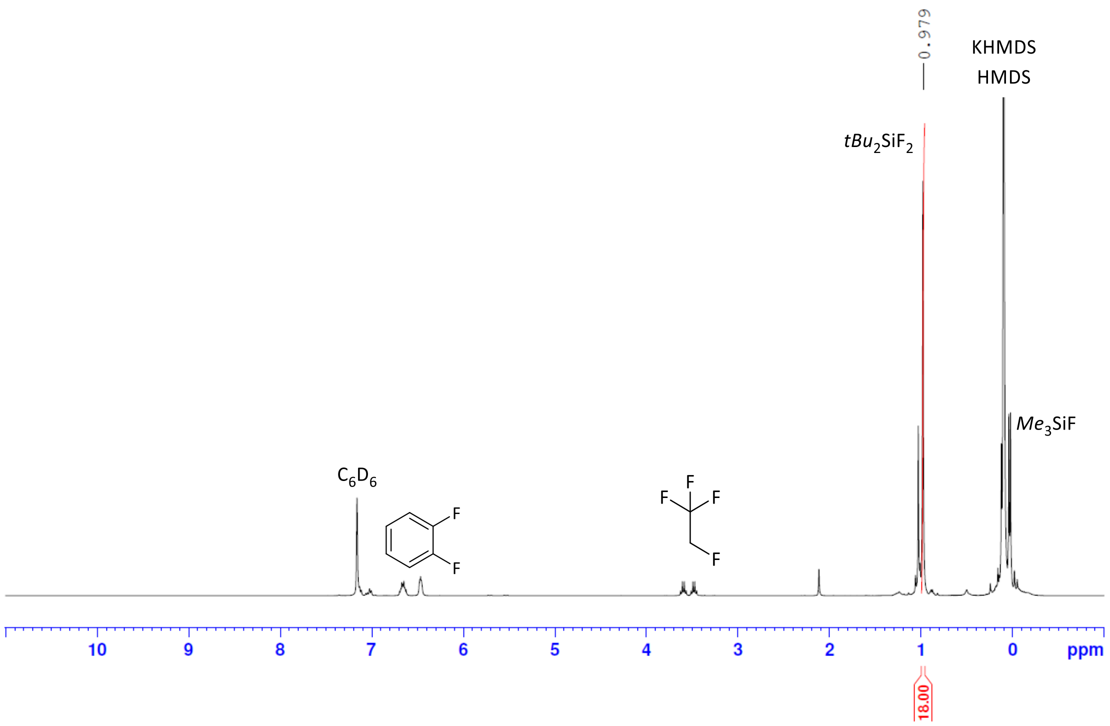

In situ ^{1}H NMR spectrum of the synthesis mixture containing di-*tert*-butyldifluorosilane **3v** (400 MHz, C_6D_6, 25 °C).

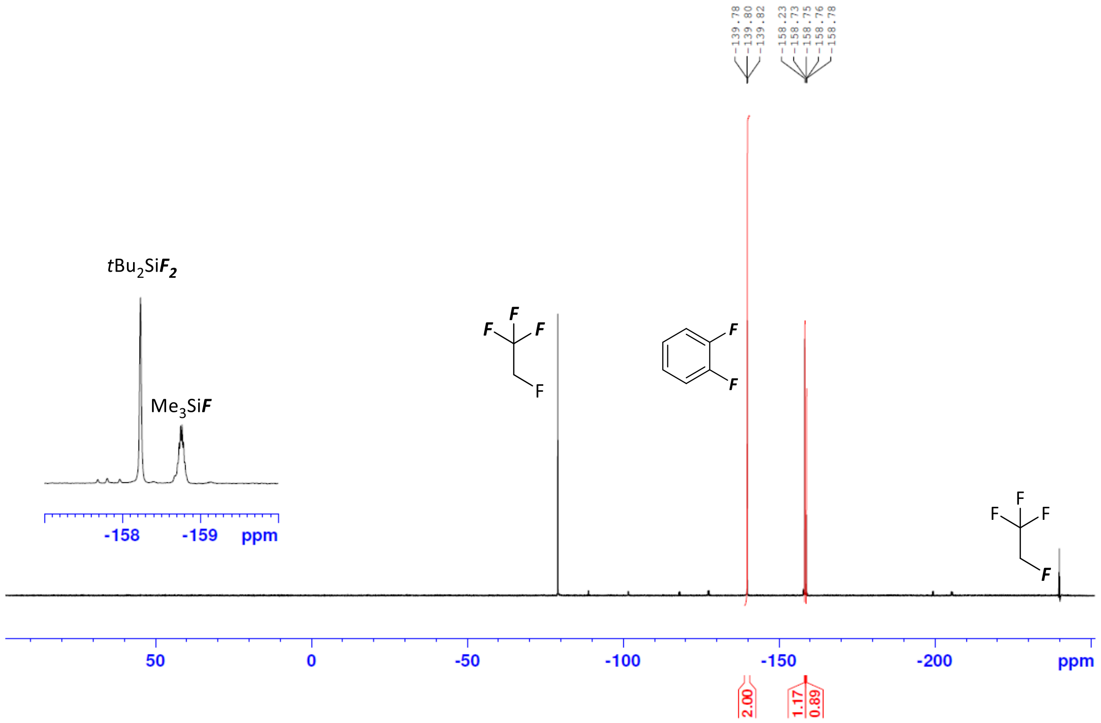

In situ quantitative ^{19}F NMR spectrum of the synthesis mixture containing di-*tert*-butyldifluorosilane **3v** (377 MHz, C_6D_6, 25 °C).

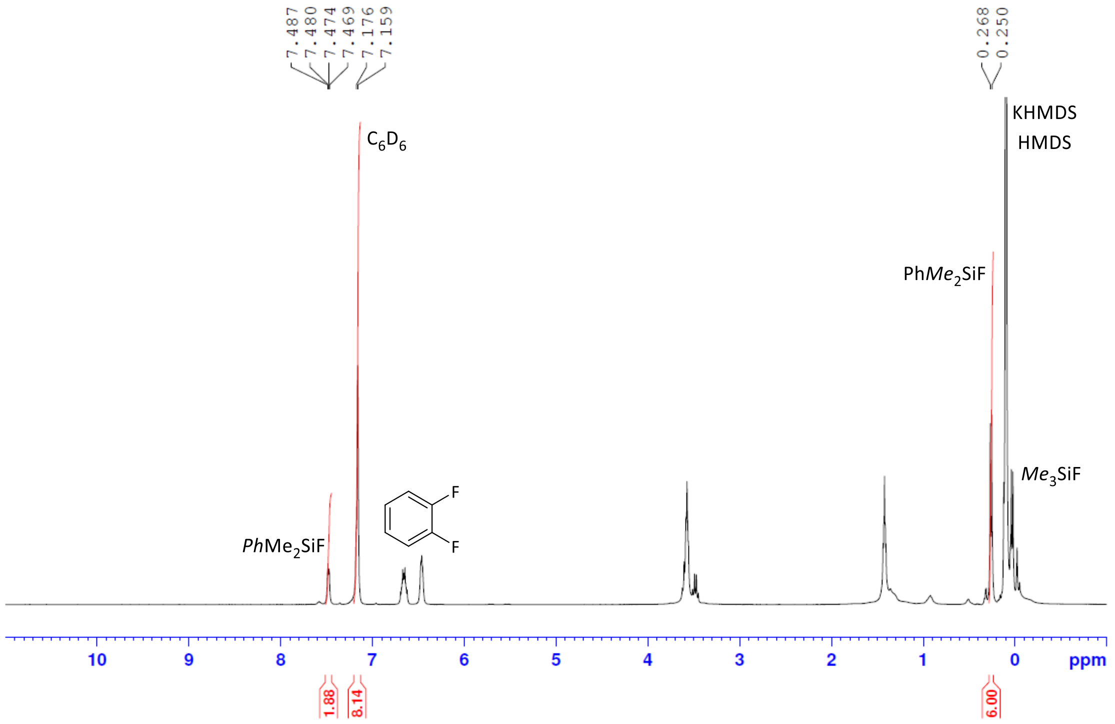

In situ ^{1}H NMR spectrum of the synthesis mixture containing fluorodimethylphenylsilane **3w** (400 MHz, C_6D_6, 25 °C).

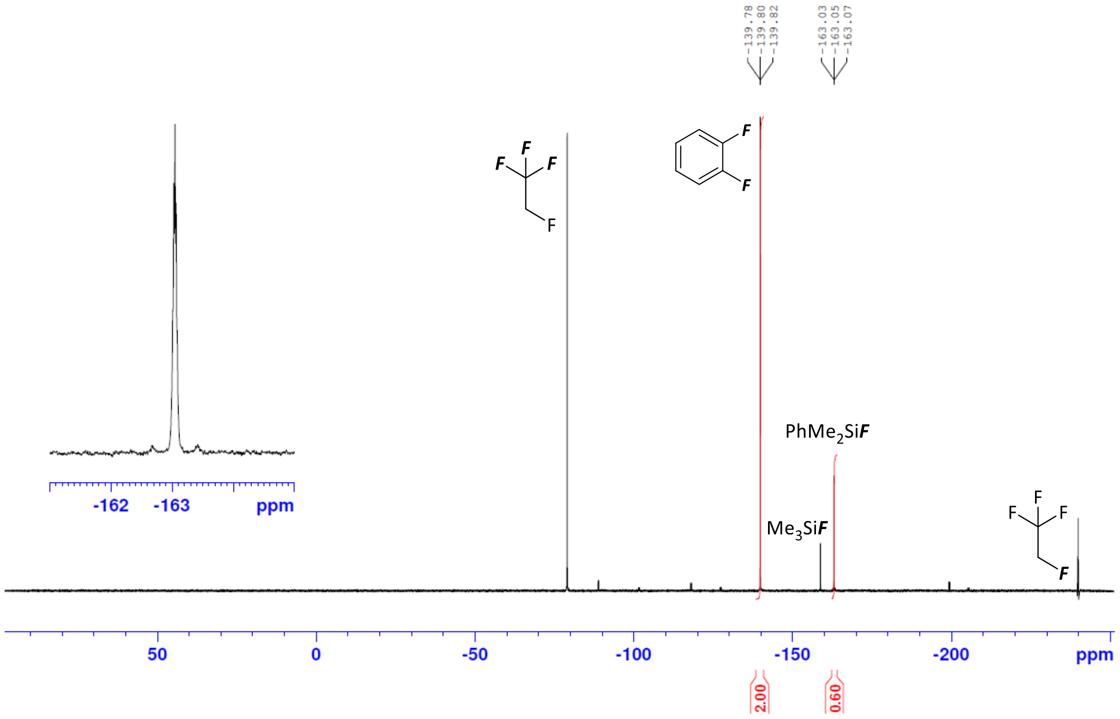

In situ quantitative ^{19}F NMR spectrum of the synthesis mixture containing fluorodimethylphenylsilane **3w** (377 MHz, C_6D_6, 25 °C).

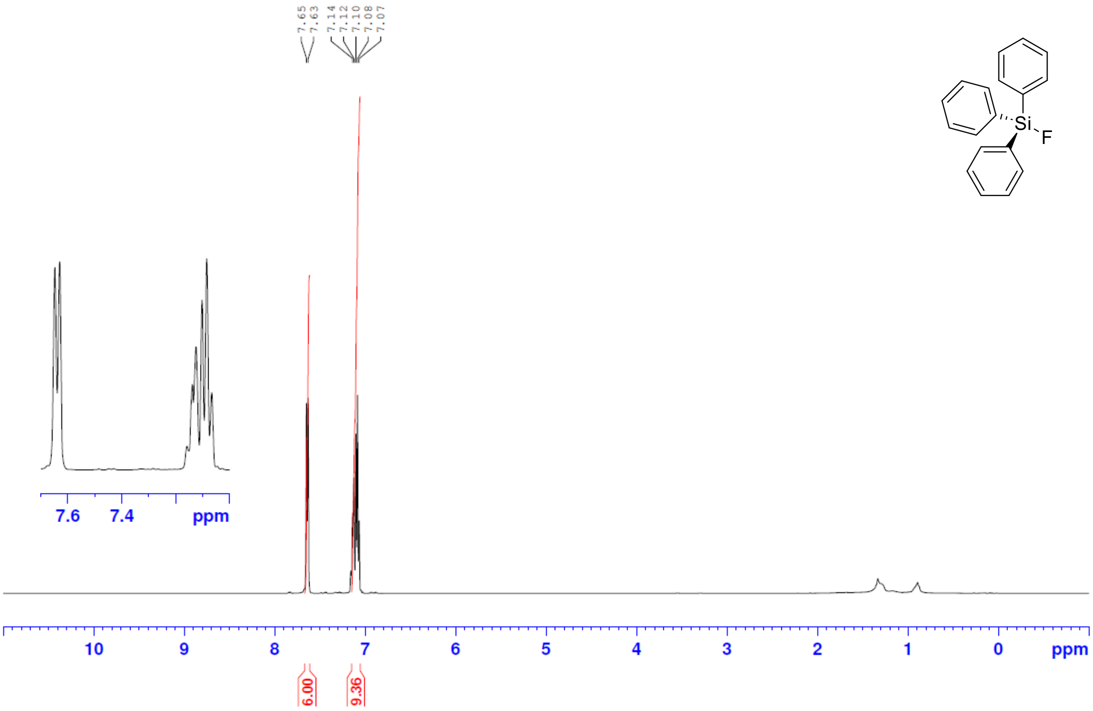

^{1}H NMR spectrum of fluorotriphenylsilane **3x** (400 MHz, C_6D_6, 25 °C).

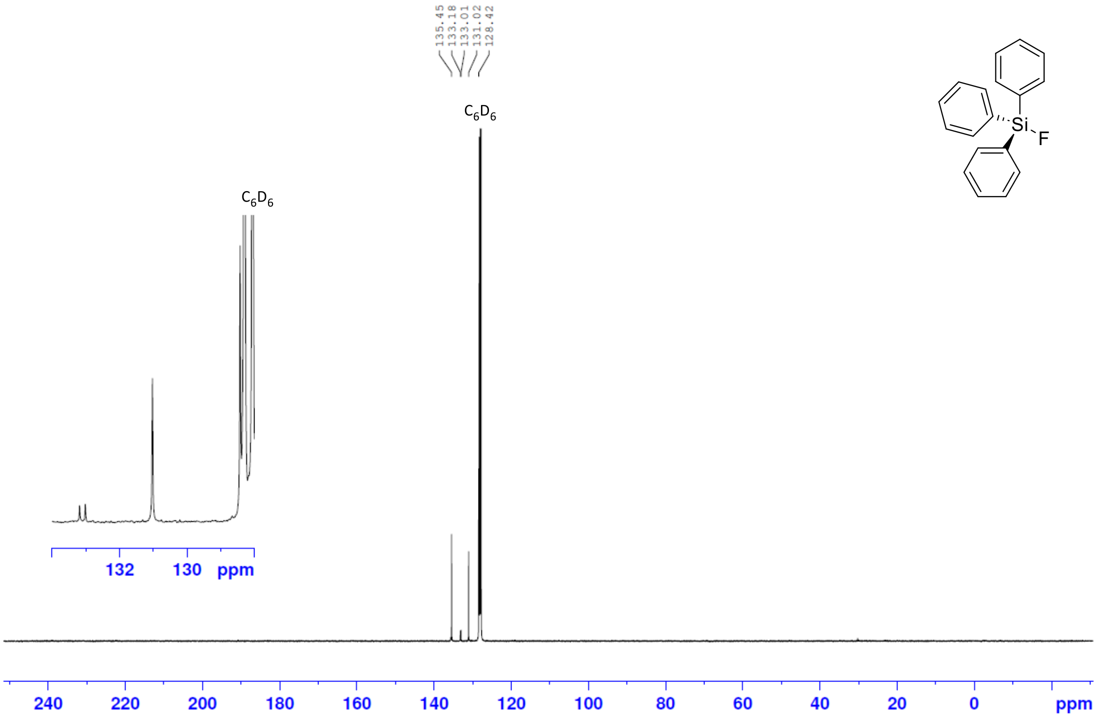

^{13}C{^{1}H} NMR spectrum of fluorotriphenylsilane **3x** (101 MHz, C_6D_6, 25 °C).

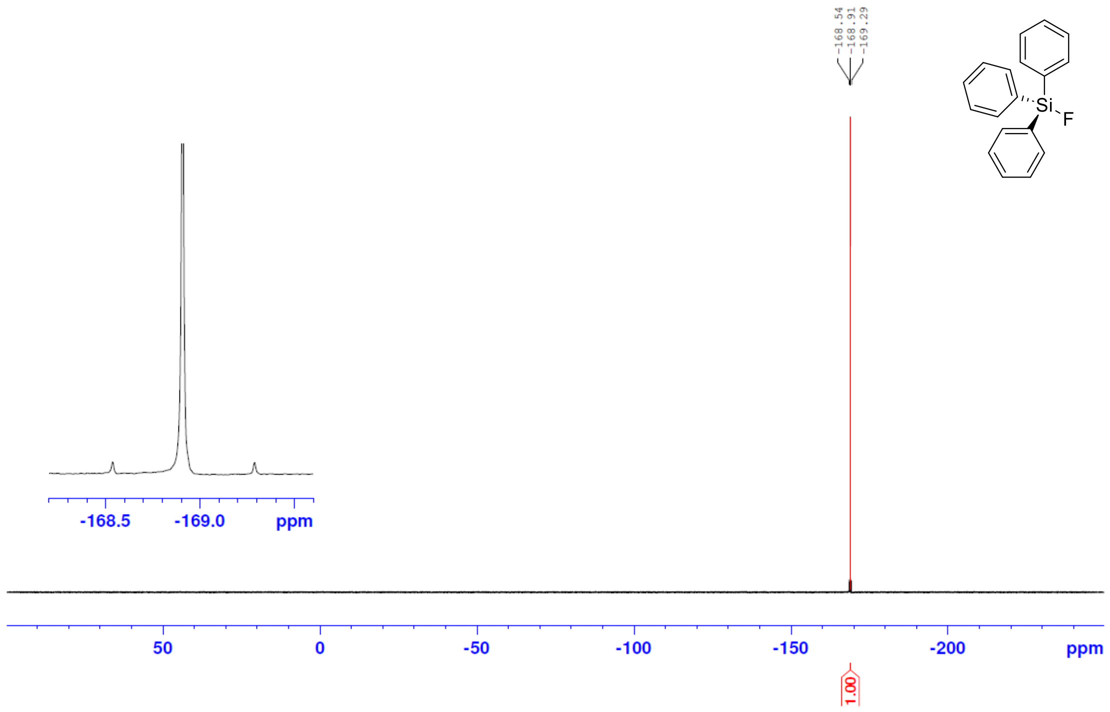

^{19}F NMR spectrum of fluorotriphenylsilane **3x** (377 MHz, C_6D_6, 25 °C).

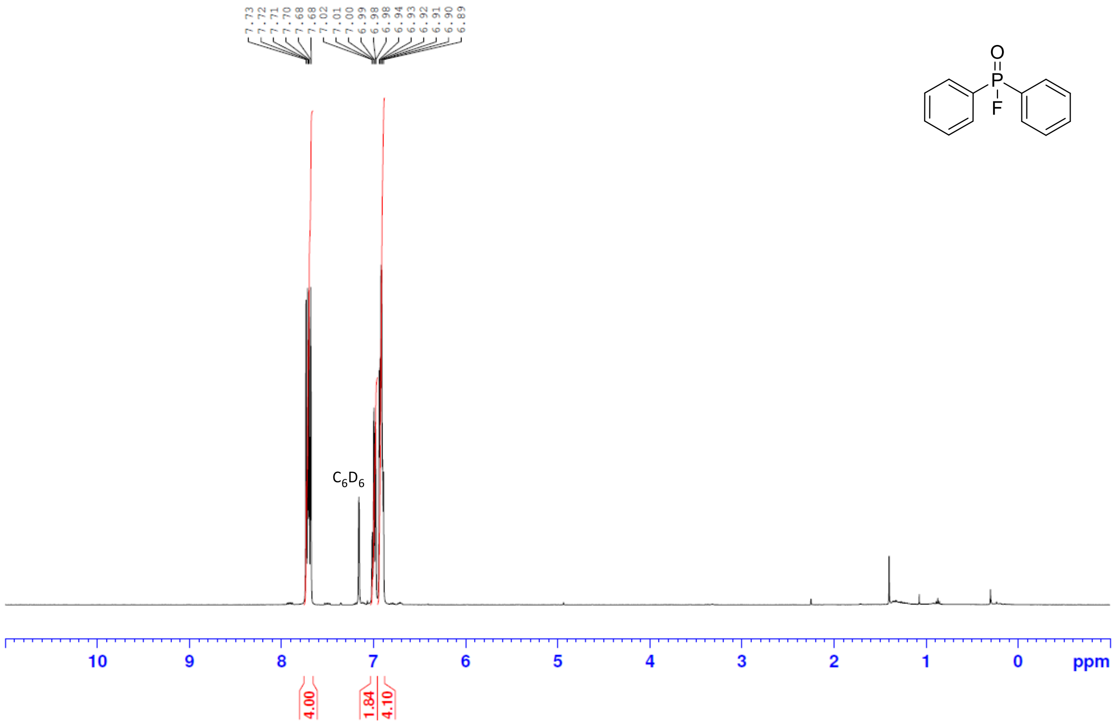

^{1}H NMR spectrum of diphenylphosphinic fluoride **3y** (400 MHz, C_6D_6, 25 °C).

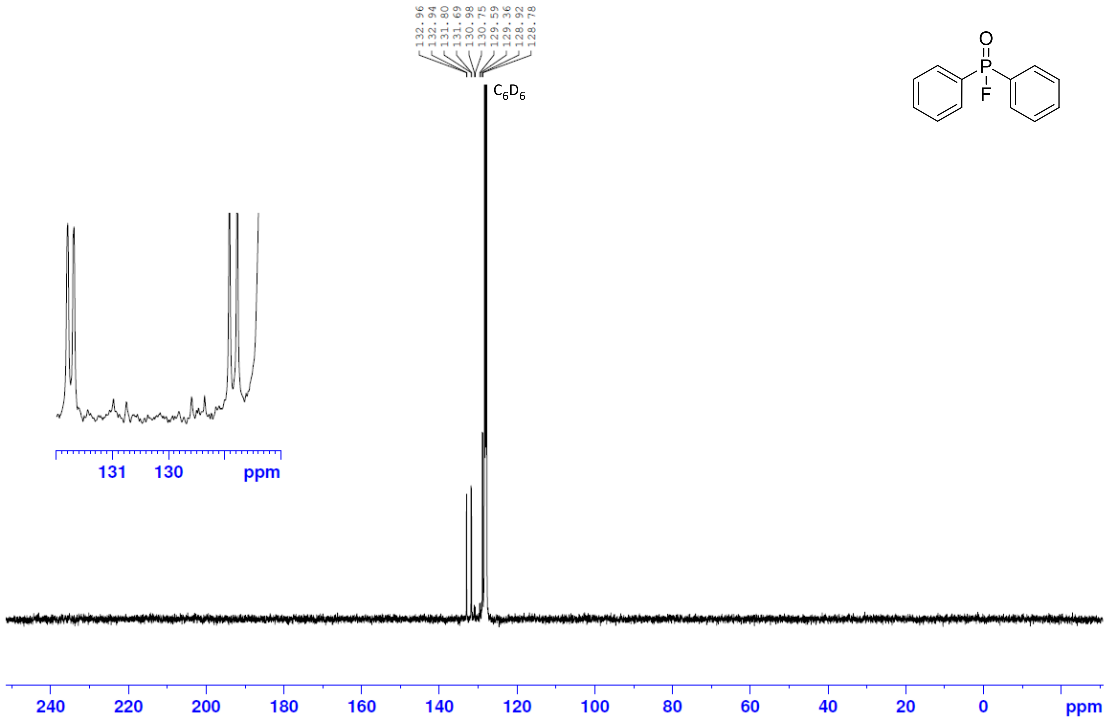

$^{13}C\{^{1}H\}$ NMR spectrum of diphenylphosphinic fluoride **3y** (101 MHz, C_6D_6, 25 °C).

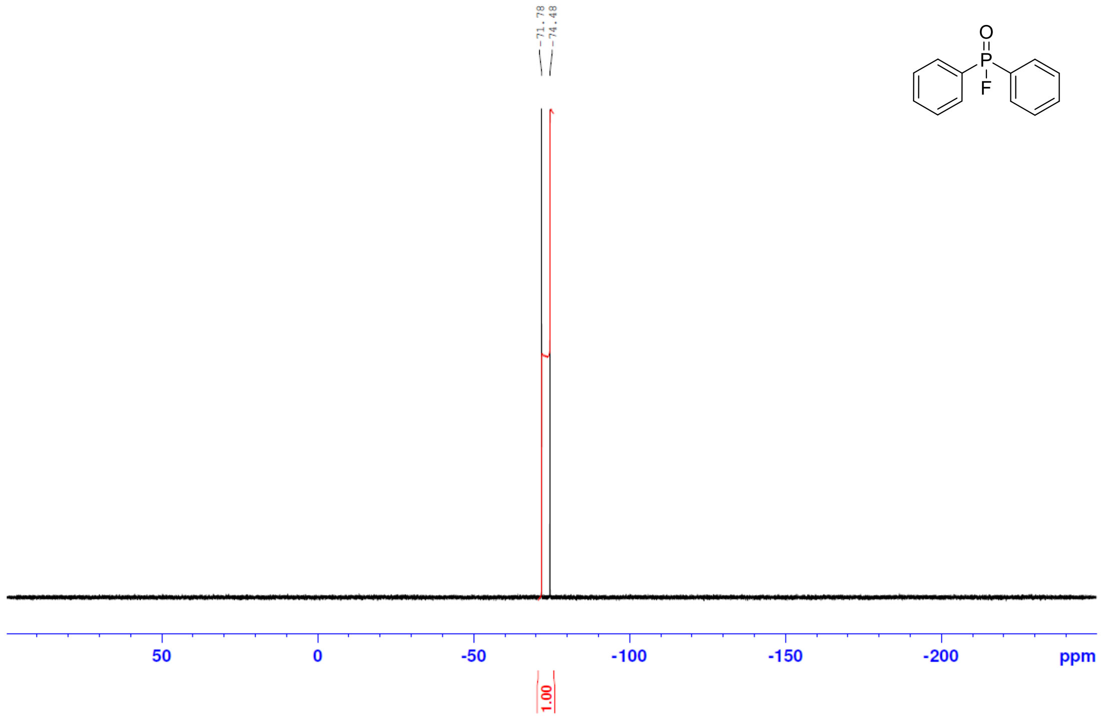

$^{19}F\{^{1}H\}$ NMR spectrum of diphenylphosphinic fluoride **3y** (377 MHz, C_6D_6, 25 °C).

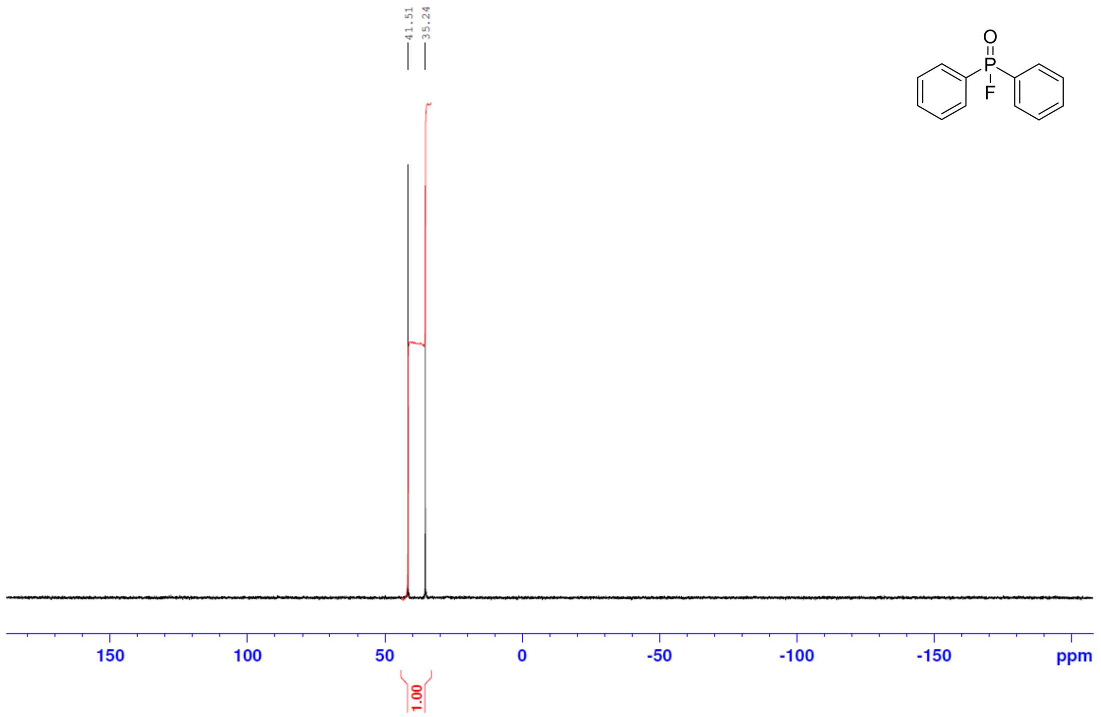

^{31}P{^{1}H} NMR spectrum of diphenylphosphinic fluoride **3y** (162 MHz, C_6D_6, 25 °C).

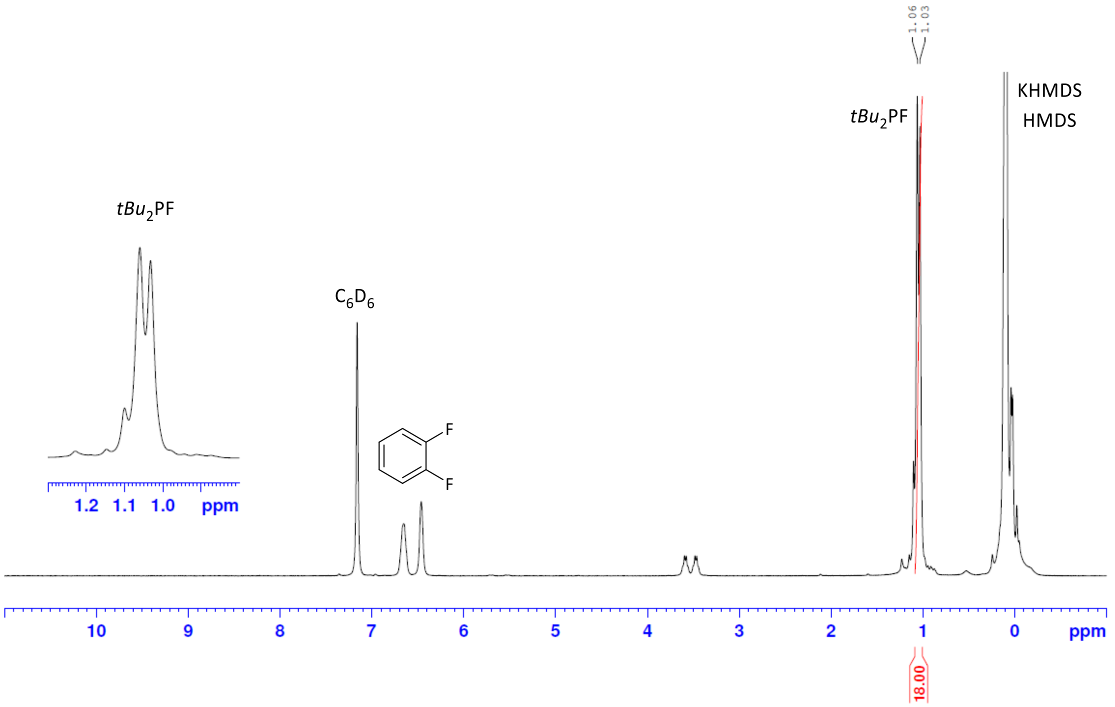

In situ ^{1}H NMR spectrum of di-*tert*-butylfluorophosphine **3z** (400 MHz, C_6D_6, 25 °C).

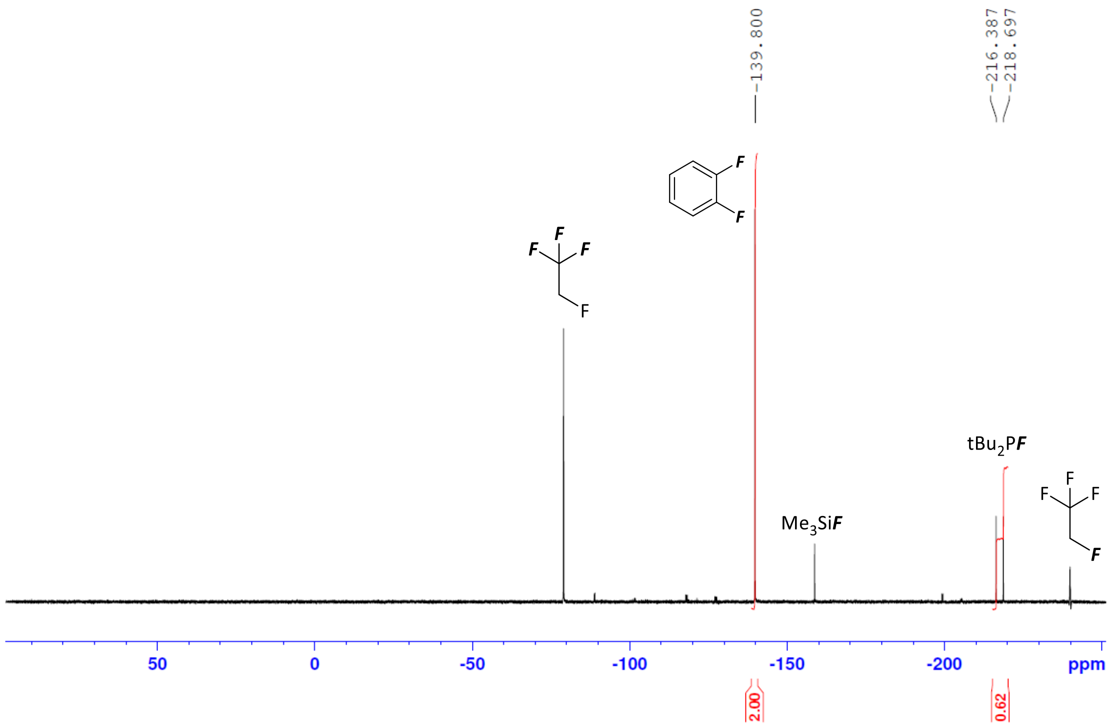

In situ ^{19}F NMR spectrum of di-*tert*-butylfluorophosphine **3z** (377 MHz, C_6D_6, 25 °C).

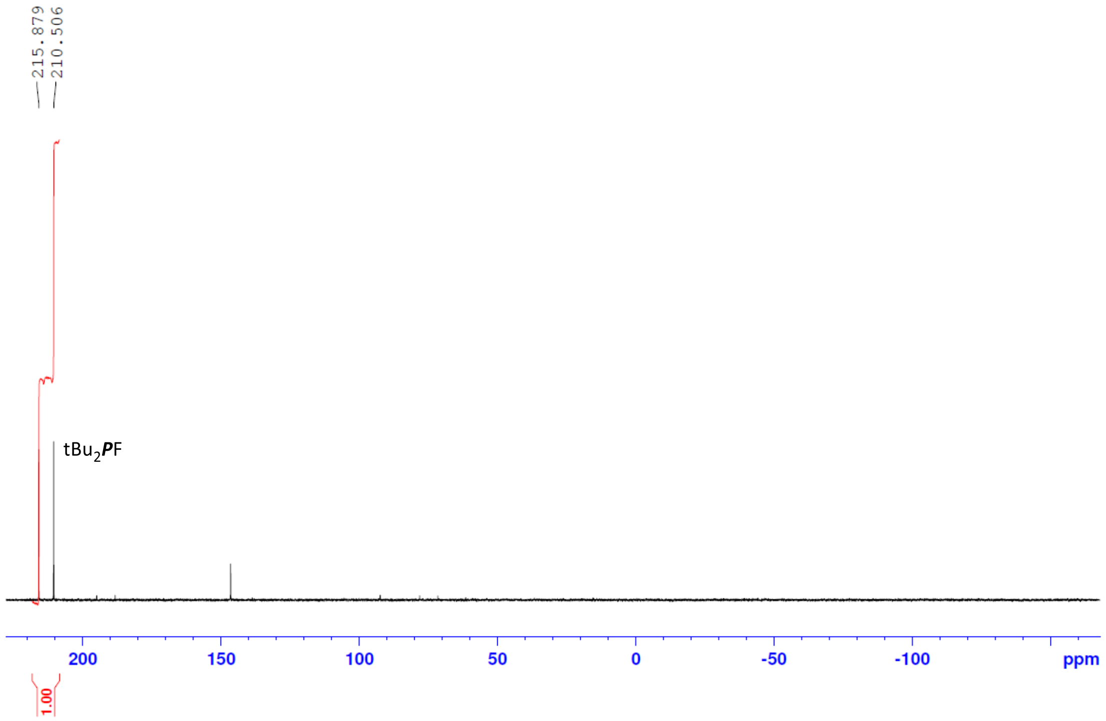

In situ $^{31}P\{^1H\}$ NMR spectrum of di-*tert*-butylfluorophosphine **3z** (162 MHz, C_6D_6, 25 °C).

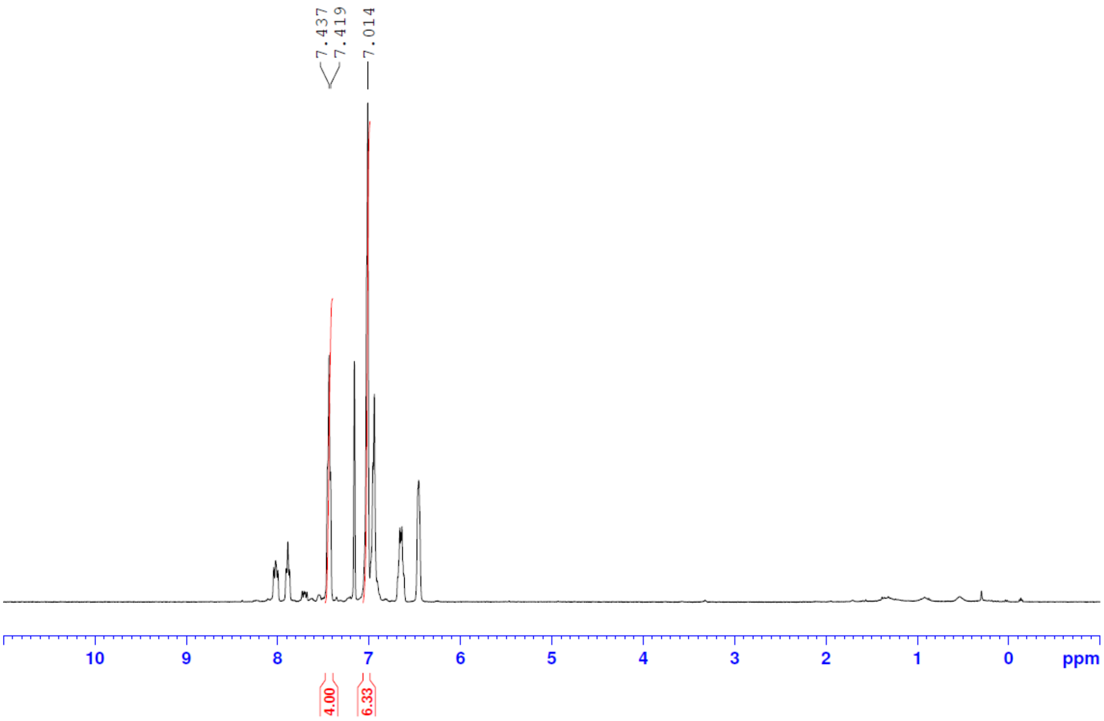

In situ ^{1}H NMR spectrum of diphenylfluorophosphine **3aa** (400 MHz, C_6D_6, 25 °C).

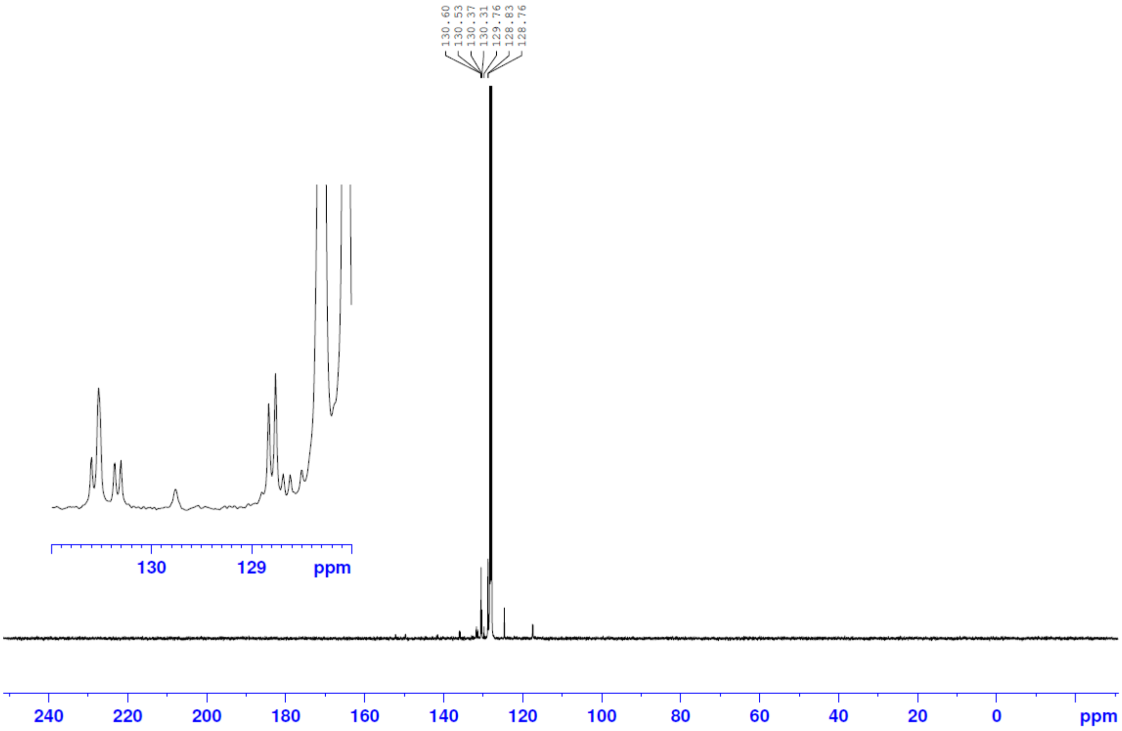

In situ ^{13}C{^{1}H} NMR spectrum of diphenylfluorophosphine **3aa** (101 MHz, C_6D_6, 25 °C).

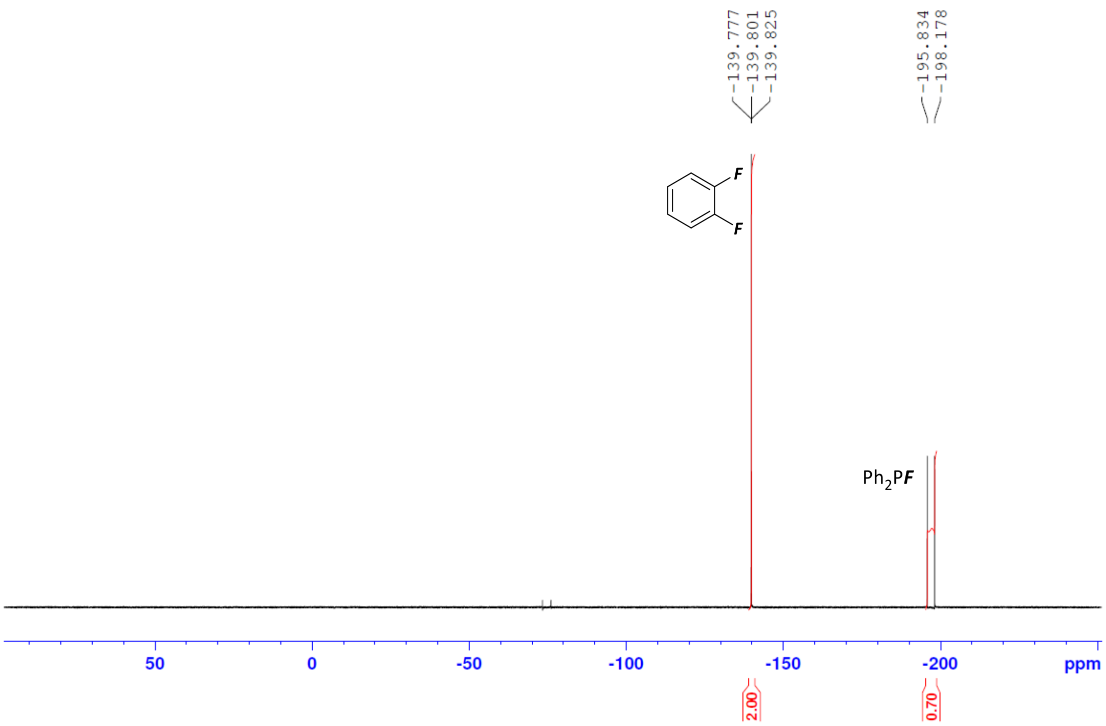

In situ ^{19}F NMR spectrum of diphenylfluorophosphine **3aa** (377 MHz, C_6D_6, 25 °C).

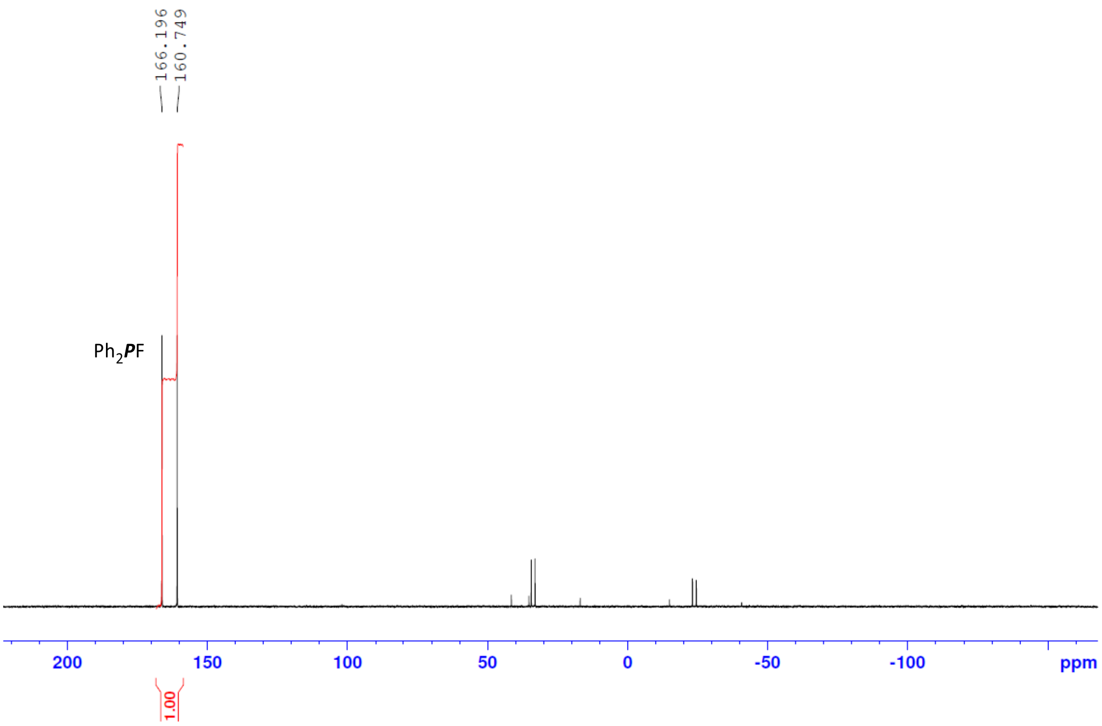

In situ ^{31}P{^{1}H} NMR spectrum of diphenylfluorophosphine **3aa** (162 MHz, C_6D_6, 25 °C).

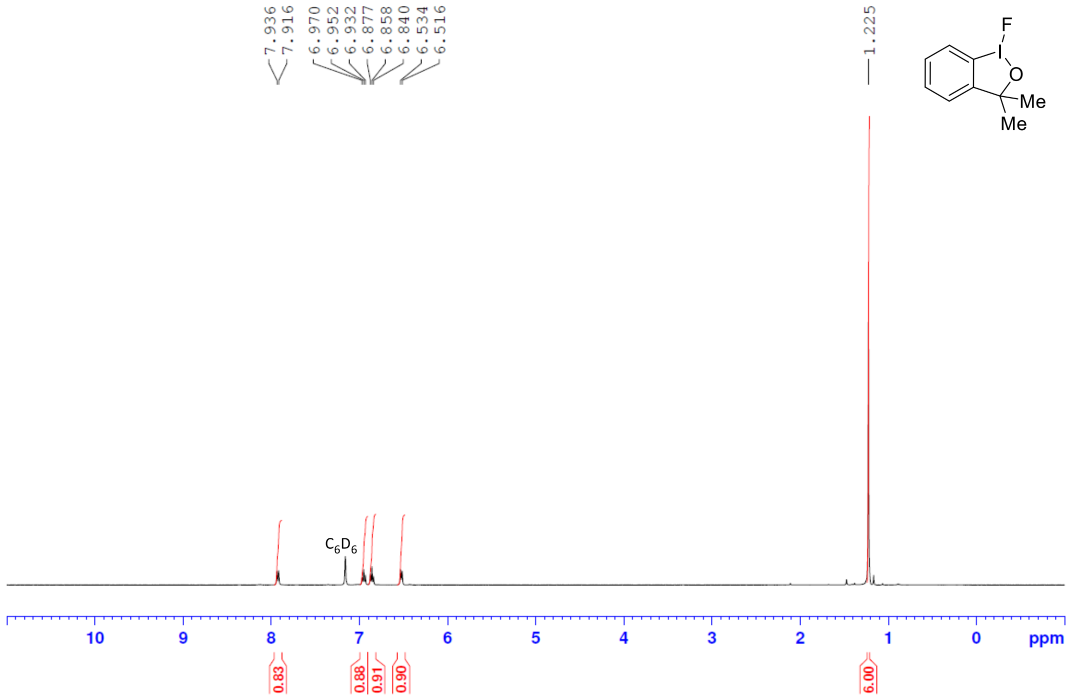

^{1}H NMR spectrum of 1-fluoro-3,3-dimethyl-1,2-benziodoxole **3ab** (400 MHz, C_6D_6, 25 °C).

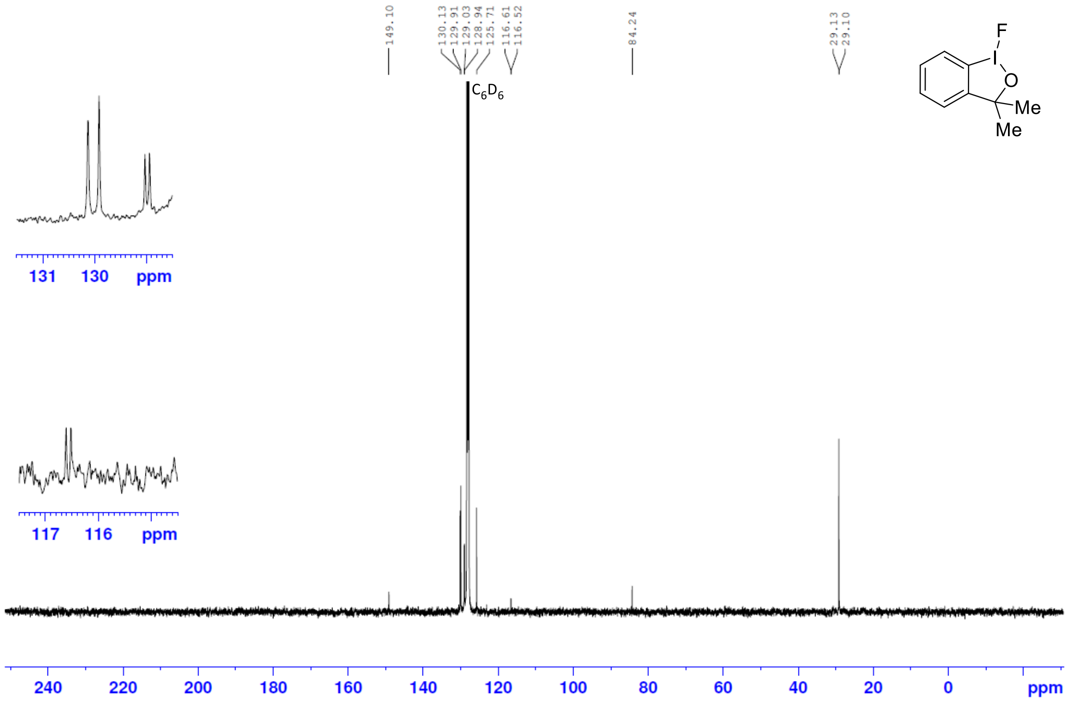

^{13}C{^{1}H} NMR spectrum of 1-fluoro-3,3-dimethyl-1,2-benziodoxole **3ab**

(101 MHz, C_6D_6, 25 °C).

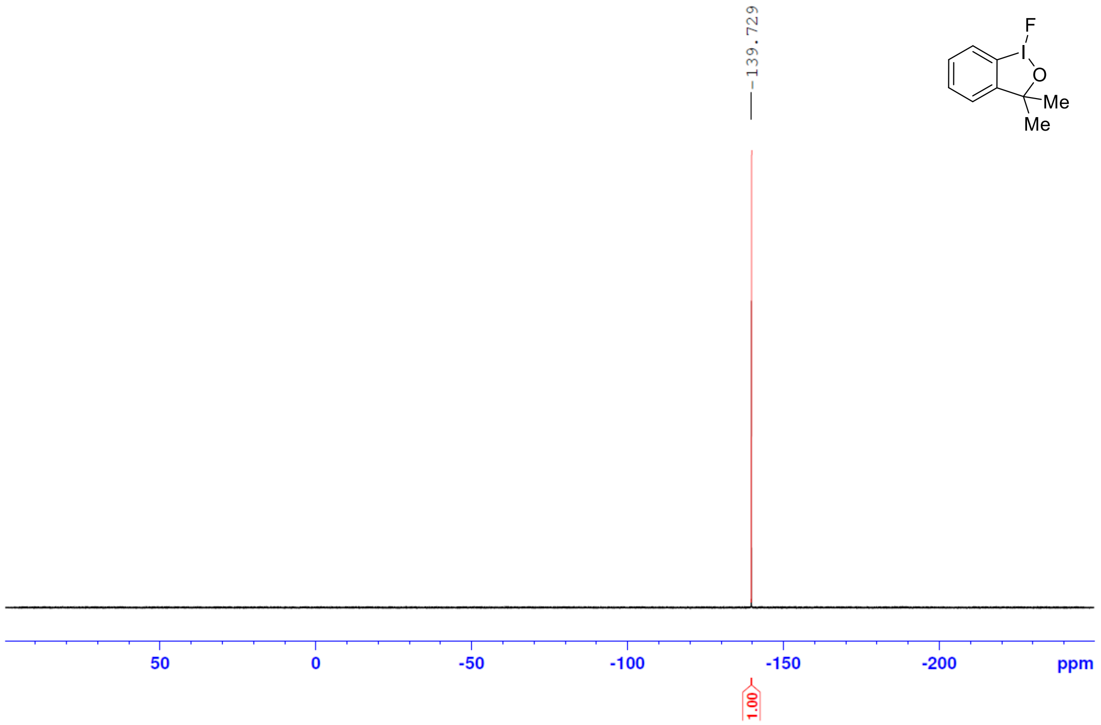

^{19}F NMR spectrum of 1-fluoro-3,3-dimethyl-1,2-benziodoxole **3ab** (377 MHz, C_6D_6, 25 °C).

15. Single-crystal X-ray crystallography

15.1. X-Ray data

Compound	**1a**	**2a**
CCDC No.	2394846	2394827
Formula	$C_{14}H_5F_9$	$C_{14}H_4F_8$
formula weight	344.18	324.17
colour, habit	colourless block	clear colourless block
temperature / K	173	173
crystal system	Monoclinic	monoclinic
space group	P2/*n* (no. 14)	*C*2/*c* (no. 15)
a / Å	10.0933(5)	9.3153(3)
b / Å	11.0359(5)	11.2248(3)
c / Å	11.0303(5)	11.2002(3)
α / deg	90	90
β / deg	90.608(4)	90.512(3)
γ / deg	90	90
V / Å^3	1228.58(10)	1171.07(6)
Z	4	4[c]
Dc / g cm^{-3}	1.861	1.839
radiation used	Mo-Kα	Cu-Kα
μ / mm^{-1}	0.204	1.792
no. of unique reflections		
measured (R_{int})	2804 (0.0254)	1152 (0.0249)
obs, |*Fo*|>4σ(|*Fo*|)	2042	1032
completeness (%)[a]	100	99.9
no. of variables	301	101
R_1 (obs), wR_2 (all)[b]	0.0450, 0.1186	0.0489, 0.1279

Supplementary Table 18: Summary of the crystallographic data for the structures of **1a** and **2a**.

The data were collected using Agilent Xcalibur 3 E (**1a**) and Xcalibur PX Ultra A (**2a**) diffractometers, and the structures were solved and refined using the OLEX2, SHELX-2013 program systems.[27,28,29] [a] Completeness to 0.84 Å resolution. [b] $R_1 = \Sigma||F_o| - |F_c||/\Sigma|F_o|$; $wR_2 = \{\Sigma[w(F_o^2 - F_c^2)^2] / \Sigma[w(F_o^2)^2]\}^{1/2}$; $w^{-1} = \sigma_2(F_o^2) + (aP)^2 + bP$. [c] The molecule has crystallographic C_2 symmetry.

15.2. Refinement details

Compound 1a

The molecule of **1a** was found to be disordered. It was modelled in two orientations at a ratio of ca. 0.74:0.26. The two orientations were restrained to be similar and only the non-hydrogen atoms of the major orientation were modelled anisotropically, the rest were modelled isotropically.

Compound 2a

The molecule was found to be disordered across a crystallographic C_2 axis with atoms C1 and C2 lying on the rotation axis. Thus, this asymmetric unit contains half a molecule of **2a**.

15.3. Crystal structures

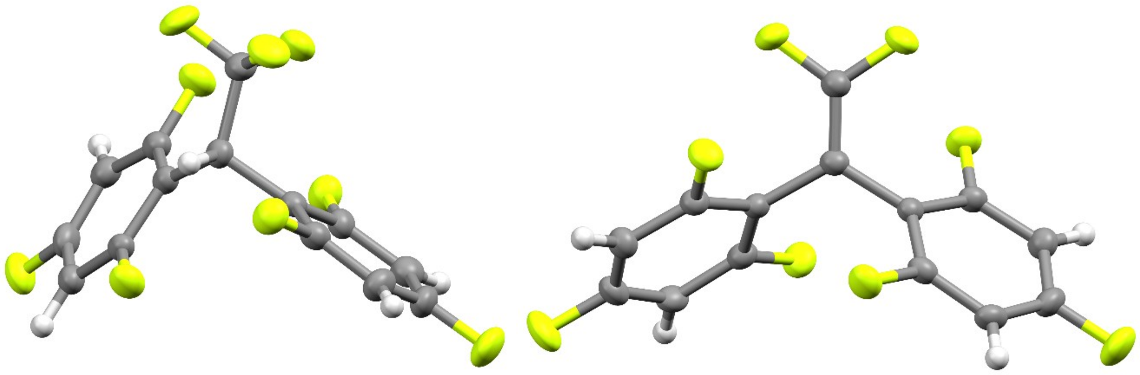

Supplementary Figure 89: Crystal structures of **1a** (left) and **2a** (right). Atoms in the minor occupancy orientation are hidden for clarity.

16. Computational chemistry – Calculations

<u>*Computational details:*</u>

All DFT computations were carried out with Gaussian16 (Revision C.01).[30] Geometry optimisations were performed using the hybrid exchange-correlation B3PW91 functional[31] including D3BJ dispersion corrections described by Grimme's D3 corrections[32] with added Becke-Johnson dampening.[33–36] A functional screening was performed (*vide infra*) using the following hybrid functionals: B3LYP-D3(BJ),[31,37,38,39] M06-2X-D3,[40] PBE0-D3(BJ),[41] ωB97X-D(2),[42,43] the *meta*-generalised gradient approximation (*meta*-GGA) functional M06-L-D3[44] and the GGA functional BP86-D3(BJ).[45,46]

Aldrichs' def2 split valence basis sets were used.[47,48]
BS1 is defined as follows: triple-ζ basis set with additional polarisation functions (def2-TZVP) was used for carbon and hydrogen atoms, and the triple-ζ basis set with double additional polarisation and diffuse functions (def2-TZVPPD) was used for nitrogen, oxygen, fluorine, silicon, and potassium atoms. No effective core potentials (ECPs) were used.
BS2 is defined as follows: Single point energy corrections of the optimised structures were performed using the larger quadruple-ζ basis set with double polarisation and diffuse functions (def2-QZVPPD).[49,50]

Geometry optimisations were performed without symmetry constraints (nosymm) and an improved numerical integration grid using a pruned grid with 99 radial shells and 590 angular points per shell (int=ultrafine) was used. Solvation was added to geometry optimisations employing toluene as the solvent using the SMD universal solvation model.[51] Def2-TZVPPD and def2-QZVPPD are not natively incorporated into the Gaussian suite of programs, and so these basis sets needed to be defined manually. The specific functions for each element (H, C, N, O, F, Si and K) were obtained from the Basis Set Exchange (https://www.basissetexchange.org/).[52,53,54]
Each element was defined as follows for def2-TZVPPD:

```
N     0
S    6   1.00
  19730.8006470             0.21887984991D-03
   2957.8958745             0.16960708803D-02
    673.22133595            0.87954603538D-02
    190.68249494            0.35359382605D-01
     62.295441898           0.11095789217
```

```
     22.654161182             0.24982972552
S    2   1.00
      8.9791477428            0.40623896148
      3.6863002370            0.24338217176
S    1   1.00
      0.84660076805           1.0000000
S    1   1.00
      0.33647133771           1.0000000
S    1   1.00
      0.13647653675           1.0000000
S    1   1.00
      0.68441605847D-01       1.0000000
P    4   1.00
     49.200380510             0.55552416751D-02
     11.346790537             0.38052379723D-01
      3.4273972411            0.14953671029
      1.1785525134            0.34949305230
P    1   1.00
      0.41642204972            .45843153697
P    1   1.00
      0.14260826011            .24428771672
D    1   1.00
      1.65400000              1.0000000
D    1   1.00
      0.46900000              1.0000000
D    1   1.00
      0.12829642058           1.0000000
F    1   1.00
      1.09300000              1.0000000
****
O     0
S    6   1.00
  27032.3826310               0.21726302465D-03
   4052.3871392               0.16838662199D-02
    922.32722710              0.87395616265D-02
    261.24070989              0.35239968808D-01
     85.354641351             0.11153519115
     31.035035245             0.25588953961
S    2   1.00
     12.260860728             0.39768730901
      4.9987076005            0.24627849430
S    1   1.00
      1.1703108158            1.0000000
S    1   1.00
      0.46474740994           1.0000000
S    1   1.00
      0.18504536357           1.0000000
S    1   1.00
      0.70288026270D-01       1.0000000
P    4   1.00
     63.274954801             0.60685103418D-02
     14.627049379             0.41912575824D-01
      4.4501223456            0.16153841088
      1.5275799647            0.35706951311
P    1   1.00
      0.52935117943            .44794207502
P    1   1.00
      0.17478421270            .24446069663
P    1   1.00
      0.51112745706D-01       1.0000000
D    1   1.00
      2.31400000              1.0000000
```

```
D    1   1.00
      0.64500000             1.0000000
D    1   1.00
      0.14696477366          1.0000000
F    1   1.00
      1.42800000             1.0000000
****
F     0
S    6   1.00
  35479.1004410              0.21545014888D-03
   5318.4728983              0.16700686527D-02
   1210.4810975              0.86733211476D-02
    342.85518140             0.35049933175D-01
    112.01943181             0.11165320133
     40.714740248            0.25988506647
S    2   1.00
     16.039678111            0.39422966880
      6.5038186740           0.24998238551
S    1   1.00
      1.5440477509           1.0000000
S    1   1.00
      0.61223452862          1.0000000
S    1   1.00
      0.24027979698          1.0000000
S    1   1.00
      0.90918446478D-01      1.0000000
P    4   1.00
     80.233900483            0.63685999134D-02
     18.594010743            0.44303143530D-01
      5.6867902653           0.16867248708
      1.9511006294           0.36166346255
P    1   1.00
      0.66970211298           .44202901491
P    1   1.00
      0.21651300410           .24319875730
P    1   1.00
      0.59613282472D-01      1.0000000
D    1   1.00
      3.10700000             1.0000000
D    1   1.00
      0.85500000             1.0000000
D    1   1.00
      0.18608388111          1.0000000
F    1   1.00
      1.91700000             1.0000000
****
Si     0
S    7   1.00
  44773.3580780              0.55914765868D-03
   6717.1992104              0.43206040189D-02
   1528.8960325              0.22187096460D-01
    432.54746585             0.86489249116D-01
    140.61505226             0.24939889716
     49.857636724            0.46017197366
     18.434974885            0.34250236575
S    3   1.00
     86.533886111            0.21300063007D-01
     26.624606846            0.94676139318D-01
      4.4953057159          -0.32616264859
S    2   1.00
      2.1035045710           1.3980803850
      1.0106094922           0.63865786699
```

```
S    1   1.00
      0.23701751489          1.0000000
S    1   1.00
      0.85703405362D-01      1.0000000
S    1   1.00
      0.37000771280D-01      1.0000000
P    5   1.00
    394.47503628             0.26285693959D-02
     93.137683104            0.20556257749D-01
     29.519608742            0.92070262801D-01
     10.781663791            0.25565889739
      4.1626574778           0.42111707185
P    1   1.00
      1.6247972989            .34401746318
P    1   1.00
      0.54306660493          1.0000000
P    1   1.00
      0.20582073956          1.0000000
P    1   1.00
      0.70053487306D-01      1.0000000
D    1   1.00
      2.30300000             1.0000000
D    1   1.00
      0.476000000            1.0000000
D    1   1.00
      0.160000000            1.0000000
D    1   1.00
      0.55036090333D-01      1.0000000
F    1   1.00
      0.336000000            1.0000000
****
K     0
S    8   1.00
 153976.1832500              0.23662636107D-03
  23082.4976720              0.18342929137D-02
   5253.2344745              0.95310527769D-02
   1486.9550133              0.38638406980D-01
    484.06333726             0.12480768502
    173.56653980             0.29278861009
     67.116381464            0.40633425860
     26.339502054            0.20077215860
S    4   1.00
    172.87693567            -0.24200960936D-01
     53.058649063           -0.11553095040
      7.9212753964           0.57455545175
      3.2108880472           0.57023185107
S    2   1.00
      4.5662070895          -0.22615763466
      0.70209907282          0.75528392045
S    1   1.00
      0.28258942635          1.0000000
S    1   1.00
      0.35805824617D-01      1.0000000
S    1   1.00
      0.15819213245D-01      1.0000000
P    6   1.00
    728.18449873             0.26150689792D-02
    172.13265061             0.20673630835D-01
     54.829847075            0.93205603870D-01
     20.166266494            0.25436518210
      7.8610728806           0.39131132810
      3.1105213132           0.22481345943
```

```
P    3   1.00
     11.757337492           -0.25777289217D-01
      1.5139617411           0.57359428604
      0.58328591795          1.0798320002
P    1   1.00
      0.21570478076          1.0000000
P    1   1.00
      0.0417370              1.0000000
P    1   1.00
      0.15208000000D-01      1.0000000
D    1   1.00
      0.9300000              1.0000000
D    1   1.00
      0.1800000              1.0000000
D    1   1.00
      0.0540000              1.0000000
****
```

And as follows for def2-QZVPPD:

```
H     0
S    4   1.00
    190.6916900              0.70815167D-03
     28.6055320              0.54678827D-02
      6.5095943              0.27966605D-01
      1.8412455              0.10764538
S    1   1.00
      0.59853725             1.0000000
S    1   1.00
      0.21397624             1.0000000
S    1   1.00
      0.80316286D-01         1.0000000
P    1   1.00
      2.29200000             1.0000000
P    1   1.00
      0.83800000             1.0000000
P    1   1.00
      0.29200000             1.0000000
P    1   1.00
      0.84063199228D-01      1.0000000
D    1   1.00
      2.06200000             1.0000000
D    1   1.00
      0.66200000             1.0000000
F    1   1.00
      1.39700000             1.0000000
****
C     0
S    8   1.00
  67025.0710290              0.38736308501D-04
  10039.9865380              0.30107917575D-03
   2284.9316911              0.15787918095D-02
    647.14122130             0.66087087195D-02
    211.09472335             0.23367123250D-01
     76.177643862            0.70420716898D-01
     29.633839163            0.17360344953
     12.187785081            0.32292305648
S    2   1.00
     53.026006299            0.74897404492D-01
     15.258502776            0.76136220983
S    1   1.00
      5.2403957464           1.0000000
```

```
S    1   1.00
      2.2905022379          1.0000000
S    1   1.00
      0.69673283006         1.0000000
S    1   1.00
      0.27599337363         1.0000000
S    1   1.00
      0.10739884389         1.0000000
S    1   1.00
      0.44981404899D-01     1.0000000
P    5   1.00
    105.12555082            0.84647553844D-03
     24.884461066           0.66274038534D-02
      7.8637230826          0.30120390419D-01
      2.8407001835          0.99951435476D-01
      1.1227137335          0.23826299282
P    1   1.00
      0.46050725555         1.0000000
P    1   1.00
      0.18937530913         1.0000000
P    1   1.00
      0.75983791611D-01     1.0000000
D    1   1.00
      1.84800000            1.0000000
D    1   1.00
      0.64900000            1.0000000
D    1   1.00
      0.22800000            1.0000000
D    1   1.00
      0.76889830417D-01     1.0000000
F    1   1.00
      1.41900000            1.0000000
F    1   1.00
      0.48500000            1.0000000
G    1   1.00
      1.01100000            1.0000000
****
N     0
S    8   1.00
  90726.8892100             0.39257887368D-04
  13590.5288010             0.30513316455D-03
   3092.9883781             0.16000560446D-02
    875.99876362            0.66982937306D-02
    285.74469982            0.23690078765D-01
    103.11913417            0.71455405268D-01
     40.128556777           0.17632774876
     16.528095704           0.32677592815
S    2   1.00
     69.390960983           0.80052094386D-01
     20.428200596           0.78268063538
S    1   1.00
      7.1292587972          1.0000000
S    1   1.00
      3.1324304893          1.0000000
S    1   1.00
      0.98755778723         1.0000000
S    1   1.00
      0.38765721307         1.0000000
S    1   1.00
      0.14909883075         1.0000000
S    1   1.00
      0.62151630318D-01     1.0000000
```

```
P    5   1.00
    150.05742670            -0.86216165986D-03
     35.491599483           -0.68571273236D-02
     11.247864223           -0.31795688855D-01
      4.0900305195          -0.10537396822
      1.6220573146          -0.24519708041
P    1   1.00
      0.66442261530          1.0000000
P    1   1.00
      0.27099770070          1.0000000
P    1   1.00
      0.10688749984          1.0000000
D    1   1.00
      2.83700000             1.0000000
D    1   1.00
      0.96800000             1.0000000
D    1   1.00
      0.33500000             1.0000000
D    1   1.00
      0.10825280010          1.0000000
F    1   1.00
      2.02700000             1.0000000
F    1   1.00
      0.68500000             1.0000000
G    1   1.00
      1.42700000             1.0000000
****
O     0
S    8   1.00
 116506.4690800              0.40383857939D-04
  17504.3497240              0.31255139004D-03
   3993.4513230              0.16341473495D-02
   1133.0063186              0.68283224757D-02
    369.99569594             0.24124410221D-01
    133.62074349             0.72730206154D-01
     52.035643649            0.17934429892
     21.461939313            0.33059588895
S    2   1.00
     89.835051252            0.96468652996D-01
     26.428010844            0.94117481120
S    1   1.00
      9.2822824649           1.0000000
S    1   1.00
      4.0947728533           1.0000000
S    1   1.00
      1.3255349078           1.0000000
S    1   1.00
      0.51877230787          1.0000000
S    1   1.00
      0.19772676454          1.0000000
S    1   1.00
      0.69638535104D-01      1.0000000
P    5   1.00
    191.15255810             0.25115697705D-02
     45.233356739            0.20039240864D-01
     14.353465922            0.93609064762D-01
      5.2422371832           0.30618127124
      2.0792418599           0.67810501439
P    1   1.00
      0.84282371424          1.0000000
P    1   1.00
      0.33617694891          1.0000000
```

```
P    1   1.00
      0.12863997974          1.0000000
P    1   1.00
      0.43598162776D-01      1.0000000
D    1   1.00
      3.77500000             1.0000000
D    1   1.00
      1.30000000             1.0000000
D    1   1.00
      0.44400000             1.0000000
D    1   1.00
      0.12546378695          1.0000000
F    1   1.00
      2.66600000             1.0000000
F    1   1.00
      0.85900000             1.0000000
G    1   1.00
      1.84600000             1.0000000
****
F     0
S    8   1.00
 132535.9734500              0.47387482743D-04
  19758.1125880              0.37070120897D-03
   4485.1996947              0.19450784713D-02
   1273.8151020              0.80573291994D-02
    418.93831236             0.27992880781D-01
    152.55721985             0.82735120175D-01
     59.821524823            0.19854169012
     24.819076932            0.34860632233
S    2   1.00
    100.74446673             0.10505068816
     30.103728290            0.94068472434
S    1   1.00
     10.814283272            1.0000000
S    1   1.00
      4.8172886770           1.0000000
S    1   1.00
      1.6559334213           1.0000000
S    1   1.00
      0.64893519582          1.0000000
S    1   1.00
      0.24778104545          1.0000000
S    1   1.00
      0.87626236800D-01      1.0000000
P    5   1.00
    240.96654114             0.30389933451D-02
     57.020699781            0.24357738582D-01
     18.126952120            0.11442925768
      6.6457404621           0.37064659853
      2.6375722892           0.79791551766
P    1   1.00
      1.0638217200           1.0000000
P    1   1.00
      0.41932562750          1.0000000
P    1   1.00
      0.15747588299          1.0000000
P    1   1.00
      0.46772400332D-01      1.0000000
D    1   1.00
      5.01400000             1.0000000
D    1   1.00
      1.72500000             1.0000000
```

```
D    1   1.00
      0.58600000              1.0000000
D    1   1.00
      0.15967986245           1.0000000
F    1   1.00
      3.56200000              1.0000000
F    1   1.00
      1.14800000              1.0000000
G    1   1.00
      2.37600000              1.0000000
****
Si     0
S    10   1.00
 918070.6956500              0.12658787192D-04
 137485.2538600              0.98408387461D-04
  31287.7727140              0.51731398244D-03
   8861.6105697              0.21785441933D-02
   2890.6943156              0.78656853716D-02
   1043.4063979              0.24987553674D-01
    406.80160276             0.69761818844D-01
    168.48360207             0.16473362733
     73.185628823            0.30285602647
     32.998485420            0.36007260439
S    3   1.00
    278.78751325             0.20185463814D-01
     85.910228722            0.17720406747
     32.992604031            0.63587860906
S    1   1.00
     15.033693254            1.0000000
S    1   1.00
      5.7257514773           1.0000000
S    1   1.00
      2.6146521029           1.0000000
S    1   1.00
      1.1757833152           1.0000000
S    1   1.00
      0.35432330868          1.0000000
S    1   1.00
      0.16248335806          1.0000000
S    1   1.00
      0.68332457181D-01      1.0000000
S    1   1.00
      0.30521078871D-01      1.0000000
P    8   1.00
   1775.8850516              0.20186971126D-03
    420.83786849             0.17544314655D-02
    136.42290243             0.95039532290D-02
     51.700991737            0.37325741590D-01
     21.559456002            0.11085396329
      9.5559200095           0.23756407046
      4.3529819473           0.35295783750
      2.0096964381           0.32885931947
P    2   1.00
     46.418153780            0.25858819154D-01
      1.9076417796          -0.59874865899
P    1   1.00
      0.92337221587          1.0000000
P    1   1.00
      0.34515720370          1.0000000
P    1   1.00
      0.13656834761          1.0000000
P    1   1.00
```

```
      0.52987060586D-01      1.0000000
D    1   1.00
      2.64500000             1.0000000
D    1   1.00
      0.608000000            1.0000000
D    1   1.00
      0.272000000            1.0000000
D    1   1.00
      0.113000000            1.0000000
D    1   1.00
      0.44018904228D-01      1.0000000
F    1   1.00
      0.541000000            1.0000000
F    1   1.00
      0.212000000            1.0000000
G    1   1.00
      0.461000000            1.0000000
****
K     0
S    11   1.00
2022075.1391000             0.10144874011D-04
 303044.6556800             0.78783500963D-04
  69013.9384900             0.41380919674D-03
  19559.2446150             0.17427571893D-02
   6383.8934901             0.63004086126D-02
   2305.3502858             0.20125347032D-01
    899.14418101            0.56966060520D-01
    372.59500133            0.13882654093
    161.97635247            0.27132285414
     73.085553853           0.36383620662
     33.595644762           0.24724464776
S    4   1.00
    685.49855365            0.44367463797D-02
    211.66163373            0.42502718169D-01
     80.851405131           0.18467742018
     35.108667236           0.29353173891
S    1   1.00
     14.409437283           1.0000000
S    1   1.00
      6.8697147262          1.0000000
S    1   1.00
      3.2121883687          1.0000000
S    1   1.00
      1.2610484365          1.0000000
S    1   1.00
      0.61438946301         1.0000000
S    1   1.00
      0.27264370973         1.0000000
S    1   1.00
      0.77173005207D-01     1.0000000
S    1   1.00
      0.40918397569D-01     1.0000000
S    1   1.00
      0.17055762425D-01     1.0000000
P    9   1.00
   3469.6649718             0.21340225970D-03
    822.02356107            0.18637844836D-02
    266.58406636            0.10211431546D-01
    101.28048161            0.40799604728D-01
     42.429490761           0.12257473393
     18.912083912           0.26405833679
      8.7017779030          0.38227164575
```

```
      4.0854567599           0.29998812383
      1.8667286060           0.78007265591D-01
P    5   1.00
     27.544639057           -0.58222994163D-02
      9.2098893609          -0.26787531329D-01
      1.7232877425           0.30666847665
      0.77969303735          0.66927944165
      0.34379047862          0.60376468615
P    1   1.00
      0.14346681918          1.0000000
P    1   1.00
      0.68000000000D-01      1.0000000
P    1   1.00
      0.32000000000D-01      1.0000000
P    1   1.00
      0.15300000000D-01      1.0000000
D    1   1.00
      1.7000000              1.0000000
D    1   1.00
      0.5100000              1.0000000
D    1   1.00
      0.1800000              1.0000000
D    1   1.00
      0.0540000              1.0000000
F    1   1.00
      2.3700000              1.0000000
F    1   1.00
      0.7900000              1.0000000
F    1   1.00
      0.0900000              1.0000000
****
```

Frequency analyses for all stationary points were performed using BS1 to confirm the nature of the structures as either minima (no imaginary frequencies) or transition states (precisely one imaginary frequency). Intrinsic reaction coordinate (IRC) calculations followed by full geometry optimisations on final points were used to connect transition states and minima located on the potential energy surface.

Thermal corrections were applied using Paton's *GoodVibes* software,[55] which incorporates Grimme's quasi-harmonic approximation to the vibrational entropy below a cut-off of 100 cm^{-1} and a frequency scaling factor = 1.0. The entropic terms for the frequencies below the cut-off are obtained from the free-rotor approximation while the standard rigid-rotor harmonic oscillator (RRHO) approximation is retained for those above the cut-off. A dampening function is used to interpolate between these two expressions close to the cutoff frequency. To account for experimental conditions, a temperature of 298.15 K and concentrations of 0.083 mol L^{-1} (fluorinated gases and species along monomeric pathway) and 0.0415 mol L^{-1} (species along dimeric pathway) were also applied. Natural bond order (NBO) analysis was performed with BS1 using NBO version 7.0.[56,57,58]

Functional screening.

A functional screening was undertaken across the entire reaction sequence (both monomeric and dimeric pathways) for each of the following functionals: B3PW91-D3(BJ), PBE0-D3(BJ), ωB97X-D(2), B3LYP-D3(BJ), M06-2X-D3, M06-L-D3 and BP86-D3(BJ) at the BS1 level of theory. The calculated energies for these are found in Tables S19-25 and an overview and comparison in Supplementary Table 26.

Supplementary Table 19: Calculated energies for reaction sequence and alternative mechanisms using B3PW91-D3(BJ) (BS1).

B3PW91-D3(BJ)	SCF (BS1, hartrees)	H corr. (BS1, hartrees)	H (BS1, hartrees)	G corr. (BS1, hartrees)	G (BS1, hartrees)	G (SCF_{BS1} + $corr_{BS1}$, kcal mol^{-1})
**KHMDS (monomer)**	-1473.292432	0.244598	-1473.047835	0.178365	-1473.114068	-924392.34
**KHMDS (dimer)**	-2946.640924	0.492484	-2946.148439	0.386634	-2946.254290	-1848801.08
**1,1,1-trifluoroethane**	-377.603894	0.057679	-377.546214	0.024155	-377.579739	-236934.68
**encounter complex (monomer)**	-1850.907885	0.304279	-1850.603605	0.221462	-1850.686423	-1161322.39
**encounter complex (dimer_intact)**	-3324.249758	0.552653	-3323.697105	0.429563	-3323.820194	-2085727.09
**encounter complex (dimer_parsep)**	-3324.239810	0.551988	-3323.687822	0.427693	-3323.812117	-2085722.02
**encounter complex (dimer_sep)**	-3324.213928	0.551256	-3323.662672	0.424064	-3323.789864	-2085708.05
**TS-H_monomer**	-1850.880535	0.297581	-1850.582954	0.218262	-1850.662273	-1161307.23
**TS-H_dimer_intact**	-3324.218019	0.546035	-3323.671985	0.426753	-3323.791266	-2085708.93
**TS-H_dimer_parsep**	-3324.215464	0.545171	-3323.670293	0.422238	-3323.793226	-2085710.16
**TS-H_dimer_sep**	-3324.192885	0.545001	-3323.647884	0.420619	-3323.772266	-2085697.01
**HMDS-H**	-873.983391	0.254553	-873.728838	0.193939	-873.789453	-548310.75
**K-CH_2CF_3**	-976.882210	0.045282	-976.836928	0.004096	-976.878114	-612999.81
**K-CH_2CF_3-KHMDS complex**	-2450.214093	0.292350	-2449.921743	0.205162	-2450.008931	-1537402.65
**TS-βF**	-976.881073	0.043959	-976.837114	0.004561	-976.876512	-612998.80
**TS-βF_khmds**	-2450.214470	0.290201	-2449.924269	0.207876	-2450.006593	-1537401.19
**1,1-difluoroethene**	-277.090023	0.041098	-277.048924	0.010376	-277.079646	-173869.97
**KF**	-699.813682	0.004410	-699.809272	-0.021687	-699.835369	-439152.99
**KF-KHMDS complex**	-2173.151104	0.250943	-2172.900160	0.176691	-2172.974412	-1363561.00

Supplementary Table 20: Calculated energies for reaction sequence and alternative mechanisms using PBE0-D3(BJ) (BS1).

PBE0-D3(BJ)	SCF (BS1, hartrees)	H corr. (BS1, hartrees)	H (BS1, hartrees)	G corr. (BS1, hartrees)	G (BS1, hartrees)	G (SCF_{BS1} + $corr_{BS1}$, kcal mol^{-1})
**KHMDS (monomer)**	-1472.687311	0.244896	-1472.442415	0.178552	-1472.508759	-924012.50
**KHMDS (dimer)**	-2945.424246	0.492929	-2944.931317	0.386040	-2945.038206	-1848037.98
**1,1,1-trifluoroethane**	-377.365022	0.057937	-377.307085	0.024454	-377.340568	-236784.60
**encounter complex (monomer)**	-1850.063407	0.304806	-1849.758601	0.221538	-1849.841869	-1160792.42
**encounter complex (dimer_intact)**	-3322.794175	0.553400	-3322.240775	0.430867	-3322.363308	-2084812.88
**encounter complex (dimer_parsep)**	-3322.786160	0.552847	-3322.233313	0.431307	-3322.354854	-2084807.57
**encounter complex (dimer_sep)**	-3322.763620	0.552012	-3322.211608	0.424088	-3322.339531	-2084797.96
**TS-H_monomer**	-1850.034327	0.298407	-1849.735920	0.218165	-1849.816162	-1160776.29
**TS-H_dimer_intact**	-3322.760879	0.546734	-3322.214146	0.427744	-3322.333135	-2084793.94
**TS-H_dimer_parsep**	-3322.759984	0.545896	-3322.214088	0.422831	-3322.337153	-2084796.46
**TS-H_dimer_sep**	-3322.740037	0.545659	-3322.194378	0.418640	-3322.321398	-2084786.58
**HMDS-H**	-873.505955	0.254877	-873.251078	0.194048	-873.311907	-548011.08
**K-CH_2CF_3**	-976.516976	0.045592	-976.471384	0.004993	-976.511982	-612770.06
**K-CH_2CF_3-KHMDS complex**	-2449.241356	0.292955	-2448.948400	0.205538	-2449.035818	-1536792.02
**TS-βF**	-976.515574	0.044129	-976.471445	0.004227	-976.511347	-612769.66
**TS-βF_khmds**	-2449.241022	0.291621	-2448.949401	0.207036	-2449.033985	-1536790.87
**1,1-difluoroethene**	-276.908765	0.041290	-276.867475	0.010592	-276.898173	-173756.10
**KF**	-699.625798	0.004416	-699.621382	-0.021656	-699.647454	-439035.07
**KF-KHMDS complex**	-2172.356637	0.251216	-2172.105421	0.176188	-2172.180448	-1363062.78

Supplementary Table 21: Calculated energies for reaction sequence and alternative mechanisms using ωB97X-D(2) (BS1).

ωB97X-D(2)	SCF (BS1, hartrees)	H corr. (BS1, hartrees)	H (BS1, hartrees)	G corr. (BS1, hartrees)	G (BS1, hartrees)	G (SCF_{BS1} + $corr_{BS1}$, kcal mol^{-1})
**KHMDS (monomer)**	-1473.364265	0.245803	-1473.118462	0.180081	-1473.184184	-924436.33
**KHMDS (dimer)**	-2946.776903	0.495378	-2946.281525	0.390325	-2946.386578	-1848884.10
**1,1,1-trifluoroethane**	-377.632468	0.058086	-377.574382	0.024641	-377.607827	-236952.31
**encounter complex (monomer)**	-1851.008181	0.306120	-1850.702062	0.225303	-1850.782878	-1161382.91
**encounter complex (dimer_intact)**	-3324.414784	0.555228	-3323.859556	0.432665	-3323.982119	-2085828.70
**encounter complex (dimer_parsep)**	-3324.401924	0.554664	-3323.847261	0.431385	-3323.970539	-2085821.43
**encounter complex (dimer_sep)**	-3324.385977	0.554054	-3323.831923	0.427597	-3323.958380	-2085813.80
**TS-H_monomer**	-1850.975342	0.299503	-1850.675839	0.220544	-1850.754799	-1161365.29
**TS-H_dimer_intact**	-3324.375600	0.549080	-3323.826520	0.430185	-3323.945415	-2085805.66
**TS-H_dimer_parsep**	-3324.376287	0.547468	-3323.828819	0.428803	-3323.947484	-2085806.96
**TS-H_dimer_sep**	-3324.358909	0.548318	-3323.810591	0.426972	-3323.931937	-2085797.21
**HMDS-H**	-874.011087	0.255917	-873.755170	0.196198	-873.814889	-548326.71
**K-CH_2CF_3**	-976.956059	0.045642	-976.910416	0.005108	-976.950951	-613045.51
**K-CH_2CF_3-KHMDS complex**	-2450.354933	0.293918	-2450.061016	0.208212	-2450.146721	-1537489.12
**TS-βF**	-976.954313	0.044103	-976.910210	0.003816	-976.950497	-613045.23
**TS-βF_khmds**	-2450.355216	0.292845	-2450.062371	0.208906	-2450.146310	-1537488.86
**1,1-difluoroethene**	-277.108117	0.041397	-277.066720	0.010723	-277.097395	-173881.11
**KF**	-699.870967	0.004404	-699.866562	-0.021714	-699.892680	-439188.96
**KF-KHMDS complex**	-2173.276563	0.252738	-2173.023825	0.179240	-2173.097323	-1363638.13

Supplementary Table 22: Calculated energies for reaction sequence and alternative mechanisms using B3LYP-D3(BJ) (BS1).

B3LYP-D3(BJ)	SCF (BS1, hartrees)	H corr. (BS1, hartrees)	H (BS1, hartrees)	G corr. (BS1, hartrees)	G (BS1, hartrees)	G (SCF_{BS1} + $corr_{BS1}$, kcal mol^{-1})
**KHMDS (monomer)**	-1473.561448	0.244546	-1473.316901	0.178338	-1473.383110	-924561.16
**KHMDS (dimer)**	-2947.179899	0.492545	-2946.687354	0.386628	-2946.793271	-1849139.30
**1,1,1-trifluoroethane**	-377.746975	0.057512	-377.689463	0.023949	-377.723026	-237024.60
**encounter complex (monomer)**	-1851.320940	0.304161	-1851.016779	0.221490	-1851.099450	-1161581.56
**encounter complex (dimer_intact)**	-3324.932477	0.552848	-3324.379629	0.431057	-3324.501420	-2086154.56
**encounter complex (dimer_parsep)**	-3324.918372	0.551744	-3324.366628	0.429707	-3324.488664	-2086146.56
**encounter complex (dimer_sep)**	-3324.896966	0.551257	-3324.345709	0.423766	-3324.473200	-2086136.85
**TS-H_monomer**	-1851.291321	0.297404	-1850.993916	0.218353	-1851.072968	-1161564.95
**TS-H_dimer_intact**	-3324.897020	0.546027	-3324.350993	0.427413	-3324.469607	-2086134.60
**TS-H_dimer_parsep**	-3324.896843	0.545090	-3324.351753	0.423077	-3324.473766	-2086137.21

**TS-H_dimer_sep**	-3324.872894	0.544978	-3324.327915	0.420736	-3324.452158	-2086123.65
**HMDS-H**	-874.195593	0.254541	-873.941052	0.194007	-874.001586	-548443.86
**K-CH_2CF_3**	-977.083590	0.045026	-977.038564	0.004158	-977.079432	-613126.14
**K-CH_2CF_3-KHMDS complex**	-2450.686963	0.292068	-2450.394895	0.205191	-2450.481772	-1537699.37
**TS-βF**	-977.083056	0.043834	-977.039222	0.004540	-977.078516	-613125.56
**TS-βF_khmds**	-2450.687128	0.290851	-2450.396277	0.204143	-2450.482985	-1537700.13
**1,1-difluoroethene**	-277.198235	0.040981	-277.157254	0.010238	-277.187997	-173937.96
**KF**	-699.911245	0.004413	-699.906832	-0.021680	-699.932925	-439214.21
**KF-KHMDS complex**	-2173.519970	0.250900	-2173.269070	0.176173	-2173.343797	-1363792.79

Supplementary Table 23: Calculated energies for reaction sequence and alternative mechanisms using M06-2X-D3 (BS1).

M06-2X-D3	SCF (BS1, hartrees)	H corr. (BS1, hartrees)	H (BS1, hartrees)	G corr. (BS1, hartrees)	G (BS1, hartrees)	G (SCF_{BS1} + $corr_{BS1}$, kcal mol^{-1})
**KHMDS (monomer)**	-1473.251739	0.245773	-1473.005965	0.180046	-1473.071693	-924365.75
**KHMDS (dimer)**	-2946.551214	0.494297	-2946.056917	0.388786	-2946.162428	-1848743.44
**1,1,1-trifluoroethane**	-377.601334	0.058388	-377.542945	0.024968	-377.576366	-236932.57
**encounter complex (monomer)**	-1850.865690	0.306065	-1850.559626	0.226092	-1850.639598	-1161293.00
**encounter complex (dimer_intact)**	-3324.156230	0.555644	-3323.600586	0.434117	-3323.722113	-2085665.54
**encounter complex (dimer_parsep)**	-3324.148633	0.554410	-3323.594223	0.433405	-3323.715228	-2085661.22
**encounter complex (dimer_sep)**	-3324.129371	0.554077	-3323.575294	0.431043	-3323.698328	-2085650.61
**TS-H_monomer**	-1850.832660	0.299489	-1850.533171	0.220734	-1850.611926	-1161275.64
**TS-H_dimer_intact**	-3324.120772	0.548463	-3323.572310	0.430632	-3323.690140	-2085645.48
**TS-H_dimer_parsep**	-3324.122040	0.547734	-3323.574306	0.426987	-3323.695053	-2085648.56
**TS-H_dimer_sep**	-3324.102493	0.547108	-3323.555385	0.423474	-3323.679018	-2085638.50
**HMDS-H**	-873.903636	0.255524	-873.648112	0.195129	-873.708506	-548259.95
**K-CH_2CF_3**	-976.920865	0.046022	-976.874843	0.006331	-976.914535	-613022.66
**K-CH_2CF_3-KHMDS complex**	-2450.210140	0.293935	-2449.916205	0.208983	-2450.001157	-1537397.78
**TS-βF**	-976.918487	0.044470	-976.874017	0.004926	-976.913560	-613022.05
**TS-βF_khmds**	-2450.210372	0.292587	-2449.917785	0.208265	-2450.002107	-1537398.37
**1,1-difluoroethene**	-277.087299	0.041562	-277.045736	0.010884	-277.076415	-173867.94
**KF**	-699.849957	0.004432	-699.845525	-0.021600	-699.871557	-439175.70
**KF-KHMDS complex**	-2173.146290	0.251908	-2172.894382	0.175933	-2172.970357	-1363558.46

Supplementary Table 24: Calculated energies for reaction sequence and alternative mechanisms using M06-L-D3 (BS1).

M06-L-D3	SCF (BS1, hartrees)	H corr. (BS1, hartrees)	H (BS1, hartrees)	G corr. (BS1, hartrees)	G (BS1, hartrees)	G (SCF_{BS1} + $corr_{BS1}$, kcal mol^{-1})
**KHMDS (monomer)**	-1473.391573	0.246804	-1473.144769	0.181656	-1473.209916	-924452.48
**KHMDS (dimer)**	-2946.828675	0.495971	-2946.332704	0.388819	-2946.439856	-1848917.53
**1,1,1-trifluoroethane**	-377.674052	0.057839	-377.616214	0.024305	-377.649748	-236978.62
**encounter complex (monomer)**	-1851.078887	0.306763	-1850.772124	0.227849	-1850.851038	-1161425.68
**encounter complex (dimer_intact)**	-3324.508726	0.558043	-3323.950683	0.439398	-3324.069328	-2085883.42
**encounter complex (dimer_parsep)**	-3324.501094	0.555734	-3323.945360	0.434180	-3324.066914	-2085881.91
**encounter complex (dimer_sep)**	-3324.484839	0.555404	-3323.929435	0.432001	-3324.052838	-2085873.07
**TS-H_monomer**	-1851.042873	0.299819	-1850.743054	0.221548	-1850.821324	-1161407.04
**TS-H_dimer_intact**	-3324.468489	0.549466	-3323.919023	0.431873	-3324.036616	-2085862.89
**TS-H_dimer_parsep**	-3324.471352	0.548941	-3323.922412	0.429329	-3324.042023	-2085866.29

**TS-H_dimer_sep**	-3324.455615	0.548478	-3323.907137	0.427044	-3324.028571	-2085857.84
**HMDS-H**	-874.043575	0.256446	-873.787129	0.195439	-873.848136	-548347.57
**K-CH_2CF_3**	-976.990543	0.045340	-976.945203	0.005412	-976.985131	-613066.96
**K-CH_2CF_3-KHMDS complex**	-2450.419020	0.294278	-2450.124742	0.211096	-2450.207923	-1537527.52
**TS-βF**	-976.988844	0.044047	-976.944797	0.004498	-976.984346	-613066.47
**TS-βF_khmds**	-2450.418895	0.293373	-2450.125522	0.210615	-2450.208280	-1537527.75
**1,1-difluoroethene**	-277.144501	0.041095	-277.103405	0.010367	-277.134133	-173904.16
**KF**	-699.868623	0.004426	-699.864197	-0.021630	-699.890253	-439187.43
**KF-KHMDS complex**	-2173.301707	0.252137	-2173.049570	0.181316	-2173.120391	-1363652.60

Supplementary Table 25: Calculated energies for reaction sequence and alternative mechanisms using BP86-D3(BJ) (BS1).

BP86-D3(BJ)	SCF (BS1, hartrees)	H corr. (BS1, hartrees)	H (BS1, hartrees)	G corr. (BS1, hartrees)	G (BS1, hartrees)	G (SCF_{BS1} + $corr_{BS1}$, kcal mol^{-1})
**KHMDS (monomer)**	-1473.586722	0.238750	-1473.347971	0.171719	-1473.415003	-924581.18
**KHMDS (dimer)**	-2947.232300	0.480694	-2946.751606	0.373375	-2946.858924	-1849180.50
**1,1,1-trifluoroethane**	-377.739397	0.055951	-377.683446	0.022173	-377.717224	-237020.96
**encounter complex (monomer)**	-1851.338310	0.296582	-1851.041728	0.213048	-1851.125262	-1161597.76
**encounter complex (dimer_intact)**	-3324.976712	0.539609	-3324.437103	0.416560	-3324.560152	-2086191.42
**encounter complex (dimer_parsep)**	-3324.966737	0.538336	-3324.428402	0.413102	-3324.553635	-2086187.33
**encounter complex (dimer_sep)**	-3324.939885	0.537459	-3324.402426	0.407993	-3324.531892	-2086173.68
**TS-H_monomer**	-1851.316113	0.289997	-1851.026115	0.209042	-1851.107070	-1161586.35
**TS-H_dimer_intact**	-3324.949271	0.532281	-3324.416990	0.407324	-3324.541947	-2086179.99
**TS-H_dimer_parsep**	-3324.947254	0.531692	-3324.415561	0.407156	-3324.540098	-2086178.83
**TS-H_dimer_sep**						
**HMDS-H**	-874.179192	0.248457	-873.930736	0.187038	-873.992154	-548437.94
**K-CH_2CF_3**	-977.118263	0.043844	-977.074419	0.002340	-977.115923	-613149.04
**K-CH_2CF_3-KHMDS complex**	-2450.746358	0.284934	-2450.461424	0.196864	-2450.549494	-1537741.86
**TS-βF**	-977.118040	0.042760	-977.075279	0.003192	-977.114847	-613148.36
**TS-βF_khmds**	-2450.747337	0.284021	-2450.463316	0.197225	-2450.550112	-1537742.25
**1,1-difluoroethene**	-277.193454	0.039880	-277.153573	0.008995	-277.184458	-173935.74
**KF**	-699.946057	0.004406	-699.941650	-0.021703	-699.967760	-439236.07
**KF-KHMDS complex**	-2173.578826	0.245095	-2173.333731	0.169749	-2173.409077	-1363833.76

Supplementary Table 26: Comparison of reaction pathway across different functionals (BS1). Energies in kcal mol⁻¹.

	B3PW91-D3(BJ)	PBE0-D3(BJ)	ωB97X-D(2)	B3LYP-D3(BJ)	M06-2X-D3	M06-L-D3	BP86-D3(BJ)
				**Monomeric deprotonation**			
**dimer + trifluoroethane**	0.00	0.00	0.00	0.00	0.00	0.00	0.00
**2*monomer + trifluoroethane**	16.41	12.98	11.43	16.97	11.95	12.57	18.15
**encounter complex (monomer)**	21.05	17.66	17.16	21.17	17.26	17.98	22.52
**TS-H_monomer**	36.20	33.79	34.78	37.79	34.62	36.62	33.93
				**Dimeric deprotonation**			
**encounter complex (dimer_intact)**	8.68	9.71	7.71	9.34	10.47	12.72	10.04
**encounter complex (dimer_parsep)**	13.75	15.01	14.98	17.34	14.79	14.24	14.13
**encounter complex (dimer_sep)**	27.71	24.63	22.61	27.04	25.39	23.07	27.77
**TS-H_dimer_intact**	26.83	28.64	30.74	29.30	30.53	33.25	21.46
**TS-H_dimer_parsep**	25.60	26.12	29.44	26.69	27.45	29.86	22.62
**TS-H_dimer_sep**	38.76	36.00	39.20	40.25	37.51	38.30	
				**β-fluoride elimination without KHMDS complex**			
**HMDS-H + KCH_2CF_3 + $KHMDS_{monomer}$**	32.88	28.94	27.85	32.74	27.65	29.13	33.30
**TS-βF + HMDS-H + $KHMDS_{monomer}$**	33.88	29.34	28.13	33.31	28.26	29.62	33.98
**HMDS-H + $KHMDS_{monomer}$ + difluoroethene + KF**	9.72	7.83	3.30	6.70	6.67	4.50	10.53
				**β-fluoride elimination with KHMDS complex**			
**HMDS-H + KCH_2CF_3-KHMDS**	22.37	19.48	20.58	20.67	18.28	21.05	21.65
**TS-βF-KHMDS + HMDS-H**	23.83	20.63	20.84	19.91	17.68	20.83	21.26
**HMDS-H + difluoroethene + KF-KHMDS**	-5.95	-7.38	-9.54	-10.72	-10.34	-8.19	-5.99

Single point corrections (BS2) and vibrational corrections (*goodvibes*) were applied to the optimised structures that were calculated with the following functionals: B3PW91-D3(BJ), PBE0-D3(BJ) and ωB97X-D(2) for a more precise comparison between these hybrid functionals (Tables S27-29). An overview and comparison are provided in Supplementary Table 30.

Supplementary Table 27: Calculated energies for reaction sequence and alternative mechanisms using B3PW91-D3(BJ) (BS2 + *goodvibes* corrections).

B3PW91-D3(BJ)	G corr. (BS1, hartrees)	G (BS1, hartrees)	SCF (BS2, hartrees)	G corr. (*goodvibes*, hartrees)	G (SCF_{BS1} + $corr_{BS1}$, kcal mol^{-1})	G (SCF_{BS2} + $corr_{goodvibes}$, kcal mol^{-1})
**KHMDS (monomer)**	0.178365	-1473.114068	-1473.352054	0.182182	-924392.3357	-924427.3532
**KHMDS (dimer)**	0.386634	-2946.254290	-2946.759295	0.392718	-1848801.083	-1848871.544
**1,1,1-trifluoroethane**	0.024155	-377.579739	-377.623746	0.024818	-236934.6844	-236946.7257
**encounter complex (monomer)**	0.221462	-1850.686423	-1850.986872	0.227950	-1161322.387	-1161367.88
**encounter complex (dimer_intact)**	0.429563	-3323.820194	-3324.387466	0.438631	-2085727.086	-2085807.81
**encounter complex (dimer_parsep)**	0.427693	-3323.812117	-3324.377564	0.437528	-2085722.018	-2085802.288
**encounter complex (dimer_sep)**	0.424064	-3323.789864	-3324.352124	0.435168	-2085708.054	-2085787.805
**TS-H_monomer**	0.218262	-1850.662273	-1850.958987	0.223180	-1161307.232	-1161353.376
**TS-H_dimer_intact**	0.426753	-3323.791266	-3324.355573	0.434604	-2085708.934	-2085790.323
**TS-H_dimer_parsep**	0.422238	-3323.793226	-3324.352829	0.431490	-2085710.163	-2085790.556
**TS-H_dimer_sep**	0.420619	-3323.772266	-3324.330515	0.430503	-2085697.011	-2085777.173
**HMDS-H**	0.193939	-873.789453	-874.029793	0.195565	-548310.7459	-548338.8426
**K-CH_2CF_3**	0.004096	-976.878114	-976.915169	0.005995	-612999.8084	-613019.2989
**K-CH_2CF_3-KHMDS complex**	0.205162	-2450.008931	-2450.306087	0.211647	-1537402.654	-1537456.312
**TS-βF**	0.004561	-976.876512	-976.914073	0.005699	-612998.8032	-613018.7969
**TS-βF_khmds**	0.207876	-2450.006593	-2450.306296	0.212451	-1537401.187	-1537455.939
**1,1-difluoroethene**	0.010376	-277.079646	-277.104854	0.011044	-173869.9716	-173878.8596
**KF**	-0.021687	-699.835369	-699.832229	-0.021020	-439152.9926	-439164.2124
**KF-KHMDS complex**	0.176691	-2172.974412	-2173.228848	0.180437	-1363561	-1363607.435

Supplementary Table 28: Calculated energies for reaction sequence and alternative mechanisms using PBE0-D3(BJ) (BS2 + *goodvibes* corrections).

PBE0-D3(BJ)	G corr. (BS1, hartrees)	G (BS1, hartrees)	SCF (BS2, hartrees)	G corr. (*goodvibes*, hartrees)	G (SCF_{BS1} + $corr_{BS1}$, kcal mol^{-1})	G (SCF_{BS2} + $corr_{goodvibes}$, kcal mol^{-1})
**KHMDS (monomer)**	0.178552	-1472.508759	-1472.744135	0.182407	-924012.4989	-924045.7374
**KHMDS (dimer)**	0.386040	-2945.038206	-2945.536906	0.392573	-1848037.98	-1848104.575
**1,1,1-trifluoroethane**	0.024454	-377.340568	-377.383865	0.025117	-236784.6025	-236796.0106
**encounter complex (monomer)**	0.221538	-1849.841869	-1850.138582	0.228237	-1160792.421	-1160835.391
**encounter complex (dimer_intact)**	0.430867	-3322.363308	-3322.925180	0.439557	-2084812.877	-2084889.631
**encounter complex (dimer_parsep)**	0.431307	-3322.354854	-3322.917376	0.439888	-2084807.572	-2084884.526
**encounter complex (dimer_sep)**	0.424088	-3322.339531	-3322.895200	0.435455	-2084797.957	-2084873.392
**TS-H_monomer**	0.218165	-1849.816162	-1850.109104	0.223665	-1160776.29	-1160819.762
**TS-H_dimer_intact**	0.427744	-3322.333135	-3322.891774	0.435438	-2084793.943	-2084871.253
**TS-H_dimer_parsep**	0.422831	-3322.337153	-3322.890718	0.432135	-2084796.465	-2084872.663
**TS-H_dimer_sep**	0.418640	-3322.321398	-3322.871122	0.429840	-2084786.578	-2084861.806
**HMDS**	0.194048	-873.311907	-873.549918	0.195747	-548011.0814	-548037.6025
**K-CH_2CF_3**	0.004993	-976.511982	-976.548559	0.006617	-612770.0573	-612788.8575
**K-CH_2CF_3-KHMDS complex**	0.205538	-2449.035818	-2449.329182	0.212146	-1536792.017	-1536842.982
**TS-βF**	0.004227	-976.511347	-976.547144	0.005584	-612769.6588	-612788.6178
**TS-βF_khmds**	0.207036	-2449.033985	-2449.328625	0.212132	-1536790.867	-1536842.641
**1,1-difluoroethene**	0.010592	-276.898173	-276.922836	0.011260	-173756.0956	-173764.5061
**KF**	-0.021656	-699.647454	-699.643723	-0.020989	-439035.0742	-439045.9038
**KF-KHMDS complex**	0.176188	-2172.180448	-2172.430933	0.180328	-1363062.781	-1363106.805

Supplementary Table 29: Calculated energies for reaction sequence and alternative mechanisms using ωB97X-D(2) (BS2 + *goodvibes* corrections).

ωB97X-D(2)	G corr. (BS1, hartrees)	G (BS1, hartrees)	SCF (BS2, hartrees)	G corr. (*goodvibes*, hartrees)	G (SCF_{BS1} + $corr_{BS1}$, kcal mol^{-1})	G (SCF_{BS2} + $corr_{goodvibes}$, kcal mol^{-1})
**KHMDS (monomer)**	0.180081	-1473.184184	-1473.423327	0.183719	-924436.3341	-924471.1132
**KHMDS (dimer)**	0.390325	-2946.386578	-2946.893920	0.396636	-1848884.095	-1848953.564
**1,1,1-trifluoroethane**	0.024641	-377.607827	-377.652378	0.025304	-236952.3099	-236964.3876
**encounter complex (monomer)**	0.225303	-1850.782878	-1851.086520	0.231003	-1161382.913	-1161428.495
**encounter complex (dimer_intact)**	0.432665	-3323.982119	-3324.551032	0.441770	-2085828.696	-2085908.479
**encounter complex (dimer_parsep)**	0.431385	-3323.970539	-3324.538247	0.441104	-2085821.429	-2085900.874
**encounter complex (dimer_sep)**	0.427597	-3323.958380	-3324.522729	0.438416	-2085813.799	-2085892.823
**TS-H_monomer**	0.220544	-1850.754799	-1851.053186	0.225564	-1161365.293	-1161410.99
**TS-H_dimer_intact**	0.430185	-3323.945415	-3324.511690	0.438608	-2085805.663	-2085885.776
**TS-H_dimer_parsep**	0.428803	-3323.947484	-3324.512206	0.437155	-2085806.962	-2085887.011
**TS-H_dimer_sep**	0.426972	-3323.931937	-3324.494974	0.435851	-2085797.206	-2085877.016
**HMDS**	0.196198	-873.814889	-874.057676	0.197537	-548326.7072	-548355.102
**K-CH_2CF_3**	0.005108	-976.950951	-976.988315	0.006678	-613045.5143	-613064.7701
**K-CH_2CF_3-KHMDS complex**	0.208212	-2450.146721	-2450.445546	0.214137	-1537489.119	-1537542.261
**TS-βF**	0.003816	-976.950497	-976.986591	0.005305	-613045.2294	-613064.5498
**TS-βF_khmds**	0.208906	-2450.146310	-2450.445744	0.214009	-1537488.861	-1537542.466
**1,1-difluoroethene**	0.010723	-277.097395	-277.123076	0.011390	-173881.1092	-173890.077
**KF**	-0.021714	-699.892680	-699.888847	-0.021047	-439188.9557	-439199.7577
**KF-KHMDS complex**	0.179240	-2173.097323	-2173.353087	0.183229	-1363638.128	-1363683.644

Supplementary Table 30: Comparison of reaction pathway across different functionals (BS2 + *goodvibes* corrections). Lowest energy pathway highlighted in green. Energies in kcal mol^{-1}.

	B3PW91-D3(BJ)	PBE0-D3(BJ)	ωB97X-D(2)
**Monomeric deprotonation**			
**dimer + trifluoroethane**	**0.00**	0.00	0.00
**2*monomer + trifluoroethane**	16.84	13.10	11.34
**encounter complex (monomer)**	23.04	19.46	18.34
**TS-H_monomer**	37.54	35.09	35.85
**Dimeric deprotonation**			
**encounter complex (dimer_intact)**	**10.46**	10.96	9.47
**encounter complex (dimer_parsep)**	15.98	16.06	17.08
**encounter complex (dimer_sep)**	30.46	27.19	25.13
**TS-H_dimer_intact**	27.95	29.33	32.18
**TS-H_dimer_parsep**	**27.71**	27.92	30.94
**TS-H_dimer_sep**	41.10	38.78	40.94
**β-fluoride elimination without KHMDS complex**			
**HMDS-H + KCH_2CF_3 + $KHMDS_{monomer}$**	32.78	28.39	26.97
**TS-βF + HMDS-H + $KHMDS_{monomer}$**	33.28	28.63	27.19
**HMDS-H + $KHMDS_{monomer}$ + difluoroethene + KF**	9.00	6.84	1.90
**β-fluoride elimination with KHMDS complex**			
**HMDS-H + KCH_2CF_3-KHMDS**	**23.12**	20.00	20.59
**TS-βF-KHMDS + HMDS-H**	**23.49**	20.34	20.38
**HMDS-H + difluoroethene + KF-KHMDS**	**-6.87**	-8.33	-10.87

Both B3PW91-D3(BJ) and PBE0-D3(BJ) functionals gave mechanisms with reasonable energies given the reaction conditions. B3PW91-D3(BJ) carried forward as it gave the lowest overall barriers.

<u>Solvation of KHMDS</u>

Solvation of both monomeric and dimeric KHMDS with THF was investigated across each functional (BS1) and with single-point corrections (BS2) using B3PW91-D3(BJ), PBE0-D3(BJ) and ωB97X-D(2). The calculated energies (Supplementary Table 31) and a comparison (Supplementary Table 32) are provided below.

Supplementary Table 31: Calculated energies for solvation of KHMDS across different functionals.

	SCF (BS1, hartrees)	H corr. (BS1, hartrees)	H (BS1, hartrees)	G corr. (BS1, hartrees)	G (BS1, hartrees)	SCF (BS2, hartrees)
**B3PW91-D3(BJ)**						
**KHMDS (monomer)**	-1473.292432	0.244598	-1473.047835	0.178365	-1473.114068	-1473.352054
**KHMDS-(THF)$_1$**	-1705.790276	0.369307	-1705.420970	0.286471	-1705.503805	-1705.865395
**KHMDS-(THF)$_2$**	-1938.280455	0.493940	-1937.786515	0.389569	-1937.890885	-1938.371052
**KHMDS-(THF)$_3$**	-2170.777231	0.618663	-2170.158568	0.499907	-2170.277324	-2170.883191
**KHMDS (dimer)**	-2946.640924	0.492484	-2946.148439	0.386634	-2946.254290	-2946.759295
**[KHMDS-THF]$_2$**	-3411.630574	0.741987	-3410.888587	0.599217	-3411.031357	-3411.779879
**[KHMDS-THF$_2$]$_2$**	-3876.619438	0.991527	-3875.627912	0.815837	-3875.803601	-3876.799422
**THF**	-232.478140	0.122330	-232.355809	0.088289	-232.389851	-232.493835
**PBE0-D3(BJ)**						
**KHMDS (monomer)**	-1472.687311	0.244896	-1472.442415	0.178552	-1472.508759	-1472.744135
**KHMDS-(THF)$_1$**	-1704.986419	0.369936	-1704.616483	0.286587	-1704.699832	-1705.057900
**KHMDS-(THF)$_2$**	-1937.279475	0.494983	-1936.784492	0.388844	-1936.890631	-1937.365609
**KHMDS-(THF)$_3$**	-2169.577720	0.619857	-2168.957862	0.501160	-2169.076560	-2169.678389
**KHMDS (dimer)**	-2945.424246	0.492929	-2944.931317	0.386040	-2945.038206	-2945.536906
**[KHMDS-THF]$_2$**	-3410.017587	0.742918	-3409.274669	0.598241	-3409.419347	-3410.159527
**[KHMDS-THF$_2$]$_2$**	-3874.610618	0.992567	-3873.618051	0.812507	-3873.798111	-3874.781567
**THF**	-232.280585	0.122706	-232.157879	0.088702	-232.191883	-232.295479
**ωB97X-D(2)**						
**KHMDS (monomer)**	-1473.364265	0.245803	-1473.118462	0.180081	-1473.184184	-1473.423327
**KHMDS-(THF)$_1$**	-1705.854920	0.371719	-1705.483200	0.287688	-1705.567231	-1705.929934
**KHMDS-(THF)$_2$**	-1938.344201	0.497602	-1937.846599	0.396964	-1937.947237	-1938.435153
**KHMDS-(THF)$_3$**	-2170.837611	0.622811	-2170.214800	0.508636	-2170.328975	-2170.943956
**KHMDS (dimer)**	-2946.776903	0.495378	-2946.281525	0.390325	-2946.386578	-2946.893920
**[KHMDS-THF]$_2$**	-3411.759414	0.746093	-3411.013321	0.606065	-3411.153349	-3411.907980
**[KHMDS-THF$_2$]$_2$**	-3876.742919	0.998060	-3875.744860	0.831446	-3875.911474	-3876.922708
**THF**	-232.474363	0.123279	-232.351084	0.089251	-232.385112	-232.490549
**B3LYP-D3(BJ)**						
**KHMDS (monomer)**	-1473.561448	0.244546	-1473.316901	0.178338	-1473.383110	
**KHMDS-(THF)$_1$**	-1706.145646	0.369283	-1705.776363	0.283422	-1705.862224	
**KHMDS-(THF)$_2$**	-1938.732698	0.493984	-1938.238714	0.394440	-1938.338257	
**KHMDS-(THF)$_3$**	-2171.314615	0.618294	-2170.696322	0.499119	-2170.815496	
**KHMDS (dimer)**	-2947.179899	0.492545	-2946.687354	0.386628	-2946.793271	
**[KHMDS-THF]$_2$**	-3412.348247	0.742907	-3411.605340	0.603781	-3411.744465	
**[KHMDS-THF$_2$]$_2$**	-3877.516245	0.991181	-3876.525064	0.816806	-3876.699439	
**THF**	-232.566261	0.122161	-232.444100	0.088065	-232.478196	
**M06-2X-D3**						
**KHMDS (monomer)**	-1473.251739	0.245773	-1473.005965	0.180046	-1473.071693	
**KHMDS-(THF)$_1$**	-1705.705482	0.371365	-1705.334117	0.285765	-1705.419717	
**KHMDS-(THF)$_2$**	-1938.158539	0.497381	-1937.661158	0.395627	-1937.762912	
**KHMDS-(THF)$_3$**	-2170.614509	0.622380	-2169.992129	0.509579	-2170.104930	
**KHMDS (dimer)**	-2946.551214	0.494297	-2946.056917	0.388786	-2946.162428	
**[KHMDS-THF]$_2$**	-3411.459459	0.745735	-3410.713725	0.608630	-3410.850830	
**[KHMDS-THF$_2$]$_2$**	-3876.370320	0.996684	-3875.373636	0.829650	-3875.540670	
**THF**	-232.437903	0.123385	-232.314518	0.089420	-232.348483	
**M06-L-D3**						
**KHMDS (monomer)**	-1473.391573	0.246804	-1473.144769	0.181656	-1473.209916	
**KHMDS-(THF)$_1$**	-1705.912758	0.372181	-1705.540576	0.288177	-1705.624581	
**KHMDS-(THF)$_2$**	-1938.438799	0.496787	-1937.942012	0.399273	-1938.039527	
**KHMDS-(THF)$_3$**	-2170.959985	0.621449	-2170.338536	0.506069	-2170.453916	
**KHMDS (dimer)**	-2946.828675	0.495971	-2946.332704	0.388819	-2946.439856	
**[KHMDS-THF]$_2$**	-3411.873933	0.746082	-3411.127851	0.606862	-3411.267071	
**[KHMDS-THF$_2$]$_2$**	-3876.924152	0.996232	-3875.927920	0.828744	-3876.095408	
**THF**	-232.506404	0.122853	-232.383551	0.088925	-232.417479	
**BP86-D3(BJ)**						
**KHMDS (monomer)**	-1473.586722	0.238750	-1473.347971	0.171719	-1473.415003	

**KHMDS-(THF)₁**	-1706.162546	0.359991	-1705.802555	0.276471	-1705.886075	
**KHMDS-(THF)₂**						
**KHMDS-(THF)₃**	-2171.305791	0.601827	-2170.703964	0.482265	-2170.823526	
**KHMDS (dimer)**	-2947.232300	0.480694	-2946.751606	0.373375	-2946.858924	
**[KHMDS-THF]₂**						
**[KHMDS-THF₂]₂**	-3877.523668	0.964944	-3876.558725	0.787426	-3876.736242	
**THF**	-232.555787	0.118714	-232.437073	0.084310	-232.471477	

Supplementary Table 32 Comparison of solvation of KHMDS across different functionals (BS1). Additional single-point corrections (BS2) added to B3PW91-D3(BJ), PBE0-D3(BJ) and ωB97X-D(2) structures (in brackets). Energies in kcal mol^{-1}.

	**B3PW91-D3(BJ)**		**PBE0-D3(BJ)**		**ωB97X-D(2)**	
	**ΔG (kcal mol^{-1})**	**ΔH (kcal mol^{-1})**	**ΔG (kcal mol^{-1})**	**ΔH (kcal mol^{-1})**	**ΔG (kcal mol^{-1})**	**ΔH (kcal mol^{-1})**
**KHMDS (monomer)**	0.00 (0.00	0.00 (0.00)	0.00 (0.00)	0.00 (0.00)	0.00 (0.00)	0.00 (0.00)
**KHMDS-(THF)₁**	0.07 (0.20)	-10.87 (-10.75)	0.51 (0.66)	-10.16 (-10.01)	1.30 (1.44)	-8.57 (-8.42)
**KHMDS-(THF)₂**	1.81 (2.07)	-16.98 (-16.72)	1.19 (1.49)	-16.52 (-16.22)	4.50 (4.80)	-16.30 (-15.99)
**KHMDS-(THF)₃**	3.95 (4.42)	-27.17 (-26.70)	4.92 (5.45)	-26.24 (-25.71)	6.62 (7.42)	-27.04 (-26.24)
**KHMDS (dimer)**	0.00 (0.00)	0.00 (0.00)	0.00 (0.00)	0.00 (0.00)	0.00 (0.00)	0.00 (0.00)
**[KHMDS-THF]₂**	1.65 (1.94)	-17.90 (-17.61)	1.65 (1.97)	-17.32 (-17.00)	2.17 (2.68)	-18.59 (-18.08)
**[KHMDS-THF₂]₂**	6.33 (7.07)	-35.29 (-34.55)	4.79 (5.59)	-34.65 (-33.84)	9.76 (11.00)	-37.02 (-35.78)

	**B3LYP-D3(BJ)**		**M06-2X-D3**		**M06-L-D3**		**BP86-D3(BJ)**	
	**ΔG (kcal mol^{-1})**	**ΔH (kcal mol^{-1})**	**ΔG (kcal mol^{-1})**	**ΔH (kcal mol^{-1})**	**ΔG (kcal mol^{-1})**	**ΔH (kcal mol^{-1})**	**ΔG (kcal mol^{-1})**	**ΔH (kcal mol^{-1})**
**KHMDS (monomer)**	0.00	0.00	0.00	0.00	0.00	0.00	0.00	0.00
**KHMDS-(THF)₁**	-0.58	-9.64	0.29	-8.56	1.77	-7.69	0.25	-10.99
**KHMDS-(THF)₂**	0.78	-21.09	3.61	-16.41	3.36	-18.91		
**KHMDS-(THF)₃**	1.38	-29.57	7.66	-26.74	5.29	-27.05	3.71	-28.10
**KHMDS (dimer)**	0.00	0.00	0.00	0.00	0.00	0.00	0.00	0.00
**[KHMDS-THF]₂**	3.26	-18.69	5.37	-17.43	4.86	-17.60		
**[KHMDS-THF₂]₂**	4.15	-38.47	9.85	-36.80	9.01	-38.29	5.39	-36.91

In general, THF solvation to either monomeric or dimeric KHMDS was calculated to be endergonic at both BS1 and BS2 levels of theory. It should be noted that these calculations do not take concentrations into account, which is expected to be highly influential in the overall stability of solvated species as the concentration of neat THF is approximately 12.3 M.

The single crystal X-ray diffraction structure of crystals obtained from THF solutions of KHMDS were found to be the *bis*-ligated dimer (*i.e.* one THF per K).[59.60] A low temperature (-20 – -30 °C) solution-based dynamics study of KHMDS in THF concluded that at these temperatures, KHMDS likely exists as a *penta*-THF ligated monomer. The authors caution that room temperature NMR spectra do not show great enough resolution to distinguish between monomeric and dimeric structures, or the THF-ligation of dimers. Moreover, DFT calculations (M06-2X-D3(0)/def2-TZVP//M06-2X-D3(0)/def2-SVP) show monomer formation from the dimer is endergonic, as well as sequential THF ligation to the monomer at room temperature.

Explicit solvation of **TS-1** with one, two or three molecules of THF was considered. Energies of these transition states are given Supplementary Figure 90 below. CH_3CF_3, $[KHMDS]_2$ and n THF is used as a reference point for these energies as coordination of THF to $[KHMDS]_2$ was calculated to be endergonic.

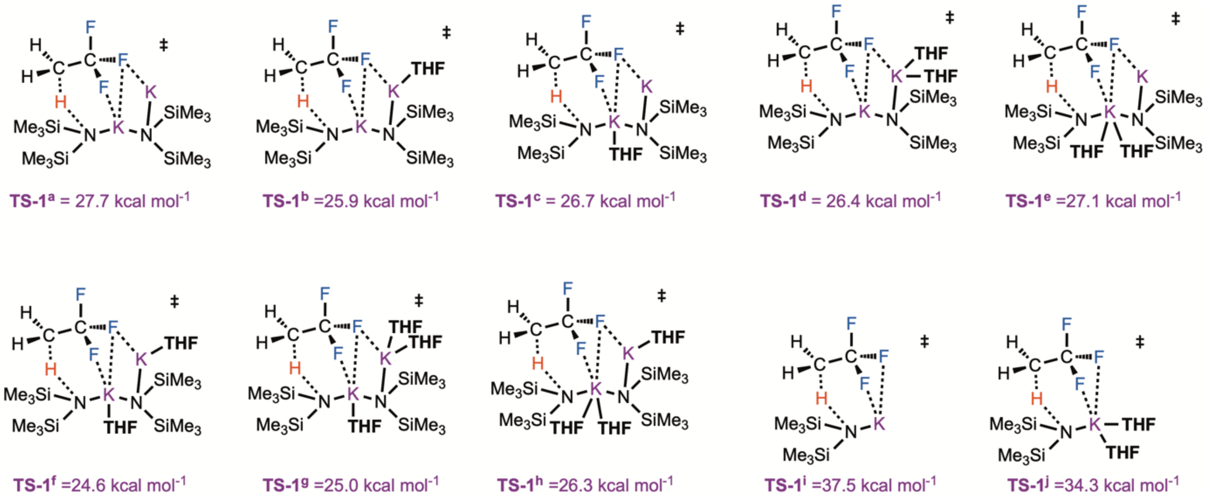

Supplementary Figure 90. Comparison of effects of explicit solvation and K+ coordination mode on **TS-1**. Gibbs energies in kcal mol^{-1} relative to dimeric ground state of $(KHMDS)_2$ + n THF + CH_3CF_3 (n = 0,1,2 or 3). B3PW91-D3(BJ) / def2-QZVPPD / SMD (THF) / goodvibes // B3PW91-D3(BJ) // def2-TZVP[C,H] / def2-TZVPP[N,O,F,Si,K] / SMD (THF).

Nuclearity of KHMDS

High-order nuclearity KHMDS structures have previously been invoked to explain their solution-based behaviour.[59,60] These high-order structures (3–5 monomer units) were attempted to be calculated using the ωB97X-D(2)/BS1 level of theory, but only the trimeric structure was found to converge. The trimer was calculated to be exergonic compared to the monomer, but less exergonic than the corresponding dimer by +0.3 kcal mol^{-1}. As expected, the enthalpy becomes increasingly negative as KHMDS monomer units are added due to the increase in bonds. The calculated energies can be found in Table 33 and a comparison found in Supplementary Table 34.

Supplementary Table 33: Calculated energies for high-order nuclearity KHMDS structures with ωB97X-D(2)/BS1.

ωB97X-D(2)	SCF (BS1, hartrees)	H corr. (BS1, hartrees)	H (BS1, hartrees)	G corr. (BS1, hartrees)	G (BS1, hartrees)
KHMDS (monomer)	-1473.364265	0.245803	-1473.118462	0.180081	-1473.184184
KHMDS (dimer)	-2946.776903	0.495378	-2946.281525	0.390325	-2946.386578
KHMDS (trimer)	-4420.172280	0.745727	-4419.426554	0.602043	-4419.570237

Supplementary Table 34: Comparison of free energy and enthalpy of higher-order KHMDS structures.

ωB97X-D(2)	ΔG (kcal mol^{-1})	ΔH (kcal mol^{-1})
KHMDS (monomer)	0.00	0.00
KHMDS (dimer)	-11.43	-27.99
KHMDS (trimer)	-11.10	-44.66

Hydrodefluorination mechanisms of 1,1,1-trifluoroethane by KHMDS

The mechanism proceeds first through nitrogen-centred deprotonation of CH_3–CF_3, to afford a metalated "KCH_2CF_3" species. This subsequently undergoes a β-fluoride elimination step to afford a "KF" species and the product 1,1-difluoroethene, alongside one equivalent of H–HMDS.

The deprotonation can proceed through either a monomeric (Supplementary Figure 91) or dimeric pathway (Supplementary Figure 92), determined by the nucleation of KHMDS. The monomeric pathway is higher in energy, even considering the energy required to split dimeric KHMDS into monomer units. Within the dimeric pathway, three transition state geometries were identified which differ by the geometry of the KHMDS moieties. Broadly these involved the KHMDS dimer with i) minimal geometric changes to its ground state structure (*TS-H_dimer_intact*), ii) a partially separated structure but with the two KHMDS units still facing each other in an *anti* arrangement (*TS-H_dimer_parsep*), and finally iii) where the dimer is completely separated and the two KHMDS units are approximately *syn* to each other (*TS-H_dimer_sep*).

These same structural variants were also calculated for the preceding encounter complex. For the transition state, the partially separated structure was found to give the lowest energy barrier.

After deprotonation, one equivalent of H–HMDS is formed, leaving one KHMDS monomer unit free from the original dimeric structure. Two separate pathways were calculated that either include this monomer as a separate species (KCH_2CF_3 + KHMDS) or explicitly incorporated as a bonded complex (KCH_2CF_3-KHMDS complex). Each had an associated β-fluoride elimination transition state geometry as well as the corresponding unbonded or bonded products, KF + KHMDS or KF-KHMDS complex, respectively. The pathway that included KCH_2CF_3 and KF still bonded to KHMDS was found to be lower in energy. Additionally, invoking these, or similar species may explain why the resultant "KF" product showed divergent reactivity to explicitly added KF powders when examining onwards reactivity.

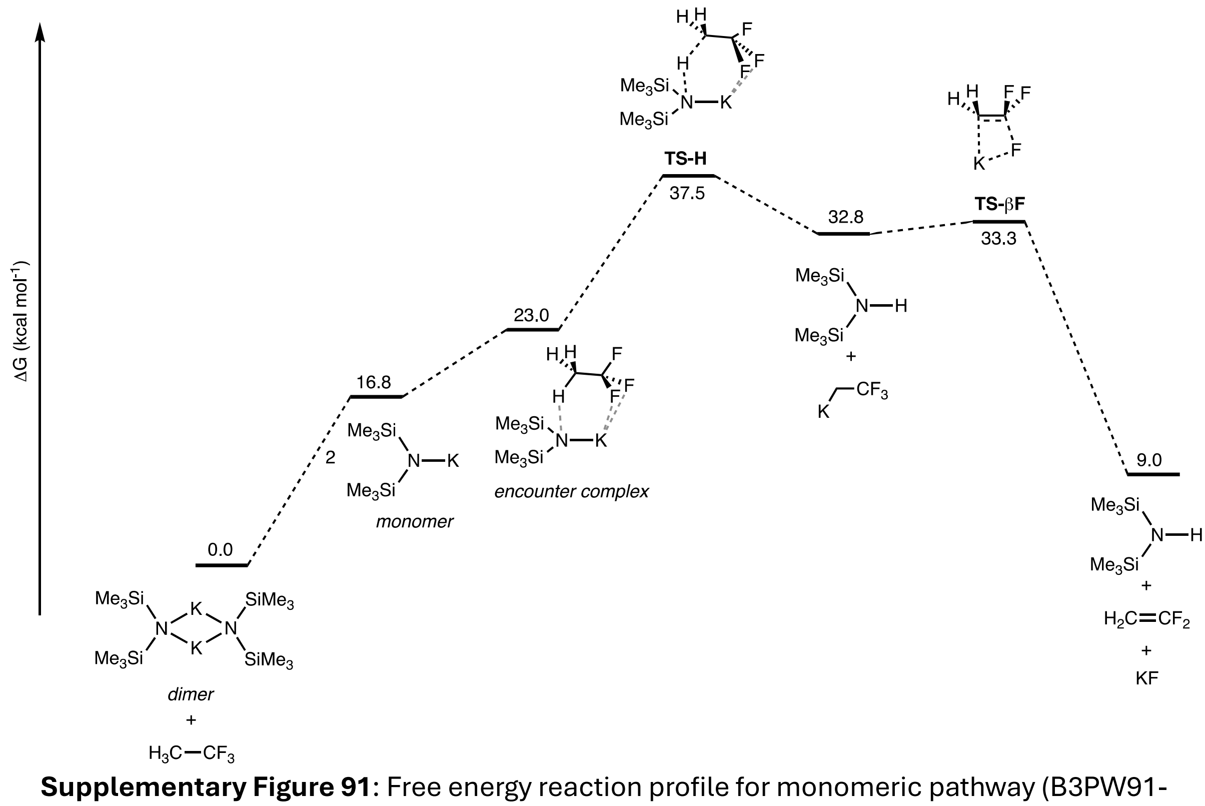

Supplementary Figure 91: Free energy reaction profile for monomeric pathway (B3PW91-D3(BJ)//BS2/*goodvibes*).

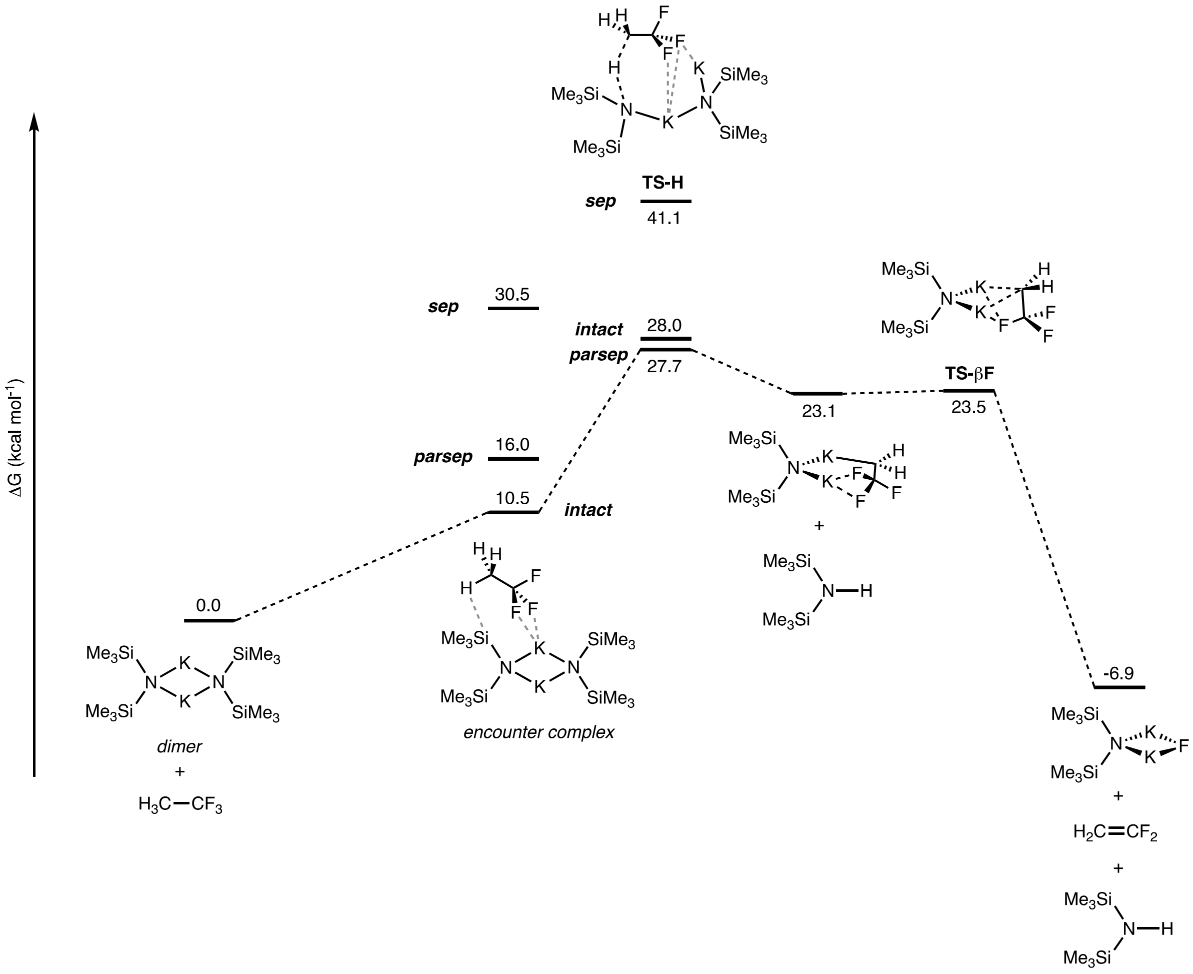

Supplementary Figure 92: Free energy reaction profile for dimeric pathway (B3PW91-D3(BJ)//BS2/*goodvibes*).

Other fluorinated gases

The dimeric reaction pathway identified for CH_3–CF_3 was calculated for two more gases (CH_2F–CF_3 and CHF_2–CF_3) with B3PW91-D3(BJ), PBE0-D3(BJ) and ωB97X-D(2) at BS1 level of theory. Additional single-point (BS2) and vibrational corrections were applied to the B3PW91-D3(BJ) optimised structures. The calculated energies for these are found in Tables S36-41 and an overview and comparison in Supplementary Table 42.

Supplementary Table 36: Calculated energies for the hydrodefluorination of $CH_2F–CF_3$ (B3PW91-D3(BJ)).

CH_2F-CF_3 // B3PW91-D3(BJ)	SCF (BS1, hartrees)	H corr. (BS1, hartrees)	H (BS1, hartrees)	G corr. (BS1, hartrees)	G (BS1, hartrees)	SCF (BS2)	G corr. (goodvibes)
CH_2F-CF_3	-476.834271	0.051503	-476.782768	0.015593	-476.818678	-476.858331	0.015695
KHMDS (dimer)	-2946.640924	0.492484	-2946.148439	0.386634	-2946.254290	-2946.759295	0.392718
encountercmplx_dimer_intact_1	-3423.478918	0.546741	-3422.932177	0.422004	-3423.056914	-3423.620824	0.431293
encountercmplx_dimer_intact_2	-3423.479037	0.546886	-3422.932152	0.422698	-3423.056339	-3423.620943	0.431768
encountercmplx_dimer_parsep_1	-3423.470554	0.545673	-3422.924881	0.418358	-3423.052196	-3423.612527	0.429139
encountercmplx_dimer_parsep_2	-3423.472731	0.545786	-3422.926944	0.419682	-3423.053048	-3423.614549	0.429907
encountercmplx_dimer_sep_1	-3423.447928	0.544872	-3422.903056	0.415607	-3423.032321	-3423.590029	0.426997
encountercmplx_dimer_sep_2	-3423.451133	0.545270	-3422.905863	0.416369	-3423.034764	-3423.593351	0.427380
TS-H_dimer_intact_1	-3423.451521	0.540013	-3422.911508	0.418892	-3423.032629	-3423.593076	0.426904
TS-H_dimer_intact_2	-3423.451501	0.540055	-3422.911446	0.418207	-3423.033294	-3423.593056	0.426695
TS-H_dimer_parsep_1	-3423.448747	0.539293	-3422.909454	0.413878	-3423.034869	-3423.590196	0.423675
TS-H_dimer_parsep_2	-3423.453419	0.539412	-3422.914008	0.415705	-3423.037714	-3423.594615	0.424906
TS-H_dimer_sep_1	-3423.428320	0.538959	-3422.889361	0.411145	-3423.017175	-3423.570032	0.422034
TS-H_dimer_sep_2	-3423.429409	0.538658	-3422.890751	0.411164	-3423.018245	-3423.570984	0.421786
HMDS-H	-873.983391	0.254553	-873.728838	0.193939	-873.789453	-874.029793	0.195565
K-$CHFCF_3$_KHMDS_1	-2549.455240	0.286546	-2549.168694	0.197815	-2549.257425	-2549.551345	0.204253
K-$CHFCF_3$_KHMDS_2	-2549.455266	0.286609	-2549.168658	0.198271	-2549.256996	-2549.551382	0.204544
TS- βF_KHMDS_1	-2549.445157	0.284977	-2549.160180	0.197938	-2549.247219	-2549.540927	0.203377
TS-βF_KHMDS_2	-2549.445150	0.284986	-2549.160164	0.197670	-2549.247480	-2549.540920	0.203251
KF_KHMDS	-2173.151104	0.250943	-2172.900160	0.176691	-2172.974412	-2173.228848	0.180437
$CHF=CF_2$	-376.318070	0.034636	-376.283434	0.001482	-376.316587	-376.337180	0.001492

Supplementary Table 37: Calculated energies for the hydrodefluorination of $CH_2F–CF_3$ (PBE0-D3(BJ)).

CH_2F-CF_3 // PBE0-D3(BJ)	SCF (BS1, hartrees)	H corr. (BS1, hartrees)	H (BS1, hartrees)	G corr. (BS1, hartrees)	G (BS1, hartrees)
CH_2F-CF_3	-476.543530	0.051806	-476.491723	0.015944	-476.527586
KHMDS (dimer)	-2945.424246	0.492929	-2944.931317	0.386040	-2945.038206
encountercmplx_dimer_intact_1	-3421.971422	0.547442	-3421.423980	0.422538	-3421.548884
encountercmplx_dimer_intact_2	-3421.971453	0.547678	-3421.423774	0.423308	-3421.548145
encountercmplx_dimer_parsep_1	-3421.966016	0.546317	-3421.419699	0.419199	-3421.546817
encountercmplx_dimer_parsep_2	-3421.967216	0.546540	-3421.420676	0.419688	-3421.547528
encountercmplx_dimer_sep_1	-3421.945755	0.545617	-3421.400138	0.415681	-3421.530074
encountercmplx_dimer_sep_2	-3421.946754	0.545952	-3421.400802	0.415509	-3421.531245
TS-H_dimer_intact_1	-3421.942728	0.540788	-3421.401940	0.419859	-3421.522869
TS-H_dimer_intact_2					
TS-H_dimer_parsep_1	-3421.941744	0.540266	-3421.401479	0.416606	-3421.525138
TS-H_dimer_parsep_2	-3421.944852	0.540325	-3421.404527	0.418011	-3421.526841
TS-H_dimer_sep_1	-3421.923355	0.539596	-3421.383758	0.410957	-3421.512398
TS-H_dimer_sep_2	-3421.925081	0.539437	-3421.385644	0.410864	-3421.514217
HMDS-H	-873.505955	0.254877	-873.251078	0.194048	-873.311907
K-$CHFCF_3$_KHMDS_1	-2548.430392	0.287176	-2548.143216	0.199181	-2548.231212
K-$CHFCF_3$_KHMDS_2	-2548.430430	0.287235	-2548.143195	0.200063	-2548.230367
TS-βF_KHMDS_1	-2548.418919	0.285489	-2548.133430	0.198348	-2548.220571
TS-βF_KHMDS_2	-2548.418916	0.285496	-2548.133420	0.198685	-2548.220231
KF_KHMDS	-2172.356637	0.251216	-2172.105421	0.176188	-2172.180448
$CHF=CF_2$	-376.084799	0.034854	-376.049945	0.001737	-376.083062

Supplementary Table 38: Calculated energies for the hydrodefluorination of $CH_2F–CF_3$ (ωB97X-D(2)).

CH_2F-CF_3 // ωB97X-D(2)	SCF (BS1, hartrees)	H corr. (BS1, hartrees)	H (BS1, hartrees)	G corr. (BS1, hartrees)	G (BS1, hartrees)
CH_2F-CF_3	-476.873891	0.051871	-476.822020	0.016055	-476.857837
KHMDS (dimer)	-2946.776903	0.495378	-2946.281525	0.390325	-2946.386578
encountercmplx_dimer_intact_1	-3423.655209	0.549612	-3423.105597	0.426395	-3423.228815
encountercmplx_dimer_intact_2	-3423.655264	0.549940	-3423.105324	0.427191	-3423.228073
encountercmplx_dimer_parsep_1	-3423.649247	0.548794	-3423.100453	0.424341	-3423.224905
encountercmplx_dimer_parsep_2	-3423.650150	0.548845	-3423.101305	0.424244	-3423.225905
encountercmplx_dimer_sep_1	-3423.630941	0.547845	-3423.083096	0.420542	-3423.210399
encountercmplx_dimer_sep_2	-3423.631925	0.548642	-3423.083283	0.424613	-3423.207312
TS-H_dimer_intact_1	-3423.622425	0.543306	-3423.079120	0.424535	-3423.197890
TS-H_dimer_intact_2	-3423.622412	0.543268	-3423.079144	0.423267	-3423.199145
TS-H_dimer_parsep_1	-3423.621278	0.542438	-3423.078840	0.419752	-3423.201526
TS-H_dimer_parsep_2	-3423.626597	0.542811	-3423.083786	0.422347	-3423.204250
TS-H_dimer_sep_1	-3423.607664	0.542202	-3423.065462	0.420819	-3423.186845
TS-H_dimer_sep_2	-3423.608213	0.541622	-3423.066592	0.416804	-3423.191409
HMDS-H	-874.011087	0.255917	-873.755170	0.196198	-873.814889
K-$CHFCF_3$_KHMDS_1	-2549.608350	0.288459	-2549.319891	0.201734	-2549.406616
K-$CHFCF_3$_KHMDS_2	-2549.608353	0.288356	-2549.319996	0.201793	-2549.406559
TS-βF_KHMDS_1	-2549.596716	0.286759	-2549.309956	0.200286	-2549.396430
TS-βF_KHMDS_2	-2549.596731	0.286558	-2549.310172	0.200816	-2549.395915
KF_KHMDS	-2173.276563	0.252738	-2173.023825	0.179240	-2173.097323
$CHF=CF_2$	-376.346790	0.034903	-376.311887	0.001818	-376.344972

Supplementary Table 39: Calculated energies for the hydrodefluorination of $CHF_2–CF_3$ (B3PW91-D3(BJ)).

CHF_2-CF_3 // B3PW91-D3(BJ)	SCF (BS1, hartrees)	H corr. (BS1, hartrees)	H (BS1, hartrees)	G corr. (BS1, hartrees)	G (BS1, hartrees)	SCF (BS2)	G corr. (goodvibes)
CHF_2-CF_3	-576.079541	0.044390	-576.035151	0.006535	-576.073006	-576.107879	0.006824
KHMDS (dimer)	-2946.640924	0.492484	-2946.148439	0.386634	-2946.254290	-2946.759295	0.392718
encountercmplx_dimer_intact_1	-3522.722481	0.538802	-3522.183680	0.409898	-3522.312584	-3522.868707	0.420480
encountercmplx_dimer_intact_2	-3522.723349	0.540192	-3522.183157	0.415276	-3522.308073	-3522.869515	0.424214
encountercmplx_dimer_intact_3	-3522.722755	0.538636	-3522.184119	0.408259	-3522.314497	-3522.869015	0.419488
encountercmplx_dimer_parsep_1	-3522.709603	0.538823	-3522.170780	0.409882	-3522.299721	-3522.855984	0.420943
encountercmplx_dimer_parsep_2	-3522.716352	0.538585	-3522.177767	0.411578	-3522.304775	-3522.862496	0.421421
encountercmplx_dimer_parsep_3	-3522.708897	0.538912	-3522.169986	0.410782	-3522.298116	-3522.855268	0.421403
encountercmplx_dimer_sep_1	-3522.697806	0.538251	-3522.159556	0.407409	-3522.290398	-3522.844203	0.418899
encountercmplx_dimer_sep_2	-3522.697102	0.538134	-3522.158968	0.407608	-3522.289494	-3522.843563	0.419128
encountercmplx_dimer_sep_3	-3522.718400	0.538355	-3522.180045	0.409986	-3522.308414	-3522.864512	0.420609
TS-H_dimer_intact	-3522.702279	0.532957	-3522.169321	0.409895	-3522.292384	-3522.847857	0.418068
TS-H_dimer_parsep	-3522.704000	0.533170	-3522.170830	0.410286	-3522.293714	-3522.849474	0.418426
TS-H_dimer_sep	-3522.687313	0.532728	-3522.154585	0.406342	-3522.280971	-3522.832759	0.416298
HMDS-H	-873.983391	0.254553	-873.728838	0.193939	-873.789453	-874.029793	0.195565
K-CF_2CF_3_KHMDS	-2648.707742	0.279572	-2648.428170	0.186631	-2648.521111	-2648.807937	0.194492
TS-βF_KHMDS_1	-2648.675357	0.278244	-2648.397113	0.187805	-2648.487552	-2648.775391	0.194417
KF_KHMDS	-2173.151104	0.250943	-2172.900160	0.176691	-2172.974412	-2173.228848	0.180437
$CF_2=CF_2$	-475.553919	0.027685	-475.526234	-0.007567	-475.561485	-475.577246	-0.007558

Supplementary Table 40: Calculated energies for the hydrodefluorination of CHF_2–CF_3 (PBE0-D3(BJ)).

CHF_2-CF_3 // PBE0-D3(BJ)	SCF (BS1, hartrees)	H corr. (BS1, hartrees)	H (BS1, hartrees)	G corr. (BS1, hartrees)	G (BS1, hartrees)
CHF_2-CF_3	-575.737580	0.044734	-575.692846	0.006935	-575.730645
KHMDS (dimer)	-2945.424246	0.492929	-2944.931317	0.386040	-2945.038206
encountercmplx_dimer_intact_1	-3521.164240	0.539446	-3520.624795	0.409342	-3520.754899
encountercmplx_dimer_intact_2	-3521.164540	0.541176	-3520.623364	0.416647	-3520.747894
encountercmplx_dimer_intact_3	-3521.166312	0.539396	-3520.626916	0.407705	-3520.758607
encountercmplx_dimer_parsep_1	-3521.154261	0.539490	-3520.614771	0.409516	-3520.744746
encountercmplx_dimer_parsep_2	-3521.159552	0.539405	-3520.620147	0.412262	-3520.747290
encountercmplx_dimer_parsep_3	-3521.153670	0.539575	-3520.614095	0.410074	-3520.743596
encountercmplx_dimer_sep_1	-3521.135726	0.538460	-3520.597266	0.404420	-3520.731306
encountercmplx_dimer_sep_2	-3521.141785	0.538979	-3520.602806	0.406607	-3520.735178
encountercmplx_dimer_sep_3	-3521.142638	0.539072	-3520.603565	0.410369	-3520.732268
TS-H_dimer_intact	-3521.142517	0.533837	-3520.608680	0.411277	-3520.731240
TS-H_dimer_parsep	-3521.142239	0.533212	-3520.609027	0.406415	-3520.735824
TS-H_dimer_sep	-3521.132472	0.533516	-3520.598956	0.406676	-3520.725797
HMDS-H	-873.505955	0.254877	-873.251078	0.194048	-873.311907
K-CF_2CF_3_KHMDS	-2647.631574	0.280218	-2647.351356	0.188995	-2647.442579
TS-βF_KHMDS	-2647.597065	0.278796	-2647.318269	0.189171	-2647.407894
KF_KHMDS	-2172.356637	0.251216	-2172.105421	0.176188	-2172.180448
CF_2=CF_2	-475.269125	0.027922	-475.241203	-0.007283	-475.276408

Supplementary Table 41: Calculated energies for the hydrodefluorination of CHF_2–CF_3 (ωB97X-D(2)).

CH_2F-CF_3 // ωB97X-D(2)	SCF (BS1, hartrees)	H corr. (BS1, hartrees)	H (BS1, hartrees)	G corr. (BS1, hartrees)	G (BS1, hartrees)
CHF_2-CF_3	-576.130342	0.044727	-576.085615	0.006980	-576.123362
KHMDS (dimer)	-2946.776903	0.495378	-2946.281525	0.390325	-2946.386578
encountercmplx_dimer_intact_1	-3522.909848	0.541461	-3522.368387	0.411710	-3522.498139
encountercmplx_dimer_intact_2	-3522.910941	0.543287	-3522.367654	0.419597	-3522.491344
encountercmplx_dimer_intact_3	-3522.909881	0.541438	-3522.368442	0.412789	-3522.497091
encountercmplx_dimer_parsep_1	-3522.905664	0.541735	-3522.363929	0.415915	-3522.489750
encountercmplx_dimer_parsep_2	-3522.905629	0.541586	-3522.364044	0.414590	-3522.491039
encountercmplx_dimer_parsep_3	-3522.898393	0.541911	-3522.356482	0.415878	-3522.482515
encountercmplx_dimer_sep_1	-3522.887765	0.541176	-3522.346589	0.410409	-3522.477355
encountercmplx_dimer_sep_2	-3522.881344	0.540631	-3522.340713	0.408393	-3522.472951
encountercmplx_dimer_sep_3	-3522.900439	0.541945	-3522.358494	0.414515	-3522.485924
TS-H_dimer_intact	-3522.885163	0.536193	-3522.348970	0.415635	-3522.469529
TS-H_dimer_parsep	-3522.887019	0.535647	-3522.351372	0.412310	-3522.474709
TS-H_dimer_sep	-3522.874502	0.535920	-3522.338582	0.411936	-3522.462565
HMDS-H	-874.011087	0.255917	-873.755170	0.196198	-873.814889
K-CF_2CF_3_KHMDS	-2648.872474	0.281126	-2648.591348	0.188569	-2648.683905
TS-βF_KHMDS	-2648.836947	0.279616	-2648.557332	0.188726	-2648.648221
KF_KHMDS	-2173.276563	0.252738	-2173.023825	0.179240	-2173.097323
CF_2=CF_2	-475.593348	0.027920	-475.565429	-0.007240	-475.600588

Supplementary Table 42: Comparison of dimeric reaction pathway for the hydrodefluorination of alternative fluorinated ethane derivatives. Additional single-point corrections (BS2) and vibrational corrections (*goodvibes*) added to B3PW91-D3(BJ) structures (in brackets). Energies in kcal mol^{-1}.

	B3PW91-D3(BJ)			PBE0-D3(BJ)			ωB97X-D(2)		
	CH_3-CF_3	CH_2F-CF_3	CHF_2-CF_3	CH_3-CF_3	CH_2F-CF_3	CHF_2-CF_3	CH_3-CF_3	CH_2F-CF_3	CHF_2-CF_3
start	0.00 (0.00)	0.00 (0.00)	0.00 (0.00)	0.00	0.00	0.00	0.00	0.00	0.00
encounter_intact	8.68 (10.46)	10.07 (12.35)	8.03 (11.36)	9.71	10.61	8.76	7.71	9.79	7.41
encounter_parsep	13.75 (15.98)	12.50 (15.42)	14.13 (16.66)	15.01	11.46	13.53	14.98	11.62	12.67
encounter_sep	27.71 (30.46)	23.97 (27.13)	11.85 (14.89)	24.63	21.68	23.56	22.61	21.35	20.45
TS-H_intact	26.83 (27.95)	24.90 (26.89)	21.91 (23.75)	28.64	26.93	23.60	30.74	28.41	25.36
TS-H_parsep	25.60 (27.71)	22.12 (24.79)	21.07 (22.96)	26.12	24.44	20.72	29.44	25.20	22.11
TS-H_sep	38.76 (41.10)	34.34 (37.66)	29.07 (32.11)	36.00	33.51	27.02	39.20	33.26	29.73
K-CH_2CF_3_KHMDS	22.37 (23.12)	16.37 (17.50)	10.50 (12.52)	19.48	14.23	9.01	20.58	14.41	6.99
TS-βF	23.83 (23.49)	22.61 (23.42)	31.56 (32.90)	20.63	21.12	30.78	20.84	21.09	29.39

end	-5.95 (-6.87)	-4.70 (-5.72)	1.22 (0.12)	-7.38	-6.04	0.06	-9.54	-8.01	-1.79

The same lowest energy pathway is identified for each fluorinated gas. The deprotonation transition state becomes increasingly lower in energy as more fluorine is added to the substrate. In the most fluorinated gas (CHF_2–CF_3) there is a change in rate-determining step, with the deprotonation now calculated to be rapid (+23.0 kcal mol^{-1}) while the β-fluoride elimination is high in energy (+32.9 kcal mol^{-1}). Due to the R-groups on each CHR_2–CF_3 derivative, there are different isomers for each intermediate and transition state depending on the orientation of the protons or fluorine atoms relative to KHMDS. For CH_2F–CF_3 there are two isomers depending on whether the fluorine in the CH_2F group is pointed towards or away from KHMDS, and for CHF_2–CF_3 there are three isomers. The energy for each is listed but only the lowest energy isomer is used for comparison in Supplementary Table 43.

Other donors

The lowest energy rate-determining transition state (deprotonation with a partially separated KHMDS dimer) identified for CH_3–CF_3 was calculated for four alternative CF_3-based fluorine donors that feature substituted aryl groups (1,1-di(2,4,6-trisubstituted-phenyl)-2,2,2-trifluoroethane ($CHAr_2$–CF_3) where substituents = H, F, Me, OMe). The B3PW91-D3(BJ) functional was employed with optimisations at the BS1 level of theory. Additional single-point (BS2) and vibrational corrections were subsequently applied. The calculated energies for these are found in Tables S43 and an overview and comparison in Supplementary Table 44.

Supplementary Table 43: Calculated energies for the rate-determining deprotonation of alternative fluorine donors (B3PW91-D3(BJ)).

$CHAr_2$–CF_3	SCF (BS1, hartrees)	H corr. (BS1, hartrees)	H (BS1, hartrees)	G corr. (BS1, hartrees)	G (BS1, hartrees)	SCF (BS2, hartrees)	G corr. (goodvibes)
			**Ar = phenyl**				
**$CHAr_2$-CF_3**	-839.736788	0.229513	-839.507275	0.172389	-839.5644	-839.783727	0.175638
**TS-H_dimer_parsep**	-3786.367506	0.718784	-3785.648722	0.580979	-3785.786527	-3786.531442	0.590418
**TS betaF KHMDS**	-2912.377464	0.463742	-2911.913722	0.359128	-2912.018335	-2912.496049	0.366887
**CAr_2=CF_2**	-739.238187	0.212923	-739.025264	0.158081	-739.080106	-739.280045	0.160858
			**Ar = 2,4,6-trifluorophenyl**				
**$CHAr_2$-CF_3**	-1435.189706	0.185423	-1435.004283	0.117827	-1435.071879	-1435.261231	0.121416
**TS-H_dimer_parsep**	-4381.814313	0.674444	-4381.139869	0.525038	-4381.289275	-4382.002404	0.536113
**CAr_2=CF_2**	-1334.695662	0.168925	-1334.526737	0.102686	-1334.592976	-1334.762167	0.106359
			**Ar = 2,4,6-trimethylphenyl**				
**$CHAr_2$-CF_3**	-1075.654085	0.404999	-1075.249085	0.326986	-1075.327099	-1075.718531	0.332164
**TS-H_dimer_parsep**	-4022.269212	0.894511	-4021.374701	0.73961	-4021.529602	-4022.450513	0.741215
**TS betaF KHMDS**	-4022.269212	0.894511	-4021.374701	0.73961	-4021.529602	-4022.4505127	0.741215
**CAr_2=CF_2**	-975.174127	0.387929	-974.786198	0.312919	-974.861209	-975.233419	0.317088
			**Ar = 2,4,6-trimethoxyphenyl**				
**$CHAr_2$-CF_3**	-1526.929891	0.439681	-1526.49021	0.348824	-1526.581068	-1527.017008	0.354681
**TS-H_dimer_parsep**	-4473.546406	0.928454	-4472.617952	0.759682	-4472.786724	-4473.749878	0.772331
**TS betaF KHMDS**	-3599.575053	0.674551	-3598.900502	0.534249	-3599.040804	-3599.733577	0.545901
**CAr_2=CF_2**	-1426.437514	0.423001	-1426.014513	0.33293	-1426.104584	-1426.519572	0.339090

Supplementary Table 44: Comparison of deprotonation free energy barrier with partially separated KHMDS dimer with various CF3-based fluorine donors (B3PW91-D3(BJ)//BS2/*goodvibes*).

	1,1,1-trifluoroethane	$CHAr_2$–CF_3			
		Ar = phenyl	Ar = 2,4,6-trifluorophenyl	Ar = 2,4,6-trimethylphenyl	Ar = 2,4,6-trimethoxyphenyl
**start**	0.00	0.00	0.00	0.00	0.00
**TS-H_parsep**	27.71	21.11	25.16	27.39	32.23
**Beta-F elimination**	23.49	7.08	-	6.93	4.39
**end**	-6.87	-17.04	-20.11	-28.88	-21.47

Computed coordinates

Mechanism

```
01_trifluoroethane_b3pw91.log

SCF (RB3PW91) =        -377.603893608
E(SCF)+ZPE(0 K)=       -377.551959
H(298 K)=              -377.546214
G(298 K)=              -377.579739
Lowest Frequency = 237.8960cm-1

C       -1.467130    0.312617    0.000754
H       -1.101317    0.829089   -0.886080
C       -0.970044    1.015704    1.218535
F       -1.387286    2.298471    1.278078
F        0.378572    1.049746    1.277552
F       -1.387249    0.425342    2.358994
H       -2.556665    0.315321    0.004768
H       -1.101884   -0.713793    0.005638
```

```
02_khmds_dimer_b3pw91.log

SCF (RB3PW91) =        -2946.64092352
E(SCF)+ZPE(0 K)=       -2946.186631
H(298 K)=              -2946.148439
G(298 K)=              -2946.254290
Lowest Frequency = 24.6746cm-1

K        3.744292    4.130319    5.692627
Si       6.531935    3.828174    7.964689
Si       5.838279    6.715084    7.124433
N        5.935826    5.048106    6.982747
C        6.588326    4.179439    9.817470
C        8.300714    3.306642    7.508267
C        5.494876    2.253407    7.783826
C        7.235176    7.622729    6.211453
C        5.867797    7.419754    8.874556
C        4.235110    7.363419    6.350494
H        6.897476    3.284198   10.367717
H        7.296517    4.976576   10.058963
H        5.607307    4.479993   10.196676
H        8.699944    2.599865    8.243447
H        8.369312    2.799666    6.539133
H        8.973719    4.170946    7.492191
H        4.474030    2.392218    8.158674
H        5.439785    1.910673    6.745801
H        5.930600    1.429314    8.357881
H        8.217411    7.261635    6.535887
H        7.178287    7.530740    5.121174
H        7.201064    8.696809    6.422945
H        6.827318    7.240153    9.367155
H        5.703919    8.502598    8.854831
H        5.085947    6.976824    9.498121
H        4.198629    8.457214    6.381237
H        4.134538    7.081233    5.297523
H        3.352523    7.003081    6.891950
N        5.125688    3.750965    3.326205
K        7.317208    4.668807    4.616292
Si       4.529423    4.970839    2.344280
Si       5.222562    2.083920    3.184798
C        4.473422    4.619683    0.491467
C        2.760472    5.491993    2.800475
C        5.566126    6.545792    2.525473
C        3.825315    1.176958    4.097967
C        5.192668    1.378996    1.434784
C        6.825516    1.435141    3.958777
H        4.164321    5.514927   -0.058804
H        3.765342    3.822504    0.249787
H        5.454545    4.319218    0.112455
H        2.361163    6.198638    2.065211
H        2.691677    5.999020    3.769571
H        2.087653    4.627547    2.816547
H        6.587036    6.407288    2.150687
H        5.621044    6.888329    3.563572
H        5.130264    7.369895    1.951537
H        2.843205    1.538580    3.773734
H        3.882435    1.268781    5.188249
H        3.858831    0.102881    3.886362
H        4.233141    1.558801    0.942265
H        5.356244    0.296109    1.454634
H        5.974572    1.821629    0.811075
H        6.861554    0.341321    3.928403
H        6.926345    1.717663    5.011632
H        7.708170    1.794964    3.417095
```

```
03_khmds_monomer_b3pw91.log

SCF (RB3PW91) =        -1473.29243231
E(SCF)+ZPE(0 K)=       -1473.067161
H(298 K)=              -1473.047835
G(298 K)=              -1473.114068
Lowest Frequency = 17.5348cm-1

K        4.266680    4.234493    5.792806
N        4.546593    3.652734    3.313501
Si       3.993066    4.841485    2.276697
Si       5.214158    2.141108    3.059850
C        2.668505    4.274097    1.051128
C        3.203521    6.248652    3.278201
C        5.341888    5.658887    1.232130
C        4.016019    0.871016    2.331023
C        6.732883    2.120565    1.933197
C        5.781714    1.405380    4.716283
H        2.260943    5.109160    0.470703
H        1.836234    3.787239    1.569681
H        3.081108    3.550296    0.340917
H        2.822278    7.037868    2.621913
H        3.925189    6.727553    3.951470
```

H	2.352375	5.897718	3.874102
H	5.799578	4.934965	0.550645
H	6.138929	6.058929	1.867556
H	4.950827	6.483549	0.625994
H	3.696059	1.166649	1.326944
H	3.115870	0.786488	2.948987
H	4.465595	-0.125329	2.256087
H	6.460594	2.406396	0.912096
H	7.195676	1.128709	1.885228
H	7.491097	2.829275	2.281418
H	6.253275	0.427250	4.575578
H	4.942109	1.245749	5.404296
H	6.521937	2.043617	5.213467

04_encountcmplx_monomer_b3pw91.log

SCF (RB3PW91) = -1850.90788454
E(SCF)+ZPE(0 K)= -1850.629559
H(298 K)= -1850.603605
G(298 K)= -1850.686423
Lowest Frequency = 22.6274cm-1

C	-5.264711	2.330607	-0.565915
C	-4.969147	1.052845	0.131265
H	-4.128323	1.202481	0.807412
H	-5.847094	0.745408	0.697995
H	-4.726990	0.296018	-0.622611
F	-4.188885	2.793195	-1.265848
F	-6.253870	2.201022	-1.485378
F	-5.632684	3.327336	0.254345
K	-4.330272	1.422215	-3.792091
N	-4.295570	-0.892990	-2.652482
Si	-2.795874	-1.393806	-2.103803
Si	-5.735481	-1.714330	-2.882646
C	-1.929840	-2.656639	-3.214858
C	-1.615366	0.087808	-2.017134
C	-2.804016	-2.166871	-0.376393
C	-6.865674	-0.736767	-4.054471
C	-5.566390	-3.434061	-3.650727
C	-6.754311	-1.952321	-1.306109
H	-1.863554	-2.290065	-4.244681
H	-2.476825	-3.603868	-3.238417
H	-0.911900	-2.870500	-2.870395
H	-0.643271	-0.203983	-1.606034
H	-2.007713	0.887060	-1.379947
H	-1.416826	0.509329	-3.010339
H	-3.249607	-1.496257	0.364509
H	-1.793199	-2.416376	-0.035424
H	-3.388856	-3.092660	-0.374054
H	-6.411507	-0.597931	-5.042811
H	-7.128618	0.247655	-3.649117
H	-7.811976	-1.263581	-4.215735
H	-5.003632	-3.393485	-4.588650
H	-6.544006	-3.879515	-3.865253
H	-5.036238	-4.114441	-2.976726
H	-7.648250	-2.558650	-1.489004
H	-7.086193	-0.991714	-0.900855
H	-6.165506	-2.452592	-0.530646

05_encountcmplx_dimer_intact_b3pw91.log

SCF (RB3PW91) = -3324.24975776
E(SCF)+ZPE(0 K)= -3323.742101
H(298 K)= -3323.697105
G(298 K)= -3323.820194
Lowest Frequency = 14.9912cm-1

K	4.249011	3.662552	5.410932
Si	7.127575	4.067054	7.830454
Si	5.594420	6.548546	7.133055
N	6.151227	4.991326	6.818950
C	8.830807	4.828907	8.157265
C	7.484004	2.381868	7.051210
C	6.404694	3.700130	9.538770
C	6.694999	7.889566	6.365076
C	5.437456	7.029958	8.952156
C	3.868583	6.812365	6.402332
H	9.447060	4.189129	8.798600
H	9.389767	4.992885	7.228984
H	8.734928	5.799126	8.654977
H	8.072540	1.760042	7.733915
H	6.572067	1.825977	6.815735
H	8.060870	2.462359	6.124831
H	6.438748	4.584732	10.180106
H	5.359153	3.387919	9.470904
H	6.962805	2.905724	10.045909
H	7.726443	7.790025	6.719442
H	6.710266	7.867075	5.270028
H	6.349122	8.890888	6.643155
H	6.403534	7.003336	9.464639
H	5.040935	8.046385	9.049567
H	4.758952	6.356452	9.483487
H	3.535846	7.844951	6.549111
H	3.836141	6.628942	5.324493
H	3.124702	6.169313	6.886861
N	5.448887	4.030856	2.931464
K	7.438232	5.124280	4.381635
Si	4.544024	5.237337	2.183028
Si	5.751533	2.495917	2.309447
C	4.484454	5.186828	0.295872
C	2.726771	5.240754	2.728416
C	5.217898	6.953798	2.608328
C	4.187679	1.481148	1.980535
C	6.745574	2.465080	0.702072
C	6.773024	1.469629	3.524557
H	3.891253	6.020744	-0.094656
H	4.033022	4.261005	-0.072637
H	5.487304	5.263072	-0.134307
H	2.148518	5.981109	2.165308
H	2.587929	5.489063	3.786106
H	2.268426	4.262675	2.548200
H	6.230229	7.096320	2.212515
H	5.238414	7.143566	3.685235
H	4.593867	7.738318	2.168106
H	3.546532	1.977272	1.245180
H	3.594760	1.345499	2.891790
H	4.422825	0.483592	1.593781
H	6.179217	2.882268	-0.134336
H	7.031119	1.442606	0.431882
H	7.665452	3.050968	0.802974
H	6.950012	0.468224	3.118162
H	6.282191	1.335804	4.492322
H	7.757056	1.911728	3.713510
C	3.387234	1.034334	7.663922
C	4.320108	0.408960	8.639735
H	3.930065	0.551545	9.646780
H	4.399062	-0.655618	8.421851
H	5.299692	0.875639	8.554168
F	3.235770	2.366659	7.868997
F	3.823073	0.914557	6.382025
F	2.155770	0.501828	7.696590

06_encountcmplx_dimer_parsep_b3pw91.log

SCF (RB3PW91) = -3324.23981002
E(SCF)+ZPE(0 K)= -3323.733039
H(298 K)= -3323.687822
G(298 K)= -3323.812117
Lowest Frequency = 20.3532cm-1

C	-7.262777	0.795790	1.712960
C	-7.218801	-0.138671	0.560598
H	-7.201019	-1.163378	0.929643
H	-8.113422	0.014711	-0.042816
H	-6.332322	0.067674	-0.052253
F	-6.263101	0.592637	2.606598
F	-7.124347	2.096505	1.319011
F	-8.407636	0.740783	2.412348

K	-5.967089	3.201225	-1.180714
N	-4.851398	0.841136	-1.677111
Si	-3.429474	0.435331	-0.901094
Si	-5.550868	0.213212	-3.060631
C	-1.850082	0.895178	-1.833210
C	-3.343140	1.361754	0.752313
C	-3.246732	-1.395807	-0.461077
C	-6.989213	1.312893	-3.635134
C	-4.392344	0.075398	-4.549484
C	-6.305392	-1.509845	-2.845004
H	-1.840800	1.957603	-2.095372
H	-1.770958	0.327803	-2.765643
H	-0.951514	0.688167	-1.241441
H	-2.378761	1.243735	1.254161
H	-4.122827	0.974120	1.413499
H	-3.500626	2.428569	0.569512
H	-4.117729	-1.756173	0.095645
H	-2.357115	-1.582210	0.150851
H	-3.157633	-2.007035	-1.364938
H	-6.653837	2.309714	-3.945604
H	-7.756907	1.427914	-2.860652
H	-7.489234	0.872602	-4.503985
H	-3.901697	1.032589	-4.752945
H	-4.924911	-0.229487	-5.457097
H	-3.606715	-0.664365	-4.364918
H	-6.753304	-1.876630	-3.775165
H	-7.086220	-1.513121	-2.078402
H	-5.542183	-2.231613	-2.537750
K	-4.775078	3.358759	2.794803
N	-5.263176	5.045458	0.770819
Si	-3.765698	5.605174	0.254508
Si	-6.717110	5.778967	1.177396
C	-3.593560	7.463279	-0.014564
C	-2.381987	5.157930	1.475736
C	-3.234840	4.830724	-1.392157
C	-7.318961	5.261003	2.904282
C	-6.744165	7.663590	1.211897
C	-8.138023	5.284634	0.020887
H	-3.759034	8.028472	0.906260
H	-4.303115	7.828264	-0.762294
H	-2.585704	7.699112	-0.372700
H	-1.430291	5.587000	1.145673
H	-2.210720	4.079533	1.563626
H	-2.580229	5.565003	2.474304
H	-3.203584	3.736930	-1.371503
H	-2.224790	5.159700	-1.656729
H	-3.888854	5.142160	-2.215198
H	-6.616331	5.557074	3.692670
H	-7.519972	4.187458	2.991194
H	-8.265312	5.761038	3.134458
H	-6.011943	8.063489	1.918810
H	-7.732358	8.021715	1.519657
H	-6.527649	8.092409	0.230157
H	-9.054756	5.815498	0.297587
H	-8.378948	4.217179	0.070627
H	-7.925535	5.549220	-1.021302

07_encountcmplx_dimer_sep_b3pw91.log

SCF (RB3PW91) = -3324.21392807
E(SCF)+ZPE(0 K)= -3323.708391
H(298 K)= -3323.662672
G(298 K)= -3323.789864
Lowest Frequency = 18.3546cm-1

C	-5.485144	2.311753	-0.591624
C	-5.993361	0.998273	-0.137352
H	-5.866790	0.917666	0.948331
H	-7.051108	0.925368	-0.387608
H	-5.438256	0.217116	-0.666034
F	-4.160287	2.475374	-0.350928
F	-5.635385	2.516185	-1.922358
F	-6.100153	3.365609	0.011075
K	-2.987720	1.390162	-2.823855
N	-4.072871	-0.898553	-2.346940
Si	-3.167397	-1.772258	-1.244299
Si	-5.255660	-1.297744	-3.457772
C	-2.301112	-3.303000	-1.942479
C	-1.761397	-0.686046	-0.571891
C	-4.141209	-2.376595	0.255198
C	-5.344196	0.023312	-4.820267
C	-4.997015	-2.939896	-4.360250
C	-7.008141	-1.383672	-2.746938
H	-1.704097	-3.051110	-2.825045
H	-3.023198	-4.068946	-2.240070
H	-1.629502	-3.752713	-1.202556
H	-1.225573	-1.190888	0.238105
H	-2.124125	0.259808	-0.156399
H	-1.016738	-0.464964	-1.347067
H	-4.609376	-1.545379	0.784806
H	-3.508493	-2.912994	0.970179
H	-4.939229	-3.058129	-0.057249
H	-4.402186	0.099870	-5.376423
H	-5.592260	1.012862	-4.418472
H	-6.122842	-0.216398	-5.552105
H	-4.003562	-2.989675	-4.816604
H	-5.738214	-3.083123	-5.154261
H	-5.086475	-3.785450	-3.671193
H	-7.723935	-1.758328	-3.486908
H	-7.356620	-0.398941	-2.422582
H	-7.046672	-2.049524	-1.878858
K	-7.175729	3.414769	2.612540
N	-6.177837	1.127814	3.277023
Si	-7.385084	-0.013867	3.452335
Si	-4.603238	1.303510	3.806257
C	-7.700342	-0.571986	5.231655
C	-9.038802	0.708119	2.852052
C	-7.151028	-1.599315	2.451984
C	-4.089218	3.131512	3.696070
C	-4.292505	0.798122	5.601536
C	-3.322971	0.375605	2.775223
H	-7.870909	0.286966	5.888669
H	-6.844139	-1.125165	5.629247
H	-8.576735	-1.225784	5.301324
H	-9.832570	-0.044659	2.897078
H	-8.987497	1.041894	1.808688
H	-9.378236	1.547626	3.471706
H	-7.088707	-1.396465	1.379218
H	-7.982519	-2.295438	2.608923
H	-6.230267	-2.114156	2.742043
H	-4.696785	3.772288	4.346980
H	-4.148799	3.516956	2.670911
H	-3.050238	3.265786	4.014403
H	-4.980349	1.309167	6.282565
H	-3.270345	1.035542	5.916583
H	-4.435723	-0.278587	5.734905
H	-2.300015	0.588134	3.104438
H	-3.397187	0.656439	1.722172
H	-3.473275	-0.705695	2.837260

08_TS-H_monomer_b3pw91.log

SCF (RB3PW91) = -1850.88053522
E(SCF)+ZPE(0 K)= -1850.608129
H(298 K)= -1850.582954
G(298 K)= -1850.662273
Lowest Frequency = -1178.7690cm-1

C	-5.031249	2.605672	-1.377155
C	-5.125543	1.351832	-0.669011
H	-4.453535	1.369889	0.191206
H	-6.155167	1.203661	-0.336820
H	-4.711801	0.158721	-1.513847
F	-3.776797	2.834289	-1.912448
F	-5.877534	2.670755	-2.470248
F	-5.299530	3.782328	-0.706185
K	-4.255629	1.075167	-4.121310
N	-4.398002	-0.831040	-2.200625

Si	-2.828180	-1.432118	-1.865515
Si	-5.792701	-1.782709	-2.500620
C	-1.970312	-2.033047	-3.431410
C	-1.771047	-0.044941	-1.166800
C	-2.807451	-2.846476	-0.628148
C	-7.052572	-0.747006	-3.448308
C	-5.429993	-3.299055	-3.547973
C	-6.618927	-2.349390	-0.912745
H	-1.850358	-1.229081	-4.166349
H	-2.524443	-2.842875	-3.912536
H	-0.966892	-2.407811	-3.203188
H	-0.757443	-0.408987	-0.970427
H	-2.175285	0.337126	-0.226278
H	-1.683149	0.800790	-1.855559
H	-3.253567	-2.535834	0.321310
H	-1.784052	-3.178106	-0.424396
H	-3.368382	-3.711379	-0.993468
H	-6.751972	-0.559814	-4.486107
H	-7.239117	0.213823	-2.959606
H	-8.011532	-1.272024	-3.502057
H	-5.000365	-3.023539	-4.515419
H	-6.354628	-3.852981	-3.742201
H	-4.735080	-3.983481	-3.054205
H	-7.539975	-2.906463	-1.111875
H	-6.873301	-1.493365	-0.280850
H	-5.952224	-2.997782	-0.337089

09_TS-H_dimer_intact_b3pw91.log

SCF (RB3PW91) = -3324.21801945
E(SCF)+ZPE(0 K)= -3323.715923
H(298 K)= -3323.671985
G(298 K)= -3323.791266
Lowest Frequency = -1065.2108cm-1

C	-5.332047	2.257022	-1.176095
C	-5.121839	0.835893	-0.986984
H	-4.428912	0.702595	-0.152707
H	-6.080727	0.383152	-0.723240
H	-4.577254	-0.153794	-2.042921
F	-4.174998	2.937179	-1.491632
F	-6.193709	2.543291	-2.213250
F	-5.850250	2.986349	-0.125398
K	-4.172057	1.530195	-3.961906
N	-4.167797	-1.095819	-2.733796
Si	-2.684124	-1.515443	-1.959175
Si	-5.584688	-2.061661	-2.928116
C	-1.694868	-2.840605	-2.870944
C	-1.608288	0.020521	-1.909233
C	-2.908319	-2.152807	-0.209650
C	-6.917526	-0.980682	-3.682750
C	-5.354599	-3.531343	-4.092767
C	-6.225030	-2.781028	-1.318676
H	-1.148231	-2.469626	-3.743897
H	-2.293622	-3.708286	-3.161851
H	-0.924205	-3.211200	-2.187172
H	-0.658853	-0.188690	-1.407103
H	-2.092936	0.836439	-1.366778
H	-1.361288	0.373527	-2.915039
H	-3.550569	-1.492865	0.378411
H	-1.942213	-2.224180	0.300204
H	-3.359340	-3.148642	-0.204948
H	-6.620243	-0.607306	-4.667013
H	-7.149126	-0.121810	-3.047959
H	-7.843233	-1.547005	-3.823229
H	-5.384661	-3.271631	-5.155962
H	-6.199714	-4.210820	-3.941389
H	-4.450570	-4.111688	-3.888016
H	-7.218457	-3.218092	-1.461869
H	-6.302895	-2.017432	-0.541117
H	-5.566587	-3.570492	-0.946730
N	-3.250690	0.706366	-6.394535
K	-3.135652	-1.789525	-5.487455
Si	-1.675010	1.276428	-6.456655
Si	-4.599260	0.800173	-7.386675
C	-1.068946	1.911021	-8.124318
C	-1.357472	2.691967	-5.234212
C	-0.438483	-0.073790	-5.957270
C	-4.326207	1.464381	-9.128735
C	-5.399307	-0.905131	-7.616465
C	-5.974599	1.880164	-6.650593
H	-0.014938	2.201945	-8.062232
H	-1.634851	2.786191	-8.454418
H	-1.156852	1.145375	-8.900300
H	-0.345816	3.090400	-5.364235
H	-1.424094	2.384672	-4.184574
H	-2.051285	3.524506	-5.395144
H	-0.450338	-0.920185	-6.654268
H	-0.607699	-0.452342	-4.943206
H	0.582782	0.320510	-5.964076
H	-3.584899	0.873549	-9.673714
H	-3.980180	2.501500	-9.114266
H	-5.260389	1.435006	-9.699553
H	-4.721860	-1.610912	-8.111574
H	-6.290543	-0.831105	-8.247803
H	-5.736388	-1.346719	-6.672125
H	-6.805374	1.975215	-7.357491
H	-5.619750	2.895272	-6.439623
H	-6.409700	1.467549	-5.733622

10_TS-H_dimer_parsep_b3pw91.log

SCF (RB3PW91) = -3324.21546418
E(SCF)+ZPE(0 K)= -3323.715064
H(298 K)= -3323.670293
G(298 K)= -3323.793226
Lowest Frequency = -1204.3260cm-1

C	-4.920032	0.845869	2.443673
C	-5.262832	-0.263447	1.589572
H	-4.603967	-1.105915	1.807586
H	-6.303807	-0.545926	1.759728
H	-5.100960	0.107948	0.146088
F	-3.621466	1.262478	2.300096
F	-5.674119	1.985433	2.167667
F	-5.067697	0.719733	3.810834
K	-3.966849	2.845316	-0.117160
N	-5.022670	0.421004	-1.071255
Si	-3.661435	-0.219506	-1.883766
Si	-6.606711	0.511671	-1.705803
C	-4.000395	-1.821681	-2.807273
C	-2.957420	1.001664	-3.136565
C	-2.302795	-0.568123	-0.630879
C	-7.568711	1.780595	-0.694511
C	-6.669237	1.036716	-3.507739
C	-7.541112	-1.112866	-1.563213
H	-4.736324	-1.680029	-3.603966
H	-4.386907	-2.586447	-2.127108
H	-3.087166	-2.213002	-3.267450
H	-2.125522	0.549324	-3.686802
H	-2.565089	1.912756	-2.671907
H	-3.711816	1.305348	-3.866788
H	-1.997913	0.320846	-0.069848
H	-1.410460	-0.948127	-1.138418
H	-2.616781	-1.320302	0.097466
H	-7.073907	2.753056	-0.765227
H	-7.601725	1.449651	0.347269
H	-8.594188	1.898111	-1.054462
H	-6.183680	2.000770	-3.676599
H	-7.709640	1.126264	-3.838047
H	-6.183987	0.299533	-4.153561
H	-8.583803	-1.007582	-1.880518
H	-7.538014	-1.474895	-0.530868
H	-7.079488	-1.885318	-2.184895
K	-7.462022	4.013459	1.662646
N	-5.326164	5.151813	0.531759
Si	-4.443616	5.769915	1.820949
Si	-5.802232	5.763353	-0.958315

C	-4.183683	7.636647	1.851773
C	-5.259969	5.365122	3.486679
C	-2.709942	5.013080	1.957919
C	-7.690930	5.788728	-1.138473
C	-5.236165	7.508787	-1.388298
C	-5.188440	4.687975	-2.394595
H	-5.136737	8.172672	1.819057
H	-3.579068	7.980231	1.008853
H	-3.668468	7.932252	2.771926
H	-4.635235	5.736080	4.305557
H	-5.372054	4.288556	3.656492
H	-6.236533	5.850442	3.606486
H	-2.140641	5.130244	1.028834
H	-2.728362	3.951577	2.228011
H	-2.136463	5.513197	2.745181
H	-8.158018	6.397089	-0.355360
H	-8.140708	4.789821	-1.118747
H	-7.980661	6.231228	-2.097143
H	-5.632970	8.252110	-0.692256
H	-5.576104	7.779624	-2.393625
H	-4.145329	7.588314	-1.376169
H	-5.572991	5.064987	-3.347501
H	-5.522119	3.648246	-2.324022
H	-4.095155	4.698194	-2.474597

11_TS-H_dimer_sep_b3pw91.log

SCF (RB3PW91) = -3324.19288492
E(SCF)+ZPE(0 K)= -3323.692938
H(298 K)= -3323.647884
G(298 K)= -3323.772266
Lowest Frequency = -1207.4437cm-1

C	-4.720581	2.759943	-1.076058
C	-5.388275	1.578070	-0.598052
H	-5.098425	1.339898	0.425965
H	-6.468536	1.678263	-0.706857
H	-5.011104	0.371239	-1.313579
F	-3.353566	2.647989	-1.101557
F	-5.068709	3.100177	-2.360721
F	-4.913596	3.963399	-0.377728
K	-3.437870	1.070803	-3.520058
N	-4.697787	-0.706283	-1.910901
Si	-3.464271	-1.602560	-1.131186
Si	-6.014656	-1.323126	-2.809868
C	-1.986035	-1.846816	-2.286330
C	-2.837348	-0.642589	0.353382
C	-3.981468	-3.317263	-0.566379
C	-6.773497	0.082247	-3.816336
C	-5.486618	-2.642716	-4.041884
C	-7.385610	-2.033728	-1.742598
H	-1.444103	-0.914666	-2.487482
H	-2.291264	-2.280702	-3.243725
H	-1.254629	-2.527876	-1.838739
H	-2.041386	-1.197262	0.859395
H	-3.634386	-0.460958	1.076495
H	-2.427227	0.327519	0.059692
H	-4.828780	-3.269214	0.120967
H	-3.155211	-3.809727	-0.043118
H	-4.264248	-3.954910	-1.408346
H	-6.143066	0.401253	-4.654697
H	-6.986649	0.957965	-3.197540
H	-7.720423	-0.242651	-4.259119
H	-4.733240	-2.260413	-4.738010
H	-6.342544	-2.979078	-4.636615
H	-5.065442	-3.521145	-3.546135
H	-8.227416	-2.372869	-2.355320
H	-7.762476	-1.287525	-1.037445
H	-7.029171	-2.888056	-1.161493
K	-6.384321	3.281247	1.895700
N	-5.825520	1.094416	3.154900
Si	-6.967426	-0.087222	2.855116
Si	-4.450659	1.115218	4.103927
C	-7.760939	-0.831062	4.405135
C	-8.408932	0.629381	1.845927
C	-6.331388	-1.546376	1.839848
C	-3.175592	2.324783	3.393341
C	-4.781969	1.709865	5.872143
C	-3.553777	-0.539844	4.298541
H	-8.208192	-0.049272	5.027960
H	-7.011810	-1.346703	5.015105
H	-8.545230	-1.556665	4.162278
H	-9.166446	-0.136093	1.648180
H	-8.082406	1.001067	0.868316
H	-8.915456	1.444549	2.376598
H	-5.950529	-1.202296	0.875461
H	-7.113275	-2.288101	1.646395
H	-5.512961	-2.052152	2.360051
H	-3.547264	3.356736	3.387083
H	-2.910848	2.056795	2.365588
H	-2.251473	2.329499	3.981472
H	-5.237205	2.706432	5.869095
H	-3.869863	1.761740	6.477166
H	-5.480830	1.034710	6.377521
H	-2.663181	-0.440017	4.929072
H	-3.233946	-0.939862	3.332813
H	-4.205860	-1.284069	4.767111

12_hmds-h_b3pw91.log

SCF (RB3PW91) = -873.983391115
E(SCF)+ZPE(0 K)= -873.746598
H(298 K)= -873.728838
G(298 K)= -873.789453
Lowest Frequency = 38.1447cm-1

N	4.581521	3.680015	3.355773
Si	4.060177	4.915299	2.257941
Si	5.186035	2.082899	3.058963
C	3.072871	4.145308	0.867187
C	3.001710	6.120714	3.219914
C	5.510037	5.837325	1.509447
C	3.803162	0.884093	2.657291
C	6.377289	2.107766	1.615156
C	6.060023	1.514916	4.612081
H	2.743401	4.916494	0.163738
H	2.184248	3.633889	1.246586
H	3.666033	3.420985	0.302234
H	2.659860	6.939335	2.579556
H	3.563249	6.562592	4.049058
H	2.118476	5.627493	3.635969
H	6.144358	5.167164	0.922608
H	6.132125	6.288073	2.288650
H	5.168454	6.638867	0.846588
H	3.279035	1.174752	1.742709
H	3.067835	0.849320	3.466748
H	4.191236	-0.129133	2.511288
H	5.894043	2.458234	0.698901
H	6.759629	1.101204	1.418097
H	7.231855	2.759037	1.817539
H	6.450031	0.499746	4.492471
H	5.377910	1.508180	5.467994
H	6.900686	2.171062	4.855839
H	4.560782	3.946628	4.331477

13_kch2cf3_b3pw91.log

SCF (RB3PW91) = -976.882209738
E(SCF)+ZPE(0 K)= -976.844914
H(298 K)= -976.836928
G(298 K)= -976.878114
Lowest Frequency = 22.8804cm-1

C	-1.634993	0.252459	0.013514
C	-1.056626	1.005136	1.080399
F	-1.627744	2.231775	1.274468
F	0.310272	1.276032	0.885205
F	-1.023944	0.472237	2.386195

```
H       -2.721502    0.234425    0.102897
H       -1.225553   -0.760247   -0.003947
K       -0.038499    1.631821   -1.872221
```

14_kch2cf3_khmds_b3pw91.log

```
SCF (RB3PW91) =       -2450.21409260
E(SCF)+ZPE(0 K)=      -2449.949250
H(298 K)=             -2449.921743
G(298 K)=             -2450.008931
Lowest Frequency = 16.6759cm-1
```

```
N       -3.395886    1.094666   -6.247129
K       -3.329354   -1.087235   -4.668759
Si      -1.797776    1.602094   -6.293279
Si      -4.583398    0.738296   -7.376692
C       -1.080255    1.990396   -7.991628
C       -1.505739    3.163225   -5.255028
C       -0.617079    0.329535   -5.521197
C       -4.089141    0.849875   -9.191500
C       -5.275835   -1.016826   -7.154407
C       -6.114246    1.845771   -7.196765
H       -0.043442    2.330949   -7.900257
H       -1.645520    2.781572   -8.492211
H       -1.085252    1.113888   -8.644933
H       -0.448089    3.443976   -5.288887
H       -1.745959    3.014745   -4.195689
H       -2.065886    4.028380   -5.628945
H       -0.672194   -0.645970   -6.018692
H       -0.785240    0.191273   -4.446253
H        0.419360    0.668484   -5.618119
H       -3.267427    0.167153   -9.425399
H       -3.769914    1.859783   -9.462321
H       -4.934363    0.584724   -9.835399
H       -4.511023   -1.791187   -7.289620
H       -6.047000   -1.213998   -7.905999
H       -5.762148   -1.161016   -6.182139
H       -6.849689    1.602910   -7.970555
H       -5.875264    2.908816   -7.317676
H       -6.628070    1.704225   -6.238484
K       -4.607526    2.906811   -4.574859
C       -5.187853    1.905361   -1.907135
C       -4.772872    0.560664   -2.063726
H       -4.614862    2.398284   -1.120759
H       -6.261178    1.959432   -1.719321
F       -3.416145    0.411589   -2.334561
F       -5.407956   -0.113220   -3.107318
F       -4.935311   -0.382703   -1.013533
```

15_TS-bF_b3pw91.log

```
SCF (RB3PW91) =       -976.881073173
E(SCF)+ZPE(0 K)=      -976.844701
H(298 K)=             -976.837114
G(298 K)=             -976.876512
Lowest Frequency = -131.5123cm-1
```

```
C       -1.622819    0.234184    0.053095
C       -1.066875    1.066791    1.048379
F       -1.823323    2.112526    1.525425
F        0.128661    1.859240    0.613825
F       -0.553475    0.434767    2.134617
H       -2.492339    0.658736   -0.440557
H       -1.737765   -0.798578    0.365064
K        0.581191    0.691389   -1.690996
```

16_TS-bF_khmds_b3pw91.log

```
SCF (RB3PW91) =       -2450.21446970
E(SCF)+ZPE(0 K)=      -2449.950586
H(298 K)=             -2449.924269
G(298 K)=             -2450.006593
Lowest Frequency = -157.4277cm-1
```

```
N       -3.574182    1.117870   -6.325186
K       -4.002349   -0.962813   -4.666467
Si      -1.916736    1.360967   -6.298699
Si      -4.776910    1.037646   -7.488589
C       -1.047643    1.435977   -7.969511
C       -1.438212    2.958701   -5.392915
C       -1.020929    0.001999   -5.321063
C       -4.261586    1.405204   -9.263892
C       -5.612171   -0.666287   -7.532672
C       -6.200511    2.235829   -7.114445
H        0.031665    1.565568   -7.836463
H       -1.409431    2.269785   -8.577043
H       -1.203172    0.516075   -8.540645
H       -0.359831    3.135476   -5.462547
H       -1.667486    2.916081   -4.321421
H       -1.933387    3.836488   -5.823254
H       -1.174834   -0.993799   -5.753604
H       -1.321842   -0.017557   -4.266695
H        0.059607    0.178514   -5.323453
H       -3.512935    0.694379   -9.623722
H       -3.837513    2.409488   -9.354513
H       -5.124635    1.347709   -9.935628
H       -4.888597   -1.471054   -7.705670
H       -6.344841   -0.717758   -8.344817
H       -6.168063   -0.886395   -6.613229
H       -6.966614    2.177614   -7.894515
H       -5.864712    3.278957   -7.082613
H       -6.709403    1.999660   -6.172502
C       -4.081857    0.833498   -2.073585
C       -5.379860    0.301487   -2.155069
F       -5.449806   -1.064490   -2.167795
F       -6.094154    0.596234   -3.452771
F       -6.351538    0.740138   -1.299663
H       -3.321647    0.109438   -1.799952
H       -4.015895    1.770741   -1.528150
K       -4.549174    2.718533   -4.365733
```

17_kf_b3pw91.log

```
SCF (RB3PW91) =       -699.813681900
E(SCF)+ZPE(0 K)=      -699.812995
H(298 K)=             -699.809272
G(298 K)=             -699.835369
Lowest Frequency = 301.3385cm-1
```

```
K        3.899640    3.954953    5.786959
F        4.772617    3.796001    3.615352
```

18_kf_khmds_b3pw91.log

```
SCF (RB3PW91) =       -2173.15110358
E(SCF)+ZPE(0 K)=      -2172.922977
H(298 K)=             -2172.900160
G(298 K)=             -2172.974412
Lowest Frequency = 20.5789cm-1
```

```
N       -3.372459    1.070420   -6.289849
K       -3.424776   -0.944591   -4.439352
Si      -1.794356    1.624825   -6.325645
Si      -4.559368    0.712144   -7.413326
C       -1.128335    2.170027   -8.003946
C       -1.543743    3.114984   -5.175674
C       -0.563763    0.334112   -5.674768
C       -4.018720    0.631212   -9.218574
C       -5.377812   -0.962683   -7.054926
C       -6.002201    1.945871   -7.363020
H       -0.108330    2.557811   -7.910446
H       -1.746249    2.963078   -8.435756
H       -1.104270    1.341780   -8.717188
H       -0.504386    3.457827   -5.210623
H       -1.747754    2.871594   -4.125981
H       -2.165517    3.969982   -5.465962
H       -0.648702   -0.613686   -6.217621
H       -0.692830    0.132617   -4.604455
```

H 0.467894 0.682013 -5.793068
H -3.213534 -0.096406 -9.358129
H -3.658182 1.598992 -9.577281
H -4.853487 0.329481 -9.860214
H -4.665181 -1.793468 -7.117264
H -6.165807 -1.171245 -7.786042
H -5.861094 -0.986371 -6.070900
H -6.710396 1.752633 -8.175744
H -5.653370 2.978042 -7.477716
H -6.583797 1.878013 -6.435623
K -4.641157 2.526804 -4.347079
F -4.593164 0.554098 -2.882678

19_difluoroethene_b3pw91.log

SCF (RB3PW91) = -277.090022750
E(SCF)+ZPE(0 K)= -277.053588
H(298 K)= -277.048924
G(298 K)= -277.079646
Lowest Frequency = 444.1583cm-1

C -2.774659 0.238202 -0.000000
H -2.229182 -0.693861 0.000000
H -3.854670 0.244180 0.000000
C -2.118195 1.375509 -0.000000
F -0.802801 1.500617 0.000000
F -2.667736 2.577151 0.000000

Functional testing
01a_trifluoroethane_pbe0.log

SCF (RPBE1PBE) = -377.365021981
E(SCF)+ZPE(0 K)= -377.312807
H(298 K)= -377.307085
G(298 K)= -377.340568
Lowest Frequency = 238.4042cm-1

C -1.466596 0.313368 0.002062
H -1.100564 0.830250 -0.884785
C -0.970125 1.015594 1.218343
F -1.386213 2.294217 1.277282
F 0.374188 1.049254 1.276787
F -1.386249 0.426826 2.354897
H -2.556407 0.316209 0.006414
H -1.101037 -0.713223 0.007239

01b_trifluoroethane_wb97xd.log

SCF (RwB97XD) = -377.632468190
E(SCF)+ZPE(0 K)= -377.580083
H(298 K)= -377.574382
G(298 K)= -377.607827
Lowest Frequency = 243.0652cm-1

C -1.467440 0.312138 -0.000046
H -1.101528 0.828740 -0.885847
C -0.969427 1.016552 1.220042
F -1.385919 2.295709 1.278779
F 0.375522 1.050224 1.278286
F -1.385798 0.427323 2.357009
H -2.556223 0.315281 0.004585
H -1.102191 -0.713471 0.005431

01c_trifluoroethane_b3lyp.log

SCF (RB3LYP) = -377.746975018
E(SCF)+ZPE(0 K)= -377.695226
H(298 K)= -377.689463
G(298 K)= -377.723026
Lowest Frequency = 236.1854cm-1

C -1.468087 0.311271 -0.001602
H -1.102135 0.827567 -0.887718
C -0.970131 1.015582 1.218304
F -1.388339 2.303550 1.279675
F 0.383743 1.050754 1.278976
F -1.388020 0.423968 2.364178
H -2.557002 0.314350 0.002667
H -1.103032 -0.714545 0.003758

01d_trifluoroethane_m062x.log

SCF (RM062X) = -377.601333521
E(SCF)+ZPE(0 K)= -377.548632
H(298 K)= -377.542945
G(298 K)= -377.576366
Lowest Frequency = 243.6372cm-1

C -1.467651 0.311954 -0.000474
H -1.099688 0.830902 -0.883454
C -0.970401 1.015273 1.217681
F -1.385729 2.292486 1.277616
F 0.372346 1.049488 1.276753
F -1.385376 0.427559 2.353356
H -2.555933 0.317310 0.007739
H -1.100572 -0.712476 0.009021

01e_trifluoroethane_m06l.log

SCF (RM06L) = -377.674052457
E(SCF)+ZPE(0 K)= -377.621969
H(298 K)= -377.616214
G(298 K)= -377.649748
Lowest Frequency = 239.4014cm-1

C -1.465845 0.314536 0.003924
H -1.104470 0.826096 -0.884004
C -0.970214 1.015670 1.218211
F -1.385503 2.296287 1.278242
F 0.375792 1.049193 1.278148
F -1.386843 0.427160 2.356699
H -2.552487 0.313386 0.003398
H -1.103434 -0.709832 0.003622

01f_trifluoroethane_bp86.log

SCF (RB-P86) = -377.739397448
E(SCF)+ZPE(0 K)= -377.689330
H(298 K)= -377.683446
G(298 K)= -377.717224
Lowest Frequency = 231.0159cm-1

C -1.469147 0.309816 -0.004135
H -1.101072 0.829537 -0.897835
C -0.969654 1.016270 1.219486
F -1.391313 2.317213 1.282242
F 0.397641 1.052099 1.281762
F -1.391357 0.419620 2.377334
H -2.566565 0.312063 -0.000754
H -1.101538 -0.724123 0.000138

02a_khmds_dimer_pbe0.log

SCF (RPBE1PBE) = -2945.42424615
E(SCF)+ZPE(0 K)= -2944.969687
H(298 K)= -2944.931317
G(298 K)= -2945.038206
Lowest Frequency = 18.7603cm-1

K 3.748386 4.127202 5.684459
Si 6.530974 3.824965 7.983674
Si 5.832805 6.714173 7.145938
N 5.935099 5.046573 7.001797
C 6.618883 4.181376 9.834285
C 8.288373 3.283253 7.507926
C 5.475514 2.259820 7.827031
C 7.223135 7.627543 6.229064

C	5.864163	7.420926	8.895023
C	4.227538	7.359539	6.374129
H	6.933037	3.284471	10.379698
H	7.335986	4.974923	10.063621
H	5.646179	4.487391	10.231442
H	8.685770	2.569264	8.237809
H	8.343167	2.778189	6.536263
H	8.972698	4.139273	7.489053
H	4.455202	2.419238	8.196562
H	5.418068	1.895941	6.795343
H	5.899446	1.441531	8.418954
H	8.209034	7.272492	6.550679
H	7.163805	7.535024	5.138226
H	7.183945	8.702010	6.440196
H	6.825099	7.245152	9.387415
H	5.698218	8.503742	8.871837
H	5.083825	6.978970	9.522067
H	4.181467	8.452805	6.423083
H	4.135718	7.094541	5.314911
H	3.344204	6.983142	6.904309
N	5.126060	3.752449	3.307275
K	7.312768	4.671832	4.624601
Si	4.530185	4.974049	2.325387
Si	5.228042	2.084816	3.163278
C	4.442472	4.617678	0.474759
C	2.772726	5.515674	2.801008
C	5.585553	6.539240	2.482168
C	3.837630	1.171765	4.080371
C	5.196353	1.377918	1.414257
C	6.833288	1.439255	3.934957
H	4.128376	5.514591	-0.070674
H	3.725399	3.824131	0.245328
H	5.415223	4.311674	0.077708
H	2.375334	6.229626	2.071086
H	2.717863	6.020757	3.772658
H	2.088444	4.659621	2.819866
H	6.605887	6.379921	2.112652
H	5.642946	6.903044	3.513886
H	5.161584	7.357547	1.890296
H	2.851756	1.527039	3.758918
H	3.897148	1.264221	5.171204
H	3.876543	0.097296	3.869200
H	4.235373	1.553792	0.921984
H	5.362145	0.295080	1.437505
H	5.976667	1.819715	0.787073
H	6.879160	0.345973	3.886158
H	6.925342	1.704398	4.994119
H	7.716595	1.815430	3.404576

02b_khmds_dimer_wb97xd.log

SCF (RwB97XD) = -2946.77690295
E(SCF)+ZPE(0 K)= -2946.319057
H(298 K)= -2946.281525
G(298 K)= -2946.386578
Lowest Frequency = 17.6900cm-1

K	3.720991	4.122517	5.698770
Si	6.533397	3.827816	7.987613
Si	5.832586	6.714808	7.147014
N	5.942138	5.050498	7.010070
C	6.615842	4.187495	9.838538
C	8.290084	3.279001	7.515569
C	5.485011	2.257689	7.823190
C	7.214623	7.635531	6.223857
C	5.865470	7.422316	8.896599
C	4.227044	7.357379	6.371063
H	6.921937	3.292580	10.389885
H	7.336589	4.977475	10.066103
H	5.644687	4.502990	10.230110
H	8.689864	2.578138	8.255294
H	8.335849	2.756601	6.553197
H	8.975782	4.132166	7.475845
H	4.461989	2.406686	8.187109
H	5.436245	1.901806	6.788457
H	5.911728	1.439204	8.411056
H	8.204054	7.277412	6.528232
H	7.139760	7.551473	5.133816
H	7.178636	8.707150	6.444527
H	6.830623	7.253837	9.382046
H	5.689768	8.502725	8.877839
H	5.094888	6.970940	9.527786
H	4.182384	8.450087	6.411612
H	4.139040	7.084096	5.313839
H	3.341807	6.985631	6.899689
N	5.119247	3.748520	3.298950
K	7.340381	4.676338	4.610362
Si	4.527416	4.971184	2.321725
Si	5.228734	2.084224	3.161789
C	4.445344	4.611994	0.470691
C	2.770389	5.518971	2.793763
C	5.574972	6.541759	2.486865
C	3.846313	1.163393	4.084250
C	5.196614	1.377029	1.412064
C	6.833861	1.441474	3.938436
H	4.139096	5.506972	-0.080469
H	3.724838	3.821884	0.242808
H	5.416644	4.296852	0.079190
H	2.370197	6.219547	2.053988
H	2.724314	6.041435	3.756090
H	2.085188	4.665408	2.833552
H	6.598048	6.393535	2.122789
H	5.623624	6.896972	3.521827
H	5.147763	7.360386	1.899553
H	2.857004	1.521694	3.779711
H	3.920908	1.247177	5.174335
H	3.882255	0.091826	3.863323
H	4.231668	1.545580	0.926228
H	5.372330	0.296620	1.430692
H	5.967462	1.828544	0.781300
H	6.878433	0.348764	3.897870
H	6.921335	1.714724	4.995715
H	7.719407	1.813144	3.410278

02c_khmds_dimer_b3lyp.log

SCF (RB3LYP) = -2947.17989891
E(SCF)+ZPE(0 K)= -2946.725466
H(298 K)= -2946.687354
G(298 K)= -2946.793271
Lowest Frequency = 22.9376cm-1

K	3.739310	4.127208	5.693080
Si	6.532539	3.822982	7.980389
Si	5.834897	6.718432	7.138829
N	5.940902	5.050360	7.003126
C	6.605140	4.173846	9.836920
C	8.298180	3.289181	7.508991
C	5.480552	2.252651	7.802937
C	7.228436	7.628752	6.214699
C	5.867517	7.429823	8.890575
C	4.224716	7.358432	6.362309
H	6.917577	3.279171	10.385110
H	7.315883	4.969740	10.072554
H	5.629089	4.476557	10.225502
H	8.695612	2.567121	8.229294
H	8.356984	2.799013	6.531245
H	8.978853	4.146906	7.502471
H	4.459474	2.403871	8.171140
H	5.425904	1.904471	6.767546
H	5.904063	1.427681	8.383793
H	8.212789	7.278476	6.542706
H	7.172508	7.520696	5.126399
H	7.188172	8.704968	6.410736
H	6.826931	7.250542	9.382280
H	5.704637	8.512224	8.869743
H	5.087617	6.989300	9.517339
H	4.176250	8.451001	6.399465

H	4.130440	7.081471	5.308143
H	3.343820	6.985959	6.897432
N	5.120452	3.748695	3.305886
K	7.322024	4.671895	4.615912
Si	4.528516	4.976008	2.328718
Si	5.226157	2.080594	3.170293
C	4.456069	4.625266	0.472159
C	2.762737	5.509379	2.800120
C	5.580116	6.546560	2.506394
C	3.832370	1.170545	4.094335
C	5.193565	1.369139	1.418571
C	6.836168	1.440388	3.946976
H	4.143561	5.519937	-0.075998
H	3.745438	3.829301	0.236427
H	5.432184	4.322695	0.083627
H	2.365071	6.231203	2.079710
H	2.703865	5.999719	3.777778
H	2.082292	4.651475	2.806835
H	6.601215	6.395683	2.138114
H	5.634726	6.894507	3.541864
H	5.156362	7.371551	1.925747
H	2.848104	1.521042	3.766304
H	3.888272	1.278527	5.182645
H	3.872412	0.094329	3.898246
H	4.234192	1.548487	0.926810
H	5.356354	0.286725	1.439438
H	5.973534	1.809585	0.791838
H	6.884419	0.347802	3.910013
H	6.930493	1.717526	5.001090
H	7.717143	1.812599	3.411801

02d_khmds_dimer_m062x.log

SCF (RM062X) = -2946.55121398
E(SCF)+ZPE(0 K)= -2946.094918
H(298 K)= -2946.056917
G(298 K)= -2946.162428
Lowest Frequency = 21.0947cm-1

K	3.738013	4.120518	5.676899
Si	6.521394	3.821453	7.958234
Si	5.827107	6.711810	7.115825
N	5.926616	5.044308	6.978713
C	6.588857	4.183085	9.808218
C	8.287922	3.296580	7.497902
C	5.476336	2.253033	7.780552
C	7.221168	7.617547	6.196958
C	5.876540	7.413309	8.866311
C	4.217440	7.352785	6.352352
H	6.890379	3.287459	10.360006
H	7.307694	4.972332	10.042129
H	5.613978	4.496917	10.190712
H	8.687083	2.593653	8.235515
H	8.359690	2.788250	6.530355
H	8.959131	4.161883	7.480810
H	4.453002	2.402175	8.142871
H	5.430324	1.910246	6.741633
H	5.899632	1.427824	8.360755
H	8.201564	7.260496	6.530394
H	7.174059	7.519870	5.107306
H	7.183303	8.691530	6.403795
H	6.847261	7.246394	9.340201
H	5.699118	8.493006	8.849706
H	5.112880	6.960138	9.503849
H	4.167952	8.445002	6.390313
H	4.121560	7.074631	5.297509
H	3.340683	6.976096	6.890952
N	5.134598	3.754716	3.330343
K	7.323182	4.678596	4.632121
Si	4.539941	4.977567	2.350742
Si	5.233606	2.087163	3.193469
C	4.472832	4.615961	0.500741
C	2.773339	5.502413	2.810801
C	5.584944	6.546002	2.528627
C	3.839423	1.181951	4.112713
C	5.183572	1.385417	1.443099
C	6.843246	1.445882	3.956718
H	4.171368	5.511577	-0.051096
H	3.754089	3.826673	0.266682
H	5.447806	4.302191	0.118435
H	2.374251	6.205312	2.073122
H	2.701453	6.010761	3.778330
H	2.102150	4.637094	2.827841
H	6.608312	6.396938	2.166372
H	5.630855	6.888713	3.567576
H	5.161661	7.371238	1.948452
H	2.859065	1.539336	3.779513
H	3.886817	1.279555	5.202358
H	3.876862	0.107960	3.905837
H	4.212735	1.552431	0.969478
H	5.360811	0.305691	1.459802
H	5.947116	1.838371	0.805268
H	6.892458	0.353645	3.918966
H	6.939460	1.724238	5.011478
H	7.719956	1.822273	3.417836

02e_khmds_dimer_m06l.log

SCF (RM06L) = -2946.82867512
E(SCF)+ZPE(0 K)= -2946.371016
H(298 K)= -2946.332704
G(298 K)= -2946.439856
Lowest Frequency = 11.2564cm-1

K	3.698568	4.111425	5.680744
Si	6.518831	3.824216	7.962402
Si	5.825812	6.701373	7.120680
N	5.911232	5.037421	6.986377
C	6.591518	4.167694	9.813925
C	8.287561	3.313769	7.497524
C	5.490263	2.247130	7.784002
C	7.229555	7.594767	6.205611
C	5.863846	7.422975	8.861184
C	4.229698	7.351770	6.341188
H	6.908222	3.275723	10.360680
H	7.298082	4.961330	10.063109
H	5.620369	4.462488	10.216213
H	8.708161	2.625860	8.235279
H	8.364856	2.792227	6.539049
H	8.957985	4.177584	7.459942
H	4.469878	2.371871	8.158801
H	5.425801	1.906883	6.746584
H	5.923028	1.416495	8.346625
H	8.210979	7.220276	6.512175
H	7.172938	7.523875	5.115539
H	7.224303	8.665206	6.426114
H	6.820924	7.255681	9.358655
H	5.703136	8.503905	8.835965
H	5.088615	6.998436	9.501881
H	4.186788	8.443435	6.354306
H	4.124470	7.058786	5.292680
H	3.340744	7.002783	6.875264
N	5.150054	3.761611	3.322670
K	7.362687	4.687583	4.628311
Si	4.542404	4.974802	2.346658
Si	5.235044	2.097624	3.188515
C	4.469902	4.631393	0.495114
C	2.773584	5.485041	2.811427
C	5.570840	6.551956	2.525200
C	3.831147	1.204690	4.103827
C	5.196597	1.375865	1.448086
C	6.831088	1.446940	3.967891
H	4.153202	5.523367	-0.051639
H	3.763408	3.837727	0.245830
H	5.441108	4.336670	0.092912
H	2.352923	6.172883	2.073643
H	2.696169	6.006579	3.769894
H	2.103285	4.621128	2.848987

H	6.591237	6.427323	2.150395
H	5.635271	6.892121	3.562645
H	5.138016	7.382602	1.962640
H	2.849795	1.579586	3.797518
H	3.888047	1.275443	5.193895
H	3.835933	0.134270	3.883222
H	4.239445	1.543251	0.950786
H	5.357154	0.294914	1.473370
H	5.971764	1.800238	0.807201
H	6.873756	0.355264	3.954936
H	6.936545	1.740076	5.016334
H	7.720031	1.795668	3.433631

02f_khmds_dimer_bp86.log

SCF (RB-P86) = -2947.23229972
E(SCF)+ZPE(0 K)= -2946.790523
H(298 K)= -2946.751606
G(298 K)= -2946.858924
Lowest Frequency = 26.4671cm-1

K	3.736084	4.126253	5.689670
Si	6.532235	3.816910	7.969562
Si	5.834105	6.723204	7.122608
N	5.926672	5.042819	6.978066
C	6.588621	4.171794	9.832808
C	8.311242	3.295440	7.505756
C	5.490432	2.232939	7.783692
C	7.239133	7.630803	6.198409
C	5.867116	7.432446	8.882539
C	4.222101	7.372692	6.341674
H	6.903602	3.273718	10.391054
H	7.297727	4.978767	10.075496
H	5.599550	4.471595	10.214804
H	8.719081	2.582558	8.242452
H	8.378670	2.785412	6.528688
H	8.989337	4.165810	7.488028
H	4.460956	2.369817	8.159039
H	5.436626	1.889113	6.737200
H	5.926749	1.399868	8.359750
H	8.229752	7.267773	6.522114
H	7.178450	7.535264	5.100221
H	7.209479	8.714122	6.405535
H	6.832038	7.246022	9.379927
H	5.707726	8.524111	8.866147
H	5.077746	6.989939	9.510980
H	4.181898	8.474545	6.367192
H	4.122106	7.085256	5.281399
H	3.331657	7.011320	6.885719
N	5.134717	3.756240	3.330939
K	7.325277	4.672905	4.619295
Si	4.529056	4.982093	2.339429
Si	5.226743	2.075801	3.186637
C	4.472923	4.627243	0.476170
C	2.749941	5.503344	2.803071
C	5.570633	6.566183	2.525491
C	3.821433	1.168771	4.111009
C	5.193401	1.366333	1.426803
C	6.838589	1.425961	3.967577
H	4.157983	5.525312	-0.082110
H	3.763882	3.820245	0.233378
H	5.462059	4.327483	0.094307
H	2.342056	6.216124	2.066302
H	2.682390	6.013430	3.780103
H	2.071958	4.632888	2.820817
H	6.600156	6.429493	2.150207
H	5.624303	6.909910	3.572023
H	5.134238	7.399246	1.949481
H	2.830917	1.532217	3.787449
H	3.882292	1.264204	5.209197
H	3.850609	0.085450	3.903823
H	4.228477	1.552932	0.929483
H	5.352527	0.274632	1.443312
H	5.982819	1.808574	0.798234
H	6.878430	0.324087	3.942368
H	6.938826	1.713680	5.027753
H	7.729074	1.786908	3.423323

03a_khmds_monomer_pbe0.log

SCF (RPBE1PBE) = -1472.68731137
E(SCF)+ZPE(0 K)= -1472.461792
H(298 K)= -1472.442415
G(298 K)= -1472.508759
Lowest Frequency = 22.0954cm-1

K	4.300303	4.228001	5.760519
N	4.560456	3.643577	3.278481
Si	3.959571	4.835324	2.273329
Si	5.253669	2.141787	3.041214
C	2.458215	4.320121	1.245072
C	3.376820	6.311534	3.316519
C	5.207085	5.538506	1.038656
C	4.175375	0.908730	2.096187
C	6.913164	2.172157	2.135313
C	5.604437	1.330569	4.722357
H	2.047086	5.157788	0.670318
H	1.659898	3.929220	1.885139
H	2.723505	3.530309	0.534333
H	2.990838	7.117851	2.683542
H	4.194422	6.743230	3.907354
H	2.560784	6.037221	3.997439
H	5.512260	4.773260	0.316941
H	6.111577	5.888634	1.547309
H	4.793474	6.380052	0.471465
H	4.015505	1.240482	1.064764
H	3.190297	0.808180	2.564229
H	4.630693	-0.087281	2.055141
H	6.792795	2.544268	1.112332
H	7.366532	1.176321	2.073672
H	7.624277	2.833377	2.642041
H	6.057133	0.340842	4.598646
H	4.686374	1.181342	5.304908
H	6.308155	1.919098	5.324678

03b_khmds_monomer_wb97xd.log

SCF (RwB97XD) = -1473.36426528
E(SCF)+ZPE(0 K)= -1473.137626
H(298 K)= -1473.118462
G(298 K)= -1473.184184
Lowest Frequency = 17.3596cm-1

K	4.244415	4.286304	5.820965
N	4.540704	3.662479	3.319837
Si	3.996131	4.836797	2.264931
Si	5.207855	2.150077	3.081889
C	2.654164	4.264547	1.060147
C	3.235819	6.278208	3.238530
C	5.343666	5.610685	1.185495
C	4.029834	0.885978	2.310445
C	6.756168	2.122979	1.995497
C	5.729729	1.404180	4.747832
H	2.261166	5.093478	0.462186
H	1.814190	3.806642	1.591787
H	3.048848	3.516362	0.365421
H	2.865317	7.059846	2.568101
H	3.969650	6.755711	3.898743
H	2.382738	5.954558	3.846258
H	5.787599	4.863229	0.520641
H	6.151215	6.022551	1.798984
H	4.953366	6.419907	0.559382
H	3.735229	1.192065	1.301818
H	3.114273	0.790215	2.902794
H	4.484841	-0.107278	2.235271
H	6.515182	2.416754	0.968984
H	7.208727	1.126672	1.954521
H	7.513355	2.820533	2.366721

H	6.194522	0.422190	4.615982
H	4.872578	1.254266	5.415156
H	6.463420	2.034564	5.263583

03c_khmds_monomer_b3lyp.log

SCF (RB3LYP) = -1473.56144757
E(SCF)+ZPE(0 K)= -1473.336204
H(298 K)= -1473.316901
G(298 K)= -1473.383110
Lowest Frequency = 21.4752cm-1

K	4.299252	4.230058	5.776903
N	4.561438	3.645147	3.282094
Si	3.969474	4.840685	2.274869
Si	5.244327	2.138018	3.043252
C	2.466800	4.327514	1.239360
C	3.389011	6.324825	3.316239
C	5.229876	5.534539	1.041173
C	4.153809	0.915849	2.089139
C	6.908281	2.161214	2.136084
C	5.587624	1.316654	4.726107
H	2.060864	5.163604	0.660149
H	1.664897	3.942118	1.876733
H	2.731457	3.534702	0.533366
H	3.008265	7.132988	2.684199
H	4.206339	6.751008	3.909785
H	2.572036	6.053429	3.995799
H	5.535924	4.765212	0.325769
H	6.132392	5.882207	1.553040
H	4.825086	6.375056	0.467669
H	3.996810	1.254280	1.060548
H	3.168429	0.821607	2.555700
H	4.599050	-0.083585	2.042475
H	6.791236	2.539978	1.116274
H	7.355275	1.163742	2.068633
H	7.623326	2.814113	2.646129
H	6.035286	0.325658	4.603337
H	4.667634	1.172785	5.305252
H	6.290502	1.901095	5.331823

03d_khmds_monomer_m062x.log

SCF (RM062X) = -1473.25173890
E(SCF)+ZPE(0 K)= -1473.025136
H(298 K)= -1473.005965
G(298 K)= -1473.071693
Lowest Frequency = 23.7510cm-1

K	4.464700	4.297146	5.719964
N	4.702911	3.699219	3.225786
Si	4.010737	4.863442	2.249309
Si	5.274461	2.141913	3.037865
C	2.606710	4.243152	1.145282
C	3.258168	6.235434	3.325855
C	5.216140	5.743890	1.090796
C	4.068375	0.947643	2.205538
C	6.885536	2.000917	2.060748
C	5.644900	1.388045	4.741686
H	2.109949	5.065034	0.620470
H	1.849612	3.710641	1.728272
H	2.985122	3.550897	0.387389
H	2.807019	7.019547	2.710847
H	4.015456	6.727719	3.947356
H	2.463745	5.855423	3.978400
H	5.663246	5.036178	0.386223
H	6.032853	6.206501	1.652644
H	4.724161	6.527833	0.506934
H	3.888547	1.234687	1.165482
H	3.101511	0.943371	2.717274
H	4.450966	-0.077751	2.202799
H	6.743141	2.343135	1.031217
H	7.252262	0.970756	2.019676
H	7.670363	2.619555	2.505630

H	6.044119	0.373997	4.647251
H	4.743633	1.308558	5.360835
H	6.396359	1.967615	5.290370

03e_khmds_monomer_m06l.log

SCF (RM06L) = -1473.39157267
E(SCF)+ZPE(0 K)= -1473.163870
H(298 K)= -1473.144769
G(298 K)= -1473.209916
Lowest Frequency = 31.5531cm-1

K	4.234629	4.259089	5.813078
N	4.533095	3.646593	3.320415
Si	4.003310	4.840765	2.283777
Si	5.197340	2.137880	3.065374
C	2.733637	4.282169	1.001444
C	3.177013	6.227965	3.277279
C	5.365963	5.689891	1.287242
C	3.990953	0.848222	2.392057
C	6.668053	2.102377	1.880852
C	5.817215	1.424692	4.708175
H	2.327113	5.117386	0.424997
H	1.889748	3.762045	1.460779
H	3.180956	3.589749	0.283517
H	2.783611	7.014005	2.628488
H	3.876242	6.723806	3.958258
H	2.330326	5.869282	3.870412
H	5.850781	4.995511	0.597349
H	6.149017	6.092115	1.934535
H	4.983841	6.520854	0.688117
H	3.628602	1.117664	1.397433
H	3.111367	0.746615	3.032457
H	4.445626	-0.142679	2.309192
H	6.354661	2.345187	0.862133
H	7.147867	1.120994	1.839396
H	7.434910	2.829086	2.158996
H	6.291013	0.449173	4.575598
H	5.004689	1.267197	5.424793
H	6.563120	2.066865	5.185751

03f_khmds_monomer_bp86.log

SCF (RB-P86) = -1473.58672172
E(SCF)+ZPE(0 K)= -1473.367670
H(298 K)= -1473.347971
G(298 K)= -1473.415003
Lowest Frequency = 19.2617cm-1

K	4.268571	4.232924	5.810963
N	4.544223	3.656826	3.330080
Si	3.986537	4.848284	2.276067
Si	5.219687	2.135487	3.063275
C	2.645375	4.272877	1.055139
C	3.203619	6.270092	3.282131
C	5.342432	5.656801	1.214015
C	4.018616	0.863562	2.314368
C	6.754695	2.125557	1.940022
C	5.779657	1.389631	4.730129
H	2.234062	5.108973	0.463613
H	1.807131	3.790823	1.585202
H	3.052462	3.534942	0.343953
H	2.818780	7.065955	2.622469
H	3.934996	6.752274	3.955609
H	2.347506	5.923605	3.888195
H	5.796528	4.921503	0.529376
H	6.151673	6.058594	1.846547
H	4.952834	6.486592	0.599339
H	3.703076	1.167260	1.302536
H	3.106797	0.775682	2.928789
H	4.468323	-0.141244	2.234802
H	6.488275	2.422838	0.912099
H	7.223739	1.128100	1.884113
H	7.516110	2.836632	2.301134

H	6.255163	0.404185	4.590849
H	4.930620	1.226696	5.418610
H	6.523215	2.029046	5.238473

04a_encountcmplx_monomer_pbe0.log

SCF (RPBE1PBE) = -1850.06340717
E(SCF)+ZPE(0 K)= -1849.784630
H(298 K)= -1849.758601
G(298 K)= -1849.841869
Lowest Frequency = 18.6211cm-1

C	-5.251496	2.347547	-0.573764
C	-5.032085	1.091773	0.186652
H	-4.220918	1.244221	0.898395
H	-5.947084	0.835019	0.720169
H	-4.776079	0.298366	-0.522416
F	-4.134896	2.744835	-1.238409
F	-6.199665	2.210996	-1.530180
F	-5.622841	3.383418	0.186562
K	-4.339329	1.409677	-3.762589
N	-4.292236	-0.919984	-2.642954
Si	-2.785105	-1.405020	-2.101626
Si	-5.734614	-1.730497	-2.891817
C	-1.931717	-2.702460	-3.181821
C	-1.605691	0.079519	-2.075253
C	-2.763685	-2.122180	-0.350466
C	-6.871277	-0.705160	-4.015423
C	-5.579280	-3.419907	-3.726845
C	-6.743266	-2.029290	-1.319376
H	-1.871088	-2.367880	-4.223159
H	-2.481340	-3.649191	-3.173211
H	-0.911808	-2.909044	-2.837750
H	-0.624048	-0.200254	-1.677640
H	-1.986466	0.892634	-1.447060
H	-1.428631	0.478335	-3.082303
H	-3.188537	-1.424125	0.378175
H	-1.747194	-2.367900	-0.022795
H	-3.354877	-3.043463	-0.305010
H	-6.428806	-0.533523	-5.004539
H	-7.124252	0.265636	-3.571075
H	-7.822832	-1.220575	-4.185107
H	-5.016830	-3.348839	-4.663708
H	-6.561076	-3.849009	-3.956762
H	-5.053835	-4.130591	-3.080089
H	-7.651512	-2.607673	-1.523088
H	-7.050772	-1.084345	-0.859746
H	-6.157008	-2.581488	-0.576999

04b_encountcmplx_monomer_wb97xd.log

SCF (RwB97XD) = -1851.00818120
E(SCF)+ZPE(0 K)= -1850.727555
H(298 K)= -1850.702062
G(298 K)= -1850.782878
Lowest Frequency = 28.9429cm-1

C	-5.286946	2.295462	-0.548018
C	-4.975704	1.038805	0.187878
H	-4.140377	1.218721	0.862793
H	-5.851771	0.733568	0.757852
H	-4.719213	0.268177	-0.542328
F	-4.216919	2.752284	-1.252959
F	-6.268490	2.128355	-1.462396
F	-5.668895	3.304085	0.244858
K	-4.313122	1.469803	-3.830049
N	-4.273110	-0.897173	-2.725789
Si	-2.789452	-1.392736	-2.138891
Si	-5.724492	-1.704071	-2.909768
C	-1.903895	-2.673137	-3.214050
C	-1.609438	0.087278	-2.032294
C	-2.830906	-2.151142	-0.404149
C	-6.873358	-0.738567	-4.071760
C	-5.586747	-3.441965	-3.644292
C	-6.711706	-1.904499	-1.307094
H	-1.789326	-2.312176	-4.241188
H	-2.471134	-3.607955	-3.257180
H	-0.905795	-2.908568	-2.829760
H	-0.643909	-0.201525	-1.605290
H	-2.017590	0.882725	-1.399986
H	-1.398109	0.512160	-3.020793
H	-3.275456	-1.469889	0.327533
H	-1.826928	-2.408858	-0.050892
H	-3.425465	-3.070496	-0.399322
H	-6.430865	-0.602996	-5.065173
H	-7.129062	0.247963	-3.667683
H	-7.821011	-1.266544	-4.216938
H	-5.043811	-3.427518	-4.594388
H	-6.571048	-3.885352	-3.827332
H	-5.046177	-4.110970	-2.967163
H	-7.616789	-2.501579	-1.460261
H	-7.021842	-0.932810	-0.911239
H	-6.113317	-2.399284	-0.535517

04c_encountcmplx_monomer_b3lyp.log

SCF (RB3LYP) = -1851.32093976
E(SCF)+ZPE(0 K)= -1851.042709
H(298 K)= -1851.016779
G(298 K)= -1851.099450
Lowest Frequency = 20.7980cm-1

C	-5.236392	2.342914	-0.587656
C	-5.042459	1.076611	0.169683
H	-4.238617	1.214455	0.890998
H	-5.966362	0.829441	0.689975
H	-4.788688	0.285550	-0.539155
F	-4.098533	2.733487	-1.241777
F	-6.178908	2.223946	-1.567101
F	-5.607995	3.387938	0.174843
K	-4.335055	1.423842	-3.756096
N	-4.291166	-0.924621	-2.647383
Si	-2.784001	-1.403209	-2.100670
Si	-5.737341	-1.730533	-2.889093
C	-1.925115	-2.703877	-3.180234
C	-1.604899	0.087312	-2.071666
C	-2.769811	-2.119840	-0.344423
C	-6.883276	-0.696158	-4.002292
C	-5.584845	-3.420442	-3.733715
C	-6.735224	-2.036855	-1.305827
H	-1.859567	-2.368320	-4.220027
H	-2.477024	-3.648059	-3.175935
H	-0.907765	-2.914148	-2.833420
H	-0.621746	-0.189468	-1.678441
H	-1.986333	0.896864	-1.441475
H	-1.433002	0.490059	-3.077180
H	-3.199335	-1.422105	0.380015
H	-1.755891	-2.363708	-0.010208
H	-3.359653	-3.040798	-0.300461
H	-6.445433	-0.520576	-4.991779
H	-7.126623	0.273368	-3.552459
H	-7.837650	-1.205079	-4.169061
H	-5.029233	-3.343883	-4.673081
H	-6.565449	-3.852609	-3.958967
H	-5.052217	-4.131096	-3.094631
H	-7.643768	-2.615430	-1.503189
H	-7.040018	-1.095291	-0.840357
H	-6.142780	-2.590104	-0.570785

04d_encountcmplx_monomer_m062x.log

SCF (RM062X) = -1850.86569040
E(SCF)+ZPE(0 K)= -1850.585002
H(298 K)= -1850.559626
G(298 K)= -1850.639598
Lowest Frequency = 27.5782cm-1

C	-5.226547	1.840187	-0.281364

C	-5.310456	0.582478	0.508354
H	-4.618332	0.649405	1.346175
H	-6.328669	0.470327	0.877297
H	-5.043451	-0.245757	-0.148537
F	-3.989359	2.059783	-0.785040
F	-6.052096	1.838267	-1.354460
F	-5.539137	2.933703	0.425981
K	-4.297087	1.491439	-3.535751
N	-4.319208	-0.893892	-2.490080
Si	-2.747853	-1.291739	-2.102967
Si	-5.746487	-1.627068	-2.940016
C	-2.125133	-2.927764	-2.815286
C	-1.543967	0.033447	-2.746454
C	-2.389037	-1.376289	-0.247228
C	-6.710774	-0.526232	-4.155590
C	-5.565312	-3.295411	-3.809490
C	-6.963296	-1.920157	-1.521120
H	-2.238378	-2.958317	-3.902626
H	-2.681485	-3.773687	-2.401670
H	-1.066859	-3.085053	-2.585318
H	-0.509876	-0.232287	-2.507539
H	-1.719682	1.012126	-2.284199
H	-1.594482	0.144524	-3.835661
H	-2.508363	-0.397377	0.225781
H	-1.364120	-1.709940	-0.057426
H	-3.064309	-2.074273	0.257217
H	-6.146926	-0.330999	-5.074960
H	-6.997757	0.432436	-3.707565
H	-7.643864	-1.009879	-4.459587
H	-4.914305	-3.219092	-4.685007
H	-6.534743	-3.675414	-4.146301
H	-5.132329	-4.044552	-3.140573
H	-7.849099	-2.463975	-1.863701
H	-7.305033	-0.974556	-1.090440
H	-6.504363	-2.504835	-0.717881

04e_encountcmplx_monomer_m06l.log

SCF (RM06L) = -1851.07888724
E(SCF)+ZPE(0 K)= -1850.797446
H(298 K)= -1850.772124
G(298 K)= -1850.851038
Lowest Frequency = 42.4221cm-1

C	-5.253644	2.237662	-0.604586
C	-4.983024	1.040932	0.227310
H	-4.170623	1.256050	0.917059
H	-5.876304	0.781559	0.789554
H	-4.710163	0.216799	-0.433945
F	-4.147811	2.651792	-1.284585
F	-6.184163	2.002844	-1.564368
F	-5.682012	3.297595	0.089284
K	-4.398476	1.462422	-3.816751
N	-4.282457	-0.881002	-2.686560
Si	-2.789452	-1.383891	-2.134966
Si	-5.730731	-1.685732	-2.884825
C	-1.908971	-2.629124	-3.251652
C	-1.614750	0.094030	-2.003382
C	-2.791219	-2.193961	-0.425456
C	-6.853271	-0.728776	-4.077650
C	-5.596072	-3.432070	-3.592174
C	-6.758309	-1.865131	-1.307159
H	-1.830850	-2.267492	-4.279838
H	-2.440690	-3.582370	-3.290151
H	-0.893710	-2.844179	-2.907381
H	-0.623289	-0.206806	-1.656002
H	-1.977212	0.847774	-1.299960
H	-1.459700	0.591765	-2.965776
H	-3.225352	-1.551326	0.344150
H	-1.782067	-2.453492	-0.094012
H	-3.370557	-3.121081	-0.429412
H	-6.402434	-0.600193	-5.066300
H	-7.122089	0.262233	-3.698245
H	-7.799893	-1.249569	-4.240160
H	-5.038881	-3.449422	-4.531684
H	-6.576257	-3.874849	-3.787848
H	-5.077676	-4.102224	-2.901586
H	-7.644564	-2.485335	-1.466218
H	-7.110837	-0.899308	-0.938436
H	-6.184663	-2.326549	-0.499320

04f_encountcmplx_monomer_bp86.log

SCF (RB-P86) = -1851.33830998
E(SCF)+ZPE(0 K)= -1851.068139
H(298 K)= -1851.041728
G(298 K)= -1851.125262
Lowest Frequency = 22.3271cm-1

C	-5.248830	2.354498	-0.570010
C	-4.986443	1.051436	0.105149
H	-4.148522	1.171109	0.803409
H	-5.885182	0.744370	0.654778
H	-4.743021	0.299805	-0.669825
F	-4.138458	2.820693	-1.255137
F	-6.237865	2.262092	-1.525261
F	-5.619132	3.357517	0.269511
K	-4.340572	1.439188	-3.771619
N	-4.301725	-0.881683	-2.635058
Si	-2.787994	-1.393820	-2.091665
Si	-5.745415	-1.721665	-2.878121
C	-1.931797	-2.675440	-3.207189
C	-1.599412	0.095933	-2.027617
C	-2.788316	-2.157290	-0.348112
C	-6.887214	-0.723248	-4.038273
C	-5.561740	-3.436447	-3.679909
C	-6.764621	-1.993520	-1.294205
H	-1.875100	-2.317063	-4.248810
H	-2.483724	-3.629290	-3.217155
H	-0.902849	-2.891013	-2.870784
H	-0.616190	-0.191854	-1.618742
H	-1.987811	0.908307	-1.390664
H	-1.406490	0.511406	-3.033293
H	-3.233065	-1.475711	0.395132
H	-1.770130	-2.408657	-0.004421
H	-3.379239	-3.088559	-0.334344
H	-6.434435	-0.566490	-5.033469
H	-7.153248	0.261587	-3.614532
H	-7.840908	-1.250876	-4.207963
H	-4.993905	-3.376832	-4.622990
H	-6.542018	-3.890330	-3.905715
H	-5.023629	-4.130160	-3.012917
H	-7.664203	-2.604025	-1.483784
H	-7.101189	-1.035361	-0.865841
H	-6.167782	-2.509034	-0.523583

05a_encountcmplx_dimer_intact_pbe0.log

SCF (RPBE1PBE) = -3322.79417468
E(SCF)+ZPE(0 K)= -3322.285779
H(298 K)= -3322.240775
G(298 K)= -3322.363308
Lowest Frequency = 25.4168cm-1

K	4.257093	3.683525	5.424660
Si	7.162288	4.091157	7.831002
Si	5.587351	6.561419	7.163282
N	6.170208	5.016038	6.833577
C	8.863606	4.859328	8.150949
C	7.525385	2.413245	7.038845
C	6.456176	3.705088	9.542382
C	6.648569	7.927604	6.385518
C	5.444656	7.036199	8.985261
C	3.845199	6.796423	6.459340
H	9.487738	4.216496	8.782154
H	9.414577	5.033337	7.219119
H	8.768604	5.825473	8.658048
H	8.137615	1.799445	7.708726

H	6.616988	1.842822	6.820994
H	8.082966	2.503536	6.100484
H	6.490068	4.583841	10.192777
H	5.411247	3.386651	9.480568
H	7.023911	2.908937	10.036896
H	7.684283	7.857242	6.736197
H	6.660901	7.900923	5.289857
H	6.277346	8.920929	6.661444
H	6.417241	7.024897	9.487218
H	5.033584	8.046746	9.087550
H	4.782525	6.352409	9.525156
H	3.491693	7.818574	6.632257
H	3.798445	6.634378	5.377428
H	3.122300	6.128347	6.943249
N	5.438112	4.011942	2.927625
K	7.415374	5.161606	4.371267
Si	4.548563	5.223775	2.166431
Si	5.719004	2.469161	2.309753
C	4.513297	5.178316	0.278557
C	2.725984	5.242373	2.691435
C	5.222470	6.938746	2.601037
C	4.145170	1.504646	1.889843
C	6.791132	2.420686	0.753776
C	6.640343	1.395733	3.563135
H	3.922177	6.012237	-0.116076
H	4.069202	4.252402	-0.100138
H	5.521345	5.259642	-0.139928
H	2.161667	5.995357	2.130002
H	2.580340	5.481428	3.751260
H	2.257248	4.271267	2.497755
H	6.246137	7.078120	2.232489
H	5.210274	7.138487	3.677338
H	4.613773	7.721696	2.136031
H	3.558937	2.016462	1.119432
H	3.499505	1.387114	2.767835
H	4.374032	0.500158	1.516345
H	6.290713	2.891688	-0.097083
H	7.032075	1.390620	0.468097
H	7.736953	2.950136	0.913998
H	6.826939	0.402017	3.141110
H	6.082458	1.242779	4.492589
H	7.615801	1.816397	3.830065
C	3.415338	1.051877	7.646976
C	4.332975	0.396212	8.615154
H	3.940455	0.529488	9.622954
H	4.394374	-0.666332	8.380750
H	5.321452	0.847683	8.540991
F	3.284856	2.379132	7.872438
F	3.853020	0.947060	6.368619
F	2.179578	0.539772	7.666704

05b_encountcmplx_dimer_intact_wb97xd.log

SCF (RwB97XD) = -3324.41478440
E(SCF)+ZPE(0 K)= -3323.904277
H(298 K)= -3323.859556
G(298 K)= -3323.982119
Lowest Frequency = 21.6031cm-1

K	4.241930	3.639777	5.374898
Si	7.099407	4.046302	7.833916
Si	5.567729	6.540756	7.151964
N	6.132729	4.989731	6.836119
C	8.813958	4.781674	8.162435
C	7.432173	2.363336	7.037267
C	6.385362	3.676550	9.545805
C	6.667728	7.891465	6.400149
C	5.398797	7.010561	8.973788
C	3.847633	6.808320	6.408135
H	9.422270	4.127016	8.795313
H	9.373985	4.948735	7.235702
H	8.731923	5.747244	8.671581
H	8.011285	1.724585	7.711827
H	6.512912	1.822558	6.794479

H	8.009946	2.446710	6.111513
H	6.422990	4.561760	10.186367
H	5.339872	3.362629	9.486704
H	6.948044	2.883909	10.049705
H	7.697517	7.794327	6.759691
H	6.690081	7.873925	5.305036
H	6.316306	8.889286	6.681815
H	6.363296	6.985419	9.489601
H	4.996736	8.023618	9.076648
H	4.723322	6.329780	9.499577
H	3.505578	7.834611	6.573344
H	3.830913	6.647483	5.325810
H	3.102705	6.148765	6.867716
N	5.466108	4.040736	2.871687
K	7.460928	5.123772	4.378283
Si	4.572319	5.260582	2.138475
Si	5.751388	2.496490	2.273951
C	4.518813	5.231104	0.250370
C	2.752616	5.271076	2.676616
C	5.249807	6.968340	2.594201
C	4.178013	1.477556	2.003472
C	6.693383	2.421668	0.637034
C	6.797937	1.492685	3.487805
H	3.945575	6.080244	-0.135403
H	4.048083	4.318045	-0.126172
H	5.523944	5.286614	-0.177470
H	2.180809	6.017627	2.116132
H	2.612850	5.513706	3.735687
H	2.288442	4.297026	2.490000
H	6.266908	7.113773	2.212802
H	5.259635	7.137692	3.675345
H	4.633503	7.762622	2.161912
H	3.526752	1.955031	1.264476
H	3.597016	1.365951	2.925442
H	4.404860	0.470050	1.639781
H	6.102274	2.827771	-0.187839
H	6.954933	1.390140	0.379422
H	7.623712	2.996186	0.690229
H	6.978180	0.487358	3.093893
H	6.318755	1.368141	4.463035
H	7.780541	1.944708	3.660465
C	3.444760	1.026639	7.724102
C	4.393476	0.483644	8.738301
H	3.982929	0.650168	9.732827
H	4.522387	-0.583684	8.565901
H	5.352069	0.990505	8.646241
F	3.234980	2.355420	7.869356
F	3.897655	0.870112	6.456242
F	2.240168	0.444570	7.769994

05c_encountcmplx_dimer_intact_b3lyp.log

SCF (RB3LYP) = -3324.93247676
E(SCF)+ZPE(0 K)= -3324.424349
H(298 K)= -3324.379629
G(298 K)= -3324.501420
Lowest Frequency = 24.5786cm-1

K	4.250595	3.660544	5.432305
Si	7.148801	4.079242	7.831882
Si	5.583434	6.563997	7.152627
N	6.163355	5.014432	6.838441
C	8.856650	4.842609	8.154207
C	7.506628	2.402054	7.027311
C	6.434630	3.694342	9.544506
C	6.663219	7.919833	6.372223
C	5.431952	7.046678	8.976259
C	3.844361	6.800770	6.432195
H	9.480718	4.198258	8.782431
H	9.405738	5.016942	7.222333
H	8.765011	5.807411	8.661874
H	8.103005	1.773686	7.696202
H	6.597789	1.844328	6.788448
H	8.077113	2.498784	6.098842

```
H        6.472907    4.572364   10.193660
H        5.388579    3.385225    9.479565
H        6.992891    2.894738   10.042052
H        7.692741    7.846568    6.736753
H        6.690403    7.876370    5.278662
H        6.296059    8.918857    6.628377
H        6.401034    7.032648    9.482316
H        5.023471    8.057465    9.076670
H        4.766871    6.365734    9.513910
H        3.492617    7.825292    6.587042
H        3.805588    6.621735    5.354673
H        3.116490    6.141686    6.918671
N        5.429347    4.009748    2.925131
K        7.427533    5.134313    4.374514
Si        4.541968    5.230288    2.175404
Si        5.719176    2.467402    2.311413
C        4.503309    5.191381    0.283109
C        2.717208    5.243889    2.707532
C        5.223954    6.943924    2.618735
C        4.143595    1.502293    1.881449
C        6.805009    2.426301    0.759722
C        6.634779    1.393970    3.574671
H        3.916088    6.027292   -0.110592
H        4.056481    4.268688   -0.096699
H        5.510408    5.268742   -0.135570
H        2.149955    6.007191    2.165246
H        2.578158    5.460383    3.771699
H        2.248259    4.277482    2.497990
H        6.246194    7.079522    2.247594
H        5.219608    7.135270    3.694524
H        4.618500    7.733179    2.162687
H        3.563595    2.014483    1.108304
H        3.493516    1.389536    2.755578
H        4.369798    0.496853    1.511504
H        6.310854    2.901096   -0.091140
H        7.051634    1.399889    0.469076
H        7.747228    2.957316    0.930186
H        6.827584    0.400847    3.156703
H        6.070432    1.241532    4.498574
H        7.605568    1.816300    3.850741
C        3.433571    1.051090    7.652960
C        4.379022    0.423585    8.619251
H        3.992634    0.553442    9.628778
H        4.465191   -0.637581    8.390764
H        5.353216    0.899395    8.533072
F        3.272723    2.386891    7.868677
F        3.863286    0.947370    6.360697
F        2.201602    0.509005    7.686779
```

05d_encountcmplx_dimer_intact_m062x.log

```
SCF (RM062X) =        -3324.15622964
E(SCF)+ZPE(0 K)=      -3323.645017
H(298 K)=             -3323.600586
G(298 K)=             -3323.722113
Lowest Frequency = 18.8354cm-1
```

```
K        4.269169    3.661627    5.393986
Si        7.137069    4.062576    7.811433
Si        5.546325    6.535985    7.148520
N        6.161376    5.005341    6.816438
C        8.856045    4.791844    8.121375
C        7.448624    2.376976    7.015047
C        6.431554    3.706599    9.529003
C        6.613364    7.929288    6.430317
C        5.355020    6.972034    8.975099
C        3.819420    6.744153    6.401515
H        9.464378    4.137098    8.753234
H        9.408946    4.949416    7.189154
H        8.782700    5.760388    8.625643
H        8.027356    1.729849    7.681535
H        6.519661    1.849223    6.778800
H        8.017417    2.460840    6.083628
H        6.491520    4.588055   10.172362
```

```
H        5.379595    3.414954    9.472335
H        6.981364    2.900654   10.025100
H        7.628166    7.872824    6.837558
H        6.690607    7.913761    5.338434
H        6.209439    8.911306    6.695080
H        6.318679    6.974714    9.492693
H        4.917216    7.968459    9.088818
H        4.702509    6.260505    9.488393
H        3.443324    7.760011    6.553939
H        3.805556    6.567663    5.321464
H        3.098764    6.066163    6.873242
N        5.474593    4.017426    2.918736
K        7.420908    5.193181    4.377697
Si        4.597101    5.247569    2.176561
Si        5.716068    2.464256    2.311549
C        4.587282    5.228700    0.288655
C        2.765875    5.256585    2.669419
C        5.278774    6.948223    2.654588
C        4.111148    1.504145    2.015348
C        6.668181    2.377560    0.681836
C        6.714073    1.417834    3.525865
H        4.032215    6.086276   -0.103558
H        4.114861    4.323481   -0.103745
H        5.602640    5.275665   -0.114581
H        2.217481    6.033480    2.127702
H        2.592778    5.445576    3.733711
H        2.302683    4.296233    2.420991
H        6.301530    7.090458    2.287328
H        5.271105    7.117027    3.735825
H        4.674183    7.746779    2.214713
H        3.485860    2.006019    1.270480
H        3.516635    1.409297    2.930481
H        4.309151    0.491677    1.650150
H        6.093461    2.799839   -0.145974
H        6.907299    1.341317    0.423557
H        7.610628    2.929662    0.746499
H        6.868776    0.410818    3.126584
H        6.216851    1.302806    4.493514
H        7.704335    1.845106    3.712576
C        3.518382    1.104716    7.637435
C        4.416239    0.535612    8.680244
H        3.948647    0.669198    9.654171
H        4.559275   -0.524149    8.477081
H        5.373092    1.053717    8.652631
F        3.289938    2.425194    7.811458
F        4.036399    0.996437    6.390664
F        2.320031    0.514871    7.599163
```

05e_encountcmplx_dimer_intact_m06l.log

```
SCF (RM06L) =         -3324.50872614
E(SCF)+ZPE(0 K)=      -3323.994728
H(298 K)=             -3323.950683
G(298 K)=             -3324.069328
Lowest Frequency = 28.9940cm-1
```

```
K        4.259128    3.629869    5.361056
Si        7.141184    4.081325    7.820714
Si        5.537225    6.522801    7.154349
N        6.144383    4.993094    6.825491
C        8.842775    4.847405    8.133096
C        7.494049    2.407883    7.019547
C        6.452243    3.698577    9.537129
C        6.616899    7.909680    6.445863
C        5.320318    6.970276    8.973642
C        3.824088    6.742190    6.385607
H        9.478165    4.200015    8.743577
H        9.394673    5.045900    7.209596
H        8.760465    5.800609    8.661279
H        8.067246    1.757032    7.684781
H        6.581524    1.864641    6.760728
H        8.079056    2.493368    6.099926
H        6.513114    4.562418   10.201462
H        5.401652    3.405700    9.495137
```

H	7.000548	2.886753	10.022605
H	7.633794	7.852002	6.843999
H	6.695797	7.903367	5.355342
H	6.230802	8.897693	6.709656
H	6.268741	6.975903	9.515466
H	4.885668	7.967218	9.083588
H	4.657407	6.272359	9.489345
H	3.443466	7.756285	6.528141
H	3.818103	6.567779	5.306494
H	3.085774	6.072289	6.837943
N	5.527711	4.040987	2.896207
K	7.497805	5.169394	4.400559
Si	4.633830	5.258123	2.163005
Si	5.737729	2.480412	2.312077
C	4.590752	5.244883	0.277689
C	2.808571	5.257070	2.678183
C	5.300236	6.961825	2.639538
C	4.126022	1.501347	2.144338
C	6.585031	2.335429	0.632306
C	6.803291	1.468761	3.495847
H	4.043635	6.107518	-0.111243
H	4.097655	4.352673	-0.114906
H	5.592837	5.276591	-0.155095
H	2.237537	6.006192	2.123492
H	2.637904	5.481472	3.734597
H	2.341345	4.290354	2.471861
H	6.318893	7.124506	2.274547
H	5.300694	7.130552	3.719668
H	4.691848	7.762316	2.212109
H	3.457693	1.952223	1.406474
H	3.564673	1.441028	3.081905
H	4.304645	0.471716	1.823083
H	5.960086	2.706138	-0.181666
H	6.827819	1.295311	0.399584
H	7.520518	2.898869	0.599768
H	6.953309	0.454279	3.118242
H	6.357977	1.362930	4.488172
H	7.799790	1.896255	3.636384
C	3.437679	1.090780	7.633004
C	4.351368	0.463128	8.619851
H	3.961293	0.621002	9.621868
H	4.415920	-0.603117	8.420292
H	5.340267	0.906829	8.542017
F	3.302486	2.427032	7.822626
F	3.883172	0.957980	6.354964
F	2.203327	0.577681	7.656589

05f_encountcmplx_dimer_intact_bp86.log

SCF (RB-P86) = -3324.97671204
E(SCF)+ZPE(0 K)= -3324.482622
H(298 K)= -3324.437103
G(298 K)= -3324.560152
Lowest Frequency = 26.8015cm-1

K	4.248613	3.644312	5.427928
Si	7.145622	4.068081	7.825743
Si	5.582908	6.560211	7.134779
N	6.151157	4.994415	6.811741
C	8.860328	4.836614	8.141623
C	7.499136	2.378741	7.028228
C	6.429333	3.698781	9.547743
C	6.681377	7.914826	6.360451
C	5.424784	7.040184	8.965575
C	3.844548	6.809417	6.400206
H	9.490191	4.193254	8.780111
H	9.415843	5.006466	7.202921
H	8.767709	5.812441	8.646570
H	8.092103	1.741630	7.705833
H	6.580166	1.822249	6.783647
H	8.078775	2.466908	6.094210
H	6.466612	4.590389	10.193147
H	5.375037	3.387954	9.483094
H	6.989993	2.898167	10.059549
H	7.721197	7.822507	6.716974
H	6.698212	7.889332	5.257222
H	6.328883	8.923561	6.635556
H	6.399239	7.022127	9.479738
H	5.015468	8.059556	9.069936
H	4.750534	6.353942	9.502453
H	3.499670	7.847799	6.537952
H	3.810917	6.615164	5.315812
H	3.099319	6.160948	6.893450
N	5.435767	4.018198	2.951391
K	7.432794	5.132024	4.374181
Si	4.535452	5.237934	2.186988
Si	5.722661	2.473503	2.307660
C	4.497662	5.192052	0.287870
C	2.704283	5.246945	2.722175
C	5.217732	6.960186	2.625390
C	4.141638	1.517223	1.849109
C	6.827379	2.455515	0.760455
C	6.631020	1.380154	3.569642
H	3.901706	6.029177	-0.114058
H	4.053223	4.258318	-0.092638
H	5.512632	5.274523	-0.133186
H	2.126338	6.003662	2.164669
H	2.561541	5.482156	3.790970
H	2.236694	4.267345	2.526511
H	6.240197	7.104790	2.234004
H	5.231195	7.150268	3.710696
H	4.595830	7.755222	2.180989
H	3.566963	2.046881	1.071547
H	3.476800	1.394741	2.721276
H	4.366515	0.507830	1.463894
H	6.337704	2.949589	-0.093269
H	7.079132	1.426844	0.450481
H	7.775165	2.987615	0.950009
H	6.826534	0.382629	3.141053
H	6.058607	1.217175	4.497198
H	7.609053	1.800463	3.857610
C	3.426795	1.024981	7.694766
C	4.396178	0.428939	8.663410
H	4.020222	0.574761	9.683825
H	4.496444	-0.643725	8.454415
H	5.370684	0.919581	8.553588
F	3.246988	2.375977	7.887803
F	3.845357	0.899541	6.386186
F	2.191074	0.461907	7.754696

06a_encountcmplx_dimer_parsep_pbe0.log

SCF (RPBE1PBE) = -3322.78616048
E(SCF)+ZPE(0 K)= -3322.277979
H(298 K)= -3322.233313
G(298 K)= -3322.354854
Lowest Frequency = 22.0056cm-1

C	-7.504919	0.876984	1.482050
C	-7.041101	-0.355174	0.798533
H	-6.660229	-1.056303	1.540839
H	-7.887239	-0.800688	0.275339
H	-6.261185	-0.089666	0.074805
F	-6.530948	1.442950	2.243974
F	-7.883829	1.834999	0.608151
F	-8.543893	0.681262	2.303161
K	-5.943558	3.221482	-1.190414
N	-4.852987	0.824896	-1.632658
Si	-3.429317	0.402211	-0.866100
Si	-5.562724	0.226665	-3.025474
C	-1.848876	0.854181	-1.800659
C	-3.313849	1.320792	0.791992
C	-3.253815	-1.433776	-0.441274
C	-6.942579	1.387027	-3.624425
C	-4.398832	0.030830	-4.503848
C	-6.405199	-1.456188	-2.820264
H	-1.825495	1.921183	-2.045856
H	-1.780801	0.300528	-2.742769

```
H       -0.950519    0.625282   -1.216014
H       -2.354227    1.161017    1.293212
H       -4.111793    0.974499    1.456790
H       -3.421766    2.394393    0.604487
H       -4.117410   -1.796599    0.126114
H       -2.355332   -1.629829    0.155062
H       -3.181393   -2.039120   -1.351346
H       -6.561893    2.367277   -3.937344
H       -7.718026    1.537284   -2.862881
H       -7.447775    0.962022   -4.498447
H       -3.867909    0.965566   -4.712830
H       -4.939977   -0.258056   -5.412043
H       -3.645002   -0.740226   -4.311735
H       -6.818526   -1.820044   -3.767770
H       -7.227656   -1.406258   -2.099489
H       -5.695673   -2.207311   -2.457283
K       -4.640889    3.465784    2.827078
N       -5.226554    5.066136    0.773534
Si       -3.750436    5.651264    0.221823
Si       -6.694804    5.775812    1.175913
C       -3.617617    7.511228   -0.054345
C       -2.332927    5.228431    1.413342
C       -3.236900    4.880307   -1.431617
C       -7.384820    5.067841    2.797254
C       -6.698267    7.643799    1.430917
C       -8.058254    5.443881   -0.100482
H       -3.771097    8.076978    0.868837
H       -4.351909    7.861084   -0.786397
H       -2.623012    7.764323   -0.437927
H       -1.395076    5.666328    1.054998
H       -2.144941    4.151856    1.502799
H       -2.506216    5.641070    2.415105
H       -3.179522    3.786630   -1.403584
H       -2.239758    5.231331   -1.718199
H       -3.913177    5.171776   -2.244625
H       -6.762575    5.324198    3.664291
H       -7.522819    3.980745    2.768612
H       -8.373829    5.492879    2.999039
H       -5.971322    7.945310    2.191456
H       -7.686376    7.977581    1.766532
H       -6.461250    8.185061    0.510899
H       -8.962574    6.006801    0.154892
H       -8.361299    4.391493   -0.145006
H       -7.759380    5.765461   -1.105122
```

06b_encountcmplx_dimer_parsep_wb97xd.log

```
SCF (RwB97XD) =       -3324.40192416
E(SCF)+ZPE(0 K)=      -3323.891987
H(298 K)=             -3323.847261
G(298 K)=             -3323.970539
Lowest Frequency = 13.6054cm-1
```

```
C       -6.756139    0.917344    1.454445
C       -6.702698   -0.496637    0.999297
H       -6.221509   -1.102026    1.765486
H       -7.718514   -0.851246    0.832161
H       -6.128182   -0.529008    0.072246
F       -5.513449    1.440151    1.655881
F       -7.351659    1.732595    0.565302
F       -7.406259    1.090062    2.619911
K       -5.223472    3.185704   -1.024420
N       -4.741618    0.635409   -1.737956
Si       -3.242685    0.164136   -1.189646
Si       -5.766644    0.219794   -2.981881
C       -2.012567   -0.374656   -2.523802
C       -2.385959    1.584086   -0.262200
C       -3.265159   -1.259562    0.061946
C       -6.989598    1.627520   -3.348082
C       -4.929875   -0.148214   -4.639995
C       -6.867332   -1.284329   -2.638836
H       -1.900908    0.392642   -3.295850
H       -2.342325   -1.293161   -3.018800
H       -1.021606   -0.567216   -2.099289
```

```
H       -1.419265    1.269046    0.143259
H       -2.985049    1.928788    0.587699
H       -2.190274    2.442806   -0.913596
H       -3.763014   -0.967226    0.991064
H       -2.250072   -1.579312    0.320336
H       -3.795251   -2.130493   -0.336666
H       -6.486062    2.529459   -3.715201
H       -7.581452    1.897007   -2.465846
H       -7.703042    1.332789   -4.124171
H       -4.289200    0.682134   -4.952655
H       -5.667425   -0.316061   -5.431943
H       -4.302236   -1.042319   -4.579619
H       -7.459030   -1.557671   -3.518822
H       -7.567427   -1.090792   -1.820727
H       -6.266661   -2.156140   -2.359692
K       -5.207374    3.714010    3.267038
N       -5.310523    5.178931    1.028523
Si       -3.799547    5.881350    0.832056
Si       -6.879677    5.751358    0.867939
C       -3.666163    7.713590    1.262336
C       -2.516144    5.017796    1.930508
C       -3.103115    5.730204   -0.926490
C       -7.994539    5.083147    2.249945
C       -7.095946    7.625045    0.907486
C       -7.735437    5.201067   -0.734252
H       -4.039637    7.914397    2.270803
H       -4.234401    8.338626    0.568838
H       -2.622165    8.040853    1.223641
H       -1.509047    5.370458    1.687928
H       -2.506769    3.931667    1.783401
H       -2.661938    5.229050    2.996841
H       -2.790950    4.711157   -1.179941
H       -2.209846    6.352699   -1.037979
H       -3.825778    6.068198   -1.676851
H       -7.700470    5.457573    3.237604
H       -8.016379    3.987866    2.279819
H       -9.029169    5.404106    2.095461
H       -6.670002    8.064065    1.813717
H       -8.157803    7.889305    0.876204
H       -6.612839    8.102145    0.050101
H       -8.715575    5.680392   -0.819865
H       -7.922987    4.122870   -0.776513
H       -7.166014    5.492153   -1.624033
```

06c_encountcmplx_dimer_parsep_b3lyp.log

```
SCF (RB3LYP) =        -3324.91837158
E(SCF)+ZPE(0 K)=      -3324.411385
H(298 K)=             -3324.366628
G(298 K)=             -3324.488664
Lowest Frequency = 22.2861cm-1
```

```
C       -6.995990    0.973145    1.553976
C       -6.805667   -0.340172    0.887779
H       -6.399876   -1.050395    1.606133
H       -7.771047   -0.691940    0.527203
H       -6.115186   -0.203744    0.049862
F       -5.808309    1.494323    2.015008
F       -7.491580    1.920319    0.715622
F       -7.817426    0.942806    2.621905
K       -5.357828    3.126503   -0.891526
N       -4.747700    0.654297   -1.678670
Si       -3.251622    0.149955   -1.134820
Si       -5.750565    0.170303   -2.924194
C       -1.927084   -0.039546   -2.479119
C       -2.548242    1.400428    0.112603
C       -3.271571   -1.506900   -0.207172
C       -7.301319    1.269535   -2.981529
C       -4.986623    0.291890   -4.656334
C       -6.385999   -1.612859   -2.783921
H       -1.847402    0.869690   -3.082668
H       -2.171090   -0.862310   -3.157834
H       -0.939417   -0.246361   -2.053409
H       -1.599997    1.055144    0.536266
```

```
H       -3.235425    1.550931    0.951514
H       -2.351164    2.373916   -0.346396
H       -3.880557   -1.448247    0.699747
H       -2.265187   -1.818922    0.091657
H       -3.692696   -2.300038   -0.832381
H       -7.062267    2.307160   -3.242355
H       -7.833025    1.275183   -2.024863
H       -8.007999    0.918196   -3.739856
H       -4.605086    1.300119   -4.845763
H       -5.714123    0.058109   -5.441036
H       -4.147300   -0.400613   -4.767598
H       -7.084104   -1.863773   -3.589519
H       -6.899954   -1.784451   -1.833864
H       -5.554349   -2.322368   -2.837461
K       -5.202189    3.873711    3.275616
N       -5.289494    5.229046    0.998782
Si      -3.746784    5.843563    0.738407
Si      -6.843377    5.851469    0.846635
C       -3.525161    7.699438    1.016744
C       -2.502239    5.008532    1.908412
C       -3.083117    5.519046   -1.012902
C       -7.942435    5.271324    2.286009
C       -6.994679    7.734248    0.814502
C       -7.748224    5.259033   -0.718027
H       -3.878312    7.998850    2.007439
H       -4.071820    8.290370    0.278214
H       -2.468296    7.975526    0.944014
H       -1.478303    5.296776    1.652597
H       -2.537195    3.915667    1.848539
H       -2.648983    5.309706    2.952883
H       -2.875832    4.463212   -1.214364
H       -2.135576    6.044145   -1.168522
H       -3.780254    5.883362   -1.774647
H       -7.604837    5.669933    3.250063
H       -7.999542    4.179511    2.356277
H       -8.970642    5.621006    2.153657
H       -6.542399    8.191992    1.697875
H       -8.046583    8.035702    0.785205
H       -6.505790    8.160016   -0.065629
H       -8.711109    5.769792   -0.815246
H       -7.976145    4.188469   -0.709556
H       -7.179759    5.486724   -1.626361
```

06d_encountcmplx_dimer_parsep_m062x.log

```
SCF (RM062X) =        -3324.14863331
E(SCF)+ZPE(0 K)=      -3323.638685
H(298 K)=             -3323.594223
G(298 K)=             -3323.715228
Lowest Frequency = 22.2418cm-1
```

```
C       -6.331103    1.040748    1.339373
C       -6.371642   -0.412351    1.039492
H       -5.845783   -0.950015    1.826486
H       -7.412568   -0.729519    1.001625
H       -5.884790   -0.558251    0.074620
F       -5.072359    1.538059    1.410310
F       -6.973740    1.792888    0.432818
F       -6.889193    1.353664    2.536221
K       -5.019860    3.232687   -1.086224
N       -4.739313    0.633247   -1.745134
Si      -3.192674    0.177653   -1.332399
Si      -5.873028    0.337410   -2.928207
C       -2.157367   -0.577432   -2.724323
C       -2.178217    1.665069   -0.725512
C       -3.081900   -1.069239    0.090821
C       -6.687854    1.947981   -3.525607
C       -5.229841   -0.506644   -4.493993
C       -7.336906   -0.720821   -2.357472
H       -2.092048    0.097427   -3.582459
H       -2.588684   -1.518759   -3.076242
H       -1.137189   -0.786964   -2.387689
H       -1.175810    1.359177   -0.410813
H       -2.637630    2.158756    0.139083
```

```
H       -2.048226    2.407788   -1.519880
H       -3.454455   -0.645204    1.027298
H       -2.044553   -1.375364    0.258963
H       -3.663115   -1.972110   -0.120480
H       -5.953654    2.658917   -3.920799
H       -7.257119    2.442836   -2.730476
H       -7.400631    1.748801   -4.331538
H       -4.417336    0.064574   -4.951717
H       -6.023928   -0.612835   -5.239632
H       -4.847460   -1.507623   -4.274847
H       -8.037519   -0.898437   -3.179458
H       -7.892943   -0.228958   -1.553814
H       -7.008183   -1.696505   -1.986509
K       -5.427436    3.649738    3.247325
N       -5.368937    5.066033    1.006492
Si      -3.871039    5.821376    1.029642
Si      -6.951769    5.567759    0.763916
C       -3.870944    7.672840    1.383862
C       -2.758685    5.056905    2.365397
C       -2.894368    5.613165   -0.580445
C       -8.098955    4.900338    2.120816
C       -7.238080    7.430508    0.727356
C       -7.705486    4.919966   -0.849348
H       -4.386282    7.900420    2.321004
H       -4.360618    8.240359    0.588819
H       -2.844704    8.042583    1.471080
H       -1.739110    5.442944    2.276475
H       -2.682794    3.967138    2.271688
H       -3.086810    5.304156    3.381931
H       -2.541886    4.588552   -0.736146
H       -1.998953    6.241596   -0.566624
H       -3.484037    5.917596   -1.451918
H       -7.851221    5.303429    3.109674
H       -8.097497    3.805727    2.172404
H       -9.133869    5.193114    1.921617
H       -6.887646    7.914992    1.642180
H       -8.304485    7.650846    0.620312
H       -6.719266    7.895785   -0.115147
H       -8.713338    5.324053   -0.982863
H       -7.809168    3.830785   -0.859089
H       -7.127852    5.226127   -1.728692
```

06e_encountcmplx_dimer_parsep_m06l.log

```
SCF (RM06L) =         -3324.50109413
E(SCF)+ZPE(0 K)=      -3323.990135
H(298 K)=             -3323.945360
G(298 K)=             -3324.066914
Lowest Frequency = 21.5627cm-1
```

```
C       -6.392376    1.032827    1.413837
C       -6.340112   -0.418241    1.137568
H       -5.783035   -0.919043    1.924631
H       -7.352405   -0.812117    1.100933
H       -5.845781   -0.556928    0.175699
F       -5.158129    1.606590    1.472938
F       -7.075731    1.729949    0.488751
F       -6.969636    1.337286    2.606275
K       -5.082044    3.209273   -1.045828
N       -4.714213    0.625572   -1.772236
Si      -3.161284    0.187168   -1.376581
Si      -5.865824    0.262349   -2.914175
C       -2.106695   -0.518788   -2.777285
C       -2.179985    1.682402   -0.740901
C       -3.016919   -1.079637    0.023554
C       -6.887886    1.797242   -3.366563
C       -5.227028   -0.404238   -4.563515
C       -7.170295   -0.988827   -2.351018
H       -2.057064    0.156756   -3.634030
H       -2.496024   -1.471404   -3.142881
H       -1.078716   -0.699884   -2.451534
H       -1.167525    1.404361   -0.437304
H       -2.638978    2.148681    0.137218
H       -2.068938    2.453093   -1.509133
```

H	-3.369865	-0.679470	0.976533
H	-1.978742	-1.387427	0.175680
H	-3.592565	-1.986533	-0.178913
H	-6.281340	2.608744	-3.780478
H	-7.442460	2.196550	-2.511938
H	-7.639748	1.563395	-4.124471
H	-4.509742	0.274617	-5.029984
H	-6.041468	-0.551236	-5.278098
H	-4.726876	-1.368243	-4.448953
H	-7.860713	-1.243776	-3.159674
H	-7.776838	-0.601682	-1.528986
H	-6.720843	-1.924139	-2.007231
K	-5.622764	3.724532	3.334312
N	-5.425984	5.107403	1.058298
Si	-3.893770	5.779677	1.071187
Si	-6.956657	5.696585	0.724658
C	-3.766588	7.599567	1.541019
C	-2.792286	4.870559	2.319055
C	-2.979120	5.630109	-0.581305
C	-8.218620	5.057930	1.986772
C	-7.158570	7.568907	0.709366
C	-7.641842	5.119762	-0.944959
H	-4.252728	7.811120	2.495766
H	-4.218789	8.256733	0.796477
H	-2.720781	7.901705	1.640064
H	-1.745425	5.159843	2.200975
H	-2.818034	3.782917	2.197589
H	-3.042973	5.100494	3.359669
H	-2.641894	4.614504	-0.804911
H	-2.074540	6.242915	-0.583822
H	-3.589803	5.975384	-1.420931
H	-8.036907	5.435713	2.997780
H	-8.257860	3.965274	2.032385
H	-9.229628	5.380251	1.727427
H	-6.851454	8.029098	1.650253
H	-8.201698	7.847749	0.539873
H	-6.571858	8.035714	-0.084612
H	-8.603668	5.595230	-1.151130
H	-7.831782	4.043956	-0.984891
H	-6.990002	5.380609	-1.784668

06f_encountcmplx_dimer_parsep_bp86.log

SCF (RB-P86) = -3324.96673746
E(SCF)+ZPE(0 K)= -3324.474398
H(298 K)= -3324.428402
G(298 K)= -3324.553635
Lowest Frequency = 25.5723cm-1

C	-7.269256	0.792488	1.707981
C	-7.203577	-0.140690	0.549059
H	-7.148274	-1.172738	0.917667
H	-8.114664	-0.015823	-0.050883
H	-6.320433	0.097945	-0.078591
F	-6.244399	0.616082	2.611053
F	-7.164545	2.119528	1.312550
F	-8.420822	0.711029	2.426869
K	-5.999950	3.206194	-1.180891
N	-4.859080	0.847403	-1.645037
Si	-3.412243	0.430749	-0.888316
Si	-5.548528	0.216733	-3.050705
C	-1.837361	0.908324	-1.841002
C	-3.309704	1.346542	0.781733
C	-3.221215	-1.415560	-0.468373
C	-7.000930	1.317150	-3.624994
C	-4.375616	0.100735	-4.543824
C	-6.295332	-1.523040	-2.847659
H	-1.835651	1.980360	-2.097880
H	-1.763988	0.344758	-2.785507
H	-0.923358	0.701042	-1.258028
H	-2.336863	1.218860	1.282227
H	-4.093484	0.956557	1.450087
H	-3.461274	2.423625	0.605939
H	-4.090326	-1.785944	0.100238

H	-2.317257	-1.612291	0.133729
H	-3.144049	-2.022612	-1.385699
H	-6.671081	2.323664	-3.938520
H	-7.776875	1.428197	-2.846787
H	-7.503226	0.873184	-4.500777
H	-3.886623	1.069603	-4.738285
H	-4.905094	-0.200612	-5.464142
H	-3.579318	-0.640566	-4.364236
H	-6.740276	-1.893092	-3.787410
H	-7.085099	-1.537916	-2.078824
H	-5.523239	-2.246565	-2.538183
K	-4.815032	3.336614	2.785985
N	-5.281496	5.038295	0.769120
Si	-3.770987	5.596744	0.245062
Si	-6.738496	5.792775	1.181800
C	-3.595808	7.461868	-0.049157
C	-2.385348	5.157639	1.483545
C	-3.241346	4.792145	-1.400180
C	-7.346708	5.263887	2.915309
C	-6.746601	7.688227	1.225286
C	-8.169688	5.309425	0.015171
H	-3.757650	8.043291	0.871740
H	-4.314070	7.822011	-0.802673
H	-2.582779	7.696488	-0.417936
H	-1.421514	5.580020	1.153018
H	-2.217746	4.071794	1.587697
H	-2.588516	5.579783	2.483513
H	-3.235077	3.689823	-1.370257
H	-2.215101	5.096615	-1.665090
H	-3.886808	5.111154	-2.237649
H	-6.636737	5.548801	3.712277
H	-7.558186	4.183422	2.994491
H	-8.295936	5.771603	3.155177
H	-6.003326	8.080569	1.937315
H	-7.736601	8.061062	1.538264
H	-6.524615	8.122839	0.238401
H	-9.092388	5.846672	0.291819
H	-8.419027	4.235311	0.060673
H	-7.953252	5.577302	-1.033697

07a_encountcmplx_dimer_sep_pbe0.log

SCF (RPBE1PBE) = -3322.76361971
E(SCF)+ZPE(0 K)= -3322.257437
H(298 K)= -3322.211608
G(298 K)= -3322.339531
Lowest Frequency = 14.2747cm-1

C	-5.491155	2.295830	-0.580814
C	-6.087514	1.004849	-0.170668
H	-5.974906	0.888283	0.913456
H	-7.145128	1.005641	-0.436144
H	-5.572084	0.202864	-0.707915
F	-4.167276	2.369327	-0.317973
F	-5.607196	2.542785	-1.903438
F	-6.049738	3.362820	0.042047
K	-3.008153	1.360008	-2.799767
N	-4.080981	-0.950555	-2.381159
Si	-3.197905	-1.816615	-1.254882
Si	-5.217551	-1.328578	-3.545732
C	-2.361581	-3.381286	-1.911277
C	-1.778316	-0.739640	-0.595754
C	-4.184040	-2.366696	0.258498
C	-5.347757	0.086501	-4.806565
C	-4.851470	-2.878385	-4.566473
C	-6.976058	-1.562554	-2.887051
H	-1.747430	-3.164730	-2.791854
H	-3.100906	-4.134380	-2.202137
H	-1.711307	-3.833615	-1.153721
H	-1.241158	-1.245632	0.213446
H	-2.131967	0.210534	-0.179274
H	-1.036486	-0.525707	-1.376156
H	-4.625194	-1.513974	0.779505
H	-3.561562	-2.909834	0.978196

H	-5.004002	-3.030319	-0.037329
H	-4.406086	0.242240	-5.347905
H	-5.640043	1.032820	-4.335000
H	-6.109271	-0.129525	-5.563876
H	-3.847209	-2.839534	-5.001192
H	-5.567505	-2.996465	-5.387763
H	-4.908196	-3.780800	-3.949148
H	-7.671166	-1.854679	-3.682353
H	-7.359482	-0.642185	-2.435295
H	-7.004539	-2.342025	-2.118022
K	-7.162565	3.409235	2.623895
N	-6.163140	1.124714	3.311562
Si	-7.383914	0.003766	3.524425
Si	-4.589098	1.330266	3.832519
C	-7.635217	-0.586616	5.303033
C	-9.046355	0.770154	3.009035
C	-7.228960	-1.566600	2.484744
C	-4.094064	3.159968	3.672528
C	-4.264745	0.878581	5.639079
C	-3.297058	0.390340	2.826374
H	-7.771248	0.258632	5.985888
H	-6.770810	-1.158514	5.655356
H	-8.515988	-1.232743	5.391381
H	-9.856751	0.037424	3.086753
H	-9.038119	1.110240	1.965587
H	-9.333445	1.613621	3.650084
H	-7.208965	-1.345373	1.412988
H	-8.069027	-2.246955	2.665827
H	-6.308049	-2.108191	2.723805
H	-4.699936	3.812088	4.314646
H	-4.165086	3.520717	2.638362
H	-3.052989	3.310677	3.977908
H	-4.950116	1.404761	6.311721
H	-3.241409	1.132060	5.938522
H	-4.399143	-0.195135	5.805457
H	-2.278145	0.610845	3.164358
H	-3.359379	0.654659	1.767213
H	-3.445311	-0.691249	2.903534

07b_encountcmplx_dimer_sep_wb97xd.log

SCF (RwB97XD) = -3324.38597701
E(SCF)+ZPE(0 K)= -3323.877299
H(298 K)= -3323.831923
G(298 K)= -3323.958380
Lowest Frequency = 11.1892cm-1

C	-5.496154	2.295804	-0.580447
C	-6.075617	1.005161	-0.128112
H	-5.955209	0.917768	0.955413
H	-7.134131	0.982640	-0.384676
H	-5.554838	0.198087	-0.646957
F	-4.172980	2.394596	-0.325185
F	-5.625931	2.503208	-1.907536
F	-6.067660	3.371563	0.017335
K	-2.897893	1.455126	-2.854817
N	-3.997015	-0.868237	-2.402742
Si	-3.161610	-1.742588	-1.249681
Si	-5.180540	-1.268007	-3.509312
C	-2.271682	-3.282157	-1.898749
C	-1.788755	-0.667985	-0.496733
C	-4.216690	-2.351680	0.194823
C	-5.222969	0.004332	-4.918327
C	-4.968062	-2.952431	-4.344731
C	-6.942160	-1.273734	-2.813178
H	-1.619296	-3.036191	-2.742679
H	-2.984635	-4.036844	-2.243689
H	-1.652754	-3.743902	-1.122040
H	-1.304408	-1.174746	0.343492
H	-2.167985	0.282666	-0.107978
H	-1.001603	-0.453796	-1.230098
H	-4.714637	-1.525237	0.704643
H	-3.620467	-2.888019	0.940135
H	-4.995372	-3.035422	-0.159377

H	-4.269952	0.045334	-5.457808
H	-5.456778	1.011323	-4.553266
H	-5.994303	-0.244608	-5.654051
H	-3.973018	-3.054535	-4.788312
H	-5.706633	-3.104792	-5.138701
H	-5.090800	-3.765430	-3.622323
H	-7.661340	-1.659967	-3.543136
H	-7.263621	-0.264998	-2.538256
H	-7.010234	-1.897484	-1.916119
K	-7.269490	3.455366	2.616886
N	-6.257679	1.181415	3.390317
Si	-7.409517	-0.017106	3.542295
Si	-4.645052	1.311516	3.800415
C	-7.639392	-0.675328	5.300718
C	-9.105968	0.655147	3.014989
C	-7.130276	-1.546137	2.465194
C	-4.062151	3.101996	3.545709
C	-4.238592	0.892577	5.599119
C	-3.480947	0.255250	2.752188
H	-7.821025	0.140662	6.007202
H	-6.744383	-1.207008	5.638173
H	-8.481582	-1.371840	5.368742
H	-9.877147	-0.118501	3.080619
H	-9.099723	1.001323	1.974521
H	-9.438443	1.482316	3.653223
H	-7.109947	-1.296241	1.400402
H	-7.922462	-2.287679	2.614073
H	-6.178261	-2.029084	2.704884
H	-4.599841	3.803778	4.194373
H	-4.183698	3.427475	2.505840
H	-2.999238	3.210145	3.782772
H	-4.861013	1.468576	6.290977
H	-3.190294	1.100998	5.837596
H	-4.417325	-0.167743	5.802345
H	-2.428526	0.447432	2.985845
H	-3.626214	0.455256	1.687965
H	-3.665431	-0.811219	2.910889

07c_encountcmplx_dimer_sep_b3lyp.log

SCF (RB3LYP) = -3324.89696619
E(SCF)+ZPE(0 K)= -3324.391309
H(298 K)= -3324.345709
G(298 K)= -3324.473200
Lowest Frequency = 8.1599cm-1

C	-5.491212	2.287332	-0.574157
C	-6.082077	0.992767	-0.155462
H	-5.961298	0.881888	0.925808
H	-7.140425	0.989911	-0.412129
H	-5.569563	0.192145	-0.692756
F	-4.157257	2.370446	-0.313186
F	-5.609536	2.532555	-1.906865
F	-6.055162	3.364883	0.048296
K	-3.028658	1.362080	-2.770414
N	-4.105708	-0.962826	-2.369470
Si	-3.220307	-1.836679	-1.250376
Si	-5.266614	-1.330582	-3.513573
C	-2.405197	-3.413021	-1.918937
C	-1.779089	-0.771786	-0.605678
C	-4.204865	-2.380504	0.271027
C	-5.404360	0.084446	-4.780043
C	-4.930471	-2.892591	-4.534346
C	-7.019614	-1.546175	-2.821185
H	-1.798617	-3.201206	-2.804706
H	-3.154775	-4.156327	-2.204490
H	-1.752273	-3.874801	-1.170578
H	-1.237120	-1.278439	0.198285
H	-2.116737	0.183147	-0.190717
H	-1.046768	-0.568453	-1.396592
H	-4.640480	-1.527624	0.791900
H	-3.584880	-2.926181	0.989520
H	-5.028056	-3.039555	-0.022045
H	-4.467452	0.232064	-5.329948

```
H        -5.681063    1.033242   -4.306726
H        -6.175476   -0.125326   -5.527910
H        -3.934074   -2.864147   -4.985166
H        -5.659777   -3.011154   -5.342672
H        -4.982555   -3.790066   -3.911328
H        -7.729047   -1.845670   -3.599800
H        -7.391078   -0.618827   -2.376781
H        -7.039685   -2.313565   -2.041319
K        -7.129009    3.436750    2.608663
N        -6.130177    1.139735    3.305866
Si        -7.355100    0.025325    3.529026
Si        -4.543241    1.336358    3.790408
C        -7.594575   -0.555641    5.316980
C        -9.021145    0.799517    3.019247
C        -7.213639   -1.553499    2.492731
C        -4.044918    3.170582    3.634506
C        -4.181741    0.865746    5.589838
C        -3.276602    0.400399    2.741995
H        -7.721214    0.294046    5.994658
H        -6.728931   -1.126160    5.665300
H        -8.475346   -1.198277    5.419254
H        -9.836933    0.075459    3.106170
H        -9.015849    1.134413    1.975141
H        -9.296025    1.649388    3.655693
H        -7.200357   -1.337623    1.421230
H        -8.054353   -2.229466    2.682111
H        -6.294444   -2.097713    2.726451
H        -4.642229    3.817755    4.287986
H        -4.133471    3.537465    2.604990
H        -3.000374    3.320969    3.923990
H        -4.848141    1.391519    6.280068
H        -3.151242    1.107095    5.871093
H        -4.323110   -0.206922    5.750434
H        -2.249444    0.618393    3.052758
H        -3.366898    0.671613    1.688459
H        -3.421516   -0.680464    2.814566
```

07d_encountcmplx_dimer_sep_m062x.log

```
SCF (RM062X) =        -3324.12937105
E(SCF)+ZPE(0 K)=      -3323.619925
H(298 K)=             -3323.575294
G(298 K)=             -3323.698328
Lowest Frequency = 10.1537cm-1

C        -5.591034    2.053872   -0.605270
C        -6.253764    0.794652   -0.183991
H        -6.113548    0.677826    0.894878
H        -7.314779    0.859743   -0.424807
H        -5.790774   -0.027300   -0.732310
F        -4.273960    2.083422   -0.309346
F        -5.665702    2.289041   -1.930084
F        -6.120425    3.150264   -0.007145
K        -2.979327    1.445512   -2.738986
N        -4.094830   -0.860275   -2.342983
Si        -3.245848   -1.711538   -1.182686
Si        -5.159101   -1.207468   -3.580307
C        -2.442540   -3.314132   -1.785921
C        -1.800940   -0.656799   -0.541938
C        -4.261848   -2.211687    0.331385
C        -5.007446    0.087317   -4.962723
C        -4.909967   -2.885900   -4.413342
C        -6.986878   -1.155973   -3.089512
H        -1.806750   -3.135375   -2.658068
H        -3.195770   -4.053847   -2.071382
H        -1.819746   -3.762246   -1.005240
H        -1.296775   -1.141695    0.299349
H        -2.120570    0.326319   -0.180892
H        -1.044011   -0.511578   -1.322061
H        -4.664572   -1.347786    0.864485
H        -3.665010   -2.789725    1.044043
H        -5.108868   -2.835697    0.026545
H        -3.997140    0.118552   -5.385603
H        -5.272207    1.092419   -4.613747
```

```
H        -5.688562   -0.137632   -5.788917
H        -3.875674   -3.018837   -4.743162
H        -5.558684   -2.999097   -5.287388
H        -5.143702   -3.702001   -3.723265
H        -7.627962   -1.480122   -3.915264
H        -7.296195   -0.144360   -2.811406
H        -7.187450   -1.811360   -2.236060
K        -7.193265    3.293877    2.552619
N        -6.156233    1.039178    3.312196
Si        -7.359575   -0.089408    3.570123
Si        -4.578308    1.303682    3.786632
C        -7.535752   -0.674319    5.358217
C        -9.042019    0.662801    3.104731
C        -7.219214   -1.663112    2.533233
C        -4.131722    3.135144    3.527830
C        -4.209242    0.944453    5.604412
C        -3.285304    0.348412    2.796395
H        -7.650910    0.171626    6.042017
H        -6.652508   -1.236527    5.674682
H        -8.404579   -1.327723    5.484049
H        -9.848989   -0.066238    3.222020
H        -9.068225    0.986958    2.057330
H        -9.300328    1.517060    3.741509
H        -7.245557   -1.449278    1.460486
H        -8.037664   -2.356270    2.752224
H        -6.279222   -2.183669    2.740202
H        -4.742077    3.803012    4.147123
H        -4.233080    3.438477    2.478646
H        -3.090514    3.327159    3.802781
H        -4.891233    1.488056    6.264591
H        -3.186719    1.231151    5.869145
H        -4.318356   -0.121838    5.822079
H        -2.265159    0.633833    3.072100
H        -3.407989    0.534659    1.726282
H        -3.385253   -0.729504    2.954563
```

07e_encountcmplx_dimer_sep_m06l.log

```
SCF (RM06L) =         -3324.48483880
E(SCF)+ZPE(0 K)=      -3323.974411
H(298 K)=             -3323.929435
G(298 K)=             -3324.052838
Lowest Frequency = 17.9679cm-1

C        -5.488149    2.185266   -0.588589
C        -6.102462    0.929093   -0.108876
H        -5.954995    0.841622    0.972052
H        -7.168023    0.943070   -0.329444
H        -5.633367    0.094103   -0.632344
F        -4.155378    2.245824   -0.354008
F        -5.625557    2.381452   -1.918271
F        -6.013186    3.290996    0.003499
K        -2.959203    1.475748   -2.828191
N        -4.042271   -0.851381   -2.408482
Si        -3.170950   -1.718006   -1.279713
Si        -5.211799   -1.248876   -3.528017
C        -2.290863   -3.258991   -1.931227
C        -1.778477   -0.635416   -0.579116
C        -4.172818   -2.322737    0.201685
C        -5.187776   -0.017862   -4.970655
C        -5.041642   -2.960325   -4.309064
C        -6.989197   -1.180711   -2.881043
H        -1.679155   -3.039605   -2.809651
H        -2.995218   -4.041932   -2.219555
H        -1.627906   -3.690782   -1.176357
H        -1.262927   -1.128277    0.248848
H        -2.131616    0.320624   -0.183549
H        -1.011094   -0.423877   -1.331182
H        -4.641907   -1.502510    0.746127
H        -3.561847   -2.875417    0.920788
H        -4.976015   -2.993472   -0.117314
H        -4.219272    0.006872   -5.478686
H        -5.424877    1.003266   -4.655751
H        -5.928956   -0.274007   -5.731826
```

H	-4.051226	-3.118744	-4.741428
H	-5.772872	-3.120388	-5.105862
H	-5.199000	-3.751356	-3.571534
H	-7.700041	-1.571586	-3.614191
H	-7.302354	-0.159649	-2.652281
H	-7.114692	-1.768261	-1.967522
K	-7.215142	3.385606	2.559055
N	-6.215924	1.138638	3.400755
Si	-7.392439	-0.030824	3.558980
Si	-4.617044	1.305477	3.838206
C	-7.678998	-0.636275	5.324441
C	-9.056010	0.671165	2.974885
C	-7.138622	-1.594677	2.530077
C	-4.081539	3.109684	3.588515
C	-4.222860	0.900386	5.639151
C	-3.410896	0.281783	2.808766
H	-7.840188	0.194215	6.015802
H	-6.819850	-1.197965	5.697885
H	-8.547675	-1.295602	5.400244
H	-9.853320	-0.073402	3.033130
H	-9.024076	0.995019	1.929234
H	-9.392123	1.519804	3.579139
H	-7.131133	-1.393237	1.455900
H	-7.931390	-2.326105	2.709924
H	-6.191970	-2.085596	2.768927
H	-4.647756	3.802834	4.218672
H	-4.186241	3.440080	2.549730
H	-3.030075	3.259835	3.844437
H	-4.877898	1.436541	6.329816
H	-3.193036	1.154940	5.903380
H	-4.348847	-0.165288	5.843376
H	-2.365844	0.495848	3.049614
H	-3.540799	0.470779	1.741138
H	-3.562564	-0.789274	2.962650

07f_encountcmplx_dimer_sep_bp86.log

SCF (RB-P86) = -3324.93988461
E(SCF)+ZPE(0 K)= -3324.449045
H(298 K)= -3324.402426
G(298 K)= -3324.531892
Lowest Frequency = 12.5056cm-1

C	-5.275656	2.485079	-0.561182
C	-5.877633	1.170382	-0.226214
H	-5.842328	1.024050	0.871582
H	-6.921254	1.162559	-0.566921
H	-5.311197	0.380340	-0.755348
F	-3.942789	2.565470	-0.238194
F	-5.336299	2.792738	-1.904397
F	-5.878192	3.550796	0.078081
K	-2.944937	1.405412	-2.824140
N	-4.017937	-0.878664	-2.299959
Si	-3.077322	-1.758954	-1.207448
Si	-5.307436	-1.350982	-3.280575
C	-2.210838	-3.286593	-1.941069
C	-1.663892	-0.651182	-0.557837
C	-4.032831	-2.379247	0.309760
C	-5.796702	0.089768	-4.429970
C	-4.940661	-2.825596	-4.426705
C	-6.891623	-1.824840	-2.337509
H	-1.639867	-3.023152	-2.847102
H	-2.941910	-4.060744	-2.225910
H	-1.508639	-3.743483	-1.222255
H	-1.099684	-1.153292	0.245459
H	-2.033382	0.295206	-0.128033
H	-0.930964	-0.415110	-1.350977
H	-4.551009	-1.551796	0.815186
H	-3.377519	-2.869137	1.049697
H	-4.800106	-3.111945	0.009268
H	-4.977354	0.362020	-5.119065
H	-6.079815	0.990225	-3.858842
H	-6.662343	-0.173618	-5.061002
H	-4.018506	-2.656886	-5.007510
H	-5.761057	-3.006046	-5.142698
H	-4.798899	-3.752632	-3.847328
H	-7.701855	-2.129564	-3.022318
H	-7.268293	-0.986838	-1.729901
H	-6.701783	-2.667263	-1.652403
K	-7.162532	3.443758	2.572803
N	-6.195931	1.111191	3.135734
Si	-7.438059	-0.022043	3.241172
Si	-4.640231	1.266943	3.762587
C	-7.810257	-0.651566	4.996498
C	-9.069245	0.769656	2.630337
C	-7.208286	-1.574268	2.171076
C	-4.119420	3.107594	3.723250
C	-4.430052	0.718898	5.571424
C	-3.298601	0.356420	2.776676
H	-7.991369	0.186221	5.690159
H	-6.966119	-1.235637	5.397675
H	-8.701588	-1.302170	5.016252
H	-9.886030	0.028703	2.613494
H	-8.983835	1.158267	1.599731
H	-9.414759	1.589006	3.286978
H	-7.085052	-1.324833	1.105001
H	-8.072306	-2.256004	2.254272
H	-6.312053	-2.135974	2.480423
H	-4.755153	3.738894	4.370299
H	-4.134674	3.524391	2.700338
H	-3.087304	3.234582	4.090885
H	-5.156149	1.223070	6.230286
H	-3.418676	0.945481	5.950820
H	-4.585983	-0.367158	5.677371
H	-2.286454	0.565541	3.163340
H	-3.318870	0.656527	1.718065
H	-3.449849	-0.733811	2.811932

08a_TS-H_monomer_pbe0.log

SCF (RPBE1PBE) = -1850.03432668
E(SCF)+ZPE(0 K)= -1849.761112
H(298 K)= -1849.735920
G(298 K)= -1849.816162
Lowest Frequency = -1207.1107cm-1

C	-5.161790	2.481316	-0.875791
C	-5.117466	1.084459	-0.497225
H	-4.410947	0.972977	0.329721
H	-6.113464	0.783873	-0.160906
H	-4.710121	0.034680	-1.500248
F	-3.947363	2.952835	-1.322719
F	-6.021654	2.726282	-1.922967
F	-5.534485	3.406956	0.062956
K	-4.236623	1.418523	-3.668240
N	-4.376653	-0.892255	-2.276921
Si	-2.803711	-1.424964	-1.861362
Si	-5.769218	-1.773664	-2.738934
C	-2.155291	-2.812700	-2.945975
C	-1.618975	0.030274	-2.056073
C	-2.692039	-2.001606	-0.077002
C	-7.018946	-0.556693	-3.455671
C	-5.449085	-3.061937	-4.066801
C	-6.590091	-2.641469	-1.288886
H	-2.228400	-2.569375	-4.010047
H	-2.693064	-3.749550	-2.777036
H	-1.099140	-2.994903	-2.718139
H	-0.627674	-0.224806	-1.667330
H	-1.960727	0.911207	-1.502006
H	-1.473122	0.309262	-3.107048
H	-3.002610	-1.211055	0.613016
H	-1.672029	-2.298257	0.189146
H	-3.346310	-2.862758	0.093204
H	-6.703614	-0.165674	-4.431061
H	-7.201812	0.287758	-2.783593
H	-7.982061	-1.049977	-3.622866
H	-4.934485	-2.633063	-4.932164
H	-6.402159	-3.472737	-4.418325

```
H       -4.847540   -3.896573   -3.697999
H       -7.521007   -3.135661   -1.586281
H       -6.826044   -1.931464   -0.489945
H       -5.928019   -3.404511   -0.866748
```

08b_TS-H_monomer_wb97xd.log

```
SCF (RwB97XD) =       -1850.97534242
E(SCF)+ZPE(0 K)=      -1850.700718
H(298 K)=             -1850.675839
G(298 K)=             -1850.754799
Lowest Frequency = -1312.9714cm-1
```

```
C       -5.071616    2.577187   -1.156263
C       -5.142220    1.231175   -0.618295
H       -4.485844    1.167853    0.252843
H       -6.171595    1.036211   -0.308492
H       -4.709689    0.118147   -1.535260
F       -3.823616    2.898698   -1.640090
F       -5.906914    2.761698   -2.238298
F       -5.376020    3.631829   -0.337060
K       -4.274008    1.279962   -3.982139
N       -4.381912   -0.844673   -2.263608
Si       -2.813816   -1.426295   -1.904560
Si       -5.782002   -1.777800   -2.578684
C       -2.003434   -2.252310   -3.388389
C       -1.726029    0.030252   -1.421539
C       -2.782736   -2.661000   -0.487666
C       -7.004409   -0.723246   -3.552670
C       -5.432008   -3.320159   -3.591247
C       -6.641665   -2.314038   -0.997128
H       -1.996272   -1.593667   -4.262822
H       -2.519593   -3.172844   -3.671820
H       -0.963824   -2.512009   -3.164662
H       -0.728616   -0.319812   -1.138921
H       -2.139777    0.577016   -0.570922
H       -1.589777    0.744606   -2.240098
H       -3.220632   -2.227474    0.416198
H       -1.761441   -2.973713   -0.249581
H       -3.354691   -3.560284   -0.735263
H       -6.660236   -0.526280   -4.574532
H       -7.191989    0.233601   -3.055010
H       -7.969168   -1.230475   -3.645316
H       -4.944730   -3.079724   -4.540067
H       -6.366667   -3.842136   -3.819336
H       -4.788599   -4.020572   -3.051676
H       -7.556958   -2.876928   -1.203666
H       -6.910348   -1.448007   -0.385807
H       -5.984886   -2.950040   -0.396419
```

08c_TS-H_monomer_b3lyp.log

```
SCF (RB3LYP) =        -1851.29132056
E(SCF)+ZPE(0 K)=      -1851.019025
H(298 K)=             -1850.993916
G(298 K)=             -1851.072968
Lowest Frequency = -1341.7545cm-1
```

```
C       -5.037896    2.571942   -1.382997
C       -5.115824    1.340436   -0.633184
H       -4.427620    1.382346    0.211835
H       -6.138377    1.194002   -0.282335
H       -4.715431    0.157060   -1.475812
F       -3.785938    2.788880   -1.947614
F       -5.900821    2.596486   -2.472976
F       -5.302722    3.776373   -0.749475
K       -4.260257    1.065601   -4.133260
N       -4.399692   -0.839116   -2.185235
Si       -2.822036   -1.427403   -1.860446
Si       -5.799703   -1.779883   -2.499113
C       -1.967178   -2.024285   -3.434876
C       -1.765857   -0.030735   -1.167913
C       -2.779535   -2.844933   -0.620672
C       -7.041015   -0.737022   -3.472505
C       -5.443391   -3.310978   -3.535126
C       -6.653749   -2.330817   -0.915602
H       -1.862179   -1.221524   -4.173065
H       -2.515600   -2.840547   -3.910331
H       -0.957941   -2.387160   -3.215723
H       -0.744694   -0.382699   -0.993107
H       -2.156213    0.339790   -0.217584
H       -1.701796    0.821780   -1.850362
H       -3.226038   -2.541146    0.330296
H       -1.752441   -3.164858   -0.419483
H       -3.331033   -3.716392   -0.982983
H       -6.720507   -0.554829   -4.505096
H       -7.229871    0.226864   -2.992169
H       -8.002107   -1.254780   -3.543432
H       -4.996007   -3.048828   -4.497604
H       -6.372215   -3.852903   -3.739949
H       -4.765783   -4.002815   -3.029150
H       -7.571443   -2.889593   -1.122593
H       -6.918080   -1.470044   -0.295295
H       -5.996749   -2.973523   -0.323326
```

08d_TS-H_monomer_m062x.log

```
SCF (RM062X) =        -1850.83265952
E(SCF)+ZPE(0 K)=      -1850.558079
H(298 K)=             -1850.533171
G(298 K)=             -1850.611926
Lowest Frequency = -1256.4044cm-1
```

```
C       -5.106992    2.542777   -0.999689
C       -5.122852    1.155071   -0.557957
H       -4.435596    1.060109    0.286258
H       -6.135504    0.923553   -0.220133
H       -4.709374    0.083175   -1.499913
F       -3.890349    2.930080   -1.506493
F       -5.983851    2.779597   -2.031767
F       -5.403345    3.515066   -0.094750
K       -4.380242    1.361020   -3.741917
N       -4.377180   -0.889835   -2.257234
Si       -2.795311   -1.435564   -1.909682
Si       -5.783221   -1.784229   -2.637437
C       -2.042026   -2.450175   -3.301454
C       -1.695945    0.074412   -1.668982
C       -2.701126   -2.470074   -0.343565
C       -6.946718   -0.665282   -3.616235
C       -5.442659   -3.308416   -3.678008
C       -6.713896   -2.338858   -1.103118
H       -2.107476   -1.925051   -4.258781
H       -2.534822   -3.418026   -3.416885
H       -0.983565   -2.640804   -3.099427
H       -0.697971   -0.226771   -1.338048
H       -2.101916    0.754258   -0.915531
H       -1.557383    0.640503   -2.596565
H       -3.122436   -1.927019    0.507263
H       -1.669766   -2.737137   -0.096285
H       -3.266357   -3.399968   -0.456226
H       -6.566599   -0.449657   -4.621188
H       -7.126209    0.280343   -3.092143
H       -7.922832   -1.138993   -3.751632
H       -4.874413   -3.066223   -4.579698
H       -6.385050   -3.769252   -3.988943
H       -4.880301   -4.059428   -3.116840
H       -7.618281   -2.897427   -1.360390
H       -7.010922   -1.482696   -0.491763
H       -6.085253   -2.984329   -0.483107
```

08e_TS-H_monomer_m06l.log

```
SCF (RM06L) =         -1851.04287266
E(SCF)+ZPE(0 K)=      -1850.767962
H(298 K)=             -1850.743054
G(298 K)=             -1850.821324
Lowest Frequency = -1145.4208cm-1
```

C	-5.094479	2.601069	-1.083431
C	-5.129637	1.229408	-0.629440
H	-4.463450	1.132040	0.229770
H	-6.146905	1.006467	-0.303577
H	-4.701975	0.106673	-1.547079
F	-3.864024	2.970517	-1.596412
F	-5.970402	2.837765	-2.128345
F	-5.378815	3.609921	-0.200558
K	-4.381404	1.379598	-3.812708
N	-4.376006	-0.873393	-2.266500
Si	-2.805334	-1.441573	-1.908221
Si	-5.777612	-1.790515	-2.604189
C	-2.015672	-2.351644	-3.348361
C	-1.709177	0.036433	-1.529401
C	-2.755140	-2.589002	-0.424094
C	-6.973085	-0.705213	-3.574180
C	-5.431892	-3.322052	-3.626370
C	-6.668464	-2.339112	-1.047464
H	-2.056711	-1.769481	-4.271891
H	-2.493606	-3.311065	-3.550302
H	-0.961617	-2.557371	-3.146269
H	-0.700605	-0.288451	-1.264816
H	-2.083575	0.628094	-0.692698
H	-1.596704	0.712846	-2.381034
H	-3.212597	-2.125214	0.452888
H	-1.734540	-2.866723	-0.151402
H	-3.298692	-3.516545	-0.617757
H	-6.613626	-0.480822	-4.582838
H	-7.172102	0.240790	-3.062631
H	-7.941434	-1.192541	-3.704054
H	-4.920952	-3.090188	-4.562535
H	-6.362782	-3.832992	-3.883646
H	-4.813996	-4.042000	-3.086050
H	-7.584560	-2.889795	-1.272412
H	-6.946902	-1.489475	-0.421381
H	-6.039261	-2.991704	-0.438876

08f_TS-H_monomer_bp86.log

SCF (RB-P86) = -1851.31611257
E(SCF)+ZPE(0 K)= -1851.051902
H(298 K)= -1851.026115
G(298 K)= -1851.107070
Lowest Frequency = -1046.7704cm-1

C	-5.039255	2.610432	-1.395195
C	-5.116169	1.376399	-0.653175
H	-4.418566	1.404592	0.195524
H	-6.145240	1.217547	-0.300923
H	-4.713267	0.176928	-1.495782
F	-3.772617	2.840859	-1.963892
F	-5.914773	2.648428	-2.496231
F	-5.306524	3.837197	-0.752844
K	-4.259476	1.070709	-4.133026
N	-4.399696	-0.823307	-2.194741
Si	-2.818873	-1.434774	-1.857936
Si	-5.802162	-1.787077	-2.499909
C	-1.956070	-2.027867	-3.437243
C	-1.760636	-0.042457	-1.142987
C	-2.800786	-2.866961	-0.624220
C	-7.066356	-0.745010	-3.456464
C	-5.430008	-3.310860	-3.552409
C	-6.636001	-2.356786	-0.904268
H	-1.830176	-1.213603	-4.171780
H	-2.516804	-2.836943	-3.930265
H	-0.946840	-2.412435	-3.212136
H	-0.736591	-0.403408	-0.951059
H	-2.166615	0.330737	-0.190355
H	-1.679133	0.818155	-1.826730
H	-3.252030	-2.562843	0.334006
H	-1.771308	-3.204693	-0.417888
H	-3.365069	-3.735674	-0.999317
H	-6.764072	-0.558830	-4.502459
H	-7.253540	0.224815	-2.967854
H	-8.034349	-1.270049	-3.511466
H	-4.991432	-3.033498	-4.524328
H	-6.358174	-3.871618	-3.755348
H	-4.731973	-4.000317	-3.052249
H	-7.562268	-2.920739	-1.103816
H	-6.896120	-1.495139	-0.268553
H	-5.964767	-3.007161	-0.320947

09a_TS-H_dimer_intact_pbe0.log

SCF (RPBE1PBE) = -3322.76087937
E(SCF)+ZPE(0 K)= -3322.258057
H(298 K)= -3322.214146
G(298 K)= -3322.333135
Lowest Frequency = -1087.5171cm-1

C	-5.336333	2.273292	-1.203123
C	-5.132481	0.853159	-0.990914
H	-4.448274	0.733873	-0.145939
H	-6.096898	0.411641	-0.723718
H	-4.586568	-0.149264	-2.033075
F	-4.176750	2.940877	-1.516131
F	-6.181186	2.544377	-2.252036
F	-5.861132	3.014035	-0.173888
K	-4.153057	1.546688	-3.968285
N	-4.176160	-1.097687	-2.717452
Si	-2.690517	-1.517854	-1.944472
Si	-5.590020	-2.069337	-2.915381
C	-1.697204	-2.836918	-2.861790
C	-1.611376	0.016331	-1.886337
C	-2.906720	-2.159881	-0.195123
C	-6.938509	-0.996402	-3.654872
C	-5.357305	-3.528076	-4.094029
C	-6.226356	-2.806815	-1.311872
H	-1.148432	-2.459542	-3.731647
H	-2.292289	-3.705689	-3.159295
H	-0.926985	-3.209399	-2.177658
H	-0.669849	-0.196325	-1.369734
H	-2.098947	0.837108	-1.352278
H	-1.347035	0.364986	-2.890189
H	-3.542695	-1.498563	0.399672
H	-1.936590	-2.234362	0.307439
H	-3.360425	-3.155054	-0.187308
H	-6.654886	-0.612737	-4.640059
H	-7.177065	-0.143675	-3.013209
H	-7.858150	-1.574769	-3.790683
H	-5.393049	-3.258747	-5.155391
H	-6.200094	-4.211883	-3.945169
H	-4.450613	-4.108722	-3.898474
H	-7.215801	-3.251439	-1.462313
H	-6.314830	-2.049117	-0.528539
H	-5.563047	-3.593802	-0.941722
N	-3.241663	0.710109	-6.406097
K	-3.131718	-1.790205	-5.489329
Si	-1.664434	1.277734	-6.470482
Si	-4.595495	0.803879	-7.392321
C	-1.062005	1.929510	-8.132763
C	-1.338735	2.680762	-5.235623
C	-0.428464	-0.079142	-5.988771
C	-4.336041	1.477516	-9.132824
C	-5.391085	-0.902733	-7.627019
C	-5.971235	1.875725	-6.644937
H	-0.007830	2.220581	-8.067925
H	-1.628494	2.808494	-8.453610
H	-1.149446	1.172393	-8.917855
H	-0.326075	3.077637	-5.366560
H	-1.401276	2.364643	-4.187564
H	-2.030187	3.517924	-5.386115
H	-0.454167	-0.925295	-6.686464
H	-0.585042	-0.458027	-4.971809
H	0.595092	0.310130	-6.009841
H	-3.597402	0.891496	-9.687665
H	-3.992925	2.516175	-9.116916
H	-5.275234	1.448067	-9.696066

```
H       -4.709633   -1.607236   -8.119546
H       -6.278996   -0.829288   -8.263981
H       -5.733747   -1.346874   -6.684918
H       -6.806835    1.967836   -7.347299
H       -5.620765    2.893214   -6.435227
H       -6.400111    1.460370   -5.725309
```

09b_TS-H_dimer_intact_wb97xd.log

```
SCF (RwB97XD) =       -3324.37559990
E(SCF)+ZPE(0 K)=      -3323.869901
H(298 K)=             -3323.826520
G(298 K)=             -3323.945415
Lowest Frequency = -1248.9204cm-1
```

```
C       -5.200893    2.480428   -1.188271
C       -4.883742    1.098140   -0.859852
H       -4.036875    1.108965   -0.168965
H       -5.745476    0.664455   -0.345528
H       -4.561818    0.021165   -1.901620
F       -4.138261    3.148837   -1.749975
F       -6.208239    2.601706   -2.112652
F       -5.599332    3.308191   -0.170827
K       -4.301144    1.532038   -4.057237
N       -4.270432   -0.970234   -2.593258
Si       -2.805788   -1.500876   -1.861877
Si       -5.714203   -1.879534   -2.812001
C       -2.071820   -3.044518   -2.678832
C       -1.542615   -0.123289   -2.030774
C       -2.990204   -1.916746   -0.041635
C       -7.185353   -0.717751   -2.888407
C       -5.735071   -2.861381   -4.424813
C       -6.039987   -3.122716   -1.443449
H       -1.349846   -2.821993   -3.471931
H       -2.829049   -3.733966   -3.064995
H       -1.504852   -3.599939   -1.926269
H       -0.593064   -0.397642   -1.562136
H       -1.894600    0.794310   -1.551155
H       -1.326383    0.108530   -3.078283
H       -3.514743   -1.126413    0.500235
H       -2.008156   -2.048814    0.422763
H       -3.549387   -2.845300    0.097668
H       -7.088937    0.050439   -3.660461
H       -7.345804   -0.210562   -1.935029
H       -8.091331   -1.286506   -3.118390
H       -5.711735   -2.219641   -5.312332
H       -6.676517   -3.415589   -4.484018
H       -4.940502   -3.612873   -4.486076
H       -6.998661   -3.626758   -1.598895
H       -6.074596   -2.630295   -0.467869
H       -5.264556   -3.892995   -1.401533
N       -3.212985    0.697495   -6.424837
K       -2.955773   -1.812297   -5.440036
Si       -1.653600    1.309838   -6.493317
Si       -4.469432    0.553502   -7.524937
C       -1.133483    2.107356   -8.120424
C       -1.344022    2.616524   -5.156560
C       -0.345103   -0.027850   -6.160980
C       -4.465945    1.755434   -8.976500
C       -4.577496   -1.178722   -8.298660
C       -6.145784    0.777716   -6.671037
H       -0.099105    2.460594   -8.062058
H       -1.763493    2.965114   -8.369944
H       -1.194678    1.398126   -8.950975
H       -0.329491    3.019042   -5.237229
H       -1.431910    2.215717   -4.140424
H       -2.031386    3.463925   -5.253348
H       -0.403781   -0.847041   -6.886891
H       -0.405895   -0.450397   -5.151037
H        0.660881    0.394256   -6.246657
H       -3.620624    1.582460   -9.647220
H       -4.411028    2.792589   -8.633263
H       -5.383127    1.648261   -9.564353
H       -3.616833   -1.491286   -8.721378
H       -5.308263   -1.191597   -9.113437
H       -4.908419   -1.949200   -7.591700
H       -6.964636    0.587835   -7.371614
H       -6.293100    1.794539   -6.289800
H       -6.279099    0.072899   -5.842536
```

09c_TS-H_dimer_intact_b3lyp.log

```
SCF (RB3LYP) =        -3324.89702003
E(SCF)+ZPE(0 K)=      -3324.394712
H(298 K)=             -3324.350993
G(298 K)=             -3324.469607
Lowest Frequency = -1224.8237cm-1
```

```
C       -5.176262    2.487377   -1.221862
C       -4.891434    1.113348   -0.862849
H       -4.053964    1.107669   -0.163411
H       -5.770472    0.687226   -0.376207
H       -4.553861    0.034347   -1.906691
F       -4.087486    3.133375   -1.786679
F       -6.177392    2.611356   -2.171205
F       -5.581067    3.364490   -0.226104
K       -4.327811    1.533826   -4.075653
N       -4.256235   -0.960283   -2.598973
Si       -2.788580   -1.494323   -1.862184
Si       -5.701296   -1.878316   -2.818049
C       -2.041844   -3.024122   -2.700729
C       -1.523360   -0.110867   -1.994729
C       -2.980387   -1.941780   -0.046041
C       -7.183943   -0.724493   -2.887801
C       -5.718768   -2.851279   -4.440941
C       -6.023632   -3.134633   -1.456021
H       -1.357341   -2.789335   -3.522316
H       -2.792836   -3.736409   -3.053508
H       -1.430684   -3.557181   -1.966788
H       -0.573358   -0.407731   -1.540961
H       -1.866049    0.790716   -1.481386
H       -1.311010    0.157638   -3.032353
H       -3.519861   -1.167975    0.504019
H       -1.998705   -2.062914    0.422121
H       -3.523788   -2.880622    0.079247
H       -7.098168    0.046053   -3.657544
H       -7.348995   -0.221628   -1.933686
H       -8.084397   -1.301515   -3.118307
H       -5.697779   -2.204623   -5.323208
H       -6.657581   -3.409039   -4.504574
H       -4.921722   -3.598639   -4.508839
H       -6.979722   -3.640898   -1.620250
H       -6.065978   -2.651379   -0.476898
H       -5.246917   -3.902612   -1.416123
N       -3.234389    0.697230   -6.423032
K       -2.982925   -1.787138   -5.417543
Si       -1.666861    1.297487   -6.477477
Si       -4.491808    0.558409   -7.526956
C       -1.113932    2.066830   -8.111067
C       -1.373684    2.622114   -5.149892
C       -0.377539   -0.054819   -6.107139
C       -4.480552    1.766021   -8.978432
C       -4.597844   -1.176675   -8.302499
C       -6.168771    0.796448   -6.669492
H       -0.074800    2.404688   -8.046358
H       -1.726648    2.930788   -8.379238
H       -1.176962    1.347513   -8.932253
H       -0.354430    3.016176   -5.208962
H       -1.491104    2.237232   -4.131454
H       -2.051909    3.472710   -5.276363
H       -0.429826   -0.875954   -6.831142
H       -0.465204   -0.472396   -5.098174
H        0.635614    0.353334   -6.171786
H       -3.634622    1.592597   -9.647348
H       -4.422381    2.801275   -8.630766
H       -5.396364    1.665844   -9.569649
H       -3.642956   -1.470883   -8.749939
H       -5.349471   -1.203278   -9.097748
```

H	-4.893321	-1.954072	-7.588468
H	-6.990942	0.620969	-7.369731
H	-6.301645	1.814581	-6.286490
H	-6.313622	0.093852	-5.842950

09d_TS-H_dimer_intact_m062x.log

SCF (RM062X) = -3324.12077243
E(SCF)+ZPE(0 K)= -3323.615731
H(298 K)= -3323.572310
G(298 K)= -3323.690140
Lowest Frequency = -1092.9391cm-1

C	-5.183103	2.502025	-1.279062
C	-4.843204	1.125474	-0.940355
H	-3.994073	1.159764	-0.253089
H	-5.697756	0.689492	-0.416152
H	-4.519091	0.040496	-1.981323
F	-4.134153	3.181892	-1.844297
F	-6.186876	2.597934	-2.208247
F	-5.598538	3.326616	-0.274003
K	-4.342193	1.554618	-4.065316
N	-4.227742	-0.959859	-2.664871
Si	-2.781871	-1.491783	-1.887063
Si	-5.683077	-1.864382	-2.856708
C	-2.022654	-3.020141	-2.708823
C	-1.521170	-0.108821	-2.001851
C	-3.023150	-1.932305	-0.079755
C	-7.156527	-0.702417	-2.863453
C	-5.743027	-2.811513	-4.489461
C	-5.971548	-3.136848	-1.508089
H	-1.313830	-2.788912	-3.510957
H	-2.769948	-3.727980	-3.082544
H	-1.437848	-3.558398	-1.957447
H	-0.577100	-0.394723	-1.529782
H	-1.880021	0.797553	-1.506483
H	-1.296857	0.146335	-3.041885
H	-3.611672	-1.174458	0.442873
H	-2.053958	-2.010790	0.421412
H	-3.532679	-2.891567	0.034427
H	-7.063367	0.119450	-3.578334
H	-7.323843	-0.263923	-1.877651
H	-8.057905	-1.259196	-3.135773
H	-5.722396	-2.151658	-5.363387
H	-6.695035	-3.347677	-4.542218
H	-4.963683	-3.575797	-4.581773
H	-6.931297	-3.638999	-1.660532
H	-5.992722	-2.665845	-0.521835
H	-5.193943	-3.905697	-1.497365
N	-3.249777	0.691939	-6.375599
K	-3.007906	-1.767077	-5.339545
Si	-1.681658	1.285795	-6.433511
Si	-4.500168	0.539111	-7.483764
C	-1.145704	2.059500	-8.065735
C	-1.379346	2.605024	-5.108985
C	-0.396943	-0.071980	-6.091903
C	-4.475301	1.729940	-8.943260
C	-4.596395	-1.194356	-8.251978
C	-6.173872	0.787639	-6.632920
H	-0.111780	2.411766	-8.002684
H	-1.772631	2.913796	-8.333137
H	-1.200164	1.336522	-8.884533
H	-0.356852	2.989442	-5.170301
H	-1.502293	2.227455	-4.088059
H	-2.050463	3.460264	-5.242246
H	-0.467836	-0.882312	-6.826282
H	-0.467927	-0.504870	-5.087672
H	0.616237	0.333455	-6.169510
H	-3.623504	1.547280	-9.602803
H	-4.419584	2.768668	-8.606100
H	-5.386198	1.622112	-9.539821
H	-3.637333	-1.477424	-8.698037
H	-5.344537	-1.219918	-9.050194
H	-4.887521	-1.979184	-7.544939
H	-6.997899	0.593599	-7.325695
H	-6.307805	1.812485	-6.268585
H	-6.308564	0.099075	-5.790864

09e_TS-H_dimer_intact_m06l.log

SCF (RM06L) = -3324.46848929
E(SCF)+ZPE(0 K)= -3323.962696
H(298 K)= -3323.919023
G(298 K)= -3324.036616
Lowest Frequency = -1131.6395cm-1

C	-5.053710	2.531366	-1.233092
C	-4.804361	1.139725	-0.924167
H	-3.952379	1.102182	-0.242196
H	-5.675351	0.745932	-0.395431
H	-4.511637	0.025741	-1.975719
F	-3.977763	3.140204	-1.850129
F	-6.096567	2.712715	-2.117570
F	-5.358102	3.395859	-0.209718
K	-4.429971	1.582147	-4.060214
N	-4.239921	-0.974988	-2.649685
Si	-2.792959	-1.519191	-1.886984
Si	-5.701245	-1.863315	-2.847598
C	-2.051017	-3.047935	-2.715807
C	-1.532970	-0.142007	-2.021842
C	-3.012387	-1.963057	-0.080601
C	-7.152116	-0.678174	-2.900389
C	-5.748001	-2.836386	-4.461701
C	-6.033257	-3.100940	-1.481891
H	-1.349879	-2.824808	-3.524791
H	-2.797928	-3.754571	-3.086603
H	-1.459016	-3.597422	-1.980962
H	-0.583864	-0.409399	-1.552980
H	-1.882457	0.775037	-1.543264
H	-1.309383	0.103587	-3.062935
H	-3.587816	-1.213153	0.463708
H	-2.042737	-2.053033	0.414305
H	-3.523766	-2.918364	0.044300
H	-7.055561	0.115296	-3.644136
H	-7.319045	-0.195479	-1.936985
H	-8.067326	-1.219593	-3.149763
H	-5.724118	-2.199450	-5.350601
H	-6.692541	-3.381512	-4.522332
H	-4.966663	-3.596936	-4.545485
H	-6.995108	-3.597309	-1.628722
H	-6.063248	-2.618408	-0.502986
H	-5.272320	-3.882934	-1.438027
N	-3.279983	0.731023	-6.370779
K	-2.993711	-1.779285	-5.385552
Si	-1.720369	1.336261	-6.417971
Si	-4.491972	0.523043	-7.504853
C	-1.179803	2.156978	-8.023536
C	-1.424435	2.619363	-5.059816
C	-0.419779	-0.014672	-6.115073
C	-4.479392	1.677346	-8.990857
C	-4.524188	-1.230383	-8.234586
C	-6.193214	0.725467	-6.703388
H	-0.154157	2.526709	-7.947645
H	-1.809258	3.008248	-8.288424
H	-1.206807	1.461033	-8.864785
H	-0.405310	3.011132	-5.101689
H	-1.547871	2.222871	-4.048015
H	-2.089938	3.481485	-5.161087
H	-0.477010	-0.812376	-6.862624
H	-0.473426	-0.477862	-5.124893
H	0.590838	0.394421	-6.186599
H	-3.613984	1.518693	-9.636091
H	-4.470098	2.726983	-8.690285
H	-5.369778	1.527062	-9.606538
H	-3.544487	-1.530021	-8.618440
H	-5.219503	-1.293736	-9.075267
H	-4.855014	-2.001647	-7.531614
H	-6.996055	0.505819	-7.411060

H	-6.378671	1.741371	-6.342003
H	-6.337718	0.038952	-5.863368

09f_TS-H_dimer_intact_bp86.log

SCF (RB-P86) = -3324.94927056
E(SCF)+ZPE(0 K)= -3324.462153
H(298 K)= -3324.416990
G(298 K)= -3324.541947
Lowest Frequency = -864.3789cm-1

C	-5.176319	2.521969	-1.212945
C	-4.886744	1.141024	-0.892874
H	-4.035793	1.112911	-0.196567
H	-5.767426	0.691391	-0.410982
H	-4.549382	0.041206	-1.954968
F	-4.081459	3.199333	-1.768487
F	-6.198400	2.676327	-2.159765
F	-5.585983	3.393650	-0.180429
K	-4.330893	1.569855	-4.063806
N	-4.253296	-0.947085	-2.636300
Si	-2.784587	-1.489907	-1.873775
Si	-5.706413	-1.883307	-2.839569
C	-2.040676	-3.033473	-2.703225
C	-1.517502	-0.100229	-2.008635
C	-2.994771	-1.924126	-0.050314
C	-7.194126	-0.726094	-2.893634
C	-5.734586	-2.855691	-4.470216
C	-6.007028	-3.146407	-1.470496
H	-1.334830	-2.809919	-3.521378
H	-2.798969	-3.746775	-3.065336
H	-1.441812	-3.574975	-1.952530
H	-0.559460	-0.393054	-1.549014
H	-1.867990	0.808182	-1.494057
H	-1.302125	0.170270	-3.054509
H	-3.543586	-1.139407	0.492138
H	-2.010212	-2.041825	0.432318
H	-3.543576	-2.869158	0.079118
H	-7.127081	0.033166	-3.688560
H	-7.335388	-0.198770	-1.938971
H	-8.108152	-1.310178	-3.090636
H	-5.719986	-2.204886	-5.360969
H	-6.684230	-3.413074	-4.527226
H	-4.938284	-3.615475	-4.546852
H	-6.965261	-3.668367	-1.629994
H	-6.051042	-2.658553	-0.484314
H	-5.215684	-3.911695	-1.433042
N	-3.245999	0.709861	-6.380324
K	-3.003990	-1.761287	-5.380666
Si	-1.659799	1.291651	-6.463500
Si	-4.499195	0.557027	-7.505608
C	-1.114948	2.038909	-8.117777
C	-1.337699	2.626475	-5.144247
C	-0.377968	-0.078159	-6.092947
C	-4.512039	1.799848	-8.935862
C	-4.559550	-1.172227	-8.315719
C	-6.187207	0.735540	-6.641609
H	-0.062263	2.365641	-8.072367
H	-1.723838	2.915554	-8.388501
H	-1.200352	1.306353	-8.936643
H	-0.305662	3.009726	-5.212170
H	-1.453527	2.246487	-4.114370
H	-2.009773	3.492532	-5.271425
H	-0.442010	-0.911055	-6.815277
H	-0.456951	-0.490364	-5.071403
H	0.646617	0.322691	-6.170408
H	-3.658463	1.654195	-9.615674
H	-4.468360	2.835309	-8.560792
H	-5.434415	1.699756	-9.532756
H	-3.584641	-1.444860	-8.754141
H	-5.301843	-1.199038	-9.131523
H	-4.857782	-1.974573	-7.616901
H	-7.011731	0.528526	-7.343961
H	-6.360940	1.754176	-6.252620
H	-6.304202	0.020158	-5.809842

10a_TS-H_dimer_parsep_pbe0_2.log

SCF (RPBE1PBE) = -3322.75998390
E(SCF)+ZPE(0 K)= -3322.258882
H(298 K)= -3322.214088
G(298 K)= -3322.337153
Lowest Frequency = -1148.3972cm-1

C	-4.913383	0.893435	2.432857
C	-5.238542	-0.235035	1.596715
H	-4.560260	-1.059180	1.829136
H	-6.271448	-0.538278	1.785261
H	-5.092988	0.109914	0.132021
F	-3.629257	1.335410	2.268206
F	-5.692936	2.008117	2.150752
F	-5.041083	0.781777	3.796889
K	-3.981000	2.855674	-0.153467
N	-5.025098	0.394774	-1.079801
Si	-3.654756	-0.242444	-1.883906
Si	-6.612455	0.477024	-1.712989
C	-3.977920	-1.847224	-2.808121
C	-2.947329	0.977545	-3.135932
C	-2.302520	-0.584175	-0.623021
C	-7.580282	1.753188	-0.714777
C	-6.676916	0.991627	-3.517781
C	-7.543712	-1.147678	-1.557826
H	-4.708654	-1.712544	-3.611610
H	-4.363591	-2.615278	-2.130400
H	-3.057661	-2.231908	-3.260726
H	-2.105521	0.526937	-3.673215
H	-2.565879	1.895940	-2.674940
H	-3.695142	1.269673	-3.878712
H	-2.016650	0.302280	-0.046902
H	-1.399931	-0.943898	-1.128067
H	-2.610508	-1.351365	0.093049
H	-7.099868	2.732396	-0.806243
H	-7.606546	1.442559	0.334245
H	-8.609288	1.851033	-1.072850
H	-6.198184	1.959447	-3.690207
H	-7.718352	1.072646	-3.848268
H	-6.186729	0.255545	-4.162126
H	-8.584658	-1.047966	-1.883621
H	-7.547476	-1.497969	-0.520810
H	-7.076372	-1.927221	-2.167372
K	-7.464915	4.040816	1.641505
N	-5.317009	5.174009	0.519630
Si	-4.433064	5.764432	1.821237
Si	-5.812369	5.806490	-0.955458
C	-4.108031	7.620718	1.856697
C	-5.284812	5.388184	3.476100
C	-2.728189	4.950086	1.980894
C	-7.703224	5.810789	-1.120045
C	-5.274732	7.566619	-1.360599
C	-5.202581	4.762006	-2.415714
H	-5.040872	8.192407	1.832307
H	-3.495947	7.946456	1.011138
H	-3.576621	7.893138	2.775228
H	-4.666391	5.752901	4.303181
H	-5.421338	4.314765	3.653511
H	-6.253625	5.894338	3.575424
H	-2.139030	5.054104	1.061971
H	-2.785280	3.887980	2.244974
H	-2.151679	5.428006	2.780247
H	-8.174451	6.399929	-0.324010
H	-8.141050	4.805420	-1.117241
H	-8.005017	6.267228	-2.068927
H	-5.665511	8.291864	-0.641441
H	-5.639545	7.853056	-2.353185
H	-4.184555	7.660580	-1.370082
H	-5.576393	5.169687	-3.360970
H	-5.552095	3.724706	-2.374156
H	-4.108181	4.759173	-2.489284

10b_TS-H_dimer_parsep_wb97xd.log

```
SCF (RwB97XD) =       -3324.37628723
E(SCF)+ZPE(0 K)=      -3323.872010
H(298 K)=             -3323.828819
G(298 K)=             -3323.947484
Lowest Frequency = -1311.2194cm-1

C       -4.887756    0.859255    2.437276
C       -5.245746   -0.232959    1.557627
H       -4.606452   -1.088830    1.783588
H       -6.290341   -0.500989    1.732471
H       -5.080069    0.113390    0.106737
F       -3.586257    1.254954    2.304148
F       -5.619261    2.014056    2.176559
F       -5.045445    0.706688    3.790597
K       -3.971308    2.831397   -0.120967
N       -5.003909    0.387511   -1.109832
Si       -3.643735   -0.275544   -1.902633
Si       -6.587742    0.501493   -1.736184
C       -3.990274   -1.898203   -2.786427
C       -2.941036    0.910529   -3.187933
C       -2.292057   -0.590321   -0.634524
C       -7.526992    1.772737   -0.705429
C       -6.645329    1.039090   -3.534577
C       -7.539452   -1.113131   -1.602817
H       -4.728234   -1.769628   -3.583746
H       -4.379751   -2.645774   -2.089488
H       -3.081331   -2.304392   -3.240744
H       -2.089352    0.458879   -3.706435
H       -2.582498    1.847646   -2.749045
H       -3.689226    1.167522   -3.942523
H       -1.981572    0.318167   -0.109297
H       -1.401439   -0.999773   -1.120585
H       -2.617833   -1.307919    0.122711
H       -7.025219    2.742672   -0.769799
H       -7.550029    1.432326    0.334078
H       -8.555445    1.903075   -1.051567
H       -6.137704    1.992657   -3.699488
H       -7.682871    1.152656   -3.864232
H       -6.175945    0.294774   -4.184252
H       -8.579779   -0.998381   -1.922518
H       -7.541878   -1.478188   -0.571633
H       -7.082309   -1.887528   -2.225452
K       -7.489037    4.008125    1.696998
N       -5.329395    5.184865    0.544491
Si       -4.442539    5.790376    1.833510
Si       -5.837134    5.799834   -0.930758
C       -4.083988    7.642010    1.821048
C       -5.298235    5.473284    3.499112
C       -2.752051    4.952530    2.028762
C       -7.728215    5.779205   -1.097854
C       -5.324642    7.566254   -1.346716
C       -5.221638    4.759748   -2.393255
H       -5.005489    8.229789    1.785002
H       -3.470869    7.934731    0.965033
H       -3.543778    7.928635    2.728954
H       -4.684968    5.869748    4.314005
H       -5.430016    4.406703    3.713265
H       -6.270077    5.975502    3.574686
H       -2.150578    5.021487    1.115487
H       -2.833287    3.899182    2.317983
H       -2.176606    5.439257    2.822195
H       -8.209910    6.359803   -0.302989
H       -8.149368    4.767532   -1.094576
H       -8.034077    6.230320   -2.046814
H       -5.714576    8.287948   -0.624468
H       -5.704524    7.846365   -2.334436
H       -4.236692    7.674013   -1.369182
H       -5.604860    5.160355   -3.336664
H       -5.558819    3.719008   -2.346844
H       -4.128459    4.768141   -2.471267
```

10c_TS-H_dimer_parsep_b3lyp.log

```
SCF (RB3LYP) =        -3324.89684278
E(SCF)+ZPE(0 K)=      -3324.396296
H(298 K)=             -3324.351753
G(298 K)=             -3324.473766
Lowest Frequency = -1346.7819cm-1

C       -4.890797    0.917234    2.407320
C       -5.244161   -0.245109    1.630420
H       -4.566164   -1.064574    1.870545
H       -6.275978   -0.529576    1.841460
H       -5.105919    0.072072    0.183823
F       -3.601589    1.350713    2.189771
F       -5.677921    2.033043    2.090543
F       -4.986210    0.874410    3.789950
K       -4.001954    2.870807   -0.226278
N       -5.020130    0.356876   -1.055722
Si       -3.641849   -0.297089   -1.830659
Si       -6.592715    0.475846   -1.715497
C       -3.988376   -1.837225   -2.859758
C       -2.831444    0.962461   -2.987669
C       -2.352466   -0.760333   -0.538299
C       -7.568448    1.755827   -0.721205
C       -6.609785    1.021064   -3.517441
C       -7.556486   -1.138445   -1.601902
H       -4.673991   -1.629086   -3.685171
H       -4.435510   -2.623097   -2.244852
H       -3.065256   -2.236626   -3.291205
H       -2.023492    0.495056   -3.559375
H       -2.378080    1.805237   -2.454166
H       -3.548378    1.370763   -3.704065
H       -2.090798    0.077120    0.114678
H       -1.431375   -1.094617   -1.025096
H       -2.702936   -1.573818    0.101520
H       -7.089937    2.734762   -0.798841
H       -7.613454    1.443302    0.324975
H       -8.590719    1.858800   -1.093325
H       -6.095457    1.973404   -3.663603
H       -7.639137    1.143662   -3.869221
H       -6.130126    0.281276   -4.163367
H       -8.587891   -1.020208   -1.948387
H       -7.588559   -1.500846   -0.570478
H       -7.088339   -1.917473   -2.209460
K       -7.448058    4.010807    1.648139
N       -5.329212    5.201298    0.496413
Si       -4.415098    5.775765    1.784415
Si       -5.869835    5.841342   -0.959342
C       -4.103913    7.638429    1.838633
C       -5.222717    5.361581    3.456945
C       -2.696588    4.969639    1.882954
C       -7.768651    5.812231   -1.080827
C       -5.369471    7.619727   -1.352399
C       -5.277482    4.818851   -2.447181
H       -5.041219    8.201221    1.839875
H       -3.514190    7.979561    0.984755
H       -3.555957    7.906341    2.747594
H       -4.593774    5.717275    4.278490
H       -5.344162    4.284494    3.613163
H       -6.195825    5.851128    3.583731
H       -2.137065    5.091935    0.949057
H       -2.741342    3.903451    2.127445
H       -2.098318    5.434437    2.672536
H       -8.233636    6.376965   -0.264823
H       -8.182214    4.798012   -1.082010
H       -8.104009    6.275144   -2.013934
H       -5.755885    8.326453   -0.614352
H       -5.758880    7.916468   -2.331482
H       -4.282542    7.732441   -1.382059
H       -5.668498    5.231161   -3.382115
H       -5.619622    3.780897   -2.408127
H       -4.185411    4.821998   -2.537079
```

10d_TS-H_dimer_parsep_m062x.log

SCF (RM062X) = -3324.12203988
E(SCF)+ZPE(0 K)= -3323.618506
H(298 K)= -3323.574306
G(298 K)= -3323.695053
Lowest Frequency = -1220.3331cm-1

C	-4.869282	0.954131	2.465431
C	-5.181715	-0.144142	1.570542
H	-4.523446	-0.980225	1.813703
H	-6.220716	-0.439112	1.734147
H	-5.045467	0.190112	0.125211
F	-3.567412	1.356376	2.384329
F	-5.590959	2.104360	2.171684
F	-5.083237	0.799722	3.803155
K	-3.925874	2.788878	0.000516
N	-5.024411	0.452650	-1.113533
Si	-3.668663	-0.137798	-1.966581
Si	-6.638252	0.542787	-1.661581
C	-4.000451	-1.702651	-2.952793
C	-3.003910	1.148719	-3.173522
C	-2.301265	-0.525902	-0.734574
C	-7.519369	1.800596	-0.562097
C	-6.813250	1.083515	-3.451980
C	-7.559121	-1.086231	-1.493037
H	-4.753147	-1.538704	-3.728936
H	-4.361127	-2.502250	-2.300044
H	-3.090553	-2.057160	-3.445681
H	-2.178316	0.740885	-3.764437
H	-2.621275	2.043536	-2.671668
H	-3.782671	1.473898	-3.869403
H	-2.038212	0.324458	-0.097365
H	-1.389906	-0.829003	-1.257670
H	-2.593067	-1.346069	-0.073595
H	-6.955826	2.738591	-0.598020
H	-7.533708	1.417176	0.462873
H	-8.545801	1.997957	-0.880932
H	-6.398037	2.076335	-3.637911
H	-7.871214	1.108052	-3.730645
H	-6.315689	0.380570	-4.126120
H	-8.618635	-0.978429	-1.742502
H	-7.489657	-1.471171	-0.471952
H	-7.134489	-1.841485	-2.160494
K	-7.486889	4.004059	1.677254
N	-5.327253	5.087565	0.543488
Si	-4.492445	5.728088	1.851162
Si	-5.748707	5.629181	-0.988206
C	-4.145231	7.579539	1.796140
C	-5.418088	5.448670	3.486015
C	-2.811324	4.897887	2.128761
C	-7.627780	5.647776	-1.242099
C	-5.167939	7.358125	-1.462304
C	-5.059017	4.495795	-2.346089
H	-5.069804	8.156283	1.706861
H	-3.502335	7.849148	0.954847
H	-3.642333	7.898763	2.713962
H	-4.828291	5.849685	4.315461
H	-5.573365	4.388011	3.713457
H	-6.382685	5.968338	3.518949
H	-2.175239	4.955778	1.238676
H	-2.903863	3.848947	2.428831
H	-2.268986	5.397678	2.936690
H	-8.115787	6.293097	-0.503363
H	-8.086714	4.654975	-1.192522
H	-7.877799	6.050571	-2.228162
H	-5.579467	8.123675	-0.799924
H	-5.483935	7.596173	-2.482557
H	-4.078116	7.436930	-1.427486
H	-5.425757	4.801419	-3.330443
H	-5.347197	3.446127	-2.223417
H	-3.964930	4.549409	-2.390129

10e_TS-H_dimer_parsep_m06l.log

SCF (RM06L) = -3324.47135233
E(SCF)+ZPE(0 K)= -3323.966670
H(298 K)= -3323.922412
G(298 K)= -3324.042023
Lowest Frequency = -1127.8561cm-1

C	-4.854865	0.908520	2.414850
C	-5.241078	-0.173818	1.551495
H	-4.631918	-1.046333	1.788802
H	-6.290434	-0.413429	1.728607
H	-5.068420	0.165851	0.070967
F	-3.533872	1.253466	2.292404
F	-5.537931	2.100122	2.118088
F	-5.036580	0.810582	3.773913
K	-3.888160	2.808263	-0.032803
N	-5.009985	0.447957	-1.139416
Si	-3.657818	-0.190284	-1.964531
Si	-6.614384	0.543381	-1.715195
C	-3.977928	-1.835765	-2.806984
C	-3.025516	0.994139	-3.281086
C	-2.261378	-0.441594	-0.733347
C	-7.513287	1.806310	-0.641475
C	-6.751849	1.067098	-3.509269
C	-7.549565	-1.074380	-1.546501
H	-4.735635	-1.753001	-3.589043
H	-4.328258	-2.586783	-2.096039
H	-3.074637	-2.230100	-3.277974
H	-2.156485	0.580018	-3.798278
H	-2.711858	1.958161	-2.872513
H	-3.779748	1.204591	-4.041499
H	-1.910924	0.484968	-0.271885
H	-1.391808	-0.884019	-1.224094
H	-2.547535	-1.111714	0.078855
H	-6.951303	2.745215	-0.632276
H	-7.586634	1.424327	0.379821
H	-8.522866	2.024302	-0.994457
H	-6.301928	2.039801	-3.711813
H	-7.801185	1.128560	-3.808023
H	-6.276894	0.343982	-4.175577
H	-8.606090	-0.963600	-1.801421
H	-7.499229	-1.461753	-0.526989
H	-7.139472	-1.844440	-2.202649
K	-7.485033	3.989361	1.703064
N	-5.307332	5.115862	0.561066
Si	-4.478292	5.749130	1.870164
Si	-5.773250	5.684059	-0.942325
C	-4.105441	7.594327	1.829583
C	-5.409831	5.480645	3.502295
C	-2.809965	4.903337	2.166074
C	-7.657703	5.699207	-1.147019
C	-5.221350	7.420228	-1.418012
C	-5.126290	4.578412	-2.340336
H	-5.013626	8.194897	1.748979
H	-3.460008	7.872631	0.994970
H	-3.596462	7.905352	2.745423
H	-4.834292	5.889107	4.336132
H	-5.566923	4.425698	3.747238
H	-6.377840	5.990466	3.533982
H	-2.149002	4.957494	1.296120
H	-2.907251	3.853614	2.455476
H	-2.272659	5.385156	2.986334
H	-8.141229	6.323520	-0.389667
H	-8.118458	4.708297	-1.107713
H	-7.942778	6.117520	-2.115379
H	-5.621080	8.185671	-0.750852
H	-5.559144	7.667115	-2.427833
H	-4.134312	7.520998	-1.412136
H	-5.527469	4.886179	-3.308961
H	-5.397907	3.524063	-2.228354
H	-4.037019	4.633714	-2.432604

10f_TS-H_dimer_parsep_bp86.log

SCF (RB-P86) = -3324.94725389

```
E(SCF)+ZPE(0 K)=      -3324.461237
H(298 K)=             -3324.415561
G(298 K)=             -3324.540098
Lowest Frequency = -1096.8357cm-1

C       -4.912400    0.838681    2.461930
C       -5.250830   -0.275127    1.612818
H       -4.573665   -1.117014    1.812922
H       -6.295668   -0.572639    1.780450
H       -5.103147    0.100131    0.174005
F       -3.595902    1.267345    2.323423
F       -5.681949    1.999227    2.174512
F       -5.068603    0.735085    3.858261
K       -3.963410    2.838790   -0.094573
N       -5.030675    0.422939   -1.060499
Si       -3.659635   -0.212637   -1.888838
Si       -6.625537    0.509746   -1.704511
C       -3.997276   -1.811465   -2.840698
C       -2.953215    1.035202   -3.130656
C       -2.296260   -0.578030   -0.629603
C       -7.596582    1.778680   -0.681412
C       -6.686923    1.047945   -3.513801
C       -7.558981   -1.128558   -1.568414
H       -4.736767   -1.658785   -3.643042
H       -4.387866   -2.592504   -2.168677
H       -3.077336   -2.200268   -3.308751
H       -2.117030    0.590649   -3.696597
H       -2.555455    1.945530   -2.650338
H       -3.714825    1.355055   -3.858595
H       -1.995447    0.307911   -0.046329
H       -1.392281   -0.947097   -1.141666
H       -2.609990   -1.350121    0.090043
H       -7.105909    2.761696   -0.760402
H       -7.617430    1.448498    0.369352
H       -8.633976    1.889039   -1.033513
H       -6.195484    2.019156   -3.677825
H       -7.733595    1.143330   -3.849299
H       -6.198849    0.309383   -4.169456
H       -8.608473   -1.030012   -1.893028
H       -7.560041   -1.495981   -0.529486
H       -7.086999   -1.904226   -2.192457
K       -7.460746    4.004787    1.670597
N       -5.329563    5.145627    0.534387
Si       -4.440856    5.776693    1.830522
Si       -5.798072    5.755928   -0.973564
C       -4.181830    7.654627    1.854295
C       -5.265188    5.368132    3.503789
C       -2.697690    5.014204    1.971735
C       -7.696922    5.786359   -1.159636
C       -5.217807    7.505675   -1.415072
C       -5.178004    4.656628   -2.403674
H       -5.142100    8.193808    1.817709
H       -3.573428    7.998268    1.003709
H       -3.663691    7.960227    2.779087
H       -4.640872    5.740807    4.332938
H       -5.376097    4.283170    3.673747
H       -6.250478    5.853908    3.623752
H       -2.121678    5.128864    1.037114
H       -2.717431    3.945343    2.245952
H       -2.117968    5.516484    2.764322
H       -8.167141    6.409400   -0.378841
H       -8.154043    4.782129   -1.127214
H       -7.989495    6.220225   -2.130605
H       -5.617437    8.261142   -0.721070
H       -5.551324    7.776494   -2.431250
H       -4.118815    7.581464   -1.396055
H       -5.564578    5.018954   -3.370654
H       -5.510917    3.609085   -2.317838
H       -4.076821    4.666681   -2.485057
```

11a_TS-H_dimer_sep_pbe0.log

```
SCF (RPBE1PBE) =      -3322.74003736
E(SCF)+ZPE(0 K)=      -3322.239694
H(298 K)=             -3322.194378
G(298 K)=             -3322.321398
Lowest Frequency = -1240.0955cm-1

C       -4.486298    2.631480   -1.096949
C       -5.185608    1.464794   -0.617249
H       -4.884200    1.222146    0.403503
H       -6.264589    1.611764   -0.697757
H       -4.882446    0.249097   -1.364394
F       -3.130142    2.469977   -1.152657
F       -4.846501    2.993305   -2.367740
F       -4.626246    3.822306   -0.385092
K       -3.347717    0.942736   -3.590558
N       -4.649131   -0.827008   -2.005179
Si       -3.462168   -1.837919   -1.293533
Si       -6.035657   -1.327781   -2.874643
C       -2.076136   -2.208201   -2.523795
C       -2.680857   -0.958954    0.168182
C       -4.102302   -3.496877   -0.687457
C       -6.684261    0.134099   -3.878234
C       -5.668680   -2.696105   -4.111292
C       -7.437103   -1.902723   -1.765311
H       -1.488276   -1.318099   -2.780738
H       -2.466291   -2.638356   -3.451941
H       -1.365976   -2.928332   -2.102829
H       -1.878130   -1.569218    0.594992
H       -3.416867   -0.780052    0.955874
H       -2.249608    0.007485   -0.110668
H       -4.888807   -3.363994    0.060620
H       -3.292051   -4.066784   -0.219764
H       -4.507120   -4.107022   -1.500036
H       -6.045685    0.380173   -4.735781
H       -6.801275    1.033957   -3.267046
H       -7.667746   -0.104448   -4.296700
H       -4.899596   -2.390578   -4.828485
H       -6.570039   -2.944885   -4.682293
H       -5.325275   -3.613165   -3.624496
H       -8.325426   -2.165496   -2.349677
H       -7.722438   -1.123063   -1.052143
H       -7.142943   -2.784596   -1.188789
K       -6.233576    3.285936    1.827061
N       -5.841769    1.162338    3.249613
Si       -6.944634   -0.045547    2.915278
Si       -4.547004    1.338399    4.286191
C       -7.776867   -0.809116    4.433932
C       -8.358144    0.642165    1.848963
C       -6.246907   -1.497802    1.927423
C       -3.735233    3.034531    4.014852
C       -4.996342    1.283241    6.124163
C       -3.151488    0.079726    4.069307
H       -8.229795   -0.038389    5.066599
H       -7.047025   -1.349712    5.046351
H       -8.562982   -1.520201    4.155307
H       -9.102308   -0.131356    1.629812
H       -8.001156    1.010362    0.879514
H       -8.886614    1.459116    2.355130
H       -5.848216   -1.155624    0.968479
H       -7.009602   -2.256912    1.721015
H       -5.430806   -1.984130    2.471945
H       -4.423540    3.860857    4.233743
H       -3.368021    3.149877    2.987313
H       -2.869891    3.170492    4.672677
H       -5.772900    2.019396    6.358805
H       -4.132644    1.491176    6.766051
H       -5.385683    0.298097    6.402269
H       -2.331832    0.258069    4.774925
H       -2.734782    0.119348    3.057809
H       -3.513563   -0.940667    4.235392
```

11b_TS-H_dimer_sep_wb97xd.log

```
SCF (RwB97XD) =       -3324.35890898
E(SCF)+ZPE(0 K)=      -3323.854785
H(298 K)=             -3323.810591
```

G(298 K)=   -3323.931937
Lowest Frequency = -1362.4774cm-1

C   -4.730252   2.732875   -0.995340
C   -5.395201   1.539256   -0.519337
H   -5.111702   1.321144   0.511625
H   -6.476174   1.645660   -0.625512
H   -4.987800   0.354839   -1.264592
F   -3.368034   2.630756   -1.008081
F   -5.067287   3.061553   -2.281715
F   -4.946463   3.923516   -0.303695
K   -3.371566   1.112279   -3.528469
N   -4.645762   -0.693978   -1.902488
Si   -3.426825   -1.603400   -1.118004
Si   -5.974696   -1.298313   -2.790296
C   -1.951318   -1.877234   -2.267493
C   -2.794938   -0.647045   0.367775
C   -3.971091   -3.305487   -0.537140
C   -6.734680   0.114245   -3.784435
C   -5.471746   -2.622129   -4.028360
C   -7.337924   -1.998840   -1.705502
H   -1.397456   -0.953241   -2.470790
H   -2.264551   -2.306635   -3.224223
H   -1.231697   -2.569188   -1.819104
H   -2.009698   -1.206474   0.884546
H   -3.598182   -0.456317   1.082176
H   -2.375056   0.319242   0.075543
H   -4.813496   -3.235536   0.154963
H   -3.151604   -3.809209   -0.015053
H   -4.271936   -3.945369   -1.371191
H   -6.113543   0.424270   -4.632534
H   -6.922275   0.991834   -3.160063
H   -7.693993   -0.195819   -4.209554
H   -4.719878   -2.246398   -4.729424
H   -6.335284   -2.948466   -4.616526
H   -5.055929   -3.505947   -3.537630
H   -8.188463   -2.337650   -2.305224
H   -7.702700   -1.247204   -0.999286
H   -6.977862   -2.850701   -1.122761
K   -6.506131   3.306438   1.955335
N   -5.967253   1.106548   3.287610
Si   -7.043587   -0.102715   2.888772
Si   -4.498935   1.134988   4.078736
C   -7.859925   -0.956130   4.368380
C   -8.478478   0.596316   1.857831
C   -6.321520   -1.492066   1.829235
C   -3.269737   2.264918   3.176117
C   -4.613092   1.835303   5.834483
C   -3.623963   -0.532493   4.272593
H   -8.377808   -0.230139   5.003325
H   -7.108002   -1.454270   4.989243
H   -8.589186   -1.712826   4.060524
H   -9.209604   -0.184610   1.626161
H   -8.136561   0.994898   0.895937
H   -9.018642   1.388512   2.389143
H   -5.929996   -1.096391   0.888190
H   -7.071493   -2.251154   1.584525
H   -5.498404   -1.992819   2.347243
H   -3.600367   3.310589   3.166933
H   -3.133063   1.946992   2.137553
H   -2.285268   2.255055   3.655349
H   -5.046525   2.841017   5.829269
H   -3.633263   1.898351   6.319618
H   -5.255294   1.207118   6.460462
H   -2.681333   -0.423911   4.819404
H   -3.394710   -0.986412   3.304968
H   -4.247712   -1.238659   4.830121

11c_TS-H_dimer_sep_b3lyp.log

SCF (RB3LYP) =   -3324.87289392
E(SCF)+ZPE(0 K)=   -3324.372830
H(298 K)=   -3324.327915
G(298 K)=   -3324.452158
Lowest Frequency = -1334.8197cm-1

C   -4.468738   2.573458   -1.062871
C   -5.203700   1.437548   -0.571243
H   -4.929588   1.202430   0.456065
H   -6.275550   1.598675   -0.681762
H   -4.903052   0.227784   -1.310234
F   -3.104888   2.382674   -1.089972
F   -4.797271   2.923925   -2.356867
F   -4.590393   3.802250   -0.373803
K   -3.278915   0.873226   -3.541385
N   -4.668772   -0.854456   -1.963293
Si   -3.488954   -1.870733   -1.247690
Si   -6.052823   -1.327785   -2.851655
C   -2.081163   -2.213703   -2.470325
C   -2.724364   -1.007688   0.236110
C   -4.133712   -3.547092   -0.684243
C   -6.690312   0.160935   -3.829617
C   -5.677829   -2.676119   -4.114435
C   -7.467021   -1.921710   -1.761790
H   -1.483416   -1.320301   -2.687933
H   -2.458532   -2.609301   -3.417708
H   -1.381068   -2.950548   -2.064486
H   -1.929324   -1.621863   0.669094
H   -3.466051   -0.828775   1.015164
H   -2.286341   -0.042585   -0.031853
H   -4.940144   -3.433775   0.043180
H   -3.334197   -4.122268   -0.206859
H   -4.513226   -4.141984   -1.518571
H   -6.023707   0.450546   -4.650413
H   -6.840701   1.035275   -3.192150
H   -7.652924   -0.074917   -4.293074
H   -4.903243   -2.359065   -4.819351
H   -6.572471   -2.917194   -4.697340
H   -5.336605   -3.599425   -3.640645
H   -8.352389   -2.167960   -2.356225
H   -7.753612   -1.157246   -1.034883
H   -7.181240   -2.815722   -1.202524
K   -6.231933   3.325475   1.811067
N   -5.801378   1.160345   3.186563
Si   -6.922952   -0.034528   2.867876
Si   -4.520346   1.362699   4.234839
C   -7.750223   -0.777987   4.405248
C   -8.345360   0.665773   1.812737
C   -6.255977   -1.507885   1.882844
C   -3.790130   3.110707   4.031397
C   -4.969327   1.211210   6.072158
C   -3.057481   0.185771   3.970874
H   -8.183581   0.002706   5.037735
H   -7.022740   -1.324877   5.012823
H   -8.550022   -1.478144   4.141367
H   -9.098540   -0.099668   1.601551
H   -7.996667   1.033300   0.841762
H   -8.860748   1.486152   2.325173
H   -5.868230   -1.187777   0.914657
H   -7.030692   -2.259055   1.698897
H   -5.439659   -1.998143   2.421060
H   -4.519519   3.892807   4.273974
H   -3.424203   3.281932   3.012165
H   -2.936305   3.266142   4.698169
H   -5.791234   1.885965   6.331045
H   -4.122467   1.450521   6.724136
H   -5.294178   0.194708   6.313350
H   -2.261947   0.357905   4.703918
H   -2.623724   0.309328   2.975000
H   -3.371115   -0.858556   4.063066

11d_TS-H_dimer_sep_m062x.log

SCF (RM062X) =   -3324.10249272
E(SCF)+ZPE(0 K)=   -3323.600189
H(298 K)=   -3323.555385
G(298 K)=   -3323.679018
Lowest Frequency = -1322.7353cm-1

C	-4.789707	2.727302	-0.914506
C	-5.442303	1.502400	-0.486026
H	-5.173477	1.270209	0.547509
H	-6.522453	1.600929	-0.611961
H	-5.007345	0.350020	-1.234742
F	-3.429131	2.634660	-0.946997
F	-5.137880	3.104528	-2.181149
F	-5.010455	3.871718	-0.170961
K	-3.502653	1.237083	-3.389455
N	-4.636817	-0.709336	-1.898049
Si	-3.434712	-1.624133	-1.094757
Si	-5.953898	-1.284345	-2.821314
C	-1.952047	-1.934702	-2.220810
C	-2.817653	-0.643028	0.383280
C	-4.003420	-3.315066	-0.502697
C	-6.600210	0.140088	-3.882352
C	-5.481161	-2.663315	-4.007557
C	-7.381025	-1.885997	-1.759238
H	-1.438452	-1.010383	-2.506557
H	-2.256079	-2.449623	-3.137037
H	-1.207954	-2.563240	-1.722421
H	-2.037811	-1.190318	0.920193
H	-3.630065	-0.436203	1.084782
H	-2.394065	0.317130	0.073546
H	-4.868262	-3.237895	0.159837
H	-3.201541	-3.812038	0.051700
H	-4.274819	-3.965101	-1.338825
H	-5.950024	0.358289	-4.737599
H	-6.728646	1.056808	-3.298527
H	-7.576841	-0.110184	-4.306022
H	-4.688763	-2.342289	-4.689992
H	-6.341926	-2.956626	-4.616112
H	-5.129928	-3.555494	-3.483583
H	-8.235688	-2.190648	-2.370093
H	-7.718350	-1.103783	-1.072165
H	-7.076091	-2.745400	-1.155509
K	-6.607756	3.193559	1.937070
N	-5.955334	1.075051	3.302377
Si	-7.030596	-0.133613	2.892718
Si	-4.468040	1.143936	4.059843
C	-7.830117	-1.017303	4.360893
C	-8.482515	0.581805	1.894745
C	-6.314145	-1.496505	1.795416
C	-3.290690	2.294775	3.117825
C	-4.558258	1.859686	5.809107
C	-3.563286	-0.506248	4.256542
H	-8.343447	-0.303807	5.012280
H	-7.071121	-1.523789	4.965294
H	-8.559067	-1.769638	4.044694
H	-9.214613	-0.199256	1.668534
H	-8.166329	0.991027	0.928484
H	-9.016627	1.362611	2.448358
H	-5.952508	-1.086581	0.846397
H	-7.057732	-2.266155	1.564220
H	-5.469257	-1.986863	2.289034
H	-3.658540	3.327710	3.105351
H	-3.167211	1.969986	2.079507
H	-2.296277	2.323048	3.574122
H	-5.010767	2.856103	5.802077
H	-3.569962	1.945420	6.271237
H	-5.174973	1.225689	6.453717
H	-2.614745	-0.375378	4.786969
H	-3.342745	-0.973061	3.293543
H	-4.168608	-1.210890	4.835677

11e_TS-H_dimer_sep_m06l.log

SCF (RM06L) = -3324.45561515
E(SCF)+ZPE(0 K)= -3323.951668
H(298 K)= -3323.907137
G(298 K)= -3324.028571
Lowest Frequency = -1167.1019cm-1

C	-4.773476	2.733376	-0.911820
C	-5.423439	1.512759	-0.508760
H	-5.172168	1.266993	0.525593
H	-6.502776	1.608653	-0.634596
H	-5.002344	0.333236	-1.256301
F	-3.405530	2.651078	-0.923459
F	-5.106126	3.133317	-2.181946
F	-4.999274	3.892985	-0.156738
K	-3.435983	1.252590	-3.357737
N	-4.656401	-0.725929	-1.904713
Si	-3.456619	-1.660923	-1.125899
Si	-5.961662	-1.286989	-2.849528
C	-1.993252	-1.978687	-2.272581
C	-2.800383	-0.701591	0.345544
C	-4.026292	-3.350324	-0.540028
C	-6.596154	0.143943	-3.901964
C	-5.474423	-2.652383	-4.041362
C	-7.404384	-1.898823	-1.819695
H	-1.460059	-1.067025	-2.557091
H	-2.300134	-2.479274	-3.193698
H	-1.250317	-2.622543	-1.795407
H	-2.048010	-1.276299	0.889900
H	-3.593998	-0.448576	1.051572
H	-2.327180	0.235583	0.043602
H	-4.864646	-3.285516	0.154519
H	-3.218028	-3.873266	-0.023018
H	-4.338040	-3.989509	-1.368477
H	-5.923549	0.402205	-4.725635
H	-6.769303	1.047960	-3.313748
H	-7.548367	-0.109148	-4.372895
H	-4.681768	-2.333304	-4.721778
H	-6.324040	-2.951675	-4.659945
H	-5.121083	-3.550284	-3.531708
H	-8.255946	-2.186631	-2.440573
H	-7.753823	-1.134721	-1.121296
H	-7.126506	-2.771751	-1.225820
K	-6.606641	3.233519	1.925940
N	-5.924939	1.089089	3.255968
Si	-7.003281	-0.130506	2.909731
Si	-4.477873	1.177753	4.077069
C	-7.770401	-0.973901	4.416818
C	-8.470271	0.554002	1.917668
C	-6.310946	-1.523777	1.838176
C	-3.298235	2.370778	3.197393
C	-4.656245	1.852670	5.834573
C	-3.526193	-0.442329	4.285250
H	-8.273292	-0.253200	5.066233
H	-7.007677	-1.467407	5.024713
H	-8.506553	-1.735343	4.145726
H	-9.200797	-0.227929	1.695760
H	-8.169296	0.962379	0.947729
H	-9.013873	1.335788	2.457359
H	-5.951159	-1.144310	0.876984
H	-7.057598	-2.293227	1.621769
H	-5.467807	-2.021382	2.323817
H	-3.686288	3.394077	3.165543
H	-3.114930	2.061809	2.164292
H	-2.323752	2.432523	3.688983
H	-5.133160	2.835897	5.845410
H	-3.695189	1.955007	6.345825
H	-5.277923	1.194016	6.446986
H	-2.602836	-0.296046	4.852534
H	-3.246987	-0.891894	3.330687
H	-4.117288	-1.184227	4.828418

12a_hmds-h_pbe0.log

SCF (RPBE1PBE) = -873.505955010
E(SCF)+ZPE(0 K)= -873.268886
H(298 K)= -873.251078
G(298 K)= -873.311907
Lowest Frequency = 36.6923cm-1

N	4.582738	3.677942	3.343763

Si	4.062369	4.919665	2.252312
Si	5.184461	2.077443	3.058031
C	3.063576	4.162708	0.862914
C	3.016259	6.125885	3.225139
C	5.511982	5.838067	1.499977
C	3.802220	0.880142	2.651697
C	6.388024	2.088603	1.624584
C	6.042556	1.515518	4.621204
H	2.734664	4.940126	0.165523
H	2.173428	3.653855	1.243661
H	3.649504	3.438007	0.289678
H	2.678189	6.951259	2.590884
H	3.584051	6.558828	4.055182
H	2.130483	5.636755	3.641450
H	6.145566	5.166139	0.913210
H	6.135901	6.290522	2.277210
H	5.169852	6.638627	0.835672
H	3.280225	1.170901	1.735201
H	3.064236	0.844843	3.459180
H	4.190684	-0.133399	2.506478
H	5.914369	2.433609	0.700490
H	6.768781	1.078954	1.438509
H	7.243723	2.738526	1.829227
H	6.428071	0.497150	4.511488
H	5.353132	1.517857	5.471702
H	6.885088	2.168651	4.868004
H	4.561938	3.945075	4.319436

12b_hmds-h_wb97xd.log

SCF (RwB97XD) = -874.011086831
E(SCF)+ZPE(0 K)= -873.772691
H(298 K)= -873.755170
G(298 K)= -873.814889
Lowest Frequency = 46.6145cm-1

N	4.581068	3.678897	3.361193
Si	4.056074	4.914188	2.265835
Si	5.189902	2.085005	3.059289
C	3.051985	4.147412	0.884979
C	3.017104	6.131051	3.232569
C	5.504799	5.819569	1.496692
C	3.812178	0.887825	2.637805
C	6.385529	2.122237	1.619330
C	6.056519	1.504844	4.610815
H	2.714261	4.918463	0.186176
H	2.168520	3.633582	1.272274
H	3.640095	3.424448	0.312832
H	2.678928	6.950790	2.592669
H	3.589076	6.569172	4.055744
H	2.132418	5.648633	3.656606
H	6.131609	5.137954	0.915155
H	6.134396	6.278463	2.264266
H	5.162571	6.612543	0.824882
H	3.287818	1.193649	1.728375
H	3.076425	0.834592	3.445165
H	4.204098	-0.120424	2.473153
H	5.901578	2.473183	0.703592
H	6.774364	1.119473	1.418905
H	7.235366	2.778188	1.824680
H	6.443978	0.490010	4.485225
H	5.371620	1.493892	5.463687
H	6.897583	2.156406	4.862610
H	4.546208	3.938212	4.337299

12c_hmds-h_b3lyp.log

SCF (RB3LYP) = -874.195593237
E(SCF)+ZPE(0 K)= -873.958747
H(298 K)= -873.941052
G(298 K)= -874.001586
Lowest Frequency = 35.6630cm-1

N	4.582357	3.678936	3.351548

Si	4.059522	4.920857	2.256687
Si	5.187179	2.077377	3.060321
C	3.063247	4.156489	0.863986
C	3.007433	6.131488	3.225678
C	5.513492	5.839256	1.502593
C	3.801200	0.877957	2.653815
C	6.384663	2.098172	1.616682
C	6.056398	1.507810	4.619624
H	2.731751	4.929618	0.164491
H	2.175594	3.644671	1.243590
H	3.651889	3.433223	0.294164
H	2.670731	6.954930	2.589688
H	3.571050	6.566472	4.056349
H	2.121029	5.644030	3.640298
H	6.146799	5.166478	0.918657
H	6.136919	6.292374	2.278602
H	5.174186	6.638533	0.836690
H	3.275843	1.172337	1.741779
H	3.066420	0.838737	3.462867
H	4.187832	-0.134523	2.502777
H	5.904658	2.444596	0.697912
H	6.769117	1.092160	1.424033
H	7.237951	2.750717	1.817455
H	6.441630	0.490880	4.504353
H	5.373285	1.506382	5.474093
H	6.899570	2.159236	4.864816
H	4.554328	3.943064	4.328256

12d_hmds-h_m062x.log

SCF (RM062X) = -873.903635789
E(SCF)+ZPE(0 K)= -873.665847
H(298 K)= -873.648112
G(298 K)= -873.708506
Lowest Frequency = 41.0040cm-1

N	5.322934	3.812728	2.975485
Si	4.309404	4.968158	2.172553
Si	5.219637	2.082213	3.008229
C	3.892733	4.351220	0.455321
C	2.704567	5.246732	3.096900
C	5.247388	6.581134	2.075192
C	3.430966	1.567332	3.202584
C	5.879629	1.314916	1.432332
C	6.234676	1.474533	4.454374
H	3.247241	5.068567	-0.059183
H	3.360004	3.396908	0.485548
H	4.794638	4.216638	-0.146653
H	2.091403	6.000031	2.594272
H	2.894772	5.593185	4.116020
H	2.117157	4.327247	3.160168
H	6.171049	6.466943	1.502714
H	5.511122	6.941348	3.073253
H	4.645009	7.354695	1.592384
H	2.813415	1.929140	2.376065
H	3.007788	1.949844	4.134521
H	3.349548	0.476895	3.217247
H	5.299558	1.632608	0.562188
H	5.835363	0.223285	1.481869
H	6.920738	1.601845	1.263182
H	6.224809	0.382930	4.506647
H	5.846794	1.860898	5.399984
H	7.276766	1.792320	4.361867
H	6.096964	4.207966	3.496739

12e_hmds-h_m06l.log

SCF (RM06L) = -874.043574716
E(SCF)+ZPE(0 K)= -873.805014
H(298 K)= -873.787129
G(298 K)= -873.848136
Lowest Frequency = 36.6764cm-1

N	4.543055	3.675528	3.352167

Si	4.047631	4.907277	2.237894
Si	5.188870	2.090160	3.087119
C	3.052656	4.150345	0.849127
C	3.015238	6.138069	3.185263
C	5.513183	5.785804	1.479411
C	3.852232	0.845427	2.684690
C	6.378512	2.150844	1.649310
C	6.063914	1.553924	4.644540
H	2.739224	4.911229	0.131438
H	2.151608	3.657066	1.217015
H	3.627399	3.408436	0.291269
H	2.682716	6.957060	2.545534
H	3.578276	6.581714	4.008493
H	2.123843	5.675076	3.611385
H	6.126147	5.112687	0.878100
H	6.161453	6.222219	2.241161
H	5.189960	6.596845	0.823300
H	3.318559	1.106089	1.769202
H	3.111025	0.778058	3.483043
H	4.267840	-0.154464	2.542637
H	5.896032	2.485878	0.729166
H	6.790859	1.160648	1.445665
H	7.217818	2.820357	1.843154
H	6.471658	0.546337	4.547912
H	5.385531	1.544150	5.499820
H	6.891831	2.219576	4.892948
H	4.359000	3.895916	4.321043

12f_hmds-h_bp86.log

SCF (RB-P86) = -874.179192254
E(SCF)+ZPE(0 K)= -873.948849
H(298 K)= -873.930736
G(298 K)= -873.992154
Lowest Frequency = 36.0298cm-1

N	4.583388	3.681439	3.366157
Si	4.059229	4.927303	2.261167
Si	5.187223	2.071582	3.059897
C	3.071034	4.147710	0.862173
C	2.992943	6.137394	3.229133
C	5.518147	5.855075	1.509858
C	3.789725	0.873988	2.654921
C	6.379536	2.099320	1.602982
C	6.068489	1.495097	4.618412
H	2.738574	4.920621	0.149765
H	2.175951	3.630678	1.241468
H	3.671346	3.418374	0.295617
H	2.651031	6.965267	2.587185
H	3.555319	6.579902	4.067747
H	2.101117	5.641617	3.644421
H	6.159509	5.179507	0.921881
H	6.143840	6.311485	2.293943
H	5.177423	6.661151	0.838888
H	3.258186	1.174509	1.738199
H	3.051683	0.836034	3.472352
H	4.173602	-0.147889	2.498654
H	5.890144	2.458923	0.683876
H	6.761137	1.086022	1.395120
H	7.243929	2.751128	1.804617
H	6.456298	0.470795	4.497314
H	5.385453	1.490394	5.483608
H	6.919733	2.150505	4.862729
H	4.582081	3.954327	4.349721

13a_kch2cf3_pbe0.log

SCF (RPBE1PBE) = -976.516975516
E(SCF)+ZPE(0 K)= -976.479268
H(298 K)= -976.471384
G(298 K)= -976.511982
Lowest Frequency = 33.5596cm-1

C	-1.454650	0.134733	0.006124
C	-0.940255	0.923602	1.083208
F	-1.088829	2.302756	0.870766
F	0.390050	0.753439	1.311885
F	-1.494853	0.804180	2.362850
H	-2.546096	0.194988	-0.015395
H	-1.126438	-0.900626	0.106046
K	-0.757516	2.130568	-1.858973

13b_kch2cf3_wb97xd.log

SCF (RwB97XD) = -976.956058532
E(SCF)+ZPE(0 K)= -976.918289
H(298 K)= -976.910416
G(298 K)= -976.950951
Lowest Frequency = 36.2657cm-1

C	-1.634728	0.250062	0.010654
C	-1.051363	1.004132	1.084280
F	-1.607329	2.236950	1.263898
F	0.310611	1.255248	0.895331
F	-1.045647	0.477661	2.381090
H	-2.718518	0.216412	0.127380
H	-1.219749	-0.760552	0.003201
K	-0.051865	1.663727	-1.899323

13c_kch2cf3_b3lyp.log

SCF (RB3LYP) = -977.083590072
E(SCF)+ZPE(0 K)= -977.046570
H(298 K)= -977.038564
G(298 K)= -977.079432
Lowest Frequency = 30.1169cm-1

C	-1.648052	0.256042	0.016992
C	-1.073628	1.011338	1.080273
F	-1.684469	2.212269	1.319873
F	0.291765	1.345601	0.856858
F	-0.969141	0.449506	2.380612
H	-2.733877	0.243405	0.085808
H	-1.234082	-0.752419	-0.015674
K	0.032896	1.577896	-1.858232

13d_kch2cf3_m062x.log

SCF (RM062X) = -976.920865355
E(SCF)+ZPE(0 K)= -976.882564
H(298 K)= -976.874843
G(298 K)= -976.914535
Lowest Frequency = 59.6140cm-1

C	-1.607884	0.130581	0.013699
C	-1.008609	0.952671	1.025790
F	-1.394199	2.278016	0.943719
F	0.369652	1.006313	0.931949
F	-1.197293	0.679233	2.371774
H	-2.693018	0.159192	0.114483
H	-1.233416	-0.889496	0.101547
K	-0.253822	2.027128	-1.636452

13e_kch2cf3_m06l.log

SCF (RM06L) = -976.990542600
E(SCF)+ZPE(0 K)= -976.953065
H(298 K)= -976.945203
G(298 K)= -976.985131
Lowest Frequency = 61.2830cm-1

C	-1.605172	0.119465	0.012578
C	-1.013869	0.946663	1.011595
F	-1.402995	2.280775	0.913891
F	0.373007	1.003934	0.919807
F	-1.195117	0.703611	2.377813
H	-2.690047	0.133714	0.124218
H	-1.236493	-0.901272	0.120425

K	-0.247902	2.056748	-1.613819

13f_kch2cf3_bp86.log

SCF (RB-P86) = -977.118263215
E(SCF)+ZPE(0 K)= -977.082655
H(298 K)= -977.074419
G(298 K)= -977.115923
Lowest Frequency = 23.5724cm-1

C	-1.642895	0.237980	0.018842
C	-1.070711	1.003935	1.071305
F	-1.659216	2.239047	1.276898
F	0.328436	1.298673	0.869962
F	-0.994476	0.474405	2.420432
H	-2.738528	0.243504	0.062856
H	-1.234244	-0.781305	-0.010630
K	-0.006956	1.627399	-1.843156

14a_kch2cf3_khmds_pbe0.log

SCF (RPBE1PBE) = -2449.24135565
E(SCF)+ZPE(0 K)= -2448.975841
H(298 K)= -2448.948400
G(298 K)= -2449.035818
Lowest Frequency = 11.4870cm-1

N	-3.386648	1.115426	-6.254517
K	-3.325853	-1.069414	-4.679513
Si	-1.784382	1.608891	-6.298878
Si	-4.589012	0.763258	-7.369504
C	-1.041021	1.929377	-8.000216
C	-1.490260	3.203022	-5.312729
C	-0.624836	0.354648	-5.467634
C	-4.123092	0.891798	-9.190705
C	-5.272811	-0.995172	-7.148722
C	-6.120049	1.864451	-7.159606
H	0.000944	2.254603	-7.907231
H	-1.585697	2.713299	-8.534955
H	-1.053421	1.031277	-8.624676
H	-0.432278	3.482778	-5.357686
H	-1.727761	3.090243	-4.247700
H	-2.051749	4.055438	-5.714141
H	-0.666100	-0.633700	-5.942042
H	-0.818633	0.243357	-4.393243
H	0.414104	0.691664	-5.548328
H	-3.307818	0.208710	-9.447271
H	-3.804024	1.904129	-9.455475
H	-4.980341	0.637710	-9.823694
H	-4.505778	-1.766604	-7.291836
H	-6.048709	-1.193040	-7.895927
H	-5.752733	-1.144966	-6.173331
H	-6.870035	1.615885	-7.918130
H	-5.889715	2.928971	-7.288830
H	-6.614029	1.722941	-6.190359
K	-4.575678	2.956847	-4.592864
C	-5.219289	1.833951	-2.000919
C	-4.772023	0.485898	-2.049810
H	-4.679010	2.378521	-1.223351
H	-6.296298	1.863893	-1.822215
F	-3.416893	0.348806	-2.314110
F	-5.388292	-0.278280	-3.029416
F	-4.910310	-0.353895	-0.929610

14b_kch2cf3_khmds_wb97xd.log

SCF (RwB97XD) = -2450.35493332
E(SCF)+ZPE(0 K)= -2450.088221
H(298 K)= -2450.061016
G(298 K)= -2450.146721
Lowest Frequency = 19.3443cm-1

N	-3.392675	1.077951	-6.251858
K	-3.337787	-1.129460	-4.640598
Si	-1.797901	1.589846	-6.297003
Si	-4.579855	0.735520	-7.383377
C	-1.070734	1.950004	-7.999067
C	-1.506930	3.170470	-5.288034
C	-0.613093	0.336841	-5.499946
C	-4.088845	0.875934	-9.198616
C	-5.271571	-1.023405	-7.191831
C	-6.116384	1.833576	-7.193007
H	-0.032913	2.285675	-7.907153
H	-1.627774	2.736507	-8.515512
H	-1.077053	1.064678	-8.640250
H	-0.452323	3.458449	-5.337001
H	-1.735981	3.038111	-4.224221
H	-2.078786	4.023863	-5.669870
H	-0.658365	-0.645886	-5.983320
H	-0.789657	0.211682	-4.424938
H	0.420974	0.682050	-5.594149
H	-3.269809	0.195898	-9.448223
H	-3.767398	1.888923	-9.454625
H	-4.936781	0.623959	-9.843368
H	-4.503586	-1.794211	-7.325543
H	-6.033666	-1.212753	-7.953895
H	-5.767362	-1.179450	-6.226454
H	-6.846297	1.600160	-7.974211
H	-5.882525	2.899504	-7.293357
H	-6.633339	1.673345	-6.239664
K	-4.625757	2.894084	-4.521156
C	-5.213657	1.923513	-1.822312
C	-4.772490	0.591863	-2.063238
H	-4.638221	2.369490	-1.010287
H	-6.281880	1.931526	-1.601569
F	-3.423303	0.493151	-2.373229
F	-5.410840	-0.027309	-3.133906
F	-4.894967	-0.399377	-1.074385

14c_kch2cf3_khmds_b3lyp.log

SCF (RB3LYP) = -2450.68696282
E(SCF)+ZPE(0 K)= -2450.422370
H(298 K)= -2450.394895
G(298 K)= -2450.481772
Lowest Frequency = 16.1300cm-1

N	-3.356445	1.102998	-6.249560
K	-3.297908	-1.090874	-4.660432
Si	-1.753479	1.593726	-6.312540
Si	-4.582696	0.775350	-7.347053
C	-1.012385	1.852728	-8.029918
C	-1.455786	3.225028	-5.379985
C	-0.595112	0.360472	-5.439599
C	-4.147463	0.939250	-9.177333
C	-5.268050	-0.988664	-7.141272
C	-6.106632	1.882794	-7.085504
H	0.033846	2.165402	-7.954456
H	-1.548741	2.624903	-8.587151
H	-1.041859	0.935849	-8.623970
H	-0.405609	3.522333	-5.456603
H	-1.667723	3.142772	-4.307732
H	-2.043838	4.052138	-5.793463
H	-0.628717	-0.636540	-5.894108
H	-0.807085	0.268443	-4.367918
H	0.443611	0.696594	-5.510788
H	-3.337220	0.262970	-9.461258
H	-3.831812	1.955684	-9.425725
H	-5.013802	0.699473	-9.801863
H	-4.501224	-1.755534	-7.301323
H	-6.054754	-1.181387	-7.876781
H	-5.729604	-1.148783	-6.159897
H	-6.877009	1.653588	-7.827931
H	-5.870914	2.946790	-7.200902
H	-6.575724	1.729706	-6.106905
K	-4.510348	2.922990	-4.515339
C	-5.398068	1.787216	-1.975205
C	-4.804603	0.511996	-2.098203

```
H       -4.907107    2.381346   -1.205499
H       -6.472915    1.713594   -1.820486
F       -3.442376    0.540061   -2.450489
F       -5.390583   -0.306056   -3.064812
F       -4.749399   -0.393633   -0.987167
```

14d_kch2cf3_khmds_m062x.log

```
SCF (RM062X) =        -2450.21014003
E(SCF)+ZPE(0 K)=      -2449.943423
H(298 K)=             -2449.916205
G(298 K)=             -2450.001157
Lowest Frequency = 19.9374cm-1
```

```
N       -3.396064    1.074584   -6.236213
K       -3.349605   -1.110391   -4.661667
Si       -1.798531    1.572909   -6.245640
Si       -4.606271    0.728914   -7.338843
C       -1.030104    1.872977   -7.939573
C       -1.534157    3.179003   -5.270518
C       -0.656417    0.329567   -5.375607
C       -4.144549    0.884166   -9.158927
C       -5.279072   -1.034483   -7.126694
C       -6.138644    1.821969   -7.100528
H        0.011817    2.191896   -7.841021
H       -1.564915    2.653228   -8.487587
H       -1.043788    0.968141   -8.552773
H       -0.481650    3.474636   -5.308821
H       -1.779767    3.069163   -4.208138
H       -2.106990    4.017074   -5.682896
H       -0.681563   -0.660905   -5.844203
H       -0.883795    0.225612   -4.308309
H        0.381856    0.669845   -5.428341
H       -3.324678    0.210856   -9.422560
H       -3.830706    1.901730   -9.405943
H       -4.997315    0.635546   -9.797649
H       -4.507029   -1.798467   -7.273244
H       -6.055813   -1.234727   -7.870435
H       -5.752141   -1.187562   -6.149634
H       -6.896528    1.580621   -7.851546
H       -5.911928    2.887383   -7.218365
H       -6.617231    1.666126   -6.126869
K       -4.583963    2.872042   -4.547064
C       -5.241509    1.884434   -1.800465
C       -4.686072    0.732640   -2.404879
H       -4.634723    2.220328   -0.962572
H       -6.284371    1.725787   -1.535199
F       -3.379314    0.906925   -2.846536
F       -5.369438    0.311186   -3.541353
F       -4.554562   -0.498032   -1.728562
```

14e_kch2cf3_khmds_m06l.log

```
SCF (RM06L) =         -2450.41901969
E(SCF)+ZPE(0 K)=      -2450.152064
H(298 K)=             -2450.124742
G(298 K)=             -2450.207923
Lowest Frequency = 40.6118cm-1
```

```
N       -3.365298    1.106480   -6.209729
K       -3.328500   -1.098154   -4.558721
Si       -1.766772    1.581153   -6.264211
Si       -4.588958    0.746233   -7.285677
C       -1.007312    1.828782   -7.969968
C       -1.459042    3.209940   -5.342537
C       -0.617851    0.355286   -5.381319
C       -4.181735    0.892103   -9.118722
C       -5.253502   -1.017687   -7.062390
C       -6.126811    1.826116   -7.030351
H        0.037281    2.140780   -7.892421
H       -1.528201    2.596964   -8.544649
H       -1.024492    0.914402   -8.566004
H       -0.406391    3.495721   -5.405528
H       -1.681804    3.147421   -4.272905
H       -2.023443    4.048564   -5.761018
H       -0.655626   -0.650201   -5.811578
H       -0.816760    0.276118   -4.307972
H        0.423458    0.676125   -5.461058
H       -3.370744    0.224353   -9.415331
H       -3.880757    1.904882   -9.393203
H       -5.048110    0.637347   -9.734356
H       -4.486157   -1.785181   -7.203010
H       -6.030033   -1.232911   -7.800108
H       -5.724849   -1.177858   -6.087958
H       -6.900655    1.571912   -7.758518
H       -5.926124    2.893056   -7.166112
H       -6.590190    1.686761   -6.049045
K       -4.538786    2.959036   -4.506049
C       -5.381099    1.785292   -1.782677
C       -4.769042    0.747295   -2.486501
H       -4.828695    2.058239   -0.887826
H       -6.431522    1.587356   -1.588275
F       -3.440493    0.997756   -2.837868
F       -5.397036    0.420675   -3.688436
F       -4.593484   -0.609435   -1.967142
```

14f_kch2cf3_khmds_bp86.log

```
SCF (RB-P86) =        -2450.74635812
E(SCF)+ZPE(0 K)=      -2450.489700
H(298 K)=             -2450.461424
G(298 K)=             -2450.549494
Lowest Frequency = 17.9567cm-1
```

```
N       -3.480767    1.046899   -6.262700
K       -3.435787   -1.142927   -4.696047
Si       -1.885752    1.608438   -6.237848
Si       -4.614586    0.687014   -7.463112
C       -1.128641    2.102155   -7.903712
C       -1.685302    3.135072   -5.111899
C       -0.688402    0.324622   -5.484550
C       -4.006246    0.732383   -9.257317
C       -5.361173   -1.055184   -7.223746
C       -6.134411    1.841401   -7.400578
H       -0.102956    2.484636   -7.767166
H       -1.716561    2.895704   -8.392137
H       -1.076537    1.249942   -8.599026
H       -0.627656    3.444907   -5.070539
H       -1.985538    2.934498   -4.068139
H       -2.248615    4.009788   -5.482683
H       -0.716291   -0.638597   -6.024081
H       -0.878647    0.138831   -4.412383
H        0.352222    0.685225   -5.543204
H       -3.167439    0.035439   -9.414154
H       -3.662792    1.737516   -9.547432
H       -4.813959    0.442850   -9.950614
H       -4.608713   -1.857945   -7.323520
H       -6.124194   -1.254472   -7.994676
H       -5.877768   -1.167525   -6.253728
H       -6.822085    1.621602   -8.234445
H       -5.853748    2.904804   -7.496362
H       -6.730914    1.714299   -6.479523
K       -4.811533    2.754294   -4.580318
C       -4.790343    2.005940   -1.748918
C       -4.592183    0.646470   -2.043260
H       -4.039490    2.404020   -1.058391
H       -5.822226    2.231457   -1.454397
F       -3.267460    0.290511   -2.329264
F       -5.341513    0.168912   -3.186460
F       -4.961522   -0.418253   -1.102845
```

15a_TS-bF_pbe0.log

```
SCF (RPBE1PBE) =      -976.515573580
E(SCF)+ZPE(0 K)=      -976.479169
H(298 K)=             -976.471445
G(298 K)=             -976.511347
Lowest Frequency = -188.2299cm-1
```

C	-1.653619	0.232838	0.085225
C	-1.085250	1.059979	1.059281
F	-1.765075	2.181364	1.403166
F	0.240961	1.735910	0.661145
F	-0.655141	0.475365	2.204337
H	-2.552235	0.612172	-0.386594
H	-1.614120	-0.830311	0.290479
K	0.497736	0.791737	-1.708184

15b_TS-bF_wb97xd.log

SCF (RwB97XD) = -976.954312934
E(SCF)+ZPE(0 K)= -976.917993
H(298 K)= -976.910210
G(298 K)= -976.950497
Lowest Frequency = -162.2671cm-1

C	-1.646049	0.232071	0.073598
C	-1.080686	1.062192	1.057809
F	-1.771375	2.177603	1.407277
F	0.231025	1.734334	0.664766
F	-0.659561	0.472399	2.205567
H	-2.559630	0.609616	-0.369770
H	-1.629555	-0.825831	0.306452
K	0.529088	0.796673	-1.736847

15c_TS-bF_b3lyp.log

SCF (RB3LYP) = -977.083056211
E(SCF)+ZPE(0 K)= -977.046760
H(298 K)= -977.039222
G(298 K)= -977.078516
Lowest Frequency = -151.8328cm-1

C	-1.624605	0.227697	0.049725
C	-1.062755	1.060669	1.043160
F	-1.842013	2.063745	1.613294
F	0.056644	1.929207	0.562618
F	-0.471647	0.417171	2.085715
H	-2.453696	0.684757	-0.482642
H	-1.818731	-0.781008	0.396685
K	0.630058	0.656817	-1.659702

15d_TS-bF_m062x.log

SCF (RM062X) = -976.918486669
E(SCF)+ZPE(0 K)= -976.881668
H(298 K)= -976.874017
G(298 K)= -976.913560
Lowest Frequency = -294.0040cm-1

C	-1.684664	0.225291	0.114556
C	-1.100307	1.052701	1.065648
F	-1.749933	2.189333	1.400333
F	0.250712	1.729541	0.642687
F	-0.644314	0.485399	2.204141
H	-2.543124	0.614401	-0.412760
H	-1.598263	-0.839759	0.271093
K	0.483149	0.802149	-1.676845

15e_TS-bF_m06l.log

SCF (RM06L) = -976.988843887
E(SCF)+ZPE(0 K)= -976.952410
H(298 K)= -976.944797
G(298 K)= -976.984346
Lowest Frequency = -189.4705cm-1

C	-1.616871	0.220047	0.042070
C	-1.071630	1.053221	1.041938
F	-1.812014	2.104536	1.514641
F	0.136379	1.854676	0.595225
F	-0.546892	0.434791	2.122507
H	-2.498854	0.648737	-0.424072
H	-1.773456	-0.794036	0.392788
K	0.596595	0.737085	-1.676246

15f_TS-bF_bp86.log

SCF (RB-P86) = -977.118039894
E(SCF)+ZPE(0 K)= -977.082944
H(298 K)= -977.075279
G(298 K)= -977.114847
Lowest Frequency = -124.2028cm-1

C	-1.623236	0.230683	0.033994
C	-1.056728	1.054498	1.036222
F	-1.858263	2.018880	1.713973
F	-0.010370	2.004531	0.528041
F	-0.392128	0.391775	2.039474
H	-2.420082	0.725071	-0.533457
H	-1.898733	-0.760866	0.405400
K	0.672797	0.594484	-1.614795

16a_TS-bF_khmds_pbe0.log

SCF (RPBE1PBE) = -2449.24102176
E(SCF)+ZPE(0 K)= -2448.976587
H(298 K)= -2448.949401
G(298 K)= -2449.033985
Lowest Frequency = -166.3256cm-1

N	-3.573323	1.119470	-6.333001
K	-4.005153	-0.965794	-4.680641
Si	-1.916028	1.360498	-6.292750
Si	-4.782307	1.028795	-7.488903
C	-1.040872	1.496899	-7.956434
C	-1.442381	2.924587	-5.328537
C	-1.022356	-0.032053	-5.361377
C	-4.273836	1.362389	-9.272687
C	-5.629522	-0.669866	-7.501131
C	-6.197352	2.240532	-7.127212
H	0.036131	1.638300	-7.814210
H	-1.412147	2.343884	-8.540845
H	-1.179868	0.592596	-8.556872
H	-0.361235	3.094851	-5.374266
H	-1.690692	2.849048	-4.262499
H	-1.922237	3.820927	-5.738967
H	-1.192673	-1.015064	-5.817465
H	-1.307802	-0.076551	-4.302776
H	0.060368	0.133315	-5.373598
H	-3.526225	0.644158	-9.621958
H	-3.849518	2.364843	-9.385495
H	-5.139736	1.291765	-9.940045
H	-4.913914	-1.483945	-7.667140
H	-6.366995	-0.727572	-8.309082
H	-6.183696	-0.872026	-6.575788
H	-6.963759	2.179535	-7.907414
H	-5.855058	3.282282	-7.105515
H	-6.709280	2.016967	-6.183029
C	-4.082850	0.893409	-2.061807
C	-5.357374	0.322703	-2.178966
F	-5.393991	-1.041591	-2.181535
F	-6.040989	0.569126	-3.511610
F	-6.359782	0.751765	-1.371982
H	-3.285852	0.189086	-1.849109
H	-4.045716	1.813824	-1.487135
K	-4.563167	2.728808	-4.386712

16b_TS-bF_khmds_wb97xd.log

SCF (RwB97XD) = -2450.35521619
E(SCF)+ZPE(0 K)= -2450.089157
H(298 K)= -2450.062371
G(298 K)= -2450.146310
Lowest Frequency = -168.2124cm-1

N	-3.568377	1.117800	-6.326370
K	-4.013444	-0.980217	-4.632852
Si	-1.912586	1.360888	-6.308100
Si	-4.769598	1.030165	-7.488331
C	-1.057423	1.477477	-7.985187
C	-1.422817	2.937674	-5.372085
C	-0.999113	-0.015638	-5.372232
C	-4.255755	1.394954	-9.265972
C	-5.602945	-0.674902	-7.535968
C	-6.201701	2.220598	-7.122125
H	0.020592	1.618158	-7.857441
H	-1.433895	2.317383	-8.575031
H	-1.205182	0.567555	-8.573709
H	-0.344309	3.110207	-5.442008
H	-1.649933	2.873118	-4.301322
H	-1.915287	3.826915	-5.780635
H	-1.169874	-1.003924	-5.814293
H	-1.274841	-0.050517	-4.311436
H	0.081391	0.156223	-5.398001
H	-3.505949	0.685380	-9.625637
H	-3.833757	2.399343	-9.362096
H	-5.119488	1.332948	-9.935586
H	-4.877985	-1.480808	-7.694842
H	-6.324627	-0.729527	-8.356999
H	-6.170087	-0.890385	-6.622645
H	-6.963616	2.157820	-7.905202
H	-5.873173	3.265527	-7.086316
H	-6.711522	1.979892	-6.182001
C	-4.102828	0.847509	-2.022284
C	-5.406052	0.328149	-2.128662
F	-5.503668	-1.031102	-2.097243
F	-6.070863	0.577745	-3.467194
F	-6.388731	0.820571	-1.332492
H	-3.349368	0.111946	-1.764050
H	-4.034962	1.774705	-1.462350
K	-4.559412	2.736273	-4.339797

16c_TS-bF_khmds_b3lyp.log

SCF (RB3LYP) = -2450.68712787
E(SCF)+ZPE(0 K)= -2450.423586
H(298 K)= -2450.396277
G(298 K)= -2450.482985
Lowest Frequency = -191.1168cm-1

N	-3.518362	1.108794	-6.326316
K	-3.882280	-0.995949	-4.664331
Si	-1.869344	1.406873	-6.327000
Si	-4.760442	1.025254	-7.448790
C	-1.041447	1.580328	-8.015717
C	-1.423520	2.989834	-5.369428
C	-0.910081	0.038264	-5.418137
C	-4.267594	1.169316	-9.266598
C	-5.726157	-0.606756	-7.302432
C	-6.077331	2.368148	-7.163519
H	0.034281	1.748840	-7.903714
H	-1.451170	2.422015	-8.579853
H	-1.174614	0.680371	-8.621655
H	-0.352830	3.202897	-5.445785
H	-1.636775	2.904967	-4.297547
H	-1.950083	3.866674	-5.761977
H	-1.057815	-0.944725	-5.879624
H	-1.182926	-0.026155	-4.358333
H	0.165859	0.236374	-5.441268
H	-3.561310	0.386139	-9.553960
H	-3.797874	2.132636	-9.481097
H	-5.145819	1.077795	-9.913521
H	-5.083404	-1.479738	-7.462760
H	-6.518699	-0.658724	-8.055125
H	-6.224346	-0.710711	-6.331758
H	-6.842881	2.335116	-7.944842
H	-5.644668	3.374404	-7.185873
H	-6.610582	2.237827	-6.214829
C	-4.189134	0.729868	-2.044985
C	-5.479691	0.205938	-2.245006
F	-5.534541	-1.160515	-2.373219
F	-6.118687	0.619067	-3.546046
F	-6.516834	0.554955	-1.405554
H	-3.472153	-0.000972	-1.687690
H	-4.172490	1.663102	-1.489478
K	-4.495445	2.688348	-4.320724

16d_TS-bF_khmds_m062x.log

SCF (RM062X) = -2450.21037217
E(SCF)+ZPE(0 K)= -2449.944836
H(298 K)= -2449.917785
G(298 K)= -2450.002107
Lowest Frequency = -205.9116cm-1

N	-3.585553	1.111325	-6.337097
K	-3.996354	-0.990498	-4.720607
Si	-1.934716	1.369189	-6.257932
Si	-4.800003	1.012717	-7.482880
C	-1.052050	1.588694	-7.907883
C	-1.497225	2.894039	-5.217089
C	-1.037689	-0.048006	-5.368739
C	-4.270074	1.254912	-9.274613
C	-5.700625	-0.656581	-7.419201
C	-6.179551	2.277892	-7.170301
H	0.019032	1.753217	-7.757413
H	-1.445654	2.445731	-8.460696
H	-1.166668	0.704691	-8.540823
H	-0.417974	3.072581	-5.228622
H	-1.773070	2.763917	-4.163922
H	-1.972869	3.804514	-5.597656
H	-1.202425	-1.016468	-5.853955
H	-1.331525	-0.123200	-4.314981
H	0.042910	0.122565	-5.368519
H	-3.527298	0.511620	-9.576037
H	-3.830859	2.244108	-9.428879
H	-5.126135	1.162904	-9.949616
H	-5.018445	-1.500749	-7.568083
H	-6.454489	-0.717403	-8.209634
H	-6.240620	-0.800218	-6.475937
H	-6.937623	2.221806	-7.957058
H	-5.804505	3.307185	-7.167024
H	-6.707282	2.091037	-6.227853
C	-3.993920	0.894774	-2.151485
C	-5.271536	0.340647	-2.278917
F	-5.337965	-1.021534	-2.270388
F	-5.939185	0.566753	-3.635730
F	-6.275613	0.804107	-1.504426
H	-3.190957	0.184476	-2.003951
H	-3.939515	1.804195	-1.566420
K	-4.593156	2.734960	-4.434125

16e_TS-bF_khmds_m06l.log

SCF (RM06L) = -2450.41889479
E(SCF)+ZPE(0 K)= -2450.152345
H(298 K)= -2450.125522
G(298 K)= -2450.208280
Lowest Frequency = -170.0888cm-1

N	-3.564887	1.114664	-6.346486
K	-3.960098	-0.992694	-4.670231
Si	-1.919504	1.383175	-6.281491
Si	-4.785283	1.009622	-7.478256
C	-1.039329	1.643215	-7.925687
C	-1.477970	2.891998	-5.220378
C	-0.994088	-0.032572	-5.423613
C	-4.286978	1.193891	-9.284531
C	-5.716219	-0.639015	-7.370391
C	-6.144404	2.301144	-7.189776
H	0.031080	1.805712	-7.776581
H	-1.422585	2.511972	-8.464268
H	-1.143181	0.782167	-8.588562

```
H        -0.401026    3.076010   -5.230767
H        -1.742423    2.754067   -4.166926
H        -1.948341    3.814485   -5.573440
H        -1.151345   -1.001551   -5.906465
H        -1.262060   -0.128801   -4.366573
H         0.084363    0.142356   -5.436755
H        -3.570024    0.430304   -9.592109
H        -3.830573    2.164311   -9.488435
H        -5.154320    1.103066   -9.943187
H        -5.063406   -1.504712   -7.515778
H        -6.485854   -0.704944   -8.143152
H        -6.244433   -0.767208   -6.420270
H        -6.906896    2.248097   -7.970553
H        -5.764018    3.326732   -7.203816
H        -6.680879    2.150867   -6.247617
C        -4.014015    0.822666   -2.190602
C        -5.326366    0.309752   -2.277977
F        -5.411881   -1.051429   -2.291197
F        -6.001748    0.622399   -3.586291
F        -6.314981    0.746853   -1.445183
H        -3.331547    0.107787   -1.741242
H        -3.978904    1.781629   -1.678164
K        -4.567067    2.753887   -4.411741
```

16f_TS-bF_khmds_bp86.log

```
SCF (RB-P86) =        -2450.74733654
E(SCF)+ZPE(0 K)=      -2450.491043
H(298 K)=             -2450.463316
G(298 K)=             -2450.550112
Lowest Frequency = -154.8315cm-1

N        -3.541137    1.063346   -6.317945
K        -3.882493   -1.058409   -4.713369
Si        -1.890771    1.421991   -6.305938
Si        -4.771223    1.029881   -7.474912
C        -1.033051    1.541369   -7.991937
C        -1.506364    3.067322   -5.412692
C        -0.913979    0.122610   -5.307349
C        -4.232422    1.094714   -9.291272
C        -5.832946   -0.544917   -7.297451
C        -6.018276    2.459528   -7.253434
H         0.047346    1.727295   -7.868833
H        -1.444644    2.362554   -8.599732
H        -1.147616    0.610138   -8.569154
H        -0.440013    3.329142   -5.518175
H        -1.693826    3.016061   -4.325103
H        -2.083183    3.910487   -5.831009
H        -0.989605   -0.888087   -5.745903
H        -1.242291    0.078579   -4.253342
H         0.159869    0.372936   -5.282051
H        -3.545589    0.269464   -9.537978
H        -3.716896    2.038720   -9.528468
H        -5.104896    1.014435   -9.961597
H        -5.244927   -1.465773   -7.458077
H        -6.643455   -0.557165   -8.045402
H        -6.327927   -0.608360   -6.312136
H        -6.755383    2.467743   -8.074141
H        -5.519711    3.444244   -7.262767
H        -6.607931    2.372921   -6.323292
C        -4.103333    0.596820   -2.104480
C        -5.423493    0.107606   -2.206115
F        -5.522243   -1.263949   -2.392026
F        -6.181283    0.613757   -3.422134
F        -6.392177    0.426276   -1.241733
H        -3.388461   -0.155646   -1.758416
H        -4.024988    1.540735   -1.553317
K        -4.531871    2.611532   -4.306815
```

17a_kf_pbe0.log

```
SCF (RPBE1PBE) =      -699.625798337
E(SCF)+ZPE(0 K)=      -699.625097
H(298 K)=             -699.621382
G(298 K)=             -699.647454
Lowest Frequency = 307.7327cm-1

K        3.901505    3.954613    5.782320
F        4.770752    3.796341    3.619991
```

17b_kf_wb97xd.log

```
SCF (RwB97XD) =       -699.870966525
E(SCF)+ZPE(0 K)=      -699.870292
H(298 K)=             -699.866562
G(298 K)=             -699.892680
Lowest Frequency = 296.0428cm-1

K        3.898032    3.955245    5.790960
F        4.774225    3.795709    3.611351
```

17c_kf_b3lyp.log

```
SCF (RB3LYP) =        -699.911245014
E(SCF)+ZPE(0 K)=      -699.910552
H(298 K)=             -699.906832
G(298 K)=             -699.932925
Lowest Frequency = 304.3971cm-1

K        3.898636    3.955135    5.789457
F        4.773621    3.795819    3.612854
```

17d_kf_m062x.log

```
SCF (RM062X) =        -699.849957109
E(SCF)+ZPE(0 K)=      -699.849222
H(298 K)=             -699.845525
G(298 K)=             -699.871557
Lowest Frequency = 322.6345cm-1

K        3.902236    3.954480    5.780503
F        4.770021    3.796474    3.621808
```

17e_kf_m06l.log

```
SCF (RM06L) =         -699.868622636
E(SCF)+ZPE(0 K)=      -699.867901
H(298 K)=             -699.864197
G(298 K)=             -699.890253
Lowest Frequency = 316.6607cm-1

K        3.900118    3.954866    5.785770
F        4.772139    3.796088    3.616541
```

17f_kf_bp86.log

```
SCF (RB-P86) =        -699.946056793
E(SCF)+ZPE(0 K)=      -699.945377
H(298 K)=             -699.941650
G(298 K)=             -699.967760
Lowest Frequency = 298.2872cm-1

K        3.898647    3.955133    5.789430
F        4.773610    3.795821    3.612881
```

18a_kf_khmds_pbe0.log

```
SCF (RPBE1PBE) =      -2172.35663650
E(SCF)+ZPE(0 K)=      -2172.128322
H(298 K)=             -2172.105421
G(298 K)=             -2172.180448
Lowest Frequency = 16.0092cm-1

N       -3.366445    1.084606   -6.310570
K       -3.487513   -0.928669   -4.459485
Si       -1.771407    1.587042   -6.330737
Si       -4.576626    0.750937   -7.415878
C       -0.991204    1.868170   -8.024452
```

C	-1.522528	3.211537	-5.379782
C	-0.626526	0.361063	-5.441681
C	-4.138926	0.952015	-9.239151
C	-5.234069	-1.021179	-7.240946
C	-6.113811	1.831942	-7.141328
H	0.050638	2.190964	-7.921391
H	-1.522721	2.639966	-8.589366
H	-0.996751	0.953967	-8.625810
H	-0.483689	3.550780	-5.456257
H	-1.725915	3.105199	-4.306785
H	-2.149728	4.018476	-5.776908
H	-0.674528	-0.640382	-5.885432
H	-0.847886	0.280370	-4.370042
H	0.417057	0.687552	-5.508707
H	-3.315316	0.293000	-9.530135
H	-3.838844	1.978958	-9.468783
H	-4.998954	0.708092	-9.872454
H	-4.439846	-1.767098	-7.363059
H	-5.987886	-1.232233	-8.007332
H	-5.729663	-1.190959	-6.276890
H	-6.864755	1.638514	-7.915108
H	-5.878204	2.901769	-7.190059
H	-6.608853	1.624582	-6.184306
K	-4.583759	2.586359	-4.368824
F	-4.626188	0.604570	-2.927194

18b_kf_khmds_wb97xd.log

SCF (RwB97XD) = -2173.27656306
E(SCF)+ZPE(0 K)= -2173.046065
H(298 K)= -2173.023825
G(298 K)= -2173.097323
Lowest Frequency = 14.9605cm-1

N	-3.372100	1.071529	-6.292686
K	-3.464643	-0.959340	-4.409459
Si	-1.786445	1.597303	-6.327412
Si	-4.567345	0.736956	-7.411601
C	-1.053979	1.967574	-8.026784
C	-1.532033	3.184501	-5.316361
C	-0.596606	0.355979	-5.521987
C	-4.077859	0.839573	-9.231449
C	-5.294085	-1.002037	-7.185197
C	-6.072849	1.880673	-7.229111
H	-0.016086	2.303236	-7.934805
H	-1.612144	2.755682	-8.540007
H	-1.061042	1.084584	-8.671514
H	-0.497167	3.531999	-5.395787
H	-1.719364	3.032683	-4.246831
H	-2.169392	4.002630	-5.669910
H	-0.656271	-0.631052	-5.993388
H	-0.778346	0.239075	-4.447015
H	0.440109	0.693837	-5.617633
H	-3.268735	0.143019	-9.468531
H	-3.740201	1.843176	-9.503688
H	-4.928580	0.590018	-9.873620
H	-4.531347	-1.782337	-7.284387
H	-6.054966	-1.204450	-7.945565
H	-5.793227	-1.122516	-6.216550
H	-6.799370	1.696383	-8.026751
H	-5.791529	2.937603	-7.290809
H	-6.611240	1.718580	-6.287863
K	-4.624435	2.555973	-4.305634
F	-4.603568	0.569073	-2.856518

18c_kf_khmds_b3lyp.log

SCF (RB3LYP) = -2173.51997011
E(SCF)+ZPE(0 K)= -2173.291875
H(298 K)= -2173.269070
G(298 K)= -2173.343797
Lowest Frequency = 15.8578cm-1

N	-3.364907	1.088022	-6.317463
K	-3.491636	-0.933273	-4.452478
Si	-1.768531	1.587561	-6.334037
Si	-4.577181	0.752026	-7.420331
C	-0.985583	1.864335	-8.031818
C	-1.520460	3.216275	-5.380947
C	-0.627089	0.357346	-5.436689
C	-4.139964	0.959649	-9.247262
C	-5.229708	-1.026742	-7.244591
C	-6.120018	1.830391	-7.136469
H	0.056382	2.184810	-7.932482
H	-1.516087	2.634576	-8.597680
H	-0.994766	0.949803	-8.630664
H	-0.482835	3.557285	-5.450444
H	-1.731071	3.108870	-4.310345
H	-2.146991	4.021615	-5.779733
H	-0.676077	-0.643312	-5.879754
H	-0.858543	0.276574	-4.368092
H	0.417407	0.679076	-5.494448
H	-3.315740	0.303834	-9.539604
H	-3.840327	1.986527	-9.472577
H	-4.997740	0.717600	-9.882772
H	-4.432628	-1.768166	-7.366740
H	-5.985036	-1.243832	-8.006268
H	-5.718899	-1.197019	-6.278378
H	-6.878154	1.636148	-7.901453
H	-5.887003	2.899764	-7.186067
H	-6.603323	1.622904	-6.174543
K	-4.585353	2.598345	-4.362735
F	-4.632986	0.608917	-2.911990

18d_kf_khmds_m062x.log

SCF (RM062X) = -2173.14628996
E(SCF)+ZPE(0 K)= -2172.917274
H(298 K)= -2172.894382
G(298 K)= -2172.970357
Lowest Frequency = 10.4634cm-1

N	-3.371123	1.114917	-6.320625
K	-3.498962	-0.872074	-4.457182
Si	-1.770745	1.595626	-6.327965
Si	-4.578102	0.747063	-7.416315
C	-1.008087	1.922776	-8.020828
C	-1.501036	3.182327	-5.322209
C	-0.635749	0.329871	-5.483564
C	-4.130627	0.925608	-9.239057
C	-5.218724	-1.027674	-7.210662
C	-6.126647	1.813643	-7.156556
H	0.034387	2.240225	-7.923141
H	-1.546051	2.710726	-8.554719
H	-1.024721	1.027077	-8.647378
H	-0.454607	3.497962	-5.371425
H	-1.725461	3.040936	-4.258598
H	-2.101877	4.016770	-5.699775
H	-0.723557	-0.663710	-5.935821
H	-0.833892	0.243940	-4.408978
H	0.412778	0.629332	-5.573129
H	-3.304723	0.264374	-9.514379
H	-3.829810	1.949141	-9.477807
H	-4.984301	0.673314	-9.875177
H	-4.416658	-1.767507	-7.304883
H	-5.959817	-1.262465	-7.980538
H	-5.723846	-1.177857	-6.249582
H	-6.879891	1.592152	-7.918463
H	-5.906418	2.884085	-7.230252
H	-6.607393	1.618974	-6.190900
K	-4.590396	2.668076	-4.449059
F	-4.628788	0.712279	-3.009884

18e_kf_khmds_m06l.log

SCF (RM06L) = -2173.30170673
E(SCF)+ZPE(0 K)= -2173.071281
H(298 K)= -2173.049570

```
G(298 K)=              -2173.120391
Lowest Frequency = -6.6645cm-1

N       -3.399752    1.181808   -6.342706
K       -3.559188   -0.792356   -4.406103
Si       -1.787249    1.598743   -6.330229
Si       -4.584721    0.759688   -7.433752
C       -0.986767    1.929199   -8.003405
C       -1.466468    3.151065   -5.290081
C       -0.695510    0.290080   -5.493652
C       -4.169882    0.967013   -9.259721
C       -5.145161   -1.043878   -7.241009
C       -6.182371    1.741413   -7.154401
H        0.057487    2.231356   -7.890668
H       -1.494555    2.727075   -8.548905
H       -0.996396    1.045689   -8.644631
H       -0.406891    3.417828   -5.297994
H       -1.726459    3.007229   -4.236422
H       -2.005511    4.029571   -5.656273
H       -0.820223   -0.706625   -5.926696
H       -0.873304    0.215803   -4.416082
H        0.363563    0.539244   -5.595849
H       -3.334669    0.333991   -9.565528
H       -3.897974    1.996171   -9.502379
H       -5.020722    0.701873   -9.892396
H       -4.314010   -1.752478   -7.301155
H       -5.847935   -1.325327   -8.029070
H       -5.675262   -1.225052   -6.300528
H       -6.948388    1.464162   -7.882710
H       -6.041146    2.821399   -7.254711
H       -6.625898    1.548154   -6.172358
K       -4.590790    2.830138   -4.495385
F       -4.458693    0.946932   -2.964052

18f_kf_khmds_bp86.log

SCF (RB-P86) =         -2173.57882564
E(SCF)+ZPE(0 K)=       -2173.356932
H(298 K)=              -2173.333731
G(298 K)=              -2173.409077
Lowest Frequency = 16.0013cm-1

N       -3.373488    1.066844   -6.284576
K       -3.425141   -0.954914   -4.442808
Si       -1.783268    1.626640   -6.323393
Si       -4.568665    0.710684   -7.419767
C       -1.109951    2.165620   -8.012946
C       -1.537557    3.129823   -5.170991
C       -0.550497    0.330050   -5.656324
C       -4.027264    0.637734   -9.236472
C       -5.388734   -0.975404   -7.059834
C       -6.020121    1.950906   -7.357784
H       -0.081413    2.554340   -7.922651
H       -1.729587    2.963614   -8.453204
H       -1.087628    1.328397   -8.728078
H       -0.491958    3.479872   -5.202694
H       -1.744073    2.888437   -4.112868
H       -2.164823    3.989379   -5.466177
H       -0.631595   -0.626816   -6.199941
H       -0.687341    0.129879   -4.578446
H        0.490818    0.677036   -5.767704
H       -3.217323   -0.095215   -9.382654
H       -3.662146    1.613423   -9.593640
H       -4.867687    0.338935   -9.885794
H       -4.671343   -1.812320   -7.126082
H       -6.185663   -1.187795   -7.792303
H       -5.872682   -1.002272   -6.067227
H       -6.738906    1.761986   -8.173184
H       -5.670800    2.991583   -7.470142
H       -6.602889    1.879039   -6.421857
K       -4.640047    2.525059   -4.342488
F       -4.593071    0.545364   -2.876823

19a_difluoroethene_pbe0.log

SCF (RPBE1PBE) =       -276.908765176
E(SCF)+ZPE(0 K)=       -276.872126
H(298 K)=              -276.867475
G(298 K)=              -276.898173
Lowest Frequency = 444.6959cm-1

C       -2.774232    0.238949   -0.000000
H       -2.227631   -0.692715    0.000000
H       -3.854457    0.246092    0.000000
C       -2.118163    1.375562   -0.000000
F       -0.806699    1.500298    0.000000
F       -2.666061    2.573613    0.000000

19b_difluoroethene_wb97xd.log

SCF (RwB97XD) =        -277.108117328
E(SCF)+ZPE(0 K)=       -277.071356
H(298 K)=              -277.066720
G(298 K)=              -277.097395
Lowest Frequency = 452.2489cm-1

C       -2.773975    0.239447   -0.000000
H       -2.228368   -0.691725    0.000000
H       -3.853261    0.245838    0.000000
C       -2.118477    1.375062   -0.000000
F       -0.806366    1.499566    0.000000
F       -2.666795    2.573611    0.000000

19c_difluoroethene_b3lyp.log

SCF (RB3LYP) =         -277.198234596
E(SCF)+ZPE(0 K)=       -277.161927
H(298 K)=              -277.157254
G(298 K)=              -277.187997
Lowest Frequency = 444.5970cm-1

C       -2.775242    0.237158    0.000000
H       -2.231414   -0.694717   -0.000000
H       -3.854271    0.241885   -0.000000
C       -2.118968    1.374148    0.000000
F       -0.797704    1.501402   -0.000000
F       -2.669644    2.581923   -0.000000

19d_difluoroethene_m062x.log

SCF (RM062X) =         -277.087298731
E(SCF)+ZPE(0 K)=       -277.050373
H(298 K)=              -277.045736
G(298 K)=              -277.076415
Lowest Frequency = 444.9689cm-1

C       -2.774731    0.237989    0.000000
H       -2.226176   -0.690789   -0.000000
H       -3.853451    0.248571   -0.000000
C       -2.119851    1.372501    0.000000
F       -0.806862    1.500265   -0.000000
F       -2.666171    2.573263   -0.000000

19e_difluoroethene_m06l.log

SCF (RM06L) =          -277.144500846
E(SCF)+ZPE(0 K)=       -277.108078
H(298 K)=              -277.103405
G(298 K)=              -277.134133
Lowest Frequency = 440.3626cm-1

C       -2.774281    0.238701   -0.000000
H       -2.232185   -0.692216    0.000000
H       -3.851602    0.242851    0.000000
C       -2.117666    1.376193   -0.000000
F       -0.806939    1.502223    0.000000
F       -2.664568    2.574047    0.000000
```

19f_difluoroethene_bp86.log

SCF (RB-P86) = -277.193453600
E(SCF)+ZPE(0 K)= -277.158329
H(298 K)= -277.153573
G(298 K)= -277.184458
Lowest Frequency = 429.4888cm-1

C	-2.778285	0.231912	-0.000000
H	-2.229421	-0.706996	0.000000
H	-3.865923	0.237391	0.000000
C	-2.116566	1.378339	-0.000000
F	-0.784951	1.505887	0.000000
F	-2.672095	2.595266	0.000000

KHMDS solvation
khmds_dimer_1thf_b3lyp.log

SCF (RB3LYP) = -3412.34824658
E(SCF)+ZPE(0 K)= -3411.656726
H(298 K)= -3411.605340
G(298 K)= -3411.744465
Lowest Frequency = 17.5213cm-1

K	5.509374	12.605450	1.934489
Si	8.591336	10.722142	0.784380
Si	6.097856	9.085611	1.328500
N	6.937425	10.488688	0.947744
C	8.957811	12.517073	0.285002
H	10.021643	12.654881	0.068620
H	8.712797	13.201394	1.103551
H	8.407399	12.859138	-0.595396
C	9.608039	10.452922	2.359833
H	9.586952	9.407333	2.677241
H	9.215677	11.055511	3.185128
H	10.656629	10.733128	2.213955
C	9.390993	9.608383	-0.529826
H	8.885006	9.679225	-1.496908
H	9.337415	8.561113	-0.216075
H	10.447078	9.847261	-0.692001
C	4.471406	9.488502	2.222803
H	3.829975	10.159067	1.643747
H	4.656718	9.945747	3.200587
H	3.888241	8.580765	2.405463
C	5.605042	8.075791	-0.204179
H	4.913435	8.613487	-0.860605
H	5.105466	7.141879	0.073507
H	6.489009	7.812563	-0.793273
C	7.000209	7.864904	2.460938
H	6.367603	7.004045	2.700862
H	7.286844	8.339565	3.403637
H	7.911759	7.482112	1.993184
C	3.160976	12.572296	4.756974
O	4.106165	13.418643	4.065810
C	3.616337	14.777094	4.031960
C	2.455228	14.826387	5.015369
C	1.896676	13.403523	4.925612
H	3.005780	11.668541	4.165780
H	3.589708	12.288958	5.722861
H	4.437969	15.443640	4.296577
H	3.286761	15.009196	3.014765
H	2.820538	15.029144	6.024333
H	1.726220	15.592021	4.752254
H	1.326959	13.109742	5.806540
H	1.255880	13.300773	4.048426
K	6.002594	11.383725	-1.542626
Si	2.853699	12.840171	-0.608614
Si	5.120087	14.920378	-0.759924
N	4.423890	13.416541	-0.509080
C	2.815603	11.042400	-1.227390
H	1.792301	10.655026	-1.220696
H	3.403596	10.369173	-0.597958
H	3.172366	10.953824	-2.260142
C	1.963579	12.802499	1.067504
H	1.894910	13.811422	1.483298
H	2.478577	12.176989	1.801656
H	0.944796	12.412243	0.975871
C	1.682151	13.769817	-1.771213
H	2.110671	13.867286	-2.773208
H	1.460372	14.775608	-1.407167
H	0.730447	13.237094	-1.868763
C	6.193777	15.473461	0.712087
H	7.035507	14.799342	0.898149
H	5.609760	15.568295	1.633760
H	6.630517	16.457581	0.516366
C	6.297600	14.926024	-2.245666
H	7.104529	14.197614	-2.130759
H	6.768493	15.903756	-2.389120
H	5.756709	14.681668	-3.164391
C	3.929890	16.366243	-1.044045
H	4.478551	17.310985	-1.117057
H	3.215201	16.462866	-0.221361
H	3.357973	16.245188	-1.967018
C	7.230965	12.308340	-4.784937
O	7.721152	11.733777	-3.561121
C	9.144186	11.913526	-3.565273
C	9.351226	13.308961	-4.137475
C	8.195805	13.451371	-5.150649
H	7.215480	11.536980	-5.561290
H	6.209585	12.644528	-4.607372
H	9.501114	11.792146	-2.545693
H	9.607900	11.147542	-4.198503
H	9.257670	14.051095	-3.344589
H	10.332162	13.422361	-4.597824
H	7.713494	14.423523	-5.065318
H	8.547409	13.338809	-6.175762

khmds_dimer_1thf_b3pw91.log

SCF (RB3PW91) = -3411.63057419
E(SCF)+ZPE(0 K)= -3410.940756
H(298 K)= -3410.888587
G(298 K)= -3411.031357
Lowest Frequency = 17.0347cm-1

K	5.520143	12.579202	1.958063
Si	8.582924	10.695340	0.765255
Si	6.100100	9.074342	1.319362
N	6.928217	10.484805	0.943703
C	8.965013	12.480837	0.257795
H	10.026709	12.603075	0.020188
H	8.748019	13.164507	1.085450
H	8.401093	12.836197	-0.609736
C	9.606403	10.426865	2.331373
H	9.584317	9.381347	2.650806
H	9.219438	11.033002	3.157298
H	10.655096	10.704406	2.177717
C	9.357905	9.567370	-0.545191
H	8.845263	9.636873	-1.509543
H	9.294149	8.522700	-0.222857
H	10.416047	9.793169	-0.715615
C	4.474771	9.462995	2.213075
H	3.834037	10.143655	1.643648
H	4.658706	9.903020	3.199604
H	3.891275	8.551593	2.378783
C	5.618193	8.062713	-0.210267
H	4.920535	8.592644	-0.867347
H	5.128320	7.123804	0.069859
H	6.505351	7.807823	-0.799117
C	7.008468	7.862615	2.449731
H	6.380751	6.997702	2.690352
H	7.291563	8.341310	3.392136
H	7.922570	7.485482	1.981066
C	3.211454	12.676120	4.812942
O	4.123343	13.498192	4.069411
C	3.596913	14.829465	3.954053
C	2.415479	14.898602	4.905642

C	1.913698	13.457954	4.894269
H	3.106085	11.717786	4.299215
H	3.632508	12.492438	5.808022
H	4.390840	15.538249	4.199234
H	3.283664	14.998603	2.918110
H	2.749708	15.177704	5.908167
H	1.665896	15.619926	4.579232
H	1.329305	13.197259	5.777106
H	1.303749	13.272918	4.007360
K	6.008992	11.389132	-1.524719
Si	2.870027	12.807205	-0.596522
Si	5.105394	14.898635	-0.715869
N	4.436497	13.384321	-0.458097
C	2.845666	11.015274	-1.220589
H	1.823284	10.623936	-1.214739
H	3.435476	10.343419	-0.589920
H	3.202240	10.929660	-2.254071
C	1.945277	12.762571	1.054444
H	1.866080	13.771510	1.469699
H	2.449073	12.135488	1.796129
H	0.929326	12.369606	0.940134
C	1.728219	13.735779	-1.782792
H	2.190376	13.852483	-2.768290
H	1.480213	14.733424	-1.411534
H	0.787542	13.191148	-1.919640
C	6.249375	15.429090	0.703460
H	7.091983	14.744740	0.844805
H	5.712285	15.528027	1.653122
H	6.686121	16.409913	0.488869
C	6.200071	14.944493	-2.258228
H	7.021425	14.224430	-2.194551
H	6.650583	15.930826	-2.411360
H	5.612923	14.705471	-3.150227
C	3.893531	16.336865	-0.904610
H	4.430178	17.290610	-0.954664
H	3.202696	16.390211	-0.057158
H	3.296250	16.246411	-1.815560
C	7.245725	12.352067	-4.770318
O	7.720278	11.752089	-3.562478
C	9.136308	11.921799	-3.562808
C	9.349431	13.332855	-4.080298
C	8.201632	13.510748	-5.088123
H	7.250757	11.602988	-5.570585
H	6.216218	12.672132	-4.602917
H	9.497662	11.758113	-2.549158
H	9.596459	11.178266	-4.227173
H	9.253135	14.045528	-3.259487
H	10.333801	13.463035	-4.530825
H	7.713657	14.477293	-4.965854
H	8.556437	13.441620	-6.116999

khmds_dimer_1thf_m062x.log

SCF (RM062X) = -3411.45945931
E(SCF)+ZPE(0 K)= -3410.764665
H(298 K)= -3410.713725
G(298 K)= -3410.850830
Lowest Frequency = 18.4181cm-1

K	5.557788	12.695493	1.862308
Si	8.555081	10.726961	0.618189
Si	6.061009	9.195846	1.449612
N	6.915902	10.537830	0.918402
C	8.908851	12.520998	0.118553
H	9.950767	12.659521	-0.186224
H	8.741923	13.182148	0.975824
H	8.280078	12.885671	-0.702325
C	9.702219	10.398576	2.082039
H	9.689113	9.344716	2.372114
H	9.404834	10.986753	2.955180
H	10.736572	10.661747	1.839398
C	9.198638	9.605962	-0.768603
H	8.628163	9.708463	-1.697337
H	9.118474	8.560069	-0.453852
H	10.250567	9.795579	-1.003915
C	4.508426	9.729739	2.397101
H	3.858769	10.386844	1.807730
H	4.776645	10.252925	3.321809
H	3.902575	8.865499	2.684854
C	5.473444	8.070673	0.042404
H	4.802836	8.571845	-0.662365
H	4.932091	7.201011	0.427929
H	6.332201	7.697507	-0.524772
C	6.994717	8.048804	2.625431
H	6.349097	7.242372	2.986663
H	7.368256	8.593814	3.497081
H	7.853294	7.585402	2.130172
C	2.948037	12.327168	4.647875
O	4.019595	13.044382	4.015827
C	3.730321	14.449683	4.003486
C	2.569336	14.642001	4.966786
C	1.810666	13.326490	4.798370
H	2.689329	11.467811	4.027655
H	3.296129	11.967581	5.620301
H	4.630542	14.992095	4.296722
H	3.448951	14.747850	2.986591
H	2.940451	14.738770	5.988700
H	1.974526	15.521079	4.725103
H	1.163798	13.088989	5.641153
H	1.208511	13.351057	3.888281
K	5.942581	11.342536	-1.556904
Si	2.865723	12.849760	-0.599167
Si	5.124490	14.923726	-0.855461
N	4.439785	13.422593	-0.563362
C	2.822133	11.027401	-1.123660
H	1.805354	10.629800	-1.057076
H	3.444144	10.397983	-0.478857
H	3.142142	10.888348	-2.162876
C	2.007776	12.907704	1.088197
H	1.846768	13.948537	1.386641
H	2.595408	12.425839	1.876297
H	1.029895	12.416382	1.064342
C	1.678044	13.727241	-1.777250
H	2.088013	13.783484	-2.790067
H	1.463112	14.746735	-1.447695
H	0.725644	13.190774	-1.833347
C	6.228388	15.497754	0.579578
H	7.079677	14.829361	0.746146
H	5.668082	15.596006	1.516315
H	6.650930	16.483327	0.363140
C	6.255814	14.912901	-2.370456
H	7.005344	14.116562	-2.312220
H	6.795252	15.858133	-2.487661
H	5.667438	14.751293	-3.279092
C	3.915645	16.348372	-1.134146
H	4.451713	17.297474	-1.231907
H	3.215016	16.447790	-0.299320
H	3.329491	16.204498	-2.045339
C	7.338546	12.370485	-4.588669
O	7.836962	11.784982	-3.382956
C	9.251059	11.965858	-3.410653
C	9.431395	13.389250	-3.914135
C	8.279879	13.541060	-4.925284
H	7.347111	11.616457	-5.381206
H	6.308371	12.682193	-4.412307
H	9.637149	11.791963	-2.409173
H	9.697799	11.239549	-4.100295
H	9.308723	14.087040	-3.084286
H	10.411695	13.550501	-4.359282
H	7.778749	14.501297	-4.817279
H	8.636856	13.457148	-5.950667

khmds_dimer_1thf_m06l.log

SCF (RM06L) = -3411.87393273
E(SCF)+ZPE(0 K)= -3411.179383
H(298 K)= -3411.127851
G(298 K)= -3411.267071

Lowest Frequency = 20.6700cm-1

K	5.550703	12.659696	1.938904
Si	8.569194	10.699971	0.676932
Si	6.088475	9.143581	1.421519
N	6.929625	10.512220	0.952026
C	8.928574	12.485936	0.159460
H	9.971957	12.624224	-0.135918
H	8.758441	13.168946	0.996851
H	8.318202	12.851294	-0.672655
C	9.704055	10.396694	2.153814
H	9.716540	9.347239	2.453970
H	9.397849	10.976874	3.027402
H	10.736843	10.675877	1.928439
C	9.247801	9.573748	-0.686775
H	8.691799	9.638238	-1.625994
H	9.196493	8.528840	-0.368136
H	10.295791	9.779544	-0.921206
C	4.524370	9.618540	2.378353
H	3.862890	10.287919	1.819642
H	4.769201	10.107301	3.326349
H	3.923837	8.741818	2.632100
C	5.517212	8.077750	-0.036640
H	4.832680	8.589788	-0.717967
H	4.992534	7.179065	0.298535
H	6.371181	7.741207	-0.630632
C	7.016659	7.949491	2.550137
H	6.385203	7.112928	2.860551
H	7.370104	8.441188	3.459179
H	7.891258	7.520778	2.054863
C	2.917649	12.455336	4.745168
O	3.977772	13.156996	4.075054
C	3.667025	14.558329	4.022123
C	2.498168	14.763816	4.959540
C	1.769148	13.433883	4.845815
H	2.680725	11.555406	4.174090
H	3.272230	12.143864	5.734133
H	4.559312	15.122062	4.303370
H	3.405252	14.828910	2.990041
H	2.853349	14.917894	5.979845
H	1.888509	15.621716	4.683820
H	1.114798	13.222826	5.689071
H	1.166303	13.406410	3.936485
K	5.985004	11.318750	-1.562721
Si	2.888938	12.833584	-0.605539
Si	5.124844	14.904530	-0.796172
N	4.461653	13.394533	-0.518676
C	2.845596	11.009244	-1.116594
H	1.830464	10.609769	-1.058391
H	3.455768	10.374500	-0.468119
H	3.170014	10.848482	-2.149607
C	1.978367	12.902380	1.051179
H	1.824211	13.939794	1.360144
H	2.524850	12.405134	1.857192
H	0.991520	12.433991	1.004032
C	1.737529	13.700038	-1.825071
H	2.182252	13.785809	-2.819616
H	1.474869	14.708620	-1.501413
H	0.800400	13.148475	-1.938647
C	6.264690	15.464932	0.613274
H	7.114780	14.796368	0.773555
H	5.737340	15.579724	1.565006
H	6.696059	16.443497	0.389318
C	6.217611	14.927863	-2.337672
H	6.981400	14.144864	-2.311332
H	6.745772	15.876341	-2.467958
H	5.620165	14.767065	-3.238875
C	3.910655	16.334046	-1.008670
H	4.434121	17.291902	-1.068071
H	3.211669	16.402629	-0.170714
H	3.315043	16.239482	-1.918298
C	7.312360	12.384252	-4.660579
O	7.834017	11.787203	-3.467991
C	9.247874	11.980740	-3.523018
C	9.407816	13.398254	-4.023859
C	8.244917	13.546452	-5.011000
H	7.287584	11.634611	-5.459310
H	6.284416	12.690224	-4.456331
H	9.651856	11.799979	-2.528950
H	9.693772	11.253688	-4.214768
H	9.298802	14.094789	-3.191195
H	10.380102	13.576327	-4.478904
H	7.748638	14.509307	-4.905567
H	8.583169	13.467512	-6.042878

khmds_dimer_1thf_pbe0.log

SCF (RPBE1PBE) = -3410.01758735
E(SCF)+ZPE(0 K)= -3409.327051
H(298 K)= -3409.274669
G(298 K)= -3409.419347
Lowest Frequency = 9.9524cm-1

K	5.506332	12.581870	1.963729
Si	8.618747	10.694383	0.778582
Si	6.130034	9.085942	1.350792
N	6.961895	10.493129	0.964172
C	9.013357	12.466922	0.237297
H	10.080224	12.576625	0.013991
H	8.785782	13.174366	1.042508
H	8.467123	12.801479	-0.650822
C	9.646594	10.445506	2.345020
H	9.610169	9.407597	2.689220
H	9.276578	11.076972	3.160143
H	10.698842	10.701989	2.177339
C	9.392194	9.546421	-0.515914
H	8.882292	9.606062	-1.483338
H	9.328256	8.504504	-0.182938
H	10.451286	9.770398	-0.686177
C	4.501679	9.483780	2.235903
H	3.857630	10.151898	1.653360
H	4.678585	9.938288	3.217917
H	3.922719	8.571017	2.412838
C	5.644057	8.061286	-0.168629
H	4.952817	8.590223	-0.834516
H	5.144026	7.130412	0.121912
H	6.529987	7.789485	-0.752780
C	7.028602	7.878619	2.493501
H	6.393518	7.019448	2.736950
H	7.311216	8.360940	3.434787
H	7.942499	7.491019	2.031624
C	3.153140	12.651058	4.792957
O	4.056806	13.479749	4.053932
C	3.550380	14.818348	3.994065
C	2.396915	14.874941	4.977549
C	1.870056	13.446716	4.924370
H	3.023461	11.707269	4.256770
H	3.591413	12.434786	5.774591
H	4.360959	15.508999	4.239284
H	3.211165	15.026022	2.972300
H	2.762990	15.108281	5.981157
H	1.653741	15.622964	4.698961
H	1.295902	13.162960	5.807208
H	1.242537	13.303467	4.040832
K	6.019964	11.401381	-1.507038
Si	2.862107	12.823110	-0.632943
Si	5.096099	14.922012	-0.696154
N	4.426406	13.402317	-0.464050
C	2.851513	11.036828	-1.274465
H	1.826934	10.650679	-1.301843
H	3.419652	10.354609	-0.633017
H	3.236591	10.958628	-2.298876
C	1.906147	12.756806	0.999852
H	1.818134	13.759892	1.429540
H	2.393881	12.116919	1.742711
H	0.891969	12.367031	0.857358
C	1.735074	13.759870	-1.826867
H	2.208097	13.886852	-2.806455

H	1.479199	14.754128	-1.450033
H	0.797291	13.213846	-1.979812
C	6.235090	15.427849	0.736818
H	7.079647	14.742713	0.870250
H	5.694268	15.511222	1.686920
H	6.671078	16.413295	0.540351
C	6.195529	15.001476	-2.234192
H	7.016886	14.278514	-2.188185
H	6.646813	15.991927	-2.360286
H	5.609947	14.787180	-3.134438
C	3.887115	16.365219	-0.864048
H	4.427423	17.317986	-0.898786
H	3.194251	16.409080	-0.016925
H	3.290813	16.291047	-1.777853
C	7.218447	12.363468	-4.779620
O	7.699672	11.773774	-3.574955
C	9.117611	11.866516	-3.638096
C	9.385777	13.255477	-4.188875
C	8.188532	13.499519	-5.121770
H	7.204135	11.604854	-5.571941
H	6.195042	12.700948	-4.605444
H	9.516528	11.697418	-2.638319
H	9.507262	11.089467	-4.310189
H	9.392842	13.982661	-3.374472
H	10.345174	13.314213	-4.704325
H	7.739057	14.475731	-4.938085
H	8.476121	13.455221	-6.173039

khmds_dimer_1thf_wb97xd.log

SCF (RwB97XD) = -3411.75941412
E(SCF)+ZPE(0 K)= -3411.064779
H(298 K)= -3411.013321
G(298 K)= -3411.153349
Lowest Frequency = 15.0091cm-1

K	5.542502	12.652777	1.908197
Si	8.550501	10.708002	0.730191
Si	6.063979	9.092849	1.357017
N	6.905669	10.479525	0.939814
C	8.902122	12.514879	0.272272
H	9.958112	12.662921	0.025155
H	8.685006	13.173680	1.120185
H	8.320143	12.882704	-0.579317
C	9.624361	10.384237	2.250998
H	9.604953	9.328455	2.535219
H	9.274656	10.964198	3.110956
H	10.668589	10.658334	2.067507
C	9.297768	9.631487	-0.639411
H	8.764859	9.729673	-1.590362
H	9.244951	8.576713	-0.349503
H	10.350797	9.866048	-0.825918
C	4.470493	9.520925	2.289926
H	3.818384	10.190574	1.719072
H	4.691065	9.993161	3.253642
H	3.884803	8.621707	2.504596
C	5.523008	8.069940	-0.145450
H	4.817036	8.603456	-0.790798
H	5.025876	7.143321	0.159627
H	6.389590	7.792285	-0.754430
C	6.984927	7.877315	2.473906
H	6.349782	7.027423	2.744058
H	7.308384	8.359516	3.401205
H	7.876291	7.477926	1.980435
C	3.175020	12.524546	4.732884
O	4.136941	13.369112	4.090237
C	3.666518	14.721441	4.062391
C	2.464465	14.767225	4.992558
C	1.906586	13.352117	4.859074
H	3.042055	11.623468	4.130347
H	3.562480	12.232724	5.714422
H	4.479613	15.378321	4.376112
H	3.383109	14.979806	3.035997
H	2.785878	14.954997	6.019140
H	1.752865	15.540350	4.706087
H	1.299966	13.046666	5.710527
H	1.305263	13.263273	3.952552
K	5.969816	11.350119	-1.580245
Si	2.834840	12.850305	-0.565072
Si	5.099764	14.907682	-0.864634
N	4.407770	13.417990	-0.549526
C	2.756079	11.043830	-1.140002
H	1.732836	10.662086	-1.071109
H	3.374037	10.383510	-0.523333
H	3.060821	10.928977	-2.186796
C	2.021496	12.850875	1.145842
H	1.967731	13.869882	1.540547
H	2.568111	12.242887	1.873481
H	1.000419	12.457648	1.106263
C	1.614724	13.763039	-1.683957
H	1.989067	13.834463	-2.709816
H	1.421474	14.779090	-1.330291
H	0.655078	13.236725	-1.717255
C	6.193688	15.519490	0.563528
H	7.049732	14.861325	0.748040
H	5.630822	15.633765	1.497027
H	6.611203	16.503186	0.327105
C	6.258517	14.855231	-2.359545
H	7.056933	14.117606	-2.232137
H	6.741823	15.821807	-2.534570
H	5.705134	14.592670	-3.266506
C	3.909812	16.338464	-1.196230
H	4.459726	17.276256	-1.326407
H	3.209919	16.478352	-0.366586
H	3.320764	16.172288	-2.102058
C	7.375045	12.313318	-4.715301
O	7.838746	11.767154	-3.482234
C	9.242832	11.994253	-3.444707
C	9.417672	13.400347	-3.996899
C	8.281108	13.514365	-5.028679
H	7.445884	11.549694	-5.497414
H	6.326052	12.584763	-4.589648
H	9.579842	11.875605	-2.417139
H	9.752579	11.252413	-4.072469
H	9.284648	14.130405	-3.197526
H	10.403983	13.548710	-4.434608
H	7.744472	14.455715	-4.921683
H	8.656256	13.453456	-6.049716

khmds_dimer_2thf_b3lyp.log

SCF (RB3LYP) = -3877.51624465
E(SCF)+ZPE(0 K)= -3876.590477
H(298 K)= -3876.525064
G(298 K)= -3876.699439
Lowest Frequency = 14.2794cm-1

K	5.446070	12.675813	1.952786
Si	8.513600	10.671395	0.990408
Si	6.019423	9.107300	1.803147
N	6.873220	10.419759	1.205072
C	8.834872	12.479957	0.509327
H	9.897214	12.664694	0.323425
H	8.529580	13.148613	1.317727
H	8.294908	12.792333	-0.388904
C	9.613367	10.352796	2.502045
H	9.671506	9.284979	2.728390
H	9.227190	10.855018	3.391798
H	10.635220	10.708733	2.333759
C	9.277029	9.604680	-0.386686
H	8.778626	9.740812	-1.349997
H	9.191354	8.544811	-0.126448
H	10.340117	9.820274	-0.537004
C	4.430670	9.664851	2.682969
H	3.768797	10.244573	2.033511
H	4.657187	10.270012	3.566069
H	3.852690	8.802280	3.028608
C	5.464572	7.881084	0.466340

H	4.870927	8.356842	-0.318500
H	4.855984	7.075691	0.891240
H	6.331818	7.418170	-0.013455
C	6.933197	8.048667	3.085658
H	6.288527	7.251537	3.470928
H	7.262953	8.649233	3.938213
H	7.819190	7.573693	2.654673
C	2.756928	12.399764	4.745787
O	3.846282	13.084662	4.098157
C	3.672361	14.511477	4.220019
C	2.544660	14.713280	5.223954
C	1.694733	13.458651	5.005319
H	2.418372	11.594316	4.094308
H	3.122809	11.961867	5.680443
H	4.622320	14.945742	4.532195
H	3.404510	14.919685	3.239574
H	2.940349	14.731245	6.241887
H	1.999438	15.640455	5.050179
H	1.063271	13.211019	5.858100
H	1.059165	13.578431	4.125666
K	5.987906	11.185409	-1.391186
Si	2.785930	12.696502	-0.594704
Si	4.982875	14.813927	-0.919443
N	4.347015	13.302995	-0.576668
C	2.775075	10.824919	-0.915924
H	1.761112	10.424498	-0.818954
H	3.397878	10.271465	-0.208582
H	3.114918	10.577347	-1.926518
C	1.870189	12.922435	1.053340
H	1.793813	13.983788	1.307215
H	2.384794	12.423936	1.878090
H	0.853603	12.517320	1.014017
C	1.623933	13.414683	-1.911105
H	2.081981	13.378572	-2.904482
H	1.369893	14.456731	-1.702579
H	0.686705	12.850020	-1.957555
C	6.107777	15.461539	0.471552
H	6.961571	14.801937	0.647422
H	5.572802	15.589874	1.418135
H	6.519878	16.440343	0.206769
C	6.095675	14.794267	-2.453324
H	6.910993	14.075299	-2.343067
H	6.547788	15.772490	-2.646604
H	5.522555	14.512277	-3.341287
C	3.732644	16.209150	-1.215429
H	4.243174	17.169528	-1.343262
H	3.041179	16.313369	-0.373802
H	3.135698	16.032003	-2.113522
C	7.383428	12.135046	-4.610588
O	7.844523	11.691240	-3.325483
C	9.255446	11.936031	-3.296100
C	9.423593	13.291668	-3.970134
C	8.295350	13.305165	-5.023557
H	7.457147	11.309250	-5.324799
H	6.336029	12.414705	-4.508276
H	9.579775	11.910337	-2.259265
H	9.777102	11.146326	-3.851057
H	9.274639	14.087685	-3.240220
H	10.413439	13.412208	-4.409302
H	7.757879	14.251697	-5.014329
H	8.684081	13.148582	-6.029427
C	4.854172	9.366910	-4.322859
O	5.388237	9.105914	-3.008648
C	6.431795	8.124445	-3.108479
C	7.007976	8.307465	-4.501433
C	5.751386	8.623750	-5.321461
H	3.817921	9.024133	-4.362716
H	4.865075	10.447464	-4.480651
H	7.145601	8.307209	-2.306367
H	6.009591	7.121516	-2.977546
H	7.698843	9.151508	-4.505373
H	7.535570	7.422542	-4.856403
H	5.959982	9.222611	-6.207175
H	5.271560	7.698845	-5.645127
C	8.214074	14.762817	3.699475
O	7.106537	13.850337	3.731281
C	7.348501	12.829970	4.725390
C	8.732633	13.112244	5.313264
C	9.396763	13.972160	4.232866
H	8.334175	15.110059	2.673835
H	7.995199	15.627369	4.337514
H	6.554089	12.868749	5.472467
H	7.309609	11.858111	4.227073
H	8.642868	13.678632	6.242076
H	9.280081	12.194897	5.526002
H	10.186966	14.613242	4.622947
H	9.814995	13.344768	3.444015

khmds_dimer_2thf_b3pw91.log

SCF (RB3PW91) = -3876.61943819
E(SCF)+ZPE(0 K)= -3875.693540
H(298 K)= -3875.627912
G(298 K)= -3875.803601
Lowest Frequency = 13.0142cm-1

K	5.456518	12.678357	1.970063
Si	8.536038	10.680314	0.975305
Si	6.074261	9.137679	1.884564
N	6.902948	10.450067	1.254473
C	8.852453	12.489723	0.514221
H	9.909612	12.677681	0.301631
H	8.569232	13.142534	1.344673
H	8.285329	12.818900	-0.361959
C	9.701303	10.315570	2.419767
H	9.755889	9.241603	2.618897
H	9.370931	10.802831	3.340116
H	10.717978	10.661964	2.203821
C	9.224533	9.629129	-0.447339
H	8.701060	9.790876	-1.393906
H	9.126738	8.566533	-0.200577
H	10.286916	9.827030	-0.627283
C	4.506218	9.692562	2.794898
H	3.821467	10.256852	2.154209
H	4.747764	10.310578	3.665909
H	3.945641	8.827712	3.164251
C	5.496355	7.901410	0.572755
H	4.876357	8.369575	-0.197324
H	4.906585	7.091753	1.016794
H	6.355946	7.445089	0.071999
C	7.022860	8.098057	3.149820
H	6.388143	7.307884	3.566088
H	7.381037	8.710471	3.982842
H	7.893280	7.614907	2.695560
C	2.669070	12.474561	4.678098
O	3.758241	13.148124	4.036736
C	3.563923	14.567388	4.106519
C	2.417820	14.787742	5.078207
C	1.589473	13.522753	4.875826
H	2.355206	11.640242	4.047995
H	3.015907	12.072238	5.637390
H	4.501205	15.028236	4.424686
H	3.310295	14.941790	3.107575
H	2.792376	14.837511	6.104045
H	1.867760	15.705307	4.867330
H	0.936156	13.292412	5.718004
H	0.977401	13.609054	3.974667
K	6.008813	11.174151	-1.338749
Si	2.824605	12.673501	-0.607122
Si	4.985129	14.804491	-0.872918
N	4.385688	13.276439	-0.540153
C	2.827824	10.789895	-0.812272
H	1.813206	10.391959	-0.707228
H	3.440582	10.287655	-0.058113
H	3.185694	10.477241	-1.798534
C	1.825854	12.994475	0.968685
H	1.728387	14.069207	1.150319
H	2.312311	12.555053	1.843852

H	0.816139	12.573947	0.908757
C	1.748025	13.308161	-2.028595
H	2.271063	13.220082	-2.986653
H	1.478868	14.359207	-1.893377
H	0.816577	12.736147	-2.104245
C	6.151709	15.433955	0.485556
H	7.004577	14.765429	0.635944
H	5.644001	15.568293	1.446722
H	6.564896	16.409651	0.209207
C	6.031119	14.834089	-2.447266
H	6.855883	14.119116	-2.380941
H	6.465623	15.820945	-2.639221
H	5.422749	14.562331	-3.315458
C	3.705197	16.183081	-1.080427
H	4.193634	17.159437	-1.173063
H	3.027840	16.232329	-0.221824
H	3.095543	16.034314	-1.975890
C	7.362670	12.166822	-4.566802
O	7.839360	11.740207	-3.290447
C	9.239546	12.001235	-3.281701
C	9.378330	13.357076	-3.951308
C	8.248928	13.345657	-4.994571
H	7.442691	11.338338	-5.279605
H	6.310268	12.430557	-4.458847
H	9.582127	11.975938	-2.249139
H	9.765740	11.220710	-3.848011
H	9.215129	14.148448	-3.217709
H	10.364486	13.500695	-4.393996
H	7.695647	14.284358	-4.990770
H	8.634552	13.187532	-6.002452
C	4.830247	9.381138	-4.284569
O	5.371430	9.116621	-2.983110
C	6.382337	8.115586	-3.095491
C	6.969657	8.313752	-4.476743
C	5.724014	8.655262	-5.293318
H	3.795124	9.029780	-4.324512
H	4.828731	10.463995	-4.433917
H	7.096457	8.261506	-2.284375
H	5.935643	7.118511	-2.989363
H	7.668643	9.152494	-4.460412
H	7.492810	7.430364	-4.844338
H	5.940922	9.271206	-6.166303
H	5.238927	7.740338	-5.639709
C	8.269791	14.676210	3.755191
O	7.146155	13.793781	3.771383
C	7.411725	12.686250	4.647407
C	8.775166	12.947023	5.271953
C	9.443488	13.847193	4.236947
H	8.385783	15.062587	2.741034
H	8.079802	15.522052	4.428372
H	6.609324	12.619583	5.385784
H	7.412919	11.768107	4.049629
H	8.665167	13.478821	6.220317
H	9.323582	12.023687	5.460126
H	10.246312	14.458274	4.650858
H	9.847592	13.252145	3.414829

khmds_dimer_2thf_bp86.log

SCF (RB-P86) = -3877.52366850
E(SCF)+ZPE(0 K)= -3876.625481
H(298 K)= -3876.558725
G(298 K)= -3876.736242
Lowest Frequency = 13.1212cm-1

K	5.448902	12.679258	1.969685
Si	8.524536	10.667280	0.962027
Si	6.038498	9.130306	1.888965
N	6.878285	10.449712	1.252410
C	8.842631	12.483795	0.484782
H	9.907338	12.675694	0.271690
H	8.553520	13.151213	1.312586
H	8.273469	12.806734	-0.402847
C	9.699091	10.298249	2.412158
H	9.744673	9.216744	2.618039
H	9.371834	10.794311	3.338706
H	10.726866	10.636114	2.194387
C	9.203312	9.601318	-0.469345
H	8.668846	9.762085	-1.419575
H	9.103904	8.531959	-0.217144
H	10.272686	9.794450	-0.662481
C	4.451393	9.704232	2.776938
H	3.773809	10.270304	2.116088
H	4.683131	10.334353	3.651984
H	3.874825	8.841072	3.149390
C	5.469155	7.877373	0.573395
H	4.855721	8.344258	-0.214379
H	4.865761	7.066916	1.017791
H	6.337887	7.409258	0.081705
C	6.978207	8.098294	3.183076
H	6.337622	7.305206	3.606064
H	7.329986	8.724275	4.019284
H	7.861130	7.607939	2.741410
C	2.656019	12.475234	4.701392
O	3.784332	13.126316	4.060992
C	3.599497	14.567010	4.102311
C	2.451753	14.816749	5.077734
C	1.593963	13.556534	4.885736
H	2.328518	11.641798	4.062228
H	2.990255	12.064589	5.670978
H	4.552683	15.022186	4.408957
H	3.343205	14.924154	3.087761
H	2.830258	14.871011	6.110474
H	1.912198	15.747550	4.855849
H	0.930172	13.348743	5.736073
H	0.979983	13.646287	3.976385
K	5.984076	11.151112	-1.341530
Si	2.805786	12.660990	-0.594263
Si	4.981219	14.806997	-0.871290
N	4.382206	13.262738	-0.545441
C	2.806510	10.768733	-0.820340
H	1.785558	10.365642	-0.712424
H	3.426975	10.252299	-0.069944
H	3.160378	10.461448	-1.818928
C	1.831366	12.969450	1.012722
H	1.735501	14.050712	1.204966
H	2.338771	12.522694	1.882776
H	0.813256	12.545667	0.972683
C	1.700576	13.316931	-1.998380
H	2.206905	13.228542	-2.974299
H	1.444043	14.378124	-1.852516
H	0.753969	12.752817	-2.061185
C	6.151844	15.432661	0.501370
H	7.013457	14.760643	0.647916
H	5.641867	15.559783	1.471546
H	6.567076	16.418683	0.232844
C	6.040582	14.841053	-2.449200
H	6.864499	14.112438	-2.381807
H	6.488811	15.831792	-2.635845
H	5.431493	14.576894	-3.329270
C	3.692917	16.193261	-1.076345
H	4.181795	17.177556	-1.178112
H	3.016780	16.247572	-0.206953
H	3.070553	16.039849	-1.972183
C	7.322676	12.174785	-4.559961
O	7.811676	11.737840	-3.270685
C	9.232323	11.985838	-3.285138
C	9.375005	13.347967	-3.961138
C	8.227037	13.347212	-5.000933
H	7.383747	11.334873	-5.274409
H	6.267606	12.454173	-4.433922
H	9.586495	11.955213	-2.247942
H	9.742802	11.191612	-3.863446
H	9.224797	14.146773	-3.220728
H	10.364400	13.484041	-4.418786
H	7.682752	14.300474	-4.993665
H	8.604335	13.179443	-6.019059
C	4.869668	9.367980	-4.302773

O	5.348477	9.069413	-2.963415
C	6.401841	8.085503	-3.067452
C	7.077766	8.383360	-4.398991
C	5.873146	8.738889	-5.289083
H	3.854348	8.956248	-4.423905
H	4.811860	10.463811	-4.398006
H	7.056777	8.202785	-2.193078
H	5.969349	7.067253	-3.049893
H	7.750261	9.246500	-4.283962
H	7.656985	7.532239	-4.782462
H	6.135035	9.426718	-6.104374
H	5.448947	7.828214	-5.736764
C	8.277190	14.689553	3.719238
O	7.120544	13.820112	3.771665
C	7.411373	12.682960	4.634140
C	8.797743	12.935053	5.230455
C	9.455386	13.835198	4.172935
H	8.367684	15.069636	2.690940
H	8.118405	15.547706	4.398517
H	6.617876	12.608268	5.392891
H	7.397273	11.768428	4.014098
H	8.714081	13.470268	6.188864
H	9.347656	12.000104	5.404628
H	10.283127	14.437403	4.571960
H	9.833911	13.231631	3.334062

khmds_dimer_2thf_m062x.log

SCF (RM062X) = -3876.37032030
E(SCF)+ZPE(0 K)= -3875.437410
H(298 K)= -3875.373636
G(298 K)= -3875.540670
Lowest Frequency = 14.6066cm-1

K	5.435401	12.660731	1.846488
Si	8.355128	10.646338	0.809773
Si	5.851893	9.108456	1.727552
N	6.720730	10.341399	1.000696
C	8.600893	12.491595	0.447757
H	9.651189	12.747796	0.277108
H	8.262408	13.083120	1.304957
H	8.036749	12.829788	-0.430295
C	9.466127	10.267220	2.294121
H	9.536189	9.189486	2.465548
H	9.096075	10.723214	3.216340
H	10.481840	10.638015	2.121720
C	9.159081	9.696232	-0.619040
H	8.680372	9.909756	-1.578582
H	9.075426	8.618753	-0.439114
H	10.223519	9.932231	-0.719268
C	4.394900	9.795682	2.730118
H	3.699571	10.389322	2.125937
H	4.750799	10.427067	3.552256
H	3.810695	8.986092	3.177938
C	5.116241	7.866797	0.502075
H	4.573823	8.349235	-0.317139
H	4.421145	7.181472	0.997464
H	5.912281	7.261312	0.057548
C	6.817950	8.046644	2.959697
H	6.171792	7.288750	3.413820
H	7.239524	8.652155	3.767418
H	7.644957	7.523856	2.470120
C	2.883958	12.362051	4.755912
O	3.980349	12.984580	4.072640
C	3.836992	14.410927	4.109946
C	2.767192	14.690401	5.154471
C	1.866022	13.466593	4.999697
H	2.501572	11.548997	4.137330
H	3.251473	11.942865	5.697578
H	4.809243	14.846798	4.341894
H	3.516231	14.762781	3.121531
H	3.210558	14.711742	6.151978
H	2.254324	15.634499	4.978926
H	1.244318	13.269864	5.871518
H	1.220987	13.581804	4.126231
K	5.775308	11.250231	-1.466065
Si	2.699664	12.765503	-0.485069
Si	4.924871	14.864518	-0.906488
N	4.257430	13.368568	-0.568440
C	2.690003	10.901398	-0.829561
H	1.696098	10.475142	-0.665123
H	3.376131	10.363689	-0.166140
H	2.967166	10.670161	-1.864082
C	1.905298	12.962989	1.224766
H	1.764229	14.025193	1.449921
H	2.522299	12.536828	2.022413
H	0.924307	12.480006	1.277695
C	1.450569	13.512693	-1.690408
H	1.825954	13.486267	-2.717766
H	1.230386	14.554597	-1.443463
H	0.505565	12.960970	-1.666127
C	6.028232	15.493676	0.504798
H	6.882400	14.830267	0.675202
H	5.482444	15.593449	1.449737
H	6.439414	16.479280	0.265885
C	6.061729	14.804432	-2.417699
H	6.832232	14.034592	-2.302711
H	6.573446	15.756739	-2.589882
H	5.485114	14.567937	-3.317570
C	3.695121	16.260008	-1.243074
H	4.213397	17.211844	-1.395356
H	3.001290	16.390784	-0.406862
H	3.101629	16.059290	-2.139163
C	7.315612	12.087992	-4.360990
O	7.848207	11.768221	-3.076530
C	9.239463	12.064963	-3.138774
C	9.306223	13.387953	-3.888598
C	8.138470	13.277795	-4.887543
H	7.416277	11.217600	-5.018354
H	6.254811	12.314963	-4.242173
H	9.624698	12.104722	-2.122102
H	9.758906	11.269124	-3.688095
H	9.137559	14.210642	-3.192837
H	10.268563	13.538891	-4.374641
H	7.548065	14.192112	-4.908454
H	8.491535	13.078492	-5.898208
C	5.287595	9.164950	-4.666861
O	5.433572	9.295034	-3.249309
C	6.411602	8.348258	-2.826193
C	7.450253	8.374132	-3.932138
C	6.565829	8.476836	-5.182125
H	4.397871	8.568792	-4.884611
H	5.144016	10.164666	-5.076696
H	6.785350	8.651924	-1.846382
H	5.954618	7.355992	-2.730549
H	8.071893	9.264576	-3.818418
H	8.090682	7.493606	-3.933164
H	7.039507	9.040969	-5.983945
H	6.330923	7.483298	-5.563256
C	8.305725	14.668283	3.511386
O	7.132079	13.852859	3.525917
C	7.300379	12.777010	4.463460
C	8.694551	12.942988	5.065591
C	9.420759	13.759091	3.996407
H	8.444691	15.045924	2.497945
H	8.168753	15.518411	4.188635
H	6.503638	12.825812	5.206992
H	7.209143	11.829734	3.918318
H	8.639213	13.510493	5.995921
H	9.167143	11.984413	5.274518
H	10.275444	14.312242	4.382410
H	9.755718	13.112188	3.182057

khmds_dimer_2thf_m06l.log

SCF (RM06L) = -3876.92415203
E(SCF)+ZPE(0 K)= -3875.991906
H(298 K)= -3875.927920

```
G(298 K)=             -3876.095408
Lowest Frequency = 18.9372cm-1

K        5.425656   12.677467    1.932926
Si        8.474343   10.674877    0.927438
Si        6.016356    9.127863    1.862736
N        6.848190   10.414775    1.196519
C        8.742930   12.495840    0.481020
H        9.791852   12.733373    0.284613
H        8.427017   13.138952    1.308079
H        8.178786   12.817682   -0.401016
C        9.653058   10.322236    2.361799
H        9.752690    9.250353    2.545073
H        9.325883   10.775336    3.299470
H       10.657210   10.701301    2.151725
C        9.185726    9.651372   -0.500614
H        8.671903    9.813869   -1.451726
H        9.100331    8.584395   -0.273633
H       10.246212    9.853794   -0.675831
C        4.488600    9.734357    2.805838
H        3.795120   10.307003    2.181942
H        4.768293   10.365361    3.655478
H        3.911292    8.902473    3.216534
C        5.387492    7.882218    0.585877
H        4.816571    8.343944   -0.223674
H        4.739182    7.126389    1.037706
H        6.221112    7.346918    0.123734
C        6.973483    8.081997    3.111730
H        6.344424    7.300290    3.546135
H        7.362256    8.679428    3.939797
H        7.826727    7.580978    2.647872
C        2.749441   12.479514    4.769335
O        3.839474   13.112064    4.084012
C        3.636135   14.532671    4.066389
C        2.505202   14.805267    5.033731
C        1.676727   13.536372    4.906969
H        2.436851   11.605200    4.194899
H        3.101519   12.130025    5.747437
H        4.577162   15.018686    4.332550
H        3.370825   14.843125    3.046107
H        2.891459   14.907849    6.049425
H        1.956860   15.713053    4.790609
H        1.018241   13.357311    5.754512
H        1.063421   13.571378    4.004477
K        5.944725   11.136990   -1.387245
Si        2.801235   12.685440   -0.580891
Si        4.983743   14.812328   -0.878562
N        4.357453   13.291974   -0.581633
C        2.809689   10.815703   -0.879929
H        1.808753   10.393118   -0.764601
H        3.448116   10.279515   -0.171036
H        3.138513   10.546808   -1.888048
C        1.899180   12.905056    1.069607
H        1.742516   13.963854    1.292331
H        2.451780   12.478955    1.911395
H        0.913982   12.430322    1.068167
C        1.618834   13.387535   -1.875583
H        2.059078   13.379778   -2.875931
H        1.334710   14.418666   -1.657231
H        0.693712   12.807075   -1.926060
C        6.065589   15.448603    0.545798
H        6.926420   14.801533    0.735904
H        5.522814   15.564551    1.488716
H        6.476102   16.432997    0.307173
C        6.137155   14.818717   -2.374470
H        6.901019   14.040343   -2.290819
H        6.662711   15.768962   -2.503101
H        5.583006   14.629352   -3.297307
C        3.736081   16.195561   -1.190136
H        4.234339   17.163161   -1.294006
H        3.016919   16.293050   -0.372651
H        3.163535   16.032742   -2.105397
C        7.333093   12.194135   -4.451322
O        7.870837   11.732342   -3.208225
C        9.274077   11.985149   -3.268232
C        9.378153   13.352595   -3.905150
C        8.226014   13.348407   -4.914519
H        7.333419   11.371784   -5.176851
H        6.295837   12.485408   -4.274082
H        9.670155   11.923128   -2.256503
H        9.764356   11.214149   -3.878940
H        9.223596   14.121371   -3.146621
H       10.348586   13.530532   -4.364275
H        7.691089   14.296217   -4.919068
H        8.583067   13.172508   -5.928057
C        4.927343    9.317774   -4.384538
O        5.383603    9.078834   -3.045227
C        6.422470    8.099977   -3.083989
C        7.097230    8.311097   -4.417200
C        5.903649    8.607876   -5.317063
H        3.903491    8.948040   -4.490780
H        4.902624   10.400532   -4.540215
H        7.066405    8.260120   -2.217961
H        5.994393    7.092120   -3.002580
H        7.768019    9.170807   -4.358227
H        7.678178    7.450704   -4.743335
H        6.158799    9.211166   -6.186154
H        5.469202    7.677207   -5.682817
C        8.262204   14.697901    3.705162
O        7.127327   13.827494    3.677901
C        7.360093   12.714981    4.559729
C        8.716862   12.947208    5.198658
C        9.410909   13.841207    4.181803
H        8.393230   15.115619    2.705500
H        8.071439   15.529586    4.395750
H        6.543682   12.659229    5.284069
H        7.342142   11.793816    3.960657
H        8.605166   13.473246    6.148017
H        9.246477   12.017059    5.396230
H       10.228367   14.423681    4.602175
H        9.807673   13.248383    3.354478

khmds_dimer_2thf_pbe0.log

SCF (RPBE1PBE) =      -3874.61061833
E(SCF)+ZPE(0 K)=      -3873.684359
H(298 K)=             -3873.618051
G(298 K)=             -3873.798111
Lowest Frequency = 8.7277cm-1

K        5.467564   12.697606    1.991170
Si        8.599807   10.743624    1.089338
Si        6.120010    9.125843    1.775965
N        6.954158   10.486699    1.264861
C        8.923153   12.553777    0.635111
H        9.990681   12.746485    0.483473
H        8.588401   13.214262    1.441475
H        8.406782   12.868167   -0.278098
C        9.662391   10.421334    2.621903
H        9.725247    9.351353    2.842454
H        9.249253   10.914781    3.506841
H       10.685368   10.788323    2.480189
C        9.401133    9.696232   -0.274194
H        8.911861    9.828229   -1.244680
H        9.332474    8.632698   -0.019216
H       10.462983    9.933009   -0.407222
C        4.514419    9.603899    2.662142
H        3.847574   10.207995    2.037899
H        4.725104   10.165232    3.579476
H        3.947749    8.713426    2.955173
C        5.615232    7.975472    0.357008
H        4.977632    8.470626   -0.383012
H        5.062071    7.103106    0.723467
H        6.504594    7.605748   -0.164893
C        7.028023    8.009335    3.005021
H        6.387552    7.182264    3.332107
H        7.333827    8.565303    3.897419
H        7.928788    7.571404    2.562748
```

C	2.709003	12.333465	4.712961
O	3.769018	13.060945	4.089826
C	3.559540	14.466075	4.259413
C	2.429320	14.605307	5.261900
C	1.616087	13.347067	4.986926
H	2.398032	11.526751	4.045406
H	3.080423	11.887310	5.644102
H	4.496680	14.919506	4.590974
H	3.282699	14.905167	3.292472
H	2.820906	14.588884	6.282950
H	1.865176	15.528526	5.123871
H	0.973742	13.052753	5.817939
H	0.994795	13.482085	4.097034
K	6.083510	11.226254	-1.338083
Si	2.849333	12.706065	-0.629150
Si	5.039853	14.823365	-0.904419
N	4.408464	13.313093	-0.547844
C	2.856466	10.834400	-0.926132
H	1.838321	10.433483	-0.874794
H	3.444940	10.291719	-0.179643
H	3.246142	10.576731	-1.917090
C	1.860845	12.944425	0.969172
H	1.756553	14.009408	1.202680
H	2.349600	12.465970	1.823691
H	0.852732	12.521597	0.891362
C	1.746406	13.398254	-2.002589
H	2.248339	13.351336	-2.975192
H	1.475243	14.441986	-1.819466
H	0.815651	12.824818	-2.081159
C	6.142556	15.487365	0.490255
H	6.994120	14.828933	0.690797
H	5.589386	15.631441	1.425686
H	6.560354	16.462452	0.216569
C	6.162544	14.792086	-2.427836
H	6.989266	14.085251	-2.304096
H	6.602220	15.774651	-2.633113
H	5.595131	14.490246	-3.314781
C	3.794399	16.210262	-1.228848
H	4.307657	17.171379	-1.347331
H	3.084409	16.315304	-0.401494
H	3.217624	16.030712	-2.140917
C	7.612558	11.963026	-4.485862
O	8.075953	11.720893	-3.162827
C	9.462164	12.029235	-3.177296
C	9.565390	13.301202	-4.001051
C	8.417684	13.155959	-5.013438
H	7.785514	11.070512	-5.100448
H	6.538045	12.146598	-4.441092
H	9.799639	12.135905	-2.147123
H	10.018394	11.205631	-3.647151
H	9.404421	14.171306	-3.361583
H	10.541857	13.405029	-4.475970
H	7.809712	14.060356	-5.050505
H	8.784821	12.957594	-6.021423
C	4.597436	9.444008	-4.389303
O	5.526381	9.439587	-3.303972
C	6.547802	8.466013	-3.540838
C	6.392783	8.041215	-4.988490
C	4.891442	8.193060	-5.194232
H	3.583838	9.468903	-3.982143
H	4.751567	10.348388	-4.991139
H	7.518565	8.918635	-3.322852
H	6.396497	7.620234	-2.860138
H	6.937567	8.722885	-5.647711
H	6.756579	7.028053	-5.163841
H	4.602281	8.294836	-6.240990
H	4.361439	7.335714	-4.770058
C	8.152182	14.827642	3.835562
O	7.081374	13.889409	3.834463
C	7.315548	12.897140	4.840677
C	8.671691	13.213267	5.461427
C	9.339085	14.073679	4.394503
H	8.290524	15.186600	2.813740
H	7.894965	15.685236	4.472344
H	6.502082	12.925970	5.571145
H	7.308843	11.913996	4.357545
H	8.544189	13.788096	6.382215
H	9.233774	12.309708	5.700760
H	10.108122	14.735884	4.794702
H	9.786955	13.448957	3.617105

khmds_dimer_2thf_wb97xd.log

SCF (RwB97XD) = -3876.74291949
E(SCF)+ZPE(0 K)= -3875.808478
H(298 K)= -3875.744860
G(298 K)= -3875.911474
Lowest Frequency = 19.1675cm-1

K	5.423755	12.684527	1.939599
Si	8.433294	10.662577	0.934851
Si	5.949041	9.101101	1.793570
N	6.802498	10.397669	1.172937
C	8.721209	12.480285	0.478466
H	9.778041	12.692961	0.289275
H	8.406653	13.126033	1.303639
H	8.165460	12.797344	-0.410633
C	9.562075	10.331486	2.416510
H	9.610938	9.262018	2.640820
H	9.205961	10.838556	3.317216
H	10.584043	10.673514	2.221863
C	9.182936	9.628406	-0.467918
H	8.674387	9.783803	-1.424150
H	9.103539	8.563059	-0.226382
H	10.243911	9.850881	-0.623427
C	4.397531	9.679005	2.718569
H	3.722865	10.265276	2.085435
H	4.659543	10.286957	3.590883
H	3.819851	8.825231	3.086271
C	5.333879	7.888380	0.476243
H	4.756970	8.382134	-0.311989
H	4.692332	7.115830	0.913393
H	6.176760	7.381045	-0.003192
C	6.886743	8.030583	3.041830
H	6.245939	7.240739	3.447719
H	7.252724	8.627563	3.882764
H	7.752369	7.546327	2.579426
C	2.801909	12.362499	4.778303
O	3.900932	13.037321	4.161337
C	3.703959	14.453222	4.216197
C	2.539406	14.679772	5.167230
C	1.716264	13.410677	4.959533
H	2.498756	11.531322	4.139620
H	3.130678	11.957774	5.741750
H	4.634715	14.919031	4.543291
H	3.463559	14.819757	3.210646
H	2.896230	14.737841	6.197846
H	1.992332	15.593390	4.938416
H	1.055945	13.181455	5.794946
H	1.115137	13.491408	4.051421
K	5.892894	11.181529	-1.419172
Si	2.730360	12.734666	-0.525391
Si	4.955946	14.813528	-0.938598
N	4.293074	13.322946	-0.578019
C	2.683984	10.870873	-0.870964
H	1.669705	10.479668	-0.743661
H	3.324700	10.304611	-0.187085
H	2.988340	10.630187	-1.895548
C	1.905800	12.934441	1.170110
H	1.861667	13.990888	1.453218
H	2.448302	12.404059	1.958440
H	0.881142	12.548206	1.170247
C	1.508078	13.491662	-1.755247
H	1.900075	13.458990	-2.776715
H	1.292023	14.536685	-1.517165
H	0.557544	12.947950	-1.746704
C	6.044485	15.489311	0.463963
H	6.898164	14.835206	0.668543

H	5.488859	15.628177	1.397926
H	6.458494	16.465554	0.192282
C	6.116862	14.737115	-2.430414
H	6.912420	14.001162	-2.279367
H	6.598506	15.701051	-2.624606
H	5.568386	14.451954	-3.333526
C	3.733535	16.207081	-1.317228
H	4.259245	17.153885	-1.479372
H	3.028901	16.357530	-0.493292
H	3.149708	15.995207	-2.217271
C	7.404775	12.124851	-4.516068
O	7.861783	11.703420	-3.234768
C	9.259936	11.956950	-3.204916
C	9.416975	13.311622	-3.878732
C	8.289548	13.312503	-4.926556
H	7.502472	11.296480	-5.226782
H	6.349303	12.383431	-4.427726
H	9.587603	11.935874	-2.167855
H	9.789960	11.171385	-3.759599
H	9.261471	14.106469	-3.148142
H	10.405396	13.439656	-4.318244
H	7.732241	14.247790	-4.905851
H	8.675924	13.171484	-5.935495
C	5.008013	9.336305	-4.422113
O	5.364031	9.092438	-3.058306
C	6.412979	8.130039	-3.014606
C	7.196358	8.355298	-4.292969
C	6.075532	8.658075	-5.288980
H	4.009044	8.936082	-4.613329
H	4.977127	10.418056	-4.572554
H	6.990304	8.297365	-2.105126
H	5.992117	7.117770	-2.975984
H	7.854044	9.218020	-4.170270
H	7.797151	7.491846	-4.575915
H	6.397690	9.294977	-6.111820
H	5.686974	7.730419	-5.711703
C	8.286943	14.747906	3.569126
O	7.158124	13.881848	3.641780
C	7.393802	12.858008	4.617050
C	8.800161	13.091175	5.164506
C	9.457517	13.917235	4.060266
H	8.386343	15.091976	2.538816
H	8.125153	15.621036	4.213038
H	6.623026	12.915641	5.388080
H	7.311969	11.886104	4.118876
H	8.757953	13.666659	6.091110
H	9.319334	12.155150	5.367081
H	10.285062	14.529437	4.416722
H	9.820577	13.269761	3.259101

khmds_monomer_1thf_b3lyp.log

SCF (RB3LYP) = -1706.14564591
E(SCF)+ZPE(0 K)= -1705.802603
H(298 K)= -1705.776363
G(298 K)= -1705.862224
Lowest Frequency = 12.9063cm-1

K	4.994608	2.917676	6.313599
N	4.798295	3.276129	3.730688
Si	4.131224	4.742552	3.285629
Si	5.333343	1.942561	2.878373
C	2.395401	4.615930	2.529465
C	3.939464	5.859180	4.811276
C	5.156330	5.737826	2.037513
C	4.045771	1.166162	1.721564
C	6.861992	2.255455	1.799908
C	5.840658	0.558702	4.083638
H	1.965342	5.600104	2.314694
H	1.711777	4.094004	3.206604
H	2.417349	4.052989	1.591587
H	3.512136	6.831892	4.548664
H	4.905322	6.050581	5.290266
H	3.274088	5.412154	5.556535
H	5.209546	5.219809	1.075097
H	6.182568	5.875092	2.391841
H	4.730319	6.729921	1.853520
H	3.784846	1.854832	0.912148
H	3.123070	0.931284	2.260991
H	4.409184	0.241100	1.261499
H	6.641050	2.980469	1.010879
H	7.221053	1.340305	1.317110
H	7.684564	2.663100	2.395901
H	6.189753	-0.328735	3.546706
H	5.001474	0.235378	4.710496
H	6.663237	0.867818	4.739484
C	2.900094	4.483917	8.892988
O	4.227378	4.430681	8.349693
C	4.814297	5.721236	8.594623
C	3.664405	6.739758	8.520240
C	2.386075	5.871202	8.525876
H	2.325150	3.665759	8.459706
H	2.946032	4.347675	9.979758
H	5.280122	5.717971	9.585080
H	5.588914	5.881209	7.845225
H	3.697185	7.416199	9.373470
H	3.724660	7.338429	7.613127
H	1.638134	6.227661	9.233088
H	1.934944	5.851447	7.534008

khmds_monomer_1thf_b3pw91.log

SCF (RB3PW91) = -1705.79027613
E(SCF)+ZPE(0 K)= -1705.446952
H(298 K)= -1705.420970
G(298 K)= -1705.503805
Lowest Frequency = 24.1324cm-1

K	2.272476	2.580548	5.726979
N	3.797051	3.256819	3.717179
Si	3.468809	4.815183	3.222082
Si	5.051694	2.174993	3.524100
C	2.801052	4.952354	1.457439
C	2.116806	5.574756	4.321283
C	4.930207	6.016290	3.298854
C	5.084473	1.305952	1.844040
C	6.787719	2.893142	3.758087
C	4.914155	0.782402	4.809966
H	2.541472	5.983087	1.191476
H	1.903934	4.337166	1.330951
H	3.543750	4.599557	0.734250
H	1.907451	6.610482	4.034517
H	2.406912	5.592096	5.377408
H	1.168368	5.030766	4.232053
H	5.683736	5.756452	2.548773
H	5.420565	5.995365	4.275946
H	4.615594	7.047493	3.103794
H	5.240090	2.030471	1.037723
H	4.133195	0.799792	1.648776
H	5.882497	0.558233	1.777902
H	7.012992	3.638633	2.989539
H	7.554863	2.113277	3.695662
H	6.890653	3.382286	4.731582
H	5.719092	0.050473	4.686108
H	3.972912	0.227276	4.715345
H	4.993161	1.162212	5.835620
C	3.115559	4.925563	8.480347
O	3.615406	3.940563	7.561969
C	4.864575	4.387148	7.000122
C	4.934120	5.870986	7.296883
C	4.224296	5.951642	8.644836
H	2.213402	5.379642	8.054402
H	2.845669	4.427369	9.414453
H	5.684428	3.843854	7.483767
H	4.857305	4.149096	5.933868
H	5.959203	6.241122	7.323674
H	4.383381	6.434091	6.539420
H	4.902445	5.654210	9.448756

H 3.835692 6.944574 8.872621

khmds_monomer_1thf_bp86.log

SCF (RB-P86) = -1706.16254620
E(SCF)+ZPE(0 K)= -1705.828926
H(298 K)= -1705.802555
G(298 K)= -1705.886075
Lowest Frequency = 20.9169cm-1

K	6.619421	4.235204	6.128427
N	5.333902	3.855098	3.896078
Si	4.609996	5.264111	3.330672
Si	5.179092	2.221454	3.525482
C	2.718490	5.181434	3.126212
C	4.950820	6.692068	4.553279
C	5.273793	5.871925	1.654226
C	3.393432	1.560504	3.500221
C	5.938691	1.714121	1.856489
C	6.085074	1.179564	4.845515
H	2.283387	6.173750	2.916087
H	2.227745	4.783940	4.028905
H	2.446352	4.519353	2.287534
H	4.464514	7.627358	4.228822
H	6.030654	6.913107	4.630113
H	4.570020	6.473789	5.565842
H	5.069137	5.131886	0.862462
H	6.366669	6.016095	1.692614
H	4.819196	6.828189	1.342313
H	2.805755	2.032968	2.696267
H	2.875765	1.766808	4.451355
H	3.363563	0.469644	3.334362
H	5.413125	2.209158	1.022644
H	5.886703	0.625382	1.682930
H	6.998551	2.014029	1.799060
H	6.018950	0.101298	4.622747
H	5.648437	1.318384	5.850681
H	7.161724	1.422264	4.895832
C	3.329630	3.831161	6.918291
O	4.521437	4.028425	7.731455
C	4.115823	4.848681	8.847251
C	3.173701	5.881648	8.235372
C	2.436462	5.071797	7.141595
H	3.672100	3.710595	5.878653
H	2.825537	2.905598	7.245543
H	3.596176	4.224268	9.598990
H	5.023516	5.270650	9.301233
H	2.490089	6.317980	8.976422
H	3.758531	6.697332	7.784494
H	1.434664	4.769775	7.476303
H	2.320534	5.656619	6.219780

khmds_monomer_1thf_m062x.log

SCF (RM062X) = -1705.70548179
E(SCF)+ZPE(0 K)= -1705.360268
H(298 K)= -1705.334117
G(298 K)= -1705.419717
Lowest Frequency = 12.9603cm-1

K	5.039984	2.864579	6.319820
N	4.825659	3.257657	3.752524
Si	4.142474	4.730052	3.361461
Si	5.361665	1.941639	2.877052
C	2.361836	4.622700	2.729577
C	4.065116	5.831309	4.903081
C	5.079977	5.720758	2.050911
C	4.093554	1.213264	1.677191
C	6.907043	2.264482	1.835245
C	5.835092	0.532157	4.057047
H	1.940216	5.610816	2.520307
H	1.715954	4.132146	3.464091
H	2.309943	4.037359	1.806591
H	3.596527	6.796482	4.688048
H	5.067822	6.035107	5.293890
H	3.478947	5.362484	5.701647
H	5.041510	5.212095	1.082519
H	6.133934	5.834009	2.321122
H	4.657821	6.721112	1.913472
H	3.863855	1.928256	0.880943
H	3.155322	0.977450	2.187815
H	4.457384	0.297294	1.201444
H	6.709029	3.022299	1.071325
H	7.253066	1.360714	1.324050
H	7.728334	2.633374	2.456806
H	6.178953	-0.349505	3.508309
H	4.984290	0.211629	4.668629
H	6.654284	0.821534	4.725451
C	2.844925	4.514199	8.829142
O	4.141999	4.425143	8.243006
C	4.765472	5.689364	8.478451
C	3.646364	6.738812	8.397191
C	2.349590	5.905088	8.452334
H	2.235674	3.700809	8.437275
H	2.931339	4.404733	9.916097
H	5.225273	5.678614	9.471403
H	5.546650	5.826451	7.730774
H	3.716041	7.437511	9.229123
H	3.706523	7.307309	7.470738
H	1.630165	6.289277	9.173212
H	1.871549	5.876854	7.473442

khmds_monomer_1thf_m06l.log

SCF (RM06L) = -1705.91275786
E(SCF)+ZPE(0 K)= -1705.566507
H(298 K)= -1705.540576
G(298 K)= -1705.624581
Lowest Frequency = 16.6282cm-1

K	5.310376	3.082091	6.292429
N	4.881314	3.274114	3.716964
Si	4.177492	4.726676	3.307421
Si	5.357252	1.926346	2.862763
C	2.407359	4.599575	2.655417
C	4.059322	5.826369	4.847548
C	5.108472	5.738223	2.010674
C	3.999971	1.110660	1.830619
C	6.786945	2.209031	1.658447
C	5.972550	0.590438	4.058879
H	1.954111	5.580708	2.489294
H	1.760928	4.058665	3.350998
H	2.366658	4.064403	1.703987
H	3.612653	6.799481	4.628788
H	5.039944	6.028196	5.290442
H	3.436250	5.369987	5.622648
H	5.087556	5.246251	1.035102
H	6.159742	5.872720	2.276196
H	4.678367	6.733910	1.872548
H	3.691442	1.755364	1.003872
H	3.105473	0.904912	2.423429
H	4.322843	0.163439	1.390266
H	6.503830	2.888653	0.851455
H	7.124933	1.280770	1.189730
H	7.652260	2.652432	2.157093
H	6.272983	-0.319610	3.534160
H	5.203987	0.290458	4.777547
H	6.850098	0.916138	4.626061
C	2.806344	4.509743	8.514977
O	4.213803	4.513990	8.254695
C	4.723411	5.709203	8.846141
C	3.680664	6.763969	8.542874
C	2.365965	5.973718	8.551804
H	2.327486	3.922634	7.729632
H	2.616943	4.009403	9.471111
H	4.848310	5.562476	9.926746
H	5.704537	5.905912	8.412852
H	3.692766	7.576680	9.266253

H	3.862608	7.195716	7.559271
H	1.778747	6.175213	9.445846
H	1.744464	6.228355	7.695581

khmds_monomer_1thf_pbe0.log

SCF (RPBE1PBE) = -1704.98641903
E(SCF)+ZPE(0 K)= -1704.642480
H(298 K)= -1704.616483
G(298 K)= -1704.699832
Lowest Frequency = 20.5610cm-1

K	2.387014	2.543127	5.761553
N	3.780480	3.256630	3.669888
Si	3.377288	4.805602	3.201523
Si	5.057138	2.207657	3.444567
C	2.684672	4.942090	1.447191
C	2.010299	5.489817	4.330671
C	4.781731	6.072441	3.285165
C	5.056348	1.311724	1.779153
C	6.780005	2.975430	3.605079
C	5.005160	0.831073	4.753956
H	2.379694	5.965432	1.200550
H	1.812515	4.291867	1.318803
H	3.434096	4.632188	0.710608
H	1.734031	6.510448	4.044674
H	2.327080	5.532245	5.379744
H	1.092683	4.891103	4.268904
H	5.556607	5.846024	2.545391
H	5.261001	6.079980	4.269215
H	4.420624	7.087102	3.081872
H	5.154799	2.026599	0.954812
H	4.118035	0.767028	1.628222
H	5.879007	0.592564	1.695398
H	6.957978	3.715167	2.817819
H	7.566173	2.215791	3.526499
H	6.904328	3.483136	4.567359
H	5.825010	0.119065	4.609731
H	4.076839	0.248149	4.702673
H	5.113143	1.227024	5.771648
C	3.219212	4.866750	8.565261
O	3.703135	3.936334	7.588862
C	4.947227	4.404589	7.045940
C	5.028694	5.867743	7.424191
C	4.336642	5.870945	8.781198
H	2.318029	5.353738	8.173855
H	2.950991	4.317259	9.471163
H	5.769209	3.832156	7.492721
H	4.936042	4.225305	5.967604
H	6.056406	6.231002	7.459046
H	4.470578	6.476929	6.707578
H	5.022182	5.517509	9.556286
H	3.959034	6.850660	9.076181

khmds_monomer_1thf_wb97xd.log

SCF (RwB97XD) = -1705.85491975
E(SCF)+ZPE(0 K)= -1705.509063
H(298 K)= -1705.483200
G(298 K)= -1705.567231
Lowest Frequency = 16.7968cm-1

K	5.014904	2.845187	6.379439
N	4.821462	3.235080	3.784382
Si	4.143861	4.700041	3.359404
Si	5.349611	1.940185	2.874171
C	2.387129	4.577443	2.662629
C	4.001849	5.818882	4.883677
C	5.120770	5.689763	2.074661
C	4.075307	1.248241	1.656608
C	6.893945	2.284432	1.834981
C	5.821453	0.492341	4.007806
H	1.961628	5.562851	2.444822
H	1.719343	4.074655	3.369676
H	2.374334	3.998342	1.733985
H	3.556579	6.786884	4.633342
H	4.984012	6.017638	5.325685
H	3.369360	5.367714	5.655687
H	5.129314	5.172162	1.110026
H	6.162051	5.821511	2.384881
H	4.691173	6.683629	1.910234
H	3.844989	1.982888	0.878249
H	3.136592	1.002034	2.162650
H	4.434765	0.342457	1.157008
H	6.696615	3.060373	1.088822
H	7.237863	1.391637	1.301999
H	7.718722	2.637280	2.462384
H	6.150807	-0.376282	3.429036
H	4.974158	0.162642	4.619956
H	6.649404	0.754006	4.677273
C	2.890242	4.532814	8.895020
O	4.196939	4.462646	8.334727
C	4.782904	5.750019	8.521306
C	3.636157	6.765478	8.418342
C	2.363486	5.899652	8.480487
H	2.308056	3.695849	8.509472
H	2.954058	4.445124	9.986637
H	5.258723	5.788286	9.507138
H	5.550626	5.883114	7.758700
H	3.681655	7.479292	9.239907
H	3.685229	7.322579	7.484031
H	1.626224	6.281794	9.185204
H	1.894857	5.836752	7.498114

khmds_monomer_2thf_b3lyp.log

SCF (RB3LYP) = -1938.73269755
E(SCF)+ZPE(0 K)= -1938.271329
H(298 K)= -1938.238714
G(298 K)= -1938.338257
Lowest Frequency = 20.1625cm-1

K	2.834276	3.190698	6.212922
N	4.214435	3.384436	3.941874
Si	4.941888	4.876009	3.782137
Si	4.390068	1.872255	3.262868
C	4.336309	5.893622	2.299264
C	4.570633	5.959351	5.300687
C	6.833633	4.839217	3.617238
C	3.690838	1.705961	1.507389
C	6.174868	1.232739	3.158095
C	3.461943	0.570708	4.298851
H	4.807266	6.881541	2.254416
H	3.252707	6.042663	2.343715
H	4.555664	5.379078	1.358440
H	5.047847	6.940907	5.219440
H	4.928465	5.504551	6.228780
H	3.495784	6.135692	5.403478
H	7.131765	4.396947	2.661943
H	7.296915	4.245403	4.409362
H	7.263470	5.845736	3.659978
H	4.223038	2.366062	0.815053
H	2.633930	1.989322	1.479360
H	3.773388	0.684537	1.120898
H	6.780918	1.857695	2.496055
H	6.213858	0.208850	2.771112
H	6.655999	1.233576	4.140541
H	3.549846	-0.425789	3.854902
H	2.389661	0.788771	4.365656
H	3.863739	0.495959	5.315865
C	2.415492	5.945977	8.491676
O	1.645042	5.207057	7.518253
C	0.765754	6.105435	6.804332
C	0.805187	7.423408	7.565974
C	2.220287	7.415912	8.150570
H	2.035807	5.710225	9.490603
H	3.455304	5.624836	8.426864
H	1.138019	6.225174	5.781906

H	-0.226887	5.655179	6.766140
H	0.611608	8.277668	6.918368
H	0.062928	7.421785	8.367007
H	2.945869	7.728402	7.397517
H	2.327894	8.058865	9.023436
C	5.269935	3.244706	9.061974
O	4.925237	2.703211	7.782479
C	6.143669	2.643239	7.011237
C	7.051780	3.767345	7.543938
C	6.280653	4.339364	8.751348
H	4.354554	3.598805	9.535178
H	5.712380	2.460593	9.688958
H	6.602904	1.659857	7.149652
H	5.866428	2.763069	5.964995
H	8.018595	3.365932	7.846286
H	7.228784	4.529803	6.787543
H	6.926612	4.560978	9.600373
H	5.759030	5.255460	8.470933

khmds_monomer_2thf_b3pw91.log

SCF (RB3PW91) = -1938.28045487
E(SCF)+ZPE(0 K)= -1937.819653
H(298 K)= -1937.786515
G(298 K)= -1937.890885
Lowest Frequency = 10.4730cm-1

K	4.800215	4.597591	5.740306
N	4.803546	3.772749	3.245722
Si	4.395222	4.993797	2.186413
Si	5.075281	2.136216	3.078668
C	2.675786	4.821562	1.410265
C	4.365084	6.663932	3.090919
C	5.586176	5.217233	0.731077
C	3.757611	1.200762	2.090728
C	6.724947	1.702417	2.255161
C	5.130256	1.302553	4.783168
H	2.417386	5.682633	0.783609
H	1.907994	4.730325	2.185922
H	2.615885	3.925958	0.783865
H	4.142817	7.485572	2.401871
H	5.332027	6.890206	3.555363
H	3.593898	6.691638	3.869639
H	5.572754	4.334681	0.082923
H	6.614671	5.349776	1.082532
H	5.328107	6.084196	0.112662
H	3.752462	1.526767	1.045463
H	2.757634	1.384691	2.496890
H	3.928894	0.118467	2.096873
H	6.761837	2.089548	1.231671
H	6.895879	0.621053	2.208324
H	7.561005	2.149905	2.803224
H	5.306810	0.225142	4.697183
H	4.184843	1.429948	5.322301
H	5.936284	1.705747	5.406791
C	2.180412	5.228404	8.089893
O	2.853206	5.924795	7.026995
C	2.970511	7.313349	7.359371
C	2.821805	7.379713	8.865700
C	1.791995	6.282288	9.117089
H	1.319407	4.698666	7.675380
H	2.872198	4.492361	8.510952
H	3.934507	7.674756	6.994229
H	2.176330	7.877197	6.855681
H	3.771014	7.134838	9.349534
H	2.499460	8.362116	9.211752
H	1.815128	5.891876	10.134769
H	0.785640	6.657669	8.917184
C	4.982713	2.337965	8.638731
O	5.497513	3.574706	8.122961
C	5.922794	4.415337	9.207046
C	5.853575	3.557990	10.459471
C	4.732845	2.582613	10.114244
H	4.081335	2.077168	8.080337
H	5.726314	1.548308	8.484341
H	6.928923	4.782147	8.991118
H	5.245104	5.273846	9.273330
H	6.793182	3.019307	10.604965
H	5.654216	4.149100	11.353612
H	4.763004	1.662863	10.698778
H	3.757573	3.054342	10.260858

khmds_monomer_2thf_m062x.log

SCF (RM062X) = -1938.15853902
E(SCF)+ZPE(0 K)= -1937.693617
H(298 K)= -1937.661158
G(298 K)= -1937.762912
Lowest Frequency = 5.8099cm-1

K	3.252282	3.815121	5.666150
N	4.091778	3.412540	3.173380
Si	4.184065	4.895407	2.422198
Si	4.828179	1.920668	3.104971
C	3.052154	5.089192	0.920030
C	3.650811	6.270226	3.622397
C	5.905854	5.398070	1.818343
C	4.078652	0.730852	1.840298
C	6.683931	1.948371	2.732164
C	4.679499	1.032906	4.778410
H	3.116460	6.089807	0.481647
H	2.007797	4.907799	1.190512
H	3.318479	4.366424	0.142663
H	3.690930	7.255740	3.148908
H	4.305817	6.313286	4.500466
H	2.619664	6.129186	3.966418
H	6.248302	4.739136	1.015217
H	6.642561	5.345661	2.625216
H	5.909756	6.420701	1.428002
H	4.173653	1.135841	0.828018
H	3.012516	0.577093	2.031361
H	4.566956	-0.248376	1.854953
H	6.883037	2.348477	1.733939
H	7.115368	0.943314	2.776131
H	7.221329	2.573172	3.451651
H	5.130952	0.036927	4.742835
H	3.634023	0.893876	5.077396
H	5.195357	1.588966	5.568919
C	2.385194	5.598897	8.653297
O	2.562112	5.839118	7.256201
C	2.435342	7.249099	7.086901
C	3.229945	7.834828	8.245427
C	2.996182	6.812595	9.375847
H	1.315402	5.515145	8.868360
H	2.867340	4.650793	8.893757
H	2.815891	7.511521	6.101808
H	1.377279	7.529175	7.151341
H	4.286355	7.878789	7.979361
H	2.900389	8.838889	8.506258
H	3.929570	6.555463	9.874439
H	2.308661	7.193750	10.129138
C	5.090348	2.038924	8.404473
O	4.796219	3.263406	7.737599
C	5.541270	4.265825	8.427119
C	5.495188	3.871711	9.911105
C	5.047175	2.393964	9.887122
H	4.354737	1.296499	8.098715
H	6.088191	1.695411	8.108475
H	6.570147	4.266478	8.053494
H	5.087078	5.232723	8.207852
H	6.472262	3.997569	10.374185
H	4.782639	4.484979	10.461060
H	5.691903	1.746742	10.478795
H	4.029034	2.296112	10.263136

khmds_monomer_2thf_m06l.log

SCF (RM06L) = -1938.43879923

E(SCF)+ZPE(0 K)= -1937.974309
H(298 K)= -1937.942012
G(298 K)= -1938.039527
Lowest Frequency = 26.2202cm-1

K	2.263424	3.537900	5.673322
N	3.928946	3.424377	3.611082
Si	4.322166	4.929105	3.024093
Si	4.612278	1.911533	3.516845
C	3.796941	5.236632	1.234583
C	3.441145	6.264356	4.042755
C	6.161492	5.374678	3.067966
C	4.804776	1.237115	1.760610
C	6.331218	1.750710	4.293827
C	3.556883	0.649802	4.458544
H	4.036970	6.246735	0.891640
H	2.721332	5.097210	1.102503
H	4.291401	4.539112	0.553853
H	3.673271	7.275829	3.700122
H	3.720581	6.220253	5.099861
H	2.352961	6.162164	3.983929
H	6.739903	4.724831	2.405829
H	6.592614	5.274492	4.067292
H	6.343412	6.402025	2.740263
H	5.512738	1.841907	1.187116
H	3.859190	1.254427	1.213429
H	5.174492	0.208382	1.739232
H	7.055310	2.431967	3.840976
H	6.735639	0.739703	4.190171
H	6.302941	1.974250	5.362885
H	3.937679	-0.367835	4.341510
H	2.518228	0.636226	4.116712
H	3.552505	0.849939	5.534612
C	2.359180	5.336162	8.848163
O	1.946768	5.514468	7.489175
C	1.713778	6.915059	7.341977
C	2.872266	7.563255	8.067913
C	3.129186	6.601588	9.235260
H	1.473506	5.201748	9.479705
H	2.950279	4.421296	8.891989
H	1.661454	7.139340	6.277559
H	0.752318	7.180635	7.801169
H	3.738977	7.610764	7.407607
H	2.649907	8.578132	8.391254
H	4.192541	6.405087	9.365187
H	2.760965	7.003298	10.177721
C	4.958223	2.299275	8.274272
O	4.371930	3.108019	7.249384
C	5.231805	4.230394	6.971875
C	6.485287	4.018645	7.794944
C	5.968527	3.188643	8.960859
H	4.159728	1.942281	8.927291
H	5.439545	1.424321	7.819941
H	5.406633	4.267383	5.893039
H	4.703744	5.146933	7.262734
H	7.225083	3.453201	7.225473
H	6.945488	4.957260	8.097717
H	6.745474	2.626384	9.474790
H	5.472986	3.827387	9.695299

khmds_monomer_2thf_pbe0.log

SCF (RPBE1PBE) = -1937.27947478
E(SCF)+ZPE(0 K)= -1936.817937
H(298 K)= -1936.784492
G(298 K)= -1936.890631
Lowest Frequency = 17.4543cm-1

K	4.270248	4.187407	5.907107
N	4.569590	3.642644	3.344485
Si	3.958556	4.823289	2.335467
Si	5.328120	2.178035	3.091931
C	2.392318	4.320241	1.396707
C	3.470785	6.358467	3.337413
C	5.159645	5.442927	1.008489
C	4.367031	0.952189	2.015510
C	7.043222	2.302637	2.298457
C	5.608043	1.294051	4.748275
H	1.992478	5.140944	0.790052
H	1.605943	4.004446	2.091117
H	2.591820	3.477978	0.725539
H	3.075738	7.148238	2.688897
H	4.332133	6.779107	3.868743
H	2.691558	6.136640	4.076212
H	5.394074	4.647716	0.292446
H	6.104470	5.771151	1.454925
H	4.746635	6.284837	0.441050
H	4.269010	1.328100	0.991245
H	3.355502	0.794631	2.404955
H	4.861654	-0.024456	1.961519
H	6.978583	2.723797	1.289554
H	7.532498	1.325024	2.218428
H	7.698149	2.957146	2.883998
H	6.095833	0.323581	4.605601
H	4.662581	1.101546	5.268723
H	6.255944	1.876166	5.414895
C	2.332926	6.411857	8.100849
O	3.409825	6.381683	7.174336
C	3.781401	7.741635	6.978354
C	3.627961	8.417654	8.343175
C	2.743670	7.442204	9.141216
H	1.412046	6.716228	7.584639
H	2.195316	5.405219	8.498503
H	4.800143	7.760693	6.588842
H	3.115003	8.196444	6.234887
H	4.595888	8.552806	8.827189
H	3.167646	9.400925	8.239495
H	3.318552	6.964547	9.936804
H	1.879047	7.927792	9.595135
C	4.601757	1.582332	8.570979
O	4.456607	2.984897	8.309276
C	4.700387	3.728244	9.507695
C	5.380927	2.764486	10.457816
C	4.718030	1.447234	10.077479
H	3.738404	1.056948	8.155449
H	5.504562	1.220523	8.066574
H	5.308800	4.601266	9.259639
H	3.743976	4.075369	9.917254
H	6.454826	2.724483	10.254801
H	5.235844	3.039334	11.503276
H	5.294098	0.568597	10.369765
H	3.726214	1.376903	10.532688

khmds_monomer_2thf_wb97xd.log

SCF (RwB97XD) = -1938.34420125
E(SCF)+ZPE(0 K)= -1937.878856
H(298 K)= -1937.846599
G(298 K)= -1937.947237
Lowest Frequency = 7.1816cm-1

K	3.342251	3.905604	5.782352
N	4.208914	3.508914	3.290900
Si	4.153795	4.896873	2.373141
Si	4.903897	2.006583	3.109216
C	2.949191	4.826702	0.914133
C	3.572804	6.361285	3.433662
C	5.809185	5.435918	1.626265
C	4.176022	0.959282	1.709501
C	6.772536	2.023920	2.797517
C	4.678727	0.981256	4.691403
H	2.916992	5.769374	0.357555
H	1.932844	4.605303	1.255484
H	3.236794	4.036555	0.213039
H	3.527565	7.288338	2.853519
H	4.255594	6.541348	4.271789
H	2.568251	6.192498	3.838060
H	6.154113	4.713708	0.879631

H	6.585293	5.509015	2.394525
H	5.734797	6.409879	1.130938
H	4.355178	1.433329	0.738953
H	3.093066	0.847836	1.823351
H	4.613993	-0.043843	1.672556
H	7.009162	2.502059	1.842063
H	7.191209	1.012130	2.772806
H	7.294719	2.582031	3.581251
H	5.102746	-0.021965	4.582579
H	3.620372	0.853784	4.946380
H	5.183961	1.449219	5.543746
C	2.338113	5.610585	8.781427
O	2.296204	5.820357	7.367150
C	2.427138	7.218559	7.131979
C	3.431393	7.677580	8.171813
C	3.035413	6.837886	9.392467
H	1.318777	5.498678	9.162271
H	2.876649	4.678194	8.959645
H	2.749695	7.360254	6.100742
H	1.457862	7.713807	7.270971
H	4.442137	7.430449	7.840793
H	3.382614	8.749316	8.359631
H	3.897065	6.558391	9.997190
H	2.343541	7.388320	10.029978
C	5.047471	2.053045	8.502170
O	4.955855	3.298982	7.823135
C	5.675845	4.239929	8.613861
C	5.474418	3.813380	10.076241
C	4.857141	2.405259	9.970861
H	4.284751	1.387095	8.099327
H	6.033828	1.607082	8.324153
H	6.734943	4.209854	8.336499
H	5.287034	5.233282	8.388210
H	6.425655	3.800252	10.606666
H	4.808994	4.496454	10.602766
H	5.335816	1.682809	10.630394
H	3.793692	2.432050	10.211203

khmds_monomer_3thf_b3lyp.log

SCF (RB3LYP) = -2171.31461539
E(SCF)+ZPE(0 K)= -2170.736010
H(298 K)= -2170.696322
G(298 K)= -2170.815496
Lowest Frequency = 7.3772cm-1

K	5.271184	4.113480	5.957557
N	5.141247	3.668436	3.310378
Si	3.815297	4.392979	2.606400
Si	6.229106	2.501665	2.825775
C	2.236399	3.337831	2.692640
C	3.389945	6.019319	3.491380
C	4.009229	4.849613	0.773358
C	5.452889	0.917050	2.118685
C	7.459559	3.077373	1.499245
C	7.303394	1.936445	4.289902
H	1.370537	3.846273	2.254826
H	1.984107	3.087479	3.727137
H	2.373053	2.393767	2.155839
H	2.487367	6.483721	3.081123
H	4.206907	6.740615	3.387964
H	3.216986	5.868084	4.560008
H	4.152110	3.956339	0.157416
H	4.878827	5.496120	0.620634
H	3.130358	5.377418	0.387359
H	4.894358	1.133911	1.202597
H	4.754823	0.462237	2.827197
H	6.210047	0.165506	1.869958
H	6.938079	3.355216	0.578169
H	8.189789	2.301176	1.245952
H	8.013769	3.958395	1.838398
H	8.035072	1.183826	3.979175
H	6.705989	1.489181	5.090593
H	7.870272	2.772067	4.715876
C	1.643556	4.759326	6.677526
O	2.995192	5.083735	7.063081
C	3.042514	6.418242	7.611191
C	1.592868	6.849177	7.795246
C	0.884017	6.077563	6.679092
H	1.670386	4.275477	5.701624
H	1.226510	4.058060	7.407068
H	3.606527	6.388808	8.543274
H	3.565118	7.069979	6.903987
H	1.222523	6.529259	8.771724
H	1.471259	7.929003	7.717957
H	-0.181598	5.939871	6.859199
H	1.011365	6.588199	5.722713
C	5.961349	4.662633	9.389009
O	6.427473	5.198996	8.131553
C	6.985326	6.512890	8.333835
C	7.194929	6.645235	9.835406
C	6.039688	5.807896	10.391005
H	4.949015	4.279808	9.250322
H	6.613077	3.831981	9.675107
H	7.907208	6.584802	7.755808
H	6.278316	7.262327	7.962870
H	8.153952	6.209776	10.124147
H	7.173229	7.682862	10.166712
H	6.212806	5.454341	11.406776
H	5.113812	6.386772	10.381148
C	3.754564	1.034754	5.726310
O	4.372188	1.729734	6.831532
C	3.513659	1.667485	7.990623
C	2.340680	0.768076	7.608040
C	2.282173	0.930352	6.086400
H	3.948604	1.605394	4.818440
H	4.211833	0.044851	5.626301
H	4.091163	1.281503	8.832624
H	3.184577	2.682603	8.227355
H	2.556576	-0.270404	7.867340
H	1.418598	1.060311	8.109727
H	1.789897	0.098922	5.583076
H	1.762508	1.851729	5.817655

khmds_monomer_3thf_b3pw91.log

SCF (RB3PW91) = -2170.77723132
E(SCF)+ZPE(0 K)= -2170.198284
H(298 K)= -2170.158568
G(298 K)= -2170.277324
Lowest Frequency = 12.3212cm-1

K	5.268379	4.092132	6.005711
N	5.129712	3.652225	3.377480
Si	3.814300	4.377381	2.654205
Si	6.211807	2.493839	2.860161
C	2.235358	3.329565	2.718760
C	3.381821	5.999065	3.534555
C	4.035270	4.833488	0.828429
C	5.431481	0.937493	2.108182
C	7.443605	3.101432	1.554907
C	7.282211	1.888219	4.305052
H	1.381051	3.835364	2.254871
H	1.960128	3.095730	3.751815
H	2.381248	2.377106	2.198677
H	2.490380	6.471062	3.107883
H	4.205901	6.715900	3.453845
H	3.185019	5.839557	4.598676
H	4.184427	3.939146	0.214627
H	4.909121	5.478231	0.689504
H	3.162598	5.363838	0.430735
H	4.877284	1.185479	1.196759
H	4.728411	0.465922	2.801293
H	6.186389	0.189798	1.839757
H	6.926041	3.393290	0.635501
H	8.180578	2.334094	1.292259
H	7.989398	3.979736	1.915820
H	8.002744	1.132256	3.975104

H	6.682465	1.433254	5.100189
H	7.862073	2.708930	4.743493
C	1.656956	4.750609	6.730866
O	2.997355	5.101995	7.102291
C	3.028285	6.449259	7.595005
C	1.579294	6.854345	7.797772
C	0.876087	6.051270	6.708227
H	1.684199	4.246633	5.763208
H	1.252458	4.056149	7.475772
H	3.617161	6.468796	8.513546
H	3.517734	7.089389	6.851867
H	1.231265	6.541658	8.785865
H	1.433792	7.930929	7.705568
H	-0.186860	5.899552	6.897626
H	0.989126	6.543764	5.739431
C	6.000173	4.735772	9.436544
O	6.462231	5.206865	8.161623
C	7.031799	6.515050	8.302114
C	7.241718	6.716908	9.791390
C	6.077656	5.924733	10.378455
H	4.986805	4.344333	9.320012
H	6.652127	3.919426	9.766481
H	7.956204	6.552238	7.722040
H	6.334542	7.257224	7.895561
H	8.194636	6.281597	10.103240
H	7.234900	7.770268	10.072724
H	6.237219	5.622052	11.413653
H	5.156514	6.511098	10.329399
C	3.748854	1.058240	5.665715
O	4.338400	1.691348	6.812105
C	3.468012	1.553434	7.944295
C	2.312151	0.671498	7.493375
C	2.275047	0.929756	5.990145
H	3.960084	1.677587	4.790741
H	4.210164	0.074122	5.520764
H	4.034336	1.123435	8.774416
H	3.122848	2.549635	8.239674
H	2.535607	-0.380202	7.689414
H	1.380084	0.922589	8.000785
H	1.791207	0.133056	5.424610
H	1.758769	1.867910	5.773771

khmds_monomer_3thf_bp86.log

SCF (RB-P86) = -2171.30579101
E(SCF)+ZPE(0 K)= -2170.744312
H(298 K)= -2170.703964
G(298 K)= -2170.823526
Lowest Frequency = 9.4094cm-1

K	5.316108	4.148590	5.959333
N	5.126102	3.654579	3.342086
Si	3.772945	4.350941	2.628407
Si	6.215985	2.490884	2.808622
C	2.198604	3.282839	2.761497
C	3.345632	5.994260	3.494530
C	3.941246	4.772369	0.777260
C	5.431773	0.898163	2.110015
C	7.406697	3.091726	1.447473
C	7.340094	1.933207	4.246150
H	1.321329	3.765685	2.296733
H	1.944913	3.080285	3.815392
H	2.342686	2.306487	2.269115
H	2.428949	6.452104	3.085583
H	4.162623	6.725787	3.375036
H	3.182994	5.851724	4.575477
H	4.081905	3.860834	0.172535
H	4.811842	5.425186	0.598739
H	3.048247	5.291653	0.387670
H	4.845105	1.117441	1.201945
H	4.748553	0.430114	2.837299
H	6.192601	0.146596	1.835784
H	6.855264	3.356460	0.529948
H	8.155013	2.326336	1.177275
H	7.952012	3.993442	1.773798
H	8.069477	1.174226	3.915813
H	6.765771	1.486255	5.075134
H	7.923281	2.778537	4.652214
C	1.688509	4.779732	6.723598
O	3.051532	5.145379	7.072517
C	3.077498	6.524005	7.535401
C	1.619259	6.928720	7.738392
C	0.903665	6.086947	6.671085
H	1.710658	4.243479	5.763520
H	1.293394	4.101640	7.500600
H	3.676868	6.565578	8.456419
H	3.564940	7.148051	6.765275
H	1.277895	6.642755	8.745596
H	1.466450	8.009188	7.613408
H	-0.165092	5.939786	6.877372
H	1.007735	6.554639	5.680142
C	5.804214	4.703397	9.423088
O	6.421169	5.184987	8.195498
C	7.072283	6.457123	8.449627
C	7.200558	6.559642	9.966332
C	5.926644	5.840836	10.436734
H	4.761226	4.426952	9.203184
H	6.347574	3.801497	9.753665
H	8.037032	6.455642	7.921293
H	6.446777	7.272974	8.043657
H	8.096841	6.021412	10.312056
H	7.263966	7.600339	10.312062
H	5.991808	5.471561	11.469089
H	5.059955	6.516466	10.364870
C	3.846179	1.086161	5.680245
O	4.459426	1.742941	6.826747
C	3.562824	1.656994	7.967997
C	2.408250	0.748323	7.541535
C	2.366358	0.964958	6.021048
H	4.053435	1.701974	4.790071
H	4.311661	0.093161	5.546878
H	4.129153	1.266184	8.827478
H	3.211577	2.675045	8.211148
H	2.641640	-0.303253	7.769551
H	1.467217	1.009118	8.045073
H	1.877347	0.144998	5.477963
H	1.841964	1.901808	5.777268

khmds_monomer_3thf_m062x.log

SCF (RM062X) = -2170.61450905
E(SCF)+ZPE(0 K)= -2170.030796
H(298 K)= -2169.992129
G(298 K)= -2170.104930
Lowest Frequency = 20.2137cm-1

K	5.424575	4.239319	5.847323
N	5.187066	3.748295	3.221544
Si	3.811337	4.434015	2.580288
Si	6.164775	2.458699	2.823302
C	2.270972	3.329299	2.668993
C	3.372302	6.020594	3.519183
C	3.932930	4.935888	0.758950
C	5.255046	0.914156	2.205564
C	7.444152	2.835474	1.481080
C	7.179685	1.904219	4.328979
H	1.381004	3.838876	2.285586
H	2.054585	3.011043	3.693476
H	2.414734	2.422996	2.072005
H	2.424624	6.450252	3.180343
H	4.149094	6.777582	3.372213
H	3.284245	5.843402	4.595964
H	4.077053	4.056155	0.123591
H	4.783338	5.603819	0.593127
H	3.030360	5.448833	0.411917
H	4.754388	1.120862	1.254100
H	4.490611	0.579527	2.912625
H	5.942967	0.079288	2.037765

H	6.948729	3.133505	0.551799
H	8.079396	1.972256	1.259492
H	8.094265	3.661646	1.783994
H	7.830837	1.060819	4.080207
H	6.547275	1.583764	5.163998
H	7.830393	2.711779	4.683334
C	1.879518	4.708434	6.619704
O	3.189142	5.093253	7.059467
C	3.168429	6.441193	7.547651
C	1.698509	6.790628	7.723845
C	1.056107	5.988419	6.593380
H	1.966369	4.230109	5.641537
H	1.464961	3.986034	7.330138
H	3.741498	6.481913	8.474609
H	3.645145	7.096119	6.810127
H	1.339842	6.435667	8.692096
H	1.516574	7.861613	7.654204
H	-0.005353	5.798323	6.742122
H	1.190898	6.504740	5.641137
C	5.818927	4.699227	9.131295
O	6.286026	5.501672	8.034994
C	6.806613	6.742495	8.522647
C	7.058955	6.520008	10.004532
C	5.906226	5.585293	10.368219
H	4.802869	4.365853	8.909426
H	6.468577	3.823804	9.223300
H	7.704719	6.985084	7.954943
H	6.064371	7.532221	8.364551
H	8.016791	6.017554	10.152217
H	7.058986	7.449716	10.570846
H	6.082019	5.008998	11.274789
H	4.984292	6.156874	10.493098
C	3.803121	1.282427	5.732332
O	4.387990	1.990685	6.835176
C	3.599382	1.799423	8.016806
C	2.553418	0.749292	7.660768
C	2.378063	0.972258	6.158798
H	3.876022	1.912374	4.842153
H	4.369662	0.361663	5.557643
H	4.256673	1.490356	8.831076
H	3.136343	2.754069	8.281104
H	2.942778	-0.253103	7.847574
H	1.633451	0.875942	8.229477
H	1.968707	0.109065	5.636563
H	1.730779	1.831796	5.973254

khmds_monomer_3thf_m06l.log

SCF (RM06L) = -2170.95998483
E(SCF)+ZPE(0 K)= -2170.377698
H(298 K)= -2170.338536
G(298 K)= -2170.453916
Lowest Frequency = 16.9371cm-1

K	5.352769	4.315108	5.851805
N	5.147004	3.712011	3.239409
Si	3.798431	4.390266	2.542651
Si	6.183621	2.484707	2.807668
C	2.237453	3.321720	2.659966
C	3.365530	6.018413	3.408047
C	3.948450	4.814281	0.705559
C	5.358282	0.895909	2.189426
C	7.419115	2.936318	1.448795
C	7.253093	1.971840	4.286468
H	1.365211	3.807774	2.213425
H	1.975526	3.085646	3.694672
H	2.369466	2.366906	2.144153
H	2.431738	6.451975	3.040319
H	4.146175	6.767757	3.253536
H	3.249436	5.897457	4.488636
H	4.071372	3.913920	0.097726
H	4.812711	5.452712	0.507348
H	3.066124	5.336345	0.325159
H	4.813383	1.072678	1.258084

H	4.638897	0.491614	2.905364
H	6.086325	0.106114	1.983873
H	6.911997	3.184093	0.513191
H	8.115785	2.122324	1.230085
H	8.017808	3.807887	1.724364
H	7.957353	1.178998	4.022232
H	6.663609	1.593467	5.126140
H	7.856259	2.806226	4.657193
C	1.813001	4.761205	6.613047
O	3.099764	5.184613	7.085895
C	3.002865	6.516127	7.614600
C	1.524124	6.830282	7.690001
C	0.968839	6.013033	6.533937
H	1.947665	4.257932	5.651699
H	1.390128	4.035550	7.319215
H	3.506519	6.542926	8.582808
H	3.530334	7.204259	6.942648
H	1.109283	6.478889	8.636464
H	1.319153	7.895792	7.610582
H	-0.096068	5.805747	6.616133
H	1.140552	6.526674	5.586318
C	5.849724	4.689115	9.184275
O	6.284290	5.503374	8.082113
C	6.924188	6.683563	8.581780
C	7.245826	6.391400	10.029108
C	6.067441	5.518499	10.432331
H	4.806572	4.408166	9.017892
H	6.449177	3.771165	9.200342
H	7.799138	6.887650	7.963013
H	6.238474	7.534968	8.488719
H	8.179308	5.830954	10.104822
H	7.345968	7.294022	10.628298
H	6.256410	4.906262	11.311558
H	5.191859	6.136967	10.638176
C	3.798870	1.264739	5.798566
O	4.330286	2.037954	6.884293
C	3.539542	1.815598	8.060626
C	2.488701	0.785643	7.686620
C	2.368750	0.966613	6.180228
H	3.916829	1.850360	4.881136
H	4.380516	0.340542	5.688110
H	4.193832	1.486341	8.872001
H	3.093535	2.770194	8.358682
H	2.841336	-0.220819	7.916876
H	1.552011	0.937766	8.219801
H	1.970359	0.093070	5.667828
H	1.726115	1.816252	5.942504

khmds_monomer_3thf_pbe0.log

SCF (RPBE1PBE) = -2169.57771987
E(SCF)+ZPE(0 K)= -2168.997556
H(298 K)= -2168.957862
G(298 K)= -2169.076560
Lowest Frequency = 12.2389cm-1

K	5.180284	3.910112	6.091800
N	5.160356	3.689541	3.427888
Si	3.890174	4.477235	2.687931
Si	6.247349	2.529693	2.923817
C	2.286047	3.465666	2.654495
C	3.461861	6.071557	3.617768
C	4.184785	5.002199	0.891857
C	5.481100	1.005814	2.095160
C	7.548717	3.151711	1.696533
C	7.237229	1.858485	4.397485
H	1.460261	4.008548	2.180127
H	1.967329	3.199572	3.668393
H	2.429456	2.529921	2.102909
H	2.589614	6.575384	3.186363
H	4.300052	6.776361	3.585492
H	3.235516	5.875793	4.670971
H	4.334847	4.131151	0.244696
H	5.076967	5.632035	0.808564

H	3.337634	5.569148	0.488685
H	4.969031	1.283356	1.167137
H	4.742826	0.525674	2.745978
H	6.237870	0.254904	1.839966
H	7.078034	3.490025	0.767166
H	8.276856	2.374692	1.436628
H	8.100804	4.002026	2.111624
H	7.969688	1.110988	4.073319
H	6.594028	1.370991	5.139283
H	7.799590	2.654145	4.901242
C	1.607736	4.680798	6.758990
O	2.935265	5.015661	7.174383
C	2.954671	6.340598	7.715092
C	1.502840	6.743172	7.890423
C	0.831766	5.982722	6.754289
H	1.657791	4.205256	5.776640
H	1.179307	3.966006	7.472125
H	3.516474	6.327445	8.651899
H	3.467321	7.006619	7.010343
H	1.124930	6.395577	8.856074
H	1.362890	7.823293	7.833766
H	-0.237475	5.828807	6.904374
H	0.977599	6.507232	5.806073
C	6.114786	4.904504	9.432087
O	6.460591	5.214887	8.078334
C	7.050210	6.516706	8.012992
C	7.388575	6.889130	9.442747
C	6.271436	6.196127	10.211922
H	5.095850	4.507477	9.453770
H	6.796407	4.130058	9.802841
H	7.921831	6.469955	7.356129
H	6.326111	7.219318	7.581993
H	8.359916	6.473212	9.724353
H	7.417187	7.968628	9.595473
H	6.509016	6.019695	11.261617
H	5.352722	6.787216	10.160193
C	3.560027	0.862481	5.679993
O	4.134913	1.527009	6.808309
C	3.291257	1.355822	7.952017
C	2.139134	0.465255	7.510083
C	2.090094	0.715075	6.007599
H	3.760250	1.465404	4.791242
H	4.036433	-0.117859	5.551202
H	3.877508	0.917190	8.764449
H	2.940766	2.342736	8.273581
H	2.370959	-0.584444	7.708546
H	1.208300	0.711491	8.022731
H	1.616587	-0.093563	5.449516
H	1.557856	1.644430	5.786874

khmds_monomer_3thf_wb97xd.log

SCF (RwB97XD) = -2170.83761083
E(SCF)+ZPE(0 K)= -2170.253685
H(298 K)= -2170.214800
G(298 K)= -2170.328975
Lowest Frequency = 19.5568cm-1

K	5.452902	4.207797	5.915519
N	5.192088	3.715084	3.263410
Si	3.825666	4.404637	2.608116
Si	6.176495	2.467806	2.762484
C	2.272358	3.323619	2.748574
C	3.402121	6.024777	3.495572
C	3.941394	4.849991	0.769892
C	5.271590	0.932303	2.112810
C	7.386830	2.934696	1.382506
C	7.261910	1.854508	4.192433
H	1.382111	3.827017	2.356317
H	2.066900	3.050338	3.788404
H	2.399026	2.391244	2.188731
H	2.472666	6.464427	3.119514
H	4.197224	6.763865	3.353181
H	3.278407	5.875980	4.572260

H	4.076462	3.952687	0.157198
H	4.793702	5.509648	0.578846
H	3.039095	5.358548	0.413533
H	4.694654	1.173482	1.213984
H	4.573012	0.531262	2.853084
H	5.970813	0.131936	1.847841
H	6.846203	3.248103	0.483527
H	8.040324	2.100948	1.104360
H	8.023991	3.770976	1.687963
H	7.917545	1.037549	3.874448
H	6.662293	1.478602	5.028311
H	7.909988	2.653017	4.571268
C	1.865059	4.728074	6.674238
O	3.177489	5.147381	7.059926
C	3.150100	6.505726	7.507146
C	1.682070	6.854218	7.692819
C	1.019647	5.990262	6.622342
H	1.931726	4.216052	5.712858
H	1.483482	4.022882	7.420365
H	3.732388	6.580186	8.426594
H	3.614818	7.142365	6.745736
H	1.343661	6.554997	8.687326
H	1.491744	7.919216	7.567843
H	-0.032952	5.789777	6.816987
H	1.108501	6.464663	5.642935
C	5.790898	4.690798	9.361623
O	6.398997	5.296680	8.213432
C	6.901410	6.590060	8.550133
C	7.020833	6.598268	10.063700
C	5.826351	5.737421	10.468766
H	4.775842	4.383241	9.100439
H	6.369022	3.801452	9.629525
H	7.851977	6.733271	8.034948
H	6.197572	7.357282	8.206550
H	7.956046	6.125968	10.371861
H	6.987044	7.605158	10.477687
H	5.932392	5.287607	11.454897
H	4.911499	6.333821	10.460190
C	3.814179	1.178216	5.705181
O	4.442142	1.861132	6.796052
C	3.650653	1.731125	7.980145
C	2.513136	0.777628	7.634128
C	2.370333	0.975834	6.126471
H	3.932152	1.788972	4.807932
H	4.317111	0.218003	5.544156
H	4.283876	1.361972	8.789709
H	3.275555	2.721078	8.257214
H	2.799768	-0.253004	7.852880
H	1.603598	1.005538	8.188840
H	1.906951	0.129214	5.621664
H	1.783298	1.870470	5.909018

KHMDS nucleation

khmds_trimer_wb97xd.log

SCF (RwB97XD) = -4420.17228041
E(SCF)+ZPE(0 K)= -4419.482477
H(298 K)= -4419.426554
G(298 K)= -4419.570237
Lowest Frequency = 22.6975cm-1

K	4.193542	3.839532	5.897127
Si	5.457382	3.984594	9.205579
Si	4.257071	6.620205	8.179928
N	5.133029	5.192126	8.087966
C	4.021252	3.475040	10.318916
C	6.880195	4.389614	10.392938
C	5.989667	2.399557	8.306679
C	5.340590	8.163360	7.961564
C	3.303529	6.904313	9.783543
C	2.976243	6.761378	6.790251
H	4.266478	2.566932	10.879170
H	3.778044	4.255178	11.045043

```
H         3.117524    3.271953    9.735842
H         6.946998    3.634149   11.182454
H         7.864729    4.411195    9.913801
H         6.720111    5.356899   10.880005
H         5.141199    1.860489    7.868690
H         6.712964    2.599409    7.508269
H         6.465990    1.699659    8.999915
H         6.102944    8.250499    8.744275
H         5.835807    8.199586    6.984192
H         4.727805    9.067776    8.024308
H         3.968325    6.896110   10.652579
H         2.792818    7.872375    9.767274
H         2.545096    6.132218    9.939696
H         2.430875    7.707257    6.863742
H         3.438855    6.749321    5.797256
H         2.225090    5.964472    6.835721
N         5.165242    3.698017    3.303424
Si         4.541519    5.119448    2.666127
Si         4.946987    2.094695    2.860289
C         4.765521    5.351078    0.806426
C         2.690240    5.365548    2.982858
C         5.412289    6.599126    3.469743
C         3.979507    1.102901    4.158076
C         4.046724    1.807113    1.226860
C         6.595466    1.172353    2.705255
H         4.487132    6.364356    0.499141
H         4.147265    4.651647    0.237193
H         5.806945    5.189588    0.509412
H         2.328882    6.283023    2.507152
H         2.432561    5.445667    4.044183
H         2.116000    4.531388    2.567421
H         6.428081    6.738977    3.085670
H         5.483187    6.491927    4.557760
H         4.879305    7.534448    3.273970
H         2.981367    1.519543    4.334853
H         4.508221    1.037702    5.116522
H         3.832718    0.071841    3.822247
H         3.056303    2.272300    1.227174
H         3.910015    0.738361    1.034137
H         4.608663    2.225883    0.387213
H         6.426575    0.118504    2.463484
H         7.171427    1.187233    3.637755
H         7.221810    1.579485    1.903416
K         7.711471    6.042904    7.502299
N         9.400903    5.332454    5.448075
Si         9.754850    6.800103    4.719514
Si        10.318008    4.062937    6.045274
C        11.444520    7.543308    5.109321
C         8.491492    8.126730    5.207436
C         9.675047    6.746179    2.821468
C         9.956190    3.709309    7.872427
C        12.190943    4.247184    5.919032
C         9.930634    2.408503    5.194980
H        11.524827    8.553859    4.695891
H        11.610469    7.612665    6.188496
H        12.257585    6.947399    4.686767
H         8.586671    9.002950    4.559136
H         7.462755    7.766897    5.097827
H         8.629448    8.490286    6.232338
H        10.282897    5.929919    2.416143
H         8.658547    6.651326    2.423216
H        10.070722    7.676444    2.402063
H        10.170196    4.570968    8.514775
H         8.917789    3.400003    8.037055
H        10.579852    2.886659    8.235332
H        12.553513    5.103681    6.493413
H        12.692872    3.353362    6.302967
H        12.509390    4.384983    4.881507
H        10.530551    1.610703    5.643245
H         8.884574    2.103681    5.316240
H        10.174100    2.411513    4.126310
K         7.866390    4.107993    3.491407
```

Fluorinated gases

ch2f-cf3_01a_ch2f-cf3_b3pw91.log

```
SCF (RB3PW91) =       -476.834270523
E(SCF)+ZPE(0 K)=      -476.789186
H(298 K)=             -476.782768
G(298 K)=             -476.818678
Lowest Frequency = 111.3607cm-1

C        0.334663   -0.221757   -0.003773
H        0.710206    0.297165    0.879517
H       -0.756357   -0.220977   -0.018724
C        0.846135    0.478582   -1.243473
F        2.186159    0.508841   -1.290234
F        0.406064    1.746602   -1.257955
F        0.423842   -0.114595   -2.369725
F        0.792771   -1.522098   -0.001531
```

ch2f-cf3_01b_ch2f-cf3_pbe0.log

```
SCF (RPBE1PBE) =      -476.543529722
E(SCF)+ZPE(0 K)=      -476.498116
H(298 K)=             -476.491723
G(298 K)=             -476.527586
Lowest Frequency = 111.4131cm-1

C        0.335510   -0.221838   -0.005112
H        0.710770    0.298008    0.878324
H       -0.755970   -0.220270   -0.020062
C        0.845692    0.478127   -1.242495
F        2.181800    0.507235   -1.288624
F        0.407334    1.742161   -1.257370
F        0.424679   -0.114308   -2.364908
F        0.793668   -1.517353   -0.005650
```

ch2f-cf3_01c_ch2f-cf3_wb97xd.log

```
SCF (RwB97XD) =       -476.873891293
E(SCF)+ZPE(0 K)=      -476.828394
H(298 K)=             -476.822020
G(298 K)=             -476.857837
Lowest Frequency = 113.9284cm-1

C        0.334508   -0.222638   -0.002982
H        0.710212    0.296099    0.879396
H       -0.755737   -0.221598   -0.018368
C        0.846692    0.479085   -1.244677
F        2.183326    0.509731   -1.291100
F        0.407596    1.743872   -1.259116
F        0.425261   -0.112434   -2.368089
F        0.791626   -1.520354   -0.000962
```

ch2f-cf3_02a_encountcmplx_dimer_intact_1_b3pw91.log

```
SCF (RB3PW91) =       -3423.47891757
E(SCF)+ZPE(0 K)=      -3422.977734
H(298 K)=             -3422.932177
G(298 K)=             -3423.056914
Lowest Frequency = 15.6057cm-1

K        4.232702    3.665981    5.377626
Si        7.106806    4.040670    7.822718
Si        5.580196    6.524620    7.128859
N        6.130159    4.964951    6.810843
C        8.812366    4.798320    8.145954
C        7.459707    2.354007    7.044068
C        6.386366    3.678826    9.532365
C        6.692547    7.862948    6.372974
C        5.415247    6.999203    8.948611
C        3.860887    6.801161    6.387911
H        9.426566    4.157537    8.788243
H        9.372211    4.958996    7.217652
H        8.719725    5.769432    8.642478
```

H 8.042950 1.729112 7.728528
H 6.547037 1.802084 6.802056
H 8.040793 2.433925 6.120330
H 6.428872 4.564033 10.172354
H 5.337838 3.375922 9.470758
H 6.940186 2.882050 10.040334
H 7.722706 7.753252 6.728031
H 6.708949 7.850457 5.277766
H 6.353510 8.864288 6.659222
H 6.378802 6.969685 9.465621
H 5.019783 8.015904 9.047266
H 4.733327 6.325007 9.474595
H 3.533131 7.834667 6.539139
H 3.835091 6.625711 5.308565
H 3.109678 6.159691 6.863164
N 5.446585 4.042166 2.913385
K 7.433738 5.120897 4.379068
Si 4.549924 5.254956 2.164472
Si 5.750675 2.509680 2.286030
C 4.500050 5.211393 0.277114
C 2.729857 5.261666 2.700485
C 5.227663 6.966888 2.600766
C 4.187060 1.496042 1.951843
C 6.747469 2.482611 0.680743
C 6.769237 1.480083 3.501074
H 3.912189 6.049165 -0.113191
H 4.046693 4.288803 -0.097084
H 5.505331 5.285198 -0.147721
H 2.157124 6.005921 2.136900
H 2.585287 5.506655 3.758213
H 2.269411 4.285782 2.513704
H 6.242014 7.107907 2.209656
H 5.244611 7.151129 3.678715
H 4.608026 7.755723 2.162085
H 3.547147 1.994962 1.217314
H 3.592158 1.357156 2.861434
H 4.422407 0.499799 1.561875
H 6.182149 2.900227 -0.156107
H 7.034506 1.460765 0.409819
H 7.666525 3.069295 0.784108
H 6.944670 0.478560 3.094355
H 6.277310 1.346792 4.468393
H 7.754021 1.919843 3.691695
C 3.404300 1.028611 7.732420
C 4.378414 0.478006 8.748194
H 4.502780 -0.591911 8.572980
H 5.331583 0.998121 8.651617
F 3.213484 2.354747 7.875632
F 3.886902 0.837499 6.487344
F 2.205412 0.447838 7.799882
F 3.865439 0.683603 10.009256

ch2f-cf3_02b_encountcmplx_dimer_intact_1_pbe0.log

SCF (RPBE1PBE) = -3421.97142210
E(SCF)+ZPE(0 K)= -3421.469657
H(298 K)= -3421.423980
G(298 K)= -3421.548884
Lowest Frequency = 19.7408cm-1

K 4.247323 3.696830 5.402491
Si 7.155188 4.077342 7.826742
Si 5.578525 6.546202 7.160792
N 6.159869 4.999911 6.829732
C 8.857779 4.845212 8.139175
C 7.514955 2.397701 7.035818
C 6.455136 3.694871 9.540860
C 6.643480 7.912508 6.388278
C 5.430442 7.017306 8.982787
C 3.839449 6.785221 6.450184
H 9.482937 4.203585 8.770565
H 9.407073 5.016731 7.205900
H 8.764474 5.812507 8.644376

H 8.125963 1.782628 7.705650
H 6.605257 1.829558 6.816393
H 8.072330 2.487165 6.097325
H 6.496052 4.573858 10.190523
H 5.407988 3.382356 9.486995
H 7.021676 2.897216 10.034323
H 7.679207 7.838512 6.738125
H 6.654885 7.891699 5.292450
H 6.274546 8.905277 6.669195
H 6.401880 7.007179 9.486955
H 5.017192 8.026884 9.085636
H 4.768519 6.331326 9.520106
H 3.487176 7.807267 6.626007
H 3.796712 6.628913 5.367182
H 3.112824 6.117080 6.928456
N 5.434074 4.022583 2.918137
K 7.412190 5.165344 4.368402
Si 4.550208 5.236390 2.152574
Si 5.714115 2.479825 2.299636
C 4.525687 5.191350 0.264738
C 2.724988 5.256028 2.668365
C 5.223636 6.949792 2.592915
C 4.140682 1.524207 1.858617
C 6.805609 2.429919 0.757683
C 6.613721 1.399112 3.562858
H 3.936873 6.025377 -0.133054
H 4.083789 4.265572 -0.116808
H 5.536122 5.272826 -0.147907
H 2.163968 6.008714 2.103266
H 2.573727 5.496509 3.727137
H 2.256645 4.285037 2.473117
H 6.249050 7.089000 2.229249
H 5.206698 7.147694 3.669528
H 4.617644 7.733953 2.126427
H 3.567103 2.039503 1.081085
H 3.482892 1.409777 2.728034
H 4.369024 0.518769 1.487389
H 6.319210 2.908624 -0.097026
H 7.042834 1.399553 0.470057
H 7.753013 2.951913 0.932310
H 6.797999 0.404199 3.142685
H 6.044578 1.249605 4.486219
H 7.588959 1.812793 3.841111
C 3.422874 1.049756 7.687748
C 4.373379 0.418949 8.675768
H 4.443210 -0.649546 8.462794
H 5.351667 0.894188 8.587216
F 3.289815 2.372643 7.882798
F 3.892106 0.887911 6.437521
F 2.204146 0.520277 7.738911
F 3.879216 0.608025 9.941978

ch2f-cf3_02c_encountcmplx_dimer_intact_1_wb97xd.log

SCF (RwB97XD) = -3423.65520934
E(SCF)+ZPE(0 K)= -3423.150660
H(298 K)= -3423.105597
G(298 K)= -3423.228815
Lowest Frequency = 24.7996cm-1

K 4.225840 3.651719 5.346719
Si 7.089135 4.027515 7.828069
Si 5.557298 6.520838 7.148841
N 6.117292 4.967575 6.831343
C 8.805741 4.762730 8.145036
C 7.418707 2.341595 7.035486
C 6.383377 3.663168 9.543609
C 6.664534 7.869375 6.403372
C 5.383719 6.987070 8.970659
C 3.841526 6.797514 6.398156
H 9.415233 4.110554 8.779314
H 9.363327 4.923979 7.215814
H 8.726889 5.730930 8.649614

H	8.002927	1.706109	7.708770
H	6.498973	1.798765	6.798376
H	7.990577	2.423247	6.106041
H	6.427853	4.548693	10.183141
H	5.335721	3.355529	9.491552
H	6.944145	2.868787	10.046893
H	7.694337	7.764938	6.760787
H	6.684840	7.859671	5.308085
H	6.318688	8.867103	6.692120
H	6.347197	6.962759	9.488373
H	4.979987	7.999382	9.074073
H	4.708371	6.304462	9.494176
H	3.503881	7.824658	6.566926
H	3.828590	6.643159	5.314767
H	3.090900	6.140119	6.851535
N	5.462721	4.053333	2.858289
K	7.456932	5.125995	4.374971
Si	4.573827	5.275761	2.122615
Si	5.752268	2.511261	2.256923
C	4.529133	5.248871	0.234465
C	2.751639	5.286421	2.652663
C	5.250808	6.981512	2.585449
C	4.180971	1.492917	1.972258
C	6.706705	2.441489	0.627425
C	6.789327	1.503999	3.476363
H	3.957610	6.098541	-0.152637
H	4.060169	4.336361	-0.145590
H	5.536187	5.305116	-0.188686
H	2.182642	6.032519	2.088767
H	2.606360	5.530410	3.710730
H	2.288071	4.312359	2.464464
H	6.269539	7.127059	2.208525
H	5.256548	7.147795	3.667125
H	4.636884	7.777489	2.152903
H	3.534754	1.972597	1.230313
H	3.593224	1.378368	2.889659
H	4.410443	0.486604	1.606950
H	6.121730	2.849799	-0.200716
H	6.970755	1.410876	0.368773
H	7.636242	3.016355	0.689635
H	6.967810	0.497706	3.084167
H	6.305217	1.382219	4.449592
H	7.772802	1.952295	3.653598
C	3.448593	1.015996	7.779217
C	4.421771	0.515392	8.825375
H	4.554329	-0.559413	8.697238
H	5.371749	1.037822	8.711730
F	3.237037	2.340416	7.873292
F	3.944110	0.787700	6.549605
F	2.260318	0.421844	7.856476
F	3.901458	0.771292	10.070925

ch2f-cf3_03a_encountcmplx_dimer_intact_2_b3pw91.log

SCF (RB3PW91) = -3423.47903720
E(SCF)+ZPE(0 K)= -3422.977601
H(298 K)= -3422.932152
G(298 K)= -3423.056339
Lowest Frequency = 18.9855cm-1

K	4.238660	3.656607	5.370325
Si	7.108162	4.036669	7.823987
Si	5.578749	6.520355	7.131090
N	6.127495	4.959965	6.815173
C	8.814237	4.794594	8.143051
C	7.457246	2.349856	7.045362
C	6.393745	3.678014	9.537887
C	6.691220	7.857699	6.373886
C	5.415080	6.996856	8.950742
C	3.859512	6.796454	6.390203
H	9.429448	4.154104	8.784653
H	9.372583	4.953890	7.213639
H	8.723013	5.766289	8.638691

H	8.037111	1.721715	7.729668
H	6.544452	1.799941	6.799111
H	8.041297	2.430069	6.123484
H	6.438523	4.564517	10.175901
H	5.345017	3.375182	9.479062
H	6.949677	2.881838	10.044431
H	7.721769	7.747542	6.727646
H	6.706111	7.845124	5.278667
H	6.353128	8.859296	6.660335
H	6.379180	6.967652	9.466766
H	5.019948	8.013730	9.048849
H	4.733493	6.323431	9.478228
H	3.530542	7.829467	6.542180
H	3.834881	6.621919	5.310697
H	3.108700	6.153355	6.863866
N	5.451349	4.043562	2.909744
K	7.434932	5.118094	4.381168
Si	4.549484	5.254178	2.163871
Si	5.757791	2.511484	2.281615
C	4.494417	5.211694	0.276681
C	2.730587	5.255416	2.704570
C	5.223425	6.967545	2.600315
C	4.195609	1.494819	1.950572
C	6.750473	2.487680	0.673744
C	6.781559	1.484696	3.494315
H	3.903276	6.048108	-0.111586
H	4.042379	4.288081	-0.096591
H	5.498297	5.288218	-0.150966
H	2.154190	5.998048	2.142581
H	2.588066	5.499974	3.762683
H	2.272469	4.278233	2.518813
H	6.236741	7.111175	2.207520
H	5.242225	7.150275	3.678510
H	4.601013	7.755458	2.163892
H	3.553139	1.992457	1.217386
H	3.602887	1.353913	2.861240
H	4.432495	0.499160	1.560109
H	6.181931	2.904391	-0.161390
H	7.038953	1.466558	0.401661
H	7.668529	3.076305	0.774919
H	6.960561	0.484544	3.085827
H	6.291485	1.346764	4.461941
H	7.765007	1.927808	3.684422
C	3.394527	1.032965	7.723230
C	4.361153	0.503867	8.757574
H	5.325655	0.996597	8.635656
F	3.227189	2.359495	7.890927
F	3.846849	0.854835	6.465073
F	2.194318	0.455857	7.795798
H	3.953148	0.692020	9.751879
F	4.510333	-0.852566	8.568269

ch2f-cf3_03b_encountcmplx_dimer_intact_2_pbe0.log

SCF (RPBE1PBE) = -3421.97145268
E(SCF)+ZPE(0 K)= -3421.469284
H(298 K)= -3421.423774
G(298 K)= -3421.548145
Lowest Frequency = 18.1068cm-1

K	4.250636	3.686484	5.394302
Si	7.153996	4.071357	7.828475
Si	5.577093	6.540622	7.162353
N	6.155508	4.992805	6.833580
C	8.856839	4.839348	8.138410
C	7.511027	2.391654	7.037922
C	6.457268	3.693099	9.545586
C	6.644907	7.904634	6.389910
C	5.429265	7.013712	8.984158
C	3.839012	6.781714	6.450548
H	9.482318	4.197736	8.769501
H	9.405500	5.009651	7.204569
H	8.764657	5.807090	8.642944

H	8.117546	1.772957	7.708443
H	6.601248	1.826049	6.812740
H	8.072824	2.481619	6.102073
H	6.502053	4.573065	10.193632
H	5.408970	3.384660	9.492146
H	7.022242	2.894113	10.038534
H	7.680626	7.828359	6.739273
H	6.655691	7.884348	5.294076
H	6.278255	8.898113	6.671285
H	6.400723	7.002650	9.488276
H	5.017582	8.024019	9.086033
H	4.766258	6.329435	9.522380
H	3.487199	7.803933	6.626310
H	3.797814	6.625761	5.367478
H	3.111341	6.113505	6.927103
N	5.438256	4.023069	2.914118
K	7.413426	5.160840	4.370645
Si	4.550434	5.236082	2.152151
Si	5.720214	2.481117	2.293435
C	4.520625	5.193442	0.264375
C	2.726200	5.251874	2.672311
C	5.221855	6.950088	2.593250
C	4.147532	1.522224	1.857253
C	6.805525	2.435951	0.746960
C	6.627273	1.402040	3.552319
H	3.929392	6.027066	-0.130672
H	4.078963	4.267449	-0.116920
H	5.529714	5.276863	-0.151140
H	2.162494	6.003816	2.108889
H	2.576687	5.491563	3.731513
H	2.259233	4.280135	2.477429
H	6.246343	7.090986	2.227675
H	5.207112	7.146311	3.670212
H	4.613760	7.734105	2.129248
H	3.568921	2.037623	1.083506
H	3.494039	1.402967	2.729236
H	4.377453	0.518393	1.482718
H	6.313995	2.913325	-0.105557
H	7.044806	1.406431	0.458048
H	7.751972	2.960895	0.917869
H	6.815345	0.409137	3.129122
H	6.060925	1.246186	4.476329
H	7.601350	1.819103	3.829750
C	3.415948	1.051817	7.682948
C	4.366553	0.453187	8.691063
H	5.347353	0.918730	8.583024
F	3.289161	2.371659	7.899390
F	3.859424	0.908069	6.420700
F	2.203359	0.509007	7.737434
H	3.968128	0.618317	9.693898
F	4.467250	-0.894519	8.448979

ch2f-cf3_03c_encountcmplx_dimer_intact_2_wb97xd.log

SCF (RwB97XD) = -3423.65526371
E(SCF)+ZPE(0 K)= -3423.150193
H(298 K)= -3423.105324
G(298 K)= -3423.228073
Lowest Frequency = 20.7590cm-1

K	4.221701	3.650305	5.317713
Si	7.071710	4.015532	7.839008
Si	5.551633	6.508665	7.145018
N	6.102564	4.950369	6.834340
C	8.783913	4.756207	8.166729
C	7.412310	2.328264	7.055526
C	6.358539	3.657159	9.553803
C	6.679087	7.849006	6.415903
C	5.358587	6.975472	8.965098
C	3.847787	6.798834	6.373528
H	9.390679	4.105197	8.804820
H	9.347510	4.918607	7.241388
H	8.699715	5.724101	8.671004

H	7.974783	1.689139	7.743659
H	6.497837	1.788910	6.791129
H	8.011827	2.409474	6.143583
H	6.400475	4.544914	10.190328
H	5.311283	3.349492	9.497443
H	6.917921	2.864292	10.060850
H	7.702865	7.736566	6.787781
H	6.715040	7.839525	5.321072
H	6.336676	8.849195	6.700182
H	6.315664	6.946655	9.494278
H	4.958591	7.989749	9.063582
H	4.673620	6.296373	9.480722
H	3.514497	7.827542	6.541276
H	3.848811	6.648264	5.289696
H	3.086971	6.144496	6.814223
N	5.478921	4.063482	2.837168
K	7.469795	5.100226	4.385058
Si	4.594165	5.293292	2.111060
Si	5.772018	2.514191	2.257644
C	4.539940	5.280945	0.223231
C	2.772150	5.312694	2.643657
C	5.283368	6.991600	2.583689
C	4.223443	1.425178	2.176252
C	6.553648	2.412085	0.540572
C	6.962910	1.594322	3.402567
H	4.001572	6.156691	-0.153300
H	4.030253	4.392126	-0.160137
H	5.545560	5.297781	-0.206443
H	2.208642	6.064597	2.081880
H	2.626239	5.553736	3.702186
H	2.301926	4.342732	2.450797
H	6.300573	7.133280	2.201326
H	5.298084	7.148254	3.666701
H	4.671624	7.795400	2.162683
H	3.472954	1.862310	1.510088
H	3.752543	1.294101	3.156925
H	4.455150	0.423937	1.798556
H	5.864308	2.742506	-0.240438
H	6.849352	1.384101	0.306955
H	7.449373	3.037919	0.476612
H	7.132480	0.574992	3.041607
H	6.580011	1.508860	4.423332
H	7.945670	2.075810	3.455768
C	3.408919	1.019196	7.743963
C	4.377563	0.523753	8.797412
H	5.333965	1.032183	8.676095
F	3.214320	2.341416	7.887696
F	3.873533	0.828720	6.495706
F	2.222085	0.421876	7.816998
H	3.956889	0.720877	9.784024
F	4.552878	-0.829632	8.631983

ch2f-cf3_04a_encountcmplx_dimer_parsep_1_b3pw91.log

SCF (RB3PW91) = -3423.47055364
E(SCF)+ZPE(0 K)= -3422.970896
H(298 K)= -3422.924881
G(298 K)= -3423.052196
Lowest Frequency = 18.8848cm-1

C	-7.537042	0.914430	1.391746
C	-7.071476	-0.312925	0.653226
H	-7.919512	-0.731131	0.109011
H	-6.263344	-0.029181	-0.040786
F	-6.555653	1.446384	2.156741
F	-7.904085	1.861353	0.507670
F	-8.573869	0.693331	2.203901
K	-5.884838	3.246436	-1.213924
N	-4.921801	0.784209	-1.571707
Si	-3.453635	0.415997	-0.858673
Si	-5.620026	0.175259	-2.967613
C	-1.927429	0.908169	-1.860170
C	-3.318911	1.356188	0.784229

C -3.205645 -1.408113 -0.428340
C -7.090713 1.254151 -3.496301
C -4.476020 0.107508 -4.471841
C -6.326493 -1.571528 -2.790470
H -1.965156 1.960565 -2.156936
H -1.855008 0.313045 -2.775509
H -1.003610 0.753764 -1.291716
H -2.338590 1.241838 1.255127
H -4.078466 0.978127 1.475141
H -3.480145 2.421902 0.594283
H -4.038482 -1.793440 0.167923
H -2.283123 -1.570651 0.140055
H -3.142423 -2.015156 -1.337249
H -6.781417 2.248776 -3.839548
H -7.823070 1.375717 -2.689697
H -7.622945 0.794792 -4.335653
H -4.004211 1.078952 -4.650450
H -5.017589 -0.175305 -5.381270
H -3.676285 -0.625161 -4.324379
H -6.736166 -1.939547 -3.737492
H -7.128493 -1.611271 -2.047415
H -5.549469 -2.272430 -2.469931
K -4.625899 3.445320 2.836908
N -5.194562 5.049011 0.785648
Si -3.718535 5.645706 0.245415
Si -6.661610 5.769929 1.173591
C -3.603303 7.506183 -0.033198
C -2.306752 5.228782 1.444418
C -3.184342 4.877265 -1.402849
C -7.407081 5.014153 2.747524
C -6.636577 7.625729 1.505273
C -7.992453 5.518274 -0.154926
H -3.758582 8.070995 0.889399
H -4.344780 7.845836 -0.762133
H -2.613642 7.769078 -0.421782
H -1.369191 5.673576 1.095525
H -2.115813 4.152812 1.526948
H -2.490097 5.633363 2.447003
H -3.118841 3.785390 -1.367261
H -2.186112 5.232566 -1.677653
H -3.850702 5.161634 -2.225740
H -6.816920 5.249170 3.642014
H -7.537317 3.928762 2.684756
H -8.403836 5.430183 2.925874
H -5.910280 7.881396 2.282419
H -7.620953 7.961794 1.848391
H -6.384232 8.200314 0.610806
H -8.890417 6.093993 0.092375
H -8.319563 4.477170 -0.249296
H -7.652297 5.867873 -1.136220
F -6.619764 -1.237091 1.576718

ch2f-cf3_04b_encountcmplx_dimer_parsep_1_pbe0.log

SCF (RPBE1PBE) = -3421.96601569
E(SCF)+ZPE(0 K)= -3421.465822
H(298 K)= -3421.419699
G(298 K)= -3421.546817
Lowest Frequency = 17.9283cm-1

C -7.572668 0.919946 1.053775
C -6.718983 -0.311223 0.912360
H -7.361551 -1.185395 0.797582
H -6.061725 -0.164557 0.043406
F -6.805645 2.029518 1.194599
F -8.319052 1.100721 -0.035327
F -8.381715 0.885966 2.113237
K -5.733365 3.337173 -1.340114
N -4.901752 0.812436 -1.647493
Si -3.417679 0.414771 -0.986276
Si -5.702056 0.195073 -2.983034
C -1.919061 0.899380 -2.030942
C -3.205281 1.328443 0.665067

C -3.187115 -1.417072 -0.573976
C -6.926243 1.466676 -3.683491
C -4.598709 -0.287387 -4.441190
C -6.756020 -1.332612 -2.609854
H -1.942788 1.963999 -2.287136
H -1.898907 0.337104 -2.970107
H -0.976818 0.702948 -1.506902
H -2.212425 1.179307 1.100256
H -3.946551 0.952955 1.378465
H -3.340743 2.403779 0.508357
H -3.980048 -1.778189 0.090023
H -2.228064 -1.606331 -0.078477
H -3.214295 -2.029399 -1.481840
H -6.421819 2.361702 -4.068376
H -7.675711 1.775454 -2.944255
H -7.482635 1.040594 -4.525368
H -3.988497 0.560338 -4.769863
H -5.189816 -0.625703 -5.299730
H -3.916365 -1.100501 -4.171385
H -7.205377 -1.744376 -3.520493
H -7.573831 -1.095450 -1.921235
H -6.153128 -2.123286 -2.150103
K -4.746255 3.291261 2.728221
N -5.199739 5.066589 0.769216
Si -3.687542 5.699618 0.404238
Si -6.704573 5.714106 1.136813
C -3.546155 7.575556 0.288623
C -2.376986 5.190230 1.681951
C -3.020461 5.056107 -1.249401
C -7.343657 5.123295 2.826206
C -6.833744 7.593150 1.207357
C -8.049858 5.174675 -0.086825
H -3.799171 8.064340 1.233787
H -4.204047 7.983972 -0.484173
H -2.519490 7.859790 0.032647
H -1.415939 5.659311 1.445250
H -2.184256 4.110967 1.704290
H -2.644456 5.521999 2.692899
H -2.926475 3.964834 -1.281529
H -2.016540 5.453171 -1.434050
H -3.641989 5.378494 -2.093746
H -6.674093 5.414669 3.645200
H -7.514845 4.041319 2.874437
H -8.311951 5.587382 3.042328
H -6.169492 8.017452 1.965960
H -7.857455 7.891471 1.458916
H -6.581061 8.054398 0.248346
H -9.008467 5.632471 0.179911
H -8.221046 4.092184 -0.088144
H -7.825522 5.497395 -1.110660
F -5.967893 -0.442786 2.063703

ch2f-cf3_04c_encountcmplx_dimer_parsep_1_wb97xd.log

SCF (RwB97XD) = -3423.64924667
E(SCF)+ZPE(0 K)= -3423.145754
H(298 K)= -3423.100453
G(298 K)= -3423.224905
Lowest Frequency = 18.3124cm-1

C -7.575744 0.933575 0.995245
C -6.788150 -0.348962 0.866082
H -7.475482 -1.173574 0.677177
H -6.073227 -0.222621 0.045972
F -6.762164 1.989422 1.240986
F -8.218942 1.199647 -0.144161
F -8.470666 0.909115 1.982360
K -5.836203 3.316490 -1.295269
N -4.894981 0.835131 -1.713340
Si -3.440109 0.457786 -0.989743
Si -5.661655 0.163229 -3.037112
C -1.903213 0.948216 -1.973619
C -3.315001 1.377344 0.666994

C	-3.211024	-1.371053	-0.559650
C	-6.898338	1.384369	-3.798918
C	-4.516615	-0.347347	-4.454115
C	-6.687130	-1.375780	-2.628769
H	-1.913851	2.014770	-2.219328
H	-1.855622	0.395605	-2.917038
H	-0.980771	0.741599	-1.420940
H	-2.341155	1.251699	1.148038
H	-4.083343	0.984279	1.340273
H	-3.475202	2.447655	0.502936
H	-4.025956	-1.735178	0.074165
H	-2.271429	-1.550924	-0.026763
H	-3.198491	-1.986731	-1.464728
H	-6.404542	2.290643	-4.167658
H	-7.679156	1.678398	-3.088270
H	-7.411430	0.936047	-4.655479
H	-3.921310	0.502189	-4.802708
H	-5.077244	-0.735822	-5.310696
H	-3.819465	-1.128864	-4.135333
H	-7.122027	-1.823737	-3.528255
H	-7.512741	-1.134054	-1.952351
H	-6.072105	-2.139231	-2.141491
K	-4.689993	3.318808	2.838200
N	-5.200485	5.077187	0.823493
Si	-3.702232	5.687120	0.380854
Si	-6.685268	5.744528	1.222760
C	-3.549806	7.562432	0.248709
C	-2.330910	5.168913	1.589303
C	-3.110556	5.029824	-1.296882
C	-7.303714	5.164974	2.924288
C	-6.781597	7.626773	1.295590
C	-8.076345	5.226415	0.040357
H	-3.765401	8.057506	1.199172
H	-4.233420	7.971477	-0.500132
H	-2.532599	7.838965	-0.046454
H	-1.380995	5.630885	1.303580
H	-2.149611	4.088141	1.595908
H	-2.541724	5.497851	2.613480
H	-3.025866	3.937931	-1.323122
H	-2.112888	5.417881	-1.523981
H	-3.762604	5.348774	-2.118063
H	-6.621842	5.449656	3.734295
H	-7.482256	4.084782	2.973819
H	-8.264343	5.636991	3.151898
H	-6.087473	8.039320	2.032751
H	-7.790806	7.943802	1.577127
H	-6.548788	8.083467	0.330175
H	-9.015055	5.708973	0.329319
H	-8.270581	4.148531	0.059291
H	-7.875690	5.529859	-0.993333
F	-6.122262	-0.570874	2.055036

ch2f-
cf3_05a_encountcmplx_dimer_parsep_2_b3pw91.log

SCF (RB3PW91) = -3423.47273054
E(SCF)+ZPE(0 K)= -3422.972730
H(298 K)= -3422.926944
G(298 K)= -3423.053048
Lowest Frequency = 15.5129cm-1

C	-7.268415	0.622041	1.744347
C	-7.383793	0.166672	0.312755
H	-6.388768	0.114357	-0.154555
F	-6.519943	-0.234195	2.447297
F	-6.675507	1.841445	1.823066
F	-8.450738	0.739635	2.358722
K	-6.099356	3.113201	-1.065290
N	-4.855984	0.806169	-1.602411
Si	-3.446764	0.413924	-0.794308
Si	-5.445890	0.255950	-3.070849
C	-1.842080	0.846738	-1.695218
C	-3.374756	1.382583	0.835425
C	-3.289622	-1.408317	-0.310598

C	-6.634832	1.519486	-3.842143
C	-4.139157	-0.070305	-4.398125
C	-6.452829	-1.341419	-2.936158
H	-1.830670	1.899166	-1.995093
H	-1.722126	0.245420	-2.600878
H	-0.965688	0.669575	-1.061950
H	-2.417256	1.261884	1.350080
H	-4.170517	1.037177	1.500959
H	-3.511100	2.443507	0.606502
H	-4.163613	-1.741574	0.258722
H	-2.400531	-1.597435	0.301209
H	-3.217308	-2.041967	-1.200605
H	-6.141616	2.477923	-4.042160
H	-7.516537	1.700901	-3.216827
H	-7.010907	1.154975	-4.803732
H	-3.525087	0.818708	-4.572046
H	-4.598468	-0.351815	-5.352043
H	-3.468511	-0.883622	-4.103688
H	-6.774166	-1.705235	-3.918222
H	-7.353187	-1.194135	-2.331529
H	-5.863329	-2.133830	-2.463619
K	-4.491564	3.513218	2.866206
N	-5.270538	5.011306	0.790061
Si	-3.848563	5.591962	0.110494
Si	-6.710014	5.699143	1.310372
C	-3.772031	7.440780	-0.249519
C	-2.336613	5.246128	1.208360
C	-3.442007	4.757352	-1.541791
C	-7.099856	5.262503	3.119644
C	-6.833625	7.578427	1.234822
C	-8.220070	5.060057	0.357366
H	-3.873615	8.039222	0.659615
H	-4.562331	7.746961	-0.940481
H	-2.812066	7.696780	-0.710500
H	-1.439296	5.683267	0.758440
H	-2.119861	4.178937	1.330027
H	-2.439997	5.701501	2.200627
H	-3.385001	3.665975	-1.477691
H	-2.466091	5.094966	-1.905064
H	-4.169424	5.019958	-2.319003
H	-6.337352	5.635719	3.814209
H	-7.240250	4.188113	3.285950
H	-8.041248	5.734316	3.419106
H	-6.058479	8.057830	1.839072
H	-7.804854	7.910392	1.616887
H	-6.735648	7.950789	0.211770
H	-9.130588	5.542212	0.727775
H	-8.375459	3.982636	0.479953
H	-8.160954	5.288361	-0.713097
H	-7.904650	-0.789908	0.279022
F	-8.126524	1.117725	-0.376569

ch2f-
cf3_05b_encountcmplx_dimer_parsep_2_pbe0.log

SCF (RPBE1PBE) = -3421.96721570
E(SCF)+ZPE(0 K)= -3421.466585
H(298 K)= -3421.420676
G(298 K)= -3421.547528
Lowest Frequency = 16.2041cm-1

C	-7.258671	0.675433	1.750110
C	-7.408171	0.215293	0.324469
H	-6.418959	0.096698	-0.140726
F	-6.550246	-0.208746	2.452011
F	-6.606708	1.860621	1.813022
F	-8.426105	0.856623	2.368395
K	-6.026647	3.123038	-1.095104
N	-4.825565	0.776392	-1.611171
Si	-3.427940	0.354922	-0.796617
Si	-5.442133	0.243061	-3.075224
C	-1.812208	0.736940	-1.701056
C	-3.322461	1.338153	0.824777
C	-3.315617	-1.464305	-0.289878

C	-6.612603	1.534315	-3.829052
C	-4.161840	-0.111758	-4.420599
C	-6.485031	-1.330915	-2.937391
H	-1.768536	1.789825	-1.999569
H	-1.712851	0.134203	-2.609101
H	-0.939586	0.531674	-1.070522
H	-2.373739	1.175600	1.345640
H	-4.136909	1.038343	1.491741
H	-3.402732	2.404285	0.587251
H	-4.192760	-1.767717	0.292328
H	-2.426011	-1.667823	0.317082
H	-3.267505	-2.112915	-1.171390
H	-6.103326	2.484115	-4.033303
H	-7.484719	1.730702	-3.193584
H	-7.006548	1.180077	-4.787784
H	-3.527360	0.761528	-4.603951
H	-4.642188	-0.381017	-5.368079
H	-3.507237	-0.942577	-4.136843
H	-6.835436	-1.675195	-3.916928
H	-7.369374	-1.169650	-2.311792
H	-5.905620	-2.143947	-2.486500
K	-4.452864	3.536030	2.836623
N	-5.260749	5.040878	0.775575
Si	-3.845554	5.660016	0.116208
Si	-6.721853	5.681138	1.297889
C	-3.792340	7.517636	-0.198947
C	-2.333221	5.307698	1.211553
C	-3.416390	4.867367	-1.551165
C	-7.094234	5.228525	3.106641
C	-6.907933	7.555404	1.225219
C	-8.209182	4.996041	0.342180
H	-3.912370	8.092782	0.723662
H	-4.579057	7.831126	-0.891564
H	-2.830485	7.797956	-0.642330
H	-1.438472	5.760710	0.771157
H	-2.106521	4.239529	1.313418
H	-2.440270	5.744009	2.212507
H	-3.308497	3.777958	-1.499465
H	-2.458869	5.253098	-1.917168
H	-4.160846	5.106144	-2.320627
H	-6.338986	5.620311	3.799704
H	-7.205191	4.149763	3.271180
H	-8.047280	5.674060	3.411275
H	-6.153077	8.061625	1.834201
H	-7.892493	7.852946	1.602538
H	-6.817788	7.933023	0.202640
H	-9.135689	5.444844	0.716278
H	-8.328421	3.912482	0.456320
H	-8.158550	5.233898	-0.727216
H	-7.986596	-0.708878	0.303064
F	-8.090501	1.200816	-0.368914

ch2f-cf3_05c_encountcmplx_dimer_parsep_2_wb97xd.log

SCF (RwB97XD) = -3423.65014952
E(SCF)+ZPE(0 K)= -3423.146546
H(298 K)= -3423.101305
G(298 K)= -3423.225905
Lowest Frequency = 22.4237cm-1

C	-7.302127	0.667542	1.725220
C	-7.440733	0.173887	0.304078
H	-6.449478	0.059732	-0.150922
F	-6.576833	-0.189108	2.445004
F	-6.672274	1.866340	1.765933
F	-8.473632	0.837744	2.338331
K	-6.065828	3.113563	-1.086410
N	-4.836689	0.784050	-1.662170
Si	-3.433099	0.416731	-0.840646
Si	-5.454374	0.192397	-3.098531
C	-1.830833	0.866053	-1.737753
C	-3.384752	1.388951	0.790351
C	-3.249800	-1.400188	-0.344167

C	-6.666117	1.426909	-3.878608
C	-4.171978	-0.171058	-4.440878
C	-6.449919	-1.406073	-2.898731
H	-1.819167	1.924564	-2.016017
H	-1.720846	0.284459	-2.657844
H	-0.949652	0.673498	-1.116748
H	-2.427369	1.283582	1.308590
H	-4.176825	1.027098	1.452941
H	-3.540102	2.449900	0.570735
H	-4.118683	-1.742305	0.227471
H	-2.358858	-1.570984	0.269329
H	-3.167125	-2.040185	-1.228474
H	-6.184851	2.385761	-4.102593
H	-7.535403	1.613647	-3.238087
H	-7.056775	1.041791	-4.825721
H	-3.566655	0.714417	-4.657671
H	-4.646035	-0.489140	-5.375244
H	-3.489397	-0.968543	-4.130685
H	-6.796684	-1.794669	-3.861872
H	-7.333894	-1.247680	-2.273066
H	-5.844822	-2.185101	-2.423774
K	-4.467585	3.500525	2.916900
N	-5.251825	5.044789	0.818239
Si	-3.826245	5.637455	0.162466
Si	-6.704741	5.713605	1.319191
C	-3.739085	7.496800	-0.142266
C	-2.316245	5.252358	1.250795
C	-3.401864	4.848376	-1.508651
C	-7.113050	5.285400	3.126033
C	-6.855327	7.591505	1.226653
C	-8.201165	5.051811	0.358898
H	-3.859145	8.068075	0.781983
H	-4.513509	7.827731	-0.839471
H	-2.769417	7.764113	-0.574293
H	-1.416808	5.695656	0.812642
H	-2.108959	4.179687	1.338858
H	-2.413221	5.677221	2.256785
H	-3.312584	3.757558	-1.461077
H	-2.436646	5.219565	-1.866616
H	-4.137632	5.101612	-2.280512
H	-6.355796	5.657848	3.825963
H	-7.254178	4.211254	3.292555
H	-8.056562	5.758051	3.415465
H	-6.096180	8.089680	1.835661
H	-7.836156	7.911440	1.592629
H	-6.749741	7.957285	0.201983
H	-9.118973	5.523076	0.723829
H	-8.342461	3.972743	0.484472
H	-8.140968	5.276625	-0.711960
H	-8.003889	-0.758501	0.299185
F	-8.136057	1.133288	-0.414608

ch2f-cf3_06a_encountcmplx_dimer_sep_1_b3pw91.log

SCF (RB3PW91) = -3423.44792776
E(SCF)+ZPE(0 K)= -3422.949595
H(298 K)= -3422.903056
G(298 K)= -3423.032321
Lowest Frequency = 14.5695cm-1

C	-3.936646	2.285080	-0.213993
C	-4.989408	1.222902	-0.057024
H	-5.095360	0.959859	1.001545
H	-4.746528	0.366190	-0.703475
F	-2.749372	1.850720	0.205147
F	-3.789604	2.668672	-1.508823
F	-4.236416	3.407274	0.468542
K	-3.243192	0.973465	-3.713462
N	-4.203145	-0.995198	-2.324662
Si	-2.951538	-1.863588	-1.628739
Si	-5.734140	-1.379884	-2.882734
C	-2.767083	-3.645544	-2.230128
C	-1.289540	-1.025361	-2.015606

C	-3.027045	-1.969067	0.254975
C	-6.426593	0.076060	-3.890210
C	-5.814069	-2.863800	-4.052062
C	-7.008814	-1.721851	-1.533932
H	-2.683048	-3.692576	-3.320238
H	-3.631678	-4.249491	-1.938102
H	-1.875917	-4.119921	-1.804707
H	-0.465541	-1.550480	-1.521675
H	-1.251443	0.009950	-1.656118
H	-1.060410	-1.034148	-3.088492
H	-3.058930	-0.980976	0.722406
H	-2.154297	-2.493869	0.658800
H	-3.917876	-2.514365	0.579651
H	-5.902080	0.212938	-4.844436
H	-6.387415	1.018427	-3.332388
H	-7.477335	-0.094281	-4.146744
H	-5.109269	-2.750224	-4.882273
H	-6.816240	-2.981177	-4.479192
H	-5.565710	-3.794529	-3.533957
H	-7.995440	-1.940352	-1.956931
H	-7.121736	-0.871336	-0.857349
H	-6.709621	-2.584083	-0.930527
K	-7.162705	3.261170	1.652285
N	-6.341384	1.237775	3.032850
Si	-7.334383	-0.096819	2.836215
Si	-5.104317	1.567064	4.110626
C	-8.262018	-0.648551	4.390333
C	-8.699633	0.293067	1.576265
C	-6.436567	-1.632361	2.203101
C	-4.753634	3.431499	4.153791
C	-5.440563	1.076260	5.906604
C	-3.457441	0.749049	3.666701
H	-8.815867	0.184687	4.835066
H	-7.575678	-1.031482	5.151076
H	-8.980481	-1.444253	4.163747
H	-9.317684	-0.589714	1.383884
H	-8.302234	0.612456	0.607835
H	-9.376812	1.071925	1.948815
H	-5.880677	-1.414692	1.289094
H	-7.122448	-2.458559	1.988905
H	-5.715170	-1.983599	2.948196
H	-5.632849	3.998949	4.480783
H	-4.437614	3.816380	3.177911
H	-3.946241	3.664960	4.855911
H	-6.388881	1.491480	6.261855
H	-4.647836	1.432166	6.573907
H	-5.495638	-0.011388	6.013837
H	-2.682908	0.960665	4.412268
H	-3.084311	1.096928	2.699608
H	-3.570024	-0.337771	3.603701
F	-6.195907	1.787998	-0.483033

ch2f-cf3_06b_encountcmplx_dimer_sep_1_pbe0.log

SCF (RPBE1PBE) = -3421.94575503
E(SCF)+ZPE(0 K)= -3421.446800
H(298 K)= -3421.400138
G(298 K)= -3421.530074
Lowest Frequency = 14.1915cm-1

C	-3.935539	2.251645	-0.207704
C	-5.000119	1.202312	-0.052685
H	-5.099591	0.933554	1.005453
H	-4.768627	0.345076	-0.701851
F	-2.758369	1.805399	0.215947
F	-3.780200	2.629733	-1.498508
F	-4.227220	3.372955	0.471550
K	-3.207945	0.953061	-3.694799
N	-4.190995	-1.039605	-2.347735
Si	-2.937245	-1.892113	-1.636871
Si	-5.709561	-1.409235	-2.947203
C	-2.706533	-3.665311	-2.247312
C	-1.285867	-1.017802	-1.987440
C	-3.038527	-2.015675	0.245265

C	-6.334081	0.033979	-4.015428
C	-5.789676	-2.926990	-4.070958
C	-7.042685	-1.678491	-1.636893
H	-2.605779	-3.704945	-3.336812
H	-3.561355	-4.291200	-1.971192
H	-1.810748	-4.122232	-1.811733
H	-0.459329	-1.531740	-1.485157
H	-1.274190	0.014469	-1.615184
H	-1.037730	-1.010508	-3.056644
H	-3.078583	-1.031675	0.723072
H	-2.168978	-2.542441	0.654445
H	-3.932322	-2.567123	0.554019
H	-5.762117	0.146547	-4.945891
H	-6.310583	0.986450	-3.471641
H	-7.374003	-0.126653	-4.319554
H	-5.065175	-2.854165	-4.889056
H	-6.784793	-3.040417	-4.515983
H	-5.570109	-3.845108	-3.517109
H	-8.014402	-1.897454	-2.093918
H	-7.170844	-0.800912	-0.996407
H	-6.785105	-2.522675	-0.989010
K	-7.142678	3.253557	1.677446
N	-6.337794	1.223615	3.068676
Si	-7.354086	-0.093202	2.875277
Si	-5.116270	1.595864	4.149508
C	-8.263062	-0.653082	4.436487
C	-8.729919	0.331918	1.638074
C	-6.498001	-1.636740	2.202198
C	-4.804670	3.468301	4.156664
C	-5.451692	1.134524	5.952777
C	-3.446987	0.809078	3.732860
H	-8.807970	0.177722	4.897361
H	-7.567489	-1.046353	5.184426
H	-8.987722	-1.444179	4.212160
H	-9.370452	-0.536669	1.451469
H	-8.338377	0.643967	0.663532
H	-9.385134	1.124765	2.021420
H	-5.968681	-1.422381	1.270352
H	-7.204917	-2.450078	2.004587
H	-5.757689	-2.006769	2.920102
H	-5.695727	4.025433	4.471032
H	-4.491428	3.840873	3.173977
H	-4.003928	3.732769	4.855993
H	-6.406996	1.542630	6.298801
H	-4.665544	1.515348	6.614600
H	-5.491020	0.047977	6.081758
H	-2.686020	1.046794	4.484940
H	-3.069349	1.153785	2.765201
H	-3.533878	-0.281545	3.681524
F	-6.197978	1.782093	-0.467047

ch2f-cf3_06c_encountcmplx_dimer_sep_1_wb97xd.log

SCF (RwB97XD) = -3423.63094105
E(SCF)+ZPE(0 K)= -3423.129078
H(298 K)= -3423.083096
G(298 K)= -3423.210399
Lowest Frequency = 16.7486cm-1

C	-3.981284	2.323894	-0.275116
C	-5.044901	1.275656	-0.064667
H	-5.094282	1.004816	0.993593
H	-4.840773	0.417934	-0.717089
F	-2.778350	1.854191	0.042605
F	-3.922979	2.729974	-1.565252
F	-4.205003	3.428661	0.452324
K	-3.147711	1.002767	-3.719380
N	-4.155799	-1.002698	-2.356278
Si	-2.943548	-1.852669	-1.578480
Si	-5.687263	-1.387866	-2.904938
C	-2.773982	-3.666293	-2.085117
C	-1.254743	-1.063002	-1.946456
C	-3.078198	-1.872904	0.306486

C	-6.355267	0.033843	-3.973277
C	-5.787263	-2.927121	-3.998893
C	-6.980081	-1.651615	-1.551574
H	-2.656090	-3.771152	-3.167919
H	-3.659548	-4.238811	-1.792789
H	-1.907680	-4.135285	-1.607033
H	-0.452869	-1.594483	-1.424908
H	-1.202592	-0.021186	-1.609263
H	-1.007852	-1.097786	-3.014361
H	-3.134507	-0.864783	0.726822
H	-2.213684	-2.368353	0.760774
H	-3.972632	-2.413622	0.628603
H	-5.805927	0.138717	-4.916791
H	-6.318866	0.991715	-3.441810
H	-7.401224	-0.136280	-4.246486
H	-5.083395	-2.865612	-4.834868
H	-6.791224	-3.056126	-4.416838
H	-5.547106	-3.832033	-3.433218
H	-7.963165	-1.879990	-1.976643
H	-7.095151	-0.770294	-0.914464
H	-6.698634	-2.487639	-0.904230
K	-7.233282	3.354949	1.728335
N	-6.422303	1.279453	3.089754
Si	-7.379601	-0.069970	2.844286
Si	-5.115746	1.546095	4.096508
C	-8.269507	-0.712140	4.385850
C	-8.761032	0.324763	1.605160
C	-6.450265	-1.556560	2.139756
C	-4.744327	3.402670	4.209296
C	-5.337995	0.955709	5.880237
C	-3.506182	0.745281	3.502234
H	-8.858129	0.078986	4.861053
H	-7.556935	-1.082357	5.128806
H	-8.950250	-1.534843	4.142620
H	-9.369742	-0.561251	1.400195
H	-8.369167	0.666074	0.641744
H	-9.442715	1.091396	1.992897
H	-5.916520	-1.289392	1.225236
H	-7.119291	-2.390006	1.902996
H	-5.707676	-1.920146	2.857430
H	-5.601863	3.959810	4.602775
H	-4.477166	3.826937	3.235016
H	-3.899862	3.600248	4.877106
H	-6.262752	1.345933	6.316280
H	-4.505977	1.273299	6.517365
H	-5.388106	-0.136560	5.927229
H	-2.690878	0.890472	4.218922
H	-3.177359	1.164715	2.547109
H	-3.635284	-0.332263	3.359687
F	-6.260896	1.854859	-0.423728

ch2f-cf3_07a_encountcmplx_dimer_sep_2_b3pw91.log

SCF (RB3PW91) = -3423.45113292
E(SCF)+ZPE(0 K)= -3422.952376
H(298 K)= -3422.905863
G(298 K)= -3423.034764
Lowest Frequency = 19.7444cm-1

C	-6.700989	4.041374	0.372031
C	-7.475211	2.748752	0.330386
H	-7.182236	2.131724	1.194856
F	-5.369939	3.821440	0.293591
F	-7.015494	4.890322	-0.604704
F	-6.925293	4.662518	1.549273
K	-4.551178	1.037020	-1.188600
N	-3.410759	-1.142592	-1.994836
Si	-1.901005	-1.439741	-1.342493
Si	-4.511091	-2.157846	-2.739226
C	-0.740922	-2.462660	-2.433893
C	-0.989898	0.192342	-1.021502
C	-1.950463	-2.338025	0.324211
C	-6.176106	-1.274552	-2.967153

C	-4.001996	-2.713912	-4.475597
C	-4.905688	-3.748886	-1.792293
H	-0.625847	-2.005185	-3.421811
H	-1.135398	-3.473123	-2.582740
H	0.256726	-2.561038	-1.991592
H	0.010001	0.018238	-0.609565
H	-1.515507	0.832971	-0.304453
H	-0.868401	0.759318	-1.950636
H	-2.547564	-1.781834	1.052913
H	-0.950763	-2.477484	0.750786
H	-2.407802	-3.326846	0.218354
H	-6.071967	-0.349306	-3.546519
H	-6.650625	-1.034017	-2.009570
H	-6.882182	-1.908127	-3.514031
H	-3.808885	-1.849262	-5.119433
H	-4.773894	-3.324652	-4.956953
H	-3.082734	-3.307060	-4.440753
H	-5.690020	-4.334197	-2.285156
H	-5.243262	-3.526693	-0.775372
H	-4.019431	-4.387036	-1.714407
K	-5.432730	3.476819	3.780495
N	-6.415435	1.189829	3.071573
Si	-7.928767	0.909285	3.743951
Si	-5.279504	0.108745	2.482002
C	-7.928862	-0.226566	5.252510
C	-8.665694	2.554249	4.333204
C	-9.184708	0.165016	2.542992
C	-3.704742	1.063006	2.003259
C	-4.674371	-1.201334	3.695485
C	-5.868602	-0.859116	0.963322
H	-7.203823	0.110884	6.000117
H	-7.661741	-1.250462	4.974555
H	-8.913419	-0.261218	5.731586
H	-9.694069	2.422860	4.684860
H	-8.696257	3.300272	3.531233
H	-8.106692	2.977825	5.176893
H	-9.314538	0.783112	1.649695
H	-10.168615	0.045854	3.009395
H	-8.856319	-0.824328	2.209439
H	-3.168798	1.376367	2.907127
H	-3.887085	1.971749	1.419094
H	-3.000446	0.435702	1.449306
H	-4.301783	-0.743520	4.617381
H	-3.863814	-1.799361	3.265561
H	-5.480687	-1.887887	3.969059
H	-5.052541	-1.275163	0.362844
H	-6.523827	-0.265749	0.318332
H	-6.474629	-1.706873	1.297095
H	-8.543303	2.963663	0.328810
F	-7.141293	2.091474	-0.848170

ch2f-cf3_07b_encountcmplx_dimer_sep_2_pbe0.log

SCF (RPBE1PBE) = -3421.94675398
E(SCF)+ZPE(0 K)= -3421.447491
H(298 K)= -3421.400802
G(298 K)= -3421.531245
Lowest Frequency = 13.3932cm-1

C	-6.699359	3.995976	0.361826
C	-7.492487	2.717883	0.276840
H	-7.256926	2.099082	1.155271
F	-5.374865	3.753657	0.330701
F	-6.962939	4.854920	-0.616309
F	-6.956237	4.609289	1.531449
K	-4.553353	0.921268	-1.191079
N	-3.357233	-1.224093	-2.007477
Si	-1.811837	-1.449825	-1.412698
Si	-4.435477	-2.240736	-2.780845
C	-0.619350	-2.334414	-2.586417
C	-1.005147	0.224055	-1.033215
C	-1.742329	-2.427323	0.207459
C	-6.122886	-1.389806	-2.964974
C	-3.931164	-2.726398	-4.538996

C	-4.785019	-3.870795	-1.885192
H	-0.572196	-1.827259	-3.555973
H	-0.943638	-3.364668	-2.769252
H	0.397848	-2.377225	-2.180600
H	0.019666	0.100074	-0.665715
H	-1.546535	0.784384	-0.261504
H	-0.957038	0.848717	-1.932158
H	-2.346193	-1.945642	0.984088
H	-0.719922	-2.521056	0.591323
H	-2.139443	-3.438597	0.068642
H	-6.048954	-0.449028	-3.524773
H	-6.590836	-1.183034	-1.995248
H	-6.822269	-2.026392	-3.517755
H	-3.769908	-1.837163	-5.158161
H	-4.692788	-3.342872	-5.030010
H	-2.995645	-3.295680	-4.534640
H	-5.564066	-4.456100	-2.386938
H	-5.112450	-3.690889	-0.855690
H	-3.884221	-4.492653	-1.840404
K	-5.449897	3.494733	3.771629
N	-6.473717	1.201668	3.137286
Si	-7.985584	1.010979	3.841993
Si	-5.383114	0.080261	2.539773
C	-8.030393	-0.135510	5.341543
C	-8.605028	2.695181	4.457304
C	-9.319041	0.364353	2.668410
C	-3.780619	0.971734	2.034323
C	-4.810174	-1.251174	3.745883
C	-6.020814	-0.869802	1.028000
H	-7.271541	0.146837	6.078906
H	-7.836055	-1.172975	5.051127
H	-9.006610	-0.110220	5.838677
H	-9.631965	2.623809	4.831019
H	-8.611882	3.446079	3.657856
H	-8.005027	3.080745	5.291621
H	-9.450841	1.015044	1.798110
H	-10.290072	0.281149	3.169110
H	-9.054660	-0.630727	2.295279
H	-3.232735	1.294093	2.928505
H	-3.937782	1.870178	1.425922
H	-3.097596	0.306385	1.495601
H	-4.400702	-0.807819	4.659641
H	-4.032499	-1.883024	3.302208
H	-5.638396	-1.904408	4.037434
H	-5.226486	-1.365831	0.458281
H	-6.612459	-0.241713	0.353447
H	-6.700314	-1.659478	1.366012
H	-8.555853	2.951301	0.218043
F	-7.105864	2.057574	-0.878401

ch2f-cf3_07c_encountcmplx_dimer_sep_2_wb97xd.log

SCF (RwB97XD) = -3423.63192521
E(SCF)+ZPE(0 K)= -3423.128546
H(298 K)= -3423.083283
G(298 K)= -3423.207312
Lowest Frequency = 20.7698cm-1

C	-7.137000	4.024892	0.488251
C	-7.573284	2.629725	0.106098
H	-7.327882	1.956239	0.933430
F	-5.807751	4.078888	0.717858
F	-7.406571	4.937628	-0.441253
F	-7.745171	4.394752	1.624224
K	-4.379786	1.077046	-1.388102
N	-3.308328	-1.221494	-2.041983
Si	-1.825191	-1.508192	-1.332844
Si	-4.429589	-2.251154	-2.727650
C	-0.658692	-2.619239	-2.326124
C	-0.888272	0.120243	-1.071577
C	-1.941505	-2.298526	0.384460
C	-6.066950	-1.340200	-3.031942
C	-3.919351	-2.946366	-4.412517

C	-4.881085	-3.761167	-1.678193
H	-0.501534	-2.224827	-3.334855
H	-1.074278	-3.626900	-2.428538
H	0.321508	-2.715097	-1.847354
H	0.101153	-0.054316	-0.636540
H	-1.412833	0.797926	-0.387970
H	-0.741374	0.647374	-2.019757
H	-2.526511	-1.669046	1.062380
H	-0.957295	-2.451426	0.840021
H	-2.440290	-3.271504	0.334022
H	-5.931434	-0.461704	-3.673495
H	-6.533866	-1.016807	-2.094935
H	-6.790980	-1.988764	-3.535027
H	-3.710540	-2.139364	-5.122113
H	-4.696320	-3.583029	-4.848716
H	-3.008494	-3.546971	-4.324294
H	-5.669326	-4.360562	-2.146045
H	-5.233963	-3.460899	-0.686785
H	-4.013535	-4.413633	-1.536188
K	-5.501020	3.615261	3.724793
N	-6.439224	1.262992	3.048179
Si	-7.907336	0.968351	3.798878
Si	-5.300967	0.231377	2.393291
C	-7.976951	-0.602238	4.846051
C	-8.347911	2.394577	4.970528
C	-9.372792	0.852229	2.606939
C	-3.775442	1.231022	1.847050
C	-4.601139	-1.083204	3.551483
C	-5.902724	-0.737962	0.879510
H	-7.172342	-0.623334	5.587070
H	-7.874301	-1.495872	4.222738
H	-8.928202	-0.683258	5.381875
H	-9.311236	2.215576	5.457931
H	-8.445081	3.349699	4.440841
H	-7.608600	2.512314	5.771123
H	-9.573049	1.812085	2.121087
H	-10.287938	0.555980	3.129703
H	-9.186381	0.113168	1.821147
H	-3.212511	1.574965	2.722201
H	-4.018962	2.124866	1.261398
H	-3.074274	0.616330	1.272914
H	-4.193194	-0.632892	4.461763
H	-3.797960	-1.650080	3.069395
H	-5.372581	-1.797286	3.852569
H	-5.086141	-1.228555	0.338678
H	-6.474003	-0.126416	0.173941
H	-6.585897	-1.527964	1.207803
H	-8.638350	2.626018	-0.119980
F	-6.870089	2.255805	-1.030727

ch2f-cf3_08a_TS-H_dimer_intact_1_b3pw91.log

SCF (RB3PW91) = -3423.45152091
E(SCF)+ZPE(0 K)= -3422.956262
H(298 K)= -3422.911508
G(298 K)= -3423.032629
Lowest Frequency = -1082.8475cm-1

C	-5.381854	2.312049	-1.207461
C	-5.218816	0.854669	-1.026837
H	-6.211686	0.456930	-0.793573
H	-4.611129	-0.125158	-2.032918
F	-4.205349	2.949470	-1.498286
F	-6.206015	2.554422	-2.263914
F	-5.894961	3.018753	-0.165308
K	-4.097259	1.531006	-3.975711
N	-4.168951	-1.080982	-2.700922
Si	-2.678172	-1.524313	-1.951598
Si	-5.582230	-2.052611	-2.897440
C	-1.667884	-2.788429	-2.930836
C	-1.622121	0.021543	-1.838555
C	-2.877939	-2.281901	-0.248813
C	-6.910838	-0.983059	-3.677164
C	-5.341035	-3.536858	-4.040465

C	-6.238250	-2.743563	-1.281916
H	-1.124425	-2.371773	-3.784412
H	-2.248144	-3.656514	-3.257104
H	-0.892306	-3.174221	-2.261130
H	-0.680070	-0.188019	-1.323055
H	-2.127806	0.816556	-1.284775
H	-1.358269	0.399142	-2.831805
H	-3.537990	-1.683283	0.381257
H	-1.907106	-2.356962	0.251688
H	-3.293029	-3.291486	-0.314473
H	-6.602808	-0.611754	-4.658783
H	-7.159700	-0.122119	-3.051464
H	-7.830444	-1.556900	-3.827285
H	-5.378338	-3.292637	-5.107143
H	-6.177603	-4.223719	-3.875971
H	-4.429523	-4.103449	-3.831196
H	-7.231603	-3.180671	-1.425557
H	-6.320430	-1.966115	-0.518385
H	-5.584871	-3.526717	-0.888881
N	-3.231494	0.693296	-6.406713
K	-3.144051	-1.800060	-5.488714
Si	-1.656832	1.266744	-6.482536
Si	-4.592236	0.799726	-7.381742
C	-1.062181	1.885904	-8.159369
C	-1.335770	2.693312	-5.272109
C	-0.418534	-0.071982	-5.963726
C	-4.338192	1.476553	-9.121344
C	-5.401450	-0.900752	-7.613771
C	-5.953121	1.880726	-6.619759
H	-0.007489	2.176136	-8.107457
H	-1.629663	2.758561	-8.493223
H	-1.156683	1.113293	-8.927650
H	-0.332659	3.104624	-5.425728
H	-1.372036	2.387455	-4.220076
H	-2.042981	3.517211	-5.418805
H	-0.436613	-0.938152	-6.635619
H	-0.584462	-0.419394	-4.938066
H	0.603355	0.320014	-5.987668
H	-3.606320	0.887039	-9.680251
H	-3.987582	2.512070	-9.102692
H	-5.279611	1.455751	-9.680493
H	-4.730481	-1.605912	-8.118411
H	-6.297146	-0.819151	-8.237784
H	-5.733763	-1.347431	-6.670086
H	-6.795784	1.977554	-7.312177
H	-5.593559	2.895491	-6.414828
H	-6.373585	1.468327	-5.695795
F	-4.428509	0.686026	0.143421

ch2f-cf3_08b_TS-H_dimer_intact_1_pbe0.log

SCF (RPBE1PBE) = -3421.94272839
E(SCF)+ZPE(0 K)= -3421.446655
H(298 K)= -3421.401940
G(298 K)= -3421.522869
Lowest Frequency = -1089.8756cm-1

C	-5.375500	2.323508	-1.225683
C	-5.224514	0.866511	-1.032313
H	-6.222877	0.481811	-0.795025
H	-4.621653	-0.124210	-2.031242
F	-4.196101	2.945704	-1.517444
F	-6.190901	2.562600	-2.284166
F	-5.883887	3.038446	-0.194274
K	-4.090288	1.549293	-3.982888
N	-4.178159	-1.085194	-2.689868
Si	-2.685738	-1.521982	-1.937461
Si	-5.588239	-2.062745	-2.889814
C	-1.670079	-2.783381	-2.915116
C	-1.630921	0.025188	-1.819739
C	-2.878662	-2.273470	-0.230629
C	-6.932752	-0.996898	-3.648374
C	-5.346640	-3.531789	-4.053020
C	-6.236138	-2.777153	-1.280672

H	-1.124736	-2.364340	-3.767397
H	-2.247235	-3.653305	-3.244256
H	-0.895071	-3.168375	-2.243400
H	-0.693699	-0.186230	-1.295062
H	-2.139950	0.821458	-1.269168
H	-1.356025	0.403443	-2.810617
H	-3.531162	-1.668295	0.402637
H	-1.903692	-2.349428	0.262522
H	-3.298276	-3.282275	-0.286472
H	-6.639596	-0.614533	-4.631222
H	-7.184040	-0.142749	-3.013154
H	-7.848438	-1.579548	-3.792506
H	-5.391951	-3.273367	-5.116768
H	-6.180439	-4.223761	-3.892244
H	-4.432053	-4.099631	-3.857182
H	-7.225153	-3.223014	-1.430288
H	-6.328333	-2.007313	-0.509364
H	-5.575809	-3.557833	-0.892741
N	-3.224740	0.697420	-6.416110
K	-3.138154	-1.801252	-5.491686
Si	-1.647903	1.266680	-6.493284
Si	-4.589060	0.801944	-7.387520
C	-1.056657	1.906846	-8.163435
C	-1.317542	2.677750	-5.267468
C	-0.409966	-0.081552	-5.998115
C	-4.345782	1.482935	-9.127037
C	-5.394693	-0.899919	-7.620485
C	-5.950752	1.877968	-6.619541
H	-0.001918	2.197596	-8.107803
H	-1.624905	2.783871	-8.486380
H	-1.150102	1.144319	-8.942580
H	-0.311085	3.083705	-5.416775
H	-1.356315	2.362291	-4.217674
H	-2.018364	3.509153	-5.406333
H	-0.442419	-0.944063	-6.675002
H	-0.560552	-0.435067	-4.971170
H	0.613874	0.305686	-6.036099
H	-3.615465	0.896766	-9.692574
H	-3.998476	2.520170	-9.109653
H	-5.291122	1.459851	-9.680164
H	-4.720647	-1.605081	-8.122101
H	-6.288009	-0.819958	-8.248999
H	-5.731153	-1.346886	-6.677471
H	-6.796093	1.972019	-7.309863
H	-5.594669	2.894921	-6.416404
H	-6.368297	1.464895	-5.693592
F	-4.438714	0.704155	0.136366

ch2f-cf3_08c_TS-H_dimer_intact_1_wb97xd.log

SCF (RwB97XD) = -3423.62242540
E(SCF)+ZPE(0 K)= -3423.123062
H(298 K)= -3423.079120
G(298 K)= -3423.197890
Lowest Frequency = -1266.3314cm-1

C	-5.369167	2.287487	-1.159620
C	-5.219735	0.823075	-0.966834
H	-6.218468	0.443550	-0.726586
H	-4.618288	-0.140430	-1.972163
F	-4.191723	2.907338	-1.458890
F	-6.190221	2.527478	-2.215056
F	-5.873282	2.996360	-0.125010
K	-4.123037	1.503019	-3.977213
N	-4.172792	-1.094304	-2.661889
Si	-2.675409	-1.527926	-1.930765
Si	-5.588260	-2.049835	-2.878476
C	-1.657085	-2.777709	-2.921474
C	-1.634615	0.029414	-1.816885
C	-2.861572	-2.296601	-0.230437
C	-6.895417	-0.957106	-3.665665
C	-5.360324	-3.529886	-4.030992
C	-6.261419	-2.737079	-1.267867
H	-1.107937	-2.343159	-3.762770

H	-2.237221	-3.637657	-3.269662
H	-0.887432	-3.179121	-2.255170
H	-0.690445	-0.164635	-1.300165
H	-2.152829	0.819111	-1.266789
H	-1.376087	0.409382	-2.811578
H	-3.525659	-1.708311	0.405104
H	-1.890339	-2.369207	0.268413
H	-3.270234	-3.308544	-0.302188
H	-6.574852	-0.603254	-4.650801
H	-7.121681	-0.084090	-3.047753
H	-7.830104	-1.505644	-3.813517
H	-5.426230	-3.276232	-5.094369
H	-6.183669	-4.228059	-3.852319
H	-4.437232	-4.087182	-3.848367
H	-7.254961	-3.171342	-1.414331
H	-6.343244	-1.958029	-0.505892
H	-5.612361	-3.521411	-0.869553
N	-3.230496	0.686171	-6.442328
K	-3.118420	-1.846410	-5.538086
Si	-1.660394	1.269523	-6.504622
Si	-4.586987	0.795649	-7.420663
C	-1.058593	1.896289	-8.176877
C	-1.349699	2.693574	-5.288070
C	-0.413763	-0.058105	-5.974217
C	-4.322930	1.470294	-9.160490
C	-5.407809	-0.899812	-7.653362
C	-5.951991	1.878625	-6.668116
H	-0.004998	2.187342	-8.119607
H	-1.625380	2.770003	-8.509048
H	-1.150013	1.128757	-8.950329
H	-0.352393	3.116480	-5.444201
H	-1.377254	2.378227	-4.238289
H	-2.066520	3.510735	-5.422893
H	-0.418457	-0.928851	-6.639950
H	-0.585934	-0.400888	-4.947265
H	0.604301	0.342620	-5.992067
H	-3.583013	0.885222	-9.713204
H	-3.978258	2.507700	-9.141874
H	-5.259294	1.443372	-9.726747
H	-4.743131	-1.611933	-8.155863
H	-6.302008	-0.811600	-8.277794
H	-5.743705	-1.342202	-6.708085
H	-6.782384	1.989888	-7.372254
H	-5.590268	2.887666	-6.441739
H	-6.388547	1.455017	-5.756461
F	-4.436719	0.661497	0.208668

ch2f-cf3_09a_TS-H_dimer_intact_2_b3pw91.log

SCF (RB3PW91) = -3423.45150142
E(SCF)+ZPE(0 K)= -3422.956193
H(298 K)= -3422.911446
G(298 K)= -3423.033294
Lowest Frequency = -1083.3088cm-1

C	-5.236344	2.324972	-1.146407
C	-4.997391	0.880756	-0.946139
H	-4.549613	-0.121406	-2.013145
F	-4.078228	2.933091	-1.525398
F	-6.137641	2.584949	-2.143805
F	-5.700012	3.036187	-0.084446
K	-4.280172	1.498297	-4.035755
N	-4.183967	-1.085990	-2.713630
Si	-2.705218	-1.531821	-1.943062
Si	-5.593418	-2.056340	-2.941951
C	-1.746385	-2.888291	-2.841606
C	-1.597187	-0.018377	-1.913700
C	-2.938483	-2.141728	-0.184984
C	-6.943328	-0.941986	-3.615426
C	-5.372484	-3.457687	-4.192881
C	-6.196928	-2.901228	-1.380971
H	-1.175639	-2.536391	-3.707256
H	-2.367846	-3.737693	-3.138782
H	-0.996918	-3.281064	-2.146813

H	-0.645565	-0.243886	-1.422817
H	-2.056448	0.812298	-1.371550
H	-1.359663	0.325017	-2.924767
H	-3.568718	-1.463317	0.395139
H	-1.973327	-2.223433	0.325150
H	-3.408145	-3.128533	-0.166000
H	-6.679988	-0.536516	-4.597511
H	-7.149466	-0.109125	-2.938565
H	-7.876857	-1.497741	-3.745264
H	-5.401275	-3.144248	-5.240746
H	-6.224479	-4.135281	-4.075081
H	-4.476685	-4.060518	-4.017218
H	-7.186935	-3.339569	-1.543517
H	-6.270572	-2.199340	-0.548496
H	-5.523137	-3.710462	-1.085827
N	-3.260450	0.684848	-6.419675
K	-3.088121	-1.791786	-5.487618
Si	-1.706243	1.315596	-6.435152
Si	-4.588982	0.750891	-7.441633
C	-1.107605	2.046508	-8.064992
C	-1.463528	2.691541	-5.150688
C	-0.427025	-0.007122	-5.971870
C	-4.257151	1.253703	-9.226427
C	-5.453116	-0.933905	-7.534732
C	-5.937495	1.931970	-6.817283
H	-0.069150	2.382659	-7.975967
H	-1.709673	2.909153	-8.363469
H	-1.151674	1.313362	-8.875038
H	-0.461918	3.124747	-5.240436
H	-1.544713	2.342513	-4.115160
H	-2.177001	3.510273	-5.297745
H	-0.421391	-0.837710	-6.687615
H	-0.577187	-0.413423	-4.965372
H	0.582505	0.416324	-5.976158
H	-3.515594	0.598648	-9.692711
H	-3.886378	2.279563	-9.297243
H	-5.175902	1.191062	-9.819114
H	-4.812927	-1.710021	-7.970719
H	-6.344598	-0.874750	-8.167243
H	-5.800418	-1.274047	-6.553028
H	-6.729640	2.039539	-7.565542
H	-5.539155	2.934783	-6.627377
H	-6.436358	1.578550	-5.907147
H	-4.268613	0.795605	-0.133686
F	-6.217651	0.340638	-0.454681

ch2f-cf3_09c_TS-H_dimer_intact_2_wb97xd.log

SCF (RwB97XD) = -3423.62241205
E(SCF)+ZPE(0 K)= -3423.123195
H(298 K)= -3423.079144
G(298 K)= -3423.199145
Lowest Frequency = -1270.1803cm-1

C	-5.253150	2.299205	-1.119036
C	-4.997920	0.854719	-0.891657
H	-4.564459	-0.136529	-1.954792
F	-4.106967	2.906314	-1.523325
F	-6.162490	2.529393	-2.109390
F	-5.706317	3.017899	-0.067852
K	-4.299982	1.466211	-4.037608
N	-4.198797	-1.102380	-2.672456
Si	-2.709495	-1.535285	-1.925625
Si	-5.608251	-2.062014	-2.909120
C	-1.749451	-2.886225	-2.832998
C	-1.614539	-0.011148	-1.920339
C	-2.920629	-2.135805	-0.160934
C	-6.959616	-0.929767	-3.552239
C	-5.408584	-3.437161	-4.192981
C	-6.198477	-2.932210	-1.356036
H	-1.160383	-2.514841	-3.678291
H	-2.376020	-3.718946	-3.165245
H	-1.017467	-3.307943	-2.137707
H	-0.655986	-0.215441	-1.434765

H	-2.081664	0.821580	-1.387692
H	-1.391081	0.319046	-2.939816
H	-3.543113	-1.452247	0.421678
H	-1.951504	-2.219051	0.340129
H	-3.394488	-3.120455	-0.131330
H	-6.708478	-0.527754	-4.539858
H	-7.139361	-0.093493	-2.871931
H	-7.903918	-1.469909	-3.664691
H	-5.468584	-3.093849	-5.230450
H	-6.248151	-4.128433	-4.071317
H	-4.501221	-4.033446	-4.057633
H	-7.192359	-3.363328	-1.509975
H	-6.254572	-2.246240	-0.508946
H	-5.524096	-3.749619	-1.085194
N	-3.248429	0.672244	-6.454332
K	-3.062080	-1.846284	-5.538282
Si	-1.699960	1.314282	-6.454568
Si	-4.570497	0.752944	-7.481806
C	-1.091023	2.047713	-8.080501
C	-1.472348	2.692750	-5.169539
C	-0.411395	0.005676	-5.975515
C	-4.223376	1.257286	-9.264159
C	-5.457059	-0.920247	-7.583860
C	-5.916342	1.942762	-6.866593
H	-0.051852	2.378331	-7.986070
H	-1.687030	2.914434	-8.378926
H	-1.135839	1.318886	-8.894226
H	-0.477862	3.140064	-5.263007
H	-1.544356	2.339146	-4.134639
H	-2.198314	3.501214	-5.309730
H	-0.384438	-0.824230	-6.690898
H	-0.572623	-0.404218	-4.971605
H	0.592227	0.441521	-5.963956
H	-3.482990	0.599960	-9.728638
H	-3.845138	2.280648	-9.330257
H	-5.138380	1.202791	-9.862409
H	-4.826164	-1.707284	-8.012856
H	-6.341485	-0.847338	-8.224050
H	-5.817549	-1.254933	-6.604369
H	-6.690763	2.072129	-7.629037
H	-5.511848	2.937245	-6.649622
H	-6.437877	1.579029	-5.973154
H	-4.261267	0.798796	-0.083270
F	-6.206911	0.319762	-0.368892

ch2f-cf3_10a_TS-H_dimer_parsep_1_b3pw91.log

SCF (RB3PW91) = -3423.44874700
E(SCF)+ZPE(0 K)= -3422.955048
H(298 K)= -3422.909454
G(298 K)= -3423.034869
Lowest Frequency = -1119.9346cm-1

C	-4.888408	0.835573	2.478724
C	-5.321460	-0.219003	1.548278
H	-6.368721	-0.448659	1.767321
H	-5.120109	0.154605	0.108771
F	-3.599383	1.234047	2.275107
F	-5.654821	1.955036	2.279985
F	-4.953307	0.589057	3.816636
K	-3.912292	2.859596	-0.151272
N	-5.018753	0.456192	-1.104439
Si	-3.656228	-0.191505	-1.911737
Si	-6.609340	0.528878	-1.727794
C	-3.958366	-1.880276	-2.677860
C	-3.052004	0.944220	-3.289038
C	-2.243430	-0.367587	-0.682544
C	-7.562973	1.789215	-0.697278
C	-6.697167	1.053958	-3.527138
C	-7.525409	-1.104354	-1.572907
H	-4.726927	-1.833272	-3.454931
H	-4.292337	-2.594886	-1.919831
H	-3.047638	-2.277920	-3.137347
H	-2.153963	0.529625	-3.759324

H	-2.790673	1.946120	-2.932007
H	-3.807776	1.065769	-4.068988
H	-1.889356	0.589040	-0.284241
H	-1.383074	-0.840408	-1.166760
H	-2.532338	-0.990519	0.167379
H	-7.066883	2.760700	-0.771110
H	-7.579429	1.451401	0.343146
H	-8.594323	1.907833	-1.039401
H	-6.203785	2.012284	-3.705122
H	-7.742243	1.153361	-3.839191
H	-6.230974	0.310142	-4.179353
H	-8.574123	-1.008427	-1.872896
H	-7.501550	-1.469524	-0.541968
H	-7.066611	-1.871192	-2.203445
K	-7.484690	4.026352	1.631250
N	-5.310043	5.123927	0.533705
Si	-4.452791	5.738793	1.842408
Si	-5.781434	5.751451	-0.952329
C	-4.137564	7.596636	1.853674
C	-5.331705	5.389308	3.489560
C	-2.748730	4.932814	2.046811
C	-7.669925	5.789672	-1.137531
C	-5.206984	7.497054	-1.367902
C	-5.169017	4.686103	-2.396572
H	-5.072683	8.161290	1.797756
H	-3.508083	7.910734	1.017499
H	-3.629473	7.887694	2.779180
H	-4.724742	5.767626	4.318357
H	-5.472989	4.320540	3.685470
H	-6.300127	5.899011	3.566208
H	-2.136571	5.037081	1.143651
H	-2.807472	3.871760	2.312452
H	-2.195103	5.415735	2.858546
H	-8.136866	6.391531	-0.349116
H	-8.128434	4.794534	-1.135941
H	-7.951296	6.248491	-2.090945
H	-5.603732	8.238001	-0.669423
H	-5.542309	7.774348	-2.373001
H	-4.116032	7.572557	-1.351391
H	-5.562737	5.065527	-3.344692
H	-5.492726	3.642880	-2.330590
H	-4.076341	4.707490	-2.484614
F	-4.566596	-1.382913	1.850097

ch2f-cf3_10b_TS-H_dimer_parsep_1_pbe0.log

SCF (RPBE1PBE) = -3421.94174436
E(SCF)+ZPE(0 K)= -3421.446735
H(298 K)= -3421.401479
G(298 K)= -3421.525138
Lowest Frequency = -1141.7230cm-1

C	-5.244728	0.894115	2.450047
C	-5.246529	-0.152753	1.414353
H	-6.112174	-0.797946	1.598499
H	-5.154308	0.267478	-0.026895
F	-4.246899	1.808798	2.278331
F	-6.408230	1.599145	2.406528
F	-5.108571	0.502628	3.741270
K	-3.899855	2.909053	-0.295421
N	-5.014751	0.503060	-1.250327
Si	-3.661629	-0.337591	-1.884877
Si	-6.578174	0.633395	-1.933641
C	-3.756387	-2.190688	-1.593606
C	-3.414638	-0.075228	-3.728771
C	-2.094982	0.289234	-1.038934
C	-7.557822	1.832611	-0.854613
C	-6.608193	1.307757	-3.685882
C	-7.520670	-0.992747	-1.951579
H	-4.630500	-2.618226	-2.095160
H	-3.841719	-2.415180	-0.526638
H	-2.867488	-2.702777	-1.977085
H	-2.452773	-0.501019	-4.035682
H	-3.408514	0.985943	-3.995271

H	-4.192880	-0.566007	-4.319709
H	-1.788366	1.278844	-1.399226
H	-1.257305	-0.381279	-1.257181
H	-2.199335	0.320466	0.050642
H	-7.053145	2.804183	-0.847608
H	-7.627314	1.438785	0.163664
H	-8.571443	1.984163	-1.236623
H	-6.021431	2.224361	-3.791381
H	-7.639980	1.539355	-3.973008
H	-6.223500	0.580677	-4.405685
H	-8.555971	-0.854831	-2.281604
H	-7.541664	-1.445981	-0.955410
H	-7.050065	-1.710627	-2.630966
K	-7.504063	4.030044	1.615085
N	-5.298863	5.134535	0.582127
Si	-4.402046	5.646378	1.910441
Si	-5.781628	5.880196	-0.845459
C	-4.167969	7.506084	2.113544
C	-5.195581	5.085298	3.540007
C	-2.650390	4.920472	1.947365
C	-7.671220	5.900462	-1.025500
C	-5.241393	7.665348	-1.113059
C	-5.163424	4.950670	-2.377519
H	-5.127783	8.031433	2.137122
H	-3.571156	7.938035	1.305750
H	-3.651739	7.717794	3.056490
H	-4.545086	5.348790	4.380837
H	-5.342637	4.001176	3.599216
H	-6.153267	5.586156	3.731077
H	-2.106045	5.134313	1.019893
H	-2.630609	3.839513	2.124927
H	-2.071807	5.368938	2.762201
H	-8.151424	6.430360	-0.193612
H	-8.110673	4.899067	-1.106149
H	-7.960943	6.429456	-1.939752
H	-5.644896	8.337227	-0.350582
H	-5.592156	8.020805	-2.088272
H	-4.151477	7.761374	-1.099741
H	-5.538831	5.424181	-3.290772
H	-5.508863	3.911994	-2.410959
H	-4.068931	4.959956	-2.450341
F	-4.086536	-0.936081	1.645086

ch2f-cf3_10c_TS-H_dimer_parsep_1_wb97xd.log

SCF (RwB97XD) = -3423.62127794
E(SCF)+ZPE(0 K)= -3423.123644
H(298 K)= -3423.078840
G(298 K)= -3423.201526
Lowest Frequency = -1234.7650cm-1

C	-4.843439	0.841914	2.474396
C	-5.303038	-0.189274	1.521013
H	-6.354995	-0.392824	1.745385
H	-5.098883	0.154816	0.076936
F	-3.546567	1.202904	2.283593
F	-5.572274	1.985580	2.285534
F	-4.930076	0.574687	3.798622
K	-3.929912	2.841239	-0.142981
N	-5.002103	0.415317	-1.137168
Si	-3.640689	-0.251316	-1.926951
Si	-6.592274	0.517899	-1.751504
C	-3.953617	-1.947280	-2.672269
C	-3.031125	0.863047	-3.318345
C	-2.238291	-0.416073	-0.685696
C	-7.517629	1.784156	-0.700672
C	-6.674637	1.056705	-3.547254
C	-7.532312	-1.101648	-1.604448
H	-4.717570	-1.901585	-3.454089
H	-4.299707	-2.649311	-1.908318
H	-3.045256	-2.359823	-3.121827
H	-2.126547	0.450307	-3.776147
H	-2.784248	1.872467	-2.972640
H	-3.782919	0.964754	-4.105334

H	-1.886871	0.545976	-0.299155
H	-1.375050	-0.898827	-1.153601
H	-2.537364	-1.025027	0.170692
H	-7.010183	2.751089	-0.765784
H	-7.526689	1.435313	0.336787
H	-8.550812	1.920883	-1.029647
H	-6.152751	2.000556	-3.722785
H	-7.716312	1.187911	-3.856548
H	-6.231285	0.303105	-4.204401
H	-8.577368	-0.993769	-1.910833
H	-7.518740	-1.466795	-0.573395
H	-7.078119	-1.873822	-2.231873
K	-7.526809	4.029330	1.665728
N	-5.325358	5.159042	0.547812
Si	-4.459334	5.763775	1.852716
Si	-5.814251	5.786912	-0.929907
C	-4.077134	7.610186	1.836753
C	-5.353235	5.471699	3.503236
C	-2.783836	4.907389	2.088495
C	-7.703723	5.784771	-1.116932
C	-5.282153	7.550069	-1.332725
C	-5.193515	4.749706	-2.392326
H	-4.989778	8.210066	1.781706
H	-3.445108	7.890544	0.990495
H	-3.549241	7.893774	2.752785
H	-4.751694	5.872356	4.324785
H	-5.500581	4.409851	3.730229
H	-6.320491	5.985739	3.554435
H	-2.152713	4.984204	1.196035
H	-2.881405	3.850910	2.360593
H	-2.229695	5.378998	2.905798
H	-8.189432	6.367253	-0.325739
H	-8.135971	4.777767	-1.125140
H	-7.993026	6.244250	-2.067012
H	-5.677473	8.273312	-0.615101
H	-5.645183	7.834675	-2.325463
H	-4.193272	7.649675	-1.338718
H	-5.575074	5.153559	-3.334953
H	-5.529067	3.708140	-2.351444
H	-4.100148	4.761219	-2.469225
F	-4.585014	-1.377275	1.823791

ch2f-cf3_11a_TS-H_dimer_parsep_2_b3pw91.log

SCF (RB3PW91) = -3423.45341904
E(SCF)+ZPE(0 K)= -3422.959326
H(298 K)= -3422.914008
G(298 K)= -3423.037714
Lowest Frequency = -1239.9609cm-1

C	-4.311693	0.945644	2.401547
C	-5.383769	0.249014	1.671392
H	-5.453726	-0.779028	2.031818
H	-5.164453	0.371806	0.199323
F	-3.124824	0.352546	2.204352
F	-4.178842	2.248915	1.963091
F	-4.455097	1.083649	3.752857
K	-3.984211	3.097393	-0.868403
N	-4.970286	0.478409	-1.043502
Si	-3.617032	-0.477012	-1.485865
Si	-6.469184	0.533425	-1.866210
C	-3.587425	-0.966997	-3.298919
C	-2.011012	0.467978	-1.180088
C	-3.540466	-2.055163	-0.469263
C	-7.592376	1.733044	-0.945579
C	-6.320988	1.151585	-3.636872
C	-7.356858	-1.122346	-1.913977
H	-3.555261	-0.093378	-3.955039
H	-4.455640	-1.569215	-3.579131
H	-2.692742	-1.565560	-3.501031
H	-1.153218	-0.199233	-1.312621
H	-1.943409	0.866085	-0.163173
H	-1.870380	1.288303	-1.893551
H	-3.414197	-1.843413	0.595401

```
H       -2.705566   -2.689839   -0.782890
H       -4.463771   -2.630759   -0.586826
H       -7.112661    2.709340   -0.836942
H       -7.838634    1.338547    0.042676
H       -8.528153    1.898506   -1.485915
H       -5.679822    2.033740   -3.720278
H       -7.307020    1.425755   -4.026043
H       -5.910268    0.382733   -4.295109
H       -8.344283   -1.039323   -2.379852
H       -7.494542   -1.512594   -0.900758
H       -6.784049   -1.863137   -2.479714
K       -6.888563    3.582067    1.861513
N       -5.322797    5.168581    0.322811
Si       -4.206207    5.826830    1.394482
Si       -6.334775    5.838801   -0.839992
C       -3.972228    7.697224    1.350443
C       -4.635067    5.446665    3.204397
C       -2.462985    5.119205    1.146994
C       -8.169662    5.586167   -0.421459
C       -6.172781    7.688448   -1.168347
C       -6.091619    5.033424   -2.538243
H       -4.898557    8.228740    1.583966
H       -3.625874    8.043774    0.373493
H       -3.223743    7.995031    2.092487
H       -3.907780    5.922642    3.870152
H       -4.608050    4.378985    3.445550
H       -5.617154    5.848884    3.480546
H       -2.088500    5.312328    0.134824
H       -2.395749    4.045311    1.349086
H       -1.760561    5.599632    1.835860
H       -8.421995    6.039201    0.544398
H       -8.476992    4.535013   -0.404874
H       -8.802435    6.071692   -1.171700
H       -6.404677    8.285708   -0.283023
H       -6.867873    7.986305   -1.960701
H       -5.164464    7.954916   -1.496449
H       -6.770779    5.477364   -3.273357
H       -6.299913    3.959912   -2.544875
H       -5.074689    5.192707   -2.915478
F       -6.603073    0.902929    2.071173
```

ch2f-cf3_11b_TS-H_dimer_parsep_2_pbe0.log

```
SCF (RPBE1PBE) =      -3421.94485198
E(SCF)+ZPE(0 K)=      -3421.449616
H(298 K)=             -3421.404527
G(298 K)=             -3421.526841
Lowest Frequency = -1191.5546cm-1
```

```
C       -4.660634    0.916992    2.535875
C       -5.573630    0.159418    1.663644
H       -5.698012   -0.845686    2.074679
H       -5.216934    0.233414    0.215470
F       -3.474523    0.318937    2.657726
F       -4.417738    2.175336    2.026852
F       -5.095706    1.178102    3.801050
K       -3.997365    3.024136   -0.736622
N       -5.004650    0.369736   -1.011884
Si       -3.608412   -0.449475   -1.582035
Si       -6.441858    0.534464   -1.932730
C       -3.972726   -1.939077   -2.670190
C       -2.488931    0.696593   -2.587939
C       -2.573253   -1.057952   -0.141374
C       -7.557511    1.831615   -1.142654
C       -6.097966    1.102055   -3.693836
C       -7.464577   -1.038404   -2.026454
H       -4.510092   -1.669540   -3.583808
H       -4.575152   -2.676926   -2.131559
H       -3.038718   -2.426839   -2.969423
H       -1.719344    0.111933   -3.103470
H       -1.945144    1.414396   -1.961539
H       -3.039665    1.249556   -3.355572
H       -2.212961   -0.234545    0.481038
H       -1.695336   -1.593298   -0.518379
```

```
H       -3.131031   -1.745496    0.500973
H       -7.022156    2.765352   -0.948203
H       -7.975881    1.452753   -0.206883
H       -8.390908    2.079687   -1.806783
H       -5.568490    2.059466   -3.728507
H       -7.039542    1.232586   -4.238332
H       -5.498932    0.372264   -4.246265
H       -8.430276   -0.853872   -2.509354
H       -7.661714   -1.426630   -1.021726
H       -6.952532   -1.822086   -2.591650
K       -7.034086    3.542040    1.811081
N       -5.328166    5.129855    0.416296
Si       -4.256681    5.711929    1.575643
Si       -6.216512    5.869929   -0.804930
C       -3.936766    7.569988    1.602418
C       -4.822194    5.307113    3.341861
C       -2.532279    4.934980    1.418931
C       -8.084832    5.652093   -0.555580
C       -5.987113    7.726108   -1.045524
C       -5.835320    5.128008   -2.508312
H       -4.851730    8.140299    1.786378
H       -3.504486    7.926914    0.663540
H       -3.230211    7.810496    2.404542
H       -4.124835    5.738580    4.067993
H       -4.853229    4.233670    3.557623
H       -5.805370    5.741943    3.560645
H       -2.089482    5.132544    0.434958
H       -2.521032    3.854764    1.600811
H       -1.853112    5.371919    2.158934
H       -8.406565    6.085485    0.399197
H       -8.417401    4.608829   -0.594393
H       -8.640063    6.175205   -1.341841
H       -6.280701    8.296186   -0.159752
H       -6.607951    8.068999   -1.880722
H       -4.949673    7.982427   -1.278678
H       -6.431579    5.623201   -3.282415
H       -6.065973    4.060580   -2.587210
H       -4.783218    5.277757   -2.780326
F       -6.842538    0.817108    1.807277
```

ch2f-cf3_11c_TS-H_dimer_parsep_2_wb97xd.log

```
SCF (RwB97XD) =       -3423.62659731
E(SCF)+ZPE(0 K)=      -3423.128191
H(298 K)=             -3423.083786
G(298 K)=             -3423.204250
Lowest Frequency = -1301.4014cm-1
```

```
C       -4.280614    0.956266    2.381120
C       -5.366657    0.266459    1.651854
H       -5.442338   -0.755025    2.029914
H       -5.160066    0.361046    0.184393
F       -3.108759    0.337382    2.198084
F       -4.116634    2.245658    1.924729
F       -4.434249    1.112424    3.720737
K       -3.986339    3.087106   -0.850229
N       -4.971321    0.435444   -1.061796
Si       -3.623491   -0.531789   -1.485525
Si       -6.465053    0.526298   -1.885382
C       -3.598985   -1.056775   -3.289328
C       -2.015384    0.412380   -1.192739
C       -3.558395   -2.089983   -0.438015
C       -7.570026    1.735179   -0.953163
C       -6.296363    1.162191   -3.648295
C       -7.384177   -1.110842   -1.961260
H       -3.552191   -0.196297   -3.961710
H       -4.478849   -1.647264   -3.558624
H       -2.716180   -1.674783   -3.481222
H       -1.156079   -0.254065   -1.313921
H       -1.952170    0.825711   -0.181542
H       -1.878023    1.224189   -1.915918
H       -3.453567   -1.855650    0.624229
H       -2.716421   -2.728104   -0.722439
H       -4.477586   -2.670908   -0.560495
```

H	-7.067225	2.696416	-0.814896
H	-7.838810	1.328113	0.024185
H	-8.493926	1.936719	-1.501289
H	-5.652469	2.044049	-3.715298
H	-7.275995	1.441407	-4.048521
H	-5.877867	0.398692	-4.308322
H	-8.363664	-1.003431	-2.437438
H	-7.541768	-1.510064	-0.954779
H	-6.819072	-1.856150	-2.528602
K	-6.899086	3.571998	1.914630
N	-5.336854	5.199244	0.350585
Si	-4.214175	5.861370	1.410172
Si	-6.340741	5.853028	-0.825574
C	-4.029395	7.738993	1.401394
C	-4.590393	5.436785	3.221908
C	-2.457137	5.205266	1.118418
C	-8.178343	5.580485	-0.434483
C	-6.193597	7.704295	-1.158218
C	-6.076380	5.048308	-2.522936
H	-4.968439	8.240657	1.649766
H	-3.698713	8.115614	0.430324
H	-3.285638	8.042616	2.145035
H	-3.849478	5.904752	3.877378
H	-4.547664	4.364058	3.436877
H	-5.568077	5.821294	3.534990
H	-2.115592	5.405772	0.096498
H	-2.354468	4.133568	1.318501
H	-1.751273	5.705671	1.788503
H	-8.451977	6.030419	0.526483
H	-8.469242	4.524619	-0.418950
H	-8.805597	6.055203	-1.195470
H	-6.429596	8.302175	-0.274479
H	-6.890194	7.995226	-1.951060
H	-5.187531	7.979017	-1.486221
H	-6.744256	5.496154	-3.265306
H	-6.289533	3.975019	-2.533238
H	-5.054435	5.202627	-2.887738
F	-6.576878	0.931681	2.062298

ch2f-cf3_12a_TS-H_dimer_sep_1_b3pw91.log

SCF (RB3PW91) = -3423.42831956
E(SCF)+ZPE(0 K)= -3422.935424
H(298 K)= -3422.889361
G(298 K)= -3423.017175
Lowest Frequency = -1182.9341cm-1

C	-5.291105	3.072985	-2.075442
C	-5.999216	1.821045	-1.747671
H	-6.919771	1.794715	-2.336223
H	-5.281953	0.531007	-1.903353
F	-4.185489	3.272960	-1.301453
F	-4.852020	3.030574	-3.367402
F	-5.985769	4.239631	-1.969281
K	-3.445299	0.723738	-4.045240
N	-4.774201	-0.601852	-2.082344
Si	-3.570927	-1.098904	-0.967938
Si	-5.843209	-1.634102	-2.938833
C	-1.901701	-1.298205	-1.836422
C	-3.335610	0.228106	0.335036
C	-3.919579	-2.717303	-0.088838
C	-6.558128	-0.687368	-4.408938
C	-4.969920	-3.129464	-3.673356
C	-7.281647	-2.231643	-1.897399
H	-1.467270	-0.340577	-2.149344
H	-1.972006	-1.953086	-2.711063
H	-1.169712	-1.747835	-1.157698
H	-2.442854	0.025963	0.931609
H	-4.190947	0.249971	1.015836
H	-3.204485	1.204247	-0.140068
H	-4.863178	-2.671301	0.458686
H	-3.125001	-2.930587	0.633150
H	-3.969263	-3.562484	-0.779475
H	-5.835647	-0.544106	-5.221602

H	-6.954659	0.290877	-4.125607
H	-7.384530	-1.256765	-4.846142
H	-4.141298	-2.834311	-4.325256
H	-5.670855	-3.710898	-4.281765
H	-4.567942	-3.798412	-2.909018
H	-7.960458	-2.864433	-2.478201
H	-7.857193	-1.386288	-1.509602
H	-6.930465	-2.812104	-1.040303
K	-4.924385	2.800853	1.646598
N	-5.545673	1.014914	3.434628
Si	-7.012244	0.250901	3.176248
Si	-4.197802	0.526711	4.291864
C	-7.735145	-0.625043	4.692418
C	-8.328430	1.499877	2.632665
C	-6.951849	-1.057312	1.810759
C	-2.720976	1.655596	3.896981
C	-4.388481	0.619496	6.173482
C	-3.612351	-1.239292	3.937143
H	-7.860898	0.072451	5.526818
H	-7.076498	-1.430998	5.032093
H	-8.713877	-1.068618	4.477676
H	-9.304780	1.020673	2.501393
H	-8.076539	1.971700	1.677126
H	-8.447064	2.292962	3.378552
H	-6.576257	-0.629474	0.877180
H	-7.936568	-1.491081	1.605464
H	-6.281445	-1.873852	2.094778
H	-2.942851	2.702697	4.135618
H	-2.418716	1.595248	2.845627
H	-1.843057	1.377509	4.489545
H	-4.673279	1.629675	6.486344
H	-3.462450	0.355128	6.696294
H	-5.171314	-0.062754	6.519569
H	-2.678335	-1.476058	4.459090
H	-3.448414	-1.396031	2.867634
H	-4.363300	-1.966695	4.262671
F	-6.401675	1.943706	-0.381841

ch2f-cf3_12b_TS-H_dimer_sep_1_pbe0.log

SCF (RPBE1PBE) = -3421.92335469
E(SCF)+ZPE(0 K)= -3421.429952
H(298 K)= -3421.383758
G(298 K)= -3421.512398
Lowest Frequency = -1158.9616cm-1

C	-5.252737	3.078644	-2.035376
C	-5.960628	1.816403	-1.746013
H	-6.877900	1.809715	-2.342755
H	-5.248422	0.521013	-1.926806
F	-4.176842	3.274183	-1.228462
F	-4.778357	3.059911	-3.310907
F	-5.959421	4.232300	-1.932997
K	-3.517504	0.737029	-4.132160
N	-4.752504	-0.611440	-2.122127
Si	-3.517177	-1.101584	-1.038935
Si	-5.851110	-1.633776	-2.954808
C	-1.867629	-1.268298	-1.947737
C	-3.275318	0.211556	0.279550
C	-3.829637	-2.724755	-0.153682
C	-6.563167	-0.687143	-4.427203
C	-5.021758	-3.154436	-3.685730
C	-7.291113	-2.188624	-1.891957
H	-1.467667	-0.301629	-2.280207
H	-1.946247	-1.930843	-2.816639
H	-1.105051	-1.694586	-1.287181
H	-2.380604	-0.001762	0.871132
H	-4.131043	0.218020	0.961729
H	-3.147971	1.198789	-0.174437
H	-4.758389	-2.682183	0.421761
H	-3.013731	-2.930530	0.547397
H	-3.893997	-3.573753	-0.839184
H	-5.853758	-0.585641	-5.258368
H	-6.923011	0.309249	-4.154737

H	-7.417621	-1.235534	-4.837602
H	-4.186317	-2.882808	-4.340024
H	-5.740995	-3.716263	-4.291904
H	-4.637644	-3.833950	-2.920815
H	-7.991534	-2.809386	-2.460590
H	-7.842940	-1.327307	-1.502831
H	-6.944428	-2.771956	-1.033951
K	-4.918371	2.760194	1.667339
N	-5.569101	0.982020	3.458756
Si	-7.043376	0.229289	3.209667
Si	-4.220670	0.524812	4.332054
C	-7.800550	-0.579817	4.745584
C	-8.333025	1.474534	2.600321
C	-6.988753	-1.127187	1.890615
C	-2.755744	1.663126	3.920094
C	-4.417094	0.648569	6.210494
C	-3.606804	-1.238007	4.010355
H	-7.920029	0.147212	5.555993
H	-7.164293	-1.389356	5.119623
H	-8.786886	-1.008403	4.533650
H	-9.312764	1.002274	2.466158
H	-8.056429	1.909582	1.633365
H	-8.457209	2.294580	3.316011
H	-6.609967	-0.729004	0.944186
H	-7.977006	-1.559686	1.696737
H	-6.323441	-1.940813	2.198067
H	-2.990374	2.713721	4.131970
H	-2.442514	1.579775	2.872587
H	-1.879056	1.410178	4.526321
H	-4.707386	1.662647	6.506345
H	-3.490626	0.397314	6.739510
H	-5.197729	-0.031792	6.566992
H	-2.669511	-1.449309	4.537991
H	-3.437720	-1.412009	2.942987
H	-4.345599	-1.973033	4.348357
F	-6.371760	1.906645	-0.384116

ch2f-cf3_12c_TS-H_dimer_sep_1_wb97xd.log

SCF (RwB97XD) = -3423.60766357
E(SCF)+ZPE(0 K)= -3423.110020
H(298 K)= -3423.065462
G(298 K)= -3423.186845
Lowest Frequency = -1264.1159cm-1

C	-5.037162	2.657587	-3.018534
C	-5.438096	2.036826	-1.736731
H	-6.523333	2.124435	-1.651265
H	-4.990808	0.600806	-1.720902
F	-3.683673	2.602089	-3.224218
F	-5.587539	1.963009	-4.047597
F	-5.353779	3.953990	-3.226204
K	-3.462508	-0.009273	-4.287633
N	-4.667163	-0.604949	-1.802568
Si	-3.304615	-0.944221	-0.824607
Si	-6.015358	-1.539254	-2.296951
C	-1.693508	-0.892368	-1.822417
C	-3.114986	0.378416	0.492444
C	-3.302280	-2.610671	0.034041
C	-7.613551	-0.606562	-1.980378
C	-5.997712	-1.865655	-4.165504
C	-6.159013	-3.241429	-1.522544
H	-1.476905	0.101268	-2.232489
H	-1.664293	-1.633495	-2.629807
H	-0.852831	-1.130163	-1.163778
H	-2.216024	0.204045	1.089707
H	-3.976311	0.365519	1.167510
H	-3.027615	1.363444	0.026066
H	-4.166384	-2.735959	0.689299
H	-2.401773	-2.690346	0.650966
H	-3.290212	-3.442865	-0.673676
H	-7.695023	0.276951	-2.618848
H	-7.688575	-0.276769	-0.941933
H	-8.474394	-1.247145	-2.194368

H	-6.096369	-0.952663	-4.763780
H	-6.860536	-2.484776	-4.429117
H	-5.113346	-2.423757	-4.494591
H	-7.073047	-3.718852	-1.889696
H	-6.219258	-3.200530	-0.433848
H	-5.320160	-3.887497	-1.792011
K	-4.762191	2.951794	1.903095
N	-5.625266	0.911232	3.328082
Si	-7.019509	0.259727	2.685677
Si	-4.450219	0.304891	4.342953
C	-8.354595	-0.173007	3.955507
C	-7.821071	1.488754	1.482592
C	-6.749920	-1.310552	1.665795
C	-2.916539	1.424493	4.316405
C	-4.960587	0.202235	6.162490
C	-3.818258	-1.425252	3.901858
H	-8.610141	0.694670	4.571863
H	-8.005196	-0.961643	4.629884
H	-9.274513	-0.529267	3.479923
H	-8.777548	1.114358	1.104737
H	-7.187004	1.665424	0.606596
H	-8.020774	2.453190	1.962849
H	-6.036789	-1.113086	0.861019
H	-7.677426	-1.670724	1.209325
H	-6.343631	-2.120731	2.278550
H	-3.159074	2.448430	4.623462
H	-2.455981	1.460851	3.322595
H	-2.147477	1.062300	5.005913
H	-5.260126	1.184645	6.541404
H	-4.150587	-0.169425	6.798979
H	-5.816089	-0.469358	6.287026
H	-2.964674	-1.715505	4.523735
H	-3.504048	-1.476467	2.855378
H	-4.599945	-2.177490	4.046622
F	-4.890643	2.883007	-0.714892

ch2f-cf3_13a_TS-H_dimer_sep_2_b3pw91.log

SCF (RB3PW91) = -3423.42940909
E(SCF)+ZPE(0 K)= -3422.936866
H(298 K)= -3422.890751
G(298 K)= -3423.018245
Lowest Frequency = -1097.3156cm-1

C	-4.050629	2.532041	-0.601911
C	-4.877376	1.345150	-0.348128
H	-4.828353	1.104939	0.718407
H	-4.665278	0.134488	-1.166892
F	-2.772903	2.312298	-0.252396
F	-4.028197	2.902767	-1.929450
F	-4.426021	3.698465	0.032048
K	-3.155629	0.778007	-3.448428
N	-4.586058	-0.892816	-1.874810
Si	-3.495195	-2.058536	-1.247896
Si	-6.009205	-1.211051	-2.774377
C	-2.193969	-2.515820	-2.539819
C	-2.583953	-1.315135	0.211831
C	-4.295645	-3.662500	-0.691790
C	-6.476925	0.340106	-3.745270
C	-5.760090	-2.570046	-4.051048
C	-7.473782	-1.678929	-1.701859
H	-1.514742	-1.685428	-2.767851
H	-2.653310	-2.847932	-3.475539
H	-1.561218	-3.333125	-2.178242
H	-1.851984	-2.024882	0.609738
H	-3.273244	-1.061324	1.018939
H	-2.042819	-0.405249	-0.062965
H	-5.042826	-3.477313	0.083290
H	-3.541901	-4.338203	-0.274186
H	-4.785380	-4.185773	-1.517112
H	-5.840184	0.501167	-4.623686
H	-6.450152	1.237699	-3.121836
H	-7.496480	0.246423	-4.132432
H	-4.944607	-2.329050	-4.740129

H	-6.668399	-2.696498	-4.649843
H	-5.534120	-3.535537	-3.591658
H	-8.373867	-1.834049	-2.305660
H	-7.690246	-0.899300	-0.967443
H	-7.274437	-2.603877	-1.154192
K	-6.822741	3.174197	1.571322
N	-6.017692	1.208812	3.056850
Si	-6.968411	-0.146394	2.841582
Si	-4.707784	1.590604	4.015878
C	-7.589534	-0.945555	4.442048
C	-8.531426	0.303273	1.861193
C	-6.146205	-1.538791	1.864895
C	-4.299839	3.437895	3.854579
C	-4.961537	1.288440	5.867598
C	-3.104772	0.689196	3.570405
H	-8.087218	-0.210541	5.082710
H	-6.759287	-1.371781	5.014081
H	-8.301321	-1.754619	4.243209
H	-9.173613	-0.571600	1.716426
H	-8.294992	0.682781	0.861234
H	-9.131566	1.056397	2.385619
H	-5.822322	-1.190781	0.881646
H	-6.826674	-2.383338	1.713975
H	-5.263753	-1.913449	2.392701
H	-5.143874	4.068050	4.159292
H	-4.020156	3.705991	2.829591
H	-3.453120	3.714539	4.491619
H	-5.868507	1.787049	6.224823
H	-4.118667	1.659604	6.461255
H	-5.069691	0.220339	6.080037
H	-2.272732	0.995234	4.214684
H	-2.813408	0.889241	2.535189
H	-3.222935	-0.393842	3.675960
F	-6.231271	1.745068	-0.613860

ch2f-cf3_13b_TS-H_dimer_sep_2_pbe0.log

SCF (RPBE1PBE) = -3421.92508074
E(SCF)+ZPE(0 K)= -3421.431848
H(298 K)= -3421.385644
G(298 K)= -3421.514217
Lowest Frequency = -1123.8819cm-1

C	-4.035599	2.519545	-0.590126
C	-4.878576	1.336172	-0.365387
H	-4.832903	1.078325	0.698396
H	-4.665247	0.132192	-1.198437
F	-2.768271	2.281518	-0.231487
F	-3.995098	2.909849	-1.906076
F	-4.408818	3.670504	0.059293
K	-3.188297	0.821217	-3.488577
N	-4.578463	-0.890688	-1.915635
Si	-3.478548	-2.052957	-1.295126
Si	-6.005513	-1.204749	-2.812389
C	-2.175340	-2.493528	-2.589303
C	-2.571254	-1.319459	0.172973
C	-4.266206	-3.664477	-0.741081
C	-6.470160	0.351150	-3.778099
C	-5.774798	-2.563360	-4.092303
C	-7.470632	-1.664331	-1.735954
H	-1.516914	-1.648973	-2.828378
H	-2.632162	-2.843463	-3.520494
H	-1.521955	-3.293639	-2.224674
H	-1.826854	-2.027272	0.552264
H	-3.259757	-1.092936	0.990617
H	-2.044009	-0.395882	-0.085252
H	-5.002344	-3.487865	0.047982
H	-3.503278	-4.339931	-0.338953
H	-4.767091	-4.185543	-1.561860
H	-5.839324	0.511028	-4.661747
H	-6.437924	1.248846	-3.153134
H	-7.492872	0.262709	-4.159423
H	-4.963034	-2.328536	-4.788653
H	-6.689595	-2.681098	-4.683702

H	-5.553711	-3.532241	-3.636117
H	-8.372053	-1.818336	-2.338680
H	-7.684832	-0.881314	-1.002938
H	-7.274450	-2.588938	-1.184940
K	-6.782618	3.150084	1.600866
N	-6.007123	1.176580	3.101477
Si	-6.992140	-0.154797	2.892093
Si	-4.695947	1.570243	4.054007
C	-7.657065	-0.917916	4.491699
C	-8.527603	0.327036	1.883737
C	-6.198952	-1.581136	1.938427
C	-4.300583	3.419085	3.877959
C	-4.937970	1.276895	5.908087
C	-3.086359	0.680740	3.607604
H	-8.149474	-0.164038	5.115046
H	-6.846105	-1.355002	5.083855
H	-8.383306	-1.713905	4.290779
H	-9.192864	-0.531305	1.740347
H	-8.266124	0.686364	0.881530
H	-9.114764	1.103388	2.389621
H	-5.845924	-1.246448	0.959391
H	-6.903928	-2.404323	1.777065
H	-5.337914	-1.983194	2.482963
H	-5.148525	4.047122	4.177837
H	-4.021097	3.681132	2.850354
H	-3.455211	3.706929	4.512509
H	-5.844023	1.774830	6.269715
H	-4.091785	1.652687	6.494607
H	-5.042005	0.208897	6.126362
H	-2.256374	0.996275	4.250474
H	-2.797079	0.880564	2.570748
H	-3.193952	-0.403785	3.715837
F	-6.221477	1.760656	-0.623644

ch2f-cf3_13c_TS-H_dimer_sep_2_wb97xd.log

SCF (RwB97XD) = -3423.60821337
E(SCF)+ZPE(0 K)= -3423.112062
H(298 K)= -3423.066592
G(298 K)= -3423.191409
Lowest Frequency = -1295.1833cm-1

C	-4.057692	2.527731	-0.628846
C	-4.865717	1.327408	-0.333446
H	-4.769861	1.105001	0.733332
H	-4.645899	0.138165	-1.162803
F	-2.762277	2.307153	-0.359841
F	-4.115125	2.898687	-1.947808
F	-4.397703	3.675291	0.036131
K	-3.079920	0.804549	-3.468586
N	-4.543217	-0.890849	-1.889710
Si	-3.475484	-2.060620	-1.237514
Si	-5.983111	-1.204117	-2.759604
C	-2.190560	-2.586409	-2.518017
C	-2.556151	-1.300800	0.209579
C	-4.303737	-3.636172	-0.637429
C	-6.460541	0.348648	-3.720923
C	-5.770504	-2.571638	-4.034017
C	-7.426779	-1.661141	-1.651926
H	-1.504843	-1.776037	-2.790671
H	-2.667817	-2.948980	-3.433376
H	-1.566678	-3.397903	-2.130603
H	-1.832082	-2.007755	0.625250
H	-3.249270	-1.027245	1.007792
H	-2.008173	-0.398871	-0.077639
H	-5.042428	-3.420040	0.137936
H	-3.560757	-4.316710	-0.209820
H	-4.809377	-4.169017	-1.447097
H	-5.832885	0.505301	-4.606044
H	-6.412147	1.243775	-3.095136
H	-7.486621	0.267433	-4.091664
H	-4.963678	-2.340390	-4.736264
H	-6.688557	-2.694933	-4.617277
H	-5.544181	-3.536148	-3.572381

H	-8.339622	-1.823665	-2.233339
H	-7.627883	-0.873590	-0.920990
H	-7.214264	-2.579276	-1.097376
K	-6.870664	3.245685	1.574228
N	-6.099971	1.257501	3.102937
Si	-7.006722	-0.116631	2.837921
Si	-4.743520	1.596575	4.008664
C	-7.625355	-0.971802	4.410603
C	-8.565199	0.302524	1.839118
C	-6.130329	-1.468518	1.847264
C	-4.330132	3.445915	3.902903
C	-4.904301	1.210691	5.855274
C	-3.165525	0.712450	3.448627
H	-8.156497	-0.268873	5.059961
H	-6.789266	-1.381518	4.986057
H	-8.305713	-1.799751	4.184517
H	-9.182521	-0.586472	1.675902
H	-8.320018	0.698080	0.847690
H	-9.190723	1.036978	2.359214
H	-5.804426	-1.098981	0.871913
H	-6.785780	-2.328410	1.674599
H	-5.243701	-1.829447	2.378222
H	-5.164114	4.067700	4.247404
H	-4.076452	3.746088	2.879928
H	-3.465779	3.697720	4.525499
H	-5.782629	1.702361	6.285198
H	-4.025835	1.539412	6.420591
H	-5.019209	0.134873	6.020216
H	-2.306599	0.984538	4.071634
H	-2.915180	0.964233	2.413661
H	-3.281321	-0.374444	3.503366
F	-6.231149	1.721885	-0.539036

ch2f-cf3_14a_kchfcf3_khmds_1_b3pw91.log

SCF (RB3PW91) = -2549.45524046
E(SCF)+ZPE(0 K)= -2549.196919
H(298 K)= -2549.168694
G(298 K)= -2549.257425
Lowest Frequency = 15.2934cm-1

N	-3.479193	1.058662	-6.308523
K	-3.764289	-1.101371	-4.735103
Si	-1.873068	1.532022	-6.238891
Si	-4.660341	0.969817	-7.493413
C	-0.959308	1.654666	-7.882928
C	-1.639796	3.221812	-5.402634
C	-0.856308	0.343560	-5.166989
C	-4.061909	0.947361	-9.280516
C	-5.720742	-0.589260	-7.284705
C	-5.916832	2.392338	-7.393940
H	0.094314	1.905891	-7.721197
H	-1.389233	2.428588	-8.524835
H	-0.995359	0.709574	-8.432152
H	-0.597580	3.548324	-5.481533
H	-1.864271	3.201368	-4.329566
H	-2.252386	3.998384	-5.874517
H	-0.836516	-0.672054	-5.579380
H	-1.222568	0.297503	-4.134754
H	0.185189	0.675571	-5.105259
H	-3.355635	0.130217	-9.453434
H	-3.561806	1.881357	-9.550647
H	-4.903401	0.810920	-9.968034
H	-5.139852	-1.509596	-7.418091
H	-6.518609	-0.614329	-8.034038
H	-6.217507	-0.624342	-6.307577
H	-6.583918	2.377000	-8.262214
H	-5.424279	3.371162	-7.388163
H	-6.571446	2.325045	-6.516338
K	-4.670791	2.591914	-4.374917
C	-5.182819	0.343039	-2.560861
C	-4.047263	0.516507	-1.651622
H	-5.773468	-0.499729	-2.187601
F	-3.277629	1.611924	-1.963679
F	-3.228267	-0.567819	-1.736071
F	-4.280601	0.680392	-0.303988
F	-6.025839	1.515475	-2.364135

ch2f-cf3_14b_kchfcf3_khmds_1_pbe0.log

SCF (RPBE1PBE) = -2548.43039214
E(SCF)+ZPE(0 K)= -2548.171350
H(298 K)= -2548.143216
G(298 K)= -2548.231212
Lowest Frequency = 19.9692cm-1

N	-3.507961	1.116874	-6.308652
K	-3.885198	-1.007880	-4.680555
Si	-1.872388	1.469458	-6.221150
Si	-4.675110	0.974503	-7.501204
C	-0.934608	1.544059	-7.854453
C	-1.534702	3.126562	-5.359441
C	-0.941353	0.202098	-5.159087
C	-4.115583	1.278664	-9.274859
C	-5.484761	-0.742635	-7.502623
C	-6.133589	2.158099	-7.221166
H	0.127936	1.745433	-7.679432
H	-1.318519	2.333716	-8.506882
H	-1.007016	0.598000	-8.399915
H	-0.467179	3.369605	-5.395752
H	-1.802176	3.114546	-4.295610
H	-2.065442	3.953839	-5.845213
H	-1.001368	-0.811790	-5.573511
H	-1.294682	0.181633	-4.120729
H	0.123449	0.454120	-5.109667
H	-3.335038	0.574981	-9.578746
H	-3.717575	2.290415	-9.399719
H	-4.954914	1.164501	-9.969544
H	-4.749605	-1.544389	-7.640893
H	-6.203014	-0.828655	-8.325202
H	-6.054301	-0.941878	-6.585974
H	-6.862355	2.063338	-8.033430
H	-5.819671	3.208879	-7.205375
H	-6.684538	1.939858	-6.297625
K	-4.654222	2.738156	-4.441632
C	-5.216063	0.571612	-2.545813
C	-4.030657	0.716232	-1.696107
H	-5.830115	-0.223942	-2.109824
F	-3.229621	1.758813	-2.079702
F	-3.270666	-0.405779	-1.784829
F	-4.191773	0.929583	-0.352040
F	-5.978946	1.791262	-2.355890

ch2f-cf3_14c_kchfcf3_khmds_1_wb97xd.log

SCF (RwB97XD) = -2549.60834977
E(SCF)+ZPE(0 K)= -2549.347522
H(298 K)= -2549.319891
G(298 K)= -2549.406616
Lowest Frequency = 12.4437cm-1

N	-3.540028	1.102243	-6.314453
K	-3.946655	-1.045999	-4.667223
Si	-1.908428	1.466101	-6.221301
Si	-4.679620	0.984956	-7.534275
C	-0.956749	1.501980	-7.849528
C	-1.579099	3.148129	-5.404408
C	-0.975043	0.234572	-5.120344
C	-4.072725	1.289428	-9.293729
C	-5.515373	-0.719114	-7.569571
C	-6.127482	2.189429	-7.290096
H	0.103779	1.704879	-7.669606
H	-1.332813	2.276437	-8.523111
H	-1.026428	0.544893	-8.374295
H	-0.516408	3.403679	-5.461504
H	-1.832539	3.157731	-4.338063
H	-2.129202	3.955219	-5.900469
H	-1.012721	-0.787612	-5.514428

```
H        -1.347876    0.228651   -4.089606
H         0.083528    0.505284   -5.058270
H        -3.288395    0.583556   -9.579549
H        -3.667019    2.298625   -9.407781
H        -4.894838    1.180490  -10.008203
H        -4.789320   -1.529941   -7.695440
H        -6.215994   -0.788214   -8.407510
H        -6.105252   -0.915643   -6.666339
H        -6.833814    2.112846   -8.122554
H        -5.794998    3.233059   -7.255016
H        -6.705838    1.974543   -6.383620
K        -4.710004    2.712739   -4.395075
C        -5.160140    0.535102   -2.450982
C        -3.954091    0.672522   -1.616047
H        -5.771642   -0.249424   -1.994198
F        -3.142067    1.697023   -2.019744
F        -3.212059   -0.461531   -1.703603
F        -4.102085    0.899387   -0.274368
F        -5.913889    1.765867   -2.237938
```

ch2f-cf3_15a_kchfcf3_khmds_2_b3pw91.log

```
SCF (RB3PW91) =       -2549.45526648
E(SCF)+ZPE(0 K)=      -2549.196830
H(298 K)=             -2549.168658
G(298 K)=             -2549.256996
Lowest Frequency = 17.0903cm-1

N        -3.276309    1.160915   -6.226970
K        -3.016079   -0.805661   -4.401613
Si        -1.756073    1.844028   -6.392222
Si        -4.526950    0.710493   -7.246712
C        -1.087664    2.010118   -8.146488
C        -1.654198    3.587434   -5.644371
C        -0.444477    0.864451   -5.431461
C        -4.137161    0.661697   -9.090186
C        -5.182706   -1.017053   -6.816749
C        -6.047175    1.835676   -7.076244
H        -0.077692    2.433436   -8.135170
H        -1.715470    2.667314   -8.754632
H        -1.035901    1.040803   -8.650081
H        -0.673813    4.032289   -5.844158
H        -1.764993    3.589202   -4.553252
H        -2.401237    4.264280   -6.074640
H        -0.359261   -0.169916   -5.784795
H        -0.639327    0.854455   -4.352151
H         0.543568    1.319512   -5.555418
H        -3.304501   -0.014396   -9.304314
H        -3.869246    1.650381   -9.472575
H        -5.005096    0.310301   -9.658156
H        -4.418189   -1.792961   -6.941840
H        -6.012268   -1.287873   -7.478021
H        -5.578811   -1.073061   -5.796040
H        -6.801189    1.579421   -7.827775
H        -5.792379    2.890586   -7.228955
H        -6.546572    1.732181   -6.105690
K        -4.493527    2.716627   -4.338178
C        -4.199534    0.761147   -2.171768
C        -5.539260    0.169468   -2.207522
F        -5.445569   -1.145191   -2.550551
F        -6.357074    0.758714   -3.141754
F        -6.314425    0.184053   -1.069069
H        -3.653772    0.283216   -1.351917
F        -4.390927    2.142107   -1.748086
```

ch2f-cf3_15b_kchfcf3_khmds_2_pbe0.log

```
SCF (RPBE1PBE) =      -2548.43043014
E(SCF)+ZPE(0 K)=      -2548.171238
H(298 K)=             -2548.143195
G(298 K)=             -2548.230367
Lowest Frequency = 24.6850cm-1

N        -3.267958    1.174398   -6.223890
K        -3.000863   -0.784840   -4.386135
Si        -1.741908    1.842679   -6.393537
Si        -4.528949    0.708168   -7.223668
C        -1.079134    2.012581   -8.149450
C        -1.621133    3.580714   -5.636614
C        -0.433872    0.847353   -5.444253
C        -4.173936    0.683442   -9.074627
C        -5.147096   -1.034288   -6.798023
C        -6.066348    1.801828   -7.013242
H        -0.066595    2.430702   -8.139703
H        -1.705485    2.675823   -8.753573
H        -1.033724    1.044844   -8.657954
H        -0.635536    4.015891   -5.834480
H        -1.731250    3.578658   -4.544824
H        -2.360349    4.269074   -6.063516
H        -0.363131   -0.188319   -5.798356
H        -0.617667    0.839464   -4.362404
H         0.558586    1.291215   -5.577390
H        -3.339532    0.017026   -9.313661
H        -3.921735    1.679289   -9.450988
H        -5.049995    0.331142   -9.630107
H        -4.367547   -1.794748   -6.928497
H        -5.973335   -1.319162   -7.458330
H        -5.540427   -1.102878   -5.776209
H        -6.835468    1.525840   -7.742840
H        -5.841329    2.862376   -7.176981
H        -6.535603    1.689727   -6.028055
K        -4.460155    2.757459   -4.344481
C        -4.225526    0.796170   -2.183471
C        -5.555255    0.188299   -2.285145
F        -5.430193   -1.117715   -2.636601
F        -6.332773    0.776168   -3.246695
F        -6.374354    0.182640   -1.186552
H        -3.716972    0.320426   -1.337886
F        -4.458712    2.166747   -1.767386
```

ch2f-cf3_15c_kchfcf3_khmds_2_wb97xd.log

```
SCF (RwB97XD) =       -2549.60835259
E(SCF)+ZPE(0 K)=      -2549.347680
H(298 K)=             -2549.319996
G(298 K)=             -2549.406559
Lowest Frequency = 21.7022cm-1

N        -3.245037    1.183120   -6.210841
K        -2.971314   -0.786366   -4.332453
Si        -1.727484    1.857490   -6.415260
Si        -4.505338    0.714474   -7.208068
C        -1.115522    2.046402   -8.189051
C        -1.587360    3.591074   -5.650947
C        -0.384767    0.862998   -5.514424
C        -4.136182    0.639594   -9.056232
C        -5.156998   -1.008099   -6.751906
C        -6.029860    1.834482   -7.041816
H        -0.103309    2.462814   -8.203907
H        -1.757647    2.717644   -8.765974
H        -1.087513    1.086242   -8.711249
H        -0.608995    4.028587   -5.872846
H        -1.669412    3.580448   -4.557529
H        -2.339458    4.280091   -6.051281
H        -0.329577   -0.172398   -5.868964
H        -0.530913    0.853883   -4.427687
H         0.601529    1.306353   -5.682638
H        -3.303583   -0.035889   -9.271720
H        -3.876759    1.622753   -9.457819
H        -5.009511    0.276538   -9.607366
H        -4.393466   -1.785489   -6.870387
H        -5.990348   -1.286990   -7.404140
H        -5.546038   -1.049874   -5.727884
H        -6.784451    1.568931   -7.788773
H        -5.781713    2.889609   -7.201087
H        -6.524089    1.735102   -6.068544
K        -4.454294    2.763100   -4.290909
C        -4.295554    0.748403   -2.141856
```

C -5.630680 0.135344 -2.250014
F -5.501054 -1.177383 -2.572925
F -6.396651 0.700355 -3.232875
F -6.456162 0.154637 -1.158412
H -3.803651 0.283796 -1.281563
F -4.542096 2.126415 -1.730177

ch2f-cf3_16a_TS-bF_khmds_1_b3pw91.log

SCF (RB3PW91) = -2549.44515709
E(SCF)+ZPE(0 K)= -2549.188320
H(298 K)= -2549.160180
G(298 K)= -2549.247219
Lowest Frequency = -432.6790cm-1

N -3.620906 1.097599 -6.303496
K -4.042949 -1.111829 -4.775267
Si -1.971065 1.386159 -6.239510
Si -4.785812 1.035705 -7.506390
C -1.053853 1.421026 -7.886927
C -1.560245 3.031183 -5.387590
C -1.075407 0.080604 -5.194299
C -4.237328 1.527871 -9.241978
C -5.553074 -0.691691 -7.678930
C -6.269551 2.154284 -7.115173
H 0.021359 1.552773 -7.725077
H -1.395354 2.239708 -8.525761
H -1.193725 0.486901 -8.439062
H -0.486915 3.241258 -5.442274
H -1.812852 3.026361 -4.321076
H -2.075877 3.871773 -5.865441
H -1.146166 -0.921228 -5.634550
H -1.451873 0.042462 -4.165596
H -0.007925 0.313038 -5.122069
H -3.463486 0.859405 -9.628445
H -3.836442 2.545677 -9.257825
H -5.083029 1.493983 -9.937067
H -4.792376 -1.458565 -7.864028
H -6.248802 -0.723417 -8.523933
H -6.136761 -0.981844 -6.797211
H -7.015427 2.089917 -7.914069
H -5.986709 3.211038 -7.039901
H -6.786077 1.857730 -6.194704
C -3.919612 0.253631 -2.073668
C -5.296058 0.385852 -2.067162
F -5.982478 -0.604477 -1.509197
F -5.941198 0.342454 -3.579268
F -5.865931 1.582733 -1.756957
H -3.432045 -0.370964 -1.340052
K -4.712313 2.650422 -4.368458
F -3.256915 1.479774 -2.293737

ch2f-cf3_16b_TS-bF_khmds_1_pbe0.log

SCF (RPBE1PBE) = -2548.41891902
E(SCF)+ZPE(0 K)= -2548.161564
H(298 K)= -2548.133430
G(298 K)= -2548.220571
Lowest Frequency = -449.1280cm-1

N -3.615698 1.098583 -6.312569
K -4.030626 -1.113496 -4.781538
Si -1.967923 1.394007 -6.233952
Si -4.788645 1.023397 -7.506581
C -1.047024 1.503637 -7.875903
C -1.569307 3.005315 -5.315014
C -1.064329 0.054741 -5.239262
C -4.245079 1.463689 -9.257320
C -5.580169 -0.696915 -7.632179
C -6.256957 2.168229 -7.133680
H 0.023932 1.659701 -7.705398
H -1.408660 2.332852 -8.490860
H -1.158803 0.583987 -8.458717
H -0.494277 3.213862 -5.345117
H -1.838340 2.960614 -4.252863
H -2.073621 3.866612 -5.768686
H -1.149851 -0.934514 -5.705582
H -1.422829 -0.009213 -4.204614
H 0.006271 0.278044 -5.178236
H -3.470440 0.784424 -9.624934
H -3.845631 2.481433 -9.307166
H -5.092657 1.406275 -9.949094
H -4.833959 -1.479634 -7.813093
H -6.287689 -0.735268 -8.467620
H -6.157207 -0.960505 -6.737153
H -7.000538 2.106362 -7.935553
H -5.959283 3.221988 -7.067770
H -6.783250 1.888540 -6.212601
C -3.918188 0.264786 -2.078655
C -5.288787 0.413347 -2.092378
F -5.998554 -0.541161 -1.512603
F -5.926726 0.326976 -3.609134
F -5.837679 1.628295 -1.837539
H -3.434600 -0.373435 -1.354749
K -4.724714 2.648247 -4.384569
F -3.233340 1.463505 -2.333469

ch2f-cf3_16c_TS-bF_khmds_1_wb97xd.log

SCF (RwB97XD) = -2549.59671573
E(SCF)+ZPE(0 K)= -2549.337591
H(298 K)= -2549.309956
G(298 K)= -2549.396430
Lowest Frequency = -472.5930cm-1

N -3.639877 1.088218 -6.322362
K -4.087626 -1.142562 -4.775632
Si -1.997074 1.396283 -6.229438
Si -4.790714 1.033707 -7.535698
C -1.058083 1.471935 -7.864565
C -1.611311 3.032740 -5.348383
C -1.094603 0.090417 -5.190974
C -4.199509 1.433037 -9.282249
C -5.631559 -0.663729 -7.657534
C -6.234121 2.225389 -7.214418
H 0.010140 1.631804 -7.686287
H -1.413408 2.287170 -8.500302
H -1.163765 0.541430 -8.429700
H -0.540677 3.255418 -5.394379
H -1.869137 3.005881 -4.283684
H -2.134116 3.876001 -5.812843
H -1.152727 -0.908046 -5.639670
H -1.482553 0.039082 -4.166918
H -0.030904 0.333490 -5.106246
H -3.429548 0.734278 -9.619960
H -3.779900 2.441352 -9.341332
H -5.031569 1.378769 -9.991494
H -4.907549 -1.469870 -7.820271
H -6.329484 -0.689290 -8.500188
H -6.224334 -0.900563 -6.766229
H -6.952630 2.186813 -8.039019
H -5.902375 3.267096 -7.136503
H -6.797524 1.965927 -6.310426
C -3.849237 0.244197 -2.078735
C -5.227456 0.337955 -2.013306
F -5.864265 -0.685000 -1.467276
F -5.922346 0.314961 -3.513404
F -5.821060 1.500140 -1.656805
H -3.316849 -0.379445 -1.377056
K -4.765154 2.629778 -4.343425
F -3.232271 1.492541 -2.299438

ch2f-cf3_17a_TS-bF_khmds_2_b3pw91.log

SCF (RB3PW91) = -2549.44515006
E(SCF)+ZPE(0 K)= -2549.188325
H(298 K)= -2549.160164
G(298 K)= -2549.247480

Lowest Frequency = -431.5503cm-1

```
N       -3.624404    1.105190   -6.307194
K       -4.061557   -1.017604   -4.673844
Si       -1.970005    1.368621   -6.253432
Si       -4.782314    0.953672   -7.508551
C       -1.149660    1.919357   -7.859572
C       -1.519721    2.696901   -4.976643
C       -1.011737   -0.169362   -5.688915
C       -4.172040    1.038452   -9.290488
C       -5.731453   -0.686779   -7.383045
C       -6.138551    2.273555   -7.365331
H       -0.085336    2.121120   -7.697945
H       -1.606108    2.836388   -8.243812
H       -1.227894    1.156745   -8.638852
H       -0.433147    2.815528   -4.912799
H       -1.860906    2.434494   -3.968711
H       -1.925103    3.680568   -5.242162
H       -1.246634   -1.040916   -6.310291
H       -1.205147   -0.424741   -4.640706
H        0.068226   -0.004525   -5.764124
H       -3.438780    0.253836   -9.498757
H       -3.702096    2.000241   -9.512482
H       -5.005390    0.906786   -9.988814
H       -5.071166   -1.556982   -7.478504
H       -6.465936   -0.764231   -8.191310
H       -6.300618   -0.772789   -6.449825
H       -6.845492    2.194011   -8.197708
H       -5.726120    3.288604   -7.395311
H       -6.734558    2.162740   -6.451729
C       -4.022208    1.106426   -2.008470
C       -5.273620    0.518547   -2.041211
F       -5.396831   -0.832855   -1.934712
F       -5.959140    0.577342   -3.534495
F       -6.227482    1.120167   -1.340094
K       -4.714250    2.760809   -4.451183
H       -3.742066    1.734769   -1.176097
F       -2.997193    0.224758   -2.412751
```

ch2f-cf3_17b_TS-bF_khmds_2_pbe0.log

```
SCF (RPBE1PBE) =      -2548.41891595
E(SCF)+ZPE(0 K)=      -2548.161549
H(298 K)=             -2548.133420
G(298 K)=             -2548.220231
Lowest Frequency = -447.5255cm-1
```

```
N       -3.618028    1.102351   -6.314877
K       -4.066647   -1.024107   -4.689860
Si       -1.964322    1.366707   -6.250344
Si       -4.787774    0.962592   -7.505998
C       -1.122232    1.859018   -7.863982
C       -1.524550    2.735672   -5.013020
C       -1.017260   -0.152412   -5.621016
C       -4.197202    1.067320   -9.293372
C       -5.734668   -0.679215   -7.386208
C       -6.143928    2.279283   -7.335571
H       -0.055334    2.045663   -7.698882
H       -1.557996    2.773371   -8.278697
H       -1.208114    1.075957   -8.622933
H       -0.438833    2.870675   -4.959696
H       -1.854153    2.496503   -3.994650
H       -1.945290    3.706757   -5.301742
H       -1.232997   -1.042785   -6.223382
H       -1.235903   -0.377943   -4.570317
H        0.064183    0.013975   -5.674593
H       -3.470702    0.281323   -9.521702
H       -3.723872    2.029748   -9.508610
H       -5.039831    0.950005   -9.983622
H       -5.074195   -1.548955   -7.489859
H       -6.473109   -0.752503   -8.191877
H       -6.300305   -0.771199   -6.450636
H       -6.858427    2.206949   -8.162707
H       -5.734201    3.296146   -7.359692
H       -6.732579    2.157884   -6.417834
C       -4.020538    1.084999   -2.011944
C       -5.260367    0.484066   -2.070089
F       -5.356916   -0.868626   -2.021874
F       -5.950648    0.585241   -3.562213
F       -6.226158    1.038683   -1.355321
K       -4.708749    2.755773   -4.453638
H       -3.752441    1.726131   -1.185859
F       -2.982349    0.243796   -2.443254
```

ch2f-cf3_17c_TS-bF_khmds_2_wb97xd.log

```
SCF (RwB97XD) =       -2549.59673090
E(SCF)+ZPE(0 K)=      -2549.337917
H(298 K)=             -2549.310172
G(298 K)=             -2549.395915
Lowest Frequency = -469.6713cm-1
```

```
N       -3.639912    1.100224   -6.321780
K       -4.091031   -1.036033   -4.653135
Si       -1.988146    1.362883   -6.250291
Si       -4.786572    0.955085   -7.531591
C       -1.149106    1.903858   -7.851559
C       -1.543660    2.694465   -4.974817
C       -1.030654   -0.169330   -5.668087
C       -4.161434    1.062572   -9.308373
C       -5.732112   -0.689102   -7.434497
C       -6.153525    2.264512   -7.391223
H       -0.085667    2.099358   -7.680304
H       -1.595145    2.821650   -8.245502
H       -1.224166    1.139131   -8.629060
H       -0.458059    2.815743   -4.907827
H       -1.888365    2.430451   -3.968305
H       -1.952415    3.676281   -5.240334
H       -1.262506   -1.048882   -6.278643
H       -1.228137   -0.411974   -4.617832
H        0.048747   -0.003625   -5.741773
H       -3.427994    0.279883   -9.522290
H       -3.686655    2.025638   -9.513811
H       -4.988946    0.942697  -10.014869
H       -5.067369   -1.556345   -7.520105
H       -6.452119   -0.764915   -8.255194
H       -6.315488   -0.781369   -6.510863
H       -6.846609    2.191551   -8.235120
H       -5.749183    3.282806   -7.399128
H       -6.761973    2.136224   -6.488374
C       -3.973749    1.104239   -1.994819
C       -5.231893    0.529774   -1.988798
F       -5.374101   -0.809750   -1.859188
F       -5.941932    0.572241   -3.479899
F       -6.164538    1.156596   -1.290757
K       -4.763648    2.755928   -4.433574
H       -3.668332    1.741738   -1.179324
F       -2.970044    0.204642   -2.408824
```

ch2f-cf3_18a_chf-cf2_b3pw91.log

```
SCF (RB3PW91) =       -376.318069619
E(SCF)+ZPE(0 K)=      -376.288850
H(298 K)=             -376.283434
G(298 K)=             -376.316587
Lowest Frequency = 233.9297cm-1
```

```
C       -0.093783   -0.409816   -0.000000
H       -1.172138   -0.464832    0.000000
C        0.580776    0.724120   -0.000000
F        0.002019    1.907013    0.000000
F        1.891410    0.825487    0.000000
F        0.559608   -1.581178    0.000000
```

ch2f-cf3_18b_chf-cf2_pbe0.log

```
SCF (RPBE1PBE) =      -376.084799141
E(SCF)+ZPE(0 K)=      -376.055342
```

H(298 K)= -376.049945
G(298 K)= -376.083062
Lowest Frequency = 233.4754cm-1

C	-0.093037	-0.409621	-0.000000
H	-1.171734	-0.463959	0.000000
C	0.580378	0.723671	-0.000000
F	0.004038	1.903521	0.000000
F	1.887543	0.823869	0.000000
F	0.560704	-1.576687	0.000000

ch2f-cf3_18c_chf-cf2_wb97xd.log

SCF (RwB97XD) = -376.346790261
E(SCF)+ZPE(0 K)= -376.317267
H(298 K)= -376.311887
G(298 K)= -376.344972
Lowest Frequency = 238.6848cm-1

C	-0.092665	-0.408860	0.000000
H	-1.170363	-0.463253	-0.000000
C	0.580466	0.722972	0.000000
F	0.003114	1.903216	-0.000000
F	1.888004	0.825085	-0.000000
F	0.559337	-1.578366	-0.000000

chf2-cf3_01a_chf2-cf3_b3pw91.log

SCF (RB3PW91) = -576.079541296
E(SCF)+ZPE(0 K)= -576.042230
H(298 K)= -576.035151
G(298 K)= -576.073006
Lowest Frequency = 79.0812cm-1

C	0.345398	-0.221212	0.004047
H	0.701131	0.281775	0.905870
C	0.847047	0.489528	-1.262723
F	2.182089	0.501770	-1.278371
F	0.412362	1.751716	-1.278793
F	0.417017	-0.119551	-2.367860
F	0.784601	-1.498118	-0.016577
F	-1.005015	-0.233961	-0.015040

chf2-cf3_01b_chf2-cf3_pbe0.log

SCF (RPBE1PBE) = -575.737580360
E(SCF)+ZPE(0 K)= -575.699894
H(298 K)= -575.692846
G(298 K)= -575.730645
Lowest Frequency = 78.9335cm-1

C	0.345847	-0.220583	0.002649
H	0.701430	0.282474	0.905139
C	0.846665	0.489006	-1.261177
F	2.177672	0.500482	-1.277391
F	0.412901	1.747272	-1.277708
F	0.416720	-0.119680	-2.362110
F	0.783936	-1.493619	-0.020096
F	-1.000542	-0.233404	-0.018752

chf2-cf3_01c_chf2-cf3_wb97xd.log

SCF (RwB97XD) = -576.130341564
E(SCF)+ZPE(0 K)= -576.092644
H(298 K)= -576.085615
G(298 K)= -576.123362
Lowest Frequency = 80.8335cm-1

C	0.344747	-0.222160	0.004564
H	0.700914	0.281528	0.904972
C	0.847255	0.489836	-1.263793
F	2.179081	0.502318	-1.279107
F	0.413934	1.749103	-1.279501
F	0.418926	-0.116800	-2.367147
F	0.782364	-1.496357	-0.015442
F	-1.002590	-0.235521	-0.013991

chf2-
cf3_02a_encountcmplx_dimer_intact_1_b3pw91.log

SCF (RB3PW91) = -3522.72248142
E(SCF)+ZPE(0 K)= -3522.230444
H(298 K)= -3522.183680
G(298 K)= -3522.312584
Lowest Frequency = 12.4243cm-1

K	4.340085	3.680872	5.467274
Si	7.251377	4.180235	7.800702
Si	5.646797	6.616867	7.118699
N	6.234520	5.072363	6.797655
C	8.904904	5.029349	8.162790
C	7.712832	2.537464	6.986886
C	6.518365	3.728521	9.482562
C	6.704075	7.980744	6.331552
C	5.505746	7.094501	8.940050
C	3.902604	6.838304	6.416487
H	9.550360	4.411020	8.796281
H	9.457815	5.239604	7.240177
H	8.754328	5.983570	8.677330
H	8.339347	1.941962	7.658934
H	6.838102	1.928767	6.744007
H	8.283660	2.670704	6.062368
H	6.457258	4.602990	10.135872
H	5.506990	3.325554	9.377880
H	7.126843	2.975334	9.994300
H	7.741122	7.908789	6.675977
H	6.709468	7.949129	5.236657
H	6.336365	8.975315	6.605792
H	6.478056	7.078893	9.440983
H	5.097174	8.105392	9.045078
H	4.842591	6.410800	9.477833
H	3.550543	7.864742	6.561467
H	3.852754	6.644600	5.341027
H	3.181688	6.184322	6.920974
N	5.460897	4.031379	2.965541
K	7.470185	5.201347	4.338309
Si	4.543107	5.222821	2.207278
Si	5.736026	2.483510	2.361676
C	4.475944	5.147768	0.321188
C	2.730237	5.221012	2.764781
C	5.207209	6.950093	2.604358
C	4.153238	1.506441	2.007960
C	6.762328	2.420636	0.776239
C	6.702069	1.436717	3.604344
H	3.873515	5.970952	-0.077866
H	4.032443	4.213168	-0.034394
H	5.476647	5.228328	-0.113366
H	2.143885	5.957601	2.205125
H	2.601040	5.470266	3.823442
H	2.275015	4.240348	2.591433
H	6.214681	7.097115	2.197796
H	5.233082	7.157748	3.677826
H	4.572720	7.721657	2.156350
H	3.543664	2.005009	1.248003
H	3.534812	1.404802	2.906740
H	4.370949	0.495882	1.645487
H	6.228660	2.863882	-0.068375
H	7.015821	1.390138	0.504810
H	7.700629	2.972075	0.899459
H	6.880881	0.436730	3.195181
H	6.176392	1.296888	4.553536
H	7.682159	1.866542	3.833543
C	3.175248	0.895846	7.593202
C	3.986503	0.028886	8.567837
H	3.931331	-1.026438	8.291887
F	3.233786	2.192539	7.921358
F	3.665916	0.772524	6.350995
F	1.901283	0.520084	7.583033

F	3.491636	0.210531	9.809849
F	5.267801	0.447472	8.549319

chf2-cf3_02b_encountcmplx_dimer_intact_1_pbe0.log

SCF (RPBE1PBE) = -3521.16424048
E(SCF)+ZPE(0 K)= -3520.671868
H(298 K)= -3520.624795
G(298 K)= -3520.754899
Lowest Frequency = 17.7559cm-1

K	4.219257	3.845538	5.492492
Si	7.134975	4.217137	7.897160
Si	5.696335	6.719031	7.113078
N	6.201959	5.135161	6.833681
C	8.752566	5.042153	8.431387
C	7.664607	2.591212	7.090587
C	6.250652	3.727308	9.493939
C	6.849463	8.002801	6.328821
C	5.529149	7.240566	8.920880
C	3.986646	7.035591	6.364149
H	9.338504	4.390521	9.089506
H	9.380484	5.285133	7.566529
H	8.566740	5.975119	8.973207
H	8.215887	1.974976	7.809140
H	6.812204	1.997312	6.748833
H	8.329946	2.736864	6.231923
H	6.090916	4.597637	10.137601
H	5.269158	3.289286	9.286460
H	6.828861	2.994116	10.066955
H	7.868252	7.891696	6.716222
H	6.895543	7.929666	5.235993
H	6.522381	9.023842	6.554844
H	6.479185	7.170166	9.459389
H	5.187322	8.279239	8.991354
H	4.800435	6.616326	9.447378
H	3.689506	8.079401	6.512172
H	3.955174	6.857010	5.284855
H	3.214559	6.420689	6.842754
N	5.403599	4.005605	2.970932
K	7.459495	5.063901	4.377824
Si	4.545948	5.233062	2.200657
Si	5.655514	2.435096	2.417249
C	4.397437	5.115426	0.322183
C	2.752933	5.376162	2.807770
C	5.342620	6.919552	2.524856
C	4.085545	1.375972	2.384993
C	6.403950	2.281198	0.688447
C	6.864854	1.516147	3.542111
H	3.859161	5.984734	-0.071832
H	3.848570	4.220848	0.011616
H	5.380127	5.087482	-0.158102
H	2.204982	6.125794	2.226025
H	2.659347	5.682843	3.855608
H	2.226893	4.422635	2.685321
H	6.343206	6.978743	2.079681
H	5.424841	7.154292	3.591111
H	4.750108	7.724718	2.077198
H	3.335568	1.812898	1.716822
H	3.626175	1.284198	3.375784
H	4.290233	0.359613	2.029882
H	5.705980	2.594476	-0.092414
H	6.687576	1.243112	0.481370
H	7.305347	2.896267	0.593245
H	6.993332	0.483936	3.198164
H	6.526073	1.466193	4.580574
H	7.861532	1.973227	3.539686
C	3.468173	0.699376	7.267726
C	4.198142	-0.150628	8.314657
H	4.348907	-1.170636	7.952754
F	3.275036	1.949864	7.698036
F	4.188509	0.769705	6.144981
F	2.288690	0.167824	6.980628

F	3.457322	-0.161978	9.438021
F	5.381119	0.425859	8.586620

chf2-cf3_02c_encountcmplx_dimer_intact_1_wb97xd.log

SCF (RwB97XD) = -3522.90984831
E(SCF)+ZPE(0 K)= -3522.414946
H(298 K)= -3522.368387
G(298 K)= -3522.498139
Lowest Frequency = 14.2132cm-1

K	4.262474	3.725813	5.425050
Si	7.122712	4.166493	7.868555
Si	5.646548	6.662238	7.109914
N	6.169819	5.088023	6.833580
C	8.799380	4.949735	8.270888
C	7.536616	2.497869	7.081323
C	6.336478	3.763534	9.539084
C	6.787085	7.962241	6.330132
C	5.483972	7.178191	8.919977
C	3.936991	6.959248	6.354312
H	9.401969	4.306892	8.920997
H	9.385259	5.138028	7.364515
H	8.672974	5.909026	8.782543
H	8.110311	1.877621	7.777048
H	6.641107	1.929965	6.814623
H	8.143049	2.596469	6.175265
H	6.270041	4.655340	10.168214
H	5.323012	3.369785	9.420180
H	6.922075	3.017477	10.085707
H	7.812212	7.844230	6.696616
H	6.812983	7.916025	5.235880
H	6.463802	8.976633	6.584951
H	6.442245	7.123673	9.445004
H	5.123045	8.208711	8.998901
H	4.776722	6.536780	9.453556
H	3.623114	7.998315	6.493702
H	3.918783	6.772075	5.276567
H	3.172896	6.331936	6.827314
N	5.459407	4.057213	2.900466
K	7.498833	5.119693	4.375338
Si	4.572567	5.277045	2.160042
Si	5.731003	2.493523	2.350980
C	4.456393	5.201835	0.276294
C	2.768290	5.343641	2.746437
C	5.304787	6.978852	2.547503
C	4.176622	1.409145	2.342272
C	6.459033	2.341784	0.613862
C	6.956328	1.599432	3.479805
H	3.915357	6.071185	-0.111119
H	3.924639	4.307109	-0.060532
H	5.447293	5.192860	-0.186406
H	2.201223	6.090761	2.181870
H	2.657719	5.614075	3.801984
H	2.276239	4.377482	2.592512
H	6.310791	7.087002	2.126531
H	5.359330	7.176111	3.622445
H	4.693131	7.777586	2.116793
H	3.412324	1.830164	1.681388
H	3.728785	1.311293	3.337304
H	4.394096	0.395647	1.989514
H	5.744896	2.645462	-0.155525
H	6.751077	1.307689	0.403544
H	7.350134	2.967569	0.502887
H	7.102136	0.566456	3.148422
H	6.615241	1.555017	4.517616
H	7.944568	2.072564	3.475954
C	3.399428	0.710361	7.473055
C	4.103489	-0.098301	8.577250
H	4.202217	-1.146386	8.288795
F	3.265961	1.995992	7.811542
F	4.109769	0.664062	6.341351
F	2.194002	0.212875	7.231961

F 3.376399 0.005366 9.705741
F 5.316332 0.438478 8.799198

chf2-cf3_03a_encountcmplx_dimer_intact_2_b3pw91.log

SCF (RB3PW91) = -3522.72334934
E(SCF)+ZPE(0 K)= -3522.229138
H(298 K)= -3522.183157
G(298 K)= -3522.308073
Lowest Frequency = 21.9812cm-1

K	4.216761	3.678118	5.345267
Si	7.098552	4.021192	7.823563
Si	5.572196	6.502116	7.126569
N	6.113753	4.938131	6.811971
C	8.802982	4.785317	8.136364
C	7.453318	2.330687	7.054887
C	6.387555	3.667285	9.538912
C	6.693429	7.834472	6.373155
C	5.403404	6.978964	8.945212
C	3.857925	6.789342	6.377975
H	9.419759	4.150173	8.781732
H	9.361125	4.940225	7.206093
H	8.709745	5.759677	8.626314
H	8.044575	1.713788	7.739786
H	6.542874	1.771357	6.820924
H	8.028159	2.407586	6.127083
H	6.426868	4.556954	10.172727
H	5.340347	3.358678	9.483539
H	6.948219	2.877469	10.050234
H	7.724109	7.715896	6.723696
H	6.704862	7.828491	5.277807
H	6.362801	8.836722	6.665902
H	6.365655	6.948311	9.464526
H	5.009846	7.996602	9.041424
H	4.718882	6.306988	9.470690
H	3.535010	7.823638	6.533929
H	3.837230	6.622100	5.297169
H	3.100146	6.149448	6.844894
N	5.444727	4.058642	2.897038
K	7.430877	5.113321	4.380445
Si	4.550054	5.271852	2.146327
Si	5.753182	2.525681	2.272503
C	4.503236	5.227621	0.259161
C	2.728556	5.278065	2.678762
C	5.227253	6.982556	2.586744
C	4.192510	1.506019	1.943084
C	6.747033	2.497124	0.665794
C	6.777026	1.505007	3.490698
H	3.917366	6.066131	-0.132522
H	4.048700	4.305543	-0.114912
H	5.509220	5.299251	-0.164320
H	2.156522	6.019573	2.110875
H	2.580626	5.527420	3.735056
H	2.269247	4.301173	2.494234
H	6.242171	7.123609	2.197265
H	5.243845	7.162685	3.665463
H	4.608387	7.773189	2.150205
H	3.549975	2.001826	1.208711
H	3.598676	1.365199	2.853081
H	4.431062	0.510232	1.553973
H	6.177575	2.907376	-0.171892
H	7.039154	1.475553	0.399361
H	7.662945	3.089434	0.764486
H	6.949073	0.499675	3.092113
H	6.290997	1.380439	4.462206
H	7.763946	1.944455	3.671495
C	3.403041	1.020300	7.755723
C	4.393126	0.512238	8.815143
H	5.363001	1.003080	8.720600
F	3.228127	2.343116	7.886942
F	3.890272	0.799346	6.526057
F	2.220447	0.427245	7.853672

F 4.532202 -0.821314 8.658880
F 3.868364 0.750095 10.034678

chf2-cf3_03b_encountcmplx_dimer_intact_2_pbe0.log

SCF (RPBE1PBE) = -3521.16454044
E(SCF)+ZPE(0 K)= -3520.669290
H(298 K)= -3520.623364
G(298 K)= -3520.747894
Lowest Frequency = 23.8561cm-1

K	4.223593	3.694527	5.364119
Si	7.140828	4.045373	7.820550
Si	5.561676	6.510410	7.155978
N	6.136288	4.960842	6.825190
C	8.840950	4.820834	8.126293
C	7.505480	2.365200	7.033843
C	6.446884	3.669627	9.538402
C	6.639838	7.874140	6.397255
C	5.400677	6.977082	8.977926
C	3.830281	6.761567	6.430958
H	9.468614	4.183242	8.759278
H	9.389409	4.990360	7.192197
H	8.745466	5.789543	8.628312
H	8.120676	1.753808	7.703232
H	6.599202	1.791929	6.814085
H	8.061424	2.456844	6.094689
H	6.490323	4.551809	10.183448
H	5.398673	3.360914	9.487243
H	7.012858	2.873833	10.035313
H	7.673636	7.790932	6.750614
H	6.655210	7.862822	5.301333
H	6.276268	8.867029	6.684627
H	6.368501	6.965488	9.488908
H	4.987373	7.986710	9.079790
H	4.734598	6.290554	9.509408
H	3.482230	7.784397	6.610378
H	3.796515	6.613663	5.346466
H	3.095040	6.094897	6.898064
N	5.423845	4.036181	2.897797
K	7.403385	5.151916	4.365975
Si	4.552546	5.258277	2.130676
Si	5.704054	2.495654	2.273137
C	4.544166	5.222621	0.242644
C	2.723150	5.283359	2.631282
C	5.231115	6.965603	2.585826
C	4.132228	1.552511	1.801636
C	6.819456	2.450598	0.748570
C	6.577509	1.402665	3.544274
H	3.961755	6.060684	-0.156077
H	4.102441	4.300337	-0.147418
H	5.558418	5.302667	-0.160800
H	2.170587	6.042671	2.066716
H	2.563840	5.517088	3.690418
H	2.251923	4.316032	2.425019
H	6.259146	7.101883	2.228549
H	5.209676	7.156398	3.663656
H	4.631559	7.755821	2.121243
H	3.574776	2.075156	1.017334
H	3.459396	1.437914	2.659453
H	4.360456	0.547479	1.429331
H	6.350307	2.941445	-0.108933
H	7.052779	1.421085	0.454848
H	7.768217	2.963087	0.942871
H	6.762996	0.409162	3.121391
H	5.993378	1.249314	4.457750
H	7.550647	1.808608	3.840696
C	3.455114	1.034253	7.750761
C	4.441090	0.521140	8.807879
H	5.416695	1.001968	8.710107
F	3.281439	2.353415	7.885069
F	3.941111	0.817076	6.524134
F	2.275470	0.443250	7.847500

F	4.564399	-0.810264	8.654758
F	3.921031	0.767051	10.023511

chf2-cf3_03c_encountcmplx_dimer_intact_2_wb97xd.log

SCF (RwB97XD) = -3522.91094052
E(SCF)+ZPE(0 K)= -3522.413042
H(298 K)= -3522.367654
G(298 K)= -3522.491344
Lowest Frequency = 19.8451cm-1

K	4.198500	3.671069	5.295006
Si	7.064803	4.005072	7.837808
Si	5.541187	6.492364	7.143343
N	6.087103	4.931483	6.832577
C	8.773816	4.756636	8.156965
C	7.413154	2.315309	7.062523
C	6.357700	3.647983	9.554133
C	6.676733	7.829620	6.420846
C	5.341104	6.956823	8.962849
C	3.842658	6.793055	6.363680
H	9.384433	4.113169	8.798987
H	9.336129	4.915493	7.230228
H	8.685234	5.727484	8.654758
H	7.987892	1.687118	7.750693
H	6.500774	1.766357	6.810442
H	8.003095	2.394390	6.144332
H	6.397804	4.536223	10.189918
H	5.310727	3.339577	9.499857
H	6.918574	2.857030	10.062531
H	7.700369	7.709122	6.790546
H	6.710616	7.827530	5.325875
H	6.340506	8.829897	6.712056
H	6.296358	6.929464	9.495329
H	4.938941	7.970286	9.060697
H	4.655736	6.275992	9.475607
H	3.516502	7.823791	6.532860
H	3.847078	6.646887	5.279174
H	3.074341	6.144302	6.799588
N	5.474270	4.076300	2.827975
K	7.464557	5.099735	4.386324
Si	4.596725	5.308332	2.096528
Si	5.768602	2.526035	2.252108
C	4.552837	5.294999	0.208627
C	2.771827	5.331978	2.619524
C	5.287159	7.004511	2.574040
C	4.220274	1.436380	2.170748
C	6.554026	2.418864	0.537413
C	6.956476	1.609605	3.403189
H	4.019788	6.172464	-0.171402
H	4.041867	4.407831	-0.176848
H	5.560802	5.307694	-0.215620
H	2.212543	6.082159	2.051294
H	2.620170	5.578436	3.676054
H	2.300979	4.361977	2.428261
H	6.306710	7.144200	2.197317
H	5.296554	7.159879	3.657353
H	4.679429	7.810085	2.150616
H	3.471108	1.871487	1.501818
H	3.746789	1.307449	3.150550
H	4.453071	0.434240	1.796129
H	5.866092	2.745375	-0.246444
H	6.851735	1.390451	0.308291
H	7.448989	3.045723	0.473309
H	7.122125	0.586854	3.050216
H	6.573441	1.533992	4.424731
H	7.941273	2.087373	3.452401
C	3.414755	1.007284	7.766823
C	4.408324	0.521365	8.837025
H	5.372131	1.023805	8.740125
F	3.214812	2.324228	7.887645
F	3.912348	0.787148	6.544173
F	2.244986	0.395005	7.860456

F	4.564624	-0.808577	8.695716
F	3.878551	0.764289	10.049811

chf2-cf3_04a_encountcmplx_dimer_intact_3_b3pw91.log

SCF (RB3PW91) = -3522.72275529
E(SCF)+ZPE(0 K)= -3522.231237
H(298 K)= -3522.184119
G(298 K)= -3522.314497
Lowest Frequency = 16.5172cm-1

K	4.300821	3.752991	5.512080
Si	7.295494	4.258026	7.790873
Si	5.657694	6.662982	7.120561
N	6.261107	5.126864	6.784379
C	8.842298	5.208299	8.326066
C	7.951745	2.720329	6.906221
C	6.488576	3.630813	9.380729
C	6.727312	8.047895	6.395204
C	5.451909	7.088709	8.949814
C	3.931135	6.896960	6.377606
H	9.503178	4.590301	8.943803
H	9.420656	5.541188	7.457189
H	8.584410	6.098429	8.907785
H	8.530318	2.101728	7.600001
H	7.152117	2.092584	6.506315
H	8.627663	2.961082	6.077592
H	6.267358	4.459518	10.059220
H	5.544720	3.120855	9.168250
H	7.136682	2.928258	9.915226
H	7.749277	7.985187	6.782966
H	6.781847	8.014403	5.301735
H	6.335586	9.036656	6.657138
H	6.403869	7.054822	9.487294
H	5.040378	8.096508	9.071672
H	4.767672	6.389900	9.440369
H	3.580044	7.921811	6.535569
H	3.906910	6.726457	5.297560
H	3.192603	6.239465	6.851574
N	5.416387	3.984658	3.000056
K	7.480435	5.109708	4.320784
Si	4.569271	5.209127	2.213965
Si	5.615059	2.411678	2.431828
C	4.521953	5.110678	0.328513
C	2.752636	5.308712	2.748162
C	5.320459	6.905294	2.596068
C	3.987495	1.517380	2.065991
C	6.663705	2.265036	0.866550
C	6.504073	1.340026	3.710627
H	3.970378	5.958868	-0.091172
H	4.032826	4.195817	-0.018558
H	5.531117	5.129587	-0.093353
H	2.213353	6.072744	2.178011
H	2.623035	5.567574	3.804437
H	2.249268	4.352324	2.572635
H	6.330164	7.001663	2.179631
H	5.366016	7.118599	3.668035
H	4.721045	7.704699	2.148708
H	3.422933	2.031191	1.281696
H	3.347007	1.472619	2.953679
H	4.154949	0.487792	1.731582
H	6.170052	2.725407	0.006761
H	6.862571	1.217889	0.613956
H	7.629835	2.764080	0.998147
H	6.631718	0.322683	3.325882
H	5.960019	1.248416	4.655486
H	7.503188	1.720951	3.943185
C	3.196347	0.795147	7.499132
C	4.149155	-0.024999	8.382865
F	3.259359	2.092073	7.839003
F	3.523505	0.702953	6.205278
F	1.944107	0.380491	7.650872
F	4.051732	-1.321728	8.022099

F	5.411252	0.390675	8.153254
H	3.901537	0.090700	9.440309

chf2-cf3_04b_encountcmplx_dimer_intact_3_pbe0.log

SCF (RPBE1PBE) = -3521.16631171
E(SCF)+ZPE(0 K)= -3520.674097
H(298 K)= -3520.626916
G(298 K)= -3520.758607
Lowest Frequency = 11.1069cm-1

K	4.482734	3.365671	5.464776
Si	7.601172	4.173392	7.613443
Si	5.504129	6.272649	7.221942
N	6.341834	4.887671	6.751924
C	8.993004	5.364168	8.091201
C	8.431893	2.810880	6.594304
C	7.087118	3.308417	9.212999
C	6.290893	7.872194	6.582214
C	5.299785	6.529552	9.082263
C	3.733160	6.267724	6.542795
H	9.812364	4.843247	8.599190
H	9.415722	5.861989	7.211165
H	8.632826	6.147089	8.766736
H	9.232365	2.343595	7.178532
H	7.721892	2.019118	6.335390
H	8.898242	3.157707	5.664435
H	6.593837	3.998503	9.903387
H	6.391449	2.489321	9.005975
H	7.954434	2.882269	9.729704
H	7.315666	7.972778	6.955493
H	6.328681	7.920606	5.488243
H	5.731574	8.753509	6.915686
H	6.265281	6.629149	9.588203
H	4.725755	7.439994	9.287836
H	4.767584	5.691133	9.542248
H	3.238182	7.216129	6.777291
H	3.687146	6.160663	5.453478
H	3.120066	5.481232	6.999484
N	5.372651	3.866074	2.916975
K	7.368722	5.190187	4.213639
Si	4.516979	5.086660	2.133030
Si	5.729573	2.337490	2.306585
C	4.674803	5.140066	0.250576
C	2.663532	5.040912	2.505432
C	5.103210	6.803818	2.687905
C	4.347796	1.504391	1.321176
C	7.264014	2.314536	1.203881
C	6.104405	1.114765	3.705901
H	4.122460	5.991920	-0.161819
H	4.285762	4.235274	-0.225272
H	5.721681	5.249593	-0.051684
H	2.123920	5.842350	1.988720
H	2.456375	5.154901	3.575762
H	2.229715	4.088364	2.182811
H	6.119913	7.027895	2.340609
H	5.062549	6.944649	3.772971
H	4.459093	7.576569	2.254910
H	4.094140	2.058480	0.412742
H	3.435580	1.421024	1.922033
H	4.636079	0.491192	1.019075
H	7.134821	2.982687	0.346020
H	7.478312	1.312398	0.816441
H	8.151728	2.650172	1.752321
H	6.485677	0.177092	3.287047
H	5.215612	0.841813	4.288325
H	6.869417	1.485060	4.395929
C	2.779141	2.144310	8.551470
C	3.651064	0.900146	8.335023
F	3.505992	3.126672	9.070003
F	2.277336	2.573043	7.385208
F	1.771475	1.872867	9.366379
F	2.883223	-0.088718	7.852132

F	4.581267	1.206812	7.398747
H	4.144513	0.587714	9.257561

chf2-cf3_04c_encountcmplx_dimer_intact_3_wb97xd.log

SCF (RwB97XD) = -3522.90988054
E(SCF)+ZPE(0 K)= -3522.414909
H(298 K)= -3522.368442
G(298 K)= -3522.497091
Lowest Frequency = 9.0804cm-1

K	4.270670	3.693167	5.393196
Si	7.122344	4.139453	7.844832
Si	5.613010	6.629740	7.118366
N	6.152517	5.063926	6.829185
C	8.812719	4.913278	8.207960
C	7.505214	2.467272	7.050921
C	6.376908	3.748570	9.537619
C	6.742975	7.949717	6.357137
C	5.437921	7.126962	8.932639
C	3.903338	6.914350	6.358128
H	9.424389	4.267740	8.846870
H	9.383188	5.097439	7.290880
H	8.702114	5.873801	8.721096
H	8.088048	1.841129	7.733533
H	6.600116	1.906216	6.801609
H	8.093987	2.562397	6.133079
H	6.341107	4.641007	10.168240
H	5.355063	3.369720	9.446579
H	6.967932	2.994323	10.066874
H	7.767127	7.839824	6.728795
H	6.776364	7.913894	5.262748
H	6.406656	8.957967	6.619401
H	6.396552	7.086753	9.458289
H	5.057359	8.149774	9.019524
H	4.742633	6.467804	9.460312
H	3.576471	7.948475	6.504137
H	3.892490	6.735353	5.278857
H	3.144651	6.273948	6.822311
N	5.475011	4.060988	2.879227
K	7.493827	5.130225	4.372945
Si	4.585193	5.282777	2.145572
Si	5.755045	2.503892	2.313845
C	4.490312	5.231844	0.259842
C	2.774851	5.326859	2.714747
C	5.297981	6.985899	2.563292
C	4.200118	1.421834	2.264675
C	6.513026	2.378344	0.587481
C	6.959218	1.591650	3.450359
H	3.945405	6.101118	-0.122272
H	3.971211	4.336628	-0.094914
H	5.486509	5.238496	-0.191460
H	2.206984	6.077692	2.155933
H	2.652869	5.580703	3.773206
H	2.292231	4.359089	2.542441
H	6.307620	7.109163	2.155309
H	5.337914	7.169880	3.641231
H	4.683885	7.784407	2.135680
H	3.445375	1.853539	1.599724
H	3.738812	1.309311	3.252123
H	4.421892	0.413550	1.899980
H	5.814212	2.699751	-0.188782
H	6.803733	1.346729	0.363733
H	7.409096	3.001337	0.503571
H	7.118866	0.567910	3.097372
H	6.594415	1.518669	4.478572
H	7.944260	2.070278	3.481773
C	3.383955	0.766486	7.587526
C	4.098521	0.003765	8.717207
F	3.355555	2.075760	7.862455
F	4.005552	0.614939	6.416117
F	2.134719	0.339595	7.459535
F	4.107166	-1.305984	8.404202

F	5.370370	0.433343	8.792086
H	3.591745	0.159069	9.671448

chf2-cf3_05a_encountcmplx_dimer_parsep_1_b3pw91.log

SCF (RB3PW91) = -3522.70960296
E(SCF)+ZPE(0 K)= -3522.217521
H(298 K)= -3522.170780
G(298 K)= -3522.299721
Lowest Frequency = 22.9125cm-1

C	-7.561059	0.982026	1.955748
C	-7.520387	-0.527652	1.671300
H	-8.101842	-0.775469	0.780798
F	-6.759817	1.302914	2.984066
F	-7.140924	1.664556	0.885555
F	-8.795728	1.372391	2.247747
K	-5.868653	3.248392	-1.311116
N	-4.757513	0.932818	-1.913244
Si	-3.407384	0.488147	-1.042527
Si	-5.458382	0.305508	-3.292029
C	-1.760708	0.983494	-1.831726
C	-3.442138	1.369134	0.640819
C	-3.243278	-1.357748	-0.662369
C	-6.782079	1.493061	-3.960938
C	-4.268912	0.009804	-4.734449
C	-6.359613	-1.338555	-3.026382
H	-1.723421	2.060484	-2.022927
H	-1.627299	0.477281	-2.793199
H	-0.904482	0.725689	-1.198441
H	-2.547565	1.174356	1.239784
H	-4.319976	1.028807	1.198074
H	-3.508240	2.447042	0.463487
H	-4.146741	-1.747756	-0.184918
H	-2.396346	-1.563341	0.001881
H	-3.083603	-1.929095	-1.582714
H	-6.357300	2.464372	-4.240855
H	-7.589344	1.661718	-3.237821
H	-7.253720	1.087319	-4.862004
H	-3.720975	0.924520	-4.982805
H	-4.790590	-0.324466	-5.638134
H	-3.530083	-0.756824	-4.478797
H	-6.856304	-1.691826	-3.936870
H	-7.122043	-1.240949	-2.246238
H	-5.661134	-2.117560	-2.704752
K	-4.552420	3.393039	2.780274
N	-5.193024	5.007600	0.744807
Si	-3.726212	5.633690	0.214837
Si	-6.675909	5.671906	1.165038
C	-3.615140	7.506679	0.035033
C	-2.293576	5.167967	1.372851
C	-3.209514	4.952625	-1.476478
C	-7.284422	5.027372	2.844617
C	-6.761755	7.548962	1.311719
C	-8.069626	5.205161	-0.036154
H	-3.780407	8.020322	0.985678
H	-4.350075	7.885023	-0.680832
H	-2.622265	7.792000	-0.328729
H	-1.363841	5.624824	1.018742
H	-2.099610	4.090236	1.413959
H	-2.451544	5.539875	2.392543
H	-3.138085	3.860577	-1.502998
H	-2.217765	5.329276	-1.746445
H	-3.890522	5.276023	-2.272519
H	-6.622863	5.319229	3.669348
H	-7.418485	3.940598	2.864656
H	-8.265547	5.454229	3.076025
H	-6.041786	7.927604	2.042631
H	-7.760264	7.857726	1.638996
H	-6.558701	8.042793	0.358084
H	-8.999804	5.703829	0.255049
H	-8.291664	4.132236	-0.040942
H	-7.852216	5.524754	-1.061793

F	-8.017370	-1.173846	2.747964
F	-6.236068	-0.896949	1.506073

chf2-cf3_05b_encountcmplx_dimer_parsep_1_pbe0.log

SCF (RPBE1PBE) = -3521.15426126
E(SCF)+ZPE(0 K)= -3520.661650
H(298 K)= -3520.614771
G(298 K)= -3520.744746
Lowest Frequency = 13.0398cm-1

C	-7.570960	0.936233	1.665822
C	-7.593174	-0.555401	1.308804
H	-8.354890	-0.765419	0.554280
F	-6.612598	1.204055	2.561489
F	-7.332347	1.668907	0.579117
F	-8.735338	1.307644	2.178242
K	-5.747632	3.327108	-1.380455
N	-4.685882	0.952462	-1.881328
Si	-3.325727	0.488719	-1.035283
Si	-5.513109	0.262471	-3.155411
C	-1.695820	0.986982	-1.856415
C	-3.320920	1.357957	0.654593
C	-3.154398	-1.358308	-0.665361
C	-6.810915	1.471487	-3.838218
C	-4.445535	-0.221221	-4.641384
C	-6.500715	-1.290680	-2.708664
H	-1.657603	2.067998	-2.029076
H	-1.592851	0.498385	-2.831552
H	-0.823278	0.712825	-1.252506
H	-2.415786	1.157350	1.236547
H	-4.190207	1.017434	1.227471
H	-3.384586	2.437803	0.481733
H	-4.028376	-1.742564	-0.130749
H	-2.269079	-1.564654	-0.052839
H	-3.052885	-1.935107	-1.591076
H	-6.358217	2.398545	-4.210733
H	-7.571661	1.730124	-3.090567
H	-7.350004	1.027032	-4.681924
H	-3.871364	0.637605	-5.005292
H	-5.045313	-0.601701	-5.476001
H	-3.728073	-1.002942	-4.368455
H	-7.015735	-1.712904	-3.579235
H	-7.260388	-1.064274	-1.952805
H	-5.849256	-2.068370	-2.296894
K	-4.517271	3.408691	2.748977
N	-5.156895	5.054633	0.737714
Si	-3.696795	5.727967	0.247583
Si	-6.663796	5.667700	1.152318
C	-3.626289	7.606702	0.113398
C	-2.274341	5.261402	1.416672
C	-3.133553	5.098932	-1.449327
C	-7.292180	4.929259	2.785478
C	-6.795955	7.532724	1.390101
C	-8.016581	5.232913	-0.105079
H	-3.821011	8.095287	1.072287
H	-4.356728	7.985416	-0.607867
H	-2.633510	7.923063	-0.225152
H	-1.347913	5.747840	1.093107
H	-2.058579	4.186211	1.429090
H	-2.457615	5.598787	2.444507
H	-3.012235	4.010593	-1.490563
H	-2.155573	5.523606	-1.699840
H	-3.817016	5.405721	-2.250584
H	-6.668047	5.215537	3.641536
H	-7.379783	3.836971	2.760354
H	-8.297372	5.304898	3.004252
H	-6.099270	7.891301	2.153869
H	-7.807978	7.801270	1.712612
H	-6.587195	8.079681	0.466474
H	-8.962281	5.707094	0.178782
H	-8.223413	4.158082	-0.162131
H	-7.772840	5.597653	-1.110031

F	-7.851635	-1.250649	2.433702
F	-6.379791	-0.906912	0.857928

chf2-
cf3_05c_encountcmplx_dimer_parsep_1_wb97xd.log

SCF (RwB97XD) = -3522.90566440
E(SCF)+ZPE(0 K)= -3522.409793
H(298 K)= -3522.363929
G(298 K)= -3522.489750
Lowest Frequency = 18.5680cm-1

C	-7.529906	0.876287	1.080453
C	-6.708902	-0.408769	0.895731
H	-6.064602	-0.314004	0.010569
F	-8.342849	0.818592	2.127093
F	-6.697555	1.918493	1.264481
F	-8.251579	1.126238	-0.007521
K	-5.826755	3.313288	-1.288190
N	-4.966607	0.778817	-1.639506
Si	-3.456012	0.468875	-0.998535
Si	-5.719800	0.150558	-2.995768
C	-1.995085	0.996353	-2.075014
C	-3.267055	1.420631	0.634470
C	-3.138565	-1.342790	-0.557140
C	-6.947230	1.394892	-3.735726
C	-4.563984	-0.315997	-4.418484
C	-6.750933	-1.394844	-2.640134
H	-2.079963	2.044893	-2.376949
H	-1.947727	0.395724	-2.988256
H	-1.041310	0.876029	-1.550740
H	-2.265770	1.321163	1.062331
H	-3.987485	1.023494	1.356225
H	-3.456805	2.485663	0.467437
H	-3.905989	-1.727892	0.121940
H	-2.166611	-1.483254	-0.072759
H	-3.152241	-1.969145	-1.454839
H	-6.448525	2.301223	-4.098176
H	-7.724275	1.685866	-3.019675
H	-7.466221	0.960284	-4.595763
H	-3.965481	0.542434	-4.738279
H	-5.123220	-0.675216	-5.288584
H	-3.870624	-1.109659	-4.122848
H	-7.165856	-1.823428	-3.558239
H	-7.591364	-1.169709	-1.976833
H	-6.146163	-2.168640	-2.156263
K	-4.553654	3.388559	2.852712
N	-5.170753	5.082103	0.816566
Si	-3.697139	5.720643	0.331148
Si	-6.659477	5.723941	1.244669
C	-3.589550	7.596925	0.178025
C	-2.282183	5.241780	1.505638
C	-3.130991	5.057707	-1.353085
C	-7.233119	5.136423	2.958931
C	-6.787832	7.603914	1.315817
C	-8.065645	5.177355	0.093344
H	-3.793882	8.096728	1.128446
H	-4.299592	7.983031	-0.558279
H	-2.585997	7.892672	-0.143948
H	-1.349678	5.713242	1.180819
H	-2.083385	4.163968	1.521124
H	-2.461398	5.583623	2.531697
H	-3.017241	3.968208	-1.364508
H	-2.149454	5.468110	-1.609059
H	-3.809310	5.348046	-2.163477
H	-6.544956	5.439162	3.756969
H	-7.387490	4.052840	3.014991
H	-8.199797	5.588192	3.201420
H	-6.086315	8.029985	2.038159
H	-7.796570	7.903649	1.617140
H	-6.582345	8.062656	0.345230
H	-9.006701	5.641158	0.404426
H	-8.239205	4.095926	0.116651
H	-7.896001	5.484976	-0.944665

F	-7.563014	-1.444715	0.777846
F	-5.972345	-0.599305	2.014785

chf2-
cf3_06a_encountcmplx_dimer_parsep_2_b3pw91.log

SCF (RB3PW91) = -3522.71635211
E(SCF)+ZPE(0 K)= -3522.224273
H(298 K)= -3522.177767
G(298 K)= -3522.304775
Lowest Frequency = 21.7396cm-1

C	-7.526218	0.912959	1.474895
C	-7.110384	-0.281028	0.609239
F	-6.511042	1.280493	2.278274
F	-7.818381	1.957188	0.686754
F	-8.581047	0.660008	2.240725
K	-5.880011	3.255441	-1.244000
N	-4.984193	0.735286	-1.509775
Si	-3.495149	0.395034	-0.819665
Si	-5.635450	0.169982	-2.951302
C	-1.991150	0.882770	-1.855118
C	-3.340866	1.362239	0.805438
C	-3.224682	-1.418175	-0.360899
C	-7.193205	1.159915	-3.395514
C	-4.500528	0.324120	-4.455638
C	-6.171438	-1.641657	-2.902200
H	-2.052786	1.921170	-2.193482
H	-1.910502	0.252977	-2.745962
H	-1.060972	0.770980	-1.287181
H	-2.349874	1.264630	1.257173
H	-4.080041	0.984649	1.518100
H	-3.518051	2.423510	0.606038
H	-4.047679	-1.802317	0.249674
H	-2.295213	-1.560739	0.201437
H	-3.164071	-2.040854	-1.259093
H	-6.965253	2.180450	-3.726749
H	-7.899889	1.214866	-2.560186
H	-7.724053	0.686097	-4.227594
H	-4.103133	1.339921	-4.548449
H	-5.031466	0.085549	-5.383863
H	-3.648017	-0.357668	-4.380168
H	-6.603883	-1.962726	-3.856082
H	-6.917424	-1.822242	-2.122942
H	-5.314766	-2.289601	-2.691952
K	-4.633966	3.470804	2.844402
N	-5.185195	5.040889	0.762672
Si	-3.701439	5.628631	0.232418
Si	-6.648346	5.774234	1.143837
C	-3.578397	7.484816	-0.068009
C	-2.304060	5.222574	1.451819
C	-3.151719	4.837569	-1.399806
C	-7.397539	5.036954	2.725280
C	-6.612837	7.632240	1.459981
C	-7.979016	5.519956	-0.184074
H	-3.738414	8.061685	0.846216
H	-4.313249	7.817715	-0.806691
H	-2.584936	7.739236	-0.452502
H	-1.361205	5.659105	1.106873
H	-2.118073	4.147046	1.550419
H	-2.495930	5.641633	2.446902
H	-3.092289	3.746126	-1.348528
H	-2.148148	5.183785	-1.666462
H	-3.805297	5.114720	-2.235320
H	-6.810694	5.285258	3.618368
H	-7.527543	3.950683	2.676880
H	-8.395373	5.453776	2.895260
H	-5.885483	7.890459	2.235257
H	-7.595497	7.976543	1.799772
H	-6.357059	8.197835	0.560789
H	-8.874688	6.100407	0.060280
H	-8.311545	4.480314	-0.275820
H	-7.637115	5.865339	-1.166333
F	-6.735744	-1.290731	1.430853

F	-8.207016	-0.681323	-0.079333
H	-6.296044	-0.004912	-0.092229

chf2-cf3_06b_encountcmplx_dimer_parsep_2_pbe0.log

SCF (RPBE1PBE) = -3521.15955225
E(SCF)+ZPE(0 K)= -3520.666673
H(298 K)= -3520.620147
G(298 K)= -3520.747290
Lowest Frequency = 22.0126cm-1

C	-7.492123	0.931086	1.488704
C	-7.097631	-0.279710	0.640042
F	-6.467311	1.302997	2.271418
F	-7.785807	1.960892	0.688902
F	-8.536116	0.696834	2.268507
K	-5.860776	3.266725	-1.264691
N	-4.958732	0.739105	-1.529517
Si	-3.478740	0.373643	-0.831112
Si	-5.628267	0.178810	-2.964990
C	-1.959763	0.842189	-1.853322
C	-3.308165	1.336628	0.796171
C	-3.240225	-1.444099	-0.371516
C	-7.120335	1.247111	-3.451582
C	-4.477059	0.219026	-4.464283
C	-6.280455	-1.593510	-2.881504
H	-1.988538	1.893716	-2.156850
H	-1.896915	0.239758	-2.764917
H	-1.034110	0.683224	-1.288536
H	-2.316666	1.220280	1.244123
H	-4.050790	0.974547	1.514501
H	-3.464613	2.402213	0.596813
H	-4.068787	-1.812666	0.242619
H	-2.312044	-1.603000	0.189155
H	-3.193134	-2.070377	-1.268781
H	-6.830997	2.249835	-3.790875
H	-7.841679	1.348873	-2.631788
H	-7.660024	0.791216	-4.288527
H	-4.021088	1.206646	-4.590591
H	-5.017330	-0.020889	-5.387177
H	-3.665222	-0.508369	-4.361393
H	-6.694295	-1.916186	-3.843482
H	-7.068620	-1.703018	-2.130237
H	-5.476379	-2.288435	-2.617090
K	-4.609869	3.484775	2.831439
N	-5.188849	5.057936	0.754605
Si	-3.708114	5.662962	0.235150
Si	-6.666197	5.758280	1.143750
C	-3.589346	7.525227	-0.026841
C	-2.307732	5.240331	1.446147
C	-3.154124	4.906923	-1.412471
C	-7.376025	5.026843	2.746014
C	-6.680963	7.621757	1.426457
C	-8.006646	5.445982	-0.161554
H	-3.763767	8.083993	0.896759
H	-4.314798	7.872160	-0.769029
H	-2.591078	7.790268	-0.392264
H	-1.366861	5.684828	1.104444
H	-2.116570	4.163648	1.529333
H	-2.495938	5.645575	2.448259
H	-3.072292	3.814905	-1.381116
H	-2.159045	5.278067	-1.679901
H	-3.818914	5.185826	-2.239290
H	-6.777766	5.293595	3.626719
H	-7.491182	3.937382	2.711792
H	-8.377586	5.430324	2.928943
H	-5.965544	7.915375	2.200817
H	-7.675114	7.945082	1.754372
H	-6.435440	8.178039	0.517765
H	-8.916426	6.001313	0.090910
H	-8.308581	4.395267	-0.237292
H	-7.692118	5.792021	-1.153278
F	-6.717503	-1.273011	1.471835

F	-8.204585	-0.683212	-0.021047
H	-6.292964	-0.020959	-0.076514

chf2-cf3_06c_encountcmplx_dimer_parsep_2_wb97xd.log

SCF (RwB97XD) = -3522.90562919
E(SCF)+ZPE(0 K)= -3522.410064
H(298 K)= -3522.364044
G(298 K)= -3522.491039
Lowest Frequency = 9.5121cm-1

C	-7.599806	0.917448	1.015834
C	-6.791816	-0.378012	0.848335
F	-6.756698	1.949393	1.209063
F	-8.301583	1.172419	-0.083827
F	-8.429197	0.874011	2.050239
K	-5.788633	3.324056	-1.313495
N	-4.963018	0.774019	-1.635684
Si	-3.475456	0.438939	-0.954350
Si	-5.692672	0.155439	-3.009137
C	-1.977313	0.936111	-1.993165
C	-3.312130	1.392556	0.680305
C	-3.203729	-1.376829	-0.499100
C	-6.887241	1.415367	-3.775959
C	-4.510257	-0.325607	-4.404747
C	-6.751850	-1.376549	-2.678407
H	-2.032949	1.986193	-2.296620
H	-1.918840	0.334645	-2.905195
H	-1.040003	0.796402	-1.444582
H	-2.324234	1.276757	1.134383
H	-4.057770	1.009876	1.384075
H	-3.478283	2.460237	0.505332
H	-3.996370	-1.745330	0.160017
H	-2.248117	-1.533294	0.012154
H	-3.204356	-2.006089	-1.394861
H	-6.369455	2.315157	-4.127764
H	-7.675690	1.716525	-3.076787
H	-7.393060	0.987136	-4.646967
H	-3.891458	0.524415	-4.708000
H	-5.053603	-0.674833	-5.288864
H	-3.836118	-1.130066	-4.094189
H	-7.147903	-1.801264	-3.606589
H	-7.606678	-1.139935	-2.037869
H	-6.169830	-2.157116	-2.177840
K	-4.637990	3.380767	2.867860
N	-5.175943	5.081292	0.814115
Si	-3.681518	5.702735	0.372525
Si	-6.669425	5.738592	1.199811
C	-3.545131	7.578085	0.230891
C	-2.307365	5.200932	1.585040
C	-3.075570	5.039650	-1.297909
C	-7.295597	5.159139	2.898417
C	-6.781657	7.619807	1.265360
C	-8.048091	5.204454	0.010126
H	-3.766314	8.076378	1.178339
H	-4.230723	7.977486	-0.521287
H	-2.529726	7.861409	-0.063935
H	-1.360717	5.665605	1.293089
H	-2.119914	4.121098	1.598003
H	-2.515072	5.536777	2.607711
H	-2.971700	3.949122	-1.310457
H	-2.083302	5.441534	-1.524374
H	-3.727800	5.339517	-2.126083
H	-6.626724	5.456786	3.714591
H	-7.461142	4.077083	2.951837
H	-8.264458	5.619801	3.113920
H	-6.095517	8.040671	2.005264
H	-7.795179	7.929060	1.539852
H	-6.547122	8.075245	0.299805
H	-8.992115	5.680663	0.292096
H	-8.235113	4.125309	0.031978
H	-7.844820	5.506336	-1.023512
F	-6.076300	-0.573171	1.980328

```
F       -7.655989   -1.404173    0.719361
H       -6.130521   -0.293575   -0.025402
```

chf2-cf3_07a_encountcmplx_dimer_parsep_3_b3pw91.log

```
SCF (RB3PW91) =       -3522.70889742
E(SCF)+ZPE(0 K)=      -3522.216575
H(298 K)=             -3522.169986
G(298 K)=             -3522.298116
Lowest Frequency = 19.6990cm-1

C       -7.737486    0.961510    1.946869
C       -7.834844   -0.484421    1.436822
F       -6.625840    1.118476    2.680560
F       -7.688137    1.830711    0.931498
F       -8.785394    1.260928    2.706067
K       -6.028113    3.117156   -1.153404
N       -4.694496    0.942211   -1.840262
Si       -3.352541    0.511054   -0.951548
Si       -5.357010    0.307826   -3.235159
C       -1.696933    1.046083   -1.694558
C       -3.441969    1.371536    0.739761
C       -3.164015   -1.335549   -0.583845
C       -6.752743    1.427282   -3.875008
C       -4.146265    0.132298   -4.680044
C       -6.153683   -1.396964   -3.029267
H       -1.663663    2.129424   -1.845511
H       -1.542825    0.575904   -2.671082
H       -0.850698    0.770858   -1.055166
H       -2.543157    1.219570    1.344979
H       -4.307583    0.990662    1.290428
H       -3.560364    2.443691    0.556882
H       -4.070669   -1.741795   -0.125459
H       -2.326140   -1.531832    0.094709
H       -2.979165   -1.899193   -1.504085
H       -6.395772    2.436594   -4.111740
H       -7.579155    1.507609   -3.158387
H       -7.183628    1.024343   -4.797471
H       -3.648536    1.084792   -4.889268
H       -4.642609   -0.197026   -5.599639
H       -3.367154   -0.600830   -4.446165
H       -6.601660   -1.754565   -3.963215
H       -6.941275   -1.371369   -2.269795
H       -5.414432   -2.138499   -2.710686
K       -4.484456    3.452292    2.841888
N       -5.202693    4.975554    0.776834
Si       -3.777927    5.559204    0.099024
Si       -6.645430    5.699719    1.238643
C       -3.673753    7.420680   -0.182884
C       -2.265782    5.140949    1.168833
C       -3.407585    4.798508   -1.595769
C       -7.280532    4.985120    2.880095
C       -6.613600    7.564188    1.518352
C       -8.062250    5.403899    0.012028
H       -3.752634    7.983355    0.750790
H       -4.466876    7.768363   -0.850988
H       -2.715439    7.678172   -0.646587
H       -1.360262    5.564471    0.722415
H       -2.080571    4.065195    1.261153
H       -2.348328    5.572858    2.173569
H       -3.372604    3.704527   -1.592779
H       -2.430129    5.136111   -1.954835
H       -4.140308    5.116966   -2.346874
H       -6.639317    5.252332    3.729200
H       -7.403270    3.897227    2.857584
H       -8.269337    5.395752    3.108340
H       -5.839975    7.845819    2.238740
H       -7.576103    7.903777    1.915791
H       -6.424571    8.115379    0.594032
H       -8.944032    5.985535    0.300295
H       -8.390112    4.359408   -0.026471
H       -7.790205    5.722178   -1.000644
F       -8.961062   -0.594542    0.700302
```

```
H       -7.858937   -1.194290    2.266881
F       -6.775445   -0.729682    0.643379
```

chf2-cf3_07b_encountcmplx_dimer_parsep_3_pbe0.log

```
SCF (RPBE1PBE) =      -3521.15367020
E(SCF)+ZPE(0 K)=      -3520.660909
H(298 K)=             -3520.614095
G(298 K)=             -3520.743596
Lowest Frequency = 17.4815cm-1

C       -7.624020    0.968893    1.754296
C       -7.736994   -0.544031    1.530103
F       -6.626917    1.229766    2.610161
F       -7.368102    1.613666    0.618268
F       -8.749206    1.445945    2.266432
K       -5.820255    3.261380   -1.328426
N       -4.648014    0.965960   -1.934961
Si       -3.329556    0.487015   -1.035731
Si       -5.445242    0.293878   -3.238265
C       -1.659034    0.966731   -1.782177
C       -3.387448    1.351295    0.656965
C       -3.202027   -1.362369   -0.658477
C       -6.818811    1.458808   -3.844122
C       -4.358990   -0.033309   -4.753669
C       -6.321634   -1.343454   -2.875069
H       -1.596798    2.048633   -1.941416
H       -1.523134    0.486690   -2.757552
H       -0.817227    0.672210   -1.145139
H       -2.506235    1.143473    1.272516
H       -4.280758    1.015215    1.194597
H       -3.439814    2.431681    0.484718
H       -4.116098   -1.734497   -0.184964
H       -2.362581   -1.580629    0.011858
H       -3.046957   -1.940213   -1.575993
H       -6.426070    2.431897   -4.164048
H       -7.587133    1.625274   -3.078524
H       -7.336212    1.032769   -4.710526
H       -3.845098    0.880363   -5.071309
H       -4.937360   -0.406879   -5.606411
H       -3.589693   -0.779375   -4.525474
H       -6.886551   -1.707704   -3.740815
H       -7.021657   -1.231000   -2.040212
H       -5.602748   -2.121023   -2.596487
K       -4.511761    3.418182    2.767201
N       -5.172135    5.033959    0.739769
Si       -3.711310    5.681711    0.217509
Si       -6.664808    5.672085    1.167789
C       -3.616247    7.557698    0.060470
C       -2.275764    5.215700    1.371984
C       -3.185645    5.024844   -1.480119
C       -7.254254    5.006638    2.846854
C       -6.783207    7.546115    1.331427
C       -8.051215    5.196300   -0.036468
H       -3.789229    8.059923    1.016524
H       -4.351048    7.939706   -0.654448
H       -2.623709    7.854732   -0.295896
H       -1.349203    5.683871    1.022834
H       -2.072197    4.138557    1.402288
H       -2.436165    5.576229    2.395929
H       -3.088686    3.933975   -1.515133
H       -2.203715    5.427317   -1.751091
H       -3.877282    5.338000   -2.271821
H       -6.597066    5.314488    3.670042
H       -7.361961    3.915977    2.868424
H       -8.245309    5.408857    3.082361
H       -6.071254    7.933388    2.066434
H       -7.788106    7.832875    1.660474
H       -6.589419    8.052627    0.381786
H       -8.988679    5.680398    0.257773
H       -8.259919    4.120574   -0.054439
H       -7.835528    5.529193   -1.058786
F       -8.722241   -0.763111    0.640129
```

H	-7.955444	-1.063070	2.466852
F	-6.580603	-0.987298	1.011352

chf2-cf3_07c_encountcmplx_dimer_parsep_3_wb97xd.log

SCF (RwB97XD) = -3522.89839331
E(SCF)+ZPE(0 K)= -3522.402379
H(298 K)= -3522.356482
G(298 K)= -3522.482515
Lowest Frequency = 19.6309cm-1

C	-7.698427	0.958765	1.920142
C	-7.801513	-0.480217	1.384434
F	-6.607773	1.089584	2.684848
F	-7.608827	1.841264	0.923182
F	-8.759525	1.261188	2.653718
K	-5.974056	3.159714	-1.208819
N	-4.686699	0.928978	-1.909041
Si	-3.355638	0.505579	-1.004534
Si	-5.386237	0.243774	-3.258474
C	-1.694402	1.014769	-1.752374
C	-3.441821	1.393237	0.674270
C	-3.179083	-1.335161	-0.602141
C	-6.769119	1.358093	-3.929474
C	-4.203253	-0.033121	-4.711154
C	-6.212056	-1.433915	-2.960847
H	-1.651276	2.095748	-1.918375
H	-1.545460	0.530585	-2.722869
H	-0.850414	0.740627	-1.110583
H	-2.558417	1.217050	1.295341
H	-4.327681	1.043298	1.214096
H	-3.522982	2.468881	0.488485
H	-4.082454	-1.726519	-0.125570
H	-2.335115	-1.523649	0.070047
H	-3.008636	-1.918642	-1.512559
H	-6.395906	2.349846	-4.209178
H	-7.580692	1.484685	-3.203218
H	-7.221757	0.926404	-4.827648
H	-3.693630	0.895606	-4.986282
H	-4.721967	-0.406904	-5.600267
H	-3.431905	-0.764888	-4.449245
H	-6.643775	-1.842074	-3.881032
H	-7.017102	-1.352487	-2.224446
H	-5.491208	-2.164223	-2.580347
K	-4.466191	3.433281	2.860502
N	-5.187975	5.027215	0.792509
Si	-3.756714	5.636147	0.158627
Si	-6.647773	5.715729	1.247125
C	-3.661533	7.505382	-0.076721
C	-2.256230	5.200775	1.238941
C	-3.332688	4.916985	-1.543280
C	-7.289064	4.981661	2.878087
C	-6.657317	7.580535	1.533490
C	-8.052366	5.401884	0.010300
H	-3.780241	8.045216	0.866161
H	-4.433886	7.862754	-0.763563
H	-2.690655	7.783325	-0.499266
H	-1.348445	5.642077	0.816275
H	-2.066183	4.123226	1.301119
H	-2.352769	5.600927	2.254993
H	-3.250371	3.824883	-1.548440
H	-2.363824	5.298367	-1.880069
H	-4.063356	5.211569	-2.305389
H	-6.658576	5.250813	3.734019
H	-7.395328	3.891817	2.847393
H	-8.285288	5.376453	3.099488
H	-5.901952	7.877572	2.266591
H	-7.632578	7.900406	1.914309
H	-6.462947	8.137566	0.613668
H	-8.943726	5.968653	0.296700
H	-8.363748	4.352768	-0.033027
H	-7.781939	5.727157	-1.000278
F	-8.905000	-0.564716	0.616966

H	-7.857551	-1.200385	2.203064
F	-6.726464	-0.728419	0.617770

chf2-cf3_08a_encountcmplx_dimer_sep_1_b3pw91.log

SCF (RB3PW91) = -3522.69780650
E(SCF)+ZPE(0 K)= -3522.206658
H(298 K)= -3522.159556
G(298 K)= -3522.290398
Lowest Frequency = 17.3337cm-1

C	-2.691683	3.747775	1.536083
C	-3.179579	2.309551	1.354830
H	-3.949017	1.995030	2.089933
F	-2.152320	3.897344	2.744210
F	-1.779443	4.082571	0.620471
F	-3.726011	4.587641	1.420740
K	-2.634984	-0.283469	-1.040223
N	-4.311927	-1.774723	-2.346685
Si	-3.565995	-3.256026	-2.580723
Si	-5.899472	-1.323015	-2.605239
C	-3.581990	-3.873749	-4.367887
C	-1.730970	-3.151953	-2.091879
C	-4.260345	-4.674032	-1.539432
C	-6.261574	0.320962	-1.720774
C	-6.347009	-1.028753	-4.418062
C	-7.202751	-2.511452	-1.924635
H	-3.162323	-3.125030	-5.047511
H	-4.606754	-4.076854	-4.695515
H	-3.007825	-4.798758	-4.489657
H	-1.223296	-4.095017	-2.318204
H	-1.590056	-2.986760	-1.016284
H	-1.192861	-2.371507	-2.643510
H	-4.284188	-4.402850	-0.479198
H	-3.657017	-5.583092	-1.639624
H	-5.283203	-4.921977	-1.837517
H	-5.576012	1.111447	-2.045374
H	-6.143691	0.144316	-0.648162
H	-7.288354	0.648135	-1.916376
H	-5.686024	-0.278751	-4.865197
H	-7.379295	-0.682337	-4.539523
H	-6.234976	-1.949391	-4.999474
H	-8.212028	-2.090455	-1.990992
H	-7.004888	-2.746676	-0.874542
H	-7.203960	-3.454644	-2.479714
K	-6.701253	2.491842	0.435874
N	-5.797052	1.352884	2.650927
Si	-5.770279	-0.331952	2.729391
Si	-6.362078	2.367582	3.872547
C	-5.360137	-1.051190	4.428477
C	-7.400285	-1.118051	2.182166
C	-4.463553	-1.071294	1.577148
C	-6.857677	4.065091	3.173776
C	-7.920601	1.776774	4.764228
C	-5.078162	2.729387	5.207522
H	-6.078755	-0.742199	5.192439
H	-4.367559	-0.726631	4.757087
H	-5.357764	-2.146246	4.402241
H	-7.352493	-2.212061	2.197970
H	-7.663282	-0.820672	1.161266
H	-8.221493	-0.810837	2.837314
H	-4.713871	-0.884071	0.530185
H	-4.422220	-2.158068	1.694150
H	-3.469154	-0.678341	1.809329
H	-7.782840	4.030661	2.584767
H	-6.071852	4.533125	2.572074
H	-7.062655	4.753992	3.999559
H	-8.742367	1.630879	4.055738
H	-8.252047	2.510032	5.507672
H	-7.762461	0.828694	5.285370
H	-5.460584	3.414895	5.971123
H	-4.177254	3.179206	4.778226
H	-4.775719	1.805649	5.710287

F -3.738593 2.218533 0.102160
F -2.103425 1.485273 1.363208

chf2-cf3_08b_encountcmplx_dimer_sep_1_pbe0.log

SCF (RPBE1PBE) = -3521.13572593
E(SCF)+ZPE(0 K)= -3520.644908
H(298 K)= -3520.597266
G(298 K)= -3520.731306
Lowest Frequency = 13.3572cm-1

C	-3.547548	3.119380	0.205178
C	-4.965149	2.622609	0.481485
H	-5.091479	2.100648	1.448517
F	-2.711075	2.075558	0.203347
F	-3.462337	3.700046	-0.996819
F	-3.156337	3.985612	1.122963
K	-3.584400	0.732270	-2.494841
N	-4.363443	-1.714638	-2.547066
Si	-3.160074	-2.735624	-2.001643
Si	-5.967907	-1.893609	-2.972839
C	-2.457444	-3.885865	-3.329397
C	-1.681026	-1.713308	-1.386101
C	-3.635691	-3.855373	-0.555427
C	-6.687644	-0.211467	-3.481917
C	-6.273362	-3.050412	-4.438430
C	-7.094555	-2.513497	-1.586698
H	-2.081831	-3.313146	-4.184398
H	-3.232986	-4.561890	-3.705548
H	-1.634136	-4.502397	-2.951094
H	-0.883283	-2.362165	-1.008715
H	-1.960644	-1.049672	-0.558221
H	-1.230568	-1.108472	-2.183789
H	-4.028742	-3.269086	0.281285
H	-2.778455	-4.432178	-0.190001
H	-4.411199	-4.569962	-0.851222
H	-6.139578	0.231096	-4.322942
H	-6.681243	0.501857	-2.648413
H	-7.731190	-0.305951	-3.801607
H	-5.680906	-2.749521	-5.309159
H	-7.327312	-3.066981	-4.738592
H	-5.986629	-4.077289	-4.186487
H	-8.143002	-2.562003	-1.902664
H	-7.036545	-1.850990	-0.717863
H	-6.800528	-3.514860	-1.255494
K	-7.958839	3.338947	2.297610
N	-6.239222	1.547071	3.091659
Si	-6.749625	0.003735	2.667161
Si	-5.291243	2.152042	4.336309
C	-7.379594	-1.060524	4.094394
C	-8.202280	0.138655	1.453799
C	-5.431222	-1.030168	1.797533
C	-5.468928	4.040922	4.395276
C	-5.735525	1.548000	6.068889
C	-3.443141	1.810745	4.134839
H	-8.151410	-0.539968	4.670868
H	-6.570826	-1.320798	4.784556
H	-7.811786	-1.997841	3.726476
H	-8.517167	-0.849590	1.103315
H	-7.943704	0.718918	0.559512
H	-9.086874	0.590709	1.921381
H	-5.100867	-0.574419	0.858769
H	-5.799055	-2.033399	1.560221
H	-4.549630	-1.143080	2.438150
H	-6.474169	4.352995	4.707153
H	-5.245051	4.507457	3.428220
H	-4.776463	4.478958	5.122027
H	-6.796374	1.709976	6.286857
H	-5.153491	2.074941	6.833411
H	-5.535397	0.478007	6.181865
H	-2.856675	2.252492	4.948107
H	-3.052620	2.210833	3.193574
H	-3.250469	0.732432	4.136393
F	-5.807188	3.686969	0.448968
F	-5.312206	1.802650	-0.544102

chf2-cf3_08c_encountcmplx_dimer_sep_1_wb97xd.log

SCF (RwB97XD) = -3522.88776477
E(SCF)+ZPE(0 K)= -3522.393388
H(298 K)= -3522.346589
G(298 K)= -3522.477355
Lowest Frequency = 15.9674cm-1

C	-2.842868	3.825559	1.405065
C	-3.147940	2.323369	1.352992
H	-3.897005	2.011475	2.101758
F	-2.419903	4.165094	2.615906
F	-1.919010	4.178366	0.516392
F	-3.961210	4.508808	1.136275
K	-2.497042	-0.368432	-1.028644
N	-4.255837	-1.822253	-2.335406
Si	-3.562918	-3.319257	-2.618664
Si	-5.831876	-1.327558	-2.579163
C	-3.685392	-3.923691	-4.406870
C	-1.705862	-3.281624	-2.215766
C	-4.253248	-4.726985	-1.559872
C	-6.159256	0.332559	-1.710626
C	-6.296813	-1.042232	-4.389789
C	-7.157149	-2.482268	-1.879957
H	-3.281544	-3.183667	-5.104750
H	-4.729813	-4.099845	-4.683683
H	-3.142284	-4.861988	-4.560177
H	-1.239497	-4.235752	-2.479291
H	-1.511947	-3.137110	-1.145873
H	-1.170888	-2.507355	-2.777765
H	-4.206458	-4.474860	-0.495758
H	-3.694259	-5.656961	-1.708175
H	-5.300729	-4.928951	-1.802125
H	-5.446042	1.102231	-2.024122
H	-6.071930	0.169075	-0.632927
H	-7.171840	0.685230	-1.933776
H	-5.637708	-0.300793	-4.852626
H	-7.327666	-0.689468	-4.499723
H	-6.199974	-1.968011	-4.965342
H	-8.155683	-2.035838	-1.933841
H	-6.952593	-2.722732	-0.832181
H	-7.190453	-3.425716	-2.433642
K	-6.661815	2.575571	0.440648
N	-5.842328	1.387688	2.701368
Si	-5.775418	-0.293292	2.738060
Si	-6.394157	2.381899	3.939902
C	-5.427331	-1.057780	4.431816
C	-7.357800	-1.104047	2.094805
C	-4.402007	-0.971524	1.622408
C	-6.843176	4.109094	3.288139
C	-7.971699	1.799182	4.805918
C	-5.121062	2.677556	5.303062
H	-6.191914	-0.796481	5.168514
H	-4.462748	-0.718669	4.822194
H	-5.391811	-2.150146	4.368022
H	-7.291956	-2.196719	2.102789
H	-7.571078	-0.799418	1.064295
H	-8.217889	-0.817806	2.708568
H	-4.608553	-0.720950	0.579645
H	-4.360092	-2.062817	1.681360
H	-3.421507	-0.584516	1.916874
H	-7.744494	4.106916	2.662989
H	-6.029478	4.582910	2.730294
H	-7.069072	4.771770	4.129169
H	-8.790457	1.680685	4.089066
H	-8.297804	2.520240	5.562744
H	-7.832778	0.837430	5.307444
H	-5.496252	3.356151	6.075690
H	-4.201112	3.111176	4.898206
H	-4.852585	1.735148	5.790225
F	-3.652235	2.053437	0.107333

F	-1.994040	1.635282	1.469176

chf2-cf3_09a_encountcmplx_dimer_sep_2_b3pw91.log

SCF (RB3PW91) = -3522.69710231
E(SCF)+ZPE(0 K)= -3522.205920
H(298 K)= -3522.158968
G(298 K)= -3522.289494
Lowest Frequency = 8.1687cm-1

C	-4.418933	2.634539	-3.119219
C	-3.772077	2.245441	-1.785984
F	-3.563780	2.445979	-4.120469
F	-5.492909	1.846166	-3.335761
F	-4.830909	3.898378	-3.139440
K	-5.689722	-1.061292	-3.244070
N	-3.513139	-0.922936	-1.817833
Si	-2.149655	-1.245484	-2.744381
Si	-3.887005	-1.221289	-0.213081
C	-1.205849	-2.811220	-2.275567
C	-2.642408	-1.460677	-4.563474
C	-0.882724	0.155480	-2.720215
C	-5.729998	-0.850473	0.046371
C	-3.654451	-2.991236	0.389742
C	-2.876938	-0.167849	1.000764
H	-1.861179	-3.687455	-2.279265
H	-0.770849	-2.726516	-1.275018
H	-0.385673	-3.003975	-2.975567
H	-1.758964	-1.602929	-5.194350
H	-3.164112	-0.580299	-4.957252
H	-3.277440	-2.341747	-4.716515
H	-1.284312	1.074266	-3.157398
H	0.018444	-0.107806	-3.284293
H	-0.577207	0.383989	-1.693971
H	-6.335260	-1.662151	-0.375514
H	-6.044230	0.078711	-0.442615
H	-6.035926	-0.787478	1.095756
H	-4.191481	-3.696617	-0.252406
H	-4.035384	-3.114155	1.409038
H	-2.600262	-3.280899	0.392449
H	-3.199434	-0.285252	2.041363
H	-2.844622	0.895916	0.742578
H	-1.836741	-0.507745	0.964667
K	-5.250836	1.803017	1.876611
N	-7.152115	0.594863	3.142922
Si	-8.620780	0.771008	2.367075
Si	-6.637291	-0.458738	4.333847
C	-9.979220	1.539078	3.437063
C	-8.444471	1.938893	0.878622
C	-9.364135	-0.826533	1.674549
C	-4.925446	0.056116	4.965862
C	-7.755334	-0.542045	5.858442
C	-6.432970	-2.249366	3.753238
H	-9.658581	2.507410	3.835488
H	-10.212117	0.895695	4.291292
H	-10.907597	1.697481	2.877115
H	-9.410845	2.096603	0.388327
H	-7.768438	1.537455	0.115743
H	-8.080952	2.929511	1.177181
H	-8.655653	-1.335957	1.014588
H	-10.280741	-0.638009	1.104422
H	-9.617263	-1.519139	2.483720
H	-4.926652	1.090641	5.325973
H	-4.155517	-0.033409	4.190599
H	-4.600148	-0.578080	5.797351
H	-7.922703	0.455463	6.277432
H	-7.329096	-1.171070	6.647723
H	-8.734442	-0.958066	5.599077
H	-6.074193	-2.902078	4.556942
H	-5.715999	-2.315272	2.929087
H	-7.382708	-2.656722	3.392890
F	-4.684553	2.504768	-0.800363
H	-3.506655	1.171272	-1.770711

F	-2.703080	3.037219	-1.572659

chf2-cf3_09b_encountcmplx_dimer_sep_2_pbe0.log

SCF (RPBE1PBE) = -3521.14178508
E(SCF)+ZPE(0 K)= -3520.649996
H(298 K)= -3520.602806
G(298 K)= -3520.735178
Lowest Frequency = 11.1615cm-1

C	-4.369940	2.667423	-3.034859
C	-3.737569	2.246804	-1.706415
F	-3.493647	2.541178	-4.022307
F	-5.414776	1.861637	-3.301017
F	-4.814443	3.915679	-3.013114
K	-5.596479	-0.998292	-3.373311
N	-3.452630	-0.955429	-1.889774
Si	-2.093556	-1.203590	-2.845134
Si	-3.821024	-1.326196	-0.299517
C	-1.148791	-2.802997	-2.513952
C	-2.599307	-1.266597	-4.672593
C	-0.821651	0.187423	-2.717285
C	-5.671517	-1.007630	-0.020122
C	-3.547273	-3.111515	0.238054
C	-2.847329	-0.295569	0.964140
H	-1.807444	-3.675684	-2.569405
H	-0.693072	-2.794718	-1.518491
H	-0.342853	-2.945134	-3.242561
H	-1.719267	-1.360615	-5.317724
H	-3.116853	-0.353122	-4.991858
H	-3.238101	-2.130340	-4.896882
H	-1.224694	1.143130	-3.067238
H	0.071593	-0.028101	-3.313972
H	-0.501034	0.325154	-1.678851
H	-6.265113	-1.793937	-0.503661
H	-6.005349	-0.048596	-0.434865
H	-5.968490	-1.035500	1.034080
H	-4.070196	-3.807408	-0.426270
H	-3.920923	-3.278358	1.254599
H	-2.486017	-3.376985	0.227533
H	-3.179940	-0.457698	1.996463
H	-2.838805	0.778329	0.745891
H	-1.797728	-0.607825	0.930720
K	-5.275546	1.629496	1.907760
N	-7.248024	0.538154	3.178318
Si	-8.695197	0.702174	2.362756
Si	-6.733712	-0.380857	4.474369
C	-10.088423	1.482748	3.376418
C	-8.466780	1.842052	0.860450
C	-9.411819	-0.907236	1.669807
C	-4.939073	0.060008	4.905485
C	-7.722608	-0.137373	6.068264
C	-6.722486	-2.247612	4.160210
H	-9.783778	2.456600	3.774578
H	-10.351680	0.848950	4.229931
H	-10.996974	1.632500	2.782038
H	-9.415530	2.002090	0.336656
H	-7.771603	1.419261	0.125163
H	-8.099094	2.832807	1.154431
H	-8.677690	-1.425272	1.043837
H	-10.307078	-0.729743	1.062771
H	-9.693168	-1.589664	2.478956
H	-4.824584	1.129843	5.116814
H	-4.241921	-0.209290	4.102181
H	-4.600853	-0.479678	5.796744
H	-7.767417	0.921349	6.344990
H	-7.286863	-0.686786	6.910457
H	-8.752955	-0.487836	5.944121
H	-6.311486	-2.804228	5.010251
H	-6.119080	-2.492566	3.279332
H	-7.734864	-2.622917	3.977121
F	-4.671177	2.441381	-0.731546
H	-3.441328	1.182597	-1.727780
F	-2.698656	3.057523	-1.446666

```
chf2-
cf3_09c_encountcmplx_dimer_sep_2_wb97xd.log

SCF (RwB97XD) =        -3522.88134357
E(SCF)+ZPE(0 K)=       -3522.387664
H(298 K)=              -3522.340713
G(298 K)=              -3522.472951
Lowest Frequency = 9.8662cm-1

C       -3.379296    2.514968   -0.927518
C       -4.382194    1.425450   -0.523826
F       -2.136921    2.128195   -0.682805
F       -3.490454    2.762437   -2.242600
F       -3.608496    3.655251   -0.277304
K       -3.627451    0.837435   -4.545722
N       -4.092201   -0.957660   -2.692929
Si      -2.698472   -1.734256   -2.176479
Si      -5.694068   -1.455724   -2.694499
C       -2.616491   -3.584153   -2.551075
C       -1.183088   -0.962996   -3.017435
C       -2.377499   -1.577601   -0.319290
C       -6.762999   -0.137441   -3.542877
C       -6.042646   -3.054871   -3.639645
C       -6.412938   -1.717711   -0.968264
H       -2.797916   -3.786901   -3.610839
H       -3.367160   -4.135127   -1.976332
H       -1.636595   -3.998754   -2.292966
H       -0.255214   -1.411269   -2.649044
H       -1.109561    0.112527   -2.818090
H       -1.195284   -1.115275   -4.103014
H       -2.166663   -0.545592   -0.024587
H       -1.519268   -2.183235   -0.010671
H       -3.244652   -1.913718    0.257857
H       -6.566287   -0.083182   -4.620651
H       -6.605655    0.857696   -3.114147
H       -7.828100   -0.365875   -3.438449
H       -5.663648   -3.001292   -4.665191
H       -7.117541   -3.257749   -3.692050
H       -5.568205   -3.914357   -3.157880
H       -7.486260   -1.930436   -0.989802
H       -6.262929   -0.845413   -0.327812
H       -5.920019   -2.564310   -0.480140
K       -6.627119    3.116060    1.712830
N       -6.708641    1.237495    3.519834
Si      -7.645008   -0.048665    3.017658
Si      -5.487045    1.420280    4.639728
C       -9.032895   -0.528042    4.211790
C       -8.531620    0.386116    1.394021
C       -6.693441   -1.652198    2.694000
C       -4.574863    3.062943    4.349664
C       -6.069582    1.484058    6.438766
C       -4.139948    0.091801    4.593843
H       -9.698905    0.319361    4.403177
H       -8.621836   -0.845206    5.175592
H       -9.641978   -1.351571    3.824230
H       -9.164001   -0.440488    1.056161
H       -7.836432    0.596983    0.574392
H       -9.189569    1.253965    1.522512
H       -5.803682   -1.473393    2.084736
H       -7.310150   -2.394622    2.177141
H       -6.360762   -2.098746    3.636477
H       -5.252478    3.923105    4.406002
H       -4.064750    3.083937    3.379145
H       -3.802218    3.224011    5.107887
H       -6.804124    2.282026    6.586888
H       -5.241908    1.657274    7.134550
H       -6.549581    0.542062    6.722944
H       -3.321995    0.325074    5.283810
H       -3.713529   -0.006541    3.591006
H       -4.540218   -0.886234    4.877118
F       -5.630760    1.929529   -0.742128
H       -4.243802    0.502687   -1.110406
F       -4.266646    1.211266    0.801235
```

```
chf2-
cf3_10a_encountcmplx_dimer_sep_3_b3pw91.log

SCF (RB3PW91) =        -3522.71840038
E(SCF)+ZPE(0 K)=       -3522.226703
H(298 K)=              -3522.180045
G(298 K)=              -3522.308414
Lowest Frequency = 17.0123cm-1

C       -9.312812   -0.702959    0.056506
C       -8.008424   -1.211160    0.675242
F       -9.839253   -1.582907   -0.798835
F      -10.207326   -0.453240    1.010211
F       -9.082394    0.435329   -0.616839
K       -6.100165    0.379234   -2.357934
N       -3.816448   -0.818270   -1.614392
Si      -2.653620    0.384631   -1.754680
Si      -3.976994   -2.387336   -2.189929
C       -1.067183   -0.061360   -2.668915
C       -3.318238    1.922845   -2.651150
C       -2.086138    1.030007   -0.065255
C       -5.494917   -2.591990   -3.314982
C       -2.536626   -3.078400   -3.189394
C       -4.264974   -3.646001   -0.800100
H       -1.260472   -0.338536   -3.708580
H       -0.548861   -0.897576   -2.191858
H       -0.381881    0.792934   -2.675049
H       -2.521755    2.665291   -2.764878
H       -4.122778    2.430499   -2.106710
H       -3.668925    1.686071   -3.662937
H       -2.905838    1.442998    0.531606
H       -1.363401    1.842515   -0.191567
H       -1.580599    0.253970    0.521387
H       -5.453901   -1.932046   -4.189782
H       -6.445964   -2.434027   -2.793738
H       -5.536231   -3.614804   -3.703207
H       -2.353684   -2.492557   -4.094131
H       -2.748833   -4.107588   -3.497725
H       -1.610824   -3.090877   -2.608053
H       -4.377075   -4.650149   -1.221581
H       -5.181252   -3.452519   -0.231773
H       -3.419789   -3.696458   -0.103779
K       -4.209788   -1.097201    1.103548
N       -6.047218    0.214649    2.558775
Si      -6.054626    1.860634    2.247361
Si      -6.226487   -0.597352    4.017474
C       -4.557939    2.795757    2.922952
C       -6.026486    2.134120    0.370136
C       -7.578862    2.787520    2.869340
C       -5.475944   -2.335707    3.899528
C       -5.382721    0.215904    5.500512
C       -8.030789   -0.850029    4.520654
H       -3.620383    2.330911    2.601658
H       -4.562315    2.801206    4.016951
H       -4.544285    3.838181    2.586414
H       -5.933236    3.190587    0.104165
H       -6.962708    1.764812   -0.058231
H       -5.176258    1.607911   -0.073841
H       -8.498103    2.331681    2.487455
H       -7.573429    3.838621    2.560572
H       -7.630928    2.765852    3.962551
H       -4.382379   -2.312114    3.819502
H       -5.874851   -2.900493    3.049475
H       -5.703461   -2.918219    4.798280
H       -4.315892    0.370468    5.310693
H       -5.475418   -0.400167    6.401681
H       -5.824400    1.192289    5.723034
H       -8.118664   -1.374029    5.478499
H       -8.577549   -1.434490    3.774446
H       -8.539925    0.113994    4.620309
F       -7.097296   -1.349590   -0.342500
H       -7.564422   -0.539614    1.436723
F       -8.225966   -2.439071    1.188836
```

chf2-cf3_10b_encountcmplx_dimer_sep_3_pbe0.log

SCF (RPBE1PBE) = -3521.14263775
E(SCF)+ZPE(0 K)= -3520.650443
H(298 K)= -3520.603565
G(298 K)= -3520.732268
Lowest Frequency = 21.5082cm-1

C	-4.036148	3.403319	-3.055465
C	-3.722379	2.647644	-1.763353
F	-3.622815	4.662678	-3.024338
F	-3.411890	2.787514	-4.076114
F	-5.339718	3.388625	-3.299582
K	-2.785409	-0.093727	-4.427949
N	-4.123982	-0.465650	-2.216186
Si	-3.106067	-1.097570	-1.039188
Si	-5.749550	-0.647149	-2.584290
C	-3.324604	-2.930385	-0.657471
C	-1.304314	-0.899172	-1.599407
C	-3.221737	-0.209931	0.626832
C	-6.067667	-0.094852	-4.370529
C	-6.439904	-2.396995	-2.467511
C	-6.880246	0.418330	-1.499960
H	-3.248028	-3.538121	-1.565051
H	-4.298898	-3.131959	-0.202868
H	-2.557031	-3.277940	0.043040
H	-0.612367	-1.197954	-0.804934
H	-1.060672	0.140667	-1.849964
H	-1.065833	-1.534153	-2.462646
H	-3.045958	0.866399	0.523192
H	-2.474678	-0.584771	1.333974
H	-4.201207	-0.378183	1.087923
H	-5.626877	-0.787202	-5.099354
H	-5.689563	0.913998	-4.573010
H	-7.141205	-0.074295	-4.585829
H	-5.861325	-3.091470	-3.085477
H	-7.479900	-2.432338	-2.810892
H	-6.419362	-2.771632	-1.440008
H	-7.928106	0.367400	-1.812206
H	-6.581891	1.472313	-1.527687
H	-6.837119	0.040682	-0.473030
K	-5.880940	1.919494	1.330044
N	-6.372279	0.204522	3.220678
Si	-7.431760	-1.047534	2.880395
Si	-5.164152	0.321570	4.372071
C	-8.088821	-1.986555	4.386885
C	-8.964352	-0.405072	1.974249
C	-6.682473	-2.361464	1.740945
C	-4.033544	1.801094	3.991328
C	-5.787816	0.622335	6.132870
C	-4.010853	-1.174518	4.498071
H	-8.605928	-1.312208	5.077674
H	-7.275034	-2.467095	4.940564
H	-8.796492	-2.771202	4.095615
H	-9.665184	-1.216106	1.746342
H	-8.710360	0.073459	1.021615
H	-9.497144	0.332075	2.584728
H	-6.346494	-1.905985	0.804046
H	-7.395390	-3.154097	1.486762
H	-5.810514	-2.830828	2.208437
H	-4.591829	2.745007	3.948768
H	-3.481856	1.674324	3.051907
H	-3.279065	1.924020	4.775827
H	-6.418970	1.516611	6.177146
H	-4.965012	0.756880	6.844248
H	-6.392404	-0.222055	6.480519
H	-3.201952	-1.005892	5.218251
H	-3.554505	-1.404607	3.530180
H	-4.559546	-2.064937	4.823775
F	-4.425023	3.226326	-0.751657
H	-3.973249	1.571117	-1.853444
F	-2.410907	2.799783	-1.495347

chf2-
cf3_10c_encountcmplx_dimer_sep_3_wb97xd.log

SCF (RwB97XD) = -3522.90043940
E(SCF)+ZPE(0 K)= -3522.404552
H(298 K)= -3522.358494
G(298 K)= -3522.485924
Lowest Frequency = 17.1296cm-1

C	-8.581667	-1.626034	0.022589
C	-8.456988	-2.983033	0.736406
F	-7.417164	-1.300440	-0.566739
F	-9.515110	-1.674561	-0.918635
F	-8.888375	-0.672470	0.887284
K	-6.059859	0.461861	-2.742450
N	-3.995733	-0.926664	-1.662882
Si	-2.731162	0.170196	-1.541030
Si	-4.154642	-2.483772	-2.262743
C	-1.066456	-0.370457	-2.244873
C	-3.122268	1.810678	-2.416308
C	-2.357924	0.673059	0.248227
C	-5.657655	-2.654365	-3.413918
C	-2.711306	-3.162556	-3.269692
C	-4.466780	-3.778822	-0.912403
H	-1.123400	-0.586648	-3.314827
H	-0.691461	-1.267698	-1.744881
H	-0.322765	0.420529	-2.104791
H	-2.260079	2.482153	-2.359806
H	-3.957099	2.354785	-1.960256
H	-3.334838	1.666619	-3.482164
H	-3.218056	1.111615	0.765682
H	-1.568244	1.430392	0.269171
H	-1.992979	-0.172751	0.842387
H	-5.563349	-2.038611	-4.316218
H	-6.608092	-2.419316	-2.922167
H	-5.746110	-3.688951	-3.759178
H	-2.487559	-2.527404	-4.131283
H	-2.950364	-4.161704	-3.647771
H	-1.799304	-3.244243	-2.673109
H	-4.519812	-4.780031	-1.350965
H	-5.417052	-3.628218	-0.389605
H	-3.659290	-3.801583	-0.172180
K	-4.812831	-1.212974	1.049521
N	-5.848112	0.687386	2.612429
Si	-6.200605	2.165951	1.933488
Si	-5.873381	0.171201	4.198428
C	-4.897048	3.505370	2.228086
C	-6.305057	1.988080	0.043642
C	-7.846617	2.936473	2.461791
C	-4.998848	-1.504701	4.375511
C	-5.009587	1.322696	5.428462
C	-7.611582	-0.102797	4.897742
H	-3.917802	3.187663	1.855978
H	-4.787843	3.712426	3.297269
H	-5.154231	4.447160	1.731820
H	-6.389030	2.952959	-0.464949
H	-7.178026	1.380777	-0.214373
H	-5.392492	1.494610	-0.305623
H	-8.675177	2.238846	2.305364
H	-8.067731	3.852272	1.903189
H	-7.832796	3.196543	3.525093
H	-3.953110	-1.454643	4.051393
H	-5.500938	-2.295644	3.806790
H	-4.989961	-1.832460	5.419792
H	-3.974779	1.513636	5.127398
H	-4.992398	0.903890	6.440169
H	-5.519602	2.289966	5.482107
H	-7.590276	-0.487060	5.923046
H	-8.167863	-0.818075	4.283351
H	-8.179585	0.832946	4.904782
F	-7.404409	-2.914903	1.581814
H	-9.364427	-3.225457	1.291455
F	-8.201218	-3.931111	-0.181685

chf2-cf3_11a_TS-H_dimer_intact_b3pw91.log

```
SCF (RB3PW91) =          -3522.70227876
E(SCF)+ZPE(0 K)=         -3522.214926
H(298 K)=                -3522.169321
G(298 K)=                -3522.292384
Lowest Frequency = -1158.7370cm-1

C       -5.350083    2.380314   -1.167661
C       -5.140984    0.890897   -0.956605
H       -4.617744   -0.091571   -1.956841
F       -4.175901    2.967365   -1.499209
F       -6.188835    2.583896   -2.210087
F       -5.847165    3.062517   -0.127023
K       -4.148644    1.503508   -4.014553
N       -4.198453   -1.062142   -2.666468
Si      -2.700631   -1.513100   -1.937412
Si      -5.592201   -2.052723   -2.902702
C       -1.709751   -2.801405   -2.905060
C       -1.620704    0.020111   -1.862829
C       -2.888800   -2.235777   -0.217647
C       -6.943335   -0.973839   -3.629332
C       -5.348653   -3.478162   -4.122714
C       -6.210267   -2.866719   -1.330481
H       -1.152147   -2.397690   -3.756036
H       -2.301986   -3.660987   -3.231396
H       -0.945510   -3.197039   -2.228140
H       -0.672907   -0.198391   -1.361577
H       -2.099183    0.833990   -1.312493
H       -1.368597    0.379478   -2.865686
H       -3.536020   -1.620966    0.410429
H       -1.913933   -2.312902    0.274543
H       -3.317010   -3.240962   -0.260267
H       -6.657984   -0.588410   -4.613016
H       -7.181314   -0.125801   -2.983052
H       -7.864168   -1.548205   -3.769328
H       -5.395715   -3.187903   -5.177153
H       -6.186731   -4.168659   -3.982144
H       -4.440493   -4.060652   -3.943464
H       -7.199730   -3.306964   -1.490975
H       -6.288398   -2.152143   -0.509092
H       -5.539079   -3.670417   -1.015347
N       -3.236010    0.682639   -6.418813
K       -3.126126   -1.803792   -5.493816
Si      -1.664317    1.267792   -6.476569
Si      -4.586806    0.779833   -7.409562
C       -1.059653    1.904005   -8.142980
C       -1.367047    2.688296   -5.253337
C       -0.420627   -0.066952   -5.959308
C       -4.311515    1.429298   -9.155780
C       -5.403128   -0.919285   -7.617982
C       -5.952239    1.874837   -6.675036
H       -0.008016    2.202691   -8.078794
H       -1.631254    2.774464   -8.475603
H       -1.140294    1.136441   -8.917784
H       -0.363060    3.103688   -5.389108
H       -1.418766    2.377248   -4.203598
H       -2.074509    3.510753   -5.407134
H       -0.426782   -0.925363   -6.641347
H       -0.589716   -0.428107   -4.938853
H        0.598224    0.333352   -5.971396
H       -3.572586    0.830996   -9.695792
H       -3.961438    2.465061   -9.149730
H       -5.245894    1.398718   -9.726087
H       -4.733121   -1.641372   -8.099429
H       -6.291008   -0.844094   -8.253829
H       -5.749485   -1.341096   -6.667899
H       -6.779569    1.978553   -7.384686
H       -5.589348    2.886764   -6.462150
H       -6.396652    1.464705   -5.760936
F       -4.327527    0.805063    0.166942
F       -6.378596    0.417871   -0.537750
```

chf2-cf3_11b_TS-H_dimer_intact_pbe0.log

```
SCF (RPBE1PBE) =         -3521.14251700
E(SCF)+ZPE(0 K)=         -3520.654182
H(298 K)=                -3520.608680
G(298 K)=                -3520.731240
Lowest Frequency = -1157.8531cm-1

C       -5.350116    2.384622   -1.186642
C       -5.145157    0.897931   -0.965418
H       -4.620311   -0.092034   -1.958696
F       -4.176445    2.965705   -1.514488
F       -6.180196    2.582429   -2.231952
F       -5.850098    3.069867   -0.155200
K       -4.138742    1.524367   -4.021061
N       -4.201563   -1.067730   -2.657791
Si      -2.702822   -1.514683   -1.925588
Si      -5.596282   -2.058935   -2.894236
C       -1.704971   -2.795810   -2.895894
C       -1.624665    0.019963   -1.839256
C       -2.887181   -2.237484   -0.204992
C       -6.954126   -0.984347   -3.615988
C       -5.351836   -3.482216   -4.116960
C       -6.219816   -2.872613   -1.323491
H       -1.147432   -2.385753   -3.744892
H       -2.292265   -3.657791   -3.227069
H       -0.939701   -3.190715   -2.218810
H       -0.681373   -0.202184   -1.329909
H       -2.106656    0.832499   -1.288318
H       -1.361966    0.384285   -2.838480
H       -3.526679   -1.616932    0.427046
H       -1.909260   -2.319319    0.281217
H       -3.321832   -3.240691   -0.241948
H       -6.676316   -0.594167   -4.600804
H       -7.195872   -0.138919   -2.966312
H       -7.872033   -1.565236   -3.752673
H       -5.402993   -3.189793   -5.171407
H       -6.188271   -4.175321   -3.975138
H       -4.441476   -4.063909   -3.942118
H       -7.204581   -3.321218   -1.492261
H       -6.313062   -2.153831   -0.506052
H       -5.546207   -3.670000   -0.995981
N       -3.231055    0.684904   -6.427984
K       -3.124750   -1.808558   -5.498572
Si      -1.657259    1.266168   -6.487579
Si      -4.585663    0.782655   -7.414630
C       -1.053907    1.916047   -8.149193
C       -1.352493    2.676256   -5.254076
C       -0.414730   -0.075121   -5.984865
C       -4.322359    1.445402   -9.157767
C       -5.395287   -0.918380   -7.633190
C       -5.953072    1.868015   -6.669189
H       -0.001882    2.213943   -8.081517
H       -1.624856    2.790272   -8.474938
H       -1.133869    1.155591   -8.931789
H       -0.346969    3.089321   -5.389383
H       -1.402492    2.358273   -4.205571
H       -2.056893    3.503431   -5.400342
H       -0.432492   -0.933384   -6.667713
H       -0.572776   -0.436727   -4.961817
H        0.606222    0.320688   -6.009157
H       -3.585194    0.853510   -9.708268
H       -3.975840    2.482937   -9.147683
H       -5.261167    1.415520   -9.721519
H       -4.719967   -1.635828   -8.115299
H       -6.280333   -0.843194   -8.273870
H       -5.745734   -1.347364   -6.686845
H       -6.784885    1.968333   -7.374846
H       -5.595399    2.882699   -6.457501
H       -6.391644    1.454821   -5.752753
F       -4.339074    0.818625    0.158934
F       -6.381803    0.432659   -0.548484
```

chf2-cf3_11c_TS-H_dimer_intact_wb97xd.log

SCF (RwB97XD) = -3522.88516307
E(SCF)+ZPE(0 K)= -3522.393655
H(298 K)= -3522.348970
G(298 K)= -3522.469529
Lowest Frequency = -1307.6537cm-1

C	-5.317919	2.351528	-1.121370
C	-5.139898	0.856606	-0.886226
H	-4.624810	-0.113771	-1.887281
F	-4.135021	2.907926	-1.458570
F	-6.147303	2.555478	-2.167575
F	-5.802714	3.053461	-0.094934
K	-4.168847	1.459408	-4.017757
N	-4.203990	-1.075692	-2.622889
Si	-2.699222	-1.521158	-1.918729
Si	-5.600303	-2.044905	-2.889732
C	-1.694085	-2.780476	-2.910635
C	-1.638268	0.025404	-1.826055
C	-2.874973	-2.272286	-0.208824
C	-6.929603	-0.934978	-3.614303
C	-5.366272	-3.454901	-4.129313
C	-6.241129	-2.867804	-1.330399
H	-1.129170	-2.342487	-3.739787
H	-2.280934	-3.629236	-3.273568
H	-0.936870	-3.198761	-2.240530
H	-0.680813	-0.184076	-1.340559
H	-2.125070	0.821609	-1.257416
H	-1.406435	0.407600	-2.826274
H	-3.527311	-1.673373	0.429420
H	-1.900806	-2.352580	0.282965
H	-3.297755	-3.279157	-0.268070
H	-6.631689	-0.554459	-4.597215
H	-7.148131	-0.082471	-2.966545
H	-7.864590	-1.483796	-3.758923
H	-5.429396	-3.141398	-5.176269
H	-6.195557	-4.155616	-3.992219
H	-4.450330	-4.032119	-3.972698
H	-7.232967	-3.298323	-1.498462
H	-6.316138	-2.160342	-0.502475
H	-5.578348	-3.679658	-1.017763
N	-3.226248	0.649869	-6.460135
K	-3.064264	-1.886627	-5.592188
Si	-1.670283	1.273561	-6.496651
Si	-4.577761	0.759387	-7.446642
C	-1.060169	1.921526	-8.157279
C	-1.414478	2.701135	-5.270771
C	-0.401500	-0.026070	-5.950966
C	-4.288282	1.360908	-9.208631
C	-5.443163	-0.920501	-7.609590
C	-5.921095	1.898431	-6.737411
H	-0.013751	2.234254	-8.084948
H	-1.640885	2.785286	-8.491607
H	-1.125806	1.156814	-8.936041
H	-0.428724	3.153999	-5.415483
H	-1.442055	2.380295	-4.222632
H	-2.153454	3.497989	-5.409067
H	-0.368117	-0.889189	-6.625771
H	-0.588024	-0.384336	-4.931753
H	0.605920	0.400832	-5.941201
H	-3.549551	0.744403	-9.728021
H	-3.930190	2.393624	-9.228648
H	-5.218547	1.322033	-9.784061
H	-4.809205	-1.673859	-8.091073
H	-6.342744	-0.830338	-8.225925
H	-5.777795	-1.311102	-6.641511
H	-6.729136	2.032408	-7.463216
H	-5.531260	2.896195	-6.508290
H	-6.396365	1.495818	-5.835135
F	-4.338810	0.772361	0.243667
F	-6.387511	0.417250	-0.467005

chf2-cf3_12a_TS-H_dimer_parsep_b3pw91.log

SCF (RB3PW91) = -3522.70399989
E(SCF)+ZPE(0 K)= -3522.216387
H(298 K)= -3522.170830
G(298 K)= -3522.293714
Lowest Frequency = -1108.2555cm-1

C	-5.879445	-0.175003	2.865039
C	-6.421903	-0.146000	1.446497
H	-5.696766	0.405488	0.257284
F	-4.824334	-0.992057	2.956046
F	-5.468029	1.069110	3.217591
F	-6.759417	-0.562471	3.808943
K	-4.378285	3.508820	-2.160340
N	-5.225945	0.865838	-0.822598
Si	-3.613150	0.251853	-0.854951
Si	-6.409014	0.621453	-2.054147
C	-3.565820	-1.611873	-1.056818
C	-2.537226	0.973273	-2.232945
C	-2.754457	0.713649	0.744203
C	-7.783800	1.884826	-1.828231
C	-5.736323	0.812599	-3.805768
C	-7.211794	-1.070602	-2.019280
H	-3.918134	-1.914081	-2.047431
H	-4.203636	-2.098155	-0.313414
H	-2.549127	-1.998414	-0.933812
H	-1.667556	0.317936	-2.347428
H	-2.122088	1.957201	-1.990977
H	-3.022808	1.013922	-3.210827
H	-2.790015	1.792254	0.918249
H	-1.698860	0.427162	0.701456
H	-3.193415	0.217717	1.610530
H	-7.438840	2.922338	-1.853261
H	-8.309520	1.725647	-0.881733
H	-8.529071	1.785880	-2.623190
H	-5.319834	1.786992	-4.076798
H	-6.563016	0.638035	-4.502126
H	-4.971997	0.060553	-4.017855
H	-7.905888	-1.174928	-2.859448
H	-7.776610	-1.228912	-1.098924
H	-6.468886	-1.868474	-2.096331
K	-6.165203	2.926137	1.103746
N	-5.069513	5.080162	-0.125770
Si	-3.654573	5.377871	0.727515
Si	-6.360361	6.018239	-0.646228
C	-3.235816	7.180597	1.079440
C	-3.637054	4.517467	2.417465
C	-2.138005	4.686081	-0.178815
C	-8.006795	5.437816	0.095019
C	-6.280144	7.863578	-0.275439
C	-6.597646	5.900540	-2.526271
H	-3.994698	7.660532	1.702723
H	-3.146817	7.760383	0.156538
H	-2.279447	7.249982	1.608530
H	-2.734311	4.788067	2.974796
H	-3.628147	3.424129	2.350182
H	-4.492313	4.820108	3.032258
H	-2.001104	5.148716	-1.163395
H	-2.172564	3.598748	-0.303507
H	-1.226614	4.891609	0.391741
H	-7.992580	5.487604	1.189977
H	-8.283758	4.421387	-0.205089
H	-8.825843	6.083217	-0.238785
H	-6.236349	8.055834	0.800058
H	-7.171399	8.366560	-0.665431
H	-5.406395	8.333692	-0.734192
H	-7.429855	6.535535	-2.847247
H	-6.842094	4.891042	-2.876225
H	-5.708674	6.248615	-3.065006
F	-6.838550	-1.436489	1.191148
F	-7.616458	0.602142	1.554248

chf2-cf3_12b_TS-H_dimer_parsep_pbe0.log

SCF (RPBE1PBE) = -3521.14223890
E(SCF)+ZPE(0 K)= -3520.655337

H(298 K)= -3520.609027
G(298 K)= -3520.735824
Lowest Frequency = -1108.0621cm-1

C	-4.810972	0.922234	2.528469
C	-5.233087	-0.177521	1.581304
H	-5.067581	0.114215	0.139046
F	-3.509494	1.203795	2.362283
F	-5.489829	2.061102	2.222009
F	-5.005506	0.709921	3.835274
K	-3.965930	2.910457	-0.312395
N	-4.998170	0.385255	-1.087354
Si	-3.631787	-0.283251	-1.874708
Si	-6.579461	0.487862	-1.735608
C	-3.974239	-1.901581	-2.765930
C	-2.906744	0.902335	-3.150861
C	-2.286603	-0.606418	-0.602655
C	-7.530405	1.774595	-0.732479
C	-6.621572	1.015733	-3.537227
C	-7.535265	-1.122488	-1.599406
H	-4.698300	-1.772341	-3.576416
H	-4.375965	-2.648381	-2.074044
H	-3.057636	-2.310120	-3.204679
H	-2.089083	0.416955	-3.695119
H	-2.482691	1.810602	-2.706839
H	-3.654374	1.211499	-3.886745
H	-1.983799	0.295977	-0.060292
H	-1.390709	-0.999475	-1.094638
H	-2.607716	-1.341700	0.140378
H	-7.047349	2.752317	-0.828240
H	-7.550986	1.459873	0.315829
H	-8.562190	1.877524	-1.080903
H	-6.117602	1.971696	-3.703537
H	-7.658865	1.124615	-3.872562
H	-6.147111	0.270247	-4.182640
H	-8.571505	-1.003555	-1.933619
H	-7.553293	-1.478599	-0.564809
H	-7.075081	-1.904609	-2.210989
K	-7.477971	4.039122	1.615368
N	-5.324944	5.163336	0.486375
Si	-4.433912	5.736434	1.792311
Si	-5.873045	5.840548	-0.951353
C	-4.015340	7.573294	1.803100
C	-5.321962	5.435341	3.444131
C	-2.771755	4.845062	1.983531
C	-7.767392	5.801975	-1.074167
C	-5.394159	7.628393	-1.300952
C	-5.272608	4.869027	-2.464587
H	-4.915981	8.193930	1.780012
H	-3.393603	7.856754	0.949343
H	-3.462206	7.825882	2.714392
H	-4.708397	5.813992	4.268546
H	-5.490858	4.374061	3.661667
H	-6.278388	5.969984	3.506585
H	-2.146587	4.949235	1.088527
H	-2.878690	3.780010	2.217918
H	-2.202934	5.278863	2.812897
H	-8.239519	6.349126	-0.248720
H	-8.181242	4.787023	-1.106508
H	-8.097414	6.289063	-1.997974
H	-5.785204	8.314304	-0.544364
H	-5.795830	7.940230	-2.271360
H	-4.308187	7.755507	-1.336837
H	-5.674657	5.307612	-3.383996
H	-5.601147	3.823980	-2.461000
H	-4.180155	4.893638	-2.560891
F	-4.556846	-1.308798	2.002830
F	-6.566254	-0.424213	1.886465

chf2-cf3_12c_TS-H_dimer_parsep_wb97xd.log

SCF (RwB97XD) = -3522.88701907
E(SCF)+ZPE(0 K)= -3522.396741
H(298 K)= -3522.351372
G(298 K)= -3522.474709
Lowest Frequency = -1272.1431cm-1

C	-4.604257	0.873461	2.614362
C	-5.510991	0.179381	1.610842
H	-5.179884	0.259889	0.208913
F	-3.413557	0.290148	2.696605
F	-4.404887	2.149629	2.187496
F	-5.088878	0.974160	3.863330
K	-3.958466	3.033759	-0.762473
N	-4.971961	0.368225	-1.047935
Si	-3.600679	-0.490936	-1.607396
Si	-6.405781	0.575712	-1.956273
C	-4.015176	-2.011172	-2.633314
C	-2.486810	0.596407	-2.681105
C	-2.553711	-1.049976	-0.155642
C	-7.488369	1.879726	-1.127346
C	-6.053413	1.169055	-3.707390
C	-7.460878	-0.972440	-2.082677
H	-4.553678	-1.753738	-3.549540
H	-4.636839	-2.708343	-2.064737
H	-3.101946	-2.538828	-2.925068
H	-1.724460	-0.013172	-3.176017
H	-1.939215	1.346054	-2.098479
H	-3.048890	1.111653	-3.465393
H	-2.174189	-0.202014	0.420036
H	-1.688023	-1.615193	-0.514099
H	-3.113325	-1.695242	0.526093
H	-6.918777	2.776770	-0.870681
H	-7.945575	1.472590	-0.222645
H	-8.294369	2.198225	-1.793445
H	-5.480233	2.100620	-3.727198
H	-6.988586	1.350738	-4.245982
H	-5.488502	0.423047	-4.273512
H	-8.425396	-0.757508	-2.553214
H	-7.657195	-1.384283	-1.088212
H	-6.967075	-1.748815	-2.672406
K	-7.097294	3.561715	1.844021
N	-5.336417	5.128638	0.429033
Si	-4.272105	5.727053	1.584744
Si	-6.235361	5.860691	-0.788198
C	-3.948262	7.585349	1.583284
C	-4.841503	5.355350	3.357583
C	-2.544803	4.950535	1.455233
C	-8.103360	5.628187	-0.541707
C	-6.022845	7.719782	-1.026239
C	-5.865456	5.126680	-2.497986
H	-4.860806	8.160684	1.758776
H	-3.516701	7.926952	0.639185
H	-3.240905	7.836341	2.380170
H	-4.145410	5.800220	4.075230
H	-4.874844	4.287340	3.595442
H	-5.824305	5.793758	3.566169
H	-2.087116	5.145897	0.478619
H	-2.532347	3.871414	1.639210
H	-1.879023	5.390908	2.203781
H	-8.434773	6.048606	0.414816
H	-8.426832	4.583348	-0.595283
H	-8.657457	6.155560	-1.324398
H	-6.319912	8.285706	-0.139855
H	-6.647844	8.057832	-1.859021
H	-4.989128	7.986811	-1.260151
H	-6.461663	5.633056	-3.263405
H	-6.107669	4.062846	-2.581998
H	-4.814911	5.267946	-2.777122
F	-5.695713	-1.103929	2.068217
F	-6.759350	0.808622	1.817533

chf2-cf3_13a_TS-H_dimer_sep_b3pw91.log

SCF (RB3PW91) = -3522.68731271
E(SCF)+ZPE(0 K)= -3522.200711
H(298 K)= -3522.154585
G(298 K)= -3522.280971

Lowest Frequency = -1208.5122cm-1

C	-3.461160	3.149107	-1.891493
C	-4.684410	2.335522	-2.263275
H	-4.713630	0.912122	-2.012500
F	-3.234615	3.039837	-0.570460
F	-2.386768	2.658666	-2.538636
F	-3.530062	4.458806	-2.175560
K	-3.741339	0.232844	-4.457707
N	-4.856900	-0.368988	-1.890498
Si	-3.518750	-1.183122	-1.182559
Si	-6.427507	-0.891859	-2.349711
C	-1.957028	-0.951982	-2.228931
C	-3.068647	-0.491956	0.505682
C	-3.710658	-3.035655	-1.004142
C	-6.787415	-0.419202	-4.145358
C	-6.737748	-2.733389	-2.239921
C	-7.774156	-0.048224	-1.341008
H	-1.704898	0.095935	-2.421150
H	-1.985133	-1.505343	-3.175599
H	-1.106464	-1.365396	-1.678128
H	-2.188912	-1.021525	0.883297
H	-3.855664	-0.626766	1.253740
H	-2.793856	0.566348	0.462498
H	-4.540526	-3.312570	-0.353213
H	-2.793145	-3.436945	-0.560966
H	-3.853468	-3.526854	-1.969474
H	-6.225929	-1.025241	-4.866833
H	-6.627524	0.643317	-4.355387
H	-7.842454	-0.615610	-4.359471
H	-6.047881	-3.306205	-2.863718
H	-7.753130	-2.934421	-2.597495
H	-6.666798	-3.109860	-1.218172
H	-8.756082	-0.338308	-1.726961
H	-7.728188	1.043665	-1.400964
H	-7.758957	-0.359433	-0.292444
K	-5.652587	1.304646	0.643815
N	-5.870453	0.298963	3.052519
Si	-6.808470	-1.081874	3.099172
Si	-4.700590	0.931737	4.059855
C	-6.821889	-2.031294	4.736384
C	-8.624486	-0.697246	2.724881
C	-6.267093	-2.339644	1.788806
C	-3.852048	2.416851	3.234774
C	-5.356215	1.581149	5.712474
C	-3.292983	-0.253731	4.508065
H	-7.193073	-1.403728	5.552743
H	-5.817702	-2.370220	5.009056
H	-7.465616	-2.916266	4.680387
H	-9.238419	-1.603064	2.672347
H	-8.739305	-0.167036	1.773547
H	-9.044511	-0.053585	3.505287
H	-6.234853	-1.888339	0.792791
H	-6.937714	-3.203810	1.731955
H	-5.261375	-2.709465	2.009999
H	-4.572815	3.188885	2.940879
H	-3.277414	2.123243	2.349217
H	-3.142040	2.892472	3.919515
H	-6.135676	2.333857	5.553635
H	-4.568524	2.036546	6.322914
H	-5.800499	0.768038	6.295827
H	-2.524055	0.233785	5.117850
H	-2.808539	-0.641550	3.606651
H	-3.669378	-1.111155	5.074851
F	-5.761045	2.956916	-1.646790
F	-4.875311	2.558094	-3.631863

chf2-cf3_13b_TS-H_dimer_sep_pbe0.log

SCF (RPBE1PBE) = -3521.13247218
E(SCF)+ZPE(0 K)= -3520.645048
H(298 K)= -3520.598956
G(298 K)= -3520.725797
Lowest Frequency = -1209.0824cm-1

C	-3.052202	2.932404	-1.739488
C	-4.394814	2.276413	-1.993603
H	-4.584130	0.857738	-1.802855
F	-2.748828	2.896011	-0.443470
F	-2.100571	2.237601	-2.406639
F	-2.953093	4.203800	-2.141972
K	-3.242453	0.230589	-4.148126
N	-4.846330	-0.406721	-1.813558
Si	-3.696063	-1.349701	-0.948860
Si	-6.308391	-0.800196	-2.624317
C	-2.071877	-1.532340	-1.906225
C	-3.220262	-0.504785	0.658461
C	-4.233543	-3.095651	-0.539118
C	-6.173048	-0.572152	-4.499107
C	-6.926906	-2.551113	-2.393265
C	-7.695976	0.356948	-2.091804
H	-1.548104	-0.580957	-2.059782
H	-2.189473	-2.051150	-2.865690
H	-1.386607	-2.151479	-1.317934
H	-2.390092	-1.050920	1.118512
H	-4.028100	-0.489344	1.398976
H	-2.872821	0.521158	0.501902
H	-5.162818	-3.125186	0.033624
H	-3.453980	-3.564537	0.071026
H	-4.366077	-3.709961	-1.433218
H	-5.463189	-1.267562	-4.964556
H	-5.942786	0.455587	-4.802709
H	-7.144657	-0.802589	-4.948069
H	-6.236215	-3.292038	-2.803599
H	-7.877649	-2.654109	-2.927940
H	-7.109342	-2.797384	-1.345242
H	-8.547978	0.243473	-2.769793
H	-7.406093	1.412233	-2.127381
H	-8.073958	0.116284	-1.092087
K	-6.032125	1.169075	0.580925
N	-6.005067	0.279176	3.041928
Si	-6.640105	-1.260198	3.134962
Si	-5.024547	1.221162	4.008733
C	-5.366359	-2.637555	3.397252
C	-7.935273	-1.510676	4.490918
C	-7.520011	-1.678297	1.507071
C	-4.441981	2.752332	3.049677
C	-5.867115	1.889639	5.565265
C	-3.440632	0.377424	4.611985
H	-4.921140	-2.570683	4.395637
H	-4.550259	-2.570158	2.670957
H	-5.816849	-3.632463	3.304097
H	-8.374591	-2.514420	4.461400
H	-8.749978	-0.785050	4.393030
H	-7.491709	-1.372883	5.483083
H	-8.343574	-0.982149	1.308684
H	-7.948120	-2.686291	1.521705
H	-6.831900	-1.645188	0.655364
H	-5.285004	3.351812	2.684624
H	-3.810434	2.485311	2.193827
H	-3.840128	3.411621	3.684659
H	-6.764218	2.463624	5.309041
H	-5.205981	2.542473	6.146572
H	-6.180799	1.068039	6.218453
H	-2.791907	1.072467	5.157405
H	-2.867145	-0.026274	3.771042
H	-3.669891	-0.455778	5.284714
F	-5.333253	3.058108	-1.345266
F	-4.635962	2.475916	-3.354805

chf2-cf3_13c_TS-H_dimer_sep_wb97xd.log

SCF (RwB97XD) = -3522.87450174
E(SCF)+ZPE(0 K)= -3522.383970
H(298 K)= -3522.338582
G(298 K)= -3522.462565
Lowest Frequency = -1355.1683cm-1

C	-3.531707	3.174084	-1.829055
C	-4.719136	2.327206	-2.259743
H	-4.730501	0.920150	-2.019286
F	-3.405036	3.121171	-0.492704
F	-2.408982	2.678459	-2.369415
F	-3.600276	4.465783	-2.168082
K	-3.710740	0.235646	-4.554278
N	-4.850003	-0.379727	-1.936039
Si	-3.514765	-1.159126	-1.195254
Si	-6.416621	-0.910353	-2.381934
C	-1.947376	-0.958623	-2.238786
C	-3.087738	-0.395562	0.468227
C	-3.693624	-3.006248	-0.941315
C	-6.788880	-0.448958	-4.177944
C	-6.721930	-2.753250	-2.248973
C	-7.765845	-0.071449	-1.371552
H	-1.697815	0.084689	-2.458249
H	-1.979497	-1.533439	-3.171978
H	-1.096410	-1.357439	-1.678961
H	-2.190365	-0.872243	0.872894
H	-3.877095	-0.534545	1.213232
H	-2.858991	0.671213	0.382783
H	-4.529277	-3.262665	-0.288846
H	-2.779279	-3.383507	-0.472445
H	-3.823696	-3.539144	-1.886148
H	-6.224850	-1.054141	-4.897599
H	-6.627184	0.613362	-4.388741
H	-7.843400	-0.645897	-4.391079
H	-6.022592	-3.336184	-2.852512
H	-7.731796	-2.967068	-2.613093
H	-6.660506	-3.110175	-1.218999
H	-8.749402	-0.368320	-1.746794
H	-7.724991	1.020087	-1.436702
H	-7.736063	-0.379617	-0.322149
K	-5.752165	1.446558	0.703231
N	-5.918183	0.355888	3.115967
Si	-6.843584	-1.032299	3.108009
Si	-4.676222	0.895784	4.088812
C	-6.917004	-1.995663	4.735421
C	-8.646790	-0.660423	2.666653
C	-6.243410	-2.274851	1.807040
C	-3.782551	2.357991	3.272195
C	-5.223591	1.518504	5.789627
C	-3.315507	-0.374545	4.442372
H	-7.329688	-1.377700	5.538891
H	-5.922639	-2.326717	5.050127
H	-7.548429	-2.885949	4.645551
H	-9.243045	-1.573796	2.570379
H	-8.734685	-0.111741	1.723108
H	-9.108569	-0.041643	3.442923
H	-6.184003	-1.814087	0.815758
H	-6.899367	-3.147532	1.722164
H	-5.239745	-2.633004	2.056450
H	-4.470048	3.177380	3.033520
H	-3.270351	2.056458	2.351643
H	-3.014953	2.771385	3.933946
H	-5.959116	2.323751	5.696133
H	-4.383508	1.898556	6.380450
H	-5.693003	0.710937	6.360747
H	-2.501286	0.054237	5.036228
H	-2.884062	-0.756485	3.512052
H	-3.712235	-1.230530	4.997289
F	-5.839034	2.939818	-1.721410
F	-4.831335	2.526033	-3.638731

chf2-cf3_14a_kcf2cf3_khmds_b3pw91.log

SCF (RB3PW91) = -2648.70774206
E(SCF)+ZPE(0 K)= -2648.457258
H(298 K)= -2648.428170
G(298 K)= -2648.521111
Lowest Frequency = 4.9933cm-1

N	-3.402338	1.102901	-6.336122
K	-3.585097	-1.047711	-4.719821
Si	-1.790029	1.558004	-6.365765
Si	-4.641082	0.973180	-7.457478
C	-1.007875	1.783598	-8.065221
C	-1.474898	3.191220	-5.446507
C	-0.699083	0.306269	-5.448271
C	-4.127804	0.801353	-9.262664
C	-5.734106	-0.532122	-7.088151
C	-5.844221	2.442228	-7.401199
H	0.050800	2.047887	-7.972239
H	-1.500261	2.582552	-8.627059
H	-1.071356	0.869492	-8.661906
H	-0.428315	3.493698	-5.556365
H	-1.648883	3.113331	-4.366324
H	-2.082331	4.012760	-5.843595
H	-0.745874	-0.691794	-5.899155
H	-0.961000	0.225887	-4.386204
H	0.351123	0.614362	-5.477628
H	-3.451491	-0.047271	-9.401728
H	-3.617016	1.696753	-9.626506
H	-5.003503	0.636567	-9.899373
H	-5.197827	-1.480176	-7.215225
H	-6.585428	-0.565625	-7.775720
H	-6.158069	-0.499687	-6.077422
H	-6.550196	2.394556	-8.236979
H	-5.317191	3.399377	-7.481173
H	-6.460414	2.458900	-6.493880
K	-4.480647	2.691237	-4.393902
C	-4.924224	0.443239	-2.622248
C	-4.416663	0.537341	-1.199846
F	-3.965406	1.787252	-0.959765
F	-3.380281	-0.306843	-1.024034
F	-5.303227	0.259959	-0.213608
F	-6.079013	1.277138	-2.621955
F	-5.477821	-0.864431	-2.700169

chf2-cf3_14b_kcf2cf3_khmds_pbe0.log

SCF (RPBE1PBE) = -2647.63157414
E(SCF)+ZPE(0 K)= -2647.380301
H(298 K)= -2647.351356
G(298 K)= -2647.442579
Lowest Frequency = 11.9874cm-1

N	-3.345460	1.202720	-6.328401
K	-3.551834	-0.859073	-4.570006
Si	-1.700439	1.502122	-6.426223
Si	-4.622261	0.958175	-7.386084
C	-0.993450	1.805366	-8.146165
C	-1.197854	3.013694	-5.393663
C	-0.665888	0.090058	-5.689754
C	-4.233812	1.093498	-9.224803
C	-5.425687	-0.747024	-7.159182
C	-6.045530	2.172203	-7.068301
H	0.080548	2.013882	-8.090348
H	-1.472751	2.661219	-8.631140
H	-1.129033	0.936535	-8.797015
H	-0.121083	3.194776	-5.480440
H	-1.393615	2.876781	-4.322479
H	-1.695400	3.931501	-5.730052
H	-0.876913	-0.870938	-6.173883
H	-0.807918	-0.022273	-4.607301
H	0.402925	0.284637	-5.829970
H	-3.491454	0.351392	-9.533680
H	-3.842108	2.082141	-9.481996
H	-5.136832	0.927938	-9.822459
H	-4.710791	-1.565896	-7.305455
H	-6.222304	-0.893350	-7.896595
H	-5.902741	-0.863820	-6.178004
H	-6.851760	2.014901	-7.792865
H	-5.730373	3.217483	-7.173511
H	-6.501205	2.037329	-6.079296
K	-4.285265	2.948896	-4.485316
C	-4.986522	0.795962	-2.654819

C   -4.728775   0.770293   -1.164583
F   -4.213269   1.945378   -0.765982
F   -3.830442   -0.186071   -0.871508
F   -5.799198   0.538553   -0.374369
F   -6.034055   1.743082   -2.794039
F   -5.648502   -0.436688   -2.895522

chf2-cf3_14c_kcf2cf3_khmds_wb97xd.log

SCF (RwB97XD) =   -2648.87247439
E(SCF)+ZPE(0 K)=   -2648.620196
H(298 K)=   -2648.591348
G(298 K)=   -2648.683905
Lowest Frequency = 3.8060cm-1

N   -3.531144   1.142112   -6.350483
K   -3.885374   -0.986196   -4.639750
Si   -1.893004   1.477980   -6.276202
Si   -4.675869   0.952663   -7.556009
C   -1.031246   1.841488   -7.913679
C   -1.514579   2.971645   -5.166979
C   -0.898313   0.071187   -5.477572
C   -4.054312   1.017821   -9.335177
C   -5.601422   -0.698585   -7.408370
C   -6.056260   2.252160   -7.450807
H   0.029245   2.057774   -7.750161
H   -1.472659   2.708138   -8.413755
H   -1.095042   0.994812   -8.602277
H   -0.439671   3.176976   -5.159527
H   -1.795614   2.799486   -4.121191
H   -2.004743   3.886930   -5.518066
H   -1.046824   -0.884332   -5.992841
H   -1.135250   -0.062444   -4.415267
H   0.173396   0.289007   -5.518994
H   -3.312085   0.238328   -9.528702
H   -3.591003   1.980303   -9.567651
H   -4.881319   0.869150 -10.036699
H   -4.927754   -1.559642   -7.485355
H   -6.332469   -0.800717   -8.216356
H   -6.170566   -0.780200   -6.474623
H   -6.745688   2.151463   -8.294802
H   -5.660728   3.273369   -7.484269
H   -6.669866   2.144661   -6.548354
K   -4.686615   2.817562   -4.494803
C   -4.929982   0.701383   -2.522806
C   -4.070663   0.857278   -1.281531
F   -3.531397   2.091426   -1.254840
F   -3.053036   -0.023906   -1.318638
F   -4.679539   0.683851   -0.090203
F   -6.013708   1.589551   -2.295667
F   -5.525945   -0.577100   -2.362802

chf2-cf3_15a_TS-bF_khmds_b3pw91_1a.log

SCF (RB3PW91) =   -2648.67535686
E(SCF)+ZPE(0 K)=   -2648.426110
H(298 K)=   -2648.397113
G(298 K)=   -2648.487552
Lowest Frequency = -439.2383cm-1

N   -3.726888   0.959480   -6.407285
K   -4.228011   -1.328807   -5.087436
Si   -2.083950   1.246079   -6.236118
Si   -4.845683   1.048421   -7.651208
C   -1.075949   1.358172   -7.825071
C   -1.712134   2.843401   -5.281143
C   -1.255362   -0.114764   -5.205877
C   -4.163217   1.326620   -9.385740
C   -5.894139   -0.530255   -7.758555
C   -6.127312   2.426214   -7.388410
H   -0.012213   1.484190   -7.596943
H   -1.384871   2.205959   -8.442374
H   -1.180837   0.451731   -8.428321
H   -0.636472   3.048132   -5.278746
H   -2.007569   2.787630   -4.227031
H   -2.200463   3.712004   -5.737180
H   -1.302290   -1.094693   -5.696096
H   -1.683679   -0.199529   -4.200481
H   -0.193033   0.107841   -5.063016
H   -3.456047   0.542380   -9.669522
H   -3.644062   2.286062   -9.462747
H   -4.972913   1.327828 -10.123099
H   -5.285506   -1.417479   -7.969427
H   -6.623566   -0.452799   -8.571380
H   -6.476418   -0.709614   -6.846564
H   -6.785966   2.504473   -8.259599
H   -5.656413   3.407902   -7.261509
H   -6.786549   2.237599   -6.532452
C   -4.014698   1.400420   -1.719329
C   -4.687580   0.192791   -1.893185
F   -3.965462   -0.904305   -2.139585
F   -5.517925   0.156432   -3.387213
F   -5.750687   -0.070063   -1.153129
K   -4.850148   2.489565   -4.420389
F   -2.646711   1.314148   -1.664988
F   -4.454719   2.177233   -0.678146

chf2-cf3_15b_TS-bF_khmds_pbe0.log

SCF (RPBE1PBE) =   -2647.59706479
E(SCF)+ZPE(0 K)=   -2647.347195
H(298 K)=   -2647.318269
G(298 K)=   -2647.407894
Lowest Frequency = -454.4186cm-1

N   -3.767880   1.169144   -6.385297
K   -4.223400   -0.996540   -4.766796
Si   -2.132805   1.485553   -6.193167
Si   -4.831316   0.955899   -7.661866
C   -1.182521   1.995423   -7.739299
C   -1.839739   2.879794   -4.939268
C   -1.185679   0.009039   -5.471617
C   -4.118416   1.118894   -9.398676
C   -5.676024   -0.744254   -7.622382
C   -6.281735   2.178907   -7.583950
H   -0.139680   2.221965   -7.491514
H   -1.615795   2.889596   -8.198163
H   -1.178940   1.203324   -8.493704
H   -0.766339   3.051365   -4.805126
H   -2.232093   2.640065   -3.943310
H   -2.265740   3.835484   -5.269566
H   -1.318803   -0.895237   -6.077100
H   -1.473337   -0.214811   -4.437247
H   -0.110570   0.217326   -5.444602
H   -3.333847   0.377759   -9.579709
H   -3.685066   2.109037   -9.567430
H   -4.899462   0.964942 -10.151257
H   -4.953757   -1.568540   -7.664022
H   -6.333529   -0.862234   -8.490487
H   -6.315978   -0.878530   -6.741306
H   -6.942421   2.048270   -8.447765
H   -5.943726   3.222057   -7.602839
H   -6.913101   2.027919   -6.699074
C   -3.704269   -0.163513   -1.886972
C   -4.770360   0.725668   -1.870221
F   -5.820926   0.459893   -1.120742
F   -5.627859   0.710270   -3.351348
F   -4.504776   2.031546   -1.919015
K   -5.024938   2.849126   -4.688833
F   -2.458272   0.392608   -1.820134
F   -3.780090   -1.206178   -1.008792

chf2-cf3_15c_TS-bF_khmds_wb97xd.log

SCF (RwB97XD) =   -2648.83694741
E(SCF)+ZPE(0 K)=   -2648.586283
H(298 K)=   -2648.557332
G(298 K)=   -2648.648221

Lowest Frequency = -468.4314cm-1

```
N       -3.683612    1.079293   -6.343628
K       -4.202055   -1.111667   -4.776532
Si       -2.039228    1.367213   -6.210991
Si       -4.803961    1.028550   -7.585967
C       -1.068870    1.480498   -7.824626
C       -1.657699    2.974153   -5.274900
C       -1.167412    0.029193   -5.188035
C       -4.170251    1.433777   -9.315565
C       -5.641357   -0.668597   -7.732680
C       -6.253334    2.219950   -7.292717
H       -0.004104    1.632932   -7.621248
H       -1.409728    2.312246   -8.446705
H       -1.165429    0.564600   -8.414571
H       -0.583735    3.184404   -5.291214
H       -1.936253    2.920589   -4.216166
H       -2.159086    3.837975   -5.724975
H       -1.249710   -0.963687   -5.644910
H       -1.553380   -0.021512   -4.163151
H       -0.098289    0.246548   -5.102041
H       -3.389435    0.738533   -9.634943
H       -3.752845    2.443635   -9.361694
H       -4.983969    1.378282  -10.045632
H       -4.912865   -1.474017   -7.877095
H       -6.316390   -0.692749   -8.593843
H       -6.258793   -0.908687   -6.859014
H       -6.952955    2.185514   -8.133596
H       -5.922405    3.260966   -7.203146
H       -6.837959    1.958100   -6.402886
C       -3.744615    0.436622   -2.131313
C       -5.117861    0.428023   -1.913050
F       -5.673609   -0.649485   -1.406123
F       -5.940023    0.369102   -3.431900
F       -5.723440    1.544102   -1.525681
K       -4.832556    2.609610   -4.383733
F       -3.123510    1.637393   -1.899689
F       -3.022466   -0.570368   -1.558643
```

chf2-cf3_16a_cf2-cf2_b3pw91.log

```
SCF (RB3PW91) =       -475.553918993
E(SCF)+ZPE(0 K)=      -475.532382
H(298 K)=             -475.526234
G(298 K)=             -475.561485
Lowest Frequency = 200.1470cm-1

C       -0.078671   -0.407826    0.000000
C        0.578013    0.736826    0.000000
F       -0.013058    1.910941   -0.000000
F        1.889876    0.819279   -0.000000
F        0.512381   -1.581943   -0.000000
F       -1.390539   -0.490252   -0.000000
```

chf2-cf3_16b_cf2-cf2_pbe0.log

```
SCF (RPBE1PBE) =      -475.269125106
E(SCF)+ZPE(0 K)=      -475.247327
H(298 K)=             -475.241203
G(298 K)=             -475.276408
Lowest Frequency = 202.5390cm-1

C       -0.078313   -0.407205   -0.000000
C        0.577655    0.736185   -0.000000
F       -0.012033    1.907150    0.000000
F        1.886091    0.818256    0.000000
F        0.511346   -1.578157    0.000000
F       -1.386745   -0.489204    0.000000
```

chf2-cf3_16c_cf2-cf2_wb97xd.log

```
SCF (RwB97XD) =       -475.593348287
E(SCF)+ZPE(0 K)=      -475.571530
H(298 K)=             -475.565429
G(298 K)=             -475.600588
Lowest Frequency = 202.7853cm-1

C       -0.077999   -0.406651    0.000000
C        0.577340    0.735653    0.000000
F       -0.011672    1.907721   -0.000000
F        1.886394    0.818844   -0.000000
F        0.510995   -1.578723   -0.000000
F       -1.387058   -0.489820   -0.000000
```

1a_diphenyltrifluoroethane_b3pw91.log

```
SCF (RB3PW91) =       -839.736788254
E(SCF)+ZPE(0 K)=      -839.521960
H(298 K)=             -839.507275
G(298 K)=             -839.564400
Lowest Frequency = 30.5654cm-1

C       -1.100615    0.541279    0.244109
H       -0.748384   -0.492667    0.232252
C       -0.571086    1.105039   -1.064384
C       -2.617176    0.502821    0.242102
C       -3.269798   -0.680348   -0.090014
C       -3.373808    1.624641    0.568219
C       -4.656570   -0.743359   -0.101136
H       -2.686599   -1.559948   -0.339880
C       -4.759615    1.564405    0.553715
H       -2.880703    2.548673    0.843589
C       -5.405329    0.380622    0.219276
H       -5.151195   -1.673064   -0.358133
H       -5.337395    2.445217    0.809406
H       -6.488234    0.333852    0.213554
C       -0.523000    1.197078    1.479094
C       -0.253687    0.379033    2.574455
C       -0.297052    2.566526    1.594259
C        0.225103    0.913125    3.761143
H       -0.425864   -0.688949    2.494073
C        0.187449    3.101595    2.780902
H       -0.493228    3.228993    0.761801
C        0.447561    2.279826    3.868256
H        0.428560    0.259674    4.601746
H        0.361645    4.169148    2.852142
H        0.824792    2.701055    4.792899
F        0.773773    1.163226   -1.074970
F       -0.930902    0.324199   -2.101747
F       -1.016862    2.342053   -1.349967
```

1b_TS-H_parsep_h_b3pw91.log

```
SCF (RB3PW91) =       -3786.36750599
E(SCF)+ZPE(0 K)=      -3785.701642
H(298 K)=             -3785.648722
G(298 K)=             -3785.786527
Lowest Frequency = -1438.0566cm-1

C       -4.394827    0.868110    2.365068
C       -5.149453   -0.262011    1.785990
H       -5.003314    0.010266    0.433034
F       -3.108237    0.897175    1.969071
F       -4.917554    2.077870    1.944027
F       -4.347145    0.995511    3.725018
K       -4.004301    2.948987   -0.544725
N       -4.788764    0.249084   -0.889770
Si       -3.359611   -0.334933   -1.637839
Si       -6.263034    0.348606   -1.750369
C       -3.623841   -1.879643   -2.682367
C       -2.596065    0.953626   -2.804175
C       -1.966955   -0.704227   -0.433823
C       -7.412702    1.536843   -0.843126
C       -6.073690    1.017985   -3.502405
C       -7.188234   -1.274562   -1.964774
H       -4.298160   -1.690152   -3.521953
H       -4.045603   -2.695610   -2.090636
H       -2.672258   -2.228263   -3.096661
```

H	-1.834539	0.470942	-3.426091
H	-2.070098	1.752371	-2.267727
H	-3.321230	1.411892	-3.480055
H	-1.626684	0.194947	0.084893
H	-1.115789	-1.089348	-1.005929
H	-2.228771	-1.448464	0.318646
H	-6.985841	2.544505	-0.847256
H	-7.567616	1.190825	0.181957
H	-8.388148	1.598028	-1.332418
H	-5.552880	1.976761	-3.549852
H	-7.063501	1.162183	-3.948535
H	-5.528229	0.315442	-4.138621
H	-8.103099	-1.110016	-2.544098
H	-7.479840	-1.719876	-1.013113
H	-6.580513	-2.002883	-2.508189
K	-7.418980	3.607505	1.610015
N	-5.501658	5.056702	0.408292
Si	-4.585584	5.663719	1.679341
Si	-6.241861	5.743859	-0.933103
C	-4.353442	7.533238	1.744618
C	-5.324411	5.214851	3.370769
C	-2.831756	4.943903	1.721165
C	-8.128448	5.523341	-0.899075
C	-5.970208	7.586560	-1.220816
C	-5.698291	4.907257	-2.542725
H	-5.311729	8.056013	1.808887
H	-3.826242	7.910238	0.864581
H	-3.765751	7.808822	2.626742
H	-4.703161	5.628476	4.171672
H	-5.366015	4.135493	3.550734
H	-6.326218	5.639147	3.509315
H	-2.288783	5.142195	0.789813
H	-2.815369	3.866958	1.917467
H	-2.251221	5.406246	2.526056
H	-8.576605	5.980457	-0.008905
H	-8.441743	4.474445	-0.952104
H	-8.583561	6.015212	-1.764929
H	-6.356889	8.192278	-0.397378
H	-6.483569	7.902656	-2.135216
H	-4.909133	7.822813	-1.339259
H	-6.206725	5.354849	-3.402663
H	-5.950337	3.842946	-2.563073
H	-4.622623	5.017460	-2.722140
C	-6.597650	-0.277135	2.141311
C	-7.434742	-1.269952	1.613913
C	-7.215267	0.670654	2.976339
C	-8.800459	-1.279987	1.842014
H	-7.005894	-2.054617	1.009070
C	-8.585811	0.658754	3.212690
H	-6.621214	1.409331	3.498120
C	-9.397123	-0.304254	2.630783
H	-9.404412	-2.062181	1.395382
H	-9.014528	1.400538	3.879672
H	-10.465804	-0.310644	2.809021
C	-4.456777	-1.589479	1.900668
C	-3.644629	-1.937186	2.982577
C	-4.610929	-2.528558	0.877276
C	-3.008064	-3.170104	3.034663
H	-3.502482	-1.241525	3.799981
C	-3.994166	-3.769103	0.936365
H	-5.190866	-2.263801	0.003528
C	-3.183567	-4.096153	2.014979
H	-2.377514	-3.409805	3.883840
H	-4.131239	-4.474842	0.124612
H	-2.689624	-5.060087	2.058751

1c_diphenyldifluoroethene_b3pw91.log

SCF (RB3PW91) = -739.238187404
E(SCF)+ZPE(0 K)= -739.039012
H(298 K)= -739.025264
G(298 K)= -739.080106
Lowest Frequency = 22.3672cm-1

C	0.261162	-0.416985	0.001605
C	0.925221	0.734872	0.000243
C	-1.212936	-0.436192	-0.123399
C	-1.884242	0.339446	-1.069099
C	-1.957554	-1.278413	0.705252
C	-3.266930	0.287120	-1.170889
H	-1.319858	0.975313	-1.740324
C	-3.338937	-1.328361	0.603569
H	-1.446684	-1.895665	1.434821
C	-3.999626	-0.544097	-0.334042
H	-3.772295	0.892731	-1.914525
H	-3.901901	-1.982813	1.259232
H	-5.079552	-0.586042	-0.416436
C	0.980915	-1.703439	0.127171
C	1.992380	-1.896823	1.068627
C	0.617713	-2.770914	-0.697016
C	2.635776	-3.121803	1.170831
H	2.266179	-1.089728	1.737115
C	1.262347	-3.993618	-0.594685
H	-0.175500	-2.636915	-1.423105
C	2.275634	-4.173588	0.338908
H	3.416184	-3.256425	1.911109
H	0.971892	-4.809581	-1.246561
H	2.777080	-5.130875	0.422083
F	2.238884	0.862905	0.040350
F	0.377015	1.935481	-0.041471

2a_diphenyltrifluoroethane_f_b3pw91.log

SCF (RB3PW91) = -1435.18970611
E(SCF)+ZPE(0 K)= -1435.023946
H(298 K)= -1435.004283
G(298 K)= -1435.071879
Lowest Frequency = 30.4015cm-1

C	-1.094556	0.657846	0.187040
H	-0.711593	-0.363552	0.171732
C	-0.609807	1.201399	-1.155703
C	-2.602060	0.548396	0.211922
C	-3.235195	-0.664017	-0.043358
C	-3.450522	1.610623	0.506893
C	-4.603069	-0.842270	-0.012493
C	-4.825313	1.504843	0.555736
C	-5.369835	0.264510	0.290407
H	-5.047974	-1.806315	-0.217917
H	-5.443230	2.361043	0.789705
C	-0.518393	1.282449	1.439296
C	-0.443180	0.479035	2.576023
C	-0.066469	2.585339	1.617370
C	0.033467	0.894973	3.800240
C	0.428911	3.069084	2.813526
C	0.464872	2.203301	3.885229
H	0.065581	0.223703	4.647466
H	0.769929	4.092137	2.895539
F	0.715581	1.406088	-1.159355
F	-0.868560	0.290491	-2.115881
F	-1.202268	2.334468	-1.549029
F	-2.912288	2.808897	0.763752
F	-2.476231	-1.729964	-0.334557
F	-6.702725	0.129548	0.327981
F	-0.099899	3.439688	0.589532
F	-0.870341	-0.789698	2.474116
F	0.937366	2.649300	5.057938

2b_TS-H_parsep_f_b3pw91.log

SCF (RB3PW91) = -4381.81431327
E(SCF)+ZPE(0 K)= -4381.197821
H(298 K)= -4381.139869
G(298 K)= -4381.289275
Lowest Frequency = -1563.0972cm-1

C	-4.576433	0.861200	2.068533
C	-5.179645	-0.429526	1.636499

H	-5.019254	-0.228617	0.252618
F	-3.301866	0.958744	1.620465
F	-5.229100	1.934414	1.524189
F	-4.487846	1.142581	3.389424
K	-4.035599	2.945038	-0.692335
N	-4.953733	0.231246	-1.067532
Si	-3.576675	-0.195302	-1.993535
Si	-6.483599	0.502012	-1.782578
C	-3.700699	-1.881600	-2.821200
C	-3.191641	0.968227	-3.446148
C	-1.981812	-0.070205	-0.991138
C	-7.482701	1.737702	-0.757129
C	-6.426056	1.250891	-3.513643
C	-7.565484	-1.022512	-2.002573
H	-4.473285	-1.851166	-3.596397
H	-3.967842	-2.675207	-2.121810
H	-2.760335	-2.157992	-3.309415
H	-2.175318	0.740981	-3.787373
H	-3.204843	2.039420	-3.219578
H	-3.859394	0.817342	-4.295023
H	-1.679303	0.972757	-0.843598
H	-1.173889	-0.542007	-1.560581
H	-2.019247	-0.542459	-0.012444
H	-6.981834	2.709720	-0.722025
H	-7.637631	1.362557	0.255663
H	-8.465309	1.897558	-1.209418
H	-5.771054	2.117911	-3.610611
H	-7.437746	1.569630	-3.786682
H	-6.108331	0.511987	-4.253989
H	-8.358870	-0.808856	-2.726660
H	-8.048586	-1.342106	-1.079266
H	-6.980686	-1.862968	-2.386234
K	-7.253260	3.812455	1.828806
N	-5.320570	5.094886	0.469907
Si	-4.233639	5.565201	1.660386
Si	-6.107333	5.879081	-0.788177
C	-3.855731	7.407378	1.785782
C	-4.819555	5.067404	3.396644
C	-2.543389	4.720460	1.486593
C	-7.996352	5.764911	-0.623987
C	-5.743833	7.713698	-1.019710
C	-5.734867	5.082099	-2.465766
H	-4.761987	7.992175	1.966215
H	-3.388741	7.790056	0.874590
H	-3.167583	7.596837	2.616417
H	-4.074504	5.366250	4.140962
H	-4.942568	3.985281	3.513690
H	-5.753357	5.566838	3.682490
H	-2.088204	4.907697	0.506953
H	-2.583426	3.639507	1.658255
H	-1.847429	5.115835	2.233459
H	-8.354853	6.221214	0.306464
H	-8.370416	4.736238	-0.681052
H	-8.482271	6.305967	-1.442386
H	-6.024180	8.300503	-0.141136
H	-6.306891	8.104519	-1.873973
H	-4.682540	7.891264	-1.214372
H	-6.267797	5.603162	-3.267582
H	-6.063671	4.039587	-2.512358
H	-4.669200	5.127203	-2.718597
C	-6.648083	-0.632164	1.839795
C	-7.222241	-1.786073	1.288195
C	-7.570363	0.148561	2.535820
C	-8.549327	-2.146335	1.393427
C	-8.917476	-0.132431	2.663157
C	-9.382972	-1.291327	2.082495
H	-8.913717	-3.055014	0.934055
H	-9.568734	0.529520	3.217983
C	-4.419324	-1.580781	2.259074
C	-4.530503	-1.895923	3.616705
C	-3.622250	-2.467629	1.542015
C	-3.873203	-2.937768	4.240919
C	-2.930024	-3.528927	2.095714
C	-3.067922	-3.731656	3.451494
H	-3.995132	-3.120999	5.299860
H	-2.322986	-4.177699	1.479178
F	-3.534101	-2.325958	0.218441
F	-5.348869	-1.160387	4.385217
F	-2.408219	-4.752557	4.023571
F	-6.426252	-2.614642	0.597909
F	-7.157234	1.284199	3.149575
F	-10.686853	-1.597953	2.192491

2c_diphenyldifluoroethene_f_b3pw91.log

SCF (RB3PW91) = -1334.69566223
E(SCF)+ZPE(0 K)= -1334.545586
H(298 K)= -1334.526737
G(298 K)= -1334.592976
Lowest Frequency = 31.6264cm-1

C	0.263435	-0.412965	-0.001895
C	0.929471	0.736348	-0.005505
C	-1.208653	-0.422464	-0.081702
C	-1.917308	0.161978	-1.128314
C	-1.984721	-1.074490	0.873699
C	-3.292458	0.125924	-1.237799
C	-3.360561	-1.153648	0.823945
C	-3.983241	-0.540215	-0.245365
H	-3.800549	0.592423	-2.070713
H	-3.921623	-1.664598	1.594471
C	0.989526	-1.693364	0.082420
C	1.846901	-2.013301	1.131961
C	0.813603	-2.693316	-0.870959
C	2.500797	-3.223064	1.246430
C	1.430531	-3.925447	-0.816188
C	2.269897	-4.156665	0.255980
H	3.155930	-3.429351	2.081787
H	1.269096	-4.668882	-1.584815
F	2.236831	0.845915	0.000494
F	0.375685	1.925557	-0.015409
F	-1.228003	0.781295	-2.094751
F	-1.358215	-1.649677	1.909205
F	-5.321005	-0.594151	-0.322798
F	2.037490	-1.104272	2.096369
F	0.005316	-2.439348	-1.909123
F	2.889240	-5.343357	0.338401

3a_diphenyltrifluoroethane_me_b3pw91.log

SCF (RB3PW91) = -1075.65408466
E(SCF)+ZPE(0 K)= -1075.273516
H(298 K)= -1075.249085
G(298 K)= -1075.327099
Lowest Frequency = 22.9847cm-1

C	-1.129189	0.768587	0.142638
H	-0.696125	-0.232463	0.130940
C	-0.723822	1.237252	-1.247584
C	-2.645251	0.561074	0.202505
C	-3.181775	-0.686163	-0.169979
C	-3.522477	1.569668	0.649794
C	-4.557792	-0.897035	-0.083328
C	-4.887130	1.310093	0.712169
C	-5.431906	0.083920	0.352031
H	-4.949422	-1.869382	-0.365103
H	-5.545895	2.100292	1.058746
C	-0.492493	1.401065	1.385280
C	-0.523712	0.567303	2.529595
C	0.103991	2.669380	1.506683
C	-0.019250	1.017256	3.741809
C	0.606507	3.067880	2.749292
C	0.551786	2.275115	3.880684
H	-0.068391	0.355548	4.600859
H	1.052752	4.054368	2.823155
F	0.601165	1.445391	-1.354516
F	-1.017121	0.282426	-2.158093
F	-1.347580	2.343434	-1.692889

C	-1.072318	-0.829861	2.503431
H	-2.129395	-0.858377	2.235363
H	-0.540580	-1.461004	1.786076
H	-0.961354	-1.290766	3.484855
C	0.278897	3.686552	0.413020
H	1.164864	3.472563	-0.188629
H	-0.563297	3.756866	-0.266085
H	0.421143	4.671797	0.857814
C	1.083967	2.745425	5.199482
H	0.285588	2.809942	5.944592
H	1.833444	2.052664	5.592143
H	1.544534	3.730563	5.112239
C	-2.376242	-1.850886	-0.676788
H	-2.852700	-2.785291	-0.376209
H	-2.331933	-1.844831	-1.768658
H	-1.348550	-1.877690	-0.319284
C	-3.078378	2.934679	1.084648
H	-2.535777	2.901485	2.030832
H	-2.428418	3.411193	0.354004
H	-3.948494	3.577960	1.220447
C	-6.908647	-0.156342	0.427371
H	-7.150779	-1.203208	0.238389
H	-7.303912	0.114916	1.409749
H	-7.440947	0.450249	-0.311589

3b_TS-H_parsep_me_b3pw91.log

E(SCF)+ZPE(0 K)= -4021.436569
H(298 K)= -4021.374701
G(298 K)= -4021.529602
Lowest Frequency = -1652.7494cm-1

C	-2.98383700	-0.27695100	1.76764100
C	-4.36550600	-0.79847200	1.60563200
H	-4.52673700	-0.40073100	0.18474100
F	-2.08816000	-1.07377500	1.15194400
F	-2.79246500	0.96825900	1.21898100
F	-2.47552700	-0.15919700	3.03747900
K	-4.50648200	2.82875900	-0.07652700
N	-4.65059800	0.18340500	-1.06785500
Si	-3.35578400	0.18559300	-2.21197700
Si	-6.22977800	0.27470500	-1.74232700
C	-3.64255100	-1.00048800	-3.65116100
C	-3.05944300	1.90826300	-2.94524200
C	-1.64270900	-0.25294800	-1.58423600
C	-7.37969800	1.18756100	-0.55275200
C	-6.39797200	1.35318000	-3.28695400
C	-6.96686000	-1.37159000	-2.29183500
H	-4.52117600	-0.75397200	-4.25024400
H	-3.76921200	-2.02254400	-3.28291600
H	-2.77589000	-0.99551200	-4.32035700
H	-2.37120400	1.82032800	-3.79262700
H	-2.56283600	2.57710900	-2.23188900
H	-3.95841800	2.40120700	-3.31550500
H	-1.29561400	0.40710500	-0.78888100
H	-0.96694600	-0.12176600	-2.43696800
H	-1.54320300	-1.28199100	-1.24216700
H	-7.16099300	2.25302300	-0.67191900
H	-7.24236300	0.89739500	0.48738200
H	-8.42787200	1.03931900	-0.82813600
H	-6.17861000	2.40656300	-3.09951300
H	-7.44480800	1.29965500	-3.60790800
H	-5.78589600	1.02714800	-4.12932900
H	-6.61947900	-1.59038800	-3.30591700
H	-8.05970500	-1.31674500	-2.32768900
H	-6.69331100	-2.21927400	-1.66495800
K	-8.34620900	3.57172900	1.30375300
N	-6.30809400	4.97264600	0.31849400
Si	-5.54942100	5.75936500	1.60044000
Si	-6.69406000	5.55781100	-1.21495200
C	-5.53670900	7.64462600	1.57846100
C	-6.36856600	5.29050300	3.24136100
C	-3.71924000	5.29337600	1.79530100
C	-8.19524300	4.65656900	-1.94002200
C	-7.18107700	7.37848800	-1.33003400
C	-5.28405300	5.35080000	-2.45858300
H	-6.54402000	8.06144700	1.50100100
H	-4.94608500	8.03856300	0.74697500
H	-5.09126800	8.02090800	2.50573000
H	-5.81135900	5.69664600	4.09127600
H	-6.40836800	4.20717600	3.38726300
H	-7.38669300	5.69158700	3.31399300
H	-3.16235500	5.46638100	0.86740500
H	-3.54909900	4.26216900	2.11905500
H	-3.25466000	5.92185400	2.56203800
H	-9.11237500	4.89122900	-1.38537600
H	-8.08839300	3.57068400	-1.98290400
H	-8.36690000	4.99414800	-2.96737700
H	-7.97988000	7.62407400	-0.62363900
H	-7.55121800	7.59978500	-2.33708000
H	-6.34276600	8.04998600	-1.13244000
H	-5.55055200	5.76480400	-3.43668100
H	-5.00301900	4.30844000	-2.63254600
H	-4.39070800	5.88044800	-2.11090300
C	-5.54345800	-0.11338400	2.25600100
C	-6.80058800	-0.74117700	2.02171800
C	-5.56686200	1.03951000	3.08315600
C	-7.96428600	-0.29186900	2.63009200
C	-6.77470500	1.46373700	3.65392700
C	-7.98371400	0.81292800	3.47025200
H	-8.89124600	-0.81446000	2.41282400
H	-6.74446500	2.32479800	4.31445700
C	-4.31958600	-2.29085800	1.96941400
C	-4.52110000	-2.71348300	3.30620600
C	-4.08947900	-3.28758500	1.00320800
C	-4.51704100	-4.07040900	3.61763800
C	-4.08996400	-4.63488400	1.36269800
C	-4.30784000	-5.05665400	2.66304500
H	-4.67708900	-4.36171300	4.65179200
H	-3.92233500	-5.37533200	0.58591600
C	-4.76461100	-1.78438700	4.46367700
H	-4.15350600	-0.88783600	4.42081500
H	-5.80873400	-1.46468100	4.50904800
H	-4.53743400	-2.30042500	5.39838400
C	-4.31888700	-6.50939800	3.03176200
H	-3.58147600	-6.72558900	3.81011900
H	-5.29509200	-6.81109700	3.42313700
H	-4.09360500	-7.13853600	2.16905600
C	-3.85294100	-2.99326400	-0.44595100
H	-2.78222800	-2.94907100	-0.66090000
H	-4.27183500	-3.78786200	-1.06688800
H	-4.28164900	-2.04541400	-0.75644300
C	-6.96885300	-1.89545700	1.08076400
H	-6.21848200	-1.88697700	0.29830100
H	-6.88452300	-2.85596100	1.59493600
H	-7.95571200	-1.85137500	0.61679400
C	-4.38067400	1.84217100	3.54966300
H	-3.84483000	1.31853400	4.34516100
H	-3.64609500	2.06786900	2.78590700
H	-4.72594500	2.78843300	3.96528300
C	-9.24360900	1.27800700	4.13851000
H	-10.09306600	1.27218200	3.44937000
H	-9.51236000	0.62042000	4.97122700
H	-9.13167700	2.28508800	4.54647000

3c_diphenyldifluoroethene_me_b3pw91.log

SCF (RB3PW91) = -975.174127093
E(SCF)+ZPE(0 K)= -974.809757
H(298 K)= -974.786198
G(298 K)= -974.861209
Lowest Frequency = 37.6877cm-1

C	0.256860	-0.426150	-0.003748
C	0.921794	0.722666	-0.008340
C	-1.228714	-0.415335	-0.074227
C	-1.900131	0.223569	-1.130080
C	-1.977459	-1.104428	0.901523

C	-3.291880	0.159400	-1.190534
C	-3.361082	-1.138606	0.800256
C	-4.042974	-0.514188	-0.240794
H	-3.797644	0.647656	-2.017927
H	-3.925068	-1.665607	1.563923
C	1.006179	-1.708606	0.073125
C	1.896364	-1.966349	1.129154
C	0.778390	-2.707284	-0.895307
C	2.531329	-3.205971	1.197483
C	1.435138	-3.925035	-0.786080
C	2.317737	-4.199272	0.255172
H	3.207400	-3.397034	2.025223
H	1.255307	-4.682103	-1.543358
F	2.235922	0.850384	-0.060246
F	0.378149	1.926124	0.038419
C	-1.195863	0.965865	-2.226990
H	-1.720954	0.827132	-3.173784
H	-1.176076	2.040110	-2.022193
H	-0.163712	0.642583	-2.357470
C	-5.538159	-0.570004	-0.320110
H	-5.995417	-0.076367	0.542551
H	-5.906341	-0.080113	-1.222665
H	-5.894961	-1.603657	-0.323579
C	-1.330146	-1.795893	2.063881
H	-0.824960	-2.714689	1.757328
H	-0.581979	-1.163675	2.543248
H	-2.080397	-2.059455	2.810513
C	-0.144432	-2.495609	-2.057841
H	-1.192315	-2.507512	-1.749505
H	0.035357	-1.536518	-2.544865
H	-0.004066	-3.283821	-2.798695
C	2.193812	-0.978360	2.218078
H	2.338180	-1.496128	3.168053
H	3.115294	-0.428649	2.005684
H	1.401101	-0.242158	2.346678
C	3.016944	-5.521763	0.339718
H	3.729881	-5.640400	-0.482011
H	3.566736	-5.621109	1.276834
H	2.306997	-6.350105	0.269879

4a_diphenyltrifluoroethane_ome_b3pw91.log

SCF (RB3PW91) = -1526.92989127
E(SCF)+ZPE(0 K)= -1526.520609
H(298 K)= -1526.490210
G(298 K)= -1526.581068
Lowest Frequency = 23.2115cm-1

C	-0.992993	0.927515	-0.118927
H	-0.629212	-0.098773	-0.191085
C	-0.575608	1.483713	-1.473383
C	-2.499824	0.793482	-0.017145
C	-3.105769	-0.441563	-0.270110
C	-3.330217	1.846401	0.396109
C	-4.479947	-0.636460	-0.116743
C	-4.699573	1.675323	0.555899
C	-5.265344	0.431465	0.296828
H	-4.920260	-1.598354	-0.322761
H	-5.340380	2.485020	0.872449
C	-0.335239	1.481773	1.131715
C	-0.261274	0.603337	2.220007
C	0.160835	2.779156	1.331854
C	0.261549	0.977582	3.457934
C	0.690801	3.173108	2.556175
C	0.734067	2.272988	3.613639
H	0.294979	0.271831	4.271716
H	1.075509	4.170602	2.708506
F	0.758037	1.651404	-1.568519
F	-0.901413	0.585193	-2.439108
F	-1.158748	2.627343	-1.852808
O	-0.740685	-0.642551	1.997604
O	1.260688	2.744049	4.769101
O	0.106490	3.619796	0.283241
O	-2.287670	-1.439989	-0.670387
O	-6.604932	0.348516	0.472252
O	-2.718373	3.021850	0.630843
C	-2.826752	-2.734688	-0.850362
H	-1.986626	-3.373049	-1.119757
H	-3.283464	-3.110538	0.070223
H	-3.563677	-2.754774	-1.659018
C	-0.730116	-1.581246	3.054251
H	-1.335710	-1.239604	3.899180
H	-1.166818	-2.491843	2.647189
H	0.287896	-1.792930	3.395572
C	1.335756	1.859556	5.872491
H	1.785389	2.427977	6.685220
H	0.342581	1.517493	6.179915
H	1.966135	0.993416	5.648426
C	0.554511	4.948562	0.444155
H	-0.002947	5.470838	1.227603
H	1.624066	4.988433	0.674079
H	0.376551	5.440947	-0.510813
C	-3.484414	4.111324	1.100715
H	-4.246201	4.410143	0.374059
H	-3.963360	3.882309	2.057770
H	-2.781806	4.931345	1.239020
C	-7.234530	-0.893413	0.213159
H	-6.854095	-1.680110	0.871977
H	-8.294224	-0.741774	0.412657
H	-7.105279	-1.197146	-0.830239

4b_TS-H_parsep_ome_b3pw91.log

SCF (RB3PW91) = -4473.54640609
E(SCF)+ZPE(0 K)= -4472.685924
H(298 K)= -4472.617952
G(298 K)= -4472.786724
Lowest Frequency = -1644.9126cm-1

C	-4.381499	1.096205	1.910011
C	-5.080224	-0.146036	1.526049
H	-5.158413	0.049437	0.083516
F	-3.132251	1.171316	1.371065
F	-4.998048	2.226969	1.436478
F	-4.160200	1.337010	3.236009
K	-4.066863	2.970608	-0.957909
N	-5.393384	0.414669	-1.211079
Si	-4.406105	-0.438150	-2.317938
Si	-6.919444	1.020549	-1.678488
C	-4.289647	-2.282520	-1.970735
C	-4.959782	-0.430000	-4.127378
C	-2.709446	0.396809	-2.414450
C	-7.553389	2.313668	-0.459632
C	-6.905206	1.967784	-3.323413
C	-8.281263	-0.256917	-1.900032
H	-5.188084	-2.758914	-2.375705
H	-4.236933	-2.513369	-0.910245
H	-3.429921	-2.729607	-2.479752
H	-4.278273	-1.089650	-4.677864
H	-4.912380	0.553516	-4.597387
H	-5.969714	-0.825407	-4.264174
H	-2.796221	1.316921	-3.005949
H	-1.971427	-0.222073	-2.932865
H	-2.303687	0.646640	-1.431794
H	-6.911292	3.199407	-0.473260
H	-7.616163	1.910119	0.549781
H	-8.552613	2.638743	-0.762640
H	-5.976118	2.508054	-3.522912
H	-7.717003	2.702644	-3.322473
H	-7.073989	1.300809	-4.170644
H	-9.159019	0.200492	-2.369500
H	-8.596018	-0.687465	-0.948873
H	-7.942955	-1.070144	-2.548904
K	-6.716951	4.200043	2.052736
N	-4.853740	5.311404	0.394129
Si	-3.549274	5.616226	1.403948
Si	-5.716731	6.228995	-0.711587
C	-2.950435	7.403207	1.509330

C	-3.906725	5.130581	3.204379
C	-2.007314	4.613071	0.941073
C	-7.564195	6.319192	-0.278884
C	-5.190538	8.026203	-0.945332
C	-5.671823	5.474000	-2.446562
H	-3.745335	8.075922	1.843441
H	-2.586698	7.770758	0.546261
H	-2.126434	7.482230	2.226701
H	-3.024133	5.314178	3.825622
H	-4.143989	4.068503	3.320328
H	-4.717753	5.728051	3.638499
H	-1.702033	4.795288	-0.095847
H	-2.138282	3.536362	1.087220
H	-1.161662	4.902722	1.573434
H	-7.730022	6.789925	0.697375
H	-8.052176	5.338302	-0.284026
H	-8.100179	6.928271	-1.014229
H	-5.273174	8.601301	-0.019345
H	-5.827126	8.509861	-1.694163
H	-4.156475	8.101693	-1.293030
H	-6.235816	6.086312	-3.157670
H	-6.126661	4.480103	-2.473170
H	-4.648511	5.401691	-2.832848
C	-6.557241	-0.241691	1.783182
C	-7.225023	-1.351189	1.214083
C	-7.375547	0.591987	2.553283
C	-8.583429	-1.597605	1.399533
C	-8.742067	0.396912	2.709258
C	-9.351957	-0.705542	2.136614
H	-9.036518	-2.462504	0.942736
H	-9.324711	1.101491	3.289141
C	-4.321386	-1.349831	2.056528
C	-4.751499	-2.020379	3.222819
C	-3.203089	-1.920000	1.425685
C	-4.231567	-3.239877	3.636583
C	-2.649833	-3.138112	1.836061
C	-3.186858	-3.807183	2.924015
H	-4.597673	-3.736655	4.523784
H	-1.816731	-3.559316	1.299466
O	-6.845744	1.700341	3.190435
O	-6.457937	-2.196804	0.493904
O	-10.688398	-0.840627	2.334261
O	-2.661812	-1.251621	0.380368
O	-5.703262	-1.405291	3.967109
O	-2.729458	-5.002532	3.375328
C	-1.648163	-5.598960	2.684700
H	-0.754230	-4.967758	2.716336
H	-1.440677	-6.536814	3.198236
H	-1.905631	-5.810516	1.641935
C	-6.536796	-2.190199	4.794010
H	-6.924569	-3.059395	4.255193
H	-6.018558	-2.525790	5.698212
H	-7.369322	-1.549150	5.082826
C	-1.458996	-1.751581	-0.166054
H	-1.603321	-2.719129	-0.656221
H	-1.132053	-1.028295	-0.906741
H	-0.684564	-1.846512	0.601266
C	-7.028439	-3.391250	0.010472
H	-7.439048	-3.996144	0.825731
H	-7.812418	-3.199417	-0.728808
H	-6.218346	-3.940935	-0.463832
C	-11.334164	-1.940535	1.721441
H	-10.946327	-2.893845	2.094853
H	-12.388146	-1.858353	1.983305
H	-11.231468	-1.909089	0.632016
C	-6.807945	1.614917	4.612272
H	-7.813693	1.522924	5.031638
H	-6.196969	0.768263	4.925491
H	-6.352655	2.537308	4.974725

4c_diphenyldifluoroethene_ome_b3pw91.log

SCF (RB3PW91) = -1426.43751414
E(SCF)+ZPE(0 K)= -1426.044196
H(298 K)= -1426.014513
G(298 K)= -1426.104584
Lowest Frequency = 22.5454cm-1

C	0.349544	-0.266451	0.000575
C	1.013781	0.879317	-0.001034
C	-1.123012	-0.271534	0.128321
C	-1.947593	0.368674	-0.802739
C	-1.743570	-1.012123	1.147128
C	-3.339407	0.279398	-0.731790
C	-3.125119	-1.121367	1.230540
C	-3.914094	-0.470743	0.286311
H	-3.954241	0.780319	-1.461606
H	-3.607845	-1.684768	2.015505
C	1.075352	-1.547808	-0.125933
C	2.045101	-1.942350	0.801826
C	0.737217	-2.457207	-1.140952
C	2.661242	-3.193619	0.730002
C	1.330526	-3.709544	-1.224940
C	2.292206	-4.067557	-0.284654
H	3.406612	-3.476376	1.455242
H	1.078643	-4.409791	-2.007785
F	2.312496	1.014651	-0.210321
F	0.484986	2.073669	0.206198
O	2.336481	-1.055249	1.775934
O	-0.186670	-2.027102	-2.025985
O	2.829237	-5.301342	-0.437132
O	-0.910488	-1.593542	2.035785
O	-1.323634	1.059905	-1.779309
O	-5.251536	-0.619043	0.438960
C	-6.103162	0.028374	-0.488998
H	-5.933575	-0.332989	-1.508034
H	-7.120242	-0.217718	-0.187702
H	-5.973093	1.114609	-0.460808
C	-1.463195	-2.432829	3.030175
H	-2.118654	-1.876798	3.707698
H	-2.018544	-3.265355	2.587474
H	-0.617658	-2.825456	3.592963
C	-2.108156	1.669827	-2.785004
H	-2.771022	2.435672	-2.370462
H	-1.403827	2.141260	-3.468782
H	-2.701731	0.931970	-3.333699
C	-0.637698	-2.924876	-3.020602
H	0.171365	-3.214558	-3.698374
H	-1.081385	-3.822132	-2.578359
H	-1.400245	-2.388296	-3.583146
C	3.266332	-1.425204	2.774614
H	4.258449	-1.613015	2.352344
H	3.324809	-0.578328	3.456847
H	2.931950	-2.308505	3.327519
C	3.812270	-5.718878	0.492816
H	3.411306	-5.754280	1.510580
H	4.106695	-6.722611	0.189943
H	4.689322	-5.064749	0.469029

17. References

1. G. K. S. Prakash, F. Paknia, T. Mathew, G. Mloston, J. P. Joschek, G. A. Olah, *Org. Lett.* **2011**, 4128–4131. DOI: https://doi.org/10.1021/ol201669a.
2. M. Li, K. Takada, J. I. Goldsmith, S. Bernhard, *Inorg. Chem.* **2016**, 518–526. DOI: https://doi.org/10.1021/acs.inorgchem.5b01709.
3. W. Dmowski, Z. Urbańczyk-Lipkowska, D. Wójcik, *J. Fluorine Chem.* **2009**, 509–511. DOI: https://doi.org/10.1016/j.jfluchem.2009.02.011.
4. P. F. Conzen, E. D. Kharasch, S. F. A. Czerner, A. A. Artru, F. M. Reichle, P. Michalowski, G. A. Rooke, G. Alec, B. M. Weiss, T. J. Ebert, *Anesthesiology* **2002**, 578–584. DOI: https://doi.org/10.1097/00000542-200209000-00010.
5. L. Jin, M. R. Davies, E. D. Kharasch, G. A. Doss, T. A. Baille, *Chem. Res. Toxicol.* **1996**, 555–561. DOI: 10.1021/tx950162m.
6. C. Patel, E. André-Joyaux, J. A. Leitch, X. Martínez de Irujo-Labalde, F. Ibba, J. Struijs, M. A. Ellwanger, R. Paton, D. L. Browne, G. Pupo, S. Aldridge, M. A. Hayward, V. Gouverneur, *Science* **2023**, 302–306. DOI: https://doi.org/10.1126/science.adi1557.
7. X. Kong, Y. Chen, Q. Liu, W. Wang, S. Zhang, X. Chen, Y.-Q. Xu, Z.-Y. Cao, *Org. Lett.* **2023**, 581–586. DOI: https://doi.org/10.1021/acs.orglett.2c03956.
8. Z. Lu, T. Kumon, G. B. Hammond, T. Umemoto, *Angew. Chem. Int. Ed.* **2021**, 16171–16177. DOI: https://doi.org/10.1002/anie.202104975.
9. S. T. Keaveney, F. Schoenebeck, *Angew. Chem. Int. Ed.* **2018**, 4073–4077. DOI: https://doi.org/10.1002/anie.201800644.
10. A. V. R. D. Lisboa, G. Duran-Camacho, A. K. Ehrlacher, M. R. Lasky, M. S. Sanford, *Org. Lett.* **2023**, 9025–9029. DOI: https://doi.org/10.1021/acs.orglett.3c03706.
11. H. Hattori, K. Ishida, Y. Ogiwara, N. Sakai, *Eur. J. Org. Chem.* **2022**, e202201118. DOI: https://doi.org/10.1002/ejoc.202201118.
12. E. M. Mahmoud, S. Mori, Y. Sumii, N. Shibata, *Org. Lett.* **2023**, 2810–2814. DOI: https://doi.org/10.1021/acs.orglett.3c00701.
13. S. B. Munoz, H. Dang, X. Ispizua-Rodriguez, T. Mathew, G. K. S. Prakash, *Org. Lett.* **2019**, 1659–1663. DOI: https://doi.org/10.1021/acs.orglett.9b00197.
14. Z. Zou, W. Chang, W. Zhang, S. Ni, Y. Pan, Y. Liang, D. Pan, Y. Wang, *J. Fluorine Chem.* **2023**, 110114. DOI: https://doi.org/10.1016/j.jfluchem.2023.110114.

15. A. Hayatifar, E.A. Elifritz, M. B. Bloom, K. M. Pixley, C. J. Fennella, C. S. Weinert, *Dalton Trans*. **2021**, 4490–4493. DOI: https://doi.org/10.1039/D1DT00754H.
16. P. Tang, N. Mankad, *Org. Lett*. **2024**, 3299–3303. DOI: https://doi.org/10.1021/acs.orglett.4c00967.
17. A. M. Żurański, S. S. Gandhi, A. G. Doyle, *J. Am. Chem. Soc*. **2023**, 7898–7909. DOI: https://doi.org/10.1021/jacs.2c13093.
18. I. Klose, C. Patel, A. Mondal, A. Schwarz, G. Pupo, V. Gouverneur, *Nature* **2024**, 359–364. DOI: https://doi.org/10.1038/s41586-024-08125-1.
19. P. Švec, P. Novák, M. Nádvorník, Z. Padělková, I. Císařová, L. Kolářová, A. Růžička, J. Holeček, *J. Flourine Chem*. **2007**, 1390–1395. DOI: https://doi.org/10.1016/j.jfluchem.2007.07.001.
20. W. W. Wilson, R. Haiges, K. O. Christe, *J. Fluorine Chem*. **2023**, 110166. DOI: https://doi.org/10.1016/j.jfluchem.2023.110166.
21. N. Oguri, N. Takeda, M. Unno, *Chem. Lett*. **2015**, 1506–1508. DOI: https://doi.org/10.1246/cl.150692.
22. P. Kläring, A.-K. Jungton, T. Braun, C. Müller, *Eur. J. Inorg. Chem*. **2012**, 1430–1436. DOI: https://doi.org/10.1002/ejic.201100917.
23. Y.-L. Huang, Q.-Q. Zhang, C.-Y. Wang, Y. Zhao, X.-S. Wang, *Org. Lett*. **2024**, 5776–5781. DOI: https://doi.org/10.1021/acs.orglett.4c01953.
24. N. Fey, M. Garland, J. P. Hopewell, C. L. McMullin, S. Mastroianni, A. G. Orpen, P. G. Pringle, *Angew. Chem. Int. Ed*. **2012**, 118 –122. DOI: https://doi.org/10.1002/anie.201105954.
25. C. Brown, M. Murray, R. Schmutzler, *J. Chem. Soc. C* **1970**, 878–881. DOI: https://doi.org/10.1039/J39700000878.
26. J. Ren, F.-H. Du, M.-C. Jia, Z.-N. Hu, Z. Chen, C. Zhang, *Angew. Chem. Int. Ed*. **2021**, 24171 – 24178. DOI: https://doi.org 10.1002/anie.202108589.
27. O. V. Dolomanov, L. J. Bourhis, R. J. Gildea, J. A. K. Howard, H. Puschmann, *J. Appl. Crystallogr*. **2009**, 339–341. DOI: https://doi.org/10.1107/S0021889808042726.
28. G. M. Sheldrick, *Acta Crystallogr. Sect. A Found. Adv*. **2015**, 71, 3–8. DOI: https://doi.org/10.1107/S2053273314026370.
29. G. M. Sheldrick, *Acta Crystallogr. Sect. C Struct. Chem*. **2015**, 3–8. DOI: https://doi.org/10.1107/S2053229614024218.
30. Gaussian 16, Revision C.01, M. J. Frisch, G. W. Trucks, H. B. Schlegel, G. E. Scuseria, M. A. Robb, J. R. Cheeseman, G. Scalmani, V. Barone, G. A. Petersson, H. Nakatsuji, X. Li, M.

Caricato, A. V. Marenich, J. Bloino, B. G. Janesko, R. Gomperts, B. Mennucci, H. P. Hratchian, J. V. Ortiz, A. F. Izmaylov, J. L. Sonnenberg, D. Williams-Young, F. Ding, F. Lipparini, F. Egidi, J. Goings, B. Peng, A. Petrone, T. Henderson, D. Ranasinghe, V. G. Zakrzewski, J. Gao, N. Rega, G. Zheng, W. Liang, M. Hada, M. Ehara, K. Toyota, R. Fukuda, J. Hasegawa, M. Ishida, T. Nakajima, Y. Honda, O. Kitao, H. Nakai, T. Vreven, K. Throssell, J. A. Montgomery, Jr., J. E. Peralta, F. Ogliaro, M. J. Bearpark, J. J. Heyd, E. N. Brothers, K. N. Kudin, V. N. Staroverov, T. A. Keith, R. Kobayashi, J. Normand, K. Raghavachari, A. P. Rendell, J. C. Burant, S. S. Iyengar, J. Tomasi, M. Cossi, J. M. Millam, M. Klene, C. Adamo, R. Cammi, J. W. Ochterski, R. L. Martin, K. Morokuma, O. Farkas, J. B. Foresman, and D. J. Fox, Gaussian, Inc., Wallingford CT, **2016**.

31. A. D. Becke, *J. Chem. Phys.* **1993**, 5648–5652. DOI: https://doi.org/10.1063/1.464913.
32. S. Grimme, J. Antony, S. Ehrlich, H. Krieg, *J. Chem. Phys.* **2010**, 154104. DOI: https://doi.org/10.1063/1.3382344.
33. A. D. Becke, E. R. Johnson, *J. Chem. Phys.* **2005**, 154101. DOI: https://doi.org/10.1063/1.2065267.
34. E. R. Johnson, A. D. Becke, *J. Chem. Phys.* **2005**, 024101. DOI: https://doi.org/10.1063/1.1949201.
35. E. R. Johnson, A. D. Becke, *J. Chem. Phys.* **2006**, 174104. DOI: https://doi.org/10.1063/1.2190220.
36. S. Grimme, S. Ehrlich, L. Goerigk, *J. Comput. Chem.* **2011**, 1456. DOI: https://doi.org/10.1002/jcc.21759.
37. C. Lee, W. Yang, R. G. Parr, *Phys. Rev. B* **1988**, 785–789. DOI: https://doi.org/10.1103/physrevb.37.785.
38. S. H. Vosko, L. Wilk, M. Nusair, *Can. J. Phys.* **1980**, 1200–1211. DOI: https://doi.org/10.1139/p80-159.
39. P. J. Stephens, F. J. Devlin, C. F. Chabalowski, M. J. Frisch, *J. Phys. Chem.* **1994**, 11623–11627. DOI: https://doi.org/10.1021/j100096a001.
40. Y. Zhao, N. E. Schultz, D. G. Truhlar, *J. Chem. Theory Comput.* **2006**, 364–382. DOI: https://doi.org/10.1021/ct0502763y.
41. C. Adamo, V. Barone, *J. Chem. Phys.* **1999**, 6158–6170. DOI: https://doi.org/10.1063/1.478522.
42. J.-D. Chai, M. Head-Gordon, *J. Chem. Phys.* **2008**, 084106. DOI: https://doi.org/10.1063/1.2834918.

43. J.-D. Chai, M. Head-Gordon, *Phys. Chem. Chem. Phys.* **2008**, 6615–6620. DOI: https://doi.org/10.1039/B810189B.
44. Y. Zhao, D. G. Truhlar, *J. Chem. Phys.* **2006**, 1941011. DOI: https://doi.org/10.1063/1.2370993.
45. A. D. Becke, *Phys. Rev. A* **1988**, 3098–3100. DOI: https://doi.org/10.1103/PhysRevA.38.3098.
46. J. P. Perdew, *Phys. Rev. B* **1986**, 8822–8824. DOI: https://doi.org/10.1103/PhysRevB.33.8822.
47. F. Weigend, R. Ahlrichs, *Phys. Chem. Chem. Phys.* **2005**, 3297–3305. DOI: https://doi.org/10.1039/B508541A.
48. F. Weigend, *Phys. Chem. Chem. Phys.* **2006**, 1057–1065. DOI: https://doi.org/10.1039/B515623H.
49. F. Weigend, F. Furche, R. Ahlrichs, *J. Chem. Phys.* **2003**, 12753–12762. DOI: https://doi.org/10.1063/1.1627293.
50. D. Rappoport, F. Furche, *J. Chem. Phys.* **2010**, 134105. DOI: https://doi.org/10.1063/1.3484283.
51. A. V. Marenich, C. J. Cramer, D. G. Truhlar, *J. Phys. Chem. B* **2009**, 6378–6396. DOI: https://doi.org/10.1021/jp810292n.
52. D. Feller, *J. Comput. Chem.* **1996**, 1571–1586. DOI: https://doi.org/10.1002/(SICI)1096-987X(199610)17:13%3C1571::AID-JCC9%3E3.0.CO;2-P.
53. K. L. Schuchardt, B. T. Didier, T. Elsethagen, L. Sun, V. Gurumoorthi, J. Chase, J. Li, T. L. Windus, *J. Chem. Inf. Model.* **2007**, 1045–1052. DOI: https://doi.org/10.1021/ci600510j.
54. B. P. Pritchard, D. Altarawy, B. Didier, T. D. Gibson, T. L. Windus, *J. Chem. Inf. Model.* **2019**, 4814–4820. DOI: https://doi.org/10.1021/acs.jcim.9b00725.
55. G. Luchini, J. V. Alegre-Requena, I. Funes-Ardoiz, R. S. Paton, GoodVibes: Automated Thermochemistry for Heterogeneous Computational Chemistry Data. *F1000Research*, **2020**, 291. DOI: https://doi.org/10.12688/f1000research.22758.1.
56. NBO 7.0. E. D. Glendening, J. K. Badenhoop, A. E. Reed, J. E. Carpenter, J. A. Bohmann, C. M. Morales, P. Karafiloglou, C. R. Landis, F. Weinhold, **2018**, Theoretical Chemistry Institute, University of Wisconsin, Madison.
57. E. D. Glendening, C. R. Landis, F. Weinhold, *J. Comput. Chem.* **2019**, 2234–2241. DOI: https://doi.org/10.1002/jcc.25873.
58. A. E. Reed, R. B. Weinstock, and F. Weinhold, *J. Chem. Phys.* **1985**, 735–746. DOI: https://doi.org/10.1063/1.449486.

59. J. A. Spivey, D. B. Collum, *J. Am. Chem. Soc.* **2024**, 17827–17837. DOI: https://doi.org/10.1021/jacs.4c03418.
60. A. I. Ojeda-Amador, A. J. Martínez-Martínez, G. M. Robertson, S. D. Robertson, A. R. Kennedy, C. T. O'Hara, *Dalton Trans.* **2017**, *46*, 6392–6403. DOI: https://doi.org/10.1039/C7DT01118K.
61. X. Cai, T. Lei, D. Sun, L. Lin, RSC Adv., **2017**, *7*, 15382-15389. DOI: https://doi.org/10.1039/C7RA01267E
62. S. M. Purushothaman, M. F. Tronco, M. Poncot, C. Chakraborty, N. Guigo, M. Malfois, N. Kalarikkal, S. Thomas, I. Royaud, D. Rouxel, *ACS Appl. Polym. Mater.*, **2024**, *6*, 8291-8305. DIO: https://doi.org/ 10.1021/acsapm.4c01157
63. N. O. Andrella, N. Xu, B. M. Gabidullin, C. Ehm and R. T. Baker, *J. Am. Chem. Soc.*, **2019**, *141*, 11506–11521. DOI: 10.1021/jacs.9b03101
64. A. Y. Jordan and T. Y. Meyer, *J. Organomet. Chem.*, **1999**, *591*, 104–113. DOI: https://doi.org/10.1016/S0022-328X(99)00423-4.
65. J. T. Welch and J. Lin, *Tetrahedron*, **1996**, *52*, 291–304. DOI: https://doi.org/10.1016/0040-4020(95)00912-R.
66. S. M. Huber, T. Steinke, P. Wonner and E. Engelage, *Synthesis*, **2021**, *53*, 2043–2050. DOI: https://doi.org/10.1055/a-1372-6309.
67. M. B. Röthel, A. Schöler, F. Buß, P. Löwe and F. Dielmann, *Chem. Eur. J.*, **2024**, *30*, e202402028. DOI: https://doi.org/10.1002/chem.202402028.
